# Comparing Kaolin and Pinolene to Improve Sustainable Grapevine Production during Drought

**DOI:** 10.1371/journal.pone.0156631

**Published:** 2016-06-13

**Authors:** Luca Brillante, Nicola Belfiore, Federica Gaiotti, Lorenzo Lovat, Luigi Sansone, Stefano Poni, Diego Tomasi

**Affiliations:** 1 Council for Agricultural Research and Economics, Viticulture Research Center, CREA-VIT, Conegliano, Italy; 2 Università Cattolica del Sacro Cuore, Dipartimento di Scienze delle produzioni vegetali sostenibili, Piacenza, Italy; UC Davis MIND Institute, UNITED STATES

## Abstract

Viticulture is widely practiced in dry regions, where the grapevine is greatly exposed to water stress. Optimizing plant water use efficiency (WUE) without affecting crop yield, grape and wine quality is crucial to limiting use of water for irrigation and to significantly improving viticulture sustainability. This study examines the use in vineyards of particle film technology (engineered kaolin) and compares it to a film-forming antitranspirant (pinolene), traditionally used to limit leaf water loss, and to an untreated control. The trial was carried out under field conditions over three growing seasons, during which moderate to very severe plant water stress (down to -1.9 MPa) was measured through stem water potential. Leaf stomatal conductance (g_s_) and photosynthesis rate (A_n_) were measured during the seasons and used to compute intrinsic WUE (WUEi, defined as A_n_/g_s_ ratio). Leaf temperature was also recorded and compared between treatments. Bunch quantity, bunch and berry weight, sugar accumulation, anthocyanin and flavonoid contents were measured. Finally, microvinifications were performed and resultant wines subjected to sensory evaluation.Results showed that the use of kaolin increased grapevine intrinsic WUE (+18% on average as compared to unsprayed vines) without affecting berry and bunch weight and quantity, or sugar level. Anthocyanin content increased (+35%) in kaolin treatment, and the wine was judged more attractive (p-value <0.05) and slightly more appreciated (p-value < 0.1) than control. Pinolene did not increase WUEi, limiting An more than g_s_; grapes with this treatment contained lower sugar and anthocyanin content than control, and the obtained wine was the least appreciated. This study demonstrates that particle film technology can improve vine WUE_i_ and wine quality at the same time, while traditional antitranspirants were not as effective for these purposes. This positive effect can be used in interaction with other already-demonstrated uses of particle film technology, such as pest control and sunburn reduction, in order to achieve more sustainable vineyard management.

## Introduction

World climate is changing and becoming warmer [1,2], with great effects on agricultural production, whose products are directly impacted by meteorological conditions. At the same time, world population is increasing and will reach 9 billion in 2050 [3]. Agriculture resides at the interface of these two crucial changes, striving to tackle the difficult task of increasing food production while coping with climate change.

Water is one of the critical, non-renewable world resources [4] and agriculture is the largest user of fresh water globally (70% in the World, 48% in Mediterranean Europe [5]). Enormous savings can be achieved by careful management, especially in dry regions, where agricultural water use is high and competition for water use characterizes human activities. Approximately 60% of grapevines are cultivated in semi-arid conditions, and thus water consumption per vine (300–700 mm) is frequently higher than the actual annual average precipitation in many viticultural regions [6]. A moderate water stress has been found useful for red-wine grape quality in several studies (e.g. [7,8]) but severe water stress causes ripening delays, low yields and reduced berry color [9], finally it determines plant death.

Optimizing water management and improving water use efficiency (WUE) in viticulture, and agriculture, is therefore a crucial goal, shared by many researchers worldwide (see reviews by [10–13]). Water use efficiency can be expressed in different ways, the intrinsic or photosynthetic WUE, here abbreviated to WUEi (An/g_s_) is the ratio of the rate of carbon assimilation (An) to the rate of stomatal transpiration (g_s_). It is directly linked to plant WUE, WUEp, defined as the ratio of total plant dry matter to the total plant water consumption [14], even if suitability of WUEi to approximate WUEp has been discussed [15]. Water savings must not be made at expenses of yield and fruit quality in order to secure economical sustainability for producers, and should be obtained by the use of sustainable and/or organic practices to be consistent in scope.

Application of antitranspirants to the canopy was largely studied, in the 1960s and '70s, in order to improve WUEi, but subsequent utilization was limited, probably due to costs and efficiency of the practice [11]. Two types of plant antitranspirants are currently available, and both can be employed in organic viticultural production. A first consists of bio-chemical active compounds such as chitosan (β-1,4-D-glucosamine), whose stomata-closing action is due to modification of abscisic acid metabolism [16]. A second class of compounds includes film-forming polymers (S1A Fig), which are inactive from a biochemical point of view and mechanically limit plant transpiration. Di-1-p-menthene (C_20_H_34_), a therpenic polymer commonly called pinolene, derived from pine resin, belongs to this class [17].

A new, inexpensive technology similar in principle to the latter category of antitranspirants, thus with similar mechanical action on gas exchanges, is currently emerging, known as particle film technology [18,19]. It involves spraying engineered clays, such as kaolin (an aluminum phyllosilicate, Al_2_Si_2_O_5_(OH)_4_), to cover leaves and fruit with thin films of nanoparticles (S1B Fig). The heating process to which kaolin is submitted makes the silicate white, and greatly increases its light reflectance properties. It was originally developed for pest control [20], because the film mechanically hampers pest suction, but the different light signature of the reflected light also causes insect avoidance for many pests [21]. In viticulture, kaolin has been proposed to control the diffusion of Pierce's disease [22]. Kaolin films not only reflects photosynthetically active and ultraviolet radiations, but also infrared, thus lowering temperatures of sprayed organs. This property has been effectively used in horticulture to prevent sunburns of fruits [23,24]. The reduction of photosynthetic photon flux density at the leaf level, can be compensated by a diffusion of these wavelengths in the interior of the canopy, at least in trees with large, three-dimensional canopies, e.g. walnut, almond, or apple trees [25,26]. The increase of light in the inner canopy can increase fruit set with a beneficial effect on yield in the second year of application [27]. Kaolin efficacy in mitigating environmental stresses, and reducing temperature can also affect fruit-quality aspects, such as total soluble solids and anthocyanin concentration, as observed in grapevine [28–30].

Recently, this technology has been proposed as a supplemental tool to save water on several species (e.g.: clementines and tomatoes [18], grapefruit [31]) although there is controversy over its effects on gas exchanges, and mechanisms of action are not yet completely understood. Some authors reported no effect or even an increase in net assimilation (An) and stomatal conductance (g_s_) [27,32], while others observed a reduction [23,33].

Compared to other fruiting species, a minor attention has been devoted to particle film technology on the grapevine, but the practice is currently emerging. Recent studies have addressed pest control [22], sunburn protection [34], fruit quality [28,29] integration within irrigation management [29,35]. However, to our knowledge, in grapevine (as well in other species), effects of particle film technology on plant water stress, leaf gas exchanges, fruit composition and wine characteristics have not been described in a single, comprehensive study, which also compared the effects of a traditional antitranspirant.

In this study we compare particle film technology (kaolin) to an antitranspirant (pinolene) and an unsprayed control, over three growing seasons in a dry-summer climate in southern Italy. The objective was to demonstrate that particle film technology is a valuable method to improve grape composition and wine quality during drought, while also having a beneficial effect on intrinsic water use efficiency, which could help increase sustainability in vineyards.

## Materials and Methods

### Experimental field site

The experiment was carried out in a commercial vineyard located in Casabona (KR), Calabria, South of Italy (39° 12’ 47” N; 16° 59’ 43” E, 46 m a. s. l.). The planted cultivar was Cabernet-Sauvignon (*Vitis vinifera* L.), grafted onto 1103 P (*Vitis berlandieri* Planch x *Vitis Rupestris* Scheele); plants were ten years old. Grapevines were spur pruned, leaving 10–12 buds per vine, and trained to a unilateral cordon with vertical shoot positioning (VSP). Rows were N-S oriented and vine spacing was 2.2 m x 0.9 m (between x in-the row distance) for a resulting vine density of 5050 plants/ha. Vineyard was equipped with a drip irrigation system, with one drip emitter per plant supplying 4L/h. During the experiment period irrigation was activated twice, for a period of 8 hours, in July and August. Soil had a loamy texture and floor management was carried out as full tillage. Meteorological variables were measured with an on site weather station, while historic data are from ARSSA-Calabria. Librandi S.P.A. gave permission to conduct the study in this vineyard, and the study did not involve endangered or protected species.

### Experimental design

The trial ran from 2012 to 2014 and was conducted using a randomized block design, with three blocks composed of 20 vines each selected on three different rows. The three selected rows were separated by two untreated rows in order to limit drift effects. Three treatments were evaluated as: a) untreated control, b) kaolin application (Surround^®^ WP, 95% kaolin, 5% inert ingredients, AgNova Technologies Pty Ltd., Australia), c) pinolene application (Vapor Gard^®^, CBC (Europe) s. r. l, Italy). Treatments were made in the morning and in absence of wind. Kaolin and pinolene were applied at bunch closure (2012-06-26, 2013-06-27, 2014-07-03) and veraison (2012-08-02, 2013-08-02, 2014-08-04). Application doses were 6 L hL^-1^ for kaolin, 2 L hL^-1^ for pinolene. A pneumatic sprayer was used (Nobili, S.p.A., Italy, model Beta), featuring a 4 + 4 spray head with venturi nozzles, a centrifugal fan (Ø 500 mm, flow rate 7500 m^3^ /h), air speed 120 m/s, working pressure 0.25–0.3 MPa. S1 Fig shows an example of pinolene (S1A Fig) and kaolin treatments (S1B Fig).

### Gas exchange, leaf temperature and solar noon stem water potential measurements

During the season, beginning the day after the first application, the solar noon stem water potential (Ψ_stem_) was measured fortnightly by pressure chamber following the procedure described in [36]. Measurements were taken at sun zenith on eight primary leaves per treatment, placed inside plastic bags and sampled from eight random vines. Single leaf gas exchange measures were taken in the morning hours (8:30–10:30) on eight primary leaves, in the same day and on same vines of the Ψ_stem_ measurements, using a portable infra-red gas analyzer (LCA4, ADC BioScientific Ltd., Herts, UK) featuring a broad leaf chamber (6.25 cm^2^). Eight primary leaves per treatment were measured among those inserted at nodes 4–6 above the distal bunch on a main shoot. Assimilation rate (An, μmol CO_2_ m^-2^ s^-1^) and stomatal conductance (g_s_, mol H_2_O m^-2^ s^-1^) were obtained by measurement of inlet and outlet CO_2_ and H_2_O relative concentration. Intrinsic water use efficiency, WUEi, was instead derived as the ratio between An and g_s_ (and then expressed in μmol CO_2_ mol^-1^ H_2_O). Leaf temperature was obtained by the infrared thermometer (accuracy: ± 0.5°C at 25°C) of the same instrument, on the same eight primary leaves and at the same time of photosynthesis and PPFD measurements.

### Yield components and grape composition

At harvest, mean bunch and berry weight were determined on three replicates of 15 bunches and 60 berries randomly sampled from each of the three blocks for all treatments. The number of bunches, and the total yield per vine were also recorded on 3 vines per block. On 5 different dates from veraison to harvest, small portions of bunches (20, to make approx. 1 kg of grapes) were randomly sampled on both sides of the row from all blocks for each treatment and then mixed. Approximately 1 kg of grapes per block was then crushed and processed to follow ripening, by measuring soluble solids (°Bx), determined by refractometry on 2 mL of juice at 20°C (digital refractometer: PR101α, ATAGO Inc., U.S.A.); total soluble solids were then transformed in g L^-1^ of sugars by multiplying for the correspondent specific gravity, as also shown in [37]. A sample of 30 berries per block was stored at -20°C for subsequent measurements of total flavonoids and anthocyanins. Skins from all 30 berries were manually removed from the pulp, and immersed for 4h in 75 mL of a simile wine solution containing 12% v/v ethanol, 2 g L ^−1^ of Na_2_S_2_O_5_, 5 g L ^-1^ of tartaric acid and adjusted to pH 3.20 with NaOH [38]. Samples were homogenized for 1 min with an Ultraturrax T18 (IKA Labortechnik, Staufen, Germany), and the extract was centrifuged for 10 min at 3500 x g and 20°C. The supernatant was then used for analysis after dilution with an ethanolic solution of HCl (70:30:1, ethanol:water:Hcl, v/v). Total flavonoid index was determined by spectrophotometry (Shimadzu Scientific Instruments, Columbia, MD, USA), reading the absorbance at 280 nm, adjusted respect to the tangent to the pick, and at 540 nm for anthocyanins. Flavonoids were expressed as mg kg^-1^ fresh weight of (+)-catechin and anthocyanin as mg kg^-1^ fresh weight of malvidin-3-glucoside [38].

### Microvinification and wine tasting

Sugar content and accumulation in grapes was used to determine the harvest date. In each year, at harvest, approximately 150 kg of grapes, collected from all blocks of each treatment, were crushed and de-stemmed. In steel tanks, musts were inoculated with 20g hl^-1^ of *Saccharomyces cerevisiae* (Zymaflore FX10, Laffort, Bordeaux, France). Sulfur dioxide was added at a dose of 1.5 g hl^-1^ as Na_2_S_2_O_5_. Skins were macerated over 4 days, with a pumpover each day to keep the cap wet. On the second day, 40 g hL^-1^ of (NH_4_)_3_PO_4_, were added and pH was adjusted with 40 g hL^-1^ of tartaric acid (C_4_H_6_O_6_) in 2012 and 2014, 90 g hL^-1^ in 2013 to reduce risks of bacterial contamination. Very basic data about finished wines are in S1 Table. Alcoholic fermentation lasted ten days, at room temperature (23–24°C); wines were then gravity-settled. Malolactic fermentation was not inoculated, but always concluded by end of November. Wines were bottled in January and subjected to sensory analysis after 6–7 months.

A tasting panel composed of 6 experienced judges tasted and evaluated the wines each vintage using a scale from 1 to 9, with 1 as the lowest value. Wines were evaluated for visual and overall preference, and also for two olfactory descriptors which classically characterize ripe Cabernet-Sauvignon wines: vegetal (presence of pyrazines) and fruity. These descriptors also allowed the differentiation of the ripeness level of the grapes used to produce the wines. 50 mL wine samples were served at 18°C, in standard coded ISO (1977) wine tasting glasses. Random codes identified each sample and the tasting order varied across judges.

### Statistics

Data were subjected to linear mixed model analysis of variance, where fixed effects were the experimental treatments, and vintage and date included as nested random effects (or vintage alone when analysis were not repeated throughout the vintage i.e. harvest data). Single date or vintage analysis were made with one way ANOVA. Multiple comparisons between effects were investigated through Tukey's all-pairwise comparisons. The word “significant” is used to indicate a p-value ≤ 0.05 as a result of a statistical test, when different it is directly specified. Analysis was performed in R v. 3.2.4 [39], using nlme and multcomp packages [40,41]. Data and R code are in S1 File.

## Results and Discussion

### Meteorological conditions in the vintages of study

Meteorological data for all growing seasons are shown in Table 1, and compared to the historical mean recorded between 1985–2010. Considering data in the growing season from 1^st^ April to 30^th^ September, minimum temperature averages were lower than the historical mean, in 2013 and 2014 (respectively -0.6°C and -0.8°C), and higher in 2012 (+1.1°C). Mean temperatures were always higher than the historical ones, +1.9°C in 2012 and 2013, +1°C in 2014, as well as the average maximum temperatures, +2.7°C in 2012, +5.6°C in 2013, +4.6°C in 2014. In all seasons, maximum temperatures reached values higher than 40°C in summer, while maximum temperatures exceeded 30°C in approximately two months. The mean photosynthetic active radiation (PAR) registered from veraison to harvest was weakest in 2014 and highest in 2013.

**Table 1:** Meteorological variables in the vintages of studies compared to 25-year historical mean (source: ARSSA Calabria).

METEOROLOGICAL VARIABLE	1985–2010	2012	2013	2014
**Min. Temperature Apr-Oct (°C)**	13.5	14.6	12.9	12.7
**Mean Temperature Apr-Oct (°C)**	20.5	22.4	22.1	21.5
**Max. Temperature Apr-Oct (°C)**	27.5	30.2	33.1	32.1
**Days with max. temperature > 30°C**	NA	59	62	62
**Rainfalls/month Apr-Oct (mm)**	40	35	30	46
**Rainfalls Apr-Oct (%)**	280	243	207	320
**Relative humidity Apr-Oct**	61	67	68	74
**Photosynthetic active radiation (μmol m**^**-2**^ **s**^**-1**^**)**	NA	1587	1648	1469

NA: Not Available

The historical average of annual rainfall is 680 mm and less than half, 280 mm (41%), falls during the vine growing period. Rainfall amounts in the growing period were lower than the average in 2012 and 2013, while they were higher in 2014. Relative humidity was always higher than the historical mean, and registered rainfall amounts were lower than approximate average water consumption by the vines, or at best in a very low range (between 300–700 mm in the growing season, [13]. S2 Fig shows rainfall and temperature trends in the studied vintages.

### Solar-noon stem water potentials

**Fig 1:**
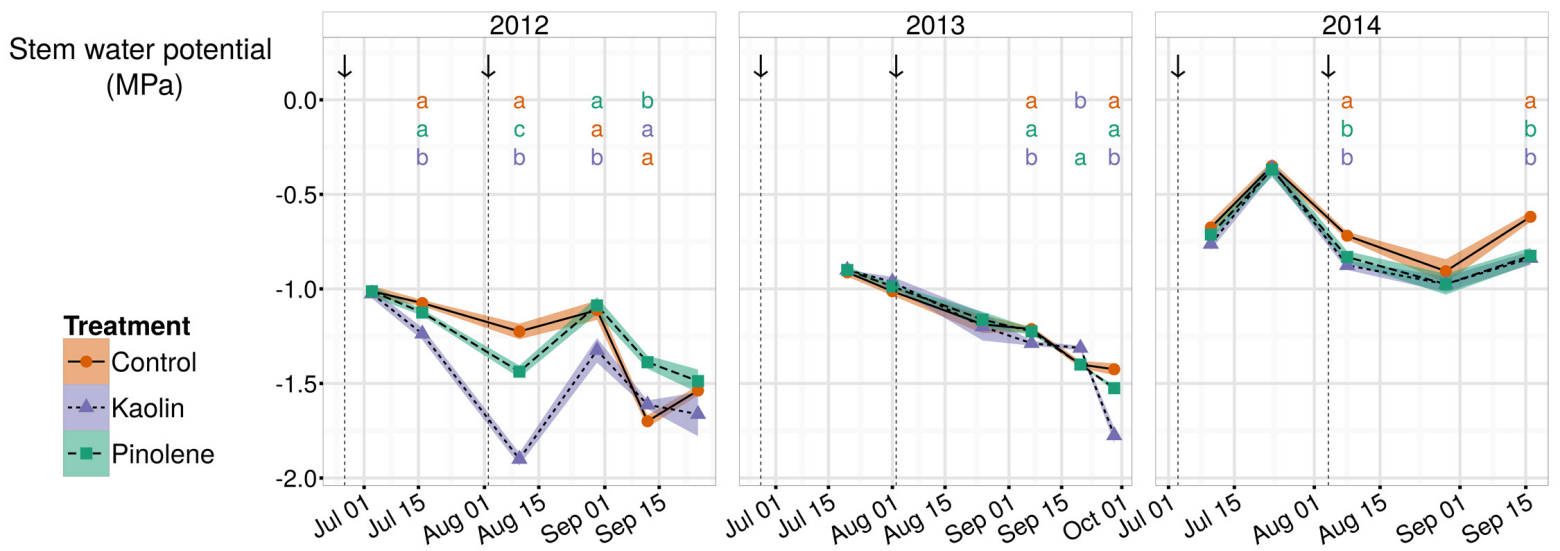
Trends in stem water potentials Ψ_stem_ in all seasons and for all treatments. Trends for stem water potentials (Ψ_stem_, MPa) in the three seasons of study (2012–2014). According to reference values reported in the text, plant water stress was severe in 2012–2013 and moderate/weak in 2014. When ANOVA was significant, different letters indicate differences between treatments with p-value ≤ 0.05 (Tukey all-pair comparisons) for the correspondent date. Arrows indicate product application dates. Shaded regions are mapped to the standard error of the mean; ● and solid line indicate the control, ▲ and short-dashed line indicate kaolin, ■ and dashed line indicate pinolene treatment.

Trends for Ψ_stem_ between bunch closure and harvest in all studied seasons are shown in Fig 1. On average, in 2012 and 2013, vines were in a moderate to severe water stress range, while in 2014 the water stress was weak or occasionally moderate, considering thresholds reported for Cabernet-Sauvignon water stress in [36]. Even if differences between theses were low, as shown in Fig 1, kaolin had the lowest Ψ_stem_ in two of the three seasons; even for data pooled over all seasons, this treatment had significantly lower values than pinolene and control (-1.09 MPa and -1.06 MPa respectively). As already observed in [42–44], this decrease in Ψ_stem_ is caused by a decrease in g_s_, here artificially obtained through kaolin application. It will be discussed in a following section. Pinolene and control did not show significantly different stem water potentials, except for the less-stressed 2014 season, but difference was minimal, as shown in Fig 1.

### Leaf temperature

Analyzing data for all seasons, the mixed model ANOVA reveals 2012 as the season with warmer leaves (44.7 ± 2.4°C), followed by 2013 (40.5 ± 2.1°C) and then 2014 (38.2 ± 2.2°C). The same analysis, reported in Table 2, also shows that pinolene treatment had significantly warmer leaves (1.19°C and 1.43°C higher than control and kaolin, respectively). Summing all data, there was no significant difference between kaolin and control. In dry seasons (2012 and 2013), leaves in the kaolin treatment were 1.30°C cooler than in control, which had a temperature similar to pinolene. On the other hand, in 2014, the temperature was 1.47°C warmer than control for kaolin, and 3.63°C for pinolene. It is also bears noting that differences between seasons were higher in control than in pinolene and kaolin, reflecting the effect of transpiration on temperature.

**Table 2:** Mean leaf temperature recorded for all treatments in the experiment.

LEAF TEMPERATURE (°C)			
Vintages	Control	Kaolin	Pinolene
**All vintages**	40.62 a	40.38 a	41.81 b
**Non stressed vintage (2014)**	36.64 a	38.11 b	40.27 c
**Stressed vintages (2012–2013)**	43.08 a	41.78 b	42.76 a

Different letters indicate significant difference with a p-value <0.05

Kaolin increases the reflection of incident radiation and, as a related effect, it should lower temperatures; on glass plates the reduction has been estimated at about 10% [27]. Conflicting results have been reported on leaves: authors in [23] observed an increase of approximately 1°C on tomato leaves sprayed with kaolin (31.8°C), compared to unsprayed leaves (30.8°C). Authors in [32] reported significant reduction of temperature on apple canopies, using a mixture of 3% (w/v) kaolin (M96-018, Engelhard Corp, Iselin, N.J.) and 4% (v/v) methanol in water [45] applied to runoff. They also showed that relative temperature reduction changes with the hour of the day, increasing towards the solar zenith (13:00–15:00 am), and decreasing in the morning (10:00 am) and in the afternoon (17:00). In our opinion, this suggests an influence of either the PPFD or the relative humidity. Under our conditions, kaolin increased leaf temperature only in the less irradiated season. Generally, kaolin had significantly warmer leaves than control when PPFD was below a threshold of 1500 μmol m^−2^s^−1^. It can be hypothesized that because kaolin acts on leaf temperature by increasing light reflection, its effect is reduced or even annulled when the PPFD is low. Furthermore, if kaolin reduces g_s_ (subject of the following section) by limiting transpiration, it causes a contemporary increase in leaf temperature. Such increase is counterbalanced by strong sunlight reflection when PPFD is high, resulting in a leaf temperature lower than the control; on the contrary, when PPFD is low, sunlight reflection is reduced and appears too low to counteract the heating caused by limited transpiration. This hypothesis is reinforced by the absence of any significant relationship between leaf temperature and relative humidity or Ψ_stem_ in our data.

### Gas exchanges, photosynthesis and water use efficiency

#### Stomatal Conductance

**Fig 2:**
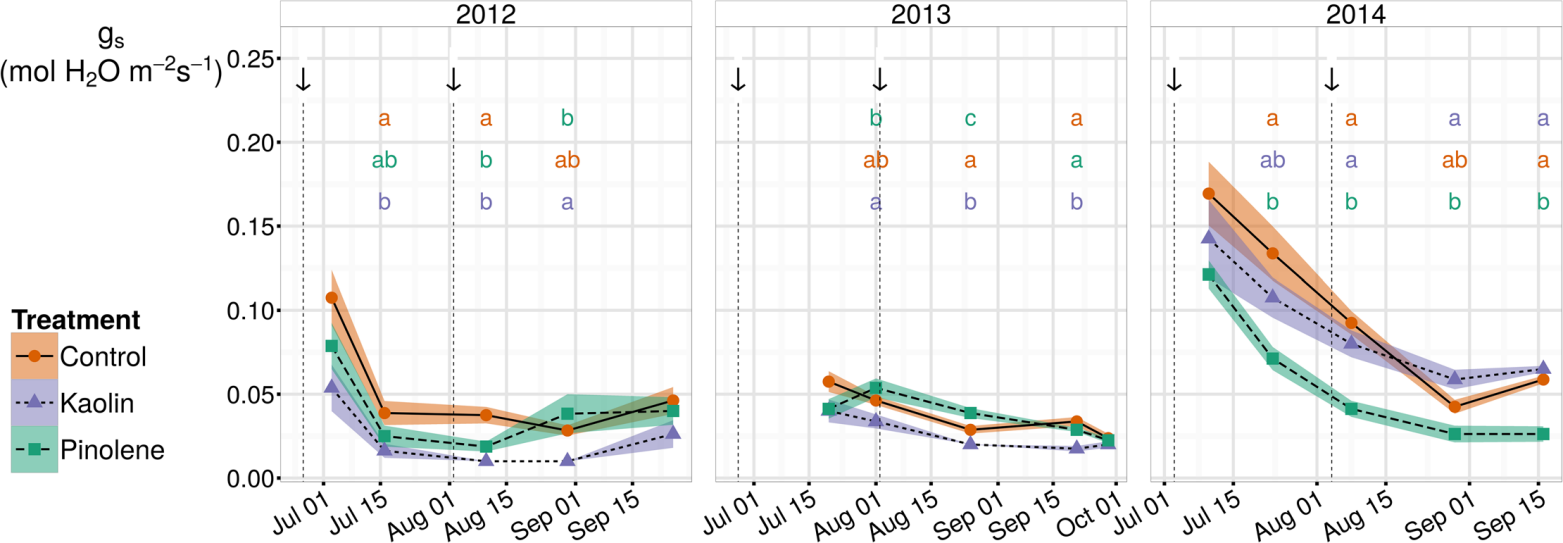
Trends for stomatal conductance (g_s_) in all seasons and for all treatments. Trends for stomatal conductance (g_s_, mol H_2_O m^-2^ s^-1^) in the three seasons of study (2012–2014). Plant water stress as measured by Ψ_stem_ was severe in 2012–2013 and moderate/weak in 2014. The use of kaolin and pinolene allowed decrease of g_s with_ respect to unsprayed control. When ANOVA was significant, different letters indicate differences between treatments with p-value ≤ 0.05 (Tukey all-pair comparisons) for the correspondent date. Arrows indicate product application dates. Shaded region are mapped to the standard error of the mean; ● and solid line indicate the control, ▲ and short-dashed line indicate kaolin, ■ and dashed line indicate pinolene treatment.

Trends in stomatal conductance for all treatments in the three seasons of the experience are shown in Fig 2. Values were generally low because of the hot climate of the experimental site, and the consequent plant water stress. Considering all treatments together, the highest value was 0.169 mol H_2_0 m^-2^ s^-1^, registered by control in 2014 (date mean). The overall mean value was 0.052 mol H_2_0 m^-2^ s^-1^. The mean for control was slightly higher, 0.064 mol H_2_0 m^-2^ s^-1^; generally, control had the greatest g_s_ in the highest amount of observation dates. As for Ψ_stem_, also for this physiological measurement, values indicate a lower vine water stress in 2014, when the mean for g_s_ was equal to 0.083 mol H_2_0 m^-2^ s^-1^, and a greater water stress in 2012 and 2013, when the mean for g_s_ was 0.038 and 0.034 mol H_2_0 m^-2^ s^-1^ respectively. According to [46], these values indicate a moderate water stress in 2014, and a severe water stress in 2012 and 2013.

From Fig 2 it also appears evident that the use of kaolin limited g_s_ in almost all dates, but especially during drought (2012–2013). Effect of kaolin on g_s_ was confirmed by mixed model ANOVA, which indicated significantly lower g_s_ average values in kaolin (- 0.016 mol H_2_0 a m^-2^ s^-1^, thus -25% respect to control). Pinolene and kaolin do not show significant differences if all data are pooled together. However, as observable in Fig 2, kaolin significantly reduces more g_s_ than pinolene if we consider only stressed seasons (2012–2013); conversely, pinolene significantly reduced g_s_ more than kaolin in the non-stressed seasons (2014).

**Fig 3:**
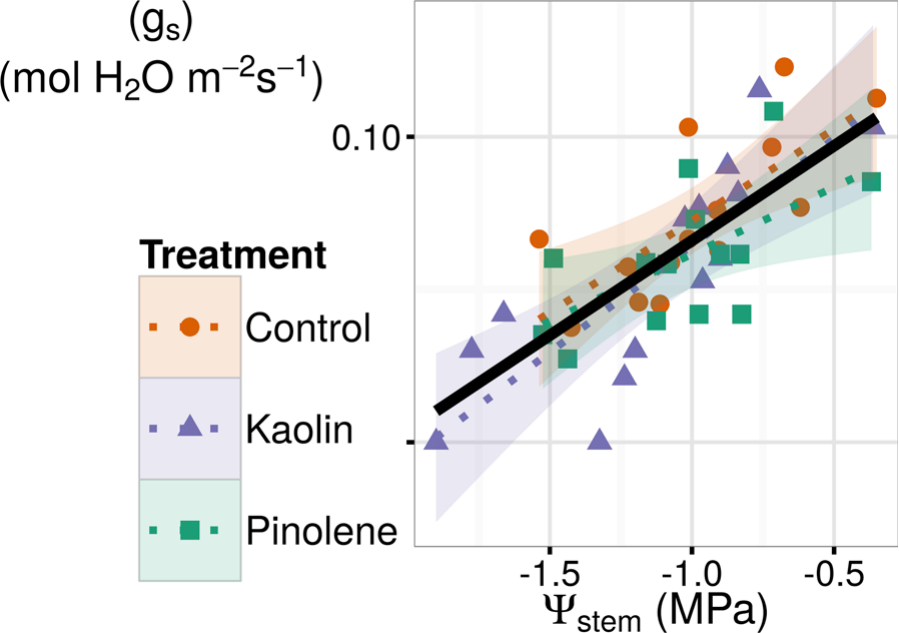
Log-linear relations between Ψ_stem_ and g_s_ in all treatments. The Ψ_stem_ and g_s_ were significantly log-linearly related in all treatments with *r* = 0.77 all groups together, *r* = 0.79 for the test, *r* = 0.82 for kaolin, *r* = 0.65 for pinolene. ANCOVA test indicated equality of slopes. Reduction in g_s_ per unit of Ψ_stem_ increases at high water stress (low Ψ_stem_), but the effect is not caused by the treatment, being equal in the control. Dotted lines are fitted for each group, while the solid line is fitted for all data pooled. Shaded regions are confidence intervals; ● and solid line indicate the control, ▲ and short-dashed line indicate kaolin, ■ and dashed line indicate pinolene treatment.

Considering pinolene, the overall experiment mean was also lower than the control (-0.018 mol H_2_0 m^-2^ s^-1^, thus -28% respect to control), but, as evident from Fig 2, it was principally caused by low values recorded in the non-stressed season (2014). If we consider only the seasons when water stress occurred (2012–2013), the difference between pinolene and control was no longer significant, while that for kaolin remained significant. Kaolin was more effective in stressed seasons (in 2012 and 2013) than in 2014, while for pinolene the effect was the opposite. Similar results were also reported by [29], who observed a reduction of g_s_ by kaolin, with respect to an unsprayed control, only during water stress, while an increase was observed in well-irrigated plants. However, Ψ_stem_ was significantly log-linearly related to g_s_ in all groups, as shown in Fig 3 (*r* = 0.77 all groups together, *r* = 0.79 for the test, *r* = 0.82 for kaolin, *r* = 0.65 for pinolene). This means that the reduction in g_s_ per unit of Ψ_stem_ increases at high water stress (low Ψ_stem_ values) compared to mild water stress, but relation does not vary between treatments. In our data, the slope of the log-linear model fitted the data, and rate of change in g_s_ with Ψ_stem_, is not significantly different across groups when an ANCOVA is performed. This analysis allows the exclusion of a direct interaction of Ψ_stem_ with the treatment in reducing g_s_. The higher efficacy of kaolin during stressed seasons is probably caused by side effects, such as reduced relative humidity, higher PAR, and lower runoff in stressed years than in non-stressed ones.

Pinolene is marketed as an antitranspirant, and its effect on g_s_ reduction has been confirmed in many studies [17,47–49], whereas kaolin is commercially described as porous and not limiting gas exchange. Some authors have found an increase in g_s_ in apple leaves sprayed with kaolin, and also reported microscope photography showing open stomata in sprayed leaves [19,32]. Others have found reductions in g_s_ similar or even greater than the ones presented here (in tomatoes, clementines, and beans) [18,23]. It is possible that the formulation of the chemical plays a role, as suggested by [19], and that the addition of gums increases the anti-transpirant effect. Conversely, loosely-bound formulations, such as the one studied here, should allow the dislocation of particles with stomata movements, then allow stomata opening. However, our results show that even those formulations induce very significant g_s_ limitation, especially during drought. There are two possible reasons, in our opinion: i) stomata opening is still possible but limited by coating ii) resistance of leaf boundary layer could increase, because of an increase in surface roughness, for example.

#### Net leaf photosynthesis

**Fig 4:**
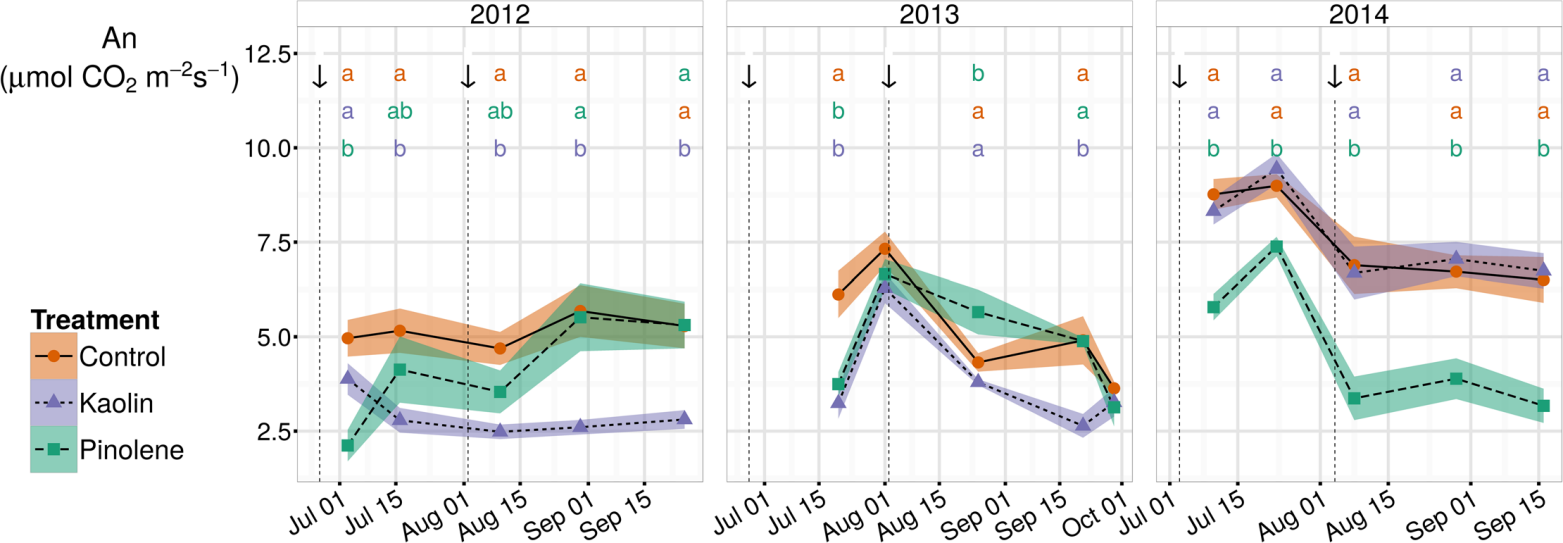
Trends for net photosynthesis (An) in all seasons and for all treatments. Trends for net photosynthesis (An, μmol CO_2_) in the three seasons of study (2012–2014). Plant water stress as measured by Ψ_stem_ was severe in 2012–2013 and moderate/weak in 2014. In pinolene, An stayed equal in all seasons; kaolin reduced An in stressed years, but not in 2014. When ANOVA was significant, different letters indicate differences between treatments with p-value ≤ 0.05 (Tukey all-pair comparisons) for the correspondent date. Arrows indicate product application dates. Shaded regions are mapped to the standard error of the mean; ● and solid line indicate the control, ▲ and short-dashed line indicate kaolin, ■ and dashed line indicate pinolene treatment.

Considering all seasons together, mean A_n_ in the control (6.01 μmol CO_2_ m^2^ s^-1^) was significantly higher than in kaolin (4.89 μmol CO_2_ m^2^ s^-1^) and pinolene (4.45 μmol CO_2_ m^2^ s^-1^), while the reduction in A_n_ by pinolene compared to kaolin was not significant at the defined threshold (p-value < 0.1). Fig 4 shows trends in all studied seasons. As expected, An was significantly higher in 2014, the season with higher Ψ_stem_ and higher stomatal g_s_ (then lower water stress), than in 2012 and 2013. Difference between seasons was not found in pinolene; in this treatment, low values of An were recorded independently of external conditions, such as plant water stress or weather.

Antitranspirants, such as pinolene, have been proposed as an alternative to defoliation in hot climates, to delay ripening and reduce sugars in grapes at harvest [17, 49, 50]. Results reported here show that pinolene is effective in reducing An, and confirm the proposition of these studies. Few studies reported data about leaf sugars in relation to antitranspirants [17, 49], and none of the here cited studies considered leaf carbohydrases. In future studies it will be useful to include those measurements to deepen comprehension about pinolene and kaolin effect on photosynthesis.

#### Intrinsic water use efficiency

**Fig 5:**
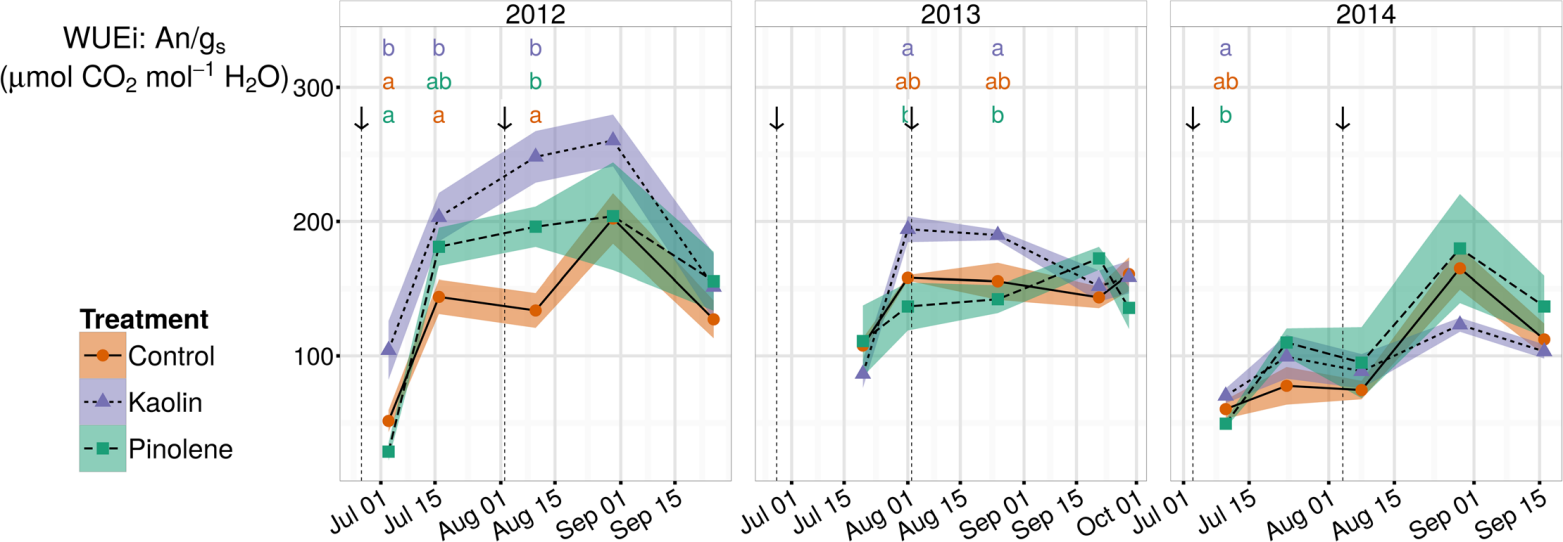
Trends for intrinsic water use efficiency (WUEi) in all seasons and for all treatments. Trends for intrinsic water use efficiency (WUEi, μmol CO_2_ mol^-1^ H_2_O) in the three seasons of study (2012–2014). Plant water stress as measured by Ψ_stem_ was severe in 2012 and moderate/weak in 2014. The use of kaolin increased WUEi, especially during drought (2012–2013). Pinolene did not significantly increase WUEi with respect to control. When ANOVA was significant, different letters indicate differences between treatments with p-value ≤ 0.05 (Tukey all-pair comparisons) for the correspondent date. Arrows indicate product application dates. Shaded regions are mapped to the standard error of the mean; ● and solid line indicate the control, ▲ and short-dashed line indicate kaolin, ■ and dashed line indicate pinolene treatment.

**Fig 6:**
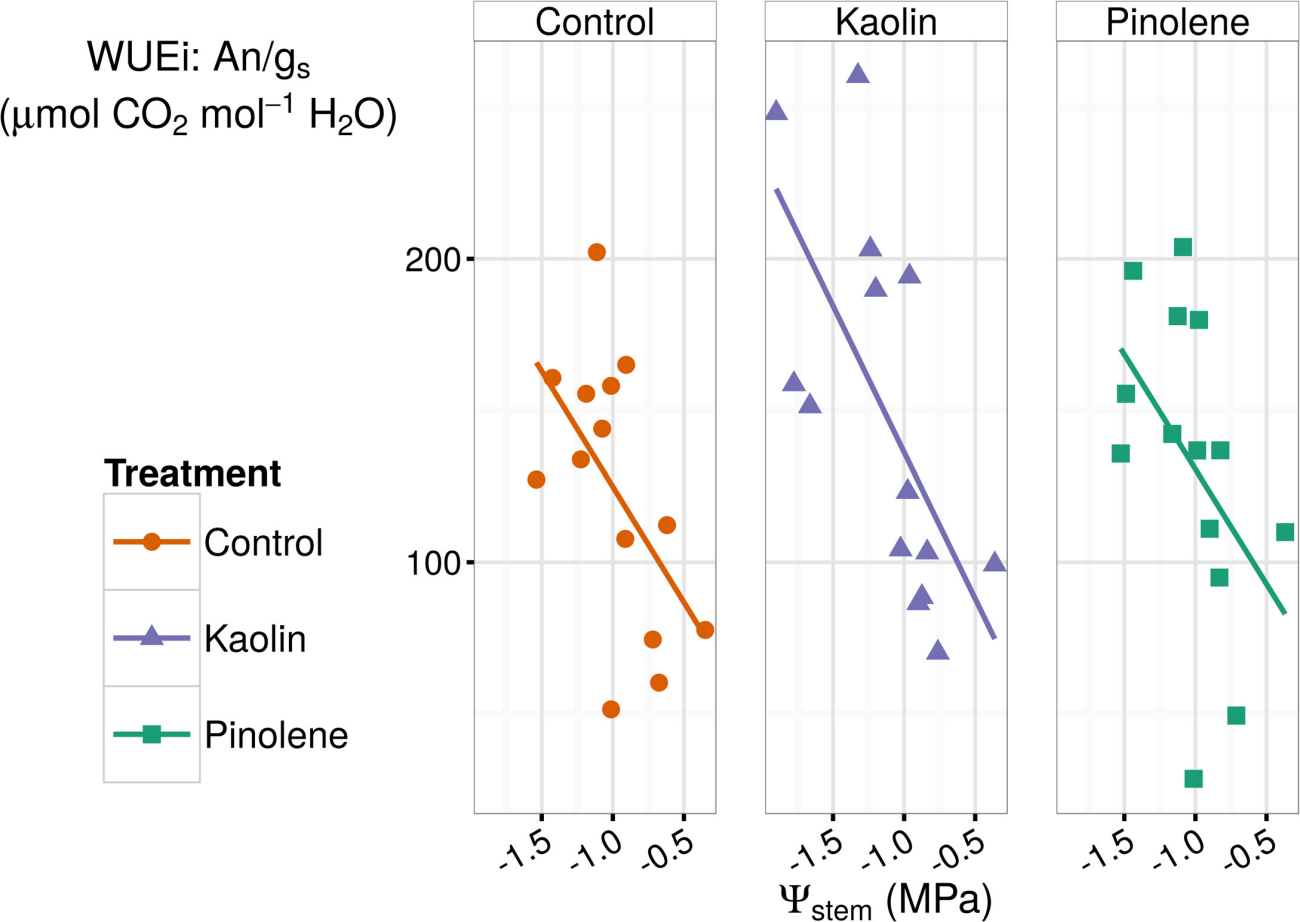
Relations between WUEi and Ψ_stem_ for all treatments. Linear correlations between intrinsic water use efficiency (WUEi, μmol CO_2_ mol^-1^ H_2_O) and stem water potentials (Ψ_stem_, Mpa). Relations were significant for control and kaolin, *(r =* -0.55 for the control, *r* = -0.66 for kaolin, p-value < 0.05), while they were not significant for pinolene (*r =* -0.46, p-value <0.1).

The WUEi (the An/g_s_ ratio) in all seasons is shown in Fig 5. Values show a large variability when compared to values reported in the literature. Among all observed data, the minimum value (28.57 μmol CO_2_ mol^-1^ H_2_O) was registered in the pinolene treatment, while the maximum value in the kaolin treatment (260.30 μmol CO_2_ mol^-1^ H_2_O). For data pooled over seasons, the mean was lower for control (124.9 μmol CO_2_ mol^-1^ H_2_O), followed by pinolene (135.6 μmol CO_2_ mol^-1^ H_2_O), while the highest average was for the kaolin treatment (148.9 μmol CO_2_ mol^-1^ H_2_O). Pooling all data, the difference between kaolin and pinolene was not significant (p-values was found < 0.1), while it was very significant if the year with lower water stress was excluded. In control and kaolin, a significant and negative linear correlation was found between WUEi and Ψ_stem_: the WUEi increased as water stress intensified (*r =* -0.55 for the control, *r* = -0.66 for kaolin), as illustrated in Fig 6. This correlation was not significant for pinolene (*r =* -0.46, p-value <0.1); however, it was significant for data pooled over all treatments (*r* = 0.59).

Results of ANOVA show that kaolin significantly increased morning vine WUEi with respect to the control; all data pooled, it was 22.5 μmol CO_2_ mol^-1^ H_2_O higher. The same analysis did not allow discrimination between pinolene and control; this treatment did not significantly increase WUEi. Even if only stressed years are considered, pinolene and control did not show significant different WUEi. Increase in morning WUEi in kaolin corresponded to an increase of approx. 18% over control mean. Differences in the extremes were even higher: kaolin increased the minimum morning WUEi by 36% and the maximum by 29%. When considering only the seasons with severe water stress (2012–2013), the effect was magnified, reaching an increment of 26% over control mean (+35.8 μmol CO_2_ mol^-1^ H_2_O). Conversely, the effect was reduced and no longer significant when the water stress was weak (2014).

**Fig 7:**
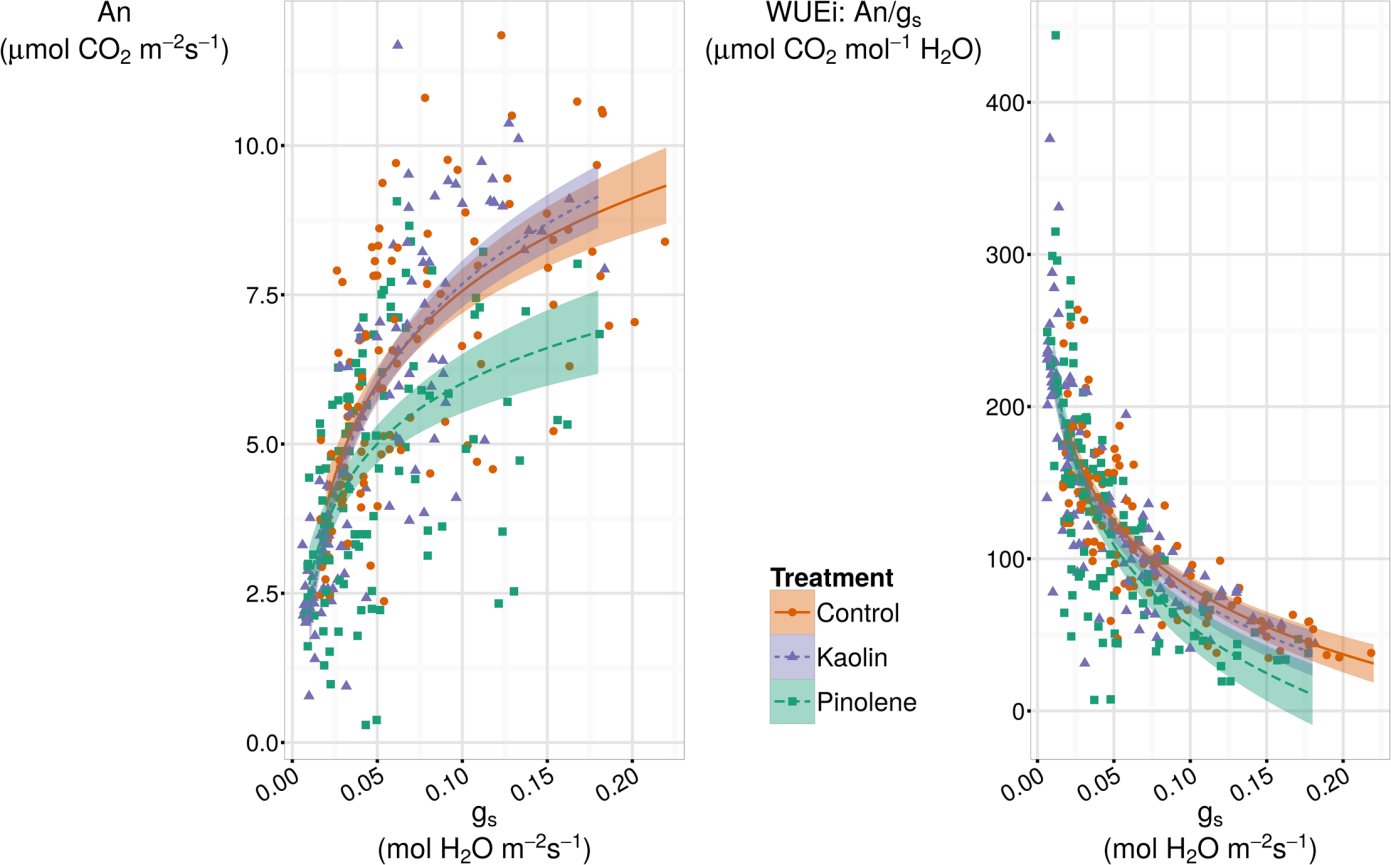
Relations between g_s,_ An, and WUEi over entire experiment. **a)** Relationships between An and g_s_ in all theses. Pinolene has lower An at equivalent g_s_ than kaolin or control treatments. These last two are not different between themselves. **b)** Relationships between intrinsic water use efficiency and g_s_ in all theses. Water use efficiency increases exponentially with decreasing g_s_. Slight jittering was used to prevent over-plotting. Lines are log-linear models fitted to the data (y = log(x)). Shaded regions are confidence intervals at 0.95 level, short-dashed line indicate kaolin, dashed line pinolene, and solid line control treatment.

The positive log-linear relationship between g_s_ and An in all treatments is shown in Fig 7A. In all experimental treatments, the relationship is highly significant, but a significant difference appears evident at a first glance at this plot. While the relationship is similar for control and kaolin treatments, pinolene clearly shows a significantly different pattern (confidence intervals do not overlap). In all groups, A_n_ increases with g_s_, but at a given g_s_ value, An is higher in kaolin and in control than in pinolene, except for very low g_s_ values. Moreover, in kaolin and control, An tends to a plateau at higher values than in pinolene. This difference may explain why pinolene does not significantly increase WUEi with respect to control: pinolene limits An more than g_s_, and the limit in An is reached very soon, at low g_s_ values. Fig 7B shows how this directly translates into a lower WUEi when pinolene is compared to control or kaolin treatment at equivalent g_s_ values.

The increase in WUEi observed for kaolin with respect to control (result obtained in 11/15 dates, when pinolene is excluded from the analysis) is explained not by an improvement of the An assimilation at equal g_s_, which is not significant (Fig 7A), but by reduction in g_s_ caused by kaolin (Fig 2 and corresponding subsection). Kaolin mechanically helps in the reduction of g_s_, which is related to the observed reduction in Ψ_stem_ presented in Fig 1, as already observed in [42,44,51]. Because g_s_ is lower in kaolin than in control, WUEi increases. The increase of WUEi with g_s_ reduction, is exponential at lower g_s_ values (Fig 7B), therefore during drought. Consequently, even the effect of kaolin on WUEi is magnified during drought, because WUEi is more sensitive to slight changes within the lowest range of g_s_ values observed during drought. For the same reason, the effect is reduced, and becomes null at higher g_s_ values, thus in absence of water stress. These data are very interesting and they will merit further attention in future studies, to better evidence if the observed increase in morning WUEi could effectively be related to an increase in whole plant water use efficiency throughout the whole day. In grapevine, leaf WUEi and plant WUE cannot be strictly related [52,53], and therefore the observed effect can be reduced. Meanwhile, it is also probable that the observed increase in WUEi at leaf level will be higher when considering the entire canopy, because kaolin reflectance increases the incident PPFD in the inner canopy leaves and therefore their photosynthesis rate. On almond and walnut, it has been observed that such increase in photosynthetic radiation-use efficiency, at the whole canopy level, counteracts the reduction in carbon assimilation caused by kaolin application and at best can also allow a gain in An [26]. This deserves further investigations in VSP systems commonly used in grapevine cultivation.

According to [6], in normal conditions a decrease in g_s_ always improves WUEi, even if, as shown in Fig 7B, substantial savings can be achieved only during drought. Particle film technology can be investigated and further developed to improve WUEi, by reducing g_s_ without excessively reducing An, and therefore without a decrease in yield or crop quality. However, in viticulture, lower crop is often synonymous with higher wine quality, and a slight reduction in yield can be readily accepted if it also allows an improvement in grape composition and wine.

### Effects on yield components, grape composition and wine preference

Data regarding yield components and grape composition at harvest for all treatments are shown in Table 3. Bunch number per vine was not significantly different between treatments, nor were bunch or berry mean weight. The drought conditions observed in this study limited the potential yield of the control, which was not different to both kaolin and pinolene treatments. However, in several species, an increase of yield was observed post kaolin application, which was mainly related to sunburn reduction [23,32]. Other studies, on apple trees, attributed this positive effect to kaolin efficacy in mitigating environmental stresses, and observed that the magnitude of the increase in yield was correlated to growing season temperature [21,24].

**Table 3:** Yield components and grape composition at harvest for all treatments in all studied vintages

Treatment	Vintage	Soluble Solids (°Bx)	pH	Titratable Acidity (g L^-1^)	Total Flavonoids (mg kg^-1^)	Antho-Cyanins (mg kg^-1^)	Bunch Number per vine	Bunch Weight (g)	Berry Weight (g)	Yield per vine (kg)
**Control**	2012	23.83 a	4.35	2.63	2205.33	751.00	17.33	182.00	1.03	3.17
**Kaolin**		21.57 b	3.72	2.37	2830.67	972.00	19.33	148.33	0.80	2.83
**Pinolene**		21.50 b	4.27	2.47	2124.67	726.67	20.00	177.00	1.03	3.57
**Control**	2013	22.50 b	4.00	2.77	2815.80 a	933.20 a	17.33	95.03 b	0.73	1.59 b
**Kaolin**		22.77 a	3.97	2.80	2779.30 a	1040.13 a	17.00	120.70 a	0.73	2.03 a
**Pinolene**		21.07 c	4.00	2.73	2119.17 b	620.53 b	18.00	108.87 b	0.93	1.99 a
**Control**	2014	19.87 a	3.93	3.77	819.33 a	320.8 a	16.33	195.00	1.40	2.93 a
**Kaolin**		21.00 a	3.92	3.73	820.77 a	405.53 a	13.33	193.33	1.37	2.25 b
**Pinolene**		17.33 b	3.89	4.13	482.57 b	195.63 b	15.73	198.33	1.40	2.90 a
**Control**	All	22.07 a	4.09 a	3.06	1946.82 a	668.33 b	17.00	157.34	1.06	2.56 ab
**Kaolin**		21.78 a	3.87 b	2.97	2143.58 a	805.88 a	16.56	154.12	0.97	2.37 b
**Pinolene**		19.97 b	4.05 a	3.12	1575.47 b	514.28 c	17.91	161.40	1.12	2.82 a

In case of significant ANOVA, different letters indicate significant difference between treatments with a p-value < = 0.05 (Tukey contrasts)

**Fig 8:**
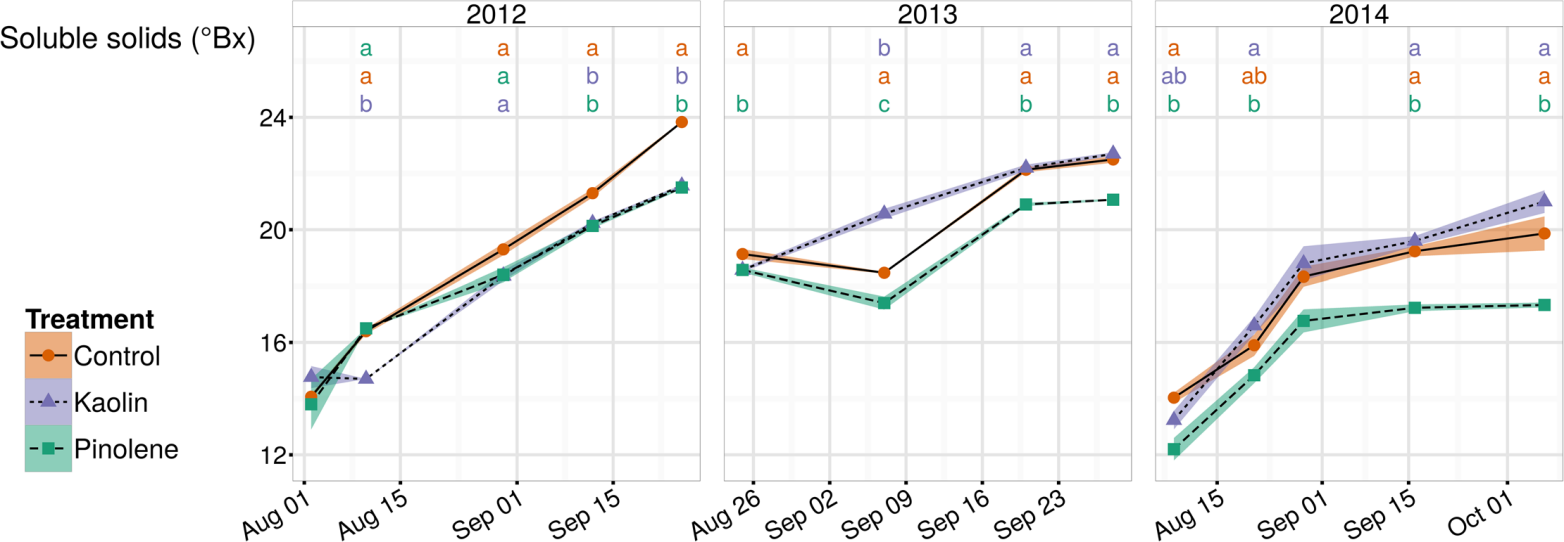
Trends for soluble solids (°Bx) in all seasons and for all treatments. Trends for soluble solids (°Bx), essentially sugars in grapevine must, in the three seasons of study (2012–2014) and for all treatments. The use of pinolene reduces sugars, with respect to both control and kaolin application; kaolin and control do not show a significant difference, with all data pooled together. When ANOVA was significant, different letters indicate differences between treatments with p-value ≤ 0.05 (Tukey all-pair comparisons) for the correspondent date. Shaded regions are mapped to the standard error of the mean, ● and solid line indicate the control, ▲ and short-dashed line indicate kaolin, ■ and dashed line indicate pinolene treatment.

Pinolene significantly reduced sugar amount with respect to kaolin application and control, both regarding data at harvest and throughout the ripening period. Fig 8 shows trends in sugar accumulation throughout ripening in all seasons and for all treatments. On average, during ripening, sugar accumulation in the pinolene treatment was reduced by 1.3°Bx (13 g L^-1^ in dissolved solids) with respect to the control, which means approx. 0.78% v/v in alcohol (considering an alcoholic production ratio of 0.06% v/v for 1 g L^-1^ of sugar), and 1.17°Bx (11.65 g L^-1^ in dissolved solids) with respect to kaolin, then 0.70% v/v in alcohol. Differences in sugar amount between kaolin and control were not significant. At harvest, difference between pinolene and control was even greater (2.09°Bx less in pinolene respect to the control, than 21 g L^-1^, or 1.06% alcohol v/v), while difference between kaolin treatment and control remained insignificant. Pinolene was already found useful in reducing sugar accumulation, and has therefore been proposed as a method to obtain low alcohol wines [17].

It is interesting to assess the effect of kaolin and pinolene on secondary metabolites, such as total flavonoids and anthocyanins, which are very important compounds for wine quality. Total flavonoids were slightly higher in the kaolin treatment than in control (+197 mg kg^-1^), but this difference was not significant, while decrease in total flavonoids in the pinolene treatment respect to both kaolin and control was significant. In addition, pinolene showed a very significant lower amount of anthocyanins than control (-154 mg kg^-1^ or 23% less) and even more than kaolin (-292 mg kg^-1^ or 36% less). Conversely, kaolin application significantly increased total anthocyanin amount with respect to control (137 mg kg^-1^, then 21%) with equivalent sugar amounts and yields between the two treatments.

The reduction in total flavonoids and anthocyanins observed in the pinolene treatment could be attributed to the reduction in An, whose effect on sugar accumulation was already observed (Fig 8). As already observed in previous studies [28,29], kaolin increased anthocyanin concentration, but such increase cannot be linked to an increase in An, which kaolin lowered. Reasons for such a positive increase in grape color can be probably found in kaolin application over berries, sprayed as part of the whole canopy at the time of treatment. Kaolin increases shade, which downregulates gene expression in the anthocyanin biosynthesis pathway [54,55], but it also lowers temperature. Authors in [34] found a decrease of approx. 5°C in kaolin-sprayed Sauvignon B. berry temperature. Temperature is the overriding variable in anthocyanin biosynthesis at photon fluxes higher than 100 μmol m^-2^ s^-1^ [56]. The optimum berry temperature for anthocyanins byosinthesis is around 30°C, while at temperatures higher than 35°C, anthocyanins stop accumulating [57] or could be degraded [58]. Therefore, the observed increase in anthocyanins in the kaolin treatment could be imputed to temperature regulation. Such a positive effect, which is even more important during drought when maturation is difficult, was already observed in grapes in [28,29]. An increase in color substances (lycopene) was also found on kaolin-sprayed tomatoes [23]. However, as already mentioned, it cannot be excluded that the observed reduction in An at the leaf level would be lower at the canopy level, where an actual increase could occur, as observed [26] on almond and walnut trees.

To our knowledge, any of the studies on particle film technology reported effects on wine sensory attributes, and therefore this question was also addressed in this study. The power of the test was low, because of number of judges, but able to significantly discriminate differences in the mean notes ≥ |1| [59]. In sensory analysis, wines from the pinolene treatment were less appreciated than wines from kaolin or control. While wines from control reached an average score of 5.5/9, wines from the kaolin treatment had a slightly higher value (scored 0.67 higher), which was not significant at a p-value < 0.05, but with a higher risk, p-value <0.1. Conversely, wines from pinolene were less appreciated (1.08 less), and in this case the difference was significant. Wines from pinolene also received a significantly lower score for attractiveness than control. Wines from kaolin were considered slightly more attractive, however, at p-value < 0.1, higher than the standard ranking. Wines from the pinolene treatment also had a significantly higher vegetal aroma than control (1.33 higher), while wines from kaolin were not considered significantly different from control. The last sensory descriptor evaluated was fruit character, which was significantly lower in pinolene than in kaolin or control, these last two groups with no difference between them.

## Conclusions

This 3-season study showed how the use of particle film technology can significantly improve grape composition during drought and wine appreciation. It also improved grapevine intrinsic water use efficiency in field conditions at the time of measurements. All this aspects together make kaolin a good supplemental tool to save water in vineyard, also considering that it is inexpensive and does not require special devices, but only commonly-used mounted sprayers. When measured, traditional film-forming antitranspirants such as pinolene (1-di-p menthene) limited An more than g_s,_ while application of these engineered clays allows an improvement of approx. 26% of WUEi in water-stressed vines. Because particle films act on WUEi by reducing g_s_, they are very effective during drought, when water-saving is even more important, while the effect is null in well-watered conditions. Particle film technology was therefore more effective in increasing WUEi during drought than common antitraspirants, and no negative effects were recorded on bunch number, berry weight, or sugar content by the use of kaolin, while anthocyanin content increased. Wines produced with use of kaolin were visually judged more attractive and slightly more appreciated than those obtained without kaolin application.

It will be necessary in the future to study how to optimize kaolin use to increase WUEi in the field, by considering time and frequency of applications, effect of hydrophobic or hydrophylic formulations, etc. It will also be interesting to integrate the practice with control of frequent problems such as pests, sunburns or UV damage, for which kaolin has been shown effective, and to evaluate the effects at canopy scale, and throughout the whole day as well. It will also interesting to compare kaolin to physiologically active antitranspirants such as the Chitosan.

In hot and dry climates, where water stress is a problem for viticulture, particle film technology appears a valid tool to increase sustainability in the vineyard, and limit irrigation use.

## Supporting Information

S1 Fig Application of pinolene and kaolin on fully-developed grapevine canopies. Antitranspirants **(a)** have been proposed as a tool to increase water use efficiency; in this study, they are compared to the recently-introduced particle film technology **(b)**. **a)** Effect on grapevine leaves of the film-forming antitranspirant pinolene **b)** Effect on grapevine leaves of engineered kaolin nanoparticles. Photos were taken just after product applications.
(JPG)

S2 Fig Meteorological trends in the studied vintages. Lines are mean temperature, bars are rainfall. Arrows and lines indicate application dates.
(TIFF)

S1 File Datasets and R code used to make figures and analysis (GZ)

S1 Table Basic chemical analysis on wines from all treatments and vintages (DOC)
