# Supplementary figures and images for: Comparing Kaolin and Pinolene to Improve Sustainable Grapevine Production during Drought
Source: PLoS One. 2016 Jun 13;11(6):e0156631. doi: 10.1371/journal.pone.0156631 (PMC4905681; doi:10.1371/journal.pone.0156631)

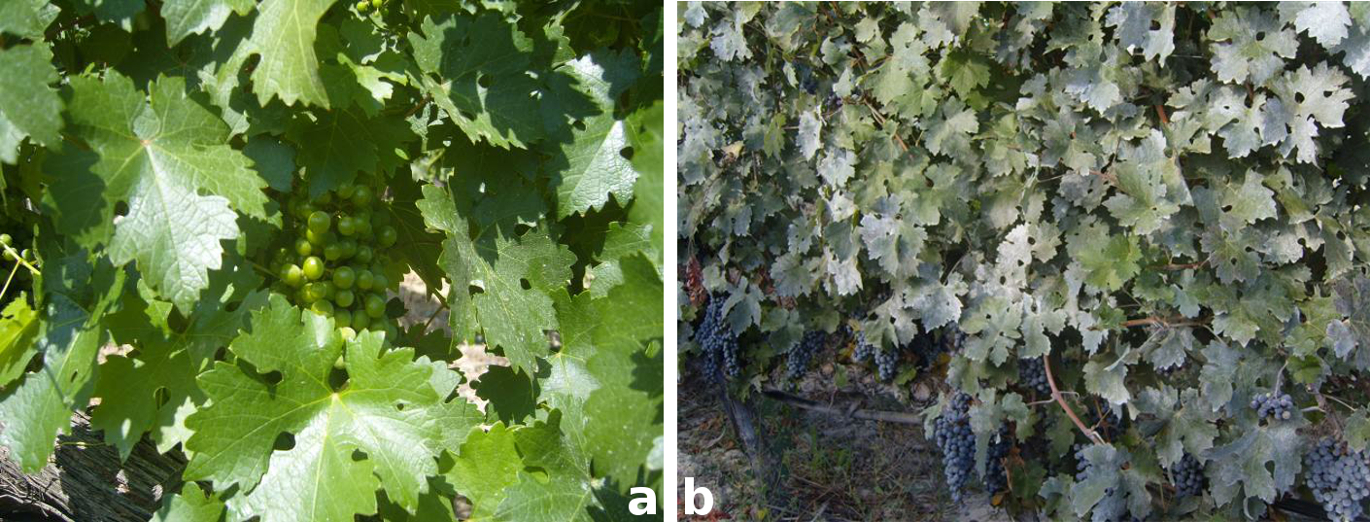

Supplement: S1 Fig — Antitranspirants (a) have been proposed as a tool to increase water use efficiency; in this study, they are compared to the recently-introduced particle film technology (b). a) Effect on grapevine leaves of the film-forming antitranspirant pinolene b) Effect on grapevine leaves of engineered kaolin nanoparticles. Photos were taken just after product applications. (JPG) [file pone.0156631.s001.jpg]
