# Supplementary material for: Comparing Kaolin and Pinolene to Improve Sustainable Grapevine Production during Drought
Source: PLoS One. 2016 Jun 13;11(6):e0156631. doi: 10.1371/journal.pone.0156631 (PMC4905681; doi:10.1371/journal.pone.0156631)
Supplement: S1 File — (GZ) [file pone.0156631.s003.gz › PONE_Brillante_et_al_2016_Code_and_Data/packrat/src/colorspace/colorspace_1.2-6.tar.gz!/colorspace/inst/doc/hcl-colors.pdf]

HCL-Based Color Palettes in R

Achim Zeileis
Universität Innsbruck

Kurt Hornik
WU Wirtschafts-
universität Wien

Paul Murrell
The University
of Auckland

Abstract

The package **colorspace** provides various functions providing perceptually-based color palettes for coding categorical data (qualitative palettes) and numerical variables (sequential and diverging palettes). We illustrate how these functions can be employed to generate various flavours of these palettes (with different emphases) and how they can be plugged into R graphics functions.

Keywords: HCL colors, qualitative palette, sequential palette, diverging palette.

1. Introduction

This is a companion vignette to Zeileis, Hornik, and Murrell (2009) providing further technical details on the construction of the palettes as well as R code illustrating the use of varying palettes in practice. The palettes as well as some graphical functions demonstrated are contained in the package **colorspace**.[1]

As a simple convenience function we will use the function

```
R> pal <- function(col, border = "light gray", ...)
+  {
+    n <- length(col)
+    plot(0, 0, type="n", xlim = c(0, 1), ylim = c(0, 1),
+      axes = FALSE, xlab = "", ylab = "", ...)
+    rect(0:(n-1)/n, 0, 1:n/n, 1, col = col, border = border)
+  }
```

which displays a set of colors using a sequence of shaded rectangles.

In the remainder of this vignette, we first outline how different types of palettes can be constructed and generated using the tools from **colorspace**. Subsequently, we present a collection of examples illustrating the tools in practice.

[1] In addition to the discussion here, a graphical user interface for choosing colors based on the ideas of Zeileis *et al.* (2009) is provided in the package as `choose_palette()`.

2. Color palettes

2.1. Qualitative palettes

Qualitative palettes are sets of colors for depicting different categories, i.e., for coding a categorical variable. To give the same perceptual weight to each category, chroma and luminance are kept constant and only the hue is varied for obtaining different colors (which are consequently all balanced towards the same gray).

In **colorspace**, qualitative palettes are implemented in the function

```
rainbow_hcl(n, c = 50, l = 70, start = 0, end = 360*(n-1)/n, ...)
```

where `n` controls the number of colors in the palette. The arguments `c` and `l` give the fixed chroma and luminance levels, respectively, and `start` and `end` specify the range of hue angles. The function is named after the base R function `rainbow()` which has a similar interface but chooses colors in HSV coordinates. Figure 1 depicts examples for generating qualitative sets of colors $(H, 50, 70)$ produced by

```
R> pal(rainbow_hcl(4, start = 30, end = 300), main = "dynamic")
R> pal(rainbow_hcl(4, start = 60, end = 240), main = "harmonic")
R> pal(rainbow_hcl(4, start = 270, end = 150), main = "cold")
R> pal(rainbow_hcl(4, start = 90, end = -30), main = "warm")
```

From left to right and top to down, this shows a palette from the full spectrum ($H = 30, 120, 210, 300$) creating a 'dynamic' set of colors, a 'harmonic' set with $H = 60, 120, 180, 240$, cold colors (from the blue/green part of the spectrum: $H = 270, 230, 190, 150$) and warm colors (from the yellow/red part of the spectrum: $H = 90, 50, 10, 330$), respectively.

2.2. Sequential palettes

Sequential palettes are used for coding numerical information that ranges in a certain interval where low values are considered to be uninteresting and high values are interesting. Suppose we need to visualize an intensity or interestingness i which (without loss of generality) is scaled to the unit interval.

Potentially, all three dimensions of HCL space can be used for coding the intensity, leading to colors from an interval of hues (i.e., differing types of colors), an interval of chroma values (i.e., differing colorfulness) and an interval of luminance (i.e., differing intensity of gray). If we allow chroma and luminance to increase non-linearly via a function of type i^p, the resulting formula is:

$$(H_2 - i \cdot (H_1 - H_2), C_{\max} - i^{p_1} \cdot (C_{\max} - C_{\min}), L_{\max} - i^{p_2} \cdot (L_{\max} - L_{\min})).$$

Two different "flavors" of this formula are readily implemented in **colorspace**, employing different defaults. For single hue palettes with $H_1 = H_2$ the function

```
sequential_hcl(n, h = 260, c = c(80, 0), l = c(30, 90), power = 1.5, ...)
```

is provided where the first element of `c` and `l` give the starting chroma and luminance coordinate (by default colorful and dark) and the second element the ending coordinate (by default gray and light). The `power` argument implements the parameter p from the i^p function (and can be a vector of length 2). Sequential palettes using an interval of hues are provided by

```
heat_hcl(n, h = c(0, 90), c = c(100, 30), l = c(50, 90), power = c(1/5, 1), ...)
```

named after the HSV-based R function `heat.colors()` and by default starts from a red and going to a yellow hue. The defaults in `heat_hcl()` are set differently compared to `sequential_hcl()` as to make the default HCL heat colors more similar to the HSV version. The defaults of `sequential_hcl()`, on the other hand, are set as to achieve a large contrast on the luminance axis. In addition `terrain_hcl()` is a wrapper for `heat_hcl()` producing colors similar to the HSV-based `terrain.colors()` from base R.

Various palettes produced from these functions are shown in Figure 2 using different pairs of hues as well as different chroma and luminance contrasts.

```
R> pal(sequential_hcl(12, c = 0, power = 2.2))
R> pal(sequential_hcl(12, power = 2.2))
R> pal(heat_hcl(12, c = c(80, 30), l = c(30, 90), power = c(1/5, 2)))
R> pal(terrain_hcl(12, c = c(65, 0), l = c(45, 90), power = c(1/2, 1.5)))
R> pal(rev(heat_hcl(12, h = c(0, -100), c = c(40, 80), l = c(75, 40),
+    power = 1)))
```

2.3. Diverging palettes

Diverging palettes are also used for coding numerical information ranging in a certain interval—however, this interval includes a neutral value. Analogously to the previous section, we suppose that we want to visualize an intensity or interestingness i from the interval $[-1, 1]$ (without loss of generality). Given useful sequential palettes, deriving diverging palettes is easy: two different hues are chosen for adding color to the same amount of 'gray' at a given intensity $|i|$.

Diverging palettes are implemented in the function

```
diverge_hcl(n, h = c(260, 0), c = 80, l = c(30, 90), power = 1.5, ...)
```

which has the same arguments as `sequential_hcl()` but takes a pair of hues `h`.

Figure 3 shows various examples of conceivable combinations of hue, chroma and luminance. The first palette uses a broader range on the luminance axis whereas the others mostly rely on chroma contrasts.

```
R> pal(diverge_hcl(7))
R> pal(diverge_hcl(7, c = 100, l = c(50, 90), power = 1))
R> pal(diverge_hcl(7, h = c(130, 43), c = 100, l = c(70, 90)))
R> pal(diverge_hcl(7, h = c(180, 330), c = 59, l = c(75, 95)))
```

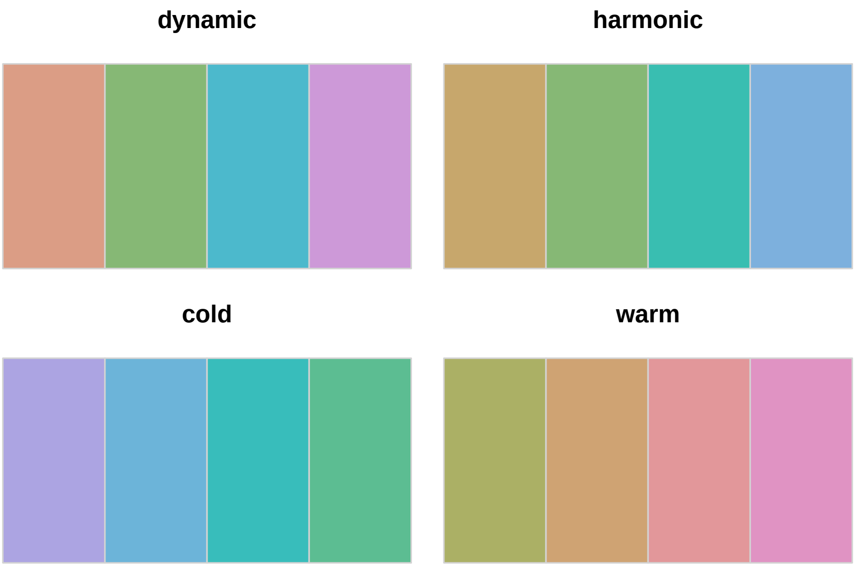

Figure 1: Examples for qualitative palettes. Hue is varied in different intervals for given $C = 50$ and $L = 70$.

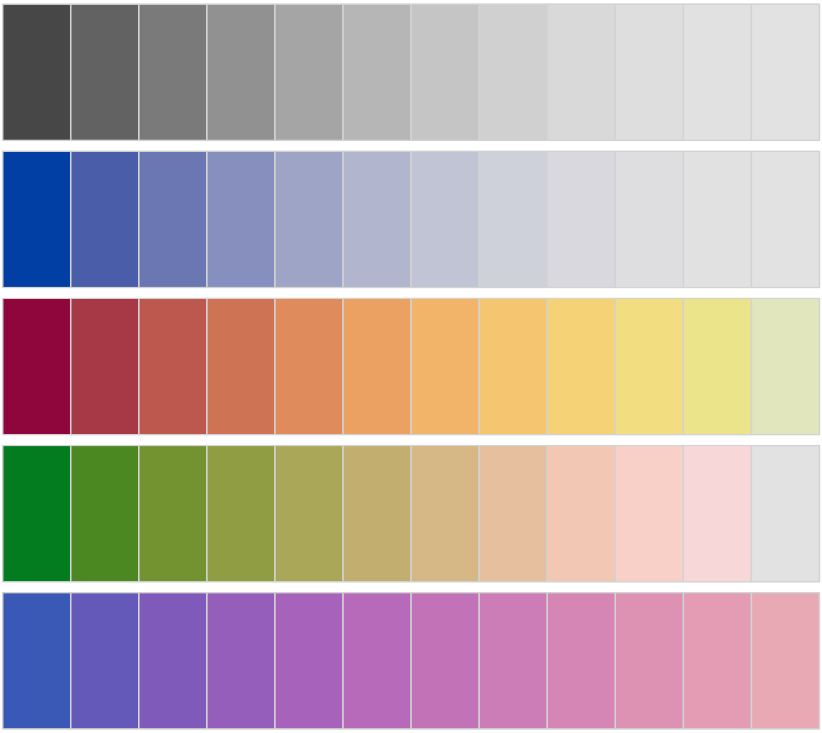

Figure 2: Examples for sequential palettes, varying only luminance (first panel), chroma and luminance (second panel), and hue, chroma and luminance (remaining panels).

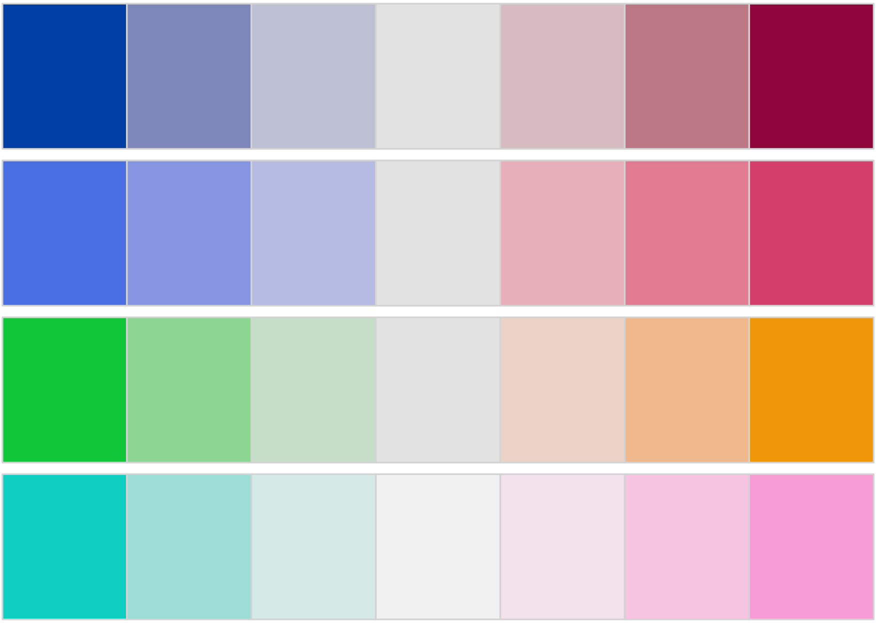

Figure 3: Examples for diverging palettes with different pairs of hues and decreasing luminance contrasts.

3. Illustrations

3.1. Qualitative palettes: Seats and votes in the German Bundestag

In this section, we show a collection of examples for the various types of palettes applied to statistical graphics. The first example illustrates qualitative palettes and visualizes data from the 2005 election for the German parliament "Bundestag". In this election, five parties were able to obtain enough votes to enter the Bundestag, the numbers of seats are given by

```
R> seats <- structure(c(226, 61, 54, 51, 222),
+    .Names = c("CDU/CSU", "FDP",  "Linke", "Gruene", "SPD"))
R> seats
```

We choose colors that are rough metaphors for the political parties, using a red hue $H = 0$ for the social democrats SPD, a blue hue $H = 240$ for the conservative CDU/CSU, a yellow hue $H = 60$ for the liberal FDP, a green hue $H = 120$ for the green party "Die Grünen" and a purple hue $H = 300$ for the leftist party "Die Linke". To obtain rather intense colors, we set chroma to $C = 60$ and luminance to $L = 75$:

```
R> parties <- rainbow_hcl(6, c = 60, l = 75)[c(5, 2, 6, 3, 1)]
R> names(parties) <- names(seats)
```

The distribution of seats is depicted in a pie chart in Figure 4. showing clearly that neither the governing coalition of SPD and Grüne nor the opposition of CDU/CSU and FDP could

assemble a majority. Given that no party would enter a coalition with the leftists, this lead to a big coalition of CDU/CSU and SPD.

```
R> pie(seats, clockwise = TRUE, col = parties, radius = 1)
```

To take a closer look at the regional distribution, we load the `Bundestag2005` data set containing a contingency table with the number of votes for each party stratified by province (Bundesland). Then, we re-order the provinces from north to south, first the 10 western provinces (the former Federal Republic of Germany, FRG), then the 6 eastern provinces (the former German Democratic Republic, GDR).

```
R> data("Bundestag2005", package = "vcd")
R> votes <- Bundestag2005[c(1, 3:5, 9, 11, 13:16, 2, 6:8, 10, 12),
+    c("CDU/CSU", "FDP", "SPD", "Gruene", "Linke")]
```

The data can then be visualized using a highlighted mosaic display via

```
R> mosaic(votes, gp = gpar(fill = parties[colnames(votes)]))
```

The annotation for this plot is clearly sub-optimal, hence we use the flexible `strucplot()` framework provided by **vcd** and display the data via

```
R> mosaic(votes, gp = gpar(fill = parties[colnames(votes)]),
+    spacing = spacing_highlighting, labeling = labeling_left,
+    labeling_args = list(rot_labels = c(0, 90, 0, 0),
+    varnames = FALSE, pos_labels = "center",
+    just_labels = c("center", "center", "center", "right")),
+    margins = unit(c(2.5, 1, 1, 12), "lines"),
+    keep_aspect_ratio = FALSE)
```

The output is shown in Figure 5 highlighting that the SPD performed better in the north and the CDU/CSU better in the south; furthermore, Die Linke performed particularly well in the eastern provinces and in Saarland.

3.2. Sequential palettes: Old Faithful geyser eruptions

To illustrate sequential palettes, a bivariate density estimation for the Old Faithful geyser eruptions data `geyser` from **MASS** is visualized. The Old Faithful geyser is one of the most popular sites in Yellowstone National Park and it is of some interest to understand the relation ship between the duration of a geyser eruption and the waiting time for this eruption. To look at the data, we use a bivariate kernel density estimate provided by the function `bkde2D()` from package **KernSmooth**.

```
R> library("KernSmooth")
R> data("geyser", package = "MASS")
R> dens <- bkde2D(geyser[,2:1], bandwidth = c(0.2, 3), gridsize = c(201, 201))
```

Subsequently, we look at the estimated kernel density by means of a heatmap (produced via `image()`) using two different sequential palettes: first with only gray colors

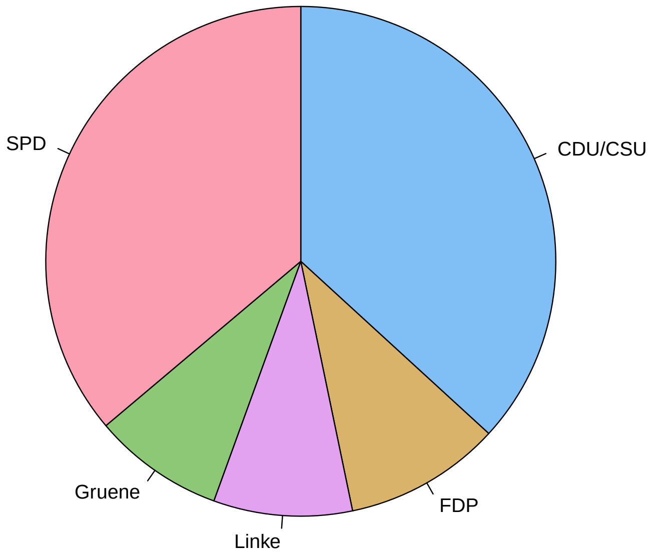

Figure 4: Seats in the German parliament.

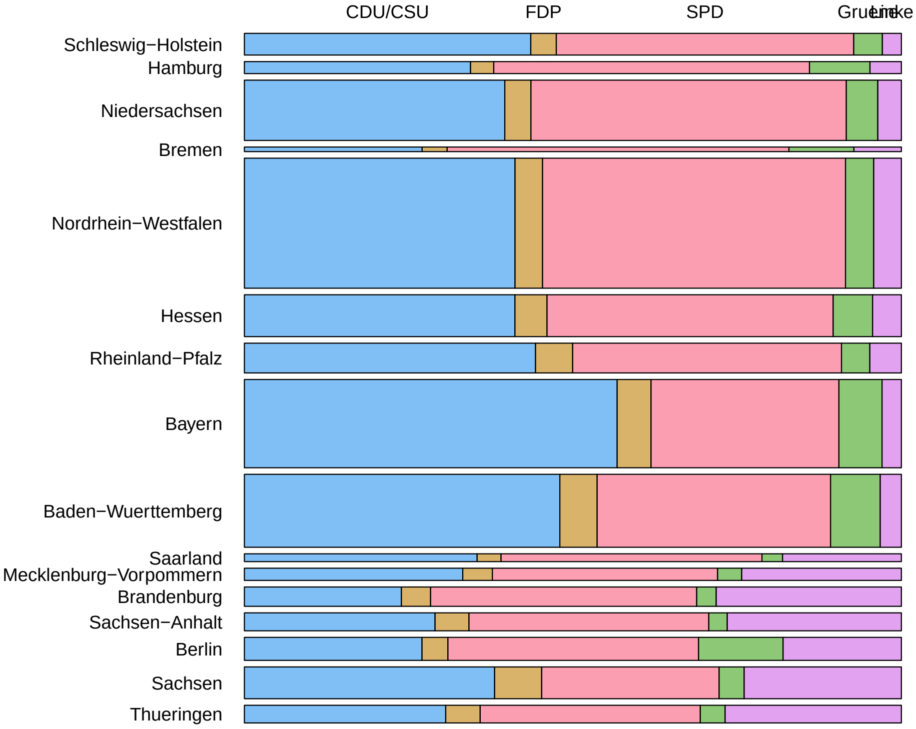

Figure 5: Votes in the German election 2005.

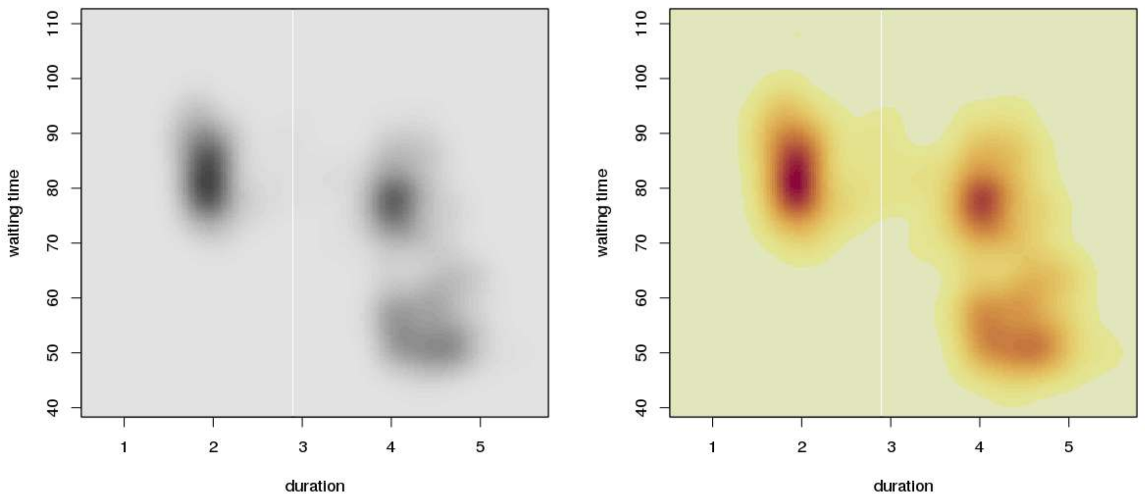

Figure 6: Bivariate density estimation for duration and waiting time for an eruption.

```
R> image(dens$x1, dens$x2, dens$fhat, xlab = "duration", ylab = "waiting time",
+    col = rev(heat_hcl(33, c = 0, l = c(30, 90), power = c(1/5, 1.3))))
```

and then using heat colors balanced towards the sam gray levels as above

```
R> image(dens$x1, dens$x2, dens$fhat, xlab = "duration", ylab = "waiting time",
+    col = rev(heat_hcl(33, c = c(80, 30), l = c(30, 90), power = c(1/5, 1.3))))
```

Figure 6 shows the resulting heatmaps revealing a multi-modal bivariate distribution: short waiting times (around 50 minutes) are typically followed by a long eruption (around 4 minutes) whereas long waiting times (around 80 minutes) can be followed by either a long or short eruption (around 4 minutes).

Another interesting question in this data set would be ask how long the waiting time for the next eruption is following a short or long eruption respectively. This can be visualized using another bivarate density estimate for the transformed data set matching the previous duration with the following waiting time:

```
R> library("KernSmooth")
R> geyser2 <- cbind(geyser$duration[-299], geyser$waiting[-1])
R> dens2 <- bkde2D(geyser2, bandwidth = c(0.2, 3), gridsize = c(201, 201))
```

Again, we look at this density using two heatmaps generated via

```
R> image(dens2$x1, dens2$x2, dens2$fhat, xlab = "duration", ylab = "waiting time",
+    col = rev(heat_hcl(33, c = 0, l = c(30, 90), power = c(1/5, 1.3))))
```

and

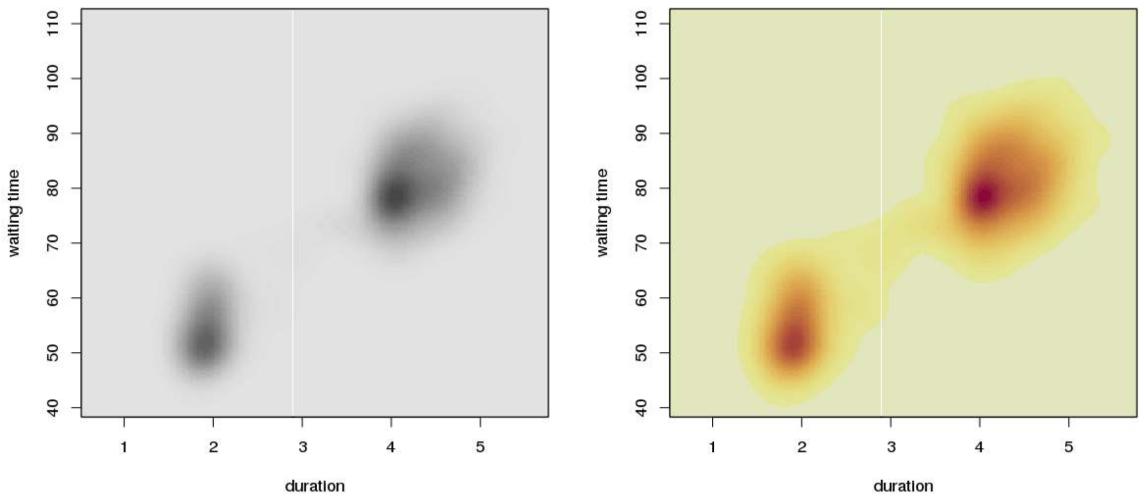

Figure 7: Bivariate density estimation for previous duration and following waiting time for an eruption.

```
R> image(dens2$x1, dens2$x2, dens2$fhat, xlab = "duration", ylab = "waiting time",
+    col = rev(heat_hcl(33, c = c(80, 30), l = c(30, 90), power = c(1/5, 1.3))))
```

Figure 7 shows the result that illustrates that long and short waiting times follow long and short eruption durations, resepectively.

3.3. Diverging palettes: Arthritis and SVM classification

Diverging palettes are particularly useful when visualizing residuals or correlations (with natural neutral value 0) or probabilities in 2-class supervised learning (with neutral value 0.5). Examples for both situations are provided here. First, we look at the outcome for the female patients from a double-blind clinical trial investigating a new treatment for rheumatoid arthritis.

```
R> art <- xtabs(~ Treatment + Improved, data = Arthritis,
+    subset = Sex == "Female")
```

For visualizing the data, we use a mosaic display with maximum shading (as derived by Zeileis, Meyer, and Hornik 2007) via

```
R> set.seed(1071)
R> mosaic(art, gp = shading_max, gp_args = list(n = 5000))
```

The mosaic rectangles in Figure 8 signal that the treatment lead to higher improvement compared to the placebo group; this effect is shown to be significant by the shading that codes the size of the Pearon residuals. Positive residuals, corresponding to more observations in the

corresponding cell than expected under independence, are depicted in blue, negative residuals in red. Light colors signal significance at 10% level, full colors significance at 1% level. The palette implicitly used in this plot is `diverge_hcl(5, c = c(100, 0), l = c(50, 90), power = 1)`, it can be modified using the arguments of `shading_max()` which has an interface similar to `diverge_hcl()`.

To illustrate the use of diverging palettes in 2-class classification, we generate some artificial date from a mixture of two bivariate normal distributions with different means and covariance matrices. The data are generated using **mvtnorm** and collected in a data frame `ex1`:

```
R> library("mvtnorm")
R> set.seed(123)
R> x1 <- rmvnorm(75, mean = c(1.5, 1.5),
+    sigma = matrix(c(1, 0.8, 0.8, 1), ncol = 2))
R> x2 <- rmvnorm(75, mean = c(-1, -1),
+    sigma = matrix(c(1, -0.3, -0.3, 1), ncol = 2))
R> X <- rbind(x1, x2)
R> ex1 <- data.frame(class = factor(c(rep("a", 75),
+    rep("b", 75))), x1 = X[,1], x2 = X[,2])
```

We fit a support vector machine (SVM) with a radial basis function kernel to this data set, using the function `ksvm()` from **kernlab**

```
R> library("kernlab")
R> fm <- ksvm(class ~ ., data = ex1, C = 0.5)
```

which can subsequently be easily visualized via

```
R> plot(fm, data = ex1)
```

The resulting plot in Figure 9 shows a heatmap with the fit of the SVM. The circles and triangles show the original observations, solid symbols correspond to the support vectors found. The shading underlying the plot visualizes the fitted decision values: values around 0 are on the decision boundary and are shaded in light gray, while regions that are firmly classified to one or the other class are shaded in full blue and red respectively. The palette used by the `plot()` method for `ksvm` objects cannot be easily modified—however, the colors employed are equivalent to `diverge_hcl(n, c = c(100, 0), l = c(50, 90), power = 1.3)`.

References

Zeileis A, Hornik K, Murrell P (2009). "Escaping RGBland: Selecting Colors for Statistical Graphics." *Computational Statistics & Data Analysis*, **53**, 3259–3270. doi:10.1016/j.csda.2008.11.033.

Zeileis A, Meyer D, Hornik K (2007). "Residual-Based Shadings for Visualizing (Conditional) Independence." *Journal of Computational and Graphical Statistics*, **16**(3), 507–525. doi:10.1198/106186007X237856.

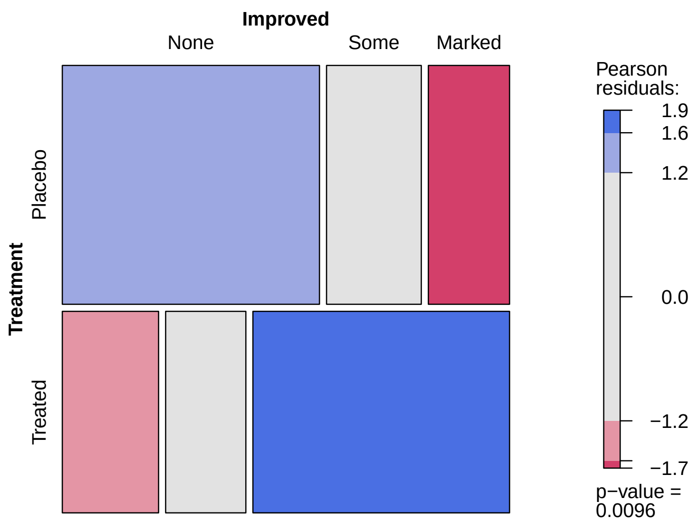

Figure 8: Extended mosaic display for arthritis data.

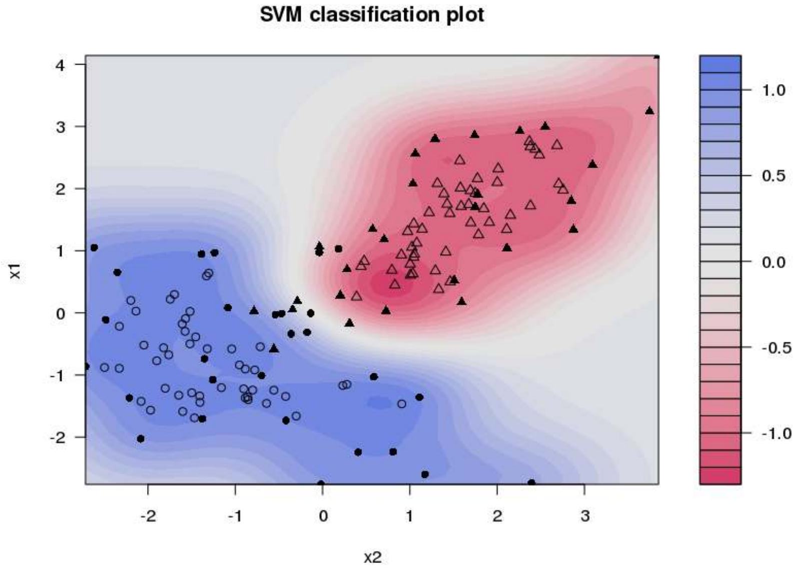

Figure 9: SVM classification plot.

Affiliation:

Achim Zeileis
Department of Statistics
Faculty of Economics and Statistics
Universität Innsbruck
Universitätsstraße 15
6020 Innsbruck, Austria
E-mail: Achim.Zeileis@R-project.org
URL: http://eeecon.uibk.ac.at/~zeileis/

Kurt Hornik
Institute for Statistics and Mathematics
Department of Finance, Accounting and Statistics
WU Wirtschaftsuniversität Wien
Augasse 2–6
1090 Wien, Austria
E-mail: Kurt.Hornik@R-project.org
URL: http://statmath.wu.ac.at/~hornik/

Paul Murrell
Department of Statistics
The University of Auckland
Private Bag 92019
Auckland, New Zealand
E-mail: paul@stat.auckland.ac.nz
URL: http://www.stat.auckland.ac.nz/~paul/
