# Supplementary material for: Comparing Kaolin and Pinolene to Improve Sustainable Grapevine Production during Drought
Source: PLoS One. 2016 Jun 13;11(6):e0156631. doi: 10.1371/journal.pone.0156631 (PMC4905681; doi:10.1371/journal.pone.0156631)
Supplement: S1 File — (GZ) [file pone.0156631.s003.gz › PONE_Brillante_et_al_2016_Code_and_Data/packrat/src/dplyr/dplyr_0.4.2.tar.gz!/dplyr/inst/doc/two-table.html]

Two-table verbs


# Two-table verbs

#### *2015-06-15*

It’s rare that a data analysis involves only a single table of data. In practice, you’ll normally have many tables that contribute to an analysis, and you need flexible tools to combine them. In dplyr, there are three families of verbs that work with two tables at a time:

- Mutating joins, which add new variables to one table from matching rows in another.
- Filtering joins, which filter observations from one table based on whether or not they match an observation in the other table.
- Set operations, which combine the observations in the data sets as if they were set elements.

(This discussion assumes that you have tidy data, where the rows are observations and the columns are variables. If you’re not familiar with that framework, I’d recommend reading up on it first.)

All two-table verbs work similarly. The first two arguments are `x` and `y`, and provide the tables to combine. The output is always a new table with the same type as `x`.

## Mutating joins

Mutating joins allow you to combine variables from multiple tables. For example, take the nycflights13 data. In one table we have flight information with an abbreviation for carrier, and in another we have a mapping between abbreviations and full names. You can use a join to add the carrier names to the flight data:

```
library("nycflights13")
# Drop unimportant variables so it's easier to understand the join results.
flights2 <- flights %>% select(year:day, hour, origin, dest, tailnum, carrier)

flights2 %>% 
  left_join(airlines)
#> Joining by: "carrier"
```

| year | month | day | hour | origin | dest | tailnum | carrier | name |
| --- | --- | --- | --- | --- | --- | --- | --- | --- |
| 2013 | 1 | 1 | 5 | EWR | IAH | N14228 | UA | United Air Lines Inc. |
| 2013 | 1 | 1 | 5 | LGA | IAH | N24211 | UA | United Air Lines Inc. |
| 2013 | 1 | 1 | 5 | JFK | MIA | N619AA | AA | American Airlines Inc. |
| 2013 | 1 | 1 | 5 | JFK | BQN | N804JB | B6 | JetBlue Airways |
| 2013 | 1 | 1 | 5 | LGA | ATL | N668DN | DL | Delta Air Lines Inc. |
| … | … | … | … | … | … | … | … | … |

### Controlling how the tables are matched

As well as `x` and `y`, each mutating join takes an argument `by` that controls which variables are used to match observations in the two tables. There are a few ways to specify it, as I illustrate below with various tables from nycflights13:

- `NULL`, the default. dplyr will will use all variables that appear in both tables, a **natural** join. For example, the flights and weather tables match on their common variables: year, month, day, hour and origin.

  ```
  flights2 %>% left_join(weather)
  #> Joining by: c("year", "month", "day", "hour", "origin")
  ```

  | year | month | day | hour | origin | dest | tailnum | carrier | temp | dewp | humid | wind\_dir |
  | --- | --- | --- | --- | --- | --- | --- | --- | --- | --- | --- | --- |
  | 2013 | 1 | 1 | 5 | EWR | IAH | N14228 | UA | NA | NA | NA | NA |
  | 2013 | 1 | 1 | 5 | LGA | IAH | N24211 | UA | NA | NA | NA | NA |
  | 2013 | 1 | 1 | 5 | JFK | MIA | N619AA | AA | NA | NA | NA | NA |
  | 2013 | 1 | 1 | 5 | JFK | BQN | N804JB | B6 | NA | NA | NA | NA |
  | 2013 | 1 | 1 | 5 | LGA | ATL | N668DN | DL | NA | NA | NA | NA |
  | … | … | … | … | … | … | … | … | … | … | … | … |

  (*Variables not shown*: wind\_speed (dbl), wind\_gust (dbl), precip (dbl), pressure (dbl), visib (dbl))
- A character vector, `by = "x"`. Like a natural join, but uses only some of the common variables. For example, `flights` and `planes` have `year` columns, but they mean different things so we only want to join by `tailnum`.

  ```
  flights2 %>% left_join(planes, by = "tailnum")
  ```

  | year.x | month | day | hour | origin | dest | tailnum | carrier | year.y |
  | --- | --- | --- | --- | --- | --- | --- | --- | --- |
  | 2013 | 1 | 1 | 5 | EWR | IAH | N14228 | UA | 1999 |
  | 2013 | 1 | 1 | 5 | LGA | IAH | N24211 | UA | 1998 |
  | 2013 | 1 | 1 | 5 | JFK | MIA | N619AA | AA | 1990 |
  | 2013 | 1 | 1 | 5 | JFK | BQN | N804JB | B6 | 2012 |
  | 2013 | 1 | 1 | 5 | LGA | ATL | N668DN | DL | 1991 |
  | … | … | … | … | … | … | … | … | … |

  (*Variables not shown*: type (chr), manufacturer (chr), model (chr), engines (int), seats (int), speed (int), engine (chr))

  Note that the year columns in the output are disambiguated with a suffix.
- A named character vector: `by = c("x" = "a")`. This will match variable `x` in table `x` to variable `a` in table `b`. The variables from use will be used in the output.

  Each flight has an origin and destination `airport`, so we need to specify which one we want to join to:

  ```
  flights2 %>% left_join(airports, c("dest" = "faa"))
  ```

  | year | month | day | hour | origin | dest | tailnum | carrier |
  | --- | --- | --- | --- | --- | --- | --- | --- |
  | 2013 | 1 | 1 | 5 | EWR | IAH | N14228 | UA |
  | 2013 | 1 | 1 | 5 | LGA | IAH | N24211 | UA |
  | 2013 | 1 | 1 | 5 | JFK | MIA | N619AA | AA |
  | 2013 | 1 | 1 | 5 | JFK | BQN | N804JB | B6 |
  | 2013 | 1 | 1 | 5 | LGA | ATL | N668DN | DL |
  | … | … | … | … | … | … | … | … |

  (*Variables not shown*: name (chr), lat (dbl), lon (dbl), alt (int), tz (dbl), dst (chr))

  ```
  flights2 %>% left_join(airports, c("origin" = "faa"))
  ```

  | year | month | day | hour | origin | dest | tailnum | carrier | name |
  | --- | --- | --- | --- | --- | --- | --- | --- | --- |
  | 2013 | 1 | 1 | 5 | EWR | IAH | N14228 | UA | Newark Liberty Intl |
  | 2013 | 1 | 1 | 5 | LGA | IAH | N24211 | UA | La Guardia |
  | 2013 | 1 | 1 | 5 | JFK | MIA | N619AA | AA | John F Kennedy Intl |
  | 2013 | 1 | 1 | 5 | JFK | BQN | N804JB | B6 | John F Kennedy Intl |
  | 2013 | 1 | 1 | 5 | LGA | ATL | N668DN | DL | La Guardia |
  | … | … | … | … | … | … | … | … | … |

  (*Variables not shown*: lat (dbl), lon (dbl), alt (int), tz (dbl), dst (chr))

### Types of join

There are four types of mutating join, which differ in their behaviour when a match is not found. We’ll illustrate each with a simple example:

```
(df1 <- data_frame(x = c(1, 2), y = 2:1))
```

| x | y |
| --- | --- |
| 1 | 2 |
| 2 | 1 |

```
(df2 <- data_frame(x = c(1, 3), a = 10, b = "a"))
```

| x | a | b |
| --- | --- | --- |
| 1 | 10 | a |
| 3 | 10 | a |

- `inner_join(x, y)` only includes observations that match in both `x` and `y`.

  ```
  df1 %>% inner_join(df2) %>% knitr::kable()
  #> Joining by: "x"
  ```

  | x | y | a | b |
  | --- | --- | --- | --- |
  | 1 | 2 | 10 | a |
- `left_join(x, y)` includes all observations in `x`, regardless of whether they match or not. This is the most commonly used join because it ensures that you don’t lose observations from your primary table.

  ```
  df1 %>% left_join(df2)
  #> Joining by: "x"
  ```

  | x | y | a | b |
  | --- | --- | --- | --- |
  | 1 | 2 | 10 | a |
  | 2 | 1 | NA | NA |
- `right_join(x, y)` includes all obserations in `y`. It’s equivalent to `left_join(y, x)`, but the columns will be ordered differently.

  ```
  df1 %>% right_join(df2)
  #> Joining by: "x"
  ```

  | x | y | a | b |
  | --- | --- | --- | --- |
  | 1 | 2 | 10 | a |
  | 3 | NA | 10 | a |

  ```
  df2 %>% left_join(df1)
  #> Joining by: "x"
  ```

  | x | a | b | y |
  | --- | --- | --- | --- |
  | 1 | 10 | a | 2 |
  | 3 | 10 | a | NA |
- `full_join()` includes all observations from `x` and `y`.

  ```
  df1 %>% full_join(df2)
  #> Joining by: "x"
  ```

  | x | y | a | b |
  | --- | --- | --- | --- |
  | 1 | 2 | 10 | a |
  | 2 | 1 | NA | NA |
  | 3 | NA | 10 | a |

The left, right and full joins are collectively know as **outer joins**. When a row doesn’t match in an outer join, the new variables are filled in with missing values.

### Observations

While mutating joins are primarily used to add new variables, they can also generate new observations. If a match is not unique, a join will add all possible combinations (the Cartesian product) of the matching observations:

```
df1 <- data_frame(x = c(1, 1, 2), y = 1:3)
df2 <- data_frame(x = c(1, 1, 2), z = c("a", "b", "a"))

df1 %>% left_join(df2)
#> Joining by: "x"
```

| x | y | z |
| --- | --- | --- |
| 1 | 1 | a |
| 1 | 1 | b |
| 1 | 2 | a |
| 1 | 2 | b |
| 2 | 3 | a |

## Filtering joins

Filtering joins match obserations in the same way as mutating joins, but affect the observations, not the variables. There are two types:

- `semi_join(x, y)` **keeps** all observations in `x` that have a match in `y`.
- `anti_join(x, y)` **drops** all observaitons in `x` that have a match in `y`.

These are most useful for diagnosing join mismatches. For example, there are many flights in the nycflights13 dataset that don’t have a matching tail number in the planes table:

```
library("nycflights13")
flights %>% 
  anti_join(planes, by = "tailnum") %>% 
  count(tailnum, sort = TRUE)
```

| tailnum | n |
| --- | --- |
|  | 2512 |
| N725MQ | 575 |
| N722MQ | 513 |
| N723MQ | 507 |
| N713MQ | 483 |
| … | … |

If you’re worried about what observations your joins will match, start with a `semi_join()` or `anti_join()`. `semi_join()` and `anti_join()` never duplicate; they only ever remove observations.

```
df1 <- data_frame(x = c(1, 1, 3, 4), y = 1:4)
df2 <- data_frame(x = c(1, 1, 2), z = c("a", "b", "a"))

# Four rows to start with:
df1 %>% nrow()
#> [1] 4
# And we get four rows after the join
df1 %>% inner_join(df2, by = "x") %>% nrow()
#> [1] 4
# But only two rows actually match
df1 %>% semi_join(df2, by = "x") %>% nrow()
#> [1] 2
```

## Set operations

The final type of two-table verb is set operations. These expect the `x` and `y` inputs to have the same variables, and treat the observations like sets:

- `intersect(x, y)`: return only observations in both `x` and `y`
- `union(x, y)`: return unique observations in `x` and `y`
- `setdiff(x, y)`: return observations in `x`, but not in `y`.

Given this simple data:

```
(df1 <- data_frame(x = 1:2, y = c(1L, 1L)))
```

| x | y |
| --- | --- |
| 1 | 1 |
| 2 | 1 |

```
(df2 <- data_frame(x = 1:2, y = 1:2))
```

| x | y |
| --- | --- |
| 1 | 1 |
| 2 | 2 |

The four possibilities are:

```
intersect(df1, df2)
```

| x | y |
| --- | --- |
| 1 | 1 |

```
# Note that we get 3 rows, not 4
union(df1, df2)
```

| x | y |
| --- | --- |
| 2 | 2 |
| 2 | 1 |
| 1 | 1 |

```
setdiff(df1, df2)
```

| x | y |
| --- | --- |
| 2 | 1 |

```
setdiff(df2, df1)
```

| x | y |
| --- | --- |
| 2 | 2 |

## Databases

Each two-table verb has a straightforward SQL equivalent:

| R | SQL |
| --- | --- |
| `inner_join()` | `SELECT * FROM x JOIN y ON x.a = y.a` |
| `left_join()` | `SELECT * FROM x LEFT JOIN y ON x.a = y.a` |
| `right_join()` | `SELECT * FROM x RIGHT JOIN y ON x.a = y.a` |
| `full_join()` | `SELECT * FROM x FULL JOIN y ON x.a = y.a` |
| `semi_join()` | `SELECT * FROM x WHERE EXISTS (SELECT 1 FROM y WHERE x.a = y.a)` |
| `anti_join()` | `SELECT * FROM x WHERE NOT EXISTS (SELECT 1 FROM y WHERE x.a = y.a)` |
| `intersect(x, y)` | `SELECT * FROM x INTERSECT SELECT * FROM y` |
| `union(x, y)` | `SELECT * FROM x UNION SELECT * FROM y` |
| `setdiff(x, y)` | `SELECT * FROM x EXCEPT SELECT * FROM y` |

`x` and `y` don’t have to be tables in the same database. If you specify `copy = TRUE`, dplyr will copy the `y` table into the same location as the `x` variable. This is useful if you’ve downloaded a summarised dataset and determined a subset of interest that you now want the full data for. You can use `semi_join(x, y, copy = TRUE)` to upload the indices of interest to a temporary table in the same database as `x`, and then perform a efficient semi join in the database.

If you’re working with large data, it maybe also be helpful to set `auto_index = TRUE`. That will automatically add an index on the join variables to the temporary table.

## Coercion rules

When joining tables, dplyr is a little more conservative than base R about the types of variable that it considers equivalent. This is mostly likely to surprise if you’re working factors:

- Factors with different levels are coerced to character with a warning:

  ```
  df1 <- data_frame(x = 1, y = factor("a"))
  df2 <- data_frame(x = 2, y = factor("b"))
  full_join(df1, df2) %>% str()
  #> Joining by: c("x", "y")
  #> Warning in outer_join_impl(x, y, by$x, by$y): joining factors with
  #> different levels, coercing to character vector
  #> Classes 'tbl_df', 'tbl' and 'data.frame':    2 obs. of  2 variables:
  #>  $ x: num  1 2
  #>  $ y: chr  "a" "b"
  ```
- Factors with the same levels in a different order are coerced to character with a warning:

  ```
  df1 <- data_frame(x = 1, y = factor("a", levels = c("a", "b")))
  df2 <- data_frame(x = 2, y = factor("b", levels = c("b", "a")))
  full_join(df1, df2) %>% str()
  #> Joining by: c("x", "y")
  #> Warning in outer_join_impl(x, y, by$x, by$y): joining factors with
  #> different levels, coercing to character vector
  #> Classes 'tbl_df', 'tbl' and 'data.frame':    2 obs. of  2 variables:
  #>  $ x: num  1 2
  #>  $ y: chr  "a" "b"
  ```
- Factors are preserved only if the levels match exactly:

  ```
  df1 <- data_frame(x = 1, y = factor("a", levels = c("a", "b")))
  df2 <- data_frame(x = 2, y = factor("b", levels = c("a", "b")))
  full_join(df1, df2) %>% str()
  #> Joining by: c("x", "y")
  #> Classes 'tbl_df', 'tbl' and 'data.frame':    2 obs. of  2 variables:
  #>  $ x: num  1 2
  #>  $ y: Factor w/ 2 levels "a","b": 1 2
  ```
- A factor and a character are coerced to character with a warning:

  ```
  df1 <- data_frame(x = 1, y = "a")
  df2 <- data_frame(x = 2, y = factor("a"))
  full_join(df1, df2) %>% str()
  #> Joining by: c("x", "y")
  #> Warning in outer_join_impl(x, y, by$x, by$y): joining factor and character
  #> vector, coercing into character vector
  #> Classes 'tbl_df', 'tbl' and 'data.frame':    2 obs. of  2 variables:
  #>  $ x: num  1 2
  #>  $ y: chr  "a" "a"
  ```

Otherwise logicals will be silently upcast to integer, and integer to numeric, but coercing to character will raise an error:

```
df1 <- data_frame(x = 1, y = 1L)
df2 <- data_frame(x = 2, y = 1.5)
full_join(df1, df2) %>% str()
#> Joining by: c("x", "y")
#> Classes 'tbl_df', 'tbl' and 'data.frame':    2 obs. of  2 variables:
#>  $ x: num  1 2
#>  $ y: num  1 1.5

df1 <- data_frame(x = 1, y = 1L)
df2 <- data_frame(x = 2, y = "a")
full_join(df1, df2) %>% str()
#> Joining by: c("x", "y")
#> Error in eval(expr, envir, enclos): cannot join on columns 'y' x 'y': Can't join on 'y' x 'y' because of incompatible types (character / integer)
```

## Multiple-table verbs

dplyr does not provide any functions for working with three or more tables. Instead use `Reduce()`, as described in Advanced R, to iteratively combine the two-table verbs to handle as many tables as you need.
