# Supplementary material for: Comparing Kaolin and Pinolene to Improve Sustainable Grapevine Production during Drought
Source: PLoS One. 2016 Jun 13;11(6):e0156631. doi: 10.1371/journal.pone.0156631 (PMC4905681; doi:10.1371/journal.pone.0156631)
Supplement: S1 File — (GZ) [file pone.0156631.s003.gz › PONE_Brillante_et_al_2016_Code_and_Data/packrat/src/dplyr/dplyr_0.4.2.tar.gz!/dplyr/inst/doc/hybrid-evaluation.html]

Hybrid evaluation


# Hybrid evaluation

#### *2015-06-15*

Consider this call to `summarise` :

```
summarise(per_day, flights = sum(flights))
```

One of the way `dplyr` achieves dramatic speedups is that expressions might not be evaluated by R, but by alternative code that is faster and uses less memory.

Conceptually the call to `summarise` above evaluates the expression `sum(flights)` on each subset of `flights` controlled by the grouping of `per_day`. This involves creating a new R vector to store the chunk and evaluate the R expression.

Evaluating the R expression might carry costs that can be avoided, i.e. S3 dispatch, …

`dplyr` recognizes the expression `sum(flights)` as the `sum` function applied to a known column of the data, making it possible to handle the dispatch early and once, avoid unneeded memory allocations and compute the result faster.

Hybrid evaluation is able to work on subexpressions. Consider:

```
foo <- function(x) x*x 
summarise(per_day, flights = foo(sum(flights)) )
```

`dplyr` will substitute the subexpressions it knows how to handle and leave the rest to standard R evaluation. Instead of evaluating `foo(sum(flights))`, R will only have to evaluate `foo(z)` where `z` is the result of the internal evaluation of `sum(flights)`.

# Implementation

Hybrid evaluation is designed to be extensible. Before we start registering custom hybrid evaluation handlers, we need to understand the system.

The first building block we need to cover is the `Result` class.

```
namespace dplyr {
    class Result {
    public:
        Result(){}
        virtual ~Result(){} ;
        
        virtual SEXP process( const GroupedDataFrame& gdf) = 0 ;
        
        virtual SEXP process( const FullDataFrame& df ) = 0 ;
        
        virtual SEXP process( const SlicingIndex& index ){
            return R_NilValue ;    
        }
        
    } ;
}
```

The two first methods deal with grouped and ungrouped data frames. We will mainly focus on the last method that operates on a `SlicingIndex`.

`SlicingIndex` is a class that encapsulates indices of a single chunk of a grouped data frame.

Hybrid evaluation really just is deriving from the `Result` class. Let’s consider a simpler version of `sum` that only handles numeric vectors. (The internal version is more complete, handles missing values, …).

```
class Sum : public Result {
  public:
    Sum( NumericVector data_ ): data(data_){}
    
    SEXP process( const SlicingIndex& index ){
      double res = 0.0 ;
      for( int i=0; i<index.size(); i++) res += data[ index[i] ] ;
      return NumericVector::create( res );
    }
    
    virtual SEXP process( const GroupedDataFrame& gdf){
      ...
    }
    virtual SEXP process( const FullDataFrame& df ){
      ...
    }
        
  private:
    NumericVector data ;
} ;
```

## Using Processor

Implementation of `Result` derived classes can be facilitated by the template class `Processor`. `Processor` is templated by two template parameters: - the R output type (`REALSXP`, `STRSXP`, …) - the class you are defining. (This uses the CRTP pattern).

When using `Processor` we only have to supply a `process_chunk` method that takes a `const SlicingIndex&` as input and returns an object suitable to go into a vector of the type controlled by the first template parameter.

The purpose of the `Processor` template is then to generate the boiler plate code for the three `process` methods defined by the `Result` interface.

A possible `Sum` implementation would then look something like this:

```
class Sum : public Processor<REALSXP, Sum> {
  public:
    Sum( NumericVector data_ ): data(data_){}
    
    double process_chunk( const SlicingIndex& index ){
      double res = 0.0 ;
      for( int i=0; i<index.size(); i++) res += data[ index[i] ] ;
      return res;
    }
    
  private:
    NumericVector data ;
}
```

Recognizing genericity here, we might want to make `Sum` a template class in order to handle more than just numeric vector :

```
template <int RTYPE>
class Sum : public Processor<REALSXP, Sum<RTYPE> > {
  public:
    typedef typename Rcpp::traits::storage_type<RTYPE>::type STORAGE ;
    
    Sum( Vector<RTYPE> data_ ): data(data_){}
    
    STORAGE process_chunk( const SlicingIndex& index ){
      STORAGE res = 0.0 ;
      for( int i=0; i<index.size(); i++) res += data[ index[i] ] ;
      return res;
    }
    
  private:
    Vector<RTYPE> data ;
}
```

Aside from not dealing with missing data and using internal knowledge of the `SlicingIndex` class, this implementation of `Sum` is close to the internal implementation in `dplyr`.

## Retrieving hybrid handlers

`dplyr` functions use the `get_handler` function to retrieve handlers for particular expressions.

```
Result* get_handler( SEXP call, const LazySubsets& subsets ){
    int depth = Rf_length(call) ;
    HybridHandlerMap& handlers = get_handlers() ;
    SEXP fun_symbol = CAR(call) ;
    if( TYPEOF(fun_symbol) != SYMSXP ) return 0 ;
    
    HybridHandlerMap::const_iterator it = handlers.find( fun_symbol ) ;
    if( it == handlers.end() ) return 0 ;
    
    return it->second( call, subsets, depth - 1 );
}
```

The `get_handler` performs a lookup in a hash table of type `HybridHandlerMap`.

```
typedef dplyr::Result* (*HybridHandler)(SEXP, const dplyr::LazySubsets&, int) ;
typedef dplyr_hash_map<SEXP,HybridHandler> HybridHandlerMap ;
```

`HybridHandlerMap` is simply a hash map where the map key is the symbol of the function and the map value type is a function pointer defined by `HybridHandler`.

The parameters of the `HybridHandler` function pointer type are: - The call we want to hybridify, e.g. something like `sum(flights)` - a `LazySubsets` reference. The only thing that is relevant about this class here is that it defines a `get_variable` method that takes a symbol `SEXP` and returns the corresponding variable from the data frame. - The number of arguments of the call. For example for `sum(flights)`, the number of arguments is `1`.

The purpose of the hybrid handler function is to return a `Result*` if it can handle the call or 0 if it cannot.

with our previous `Sum` template class, we could define a hybrid handler function like this:

```
Result* sum_handler(SEXP call, const LazySubsets& subsets, int nargs ){
  // we only know how to deal with argument
  if( nargs != 1 ) return 0 ;
  
  // get the first argument
  SEXP arg = CADR(call) ;
  
  // if this is a symbol, extract the variable from the subsets
  if( TYPEOF(arg) == SYMSXP ) arg = subsets.get_variable(arg) ;
  
  // we know how to handle integer vectors and numeric vectors
  switch( TYPEOF(arg) ){
  case INTSXP: return new Sum<INTSXP>(arg) ;
  case REALSXP: return new Sum<REALSXP>(arg) ;
  default: break ;
  }
  
  // we are here if we could not handle the call
  return 0 ;
}
```

## Registering hybrid handlers

`dplyr` enables users, most likely packages, to register their own hybrid handlers through the `registerHybridHandler`.

```
void registerHybridHandler( const char* , HybridHandler ) ;
```

To register the handler we created above, we then simply:

```
registerHybridHandler( "sum", sum_handler )  ;
```

## Putting it all together

We are going to register a handler called `hitchhiker` that always returns the answer to everything, i.e. `42`.

The code below is suitable to run through `sourceCpp`.

```
#include <dplyr.h>
// [[Rcpp::depends(dplyr,BH)]]

using namespace dplyr ;
using namespace Rcpp ;

// the class that derives from Result through Processor
class Hitchhiker : public Processor<INTSXP,Hitchhiker>{
public:
    
    // always returns 42, as it is the answer to everything
    int process_chunk( const SlicingIndex& ){
        return 42 ;    
    }
} ;

// we actually don't need the arguments
// we can just let this handler return a new Hitchhiker pointer
Result* hitchhiker_handler( SEXP, const LazySubsets&, int ){
    return new Hitchhiker ;        
}

// registration of the register, called from R, so exprted through Rcpp::export
// [[Rcpp::export]]
void registerHitchhiker(){
    registerHybridHandler( "hitchhiker", hitchhiker_handler );    
}

/*** R
    require(dplyr)
    registerHitchhiker()
    
    n  <- 10000
    df <- group_by( tbl_df( data.frame( 
        id = sample( letters[1:4], 1000, replace = TRUE ), 
        x  = rnorm(n)
        ) ), id )
    summarise( df, y = hitchhiker() )
    # Source: local data frame [4 x 2]
    # Groups:
    # 
    #   id  y
    # 1  a 42
    # 2  b 42
    # 3  c 42
    # 4  d 42
    
    summarise(df, y = mean(x) + hitchhiker())
    # Source: local data frame [4 x 2]
    # Groups:
    # 
    #   id        y
    # 1  a 42.00988
    # 2  b 42.00988
    # 3  c 42.01440
    # 4  d 41.99160
*/
```

## Registering hybrid handlers with a package

To register custom handlers in packages, the best place is the `init` entry point that R automatically calls when a package is loaded.

Instead of exposing the `registerHitchhiker` function as above, packages would typically register handlers like this:

```
#include <Rcpp.h>
#include <dplyr.h>

// R automatically calls this function when the maypack package is loaded. 
extern "C" void R_init_mypack( DllInfo* info ){
  registerHybridHandler( "hitchhiker", hitchhiker_handler );
}
```

For this your package must know about Rcpp and dplyr’s headers, which requires this information in the `DESCRIPTION` file:

```
LinkingTo: Rcpp, dplyr, BH
```

The `Makevars` and `Makevars.win` are similar to those used for any package that uses `Rcpp` features. See the `Rcpp` vignettes for details.
