# Supplementary material for: Comparing Kaolin and Pinolene to Improve Sustainable Grapevine Production during Drought
Source: PLoS One. 2016 Jun 13;11(6):e0156631. doi: 10.1371/journal.pone.0156631 (PMC4905681; doi:10.1371/journal.pone.0156631)
Supplement: S1 File — (GZ) [file pone.0156631.s003.gz › PONE_Brillante_et_al_2016_Code_and_Data/packrat/src/dplyr/dplyr_0.4.2.tar.gz!/dplyr/inst/doc/nse.html]

Non-standard evaluation


# Non-standard evaluation

#### *2015-06-15*

Dplyr uses non-standard evaluation (NSE) in all of the most important single table verbs: `filter()`, `mutate()`, `summarise()`, `arrange()`, `select()` and `group_by()`. NSE is important not only to save you typing, but for database backends, is what makes it possible to translate your R code to SQL. However, while NSE is great for interactive use it’s hard to program with. This vignette describes how you can opt out of NSE in dplyr, and instead rely only on SE (along with a little quoting).

Behind the scenes, NSE is powered by the lazyeval package. The goal is to provide an approach to NSE that you can learn once and then apply in many places (dplyr is the first of my packages to use this approach, but over time I will adopt it everywhere). You may want to read the lazyeval vignettes, if you like to learn more about the underlying details, or if you’d like to use this approach in your own packages.

## Standard evaluation basics

Every function in dplyr that uses NSE also has a version that uses SE. There’s a consistent naming scheme: the SE is the NSE name with `_` on the end. For example, the SE version of `summarise()` is `summarise_()`, the SE version of `arrange()` is `arrange_()`. These functions work very similarly to their NSE cousins, but the inputs must be “quoted”:

```
# NSE version:
summarise(mtcars, mean(mpg))
#>   mean(mpg)
#> 1  20.09062

# SE versions:
summarise_(mtcars, ~mean(mpg))
#>   mean(mpg)
#> 1  20.09062
summarise_(mtcars, quote(mean(mpg)))
#>   mean(mpg)
#> 1  20.09062
summarise_(mtcars, "mean(mpg)")
#>   mean(mpg)
#> 1  20.09062
```

There are three ways to quote inputs that dplyr understands:

- With a formula, `~ mean(mpg)`.
- With `quote()`, `quote(mean(mpg))`.
- As a string: `"mean(mpg)"`.

It’s best to use a formula, because a formula captures both the expression to evaluate, and the environment in which it should be a evaluated. This is important if the expression is a mixture of variables in the data frame and objects in the local environment:

```
constant1 <- function(n) ~n
summarise_(mtcars, constant1(4))
#>   n
#> 1 4
```

```
# Using anything other than a formula will fail because it doesn't
# know which environment to look in
constant2 <- function(n) quote(n)
summarise_(mtcars, constant2(4))
#> Error in eval(expr, envir, enclos): binding not found: 'n'
```

## Setting variable names

If you also want to output variables to vary, you need to pass a list of quoted objects to the `.dots` argument:

```
n <- 10
dots <- list(~mean(mpg), ~n)
summarise_(mtcars, .dots = dots)
#>   mean(mpg)  n
#> 1  20.09062 10

summarise_(mtcars, .dots = setNames(dots, c("mean", "count")))
#>       mean count
#> 1 20.09062    10
```

## Mixing constants and variables

What if you need to mingle constants and variables? Use the handy `lazyeval::interp()`:

```
library(lazyeval)
# Interp works with formulas, quoted calls and strings (but formulas are best)
interp(~ x + y, x = 10)
#> ~10 + y
interp(quote(x + y), x = 10)
#> 10 + y
interp("x + y", x = 10)
#> [1] "10 + y"

# Use as.name if you have a character string that gives a variable name
interp(~ mean(var), var = as.name("mpg"))
#> ~mean(mpg)
# or supply the quoted name directly
interp(~ mean(var), var = quote(mpg))
#> ~mean(mpg)
```

Because every action in R is a function call you can use this same idea to modify functions:

```
interp(~ f(a, b), f = quote(mean))
#> ~mean(a, b)
interp(~ f(a, b), f = as.name("+"))
#> ~a + b
interp(~ f(a, b), f = quote(`if`))
#> ~if (a) b
```

If you already have a list of values, use `.values`:

```
interp(~ x + y, .values = list(x = 10))
#> ~10 + y

# You can also interpolate variables defined in the current
# environment, but this is a little risky becuase it's easy
# for this to change without you realising
y <- 10
interp(~ x + y, .values = environment())
#> ~x + 10
```
