# Supplementary material for: Comparing Kaolin and Pinolene to Improve Sustainable Grapevine Production during Drought
Source: PLoS One. 2016 Jun 13;11(6):e0156631. doi: 10.1371/journal.pone.0156631 (PMC4905681; doi:10.1371/journal.pone.0156631)
Supplement: S1 File — (GZ) [file pone.0156631.s003.gz › PONE_Brillante_et_al_2016_Code_and_Data/packrat/src/dplyr/dplyr_0.4.2.tar.gz!/dplyr/inst/doc/new-sql-backend.html]

Adding a new SQL backend


# Adding a new SQL backend

#### *2015-06-15*

This document describes how to describe a new SQL backend to dplyr. It’s a work in progress, but will hopefully get started. If you’re familiar with how your database works, and at least one other database that dplyr already supports, this should be reasonably simple, and I’m happy to help if you get stuck. It’s also possible that a new database backend may need new methods - I’m also happy to add those as needed. If you need more help, please email the mailing list and I’ll help you get unstuck.

dplyr uses S3, so make sure that you’re familiar with the basics before you start.

See `?backend_sql` and `?backend_db` for a complete list of generics.

## Create the src object

Start by creating a new src function to represent the backend. Assuming we’re going to create a src for mssql, you’d call it `src_mssql()`, and you’d follow the pattern of an existing src. A simplified version of `src_postgres()` is show below:

```
src_postgres <- function(dbname = NULL, host = NULL, port = NULL, user = NULL,
                         password = NULL, ...) {

  con <- dbConnect(PostgreSQL(), host = host %||% "", dbname = dbname %||% "",
    user = user, password = password %||% "", port = port %||% "", ...)

  src_sql("postgres", con)
}
```

Use `src_sql()` to create a new S3 object with the correct structure. It must have a DBI connection, but it can store anything else that might be useful.

Next, implement a method for `src_desc()` that briefly describes the source:

```
#' @export
src_desc.src_postgres <- function(x) {
  info <- dbGetInfo(con)
  host <- if (info$host == "") "localhost" else info$host

  paste0("postgres ", info$serverVersion, " [", info$user, "@",
    host, ":", info$port, "/", info$dbname, "]")
}
```

If you read the source code for the real `src_postgres()` you’ll notice that it caches the `getGetInfo()` field on creation, since this saves a little time when printing tbls.

Before continuing, check that you can create a connection to a local database. Don’t worry if the object doesn’t print correctly, you’ll need to add a few other methods first.

## Table listing

Next implement two methods that allow dplyr to query the tables present in a database:

- `db_list_tables()`
- `db_has_table()`

These should be very straightforward if there’s an existing DBI interface, and only a little harder if not. This methods basically provide a shim around individual database packages to make them behave the same. See the documentation for more details.

Once you have implemented these methods you should be able to connect to a local database get a useful description, including a list of tables. Don’t continue on until you have that working.

## tbl

Next implement the `tbl()` method for your data source. This will probably just be

```
tbl.src_mssql <- function(src, from, ...) {
  tbl_sql("mssql", src = src, from = from, ...)
}
```

Before continuing, make sure you can connect to an existing table, and that the results are printed reasonably. If not, that may indicate your database uses a non-standard DBI interface. That means you’ll probably you’ll probably also need to subclass the R6 Query class, and the `S3` query method that creates a new query object. If that happens to you, I’d strongly recommend asking for help.

This is also a good time implement `explain()`, by adding a method for `db_explain()`.

If your database uses non-standard quoting (i.e. something other than `"` for identifiers and `'` for strings), implement methods for `sql_escape_string()` and `sql_escape_ident()`.

At this point, all the basic verbs (`summarise()`, `filter()`, `arrange()`, `mutate()` etc) should also work, but it’s hard to test without some data.

## `copy_to()`

Next, implement the methods that power `copy_to()` work. Once you’ve implemented these methods, you’ll be able copy datasets from R into your database, which will make testing much easier.

- `db_data_type()`
- `sql_begin()`, `sql_commit()`, `sql_rollback()`
- `sql_create_table()`, `sql_insert_into()`, `sql_drop_table()`
- `sql_create_index()`, `sql_analyze()`

If the database doesn’t support a function, just return `TRUE` without doing anything. If you find these methods a very poor match to your backend, you may find it easier to provide a direct `copy_to()` method.

At this point, you should be able to copy the nycflights13 data packages into your database with (e.g.):

```
copy_nycflights13(src_mssql(...))
copy_lahman(src_mssql(...))
```

Don’t proceed further until this works, and you’ve verified that the basic single table verbs word.

## Query metadata

If you database provides a nice way to access query metadata, implement `db_query_fields()` and `db_query_rows()` which return field names and row count for a given query.

## Compute, collect and collapse

Next, check that `collapse()`, `compute()`, and `collect()` work.

- If `collapse()` fails, your database has a non-standard way of constructing subqueries. Add a method for `sql_subquery()`.
- If `compute()` fails, your database has a non-standard way of saving queries in temporary tables. Add a method for `db_save_query()`.

## Multi table verbs

Next check the multitable verbs:

- `left_join()`, `inner_join()`: powered by `sql_join()`
- `semi_join()`, `anti_join()`: powered by `sql_semi_join()`
- `union()`, `intersect()`, `setdiff()`: powered by `sql_set_op()`

## sql translation

To finish off, you can add custom R -> SQL translation by providing a method for `src_translate_env()`. This function should return an object created by `sql_variant()`. See existing methods for examples.
