# Supplementary material for: Comparing Kaolin and Pinolene to Improve Sustainable Grapevine Production during Drought
Source: PLoS One. 2016 Jun 13;11(6):e0156631. doi: 10.1371/journal.pone.0156631 (PMC4905681; doi:10.1371/journal.pone.0156631)
Supplement: S1 File — (GZ) [file pone.0156631.s003.gz › PONE_Brillante_et_al_2016_Code_and_Data/packrat/src/dplyr/dplyr_0.4.2.tar.gz!/dplyr/inst/doc/window-functions.html]

Window functions and grouped mutate/filter


# Window functions and grouped mutate/filter

#### *2015-06-15*

A **window function** is a variation on an aggregation function. Where an aggregation function, like `sum()` and `mean()`, takes n inputs and return a single value, a window function returns n values. The output of a window function depends on all its input values, so window functions don’t include functions that work element-wise, like `+` or `round()`. Window functions include variations on aggregate functions, like `cumsum()` and `cummean()`, functions for ranking and ordering, like `rank()`, and functions for taking offsets, like `lead()` and `lag()`.

Window functions are used in conjunction with `mutate` and `filter` to solve a wide range of problems, some of which are shown below:

```
library(Lahman)
batting <- select(tbl_df(Batting), playerID, yearID, teamID, G, AB:H) 
batting <- arrange(batting, playerID, yearID, teamID)
players <- group_by(batting, playerID)

# For each player, find the two years with most hits
filter(players, min_rank(desc(H)) <= 2 & H > 0)
# Within each player, rank each year by the number of games played
mutate(players, G_rank = min_rank(G))

# For each player, find every year that was better than the previous year
filter(players, G > lag(G))
# For each player, compute avg change in games played per year
mutate(players, G_change = (G - lag(G)) / (yearID - lag(yearID)))

# For each player, find all where they played more games than average
filter(players, G > mean(G))
# For each, player compute a z score based on number of games played
mutate(players, G_z = (G - mean(G)) / sd(G))
```

This vignette is broken down into two sections. First you’ll learn about the five families of window functions in R, and what you can use them for. If you’re only working with local data sources, you can stop there. Otherwise, continue on to learn about window functions in SQL. They are relatively new, but are supported by Postgres, Amazon’s Redshift and Google’s bigquery. The window functions themselves are basically the same (modulo a few name conflicts), but their specification is a little different. I’ll briefy review how they work, and then show how dplyr translates their R equivalents to SQL.

Before reading this vignette, you should be familiar with `mutate()` and `filter()`. If you want to use window functions with SQL databases, you should also be familiar with the basics of dplyr’s SQL translation.

## Types of window functions

There are five main families of window functions. Two families are unrelated to aggregation functions:

- Ranking and ordering functions: `row_number()`, `min_rank` (`RANK` in SQL), `dense_rank()`, `cume_dist()`, `percent_rank()`, and `ntile()`. These functions all take a vector to order by, and return various types of ranks.
- Offsets `lead()` and `lag()` allow you to access the previous and next values in a vector, making it easy to compute differences and trends.

The other three families are variations on familiar aggregate functions:

- Cumulative aggregates: `cumsum()`, `cummin()`, `cummax()` (from base R), and `cumall()`, `cumany()`, and `cummean()` (from dplyr).
- Rolling aggregates operate in a fixed width window. You won’t find them in base R or in dplyr, but there are many implementations in other packages, such as RcppRoll.
- Recycled aggregates, where an aggregate is repeated to match the length of the input. These are not needed in R because vector recycling automatically recycles aggregates where needed. They are important in SQL, because the presence of an aggregation function usually tells the database to return only one row per group.

Each family is described in more detail below, focussing on the general goals and how to use them with dplyr. For more details, refer to the individual function documentation.

### Ranking functions

The ranking functions are variations on a theme, differing in how they handle ties:

```
x <- c(1, 1, 2, 2, 2)

row_number(x)
#> [1] 1 2 3 4 5
min_rank(x)
#> [1] 1 1 3 3 3
dense_rank(x)
#> [1] 1 1 2 2 2
```

If you’re familiar with R, you may recognise that `row_number()` and `min_rank()` can be computed with the base `rank()` function and various values of the `ties.method` argument. These functions are provided to save a little typing, and to make it easier to convert between R and SQL.

Two other ranking functions return numbers between 0 and 1. `percent_rank()` gives the percentage of the rank; `cume_dist()` gives the proportion of values less than or equal to the current value.

```
cume_dist(x)
#> [1] 0.4 0.4 1.0 1.0 1.0
percent_rank(x)
#> [1] 0.0 0.0 0.5 0.5 0.5
```

These are useful if you want to select (for example) the top 10% of records within each group. For example:

```
# Selects best two years
filter(players, min_rank(desc(G)) < 2)

# Selects best 10% of years
filter(players, cume_dist(desc(G)) < 0.1)
```

Finally, `ntile()` divides the data up into `n` evenly sized buckets. It’s a coarse ranking, and it can be used in with `mutate()` to divide the data into buckets for further summary. For example, we could use `ntile()` to divide the players within a team into four ranked groups, and calculate the average number of games within each group.

```
by_team_player <- group_by(batting, teamID, playerID)
by_team <- summarise(by_team_player, G = sum(G))
by_team_quartile <- group_by(by_team, quartile = ntile(G, 4))
summarise(by_team_quartile, mean(G))
```

| quartile | mean(G) |
| --- | --- |
| 1 | 5.355776 |
| 2 | 24.912267 |
| 3 | 77.288933 |
| 4 | 373.693195 |

All ranking functions rank from lowest to highest so that small input values get small ranks. Use `desc()` to rank from highest to lowest.

### Lead and lag

`lead()` and `lag()` produce offset versions of a input vector that is either ahead of or behind the original vector.

```
x <- 1:5
lead(x)
#> [1]  2  3  4  5 NA
lag(x)
#> [1] NA  1  2  3  4
```

You can use them to:

- Compute differences or percent changes.

  ```
  # Compute the relative change in games played
  mutate(players, G_delta = G - lag(G))
  ```

  Using `lag()` is more convenient than `diff()` because for `n` inputs `diff()` returns `n - 1` outputs.
- Find out when a value changes.

  ```
  # Find when a player changed teams
  filter(players, teamID != lag(teamID))
  ```

`lead()` and `lag()` have an optional argument `order_by`. If set, instead of using the row order to determine which value comes before another, they will use another variable. This important if you have not already sorted the data, or you want to sort one way and lag another.

Here’s a simple example of what happens if you don’t specify `order_by` when you need it:

```
df <- data.frame(year = 2000:2005, value = (0:5) ^ 2)
scrambled <- df[sample(nrow(df)), ]

wrong <- mutate(scrambled, running = cumsum(value))
arrange(wrong, year)
#>   year value running
#> 1 2000     0      14
#> 2 2001     1       1
#> 3 2002     4       5
#> 4 2003     9      14
#> 5 2004    16      55
#> 6 2005    25      39

right <- mutate(scrambled, running = order_by(year, cumsum(value)))
arrange(right, year)
#>   year value running
#> 1 2000     0       0
#> 2 2001     1       1
#> 3 2002     4       5
#> 4 2003     9      14
#> 5 2004    16      30
#> 6 2005    25      55
```

### Cumulative aggregates

Base R provides cumulative sum (`cumsum()`), cumulative min (`cummin()`) and cumulative max (`cummax()`). (It also provides `cumprod()` but that is rarely useful). Other common accumulating functions are `cumany()` and `cumall()`, cumulative versions of `||` and `&&`, and `cummean()`, a cumulative mean. These are not included in base R, but efficient versions are provided by `dplyr`.

`cumany()` and `cumall()` are useful for selecting all rows up to, or all rows after, a condition is true for the first (or last) time. For example, we can use `cumany()` to find all records for a player after they played a year with 150 games:

```
filter(players, cumany(G > 150))
```

Like lead and lag, you may want to control the order in which the accumulation occurs. None of the built in functions have an `order_by` argument so `dplyr` provides a helper: `order_by()`. You give it the variable you want to order by, and then the call to the window function:

```
x <- 1:10
y <- 10:1
order_by(y, cumsum(x))
#>  [1] 55 54 52 49 45 40 34 27 19 10
```

This function uses a bit of non-standard evaluation, so I wouldn’t recommend using it inside another function; use the simpler but less concise `with_order()` instead.

### Recycled aggregates

R’s vector recycling make it easy to select values that are higher or lower than a summary. I call this a recycled aggregate because the value of the aggregate is recycled to be the same length as the original vector. Recycled aggregates are useful if you want to find all records greater than the mean or less than the median:

```
filter(players, G > mean(G))
filter(players, G < median(G))
```

While most SQL databases don’t have an equivalent of `median()` or `quantile()`, when filtering you can achieve the same effect with `ntile()`. For example, `x > median(x)` is equivalent to `ntile(x, 2) == 2`; `x > quantile(x, 75)` is equivalent to `ntile(x, 100) > 75` or `ntile(x, 4) > 3`.

```
filter(players, ntile(G, 2) == 2)
```

You can also use this idea to select the records with the highest (`x == max(x)`) or lowest value (`x == min(x)`) for a field, but the ranking functions give you more control over ties, and allow you to select any number of records.

Recycled aggregates are also useful in conjunction with `mutate()`. For example, with the batting data, we could compute the “career year”, the number of years a player has played since they entered the league:

```
mutate(players, career_year = yearID - min(yearID) + 1)
```

| playerID | yearID | teamID | G | AB | R | H | career\_year |
| --- | --- | --- | --- | --- | --- | --- | --- |
| aardsda01 | 2004 | SFN | 11 | 0 | 0 | 0 | 1 |
| aardsda01 | 2006 | CHN | 45 | 2 | 0 | 0 | 3 |
| aardsda01 | 2007 | CHA | 25 | 0 | 0 | 0 | 4 |
| aardsda01 | 2008 | BOS | 47 | 1 | 0 | 0 | 5 |
| … | … | … | .. | .. | . | . | … |

Or, as in the introductory example, we could compute a z-score:

```
mutate(players, G_z = (G - mean(G)) / sd(G))
```

| playerID | yearID | teamID | G | AB | R | H | G\_z |
| --- | --- | --- | --- | --- | --- | --- | --- |
| aardsda01 | 2004 | SFN | 11 | 0 | 0 | 0 | -1.1167685 |
| aardsda01 | 2006 | CHN | 45 | 2 | 0 | 0 | 0.3297126 |
| aardsda01 | 2007 | CHA | 25 | 0 | 0 | 0 | -0.5211586 |
| aardsda01 | 2008 | BOS | 47 | 1 | 0 | 0 | 0.4147997 |
| … | … | … | .. | .. | . | . | … |

## Window functions in SQL

Window functions have a slightly different flavour in SQL. The syntax is a little different, and the cumulative, rolling and recycled aggregate functions are all based on the simple aggregate function. The goal in this section is not to tell you everything you need to know about window functions in SQL, but to remind you of the basics and show you how dplyr translates your R expressions in to SQL.

### Structure of a window function in SQL

In SQL, window functions have the form `[expression] OVER ([partition clause] [order clause] [frame_clause])`:

- The **expression** is a combination of variable names and window functions. Support for window functions varies from database to database, but most support the ranking functions, `lead`, `lag`, `nth`, `first`, `last`, `count`, `min`, `max`, `sum`, `avg` and `stddev`. dplyr generates this from the R expression in your `mutate` or `filter` call.
- The **partition clause** specifies how the window function is broken down over groups. It plays an analogous role to `GROUP BY` for aggregate functions, and `group_by()` in dplyr. It is possible for different window functions to be partitioned into different groups, but not all databases support it, and neither does dplyr.
- The **order clause** controls the ordering (when it makes a difference). This is important for the ranking functions since it specifies which variables to rank by, but it’s also needed for cumulative functions and lead. Whenever you’re thinking about before and after in SQL, you must always tell it which variable defines the order. In dplyr you do this with `arrange()`. If the order clause is missing when needed, some databases fail with an error message while others return non-deterministic results.
- The **frame clause** defines which rows, or **frame**, that are passed to the window function, describing which rows (relative to the current row) should be included. The frame clause provides two offsets which determine the start and end of frame. There are three special values: -Inf means to include all preceeding rows (in SQL, “unbounded preceding”), 0 means the current row (“current row”), and Inf means all following rows (“unbounded following)”. The complete set of options is comprehensive, but fairly confusing, and is summarised visually below.

  Of the many possible specifications, there are only three that commonly used. They select between aggregation variants:

  - Recycled: `BETWEEN UNBOUND PRECEEDING AND UNBOUND FOLLOWING`
  - Cumulative: `BETWEEN UNBOUND PRECEEDING AND CURRENT ROW`
  - Rolling: `BETWEEN 2 PRECEEDING AND 2 FOLLOWING`

  dplyr generates the frame clause based on whether your using a recycled aggregate or a cumulative aggregate.

It’s easiest to understand these specifications by looking at a few examples. Simple examples just need the partition and order clauses:

- Rank each year within a player by number of home runs: `RANK() OVER (PARTITION BY playerID ORDER BY desc(H))`
- Compute change in number of games from one year to the next: `G - LAG(G) OVER (PARTITION G playerID ORDER BY yearID)`

Aggregate variants are more verbose because we also need to supply the frame clause:

- Running sum of G for each player: `SUM(G) OVER (PARTITION BY playerID ORDER BY yearID BETWEEN UNBOUND PRECEEDING AND CURRENT ROW)`
- Compute the career year: `YearID - min(YearID) OVER (PARTITION BY playerID BETWEEN UNBOUND PRECEEDING AND UNBOUND FOLLOWING) + 1`
- Compute a rolling average of games player: `MEAN(G) OVER (PARTITION BY playerID ORDER BY yearID BETWEEN 2 PRECEEDING AND 2 FOLLOWING)`

You’ll notice that window functions in SQL are more verbose than in R. This is because different window functions can have different partitions, and the frame specification is more general than the two aggregate variants (recycled and cumulative) provided by dplyr. dplyr makes a tradeoff: you can’t access rarely used window function capabilities (unless you write raw SQL), but in return, common operations are much more succinct.

### Translating dplyr to SQL

To see how individual window functions are translated to SQL, we can use `translate_sql()` with the argument `window = TRUE`.

```
if (has_lahman("postgres")) {
  players_db <- group_by(tbl(lahman_postgres(), "Batting"), playerID)
  
  print(translate_sql(mean(G), tbl = players_db, window = TRUE))
  print(translate_sql(cummean(G), tbl = players_db, window = TRUE))
  print(translate_sql(rank(G), tbl = players_db, window = TRUE))
  print(translate_sql(ntile(G, 2), tbl = players_db, window = TRUE))
  print(translate_sql(lag(G), tbl = players_db, window = TRUE))
}
```

If the tbl has been arranged previously, then that ordering will be used for the order clause:

```
if (has_lahman("postgres")) {
  players_by_year <- arrange(players_db, yearID)
  print(translate_sql(cummean(G), tbl = players_by_year, window = TRUE))
  print(translate_sql(rank(), tbl = players_by_year, window = TRUE))
  print(translate_sql(lag(G), tbl = players_by_year, window = TRUE))
}
```

There are some challenges when translating window functions between R and SQL, because dplyr tries to keep the window functions as similar as possible to both the existing R analogues and to the SQL functions. This means that there are three ways to control the order clause depending on which window function you’re using:

- For ranking functions, the ordering variable is the first argument: `rank(x)`, `ntile(y, 2)`. If omitted or `NULL`, will use the default ordering associated with the tbl (as set by `arrange()`).
- Accumulating aggegates only take a single argument (the vector to aggregate). To control ordering, use `order_by()`.
- Aggregates implemented in dplyr (`lead`, `lag`, `nth_value`, `first_value`, `last_value`) have an `order_by` argument. Supply it to override the default ordering.

The three options are illustrated in the snippet below:

```
mutate(players,
  min_rank(yearID),
  order_by(yearID, cumsum(G)),
  lead(order_by = yearID, G)
)
```

Currently there is no way to order by multiple variables, except by setting the default ordering with `arrange()`. This will be added in a future release.

### Translating filters based on window functions

There are some restrictions on window functions in SQL that make their use with `WHERE` somewhat challenging. Take this simple example, where we want to find the year each player played the most games:

```
filter(players, rank(G) == 1)
```

The following straightforward translation does not work because window functions are only allowed in `SELECT` and `ORDER_BY`.

```
SELECT *
FROM Batting
WHERE rank() OVER (PARTITION BY "playerID" ORDER BY "G") = 1;
```

Computing the window function in `SELECT` and referring to it in `WHERE` or `HAVING` doesn’t work either, because `WHERE` and `HAVING` are computed before windowing functions.

```
SELECT *, rank() OVER (PARTITION BY "playerID" ORDER BY "G") as rank
FROM Batting
WHERE rank = 1;

SELECT *, rank() OVER (PARTITION BY "playerID" ORDER BY "G") as rank
FROM Batting
HAVING rank = 1;
```

Instead, we must use a subquery:

```
SELECT *
FROM (
  SELECT *, rank() OVER (PARTITION BY "playerID" ORDER BY "G") as rank
  FROM Batting
) tmp
WHERE rank = 1;
```

And even that query is a slightly simplification because it will also add a rank column to the original columns. dplyr takes care of generating the full, verbose, query, so you can focus on your data analysis challenges.
