# Supplementary material for: Comparing Kaolin and Pinolene to Improve Sustainable Grapevine Production during Drought
Source: PLoS One. 2016 Jun 13;11(6):e0156631. doi: 10.1371/journal.pone.0156631 (PMC4905681; doi:10.1371/journal.pone.0156631)
Supplement: S1 File — (GZ) [file pone.0156631.s003.gz › PONE_Brillante_et_al_2016_Code_and_Data/packrat/src/dplyr/dplyr_0.4.2.tar.gz!/dplyr/inst/doc/introduction.html]

Introduction to dplyr


# Introduction to dplyr

#### *2015-06-15*

When working with data you must:

- Figure out what you want to do.
- Precisely describe what you want in the form of a computer program.
- Execute the code.

The dplyr package makes each of these steps as fast and easy as possible by:

- Elucidating the most common data manipulation operations, so that your options are helpfully constrained when thinking about how to tackle a problem.
- Providing simple functions that correspond to the most common data manipulation verbs, so that you can easily translate your thoughts into code.
- Using efficient data storage backends, so that you spend as little time waiting for the computer as possible.

The goal of this document is to introduce you to the basic tools that dplyr provides, and show how you to apply them to data frames. Other vignettes provide more details on specific topics:

- databases: as well as in memory data frames, dplyr also connects to databases. It allows you to work with remote, out-of-memory data, using exactly the same tools, because dplyr will translate your R code into the appropriate SQL.
- benchmark-baseball: see how dplyr compares to other tools for data manipulation on a realistic use case.
- window-functions: a window function is a variation on an aggregation function. Where an aggregate function uses `n` inputs to produce 1 output, a window function uses `n` inputs to produce `n` outputs.

## Data: nycflights13

To explore the basic data manipulation verbs of dplyr, we’ll start with the built in `nycflights13` data frame. This dataset contains all 336776 flights that departed from New York City in 2013. The data comes from the US Bureau of Transportation Statistics, and is documented in `?nycflights13`

```
library(nycflights13)
dim(flights)
#> [1] 336776     16
head(flights)
#> Source: local data frame [6 x 16]
#> 
#>    year month day dep_time dep_delay arr_time arr_delay carrier tailnum
#> 1  2013     1   1      517         2      830        11      UA  N14228
#> 2  2013     1   1      533         4      850        20      UA  N24211
#> 3  2013     1   1      542         2      923        33      AA  N619AA
#> 4  2013     1   1      544        -1     1004       -18      B6  N804JB
#> ..  ...   ... ...      ...       ...      ...       ...     ...     ...
#> Variables not shown: flight (int), origin (chr), dest (chr), air_time
#>   (dbl), distance (dbl), hour (dbl), minute (dbl)
```

dplyr can work with data frames as is, but if you’re dealing with large data, it’s worthwhile to convert them to a `tbl_df`: this is a wrapper around a data frame that won’t accidentally print a lot of data to the screen.

## Single table verbs

Dplyr aims to provide a function for each basic verb of data manipulating:

- `filter()` (and `slice()`)
- `arrange()`
- `select()` (and `rename()`)
- `distinct()`
- `mutate()` (and `transmute()`)
- `summarise()`
- `sample_n()` and `sample_frac()`

If you’ve used plyr before, many of these will be familar.

## Filter rows with `filter()`

`filter()` allows you to select a subset of the rows of a data frame. The first argument is the name of the data frame, and the second and subsequent are filtering expressions evaluated in the context of that data frame:

For example, we can select all flights on January 1st with:

```
filter(flights, month == 1, day == 1)
#> Source: local data frame [842 x 16]
#> 
#>    year month day dep_time dep_delay arr_time arr_delay carrier tailnum
#> 1  2013     1   1      517         2      830        11      UA  N14228
#> 2  2013     1   1      533         4      850        20      UA  N24211
#> 3  2013     1   1      542         2      923        33      AA  N619AA
#> 4  2013     1   1      544        -1     1004       -18      B6  N804JB
#> ..  ...   ... ...      ...       ...      ...       ...     ...     ...
#> Variables not shown: flight (int), origin (chr), dest (chr), air_time
#>   (dbl), distance (dbl), hour (dbl), minute (dbl)
```

This is equivalent to the more verbose:

```
flights[flights$month == 1 & flights$day == 1, ]
```

`filter()` works similarly to `subset()` except that you can give it any number of filtering conditions which are joined together with `&` (not `&&` which is easy to do accidentally!). You can use other boolean operators explicitly:

```
filter(flights, month == 1 | month == 2)
```

To select rows by position, use `slice()`:

```
slice(flights, 1:10)
#> Source: local data frame [10 x 16]
#> 
#>    year month day dep_time dep_delay arr_time arr_delay carrier tailnum
#> 1  2013     1   1      517         2      830        11      UA  N14228
#> 2  2013     1   1      533         4      850        20      UA  N24211
#> 3  2013     1   1      542         2      923        33      AA  N619AA
#> 4  2013     1   1      544        -1     1004       -18      B6  N804JB
#> ..  ...   ... ...      ...       ...      ...       ...     ...     ...
#> Variables not shown: flight (int), origin (chr), dest (chr), air_time
#>   (dbl), distance (dbl), hour (dbl), minute (dbl)
```

## Arrange rows with `arrange()`

`arrange()` works similarly to `filter()` except that instead of filtering or selecting rows, it reorders them. It takes a data frame, and a set of column names (or more complicated expressions) to order by. If you provide more than one column name, each additional column will be used to break ties in the values of preceding columns:

```
arrange(flights, year, month, day)
#> Source: local data frame [336,776 x 16]
#> 
#>    year month day dep_time dep_delay arr_time arr_delay carrier tailnum
#> 1  2013     1   1      517         2      830        11      UA  N14228
#> 2  2013     1   1      533         4      850        20      UA  N24211
#> 3  2013     1   1      542         2      923        33      AA  N619AA
#> 4  2013     1   1      544        -1     1004       -18      B6  N804JB
#> ..  ...   ... ...      ...       ...      ...       ...     ...     ...
#> Variables not shown: flight (int), origin (chr), dest (chr), air_time
#>   (dbl), distance (dbl), hour (dbl), minute (dbl)
```

Use `desc()` to order a column in descending order:

```
arrange(flights, desc(arr_delay))
#> Source: local data frame [336,776 x 16]
#> 
#>    year month day dep_time dep_delay arr_time arr_delay carrier tailnum
#> 1  2013     1   9      641      1301     1242      1272      HA  N384HA
#> 2  2013     6  15     1432      1137     1607      1127      MQ  N504MQ
#> 3  2013     1  10     1121      1126     1239      1109      MQ  N517MQ
#> 4  2013     9  20     1139      1014     1457      1007      AA  N338AA
#> ..  ...   ... ...      ...       ...      ...       ...     ...     ...
#> Variables not shown: flight (int), origin (chr), dest (chr), air_time
#>   (dbl), distance (dbl), hour (dbl), minute (dbl)
```

`dplyr::arrange()` works the same way as `plyr::arrange()`. It’s a straighforward wrapper around `order()` that requires less typing. The previous code is equivalent to:

```
flights[order(flights$year, flights$month, flights$day), ]
flights[order(desc(flights$arr_delay)), ]
```

## Select columns with `select()`

Often you work with large datasets with many columns where only a few are actually of interest to you. `select()` allows you to rapidly zoom in on a useful subset using operations that usually only work on numeric variable positions:

```
# Select columns by name
select(flights, year, month, day)
#> Source: local data frame [336,776 x 3]
#> 
#>    year month day
#> 1  2013     1   1
#> 2  2013     1   1
#> 3  2013     1   1
#> 4  2013     1   1
#> ..  ...   ... ...
# Select all columns between year and day (inclusive)
select(flights, year:day)
#> Source: local data frame [336,776 x 3]
#> 
#>    year month day
#> 1  2013     1   1
#> 2  2013     1   1
#> 3  2013     1   1
#> 4  2013     1   1
#> ..  ...   ... ...
# Select all columns except those from year to day (inclusive)
select(flights, -(year:day))
#> Source: local data frame [336,776 x 13]
#> 
#>    dep_time dep_delay arr_time arr_delay carrier tailnum flight origin
#> 1       517         2      830        11      UA  N14228   1545    EWR
#> 2       533         4      850        20      UA  N24211   1714    LGA
#> 3       542         2      923        33      AA  N619AA   1141    JFK
#> 4       544        -1     1004       -18      B6  N804JB    725    JFK
#> ..      ...       ...      ...       ...     ...     ...    ...    ...
#>    dest
#> 1   IAH
#> 2   IAH
#> 3   MIA
#> 4   BQN
#> ..  ...
#> Variables not shown: air_time (dbl), distance (dbl), hour (dbl), minute
#>   (dbl)
```

This function works similarly to the `select` argument to the `base::subset()`. It’s its own function in dplyr, because the dplyr philosophy is to have small functions that each do one thing well.

There are a number of helper functions you can use within `select()`, like `starts_with()`, `ends_with()`, `matches()` and `contains()`. These let you quickly match larger blocks of variable that meet some criterion. See `?select` for more details.

You can rename variables with `select()` by using named arguments:

```
select(flights, tail_num = tailnum)
#> Source: local data frame [336,776 x 1]
#> 
#>    tail_num
#> 1    N14228
#> 2    N24211
#> 3    N619AA
#> 4    N804JB
#> ..      ...
```

But because `select()` drops all the variables not explicitly mentioned, it’s not that useful. Instead, use `rename()`:

```
rename(flights, tail_num = tailnum)
#> Source: local data frame [336,776 x 16]
#> 
#>    year month day dep_time dep_delay arr_time arr_delay carrier tail_num
#> 1  2013     1   1      517         2      830        11      UA   N14228
#> 2  2013     1   1      533         4      850        20      UA   N24211
#> 3  2013     1   1      542         2      923        33      AA   N619AA
#> 4  2013     1   1      544        -1     1004       -18      B6   N804JB
#> ..  ...   ... ...      ...       ...      ...       ...     ...      ...
#> Variables not shown: flight (int), origin (chr), dest (chr), air_time
#>   (dbl), distance (dbl), hour (dbl), minute (dbl)
```

## Extract distinct (unique) rows

A common use of `select()` is to find out which values a set of variables takes. This is particularly useful in conjunction with the `distinct()` verb which only returns the unique values in a table.

```
distinct(select(flights, tailnum))
#> Source: local data frame [4,044 x 1]
#> 
#>    tailnum
#> 1   N14228
#> 2   N24211
#> 3   N619AA
#> 4   N804JB
#> ..     ...
distinct(select(flights, origin, dest))
#> Source: local data frame [224 x 2]
#> 
#>    origin dest
#> 1     EWR  IAH
#> 2     LGA  IAH
#> 3     JFK  MIA
#> 4     JFK  BQN
#> ..    ...  ...
```

(This is very similar to `base::unique()` but should be much faster.)

## Add new columns with `mutate()`

As well as selecting from the set of existing columns, it’s often useful to add new columns that are functions of existing columns. This is the job of `mutate()`:

```
mutate(flights,
  gain = arr_delay - dep_delay,
  speed = distance / air_time * 60)
#> Source: local data frame [336,776 x 18]
#> 
#>    year month day dep_time dep_delay arr_time arr_delay carrier tailnum
#> 1  2013     1   1      517         2      830        11      UA  N14228
#> 2  2013     1   1      533         4      850        20      UA  N24211
#> 3  2013     1   1      542         2      923        33      AA  N619AA
#> 4  2013     1   1      544        -1     1004       -18      B6  N804JB
#> ..  ...   ... ...      ...       ...      ...       ...     ...     ...
#> Variables not shown: flight (int), origin (chr), dest (chr), air_time
#>   (dbl), distance (dbl), hour (dbl), minute (dbl), gain (dbl), speed (dbl)
```

`dplyr::mutate()` works the same way as `plyr::mutate()` and similarly to `base::transform()`. The key difference between `mutate()` and `transform()` is that mutate allows you to refer to columns that you just created:

```
mutate(flights,
  gain = arr_delay - dep_delay,
  gain_per_hour = gain / (air_time / 60)
)
#> Source: local data frame [336,776 x 18]
#> 
#>    year month day dep_time dep_delay arr_time arr_delay carrier tailnum
#> 1  2013     1   1      517         2      830        11      UA  N14228
#> 2  2013     1   1      533         4      850        20      UA  N24211
#> 3  2013     1   1      542         2      923        33      AA  N619AA
#> 4  2013     1   1      544        -1     1004       -18      B6  N804JB
#> ..  ...   ... ...      ...       ...      ...       ...     ...     ...
#> Variables not shown: flight (int), origin (chr), dest (chr), air_time
#>   (dbl), distance (dbl), hour (dbl), minute (dbl), gain (dbl),
#>   gain_per_hour (dbl)
```

```
transform(flights,
  gain = arr_delay - delay,
  gain_per_hour = gain / (air_time / 60)
)
#> Error: object 'gain' not found
```

If you only want to keep the new variables, use `transmute()`:

```
transmute(flights,
  gain = arr_delay - dep_delay,
  gain_per_hour = gain / (air_time / 60)
)
#> Source: local data frame [336,776 x 2]
#> 
#>    gain gain_per_hour
#> 1     9      2.378855
#> 2    16      4.229075
#> 3    31     11.625000
#> 4   -17     -5.573770
#> ..  ...           ...
```

## Summarise values with `summarise()`

The last verb is `summarise()`, which collapses a data frame to a single row. It’s not very useful yet:

```
summarise(flights,
  delay = mean(dep_delay, na.rm = TRUE))
#> Source: local data frame [1 x 1]
#> 
#>      delay
#> 1 12.63907
```

This is exactly equivalent to `plyr::summarise()`.

## Randomly sample rows with `sample_n()` and `sample_frac()`

You can use `sample_n()` and `sample_frac()` to take a random sample of rows, either a fixed number for `sample_n()` or a fixed fraction for `sample_frac()`.

```
sample_n(flights, 10)
#> Source: local data frame [10 x 16]
#> 
#>    year month day dep_time dep_delay arr_time arr_delay carrier tailnum
#> 1  2013     9  26     1816        16     1932        12      WN  N550WN
#> 2  2013     6   3     1453        -2     1810        18      B6  N659JB
#> 3  2013     9  15     1515        -5     1802       -36      DL  N365NB
#> 4  2013     1  23     1503        33     1720        43      EV  N12922
#> ..  ...   ... ...      ...       ...      ...       ...     ...     ...
#> Variables not shown: flight (int), origin (chr), dest (chr), air_time
#>   (dbl), distance (dbl), hour (dbl), minute (dbl)
sample_frac(flights, 0.01)
#> Source: local data frame [3,368 x 16]
#> 
#>    year month day dep_time dep_delay arr_time arr_delay carrier tailnum
#> 1  2013     7  30     1730        -5     1839       -27      DL  N345NW
#> 2  2013     8   6      935        -5     1203        -9      9E  N8839E
#> 3  2013    10  30     1502         2     1742        -9      UA  N36472
#> 4  2013     6  10      842        -8     1026         1      MQ  N815MQ
#> ..  ...   ... ...      ...       ...      ...       ...     ...     ...
#> Variables not shown: flight (int), origin (chr), dest (chr), air_time
#>   (dbl), distance (dbl), hour (dbl), minute (dbl)
```

Use `replace = TRUE` to perform a bootstrap sample, and optionally weight the sample with the `weight` argument.

## Commonalities

You may have noticed that all these functions are very similar:

- The first argument is a data frame.
- The subsequent arguments describe what to do with it, and you can refer to columns in the data frame directly without using `$`.
- The result is a new data frame

Together these properties make it easy to chain together multiple simple steps to achieve a complex result.

These five functions provide the basis of a language of data manipulation. At the most basic level, you can only alter a tidy data frame in five useful ways: you can reorder the rows (`arrange()`), pick observations and variables of interest (`filter()` and `select()`), add new variables that are functions of existing variables (`mutate()`) or collapse many values to a summary (`summarise()`). The remainder of the language comes from applying the five functions to different types of data, like to grouped data, as described next.

# Grouped operations

These verbs are useful, but they become really powerful when you combine them with the idea of “group by”, repeating the operation individually on groups of observations within the dataset. In dplyr, you use the `group_by()` function to describe how to break a dataset down into groups of rows. You can then use the resulting object in exactly the same functions as above; they’ll automatically work “by group” when the input is a grouped.

The verbs are affected by grouping as follows:

- grouped `select()` is the same as ungrouped `select()`, excepted that retains grouping variables are always retained.
- grouped `arrange()` orders first by grouping variables
- `mutate()` and `filter()` are most useful in conjunction with window functions (like `rank()`, or `min(x) == x`), and are described in detail in `vignette("window-functions")`.
- `sample_n()` and `sample_frac()` sample the specified number/fraction of rows in each group.
- `slice()` extracts rows within each group.
- `summarise()` is easy to understand and very useful, and is described in more detail below.

In the following example, we split the complete dataset into individual planes and then summarise each plane by counting the number of flights (`count = n()`) and computing the average distance (`dist = mean(Distance, na.rm = TRUE)`) and delay (`delay = mean(ArrDelay, na.rm = TRUE)`). We then use ggplot2 to display the output.

```
by_tailnum <- group_by(flights, tailnum)
delay <- summarise(by_tailnum,
  count = n(),
  dist = mean(distance, na.rm = TRUE),
  delay = mean(arr_delay, na.rm = TRUE))
delay <- filter(delay, count > 20, dist < 2000)

# Interestingly, the average delay is only slightly related to the
# average distance flown by a plane.
ggplot(delay, aes(dist, delay)) +
  geom_point(aes(size = count), alpha = 1/2) +
  geom_smooth() +
  scale_size_area()
```

You use `summarise()` with **aggregate functions**, which take a vector of values, and return a single number. There are many useful functions in base R like `min()`, `max()`, `mean()`, `sum()`, `sd()`, `median()`, and `IQR()`. dplyr provides a handful of others:

- `n()`: number of observations in the current group
- `n_distinct(x)`: count the number of unique values in `x`.
- `first(x)`, `last(x)` and `nth(x, n)` - these work similarly to `x[1]`, `x[length(x)]`, and `x[n]` but give you more control of the result if the value isn’t present.

For example, we could use these to find the number of planes and the number of flights that go to each possible destination:

```
destinations <- group_by(flights, dest)
summarise(destinations,
  planes = n_distinct(tailnum),
  flights = n()
)
#> Source: local data frame [105 x 3]
#> 
#>    dest planes flights
#> 1   ABQ    108     254
#> 2   ACK     58     265
#> 3   ALB    172     439
#> 4   ANC      6       8
#> ..  ...    ...     ...
```

You can also use any function that you write yourself. For performance, dplyr provides optimised C++ versions of many of these functions. If you want to provide your own C++ function, see the hybrid-evaluation vignette for more details.

When you group by multiple variables, each summary peels off one level of the grouping. That makes it easy to progressively roll-up a dataset:

```
daily <- group_by(flights, year, month, day)
(per_day   <- summarise(daily, flights = n()))
#> Source: local data frame [365 x 4]
#> Groups: year, month
#> 
#>    year month day flights
#> 1  2013     1   1     842
#> 2  2013     1   2     943
#> 3  2013     1   3     914
#> 4  2013     1   4     915
#> ..  ...   ... ...     ...
(per_month <- summarise(per_day, flights = sum(flights)))
#> Source: local data frame [12 x 3]
#> Groups: year
#> 
#>    year month flights
#> 1  2013     1   27004
#> 2  2013     2   24951
#> 3  2013     3   28834
#> 4  2013     4   28330
#> ..  ...   ...     ...
(per_year  <- summarise(per_month, flights = sum(flights)))
#> Source: local data frame [1 x 2]
#> 
#>   year flights
#> 1 2013  336776
```

However you need to be careful when progressively rolling up summaries like this: it’s ok for sums and counts, but you need to think about weighting for means and variances, and it’s not possible to do exactly for medians.

## Chaining

The dplyr API is functional in the sense that function calls don’t have side-effects, and you must always save their results. This doesn’t lead to particularly elegant code if you want to do many operations at once. You either have to do it step-by-step:

```
a1 <- group_by(flights, year, month, day)
a2 <- select(a1, arr_delay, dep_delay)
a3 <- summarise(a2,
  arr = mean(arr_delay, na.rm = TRUE),
  dep = mean(dep_delay, na.rm = TRUE))
a4 <- filter(a3, arr > 30 | dep > 30)
```

Or if you don’t want to save the intermediate results, you need to wrap the function calls inside each other:

```
filter(
  summarise(
    select(
      group_by(flights, year, month, day),
      arr_delay, dep_delay
    ),
    arr = mean(arr_delay, na.rm = TRUE),
    dep = mean(dep_delay, na.rm = TRUE)
  ),
  arr > 30 | dep > 30
)
#> Source: local data frame [49 x 5]
#> Groups: year, month
#> 
#>    year month day      arr      dep
#> 1  2013     1  16 34.24736 24.61287
#> 2  2013     1  31 32.60285 28.65836
#> 3  2013     2  11 36.29009 39.07360
#> 4  2013     2  27 31.25249 37.76327
#> ..  ...   ... ...      ...      ...
```

This is difficult to read because the order of the operations is from inside to out, and the arguments are a long way away from the function. To get around this problem, dplyr provides the `%>%` operator. `x %>% f(y)` turns into `f(x, y)` so you can use it to rewrite multiple operations so you can read from left-to-right, top-to-bottom:

```
flights %>%
  group_by(year, month, day) %>%
  select(arr_delay, dep_delay) %>%
  summarise(
    arr = mean(arr_delay, na.rm = TRUE),
    dep = mean(dep_delay, na.rm = TRUE)
  ) %>%
  filter(arr > 30 | dep > 30)
```

# Other data sources

As well as data frames, dplyr works with data stored in other ways, like data tables, databases and multidimensional arrays.

## Data table

dplyr also provides data table methods for all verbs. If you’re using data.tables already this lets you use dplyr syntax for data manipulation, and data.table for everything else.

For multiple operations, data.table can be faster because you usually use it with multiple verbs at the same time. For example, with data table you can do a mutate and a select in a single step, and it’s smart enough to know that there’s no point in computing the new variable for the rows you’re about to throw away.

The advantages of using dplyr with data tables are:

- For common data manipulation tasks, it insulates you from reference semantics of data.tables, and protects you from accidentally modifying your data.
- Instead of one complex method built on the subscripting operator (`[`), it provides many simple methods.

## Databases

dplyr also allows you to use the same verbs with a remote database. It takes care of generating the SQL for you so that you can avoid the cognitive challenge of constantly swiching between languages. See the databases vignette for more details.

Compared to DBI and the database connection algorithms:

- it hides, as much as possible, the fact that you’re working with a remote database
- you don’t need to know any sql (although it helps!)
- it abstracts over the many differences between the different DBI implementations

## Multidimensional arrays / cubes

`tbl_cube()` provides an experimental interface to multidimensional arrays or data cubes. If you’re using this form of data in R, please get in touch so I can better understand your needs.

# Comparisons

Compared to all existing options, dplyr:

- abstracts away how your data is stored, so that you can work with data frames, data tables and remote databases using the same functions. This lets you think about what you want to achieve, not the logistics of data storage.
- it provides a thoughtful default `print()` method so you don’t accidentally print pages of data to the screen (this was inspired by data table’s output)

Compared to base functions:

- dplyr is much more consistent; functions have the same interface so that once you’ve mastered one, you can easily pick up the others
- base functions tend to be based around vectors; dplyr is centered around data frames

Compared to plyr:

- dplyr is much much faster
- it provides a better thought out set of joins
- it only provides tools for working with data frames (e.g. most of dplyr is equivalent to `ddply()` + various functions, `do()` is equivalent to `dlply()`)

Compared to virtual data frame approaches:

- it doesn’t pretend that you have a data frame: if you want to run lm etc, you’ll still need to manually pull down the data
- it doesn’t provide methods for R summary functions (e.g. `mean()`, or `sum()`)
