# Supplementary material for: Comparing Kaolin and Pinolene to Improve Sustainable Grapevine Production during Drought
Source: PLoS One. 2016 Jun 13;11(6):e0156631. doi: 10.1371/journal.pone.0156631 (PMC4905681; doi:10.1371/journal.pone.0156631)
Supplement: S1 File — (GZ) [file pone.0156631.s003.gz › PONE_Brillante_et_al_2016_Code_and_Data/packrat/src/dplyr/dplyr_0.4.2.tar.gz!/dplyr/inst/doc/databases.html]

Databases


# Databases

#### *2015-06-15*

As well as working with local in-memory data like data frames and data tables, dplyr also works with remote on-disk data stored in databases. Generally, if your data fits in memory there is no advantage to putting it in a database: it will only be slower and more hassle. The reason you’d want to use dplyr with a database is because either your data is already in a database (and you don’t want to work with static csv files that someone else has dumped out for you), or you have so much data that it does not fit in memory and you have to use a database. Currently dplyr supports the three most popular open source databases (sqlite, mysql and postgresql), and google’s bigquery.

Since R almost exclusively works with in-memory data, if you do have a lot of data in a database, you can’t just dump it into R. Instead, you’ll have to work with subsets or aggregates, and dplyr aims to make that as easy as possible. If you’re working with large data, it’s also likely that you’ll need support to get the data into the database and to ensure you have the right indices for good performance. dplyr provides some simple tools to help with these tasks but they are no substitute for a local expert.

The motivation for supporting databases in dplyr is that you never pull down the right subset or aggregate from the database the first time, and usually you have to iterate between R and SQL many times before you get the perfect dataset. Switching between languages is cognitively challenging (especially because R and SQL are so perilously similar), so dplyr allows you to write R code that is automatically translated to SQL. The goal of dplyr is not to replace every SQL function with an R function: that would be difficult and error prone. Instead, dplyr only generates `SELECT` statements, the SQL you write most often as an analyst.

To get the most out of this chapter, you’ll need to be familiar with querying SQL databases using the `SELECT` statement. If you have some familiarity with SQL and you’d like to learn more, I found how indexes work in SQLite and 10 easy steps to a complete understanding of SQL to be particularly helpful.

## Getting started

To experiement with databases, it’s easiest to get started with SQLite because everything you need is included in the R package. You don’t need to install anything else, and you don’t need to deal with the hassle of setting up a database server. Using a SQLite database in dplyr is really easy: just give it a path and the ok to create it.

```
my_db <- src_sqlite("my_db.sqlite3", create = T)
```

The main new concept here is the `src`, which is a collection of tables. Use `src_sqlite()`, `src_mysql()`, `src_postgres()` and `src_bigquery()` to connect to the different databases supported by dplyr.

`my_db` currently has no data in it, so we’ll load it up with the `flights` data using the convenient `copy_to()` function. This is a quick and dirty way of getting data into a database, but it’s not suitable for very large datasets because all the data has to flow through R.

```
library(nycflights13)
flights_sqlite <- copy_to(my_db, flights, temporary = FALSE, indexes = list(
  c("year", "month", "day"), "carrier", "tailnum"))
```

As you can see, the `copy_to()` operation has an additional argument that allows you to supply indexes for the table. Here we set up indexes that will allow us to quickly process the data by day, by carrier and by plane. `copy_to()` also executes the SQL `ANALYZE` command: this ensures that the database has up-to-date table statistics and can pick appropriate query optimisations.

For this particular dataset, there’s a built-in `src` that will cache `flights` in a standard location:

```
flights_sqlite <- tbl(nycflights13_sqlite(), "flights")
#> Caching nycflights db at /private/tmp/Rtmpv9yK7F/Rinste591bbc3024/dplyr/db/nycflights13.sqlite
flights_sqlite
#> Source: sqlite 3.8.6 [/private/tmp/Rtmpv9yK7F/Rinste591bbc3024/dplyr/db/nycflights13.sqlite]
#> From: flights [336,776 x 16]
#> 
#>    year month day dep_time dep_delay arr_time arr_delay carrier tailnum
#> 1  2013     1   1      517         2      830        11      UA  N14228
#> 2  2013     1   1      533         4      850        20      UA  N24211
#> 3  2013     1   1      542         2      923        33      AA  N619AA
#> 4  2013     1   1      544        -1     1004       -18      B6  N804JB
#> ..  ...   ... ...      ...       ...      ...       ...     ...     ...
#> Variables not shown: flight (int), origin (chr), dest (chr), air_time
#>   (dbl), distance (dbl), hour (dbl), minute (dbl)
```

You can also create `tbl` from arbitrary SQL:

```
tbl(my_db, sql("SELECT * FROM flights"))
```

## Basic verbs

Remote data sources use exactly the same five verbs as local data sources:

```
select(flights_sqlite, year:day, dep_delay, arr_delay)
#> Source: sqlite 3.8.6 [/private/tmp/Rtmpv9yK7F/Rinste591bbc3024/dplyr/db/nycflights13.sqlite]
#> From: flights [336,776 x 5]
#> 
#>    year month day dep_delay arr_delay
#> 1  2013     1   1         2        11
#> 2  2013     1   1         4        20
#> 3  2013     1   1         2        33
#> 4  2013     1   1        -1       -18
#> ..  ...   ... ...       ...       ...
filter(flights_sqlite, dep_delay > 240)
#> Source: sqlite 3.8.6 [/private/tmp/Rtmpv9yK7F/Rinste591bbc3024/dplyr/db/nycflights13.sqlite]
#> From: flights [1,524 x 16]
#> Filter: dep_delay > 240 
#> 
#>    year month day dep_time dep_delay arr_time arr_delay carrier tailnum
#> 1  2013     1   1      848       853     1001       851      MQ  N942MQ
#> 2  2013     1   1     1815       290     2120       338      EV  N17185
#> 3  2013     1   1     1842       260     1958       263      EV  N18120
#> 4  2013     1   1     2115       255     2330       250      9E  N924XJ
#> ..  ...   ... ...      ...       ...      ...       ...     ...     ...
#> Variables not shown: flight (int), origin (chr), dest (chr), air_time
#>   (dbl), distance (dbl), hour (dbl), minute (dbl)
arrange(flights_sqlite, year, month, day)
#> Source: sqlite 3.8.6 [/private/tmp/Rtmpv9yK7F/Rinste591bbc3024/dplyr/db/nycflights13.sqlite]
#> From: flights [336,776 x 16]
#> Arrange: year, month, day 
#> 
#>    year month day dep_time dep_delay arr_time arr_delay carrier tailnum
#> 1  2013     1   1      517         2      830        11      UA  N14228
#> 2  2013     1   1      533         4      850        20      UA  N24211
#> 3  2013     1   1      542         2      923        33      AA  N619AA
#> 4  2013     1   1      544        -1     1004       -18      B6  N804JB
#> ..  ...   ... ...      ...       ...      ...       ...     ...     ...
#> Variables not shown: flight (int), origin (chr), dest (chr), air_time
#>   (dbl), distance (dbl), hour (dbl), minute (dbl)
mutate(flights_sqlite, speed = air_time / distance)
#> Source: sqlite 3.8.6 [/private/tmp/Rtmpv9yK7F/Rinste591bbc3024/dplyr/db/nycflights13.sqlite]
#> From: flights [336,776 x 17]
#> 
#>    year month day dep_time dep_delay arr_time arr_delay carrier tailnum
#> 1  2013     1   1      517         2      830        11      UA  N14228
#> 2  2013     1   1      533         4      850        20      UA  N24211
#> 3  2013     1   1      542         2      923        33      AA  N619AA
#> 4  2013     1   1      544        -1     1004       -18      B6  N804JB
#> ..  ...   ... ...      ...       ...      ...       ...     ...     ...
#> Variables not shown: flight (int), origin (chr), dest (chr), air_time
#>   (dbl), distance (dbl), hour (dbl), minute (dbl), speed (dbl)
summarise(flights_sqlite, delay = mean(dep_time))
#> Source: sqlite 3.8.6 [/private/tmp/Rtmpv9yK7F/Rinste591bbc3024/dplyr/db/nycflights13.sqlite]
#> From: <derived table> [?? x 1]
#> 
#>      delay
#> 1  1349.11
#> ..     ...
```

The most important difference is that the expressions in `select()`, `filter()`, `arrange()`, `mutate()`, and `summarise()` are translated into SQL so they can be run on the database. This translation is almost perfect for the most common operations but there are some limitations, which you’ll learn about later.

## Laziness

When working with databases, dplyr tries to be as lazy as possible. It’s lazy in two ways:

- It never pulls data back to R unless you explicitly ask for it.
- It delays doing any work until the last possible minute, collecting together everything you want to do then sending that to the database in one step.

For example, take the following code:

```
c1 <- filter(flights_sqlite, year == 2013, month == 1, day == 1)
c2 <- select(c1, year, month, day, carrier, dep_delay, air_time, distance)
c3 <- mutate(c2, speed = distance / air_time * 60)
c4 <- arrange(c3, year, month, day, carrier)
```

Suprisingly, this sequence of operations never actually touches the database. It’s not until you ask for the data (e.g. by printing `c4`) that dplyr generates the SQL and requests the results from the database, and even then it only pulls down 10 rows.

```
c4
#> Source: sqlite 3.8.6 [/private/tmp/Rtmpv9yK7F/Rinste591bbc3024/dplyr/db/nycflights13.sqlite]
#> From: flights [842 x 8]
#> Filter: year == 2013, month == 1, day == 1 
#> Arrange: year, month, day, carrier 
#> 
#>    year month day carrier dep_delay air_time distance    speed
#> 1  2013     1   1      9E         0      189     1029 326.6667
#> 2  2013     1   1      9E        -9       57      228 240.0000
#> 3  2013     1   1      9E        -3       68      301 265.5882
#> 4  2013     1   1      9E        -6       57      209 220.0000
#> ..  ...   ... ...     ...       ...      ...      ...      ...
```

To pull down all the results use `collect()`, which returns a `tbl_df()`:

```
collect(c4)
#> Source: local data frame [842 x 8]
#> 
#>    year month day carrier dep_delay air_time distance    speed
#> 1  2013     1   1      9E         0      189     1029 326.6667
#> 2  2013     1   1      9E        -9       57      228 240.0000
#> 3  2013     1   1      9E        -3       68      301 265.5882
#> 4  2013     1   1      9E        -6       57      209 220.0000
#> ..  ...   ... ...     ...       ...      ...      ...      ...
```

You can see the query dplyr has generated by looking at the `query` component of the object:

```
c4$query
#> <Query> SELECT "year" AS "year", "month" AS "month", "day" AS "day", "carrier" AS "carrier", "dep_delay" AS "dep_delay", "air_time" AS "air_time", "distance" AS "distance", "distance" / "air_time" * 60.0 AS "speed"
#> FROM "flights"
#> WHERE "year" = 2013.0 AND "month" = 1.0 AND "day" = 1.0
#> ORDER BY "year", "month", "day", "carrier"
#> <SQLiteConnection>
```

You can also ask the database how it plans to execute the query with `explain()`. The output for SQLite is explained in more detail on the SQLite website and is helpful if you’re trying to figure out what indexes are being used.

```
explain(c4)
#> <SQL>
#> SELECT "year" AS "year", "month" AS "month", "day" AS "day", "carrier" AS "carrier", "dep_delay" AS "dep_delay", "air_time" AS "air_time", "distance" AS "distance", "distance" / "air_time" * 60.0 AS "speed"
#> FROM "flights"
#> WHERE "year" = 2013.0 AND "month" = 1.0 AND "day" = 1.0
#> ORDER BY "year", "month", "day", "carrier"
#> 
#> 
#> <PLAN>
#>   selectid order from
#> 1        0     0    0
#> 2        0     0    0
#>                                                                                   detail
#> 1 SEARCH TABLE flights USING INDEX flights_year_month_day (year=? AND month=? AND day=?)
#> 2                                             USE TEMP B-TREE FOR RIGHT PART OF ORDER BY
```

### Forcing computation

There are three ways to force the computation of a query:

- `collect()` executes the query and returns the results to R.
- `compute()` executes the query and stores the results in a temporary table in the database.
- `collapse()` turns the query into a table expresion.

You are most likely to use `collect()`: once you have interactively converged on the right set of operations, use `collect()` to pull down the data into a local `tbl_df()`. If you have some knowledge of SQL, you can use `compute()` and `collapse()` to optimise performance.

### Performance considerations

dplyr tries to prevent you from accidentally performing expensive query operations:

- `nrow()` is always `NA`: in general, there’s no way to determine how many rows a query will return unless you actually run it.
- Printing a tbl only runs the query enough to get the first 10 rows
- You can’t use `tail()` on database tbls: you can’t find the last rows without executing the whole query.

## SQL translation

When doing simple mathematical operations of the form you normally use when filtering, mutating and summarising it’s relatively straightforward to translate R code to SQL (or indeed to any programming language).

To experiment with the translation, use `translate_sql()`. The following examples work through some basic differences between R and SQL.

```
# In SQLite variable names are escaped by double quotes:
translate_sql(x)
#> <SQL> "x"
# And strings are escaped by single quotes
translate_sql("x")
#> <SQL> 'x'

# Many functions have slightly different names
translate_sql(x == 1 && (y < 2 || z > 3))
#> <SQL> "x" = 1.0 AND ("y" < 2.0 OR "z" > 3.0)
translate_sql(x ^ 2 < 10)
#> <SQL> POWER("x", 2.0) < 10.0
translate_sql(x %% 2 == 10)
#> <SQL> "x" % 2.0 = 10.0

# R and SQL have different defaults for integers vs reals.
# In R, 1 is a real, and 1L is an integer
# In SQL, 1 is an integer, and 1.0 is a real
translate_sql(1)
#> <SQL> 1.0
translate_sql(1L)
#> <SQL> 1
```

dplyr knows how to convert the following R functions to SQL:

- basic math operators: `+`, `-`, `*`, `/`, `%%`, `^`
- math functions: `abs`, `acos`, `acosh`, `asin`, `asinh`, `atan`, `atan2`, `atanh`, `ceiling`, `cos`, `cosh`, `cot`, `coth`, `exp`, `floor`, `log`, `log10`, `round`, `sign`, `sin`, `sinh`, `sqrt`, `tan`, `tanh`
- logical comparisons: `<`, `<=`, `!=`, `>=`, `>`, `==`, `%in%`
- boolean operations: `&`, `&&`, `|`, `||`, `!`, `xor`
- basic aggregations: `mean`, `sum`, `min`, `max`, `sd`, `var`

The basic techniques underying the implementation of `translate_sql()` are described in the Advanced R book. `translate_sql()` is built on top of R’s parsing engine and has been carefully designed to generate correct sql. It also protects you against SQL injection attacks by correctly escaping strings and variable names as needed by the database that you’re connecting to.

It’s not possible to provide a perfect translation because databases don’t have all the functions that R does. The goal of dplyr is to provide a semantic translation: to translate what you mean, not the precise details. Even for functions that exist both in databases and R you shouldn’t expect results to be exactly the same; database programmers have different priorities to R core. For example, in R, `mean()` loops through the data twice in order to get a higher level of numerical accuracy at the cost of being twice as slow. R’s `mean()` also provides a `trim` option for computing trimmed means, which databases do not provide. Databases automatically drop NULLs (their equivalent of missing values) whereas in R you have to ask nicely. This means the essense of simple calls like `mean(x)` will be translated accurately, but more complicated calls like `mean(x, trim = 0.5, na.rm = TRUE)` will raise an error:

```
translate_sql(mean(x, trim = T))
# Error: Invalid number of args to SQL AVG. Expecting 1
```

Any function that dplyr doesn’t know how to convert it leaves as is - that means if you want to use any other function that database provides, you can use it as is. Here a couple of examples that will work with SQLite:

```
translate_sql(glob(x, y))
#> <SQL> GLOB("x", "y")
translate_sql(x %like% "ab*")
#> <SQL> "x" LIKE 'ab*'
```

## Grouping

SQLite lacks window functions, which are needed for grouped mutation and filtering. This means that the only really useful operations for grouped sqlite tables are in `summarise()`. The grouped summarise from the introduction translates well - the only difference is that databases always drop NULLs (their equivalent of missing values), so we don’t supply `na.rm = TRUE`.

```
by_tailnum <- group_by(flights_sqlite, tailnum)
delay <- summarise(by_tailnum,
  count = n(),
  dist = mean(distance),
  delay = mean(arr_delay)
)
delay <- filter(delay, count > 20, dist < 2000)
delay_local <- collect(delay)
```

Other databases do support window functions and you can learn about them in the corresponding vignette. It’s sometimes possible to simulate grouped filters and mutates using self joins, where you join the original table with a summarised version, but that topic is beyond the scope of this intro.

## Other databases

Using other databases instead of SQLite works similarly, the overall workflow is the same regardless of what database you’re connecting to. The following sections go in to more details on the pecularities of each database engine. All of these databases follow a client-server model - as well as your computer which is connecting to the databse, there is another computer actually running it (that might be your computer but usually isn’t). Getting one of these databases setup up is beyond the scope of this article, but there are plenty of tutorials available on the web.

### Postgresql

`src_postgres()` has five arguments: `dbname`, `host`, `port`, `user` and `password`. If you are running a local postgresql database with the default settings you’ll only need `dbname`, but in most cases you’ll need all five. dplyr uses the RPostgreSQL package to connect to postgres databases, which means you can’t currently connect to remote databases that require a SSL connection (e.g. Heroku).

For example, the following code allows me to connect to a local postgresql database that contains a copy of the `flights` data:

```
if (has_lahman("postgres")) {
  flights_postgres <- tbl(src_postgres("nycflights13"), "flights")
}
```

Postgres is a considerably more powerful database than SQLite. It has:

- a much wider range of functions built in to the database
- support for window functions, which allow grouped subset and mutates to work.

The following examples shows the grouped filter and mutate possible with PostgreSQL. The SQL generated from the grouped filter is quite complex because you can’t filter on window functions directly; instead they have to go in a subquery.

```
if (has_lahman("postgres")) {
  daily <- group_by(flights_postgres, year, month, day)

  # Find the most and least delayed flight each day
  bestworst <- daily %>% 
    select(flight, arr_delay) %>% 
    filter(arr_delay == min(arr_delay) || arr_delay == max(arr_delay))
  bestworst$query

  # Rank each flight within a daily
  ranked <- daily %>% 
    select(arr_delay) %>% 
    mutate(rank = rank(desc(arr_delay)))
  ranked$query
}
```

### MySQL and MariaDB

You can connect to MySQL and MariaDB (a recent fork of MySQL) through `src_mysql()`, mediated by the RMySQL package. Like PostgreSQL, you’ll need to provide a `dbname`, `username`, `password`, `host`, and `port`.

In terms of functionality, MySQL lies somewhere between SQLite and PostgreSQL. It provides a wider range of built-in functions, but it does not support window functions (so you can’t do grouped mutates and filters).

### Bigquery

Bigquery is a hosted database server provided by google. To connect, you need to provide your `project`, `dataset` and optionally a project for `billing` (if billing for `project` isn’t enabled). After you create the src, your web browser will open and ask you to authenticate. Your credentials are stored in a local cache, so you should only need to do this once.

Bigquery supports only a single SQL statement: SELECT. Fortunately this is all you need for data analysis, and within SELECT bigquery provides comprehensive coverage at a similar level to postgresql.

## Picking a database

If you don’t already have a database, here’s some advice from my experiences setting up and running all of them. SQLite is by far the easiest to get started with, but the lack of window functions makes it limited for data analysis. PostgreSQL is not too much harder to use and has a wide range of built in functions. Don’t bother with MySQL/MariaDB: it’s a pain to set up and the documentation is subpar. Google bigquery might be a good fit if you have very large data, or you’re willing to pay (a small amount of) money for someone else to look after your database.
