# Supplementary material for: Comparing Kaolin and Pinolene to Improve Sustainable Grapevine Production during Drought
Source: PLoS One. 2016 Jun 13;11(6):e0156631. doi: 10.1371/journal.pone.0156631 (PMC4905681; doi:10.1371/journal.pone.0156631)
Supplement: S1 File — (GZ) [file pone.0156631.s003.gz › PONE_Brillante_et_al_2016_Code_and_Data/packrat/src/dplyr/dplyr_0.4.2.tar.gz!/dplyr/inst/doc/data_frames.html]

Data frames


# Data frames

#### *2015-06-15*

## Creating

`data_frame()` is a nice way to create data frames. It encapsulates best practices for data frames:

- It never changes the type of its inputs (i.e. no more `stringsAsFactors = FALSE`!)

  ```
  data.frame(x = letters) %>% sapply(class)
  #>        x 
  #> "factor"
  data_frame(x = letters) %>% sapply(class)
  #>           x 
  #> "character"
  ```

  This makes it easier to use with list-columns:

  ```
  data_frame(x = 1:3, y = list(1:5, 1:10, 1:20))
  #> Source: local data frame [3 x 2]
  #> 
  #>   x         y
  #> 1 1  <int[5]>
  #> 2 2 <int[10]>
  #> 3 3 <int[20]>
  ```

  List-columns are most commonly created by `do()`, but they can be useful to create by hand.
- It never adjusts the names of variables:

  ```
  data.frame(`crazy name` = 1) %>% names()
  #> [1] "crazy.name"
  data_frame(`crazy name` = 1) %>% names()
  #> [1] "crazy name"
  ```
- It evaluates its arguments lazyily and in order:

  ```
  data_frame(x = 1:5, y = x ^ 2)
  #> Source: local data frame [5 x 2]
  #> 
  #>    x  y
  #> 1  1  1
  #> 2  2  4
  #> 3  3  9
  #> 4  4 16
  #> .. . ..
  ```
- It adds `tbl_df()` class to output so that if you accidentaly print a large data frames you only get the first few rows.

  ```
  data_frame(x = 1:5) %>% class()
  #> [1] "tbl_df"     "tbl"        "data.frame"
  ```
- It never uses `row.names()`, because the whole point of tidy data is to store variables in a consistent way, so we shouldn’t put one variable in a special attribute.
- It only recycles vectors of length 1. Recycling vectors of other lengths is a frequent source of bugs.

## Coercion

To complement `data_frame()`, dplyr provides `as_data_frame()` for coercing lists into data frames. It does two things:

- Checks that the input list is valid for a data frame, i.e. that each element is named, is a 1d atomic vector or list, and all elements have the same length.
- Sets the class and attributes of the list to make it behave like a data frame. This modification does not require a deep copy of the input list, so is very fast.

This is much simpler than `as.data.frame()`. It’s hard to explain precisely what `as.data.frame()` does, but it’s similar to `do.call(cbind, lapply(x, data.frame))` - i.e. it coerces each component to a data frame and then `cbinds()` them all together. Consequently `as_data_frame()` is much faster than `as.data.frame()`:

```
l2 <- replicate(26, sample(100), simplify = FALSE)
names(l2) <- letters
microbenchmark::microbenchmark(
  as_data_frame(l2),
  as.data.frame(l2)
)
#> Unit: microseconds
#>               expr      min       lq      mean   median       uq      max
#>  as_data_frame(l2)  104.167  111.977  127.4641  119.241  137.789  208.559
#>  as.data.frame(l2) 1474.734 1531.581 1751.9712 1579.507 1839.423 3307.849
#>  neval cld
#>    100  a 
#>    100   b
```

The speed of `as.data.frame()` is not usually a bottleneck in interatively use, but can be a problem when combining thousands of messy inputs into one tidy data frame.

## Memory

One of the reasons that dplyr is fast is that it is very careful about when it makes copies of columns. This section describes how this works, and gives you some useful tools for understanding the memory usage of data frames in R.

The first tool we’ll use is `dplyr::location()`. It tells us three things about a data frame:

- where the object itself is located in memory
- where each column is located
- where each attribute is located

```
location(iris)
#> <0x7f9ef3601c20>
#> Variables:
#>  * Sepal.Length: <0x7f9ef7ff7a00>
#>  * Sepal.Width:  <0x7f9ef43d8c00>
#>  * Petal.Length: <0x7f9ef4771000>
#>  * Petal.Width:  <0x7f9ef4483e00>
#>  * Species:      <0x7f9ef2d50630>
#> Attributes:
#>  * names:        <0x7f9ef3601c88>
#>  * row.names:    <0x7f9ef2d07300>
#>  * class:        <0x7f9ef53b7ec8>
```

It’s useful to know the memory address, because if the address changes, then you know R has made a copy. Copies are bad because it takes time to copy a vector. This isn’t usually a bottleneck if you have a few thousand values, but if you have millions or tens of millions it starts to take up a significant amount of time. Unnecessary copies are also bad because they take up memory.

R tries to avoid making copies where possible. For example, if you just assign `iris` to another variable, it continues to the point same location:

```
iris2 <- iris
location(iris2)
#> <0x7f9ef3601c20>
#> Variables:
#>  * Sepal.Length: <0x7f9ef7ff7a00>
#>  * Sepal.Width:  <0x7f9ef43d8c00>
#>  * Petal.Length: <0x7f9ef4771000>
#>  * Petal.Width:  <0x7f9ef4483e00>
#>  * Species:      <0x7f9ef2d50630>
#> Attributes:
#>  * names:        <0x7f9ef3601c88>
#>  * row.names:    <0x7f9ef2ca5bb0>
#>  * class:        <0x7f9ef53b7ec8>
```

Rather than carefully comparing long memory locations, we can instead use the `dplyr::changes()` function to highlights changes between two versions of a data frame. This shows us that `iris` and `iris2` are identical: both names point to the same location in memory.

```
changes(iris2, iris)
#> <identical>
```

What do you think happens if you modify a single column of `iris2`? In R 3.1.0 and above, R knows enough to only modify one column and leave the others pointing to the existing location:

```
iris2$Sepal.Length <- iris2$Sepal.Length * 2
changes(iris, iris2)
#> Changed variables:
#>              old            new           
#> Sepal.Length 0x7f9ef7ff7a00 0x7f9ef74cd200
#> 
#> Changed attributes:
#>              old            new           
#> row.names    0x7f9ef2c9c590 0x7f9ef2c9c810
```

(This was not the case prior to R 3.1.0: R created a deep copy of the entire data frame.)

dplyr is similarly smart:

```
iris3 <- mutate(iris, Sepal.Length = Sepal.Length * 2)
changes(iris3, iris)
#> Changed variables:
#>              old            new           
#> Sepal.Length 0x7f9ef591b800 0x7f9ef7ff7a00
#> 
#> Changed attributes:
#>              old            new           
#> class        0x7f9ef535eb78 0x7f9ef53b7ec8
#> names        0x7f9ef8bbb470 0x7f9ef3601c88
#> row.names    0x7f9ef6208610 0x7f9ef6250b50
```

It’s smart enough to create only one new column: all the other columns continue to point at their old locations. You might notice that the attributes have still been copied. This has little impact on performance because the attributes are usually short vectors and copying makes the internal dplyr code considerably simpler.

dplyr never makes copies unless it has to:

- `tbl_df()` and `group_by()` don’t copy columns
- `select()` never copies columns, even when you rename them
- `mutate()` never copies columns, except when you modify an existing column
- `arrange()` must copy because you’re changing the order of every column. This is an expensive operation for big data, but you can generally avoid it using the order argument to window functions
- `summarise()` creates new data, but it’s usually at least an order of magnitude smaller than the original data.

This means that dplyr lets you work with data frames with very little memory overhead.

data.table takes this idea one step further than dplyr, and provides functions that modify a data table in place. This avoids the need to copy the pointers to existing columns and attributes, and provides speed up when you have many columns. dplyr doesn’t do this with data frames (although it could) because I think it’s safer to keep data immutable: all dplyr data frame methods return a new data frame, even while they share as much data as possible.
