# Supplementary material for: Comparing Kaolin and Pinolene to Improve Sustainable Grapevine Production during Drought
Source: PLoS One. 2016 Jun 13;11(6):e0156631. doi: 10.1371/journal.pone.0156631 (PMC4905681; doi:10.1371/journal.pone.0156631)
Supplement: S1 File — (GZ) [file pone.0156631.s003.gz › PONE_Brillante_et_al_2016_Code_and_Data/packrat/src/ggplot2/ggplot2_1.0.1.tar.gz!/ggplot2/inst/staticdocs/head.html]

{{pagetitle}}. {{#package}}{{package}} {{version}}{{/package}}
