# Supplementary material for: Comparing Kaolin and Pinolene to Improve Sustainable Grapevine Production during Drought
Source: PLoS One. 2016 Jun 13;11(6):e0156631. doi: 10.1371/journal.pone.0156631 (PMC4905681; doi:10.1371/journal.pone.0156631)
Supplement: S1 File — (GZ) [file pone.0156631.s003.gz › PONE_Brillante_et_al_2016_Code_and_Data/packrat/src/ggplot2/ggplot2_1.0.1.tar.gz!/ggplot2/inst/doc/release.html]

Releasing a new version of ggplot2


# Releasing a new version of ggplot2

## Release candidate phase

After issues resolved for a given release:

1. Pass R CMD check.
2. In DESCRIPTION and NEWS, remove the .99 version suffix and increment the
   version number. For example, 0.9.2.99 becomes 0.9.3.
3. Do the same for any packages that ggplot2 depends on, such as scales and
   gtable.
4. Update ggplot2's Import dependency versions to use the final release numbers
   of scales and gtable.
5. Commit these changes to a branch with `ggplot2-<version>-rc`, and push the
   branch.
6. Check packages that depend on ggplot2 with `devtools::revdep_check()` and
   run visual tests.
7. Email ggplot2, ggplot2-dev, and bcc the maintainers of packages that depend
   on ggplot2 (`revdep_maintainers("ggplot2")`).

   ```
   Hi all,

   We're very please to announce a release candidate for ggplot2 1.0.0! This
   release celebrates the ggplot2 community: all improvements have been 
   contributed via pull requests.

   We've made every effort to make sure that your existing ggplot2 graphics
   continue to work. ggplot2 1.0.0 has passed R CMD check, all our existing 
   visual tests, and R CMD check on all dependencies. But it's still possible 
   that some bugs may have crept in, so we'd really appreciate it if you'd 
   try it out. It's easy to install the development version: first install 
   devtools, then run `devtools::install_github("hadley/ggplot2@ggplot2-1.0.0-rc")`.

   We plan to submit ggplot2 to cran in two weeks, May 9. Please let us know if 
   you have any problems - your feedback is much appreciated. (If you're pretty
   sure you've discovered a new bug, please start a new thread or file
   an issue on github, otherwise it's a bit hard to track what's going on).

   Hadley & Winston
   ```
8. Notify cran:

   ```
   Dear CRAN maintainers,

   ggplot2 1.0.0 has entered the release candidate phase and will be
   submitted to CRAN in two weeks.

   Included below is the email that I sent to the ggplot2 mailing and all
   maintainers of packages that depend on ggplot2.

   Regards,

   Hadley
   ```

If problems arise during the RC period, make fixes on the branch. Those fixes
later get merged back into master.

## Release

When the package is accepted on CRAN:

1. Create a new release at https://github.com/hadley/ggplot2/releases.
   The tag name should be of the form `v1.0.0`.
2. If any Check out the new branch, or merge it into master. (Need to get off the
   rc branch so it can be deleted):

   ```
   VERSION=1.0.0
   git checkout v$VERSION
   # Or
   git checkout master
   git merge v$VERSION
   ```
3. Delete the `-rc` branch, with:

   ```
   git branch -d v$VERSION-rc
   git push origin :v$VERSION-rc
   ```
4. Once you push the tag github, travis will automatically build the website.
