# Supplementary material for: Comparing Kaolin and Pinolene to Improve Sustainable Grapevine Production during Drought
Source: PLoS One. 2016 Jun 13;11(6):e0156631. doi: 10.1371/journal.pone.0156631 (PMC4905681; doi:10.1371/journal.pone.0156631)
Supplement: S1 File — (GZ) [file pone.0156631.s003.gz › PONE_Brillante_et_al_2016_Code_and_Data/packrat/src/ggplot2/ggplot2_1.0.1.tar.gz!/ggplot2/inst/doc/development.html]

Contributing to ggplot2 development


# Contributing to ggplot2 development

The goal of this guide is to help you get up and contributing to ggplot2 as quickly as possible. It's still a work in progress and very rough. Your feedback is much appreciated and so are pull requests :). Rather than emailing me directly about questions, please discuss ggplot2 development issues on the ggplot2-dev mailing list. That way multiple people can learn at the same time.

To contribute a change to ggplot2, you follow these steps:

1. Set up a local ggplot2 development environment.
2. Create a branch in git and make your changes.
3. Push branch to github and issue pull request (PR).
4. Discuss the pull request.
5. Iterate until either we accept the PR or decide that it's not
   a good fit for ggplot2.

Each of these steps are described in more detail below. This might feel overwhelming the first time you get set up, but it gets easier with practice. If you get stuck at any point, please reach out for help on the ggplot2-dev mailing list

## Basic setup

If you want to do development on ggplot2 and share the changes with other people, you'll have to set up an account on GitHub. You'll make a fork of Hadley's repository and store it on GitHub. You'll also make a copy of your repository on your local machine.

1. Join github.
2. Set up git and github.
3. Fork of the main repo by going to https://github.com/hadley/ggplot2/fork.
   A fork is a copy of ggplot2 that you can work on independently.
   See fork a repo for
   more details.
4. Clone your fork to your local computer (change `myname` to your account
   name):

   ```
   git clone git@github.com:myname/ggplot2.git
   cd ggplot2
   ```
5. Once you've done that, add the main repository as a remote and set up
   the master branch to track the main ggplot2 repo. This will make it
   easier to keep up to date.

   ```
   git remote add hadley git://github.com/hadley/ggplot2.git
   git branch --set-upstream master hadley/master
   ```

Now on your local machine you have the local repository and two remote repositories: `origin` (the default), which is your personal ggplot2 repo at GitHub, and `hadley` which is Hadley's. You'll be able to push changes to your own repository only.

### Development environment

You'll also need a copy of the packages I use for package development. Get them by running the following R code:

```
install.packages(c("devtools", "testthat"))
devtools::install_github("klutometis/roxygen")
```

Next, install all the suggested packages that ggplot2 needs. To do this either open the ggplot2 rstudio project, or set your working directory to the ggplot2 directory, then run:

```
install_deps(dep = T)
```

The key functions you'll use when working on ggplot2:

- `devtools::load_all()`: loads all code into your current environment
- `devtools::document()`: updates the roxygen comments
- `devtools::test()`: run all unit tests
- `devtools::check()`: runs R CMD check on the package to check for errors.

If you're using Rstudio, I highly recommend learning the keyboard shortcuts for these commands. You can find them in the `Build` menu.

## Making changes

When you want to make changes, you should work on a new branch. Otherwise things can get a bit confusing when it comes time to merge it into the main repo with a pull request. You probably want to start with the `master` branch.

```
# Fetch the latest version of hadley/ggplot2
git fetch hadley
git checkout hadley/master
```

At this point it'll give you a warning about being in “detached HEAD” state. Don't worry about it. Just start a new branch with the current state as the starting point:

```
git checkout -b myfix
```

To check what branch you're currently on, run `git branch`.

Now you can make your changes and commit them to this branch on your local repository. If you decide you want to start over, you can just check out `hadley/master` again, make a new branch, and begin anew.

When you feel like sharing your changes, push them to your GitHub repo:

```
git push
```

Then you can submit a pull request if you want it to be integrated in the main branch.

If you've been working on your for a while, it's possible that it won't merge properly because something has changed in both the main repo and in your branch. You can test it out by checking out the main branch and merging yourself.

First, make a new branch called `testmerge`, based off the main branch:

```
git fetch hadley
git checkout hadley/master

git checkout -b testmerge
```

Then try merging your branch into testmerge:

```
git merge myfix
```

If there are no errors, great. You can switch back to your `myfix` branch and delete `testmerge`:

```
git checkout myfix
git branch -D testmerge
```

If there are any merge conflicts, you may want to rebase your changes on top of the current master version, or just resolve the conflicts and commit it to your branch. Rebasing may make for a somewhat cleaner commit history, but there is a possibility of messing things up. If you want to be safe, you can just make a new branch and rebase that on top of the current master.

## What makes a good pull request?

Pull requests will be evaluated against the a seven point checklist:

1. **Motivation**. Your pull request must clearly and concisely motivates the
   need for change. Unfortunately neither Winston nor I have much time to
   work on ggplot2 these days, so you need to describe the problem and show
   how your pull request solves it as concisely as possible.

   Also include this motivation in `NEWS` so that when a new release of
   ggplot2 comes out it's easy for users to see what's changed. Add your
   item at the top of the file and use markdown for formatting. The
   news item should end with `(@yourGithubUsername, #the_issue_number)`.
2. **Only related changes**. Before you submit your pull request, please
   check to make sure that you haven't accidentally included any unrelated
   changes. These make it harder to see exactly what's changed, and to
   evaluate any unexpected side effects.

   Each PR corresponds to a git branch, so if you expect to submit
   multiple changes make sure to create multiple branches. If you have
   multiple changes that depend on each other, start with the first one
   and don't submit any others until the first one has been processed.
3. **Use ggplot2 coding style**. Please follow the
   official ggplot2 style. Maintaing
   a consistent style across the whole code base makes it much easier to
   jump into the code.
4. If you're adding new parameters or a new function, you'll also need
   to document them with roxygen.
   Make sure to re-run `devtools::document()` on the code before submitting.

   Currently, ggplot2 uses the development version of roxygen2, which you
   can get with `install_github("klutometis/roxygen")`. This will be
   available on CRAN in the near future.
5. If fixing a bug or adding a new feature to a non-graphical function,
   please add a testthat unit test.
6. If fixing a bug in the visual output, please add a visual test.
   (Instructions to follow soon)
7. If you're adding a new graphical feature, please add a short example
   to the appropriate function.

This seems like a lot of work but don't worry if your pull request isn't perfect. It's a learning process and Winston and I will be on hand to help you out. A pull request is a process, and unless you've submitted a few in the past it's unlikely that your pull request will be accepted as is.

Finally, remember that ggplot2 is a mature package used by thousands of people. This means that it's extremely difficult (i.e. impossible) to change any existing functionality without breaking someone's code (or another package on CRAN). Please don't submit pull requests that change existing behaviour. Instead, think about how you can add a new feature in a minimally invasive way.

## Advanced techniques

### Fetching a branch from someone else's repository

Sometimes you will want to fetch a branch from someone's repository, but without going to the trouble of seting it up as a remote. This is handy if you just want to quickly try out a someone's work.

This will fetch a branch from someone's remote repository and check it out (replace `username` and `somebranch`):

```
git fetch https://github.com/username/ggplot2.git somebranch
git checkout FETCH_HEAD
```

Just doing the above won't create a local branch – you'll be in “detached HEAD” state. If you'd like to create a local branch to work on, run (you can replace `somebranch` with whatever name you like):

```
git checkout -b somebranch
```

### Adding other repositories as remotes

If you often work off of someone else's repository, it can be useful to add their repo as a *remote*. This makes it easier to fetch changes in their repository. If the person's GitHub account is `otherdevel`, you would do the following:

```
git remote add otherdevel git://github.com/otherdevel/ggplot2.git
git fetch otherdevel

git checkout otherdevel/somebranch
```

If you don't want to follow them any more, run:

```
git remote rm otherdevel
```

### Delete a branch from GitHub

If you pushed a branch to your GitHub repo but it's no longer needed there, you can remove it with:

```
git push origin :mybranch
```

### Visualizing the development tree

GitHub has a very nice development tree view, but it of course only shows commits that have been pushed to GitHub. You may also want to view the tree on your local machine, to see how your local changes relate to the main tree. There are a number of programs out there that will do this.

Mac:

- gitk: Pretty basic, included with git. Run `gitk -a` to view all branches (by default it just shows you the current branch).
- gitx: This is a bit nicer than gitk.
- SourceTree: This is also a nice program. Normally it costs money, but it is temporarily free from the web page or the Mac App store.

Linux:

- gitk: (See gitk in Mac section)
- gitg: This is nicer than gitk. By default it only shows the current branch; select “Local Branches” or “All Branches” to view others.
