# Supplementary material for: Comparing Kaolin and Pinolene to Improve Sustainable Grapevine Production during Drought
Source: PLoS One. 2016 Jun 13;11(6):e0156631. doi: 10.1371/journal.pone.0156631 (PMC4905681; doi:10.1371/journal.pone.0156631)
Supplement: S1 File — (GZ) [file pone.0156631.s003.gz › PONE_Brillante_et_al_2016_Code_and_Data/packrat/src/lazyeval/lazyeval_0.1.10.tar.gz!/lazyeval/inst/doc/lazyeval.html]

Lazyeval: a new approach to NSE


# Lazyeval: a new approach to NSE

#### *2015-01-02*

This document outlines a new approach to non-standard evaluation (NSE). There are three key ideas:

- Instead of using `substitute()`, use `lazyeval::lazy()` to capture both expression and environment. (Or use `lazyeval::lazy_dots(...)` to capture promises in `...`)
- Every function that uses NSE should have a standard evaluation (SE) escape hatch that does the actual computation. The SE-function name should end with `_`.
- The SE-function has a flexible input specification to make it easy for people to program with.

## `lazy()`

The key tool that makes this approach possible is `lazy()`, an equivalent to `substitute()` that captures both expression and environment associated with a function argument:

```
library(lazyeval)
f <- function(x = a - b) {
  lazy(x)
}
f()
#> <lazy>
#>   expr: a - b
#>   env:  <environment: 0x7fd2f3a406b0>
f(a + b)
#> <lazy>
#>   expr: a + b
#>   env:  <environment: R_GlobalEnv>
```

As a complement to `eval()`, the lazy package provides `lazy_eval()` that uses the environment associated with the lazy object:

```
a <- 10
b <- 1
lazy_eval(f())
#> [1] 9
lazy_eval(f(a + b))
#> [1] 11
```

The second argument to lazy eval is a list or data frame where names should be looked up first:

```
lazy_eval(f(), list(a = 1))
#> [1] 0
```

`lazy_eval()` also works with formulas, since they contain the same information as a lazy object: an expression (only the RHS is used by convention) and an environment:

```
lazy_eval(~ a + b)
#> [1] 11
h <- function(i) {
  ~ 10 + i
}
lazy_eval(h(1))
#> [1] 11
```

## Standard evaluation

Whenever we need a function that does non-standard evaluation, always write the standard evaluation version first. For example, let’s implement our own version of `subset()`:

```
subset2_ <- function(df, condition) {
  r <- lazy_eval(condition, df)
  r <- r & !is.na(r)
  df[r, , drop = FALSE]
} 

subset2_(mtcars, lazy(mpg > 31))
#>     mpg cyl disp hp drat   wt qsec vs am gear carb
#> 18 32.4   4 78.7 66 4.08 2.20 19.5  1  1    4    1
#> 20 33.9   4 71.1 65 4.22 1.83 19.9  1  1    4    1
```

`lazy_eval()` will always coerce it’s first argument into a lazy object, so a variety of specifications will work:

```
subset2_(mtcars, ~mpg > 31)
#>     mpg cyl disp hp drat   wt qsec vs am gear carb
#> 18 32.4   4 78.7 66 4.08 2.20 19.5  1  1    4    1
#> 20 33.9   4 71.1 65 4.22 1.83 19.9  1  1    4    1
subset2_(mtcars, quote(mpg > 31))
#>     mpg cyl disp hp drat   wt qsec vs am gear carb
#> 18 32.4   4 78.7 66 4.08 2.20 19.5  1  1    4    1
#> 20 33.9   4 71.1 65 4.22 1.83 19.9  1  1    4    1
subset2_(mtcars, "mpg > 31")
#>     mpg cyl disp hp drat   wt qsec vs am gear carb
#> 18 32.4   4 78.7 66 4.08 2.20 19.5  1  1    4    1
#> 20 33.9   4 71.1 65 4.22 1.83 19.9  1  1    4    1
```

Note that quoted called and strings don’t have environments associated with them, so `as.lazy()` defaults to using `baseenv()`. This will work if the expression is self-contained (i.e. doesn’t contain any references to variables in the local environment), and will otherwise fail quickly and robustly.

## Non-standard evaluation

With the SE version in hand, writing the NSE version is easy. We just use `lazy()` to capture the unevaluated expression and corresponding environment:

```
subset2 <- function(df, condition) {
  subset2_(df, lazy(condition))
}
subset2(mtcars, mpg > 31)
#>     mpg cyl disp hp drat   wt qsec vs am gear carb
#> 18 32.4   4 78.7 66 4.08 2.20 19.5  1  1    4    1
#> 20 33.9   4 71.1 65 4.22 1.83 19.9  1  1    4    1
```

This standard evaluation escape hatch is very important because it allows us to implement different NSE approaches. For example, we could create a subsetting function that finds all rows where a variable is above a threshold:

```
above_threshold <- function(df, var, threshold) {
  cond <- interp(~ var > x, var = lazy(var), x = threshold)
  subset2_(df, cond)
}
above_threshold(mtcars, mpg, 31)
#>     mpg cyl disp hp drat   wt qsec vs am gear carb
#> 18 32.4   4 78.7 66 4.08 2.20 19.5  1  1    4    1
#> 20 33.9   4 71.1 65 4.22 1.83 19.9  1  1    4    1
```

Here we’re using `interp()` to modify a formula. We use the value of `threshold` and the expression in by `var`.

## Scoping

Because `lazy()` captures the environment associated with the function argument, we automatically avoid a subtle scoping bug present in `subset()`:

```
x <- 31
f1 <- function(...) {
  x <- 30
  subset(mtcars, ...)
}
# Uses 30 instead of 31
f1(mpg > x)
#>     mpg cyl disp  hp drat   wt qsec vs am gear carb
#> 18 32.4   4 78.7  66 4.08 2.20 19.5  1  1    4    1
#> 19 30.4   4 75.7  52 4.93 1.61 18.5  1  1    4    2
#> 20 33.9   4 71.1  65 4.22 1.83 19.9  1  1    4    1
#> 28 30.4   4 95.1 113 3.77 1.51 16.9  1  1    5    2

f2 <- function(...) {
  x <- 30
  subset2(mtcars, ...)
}
# Correctly uses 31
f2(mpg > x)
#>     mpg cyl disp hp drat   wt qsec vs am gear carb
#> 18 32.4   4 78.7 66 4.08 2.20 19.5  1  1    4    1
#> 20 33.9   4 71.1 65 4.22 1.83 19.9  1  1    4    1
```

`lazy()` has another advantage over `substitute()` - by default, it follows promises across function invocations. This simplifies the casual use of NSE.

```
x <- 31
g1 <- function(comp) {
  x <- 30
  subset(mtcars, comp)
}
g1(mpg > x)
#> Error: object 'mpg' not found
```

```
g2 <- function(comp) {
  x <- 30
  subset2(mtcars, comp)
}
g2(mpg > x)
#>     mpg cyl disp hp drat   wt qsec vs am gear carb
#> 18 32.4   4 78.7 66 4.08 2.20 19.5  1  1    4    1
#> 20 33.9   4 71.1 65 4.22 1.83 19.9  1  1    4    1
```

Note that `g2()` doesn’t have a standard-evaluation escape hatch, so it’s not suitable for programming with in the same way that `subset2_()` is.
