# Supplementary material for: Comparing Kaolin and Pinolene to Improve Sustainable Grapevine Production during Drought
Source: PLoS One. 2016 Jun 13;11(6):e0156631. doi: 10.1371/journal.pone.0156631 (PMC4905681; doi:10.1371/journal.pone.0156631)
Supplement: S1 File — (GZ) [file pone.0156631.s003.gz › PONE_Brillante_et_al_2016_Code_and_Data/packrat/src/lazyeval/lazyeval_0.1.10.tar.gz!/lazyeval/inst/doc/chained-promises.html]

Chained promises


# Chained promises

#### *2015-01-02*

Take the following example:

```
library(lazyeval)
f1 <- function(x) lazy(x)
g1 <- function(y) f1(y)

g1(a + b)
#> <lazy>
#>   expr: a + b
#>   env:  <environment: R_GlobalEnv>
```

`lazy()` returns `a + b` because it always tries to find the top-level promise.

In this case the process looks like this:

1. Find the object that `x` is bound to.
2. It’s a promise, so find the expr it’s bound to (`y`, a symbol) and the environment in which it should be evaluated (the environment of `g()`).
3. Since `x` is bound to a symbol, look up its value: it’s bound to a promise.
4. That promise has expression `a + b` and should be evaluated in the global environment.
5. The expression is not a symbol, so stop.

Occasionally, you want to avoid this recursive behaviour, so you can use `follow_symbol = FALSE`:

```
f2 <- function(x) lazy(x, .follow_symbols = FALSE)
g2 <- function(y) f2(y)

g2(a + b)
#> <lazy>
#>   expr: x
#>   env:  <environment: 0x7fd2f61f0408>
```

Either way, if you evaluate the lazy expression you’ll get the same result:

```
a <- 10
b <- 1

lazy_eval(g1(a + b))
#> [1] 11
lazy_eval(g2(a + b))
#> [1] 11
```
