# Supplementary material for: Comparing Kaolin and Pinolene to Improve Sustainable Grapevine Production during Drought
Source: PLoS One. 2016 Jun 13;11(6):e0156631. doi: 10.1371/journal.pone.0156631 (PMC4905681; doi:10.1371/journal.pone.0156631)
Supplement: S1 File — (GZ) [file pone.0156631.s003.gz › PONE_Brillante_et_al_2016_Code_and_Data/packrat/src/lazyeval/lazyeval_0.1.10.tar.gz!/lazyeval/inst/doc/benchmark.html]

Benchmarks


# Benchmarks

#### *2015-01-02*

There is some overhead of using `lazy()` instead of `substitute()`, however, on my computer it’s on the order of around 1.5µs, so it’s extremely unlikely to be a bottleneck in practice.

```
library(microbenchmark)
library(lazyeval)

microbenchmark(
  substitute = substitute(x),
  lazy = lazy(x)
)
#> Unit: nanoseconds
#>        expr  min   lq median   uq   max neval
#>  substitute  172  186    266  328 12807   100
#>        lazy 1609 1691   1802 1981 68902   100
```
