# Supplementary material for: Comparing Kaolin and Pinolene to Improve Sustainable Grapevine Production during Drought
Source: PLoS One. 2016 Jun 13;11(6):e0156631. doi: 10.1371/journal.pone.0156631 (PMC4905681; doi:10.1371/journal.pone.0156631)
Supplement: S1 File — (GZ) [file pone.0156631.s003.gz › PONE_Brillante_et_al_2016_Code_and_Data/packrat/src/magrittr/magrittr_1.5.tar.gz!/magrittr/inst/doc/magrittr.html]

Abstract


*This version: November, 2014. Stefan Milton Bache*

# Abstract

*The magrittr* (to be pronounced with a sophisticated french accent) is
a package with two aims: to decrease development time and to improve
readability and maintainability of code. Or even shortr: to make your code smokin' (puff puff)!

To archive its humble aims, *magrittr* (remember the accent) provides a new
“pipe”-like operator, `%>%`, with which you may pipe a value forward into an
expression or function call; something along the lines of `x %>% f`, rather
than `f(x)`. This is not an unknown feature
elsewhere; a prime example is the `|>` operator used extensively in `F#`
(to say the least) and indeed this – along with Unix pipes – served as a
motivation for developing the magrittr package.

This vignette describes the main features of *magrittr* and demonstrates
some features which has been added since the initial release.

# Introduction and basics

At first encounter, you may wonder whether an operator such as `%>%` can really
be all that beneficial; but as you may notice, it semantically changes your
code in a way that makes it more intuitive to both read and write.

Consider the following example, in which the `mtcars` dataset shipped with
R is munged a little.

```
library(magrittr)

car_data <- 
  mtcars %>%
  subset(hp > 100) %>%
  aggregate(. ~ cyl, data = ., FUN = . %>% mean %>% round(2)) %>%
  transform(kpl = mpg %>% multiply_by(0.4251)) %>%
  print
```

```
  cyl   mpg  disp    hp drat   wt  qsec   vs   am gear carb    kpl
1   4 25.90 108.0 111.0 3.94 2.15 17.75 1.00 1.00 4.50 2.00 11.010
2   6 19.74 183.3 122.3 3.59 3.12 17.98 0.57 0.43 3.86 3.43  8.391
3   8 15.10 353.1 209.2 3.23 4.00 16.77 0.00 0.14 3.29 3.50  6.419
```

We start with a value, here `mtcars` (a `data.frame`). Based on this, we
first extract a subset, then we aggregate the information based on the number
of cylinders, and then we transform the dataset by adding a variable
for kilometers per liter as supplement to miles per gallon. Finally we print
the result before assigning it.
Note how the code is arranged in the logical
order of how you think about the task: data->transform->aggregate, which
is also the same order as the code will execute. It's like a recipe – easy to
read, easy to follow!

A horrific alternative would be to write

```
car_data <- 
  transform(aggregate(. ~ cyl, 
                      data = subset(mtcars, hp > 100), 
                      FUN = function(x) round(mean(x, 2))), 
            kpl = mpg*0.4251)
```

There is a lot more clutter with parentheses, and the mental task of deciphering
the code is more challenging—in particular if you did not write it yourself.

Note also how “building” a function on the fly for use in `aggregate` is very
simple in *magrittr*: rather than an actual value as left-hand side in
pipeline, just use the placeholder. This is also very useful in R's
`*apply` family of functions.

Granted: you may make the second example better, perhaps throw in a few temporary
variables (which is often avoided to some degree when using *magrittr*),
but one often sees cluttered lines like the ones presented.

And here is another selling point. Suppose I want to quickly want to add
another step somewhere in the process. This is very easy in the
to do in the pipeline version, but a little more challenging in the
“standard” example.

The combined example shows a few neat features of the pipe (which it is not):

1. By default the left-hand side (LHS) will be *piped in* as the first argument of
   the function appearing on the right-hand side (RHS). This is the case in the
   `subset` and `transform` expressions.
2. `%>%` may be used in a nested fashion, e.g. it may appear in expressions within
   arguments. This is used in the `mpg` to `kpl` conversion.
3. When the LHS is needed at a position other than the first, one can use
   the dot,`'.'`, as placeholder. This is used in the `aggregate` expression.
4. The dot in e.g. a formula is *not* confused with a placeholder, which is
   utilized in the `aggregate` expression.
5. Whenever only *one* argument is needed, the LHS, then one can omit the
   empty parentheses. This is used in the call to `print` (which also returns its
   argument). Here, `LHS %>% print()`, or even `LHS %>% print(.)` would also work.
6. A pipeline with a dot (`.`) as LHS will create a unary function. This is
   used to define the aggregator function.

One feature, which was not utilized above is piping into *anonymous functions*,
or *lambdas*. This is possible using standard function definitions, e.g.

```
car_data %>%
(function(x) {
  if (nrow(x) > 2) 
    rbind(head(x, 1), tail(x, 1))
  else x
})
```

However, *magrittr* also allows a short-hand notation:

```
car_data %>%
{ 
  if (nrow(.) > 0)
    rbind(head(., 1), tail(., 1))
  else .
}
```

```
  cyl mpg disp  hp drat wt qsec vs am gear carb    kpl
1   4  26  108 111    4  2   18  1  1    4    2 11.053
3   8  15  350 192    3  4   17  0  0    3    4  6.377
```

Since all right-hand sides are really “body expressions” of unary functions, this
is only the natural extension the simple right-hand side expressions. Of course
longer and more complex functions can be made using this approach.

In the first example the anonymous function is enclosed in parentheses.
Whenever you want to use a function- or call-generating statement as right-hand side,
parentheses are used to evaluate the right-hand side before piping takes place.

Another, less useful example is:

```
1:10 %>% (substitute(f(), list(f = sum)))
```

```
[1] 55
```

# Additional pipe operators

*magrittr* also provides three related pipe operators. These are not as
common as `%>%` but they become useful in special cases.

The “tee” operator, `%T>%` works like `%>%`, except it returns the left-hand
side value, and not the result of the right-hand side operation.
This is useful when a step in a pipeline is used for its side-effect (printing,
plotting, logging, etc.). As an example (where the actual plot is omitted here):

```
rnorm(200) %>%
matrix(ncol = 2) %T>%
plot %>% # plot usually does not return anything. 
colSums
```

```
[1]  6.916 -1.605
```

The “exposition” pipe operator, `%$%` exposes the names within the left-hand side
object to the right-hand side expression. Essentially, it is a short-hand for
using the `with` functions (and the same left-hand side objects are accepted).
This operator is handy when functions do not themselves have a data argument, as for
example `lm` and `aggregate` do. Here are a few examples as illustration:

```
iris %>%
  subset(Sepal.Length > mean(Sepal.Length)) %$%
  cor(Sepal.Length, Sepal.Width)

data.frame(z = rnorm(100)) %$% 
  ts.plot(z)
```

Finally, the compound assignment pipe operator `%<>%` can be used as the first pipe
in a chain. The effect will be that the result of the pipeline is assigned to the
left-hand side object, rather than returning the result as usual. It is essentially
shorthand notation for expressions like `foo <- foo %>% bar %>% baz`, which
boils down to `foo %<>% bar %>% baz`. Another example is

```
iris$Sepal.Length %<>% sqrt
```

The `%<>%` can be used whenever `expr <- ...` makes sense, e.g.

- `x %<>% foo %>% bar`
- `x[1:10] %<>% foo %>% bar`
- `x$baz %<>% foo %>% bar`

# Aliases

In addition to the `%>%`-operator, *magrittr* provides some aliases for other
operators which make operations such as addition or multiplication fit well
into the *magrittr*-syntax. As an example, consider:

```
rnorm(1000)    %>%
multiply_by(5) %>%
add(5)         %>%
{ 
   cat("Mean:", mean(.), 
       "Variance:", var(.), "\n")
   head(.)
}
```

```
Mean: 5.04 Variance: 23.25
```

```
[1]  10.019 -10.231  -3.341  -4.997  -1.728   8.690
```

which could be written in more compact form as

```
rnorm(100) %>% `*`(5) %>% `+`(5) %>% 
{
  cat("Mean:", mean(.), "Variance:", var(.),  "\n")
  head(.)
}
```

To see a list of the aliases, execute e.g. `?multiply_by`.

# Development

The *magrittr* package is also available in a development version at the
GitHub development page:
github.com/smbache/magrittr.
