# Supplementary material for: Comparing Kaolin and Pinolene to Improve Sustainable Grapevine Production during Drought
Source: PLoS One. 2016 Jun 13;11(6):e0156631. doi: 10.1371/journal.pone.0156631 (PMC4905681; doi:10.1371/journal.pone.0156631)
Supplement: S1 File — (GZ) [file pone.0156631.s003.gz › PONE_Brillante_et_al_2016_Code_and_Data/packrat/src/multcomp/multcomp_1.4-0.tar.gz!/multcomp/inst/doc/generalsiminf.pdf]

Simultaneous Inference in General Parametric Models *

Torsten Hothorn
Institut für Statistik
Ludwig-Maximilians-Universität München
Ludwigstraße 33, D–80539 München, Germany

Frank Bretz
Statistical Methodology, Clinical Information Sciences
Novartis Pharma AG
CH-4002 Basel, Switzerland

Peter Westfall
Texas Tech University
Lubbock, TX 79409, U.S.A

March 5, 2015

Abstract

Simultaneous inference is a common problem in many areas of application. If multiple null hypotheses are tested simultaneously, the probability of rejecting erroneously at least one of them increases beyond the pre-specified significance level. Simultaneous inference procedures have to be used which adjust for multiplicity and thus control the overall type I error rate. In this paper we describe simultaneous inference procedures in general parametric models, where the experimental questions are specified through a linear combination of elemental model parameters. The framework described here is quite general and extends the canonical theory of multiple comparison procedures in ANOVA models to linear regression problems, generalized linear models, linear mixed effects models, the Cox model, robust linear models, etc. Several examples using a variety of different statistical models illustrate the breadth

*This is a preprint of an article published in Biometrical Journal, Volume 50, Number 3, 346–363. Copyright © 2008 WILEY-VCH Verlag GmbH & Co. KGaA, Weinheim; available online http://www.biometrical-journal.com.

of the results. For the analyses we use the R add-on package **multcomp**, which provides a convenient interface to the general approach adopted here.

Key words: multiple tests, multiple comparisons, simultaneous confidence intervals, adjusted p-values, multivariate normal distribution, robust statistics.

1 Introduction

Multiplicity is an intrinsic problem of any simultaneous inference. If each of k, say, null hypotheses is tested at nominal level α, the overall type I error rate can be substantially larger than α. That is, the probability of at least one erroneous rejection is larger than α for $k \geq 2$. Common multiple comparison procedures adjust for multiplicity and thus ensure that the overall type I error remains below the pre-specified significance level α. Examples of such multiple comparison procedures include Dunnett's many-to-one comparisons, Tukey's all-pairwise comparisons, sequential pairwise contrasts, comparisons with the average, changepoint analyses, dose-response contrasts, etc. These procedures are all well established for classical regression and ANOVA models allowing for covariates and/or factorial treatment structures with i.i.d. normal errors and constant variance, see Bretz et al. (2008) and the references therein. For a general reading on multiple comparison procedures we refer to Hochberg and Tamhane (1987) and Hsu (1996).

In this paper we aim at a unified description of simultaneous inference procedures in parametric models with generally correlated parameter estimates. Each individual null hypothesis is specified through a linear combination of elemental model parameters and we allow for k of such null hypotheses to be tested simultaneously, regardless of the number of elemental model parameters p. The general framework described here extends the current canonical theory with respect to the following aspects: (i) model assumptions such as normality and homoscedasticity are relaxed, thus allowing for simultaneous inference in generalized linear models, mixed effects models, survival models, etc.; (ii) arbitrary linear functions of the elemental parameters are allowed, not just contrasts of means in AN(C)OVA models; (iii) computing the reference distribution is feasible for arbitrary designs, especially for unbalanced designs; and (iv) a unified implementation is provided which allows for a fast transition of the theoretical results to the desks of data analysts interested in simultaneous inferences for multiple hypotheses.

Accordingly, the paper is organized as follows. Section 2 defines the general model and obtains the asymptotic or exact distribution of linear functions of elemental model parameters under rather weak conditions. In Section 3 we describe the framework for simultaneous inference procedures in general parametric models. An overview about important applications of the methodology is given in Section 4 followed by a short discussion of the software implementation in Section 5. Most interesting from a practical point of view is Section 6 where we analyze four rather challenging problems with the tools developed in this paper.

2 Model and Parameters

In this section we introduce the underlying model assumptions and derive some asymptotic results necessary in the subsequent sections. The results from this section form the basis for the simultaneous inference procedures described in Section 3.

Let $\mathcal{M}((\mathbf{Z}_1, \ldots, \mathbf{Z}_n), \theta, \eta)$ denote a semi-parametric statistical model. The set of n observations is described by $(\mathbf{Z}_1, \ldots, \mathbf{Z}_n)$. The model contains fixed but unknown elemental parameters $\theta \in \mathbb{R}^p$ and other (random or nuisance) parameters η. We are primarily interested in the linear functions $\vartheta := \mathbf{K}\theta$ of the parameter vector θ as specified through the constant matrix $\mathbf{K} \in \mathbb{R}^{k,p}$. In what follows we describe the underlying model assumptions, the limiting distribution of estimates of our parameters of interest ϑ, as well as the corresponding test statistics for hypotheses about ϑ and their limiting joint distribution.

Suppose $\hat{\theta}_n \in \mathbb{R}^p$ is an estimate of θ and $\mathsf{S}_n \in \mathbb{R}^{p,p}$ is an estimate of $\mathrm{cov}(\hat{\theta}_n)$ with

$$a_n \mathsf{S}_n \xrightarrow{\mathbb{P}} \Sigma \in \mathbb{R}^{p,p} \tag{1}$$

for some positive, nondecreasing sequence a_n. Furthermore, we assume that a multivariate central limit theorem holds, i.e.,

$$a_n^{1/2}(\hat{\theta}_n - \theta) \xrightarrow{d} \mathcal{N}_p(0, \Sigma). \tag{2}$$

If both (1) and (2) are fulfilled we write $\hat{\theta}_n \overset{a}{\sim} \mathcal{N}_p(\theta, \mathsf{S}_n)$. Then, by Theorem 3.3.A in Serfling (1980), the linear function $\hat{\vartheta}_n = \mathbf{K}\hat{\theta}_n$, i.e., an estimate of our parameters of interest, also follows an approximate multivariate normal distribution

$$\hat{\vartheta}_n = \mathbf{K}\hat{\theta}_n \overset{a}{\sim} \mathcal{N}_k(\vartheta, \mathsf{S}_n^\star)$$

with covariance matrix $\mathsf{S}_n^\star := \mathbf{K}\mathsf{S}_n\mathbf{K}^\top$ for any fixed matrix $\mathbf{K} \in \mathbb{R}^{k,p}$. Thus we need not to distinguish between elemental parameters θ or derived parameters $\vartheta = \mathbf{K}\theta$ that are of interest to the researcher. Instead we simply assume for the moment that we have (in analogy to (1) and (2))

$$\hat{\vartheta}_n \overset{a}{\sim} \mathcal{N}_k(\vartheta, \mathsf{S}_n^\star) \text{ with } a_n \mathsf{S}_n^\star \xrightarrow{\mathbb{P}} \Sigma^\star := \mathbf{K}\Sigma\mathbf{K}^\top \in \mathbb{R}^{k,k} \tag{3}$$

and that the k parameters in ϑ are themselves the parameters of interest to the researcher. It is assumed that the diagonal elements of the covariance matrix are positive, i.e., $\Sigma_{jj}^\star > 0$ for $j = 1, \ldots, k$.

Then, the standardized estimator $\hat{\vartheta}_n$ is again asymptotically normally distributed

$$\mathbf{T}_n := \mathbf{D}_n^{-1/2}(\hat{\vartheta}_n - \vartheta) \overset{a}{\sim} \mathcal{N}_k(0, \mathbf{R}_n) \tag{4}$$

where $\mathbf{D}_n = \mathrm{diag}(\mathsf{S}_n^\star)$ is the diagonal matrix given by the diagonal elements of $\mathsf{S}_n^\star$ and

$$\mathbf{R}_n = \mathbf{D}_n^{-1/2}\mathsf{S}_n^\star\mathbf{D}_n^{-1/2} \in \mathbb{R}^{k,k}$$

is the correlation matrix of the k-dimensional statistic $\mathbf{T}_n$. To demonstrate (4), note that with (3) we have $a_n \mathsf{S}_n^\star \xrightarrow{\mathbb{P}} \Sigma^\star$ and $a_n \mathbf{D}_n \xrightarrow{\mathbb{P}} \operatorname{diag}(\Sigma^\star)$. Define the sequence $\tilde{a}_n$ needed to establish $\tilde{a}$-convergence in (4) by $\tilde{a}_n \equiv 1$. Then we have

$$\begin{aligned} \tilde{a}_n \mathbf{R}_n &= \mathbf{D}_n^{-1/2} \mathsf{S}_n^\star \mathbf{D}_n^{-1/2} \\ &= (a_n \mathbf{D}_n)^{-1/2} (a_n \mathsf{S}_n^\star)(a_n \mathbf{D}_n)^{-1/2} \\ &\xrightarrow{\mathbb{P}} \operatorname{diag}(\Sigma^\star)^{-1/2}\, \Sigma^\star \operatorname{diag}(\Sigma^\star)^{-1/2} =: \mathbf{R} \in \mathbb{R}^{k,k} \end{aligned}$$

where the convergence in probability to a constant follows from Slutzky's Theorem (Theorem 1.5.4, Serfling, 1980) and therefore (4) holds. To finish note that

$$\mathbf{T}_n = \mathbf{D}_n^{-1/2}(\hat{\vartheta}_n - \vartheta) = (a_n \mathbf{D}_n)^{-1/2} a_n^{1/2} (\hat{\vartheta}_n - \vartheta) \xrightarrow{d} \mathcal{N}_k(0, \mathbf{R}).$$

For the purposes of multiple comparisons, we need convergence of multivariate probabilities calculated for the vector $\mathbf{T}_n$ when $\mathbf{T}_n$ is assumed normally distributed with $\mathbf{R}_n$ treated as if it were the true correlation matrix. However, such probabilities $\mathbb{P}(\max(|\mathbf{T}_n| \leq t)$ are continuous functions of $\mathbf{R}_n$ (and a critical value t) which converge by $\mathbf{R}_n \xrightarrow{\mathbb{P}} \mathbf{R}$ as a consequence of Theorem 1.7 in Serfling (1980). In cases where $\mathbf{T}_n$ is assumed multivariate t distributed with $\mathbf{R}_n$ treated as the estimated correlation matrix, we have similar convergence as the degrees of freedom approach infinity.

Since we only assume that the parameter estimates are asymptotically normally distributed with a consistent estimate of the associated covariance matrix being available, our framework covers a large class of statistical models, including linear regression and ANOVA models, generalized linear models, linear mixed effects models, the Cox model, robust linear models, etc. Standard software packages can be used to fit such models and obtain the estimates $\hat{\theta}_n$ and S_n which are essentially the only two quantities that are needed for what follows in Section 3. It should be noted that the elemental parameters θ are not necessarily means or differences of means in AN(C)OVA models. Also, we do not restrict our attention to contrasts of such means, but allow for any set of constants leading to the linear functions $\vartheta = \mathbf{K}\theta$ of interest. Specific examples for $\mathbf{K}$ and θ will be given later in Sections 4 and 6.

3 Global and Simultaneous Inference

Based on the results from Section 2, we now focus on the derivation of suitable inference procedures. We start considering the general linear hypothesis (Searle, 1971) formulated in terms of our parameters of interest ϑ

$$H_0 : \vartheta := \mathbf{K}\theta = \mathbf{m}.$$

Under the conditions of H_0 it follows from Section 2 that

$$\mathbf{T}_n = \mathbf{D}_n^{-1/2}(\hat{\vartheta}_n - \mathbf{m}) \overset{a}{\sim} \mathcal{N}_k(0, \mathbf{R}_n).$$

This approximating distribution will now be used as the reference distribution when constructing the inference procedures. The global hypothesis H_0 can be tested using standard global tests, such as the F- or the χ^2-test. An alternative approach is to use maximum tests, as explained in Subsection 3.1. Note that a small global p-value (obtained from one of these procedures) leading to a rejection of H_0 does not give further indication about the nature of the significant result. Therefore, one is often interested in the individual null hypotheses

$$H_0^j : \vartheta_j = \mathbf{m}_j.$$

Testing the hypotheses set $\{H_0^1, \ldots, H_0^k\}$ simultaneously thus requires the individual assessments while maintaining the familywise error rate, as discussed in Subsection 3.2

At this point it is worth considering two special cases. A stronger assumption than asymptotic normality of $\hat{\theta}_n$ in (2) is exact normality, i.e., $\hat{\theta}_n \sim \mathcal{N}_p(\theta, \Sigma)$. If the covariance matrix Σ is known, it follows by standard arguments that $\mathbf{T}_n \sim \mathcal{N}_k(0, \mathbf{R})$, when $\mathbf{T}_n$ is normalized using fixed, known variances. Otherwise, in the typical situation of linear models with normal i.i.d. errors, $\Sigma = \sigma^2\mathbf{A}$, where σ^2 is unknown but $\mathbf{A}$ is fixed and known, the exact distribution of $\mathbf{T}_n$ is a k-dimensional multivariate $t_k(\nu, \mathbf{R})$ distribution with ν degrees of freedom ($\nu = n - p - 1$ for linear models), see Tong (1990).

3.1 Global Inference

The F- and the χ^2-test are classical approaches to assess the global null hypothesis H_0. Standard results (such as Theorem 3.5, Serfling, 1980) ensure that

$$\begin{aligned} X^2 &= \mathbf{T}_n^\top \mathbf{R}_n^+ \mathbf{T}_n \xrightarrow{d} \chi^2(\text{Rank}(\mathbf{R})) \quad \text{when } \hat{\theta}_n \overset{a}{\sim} \mathcal{N}_p(\theta, \mathsf{S}_n) \\ F &= \frac{\mathbf{T}_n^\top \mathbf{R}^+ \mathbf{T}_n}{\text{Rank}(\mathbf{R})} \sim \mathcal{F}(\text{Rank}(\mathbf{R}), \nu) \quad \text{when } \hat{\theta}_n \sim \mathcal{N}_p(\theta, \sigma^2\mathbf{A}), \end{aligned}$$

where $\text{Rank}(\mathbf{R})$ and ν are the corresponding degrees of freedom of the χ^2 and $\mathcal{F}$ distribution, respectively. Furthermore, $\text{Rank}(\mathbf{R}_n)^+$ denotes the Moore-Penrose inverse of the correlation matrix $\text{Rank}(\mathbf{R})$.

Another suitable scalar test statistic for testing the global hypothesis H_0 is to consider the maximum of the individual test statistics $T_{1,n}, \ldots, T_{k,n}$ of the multivariate statistic $\mathbf{T}_n = (T_{1,n}, \ldots, T_{k,n})$, leading to a max-$t$ type test statistic $\max(|\mathbf{T}_n|)$. The distribution of this statistic under the conditions of H_0 can be handled through the k-dimensional distribution

$$\mathbb{P}(\max(|\mathbf{T}_n|) \le t) \cong \int_{-t}^{t} \cdots \int_{-t}^{t} \varphi_k(x_1, \ldots, x_k; \mathbf{R}, \nu)\, dx_1 \cdots dx_k =: g_\nu(\mathbf{R}, t) \tag{5}$$

for some $t \in \mathbb{R}$, where φ_k is the density function of either the limiting k-dimensional multivariate normal (with $\nu = \infty$ and the '$\approx$' operator) or the exact multivariate $t_k(\nu, \mathbf{R})$-distribution (with $\nu < \infty$ and the '$=$' operator). Since $\mathbf{R}$ is usually unknown, we plug-in the consistent estimate $\mathbf{R}_n$ as discussed in Section 2. The resulting global p-value (exact or approximate, depending on context) for H_0 is $1 - g_\nu(\mathbf{R}_n, \max|\mathbf{t}|)$ when $\mathbf{T} = \mathbf{t}$ has been observed. Efficient methods for approximating the above multivariate normal and t integrals are described in Genz (1992); Genz and Bretz (1999); Bretz et al. (2001) and Genz and Bretz (2002).

In contrast to the global F- or χ^2-test, the max-t test based on the test statistic $\max(|\mathbf{T}_n|)$ also provides information, which of the k individual null hypotheses $H_0^j, j = 1, \dots, k$ is significant, as well as simultaneous confidence intervals, as shown in the next subsection.

3.2 Simultaneous Inference

We now consider testing the k null hypotheses $H_0^1, \dots, H_0^k$ individually and require that the familywise error rate, i.e., the probability of falsely rejecting at least one true null hypothesis, is bounded by the nominal significance level $\alpha \in (0, 1)$. In what follows we use adjusted p-values to describe the decision rules. Adjusted p-values are defined as the smallest significance level for which one still rejects an individual hypothesis H_0^j, given a particular multiple test procedure. In the present context of single-step tests, the (at least asymptotic) adjusted p-value for the jth individual two-sided hypothesis $H_0^j : \vartheta_j = \mathbf{m}_j, j = 1, \dots, k$, is given by

$$p_j = 1 - g_\nu(\mathbf{R}_n, |t_j|),$$

where $t_1, \dots, t_k$ denote the observed test statistics. By construction, we can reject an individual null hypothesis H_0^j, $j = 1, \dots, k$, whenever the associated adjusted p-value is less than or equal to the pre-specified significance level α, i.e., $p_j \leq \alpha$. The adjusted p-values are calculated from expression (5).

Similar results also hold for one-sided testing problems. The adjusted p-values for one-sided cases are defined analogously, using one-sided multidimensional integrals instead of the two-sided integrals (5). Again, we refer to Genz (1992); Genz and Bretz (1999); Bretz et al. (2001) and Genz and Bretz (2002) for the numerical details.

In addition to a simultaneous test procedure, a (at least approximate) simultaneous $(1 - 2\alpha) \times 100\%$ confidence interval for ϑ is given by

$$\hat{\vartheta}_n \pm q_\alpha \mathbf{D}_n^{1/2}$$

where q_α is the $1 - \alpha$ quantile of the distribution (asymptotic, if necessary) of $\mathbf{T}_n$. This quantile can be calculated or approximated via (5), i.e., q_α is chosen such that $g_\nu(\mathbf{R}_n, q_\alpha) = 1 - \alpha$. The corresponding one-sided versions are defined analogously.

It should be noted that the simultaneous inference procedures described so far belong to the class of single-step procedures, since a common critical value q_α is used for the individual tests. Single-step procedures have the advantage that corresponding simultaneous confidence intervals are easily available, as previously noted. However, single-step procedures can always be improved by stepwise extensions based on the closed test procedure. That is, for a given family of null hypotheses $H_0^1, \dots, H_0^k$, an individual hypothesis H_0^j is rejected only if all intersection hypotheses $H_J = \bigcap_{i \in J} H_0^i$ with $j \in J \subseteq \{1, \dots, k\}$ are rejected (Marcus et al., 1976). Such stepwise extensions can thus be applied to any of the methods discussed in this paper, see for example Westfall (1997) and Westfall and Tobias (2007).

4 Applications

The methodological framework described in Sections 2 and 3 is very general and thus applicable to a wide range of statistical models. Many estimation techniques, such as (restricted) maximum likelihood and M-estimation, provide at least asymptotically normal estimates of the elemental parameters together with consistent estimates of their covariance matrix. In this section we illustrate the generality of the methodology by reviewing some potential applications. Detailed numerical examples are discussed in Section 6. In what follows, we assume $\mathbf{m} = 0$ only for the sake of simplicity. The next paragraphs highlight a subjective selection of some special cases of practical importance.

Multiple Linear Regression. In standard regression models the observations $\mathbf{Z}_i$ of subject $i = 1, \dots, n$ consist of a response variable Y_i and a vector of covariates $\mathbf{X}_i = (X_{i1}, \dots, X_{iq})$, such that $\mathbf{Z}_i = (Y_i, \mathbf{X}_i)$ and $p = q + 1$. The response is modelled by a linear combination of the covariates with normal error ε_i and constant variance σ^2,

$$Y_i = \beta_0 + \sum_{j=1}^{q} \beta_j X_{ij} + \sigma \varepsilon_i,$$

where $\varepsilon = (\varepsilon_1, \dots, \varepsilon_n)^\top \sim \mathcal{N}_n(0, \mathbf{I}_n)$. The elemental parameter vector is $\theta = (\beta_0, \beta_1, \dots, \beta_q)$, which is usually estimated by

$$\hat{\theta}_n = \left(\mathbf{X}^\top \mathbf{X}\right)^{-1} \mathbf{X}^\top \mathbf{Y} \sim \mathcal{N}_{q+1}\left(\theta, \sigma^2 \left(\mathbf{X}^\top \mathbf{X}\right)^{-1}\right),$$

where $\mathbf{Y} = (Y_1, \dots, Y_n)$ denotes the response vector and $\mathbf{X} = (1, (X_{ij}))_{ij}$ denotes the design matrix, $i = 1, \dots, n, j = 1, \dots, q$. Thus, for every matrix $\mathbf{K} \in \mathbb{R}^{k, q+1}$ of constants determining the experimental questions of interest we have

$$\hat{\vartheta}_n = \mathbf{K}\hat{\theta}_n \sim \mathcal{N}_k(\mathbf{K}\theta, \sigma^2 \mathbf{K} \left(\mathbf{X}^\top \mathbf{X}\right)^{-1} \mathbf{K}^\top).$$

Under the null hypothesis $\vartheta = 0$ the standardized test statistic follows a multivariate t distribution

$$\mathbf{T}_n = \mathbf{D}_n^{-1/2}\hat{\vartheta}_n \sim t_{q+1}(n-q, \mathbf{R}),$$

where $\mathbf{D}_n = \hat{\sigma}^2 \text{diag}(\mathbf{K}\left(\mathbf{X}^\top\mathbf{X}\right)^{-1}\mathbf{K}^\top)$ is the diagonal matrix of the estimated variances of $\mathbf{K}\hat{\theta}$ and $\mathbf{R}$ is the correlation matrix as given in Section 3. The body fat prediction example presented in Subsection 6.2 illustrates the application of simultaneous inference procedures in the context of variable selection in linear regression models.

One-way ANOVA. Consider a one-way ANOVA model for a factor measured at q levels with a continuous response

$$Y_{ij} = \mu + \gamma_j + \varepsilon_{ij} \tag{6}$$

and independent normal errors $\varepsilon_{ij} \sim \mathcal{N}_1(0, \sigma^2), j = 1, \dots, q, i = 1, \dots, n_j$. Note that the model description in (6) is overparameterized. A standard approach is to consider a suitable re-parametrization. The so-called "treatment contrast" vector $\theta = (\mu, \gamma_2 - \gamma_1, \gamma_3 - \gamma_1, \dots, \gamma_q - \gamma_1)$ is, for example, the default re-parametrization used as elemental parameters in the R-system for statistical computing (R Development Core Team, 2008).

Many classical multiple comparison procedures can be embedded into this framework, including Dunnett's many-to-one comparisons and Tukey's all-pairwise comparisons. For Dunnett's procedure, the differences $\gamma_j - \gamma_1$ are tested for all $j = 2, \dots, q$, where γ_1 denotes the mean treatment effect of a control group. In the notation from Section 2 we thus have

$$\mathbf{K}_{\text{Dunnett}} = (0, \text{diag}(q))$$

resulting in the parameters of interest

$$\vartheta_{\text{Dunnett}} = \mathbf{K}_{\text{Dunnett}}\theta = (\gamma_2 - \gamma_1, \gamma_3 - \gamma_1, \dots, \gamma_q - \gamma_1)$$

of interest. For Tukey's procedure, the interest is in all-pairwise comparisons of the parameters $\gamma_1, \dots, \gamma_q$. For $q = 3$, for example, we have

$$\mathbf{K}_{\text{Tukey}} = \begin{pmatrix} 0 & 1 & 0 \\ 0 & 0 & 1 \\ 0 & 1 & -1 \end{pmatrix}$$

with parameters of interest

$$\vartheta_{\text{Tukey}} = \mathbf{K}_{\text{Tukey}}\theta = (\gamma_2 - \gamma_1, \gamma_3 - \gamma_1, \gamma_2 - \gamma_3).$$

Many further multiple comparison procedures have been investigated in the past, which all fit into this framework. We refer to Bretz et al. (2001) for a related comprehensive list.

Note that under the standard ANOVA assumptions of i.i.d. normal errors with constant variance the vector of test statistics $\mathbf{T}_n$ follows a multivariate t distribution. Thus, related simultaneous tests and confidence intervals do not rely on asymptotics and can be computed analytically instead, as shown in Section 3. To illustrate simultaneous inference procedures in one-way ANOVA models, we consider all pairwise comparisons of expression levels for various genetic conditions of alcoholism in Subsection 6.1.

Further parametric models. In *generalized linear models*, the exact distribution of the parameter estimates is usually unknown and thus the asymptotic normal distribution is the basis for all inference procedures. When we are interested in inference about model parameters corresponding to levels of a certain factor, the same multiple comparison procedures as sketched above are available.

Linear and non-linear mixed effects models fitted by restricted maximum-likelihood provide the data analyst with asymptotically normal estimates and a consistent covariance matrix as well so that all assumptions of our framework are met and one can set up simultaneous inference procedures for these models as well. The same is true for the *Cox model* or other parametric survival models such as the *Weibull model*.

We use logistic regression models to estimated the probability of suffering from Alzheimer's disease in Subsection 6.3, compare several risk factors for survival of leukemia patients by means of a Weibull model in Subsection 6.4 and obtain probability estimates of deer browsing for various tree species from mixed models in Subsection 6.5.

Robust simultaneous inference. Yet another application is to use robust variants of the previously discussed statistical models. One possibility is to consider the use of sandwich estimators S_n for the covariance matrix $\operatorname{cov}(\hat{\theta}_n)$ when, for example, the variance homogeneity assumption is violated. An alternative is to apply robust estimation techniques in linear models, for example S-, M- or MM-estimation (see Rousseeuw and Leroy, 2003; Stefanski and Boos, 2002; Yohai, 1987, for example), which again provide us with asymptotically normal estimates. The reader is referred to Subsection 6.2 for some numerical examples illustrating these ideas.

5 Implementation

The **multcomp** package (Hothorn et al., 2008) in R (R Development Core Team, 2008) provides a general implementation of the framework for simultaneous inference in semi-parametric models described in Sections 2 and 3. The numerical examples in Section 6 will all be analyzed using the **multcomp** package. In this section we briefly introduce the user-interface and refer the reader to the online documentation of the package for the technical details.

Estimated model coefficients $\hat{\theta}_n$ and their estimated covariance matrix S_n are accessible in R via `coef()` and `vcov()` methods available for most statistical models in R, such as objects of class *lm*, *glm*, *coxph*, *nlme*, *mer* or *survreg*. Having this information at hand, the `glht()` function sets up the general linear hypothesis for a model '`model`' and a representation of the matrix $\mathbf{K}$ (via its `linfct` argument):

```
glht(model, linfct, alternative = c("two.sided", "less", "greater"),
     rhs = 0, ...)
```

The two remaining arguments `alternative` and `rhs` define the direction of the alternative (see Section 3) and $\mathbf{m}$, respectively.

The matrix $\mathbf{K}$ can be described in three different ways:

- by a matrix with `length(coef(model))` columns, or
- by an expression or character vector giving a symbolic description of the linear functions of interest, or
- by an object of class *mcp* (for multiple comparison procedure).

The last alternative is convenient when contrasts of factor levels are to be compared and the model contrasts used to define the design matrix of the model have to be taken into account. The `mcp()` function takes the name of the factor to be tested as an argument as well as a character defining the type of comparisons as its value. For example, `mcp(treat = "Tukey")` sets up a matrix $\mathbf{K}$ for Tukey's all-pairwise comparisons among the levels of the factor `treat`, which has to appear on right hand side of the model formula of `model`. In this particular case, we need to assume that `model.frame()` and `model.matrix()` methods for `model` are available as well.

The `mcp()` function must be used with care when defining parameters of interest in two-way ANOVA or ANCOVA models. Here, the definition of treatment differences (such as Tukey's all-pair comparisons or Dunnett's comparison with a control) might be problem-specific. For example, in an ANCOVA model (here without intercept term)

$$Y_{ij} = \gamma_j + \beta_j X_i + \varepsilon_{ij}; \quad j = 1, \dots, q, i = 1, \dots, n_j$$

the parameters of interest might be $\gamma_j - \gamma_1 + \beta_j x - \beta_1 x$ for some value x of the continuous covariate X rather than the comparisons with a control $\gamma_j - \gamma_1$ that would be computed by `mcp()` with `"Dunnett"` option. The same problem occurs when interaction terms are present in a two-way ANOVA model, where the hypotheses might depend on the sample sizes. Because it is impossible to determine the parameters of interest automatically in this case, `mcp()` in **multcomp** will by default generate comparisons for the main effects γ_j only, ignoring covariates and interactions. Since version 1.1-2, one can specify to average over interaction terms and covariates using arguments `interaction_average = TRUE` and `covariate_average = TRUE` respectively, whereas versions older than 1.0-0 automatically averaged over interaction terms. We suggest to the users, however, that they write out,

manually, the set of contrasts they want. One should do this whenever there is doubt about what the default contrasts measure, which typically happens in models with higher order interaction terms. We refer to Hsu (1996), Chapter 7, and Searle (1971), Chapter 7.3, for further discussions and examples on this issue.

Objects of class *glht* returned by `glht()` include `coef()` and `vcov()` methods to compute $\hat{\vartheta}_n$ and $\mathsf{S}_n^\star$. Furthermore, a `summary()` method is available to perform different tests (max t, χ^2 and F-tests) and p-value adjustments, including those taking logical constraints into account (Shaffer, 1986; Westfall, 1997). In addition, the `confint()` method applied to objects of class *glht* returns simultaneous confidence intervals and allows for a graphical representation of the results. The numerical accuracy of adjusted p-values and simultaneous confidence intervals implemented in **multcomp** is continuously checked against results reported by Westfall et al. (1999).

6 Illustrations

6.1 Genetic Components of Alcoholism

Various studies have linked alcohol dependence phenotypes to chromosome 4. One candidate gene is *NACP* (non-amyloid component of plaques), coding for alpha synuclein. Bönsch et al. (2005) found longer alleles of *NACP*-REP1 in alcohol-dependent patients compared with healthy controls and report that the allele lengths show some association with levels of expressed alpha synuclein mRNA in alcohol-dependent subjects (see Figure 1). Allele length is measured as a sum score built from additive dinucleotide repeat length and categorized into three groups: short ($0 - 4$, $n = 24$), intermediate ($5 - 9$, $n = 58$), and long ($10 - 12$, $n = 15$). The data are available from package **coin**. Here, we are interested in comparing the distribution of the expression level of alpha synuclein mRNA in three groups of subjects defined by the allele length.

Thus, we fit a simple one-way ANOVA model to the data and define $\mathbf{K}$ such that $\mathbf{K}\theta$ contains all three group differences (Tukey's all-pairwise comparisons):

```
R> data("alpha", package = "coin")
R> amod <- aov(elevel ~ alength, data = alpha)
R> amod_glht <- glht(amod, linfct = mcp(alength = "Tukey"))
R> amod_glht$linfct
```

```
                          (Intercept) alengthintermediate alengthlong
intermediate - short                0                   1           0
long - short                        0                   0           1
long - intermediate                 0                  -1           1
attr(,"type")
[1] "Tukey"
```

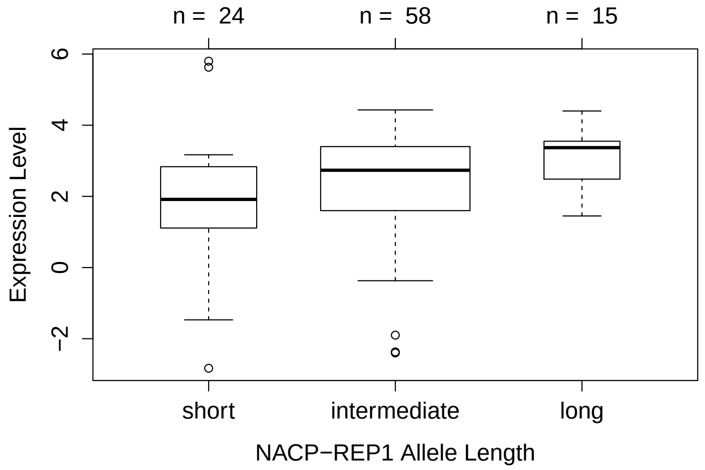

Figure 1: `alpha` data: Distribution of levels of expressed alpha synuclein mRNA in three groups defined by the *NACP*-REP1 allele lengths.

The `amod_glht` object now contains information about the estimated linear function $\hat{\vartheta}_n$ and their covariance matrix $\mathsf{S}_n^\star$ which can be inspected via the `coef()` and `vcov()` methods:

```
R> coef(amod_glht)
```

```
intermediate - short          long - short  long - intermediate
           0.4341523             1.1887500            0.7545977
```

```
R> vcov(amod_glht)
```

```
                     intermediate - short long - short long - intermediate
intermediate - short           0.14717604    0.1041001         -0.04307591
long - short                   0.10410012    0.2706603          0.16656020
long - intermediate           -0.04307591    0.1665602          0.20963611
```

The `summary()` and `confint()` methods can be used to compute a summary statistic including adjusted p-values and simultaneous confidence intervals, respectively:

```
R> confint(amod_glht)
```

```
         Simultaneous Confidence Intervals

Multiple Comparisons of Means: Tukey Contrasts


Fit: aov(formula = elevel ~ alength, data = alpha)

Quantile = 2.3718
95% family-wise confidence level
```

```
Linear Hypotheses:
                           Estimate lwr      upr
intermediate - short == 0  0.43415 -0.47574  1.34404
long - short == 0          1.18875 -0.04515  2.42265
long - intermediate == 0   0.75460 -0.33133  1.84053
```

```
R> summary(amod_glht)
```

```
         Simultaneous Tests for General Linear Hypotheses

Multiple Comparisons of Means: Tukey Contrasts


Fit: aov(formula = elevel ~ alength, data = alpha)

Linear Hypotheses:
                           Estimate Std. Error t value Pr(>|t|)
intermediate - short == 0    0.4342     0.3836   1.132   0.4924
long - short == 0            1.1888     0.5203   2.285   0.0615 .
long - intermediate == 0     0.7546     0.4579   1.648   0.2270
---
Signif. codes:  0 '***' 0.001 '**' 0.01 '*' 0.05 '.' 0.1 ' ' 1
(Adjusted p values reported -- single-step method)
```

Because of the variance heterogeneity that can be observed in Figure 1, one might be concerned with the validity of the above results stating that there is no difference between any combination of the three allele lengths. A sandwich estimator S_n might be more appropriate in this situation, and the `vcov` argument can be used to specify a function to compute some alternative covariance estimator S_n as follows:

```
R> amod_glht_sw <- glht(amod, linfct = mcp(alength = "Tukey"),
+                        vcov = sandwich)
R> summary(amod_glht_sw)
```

```
         Simultaneous Tests for General Linear Hypotheses

Multiple Comparisons of Means: Tukey Contrasts


Fit: aov(formula = elevel ~ alength, data = alpha)

Linear Hypotheses:
                           Estimate Std. Error t value Pr(>|t|)
intermediate - short == 0    0.4342     0.4239   1.024   0.5594
long - short == 0            1.1888     0.4432   2.682   0.0227 *
long - intermediate == 0     0.7546     0.3184   2.370   0.0502 .
---
Signif. codes:  0 '***' 0.001 '**' 0.01 '*' 0.05 '.' 0.1 ' ' 1
```

```
(Adjusted p values reported -- single-step method)
```

We used the `sandwich()` function from package **sandwich** (Zeileis, 2004, 2006) which provides us with a heteroscedasticity-consistent estimator of the covariance matrix. This result is more in line with previously published findings for this study obtained from non-parametric test procedures such as the Kruskal-Wallis test. A comparison of the simultaneous confidence intervals calculated based on the ordinary and sandwich estimator is given in Figure 2.

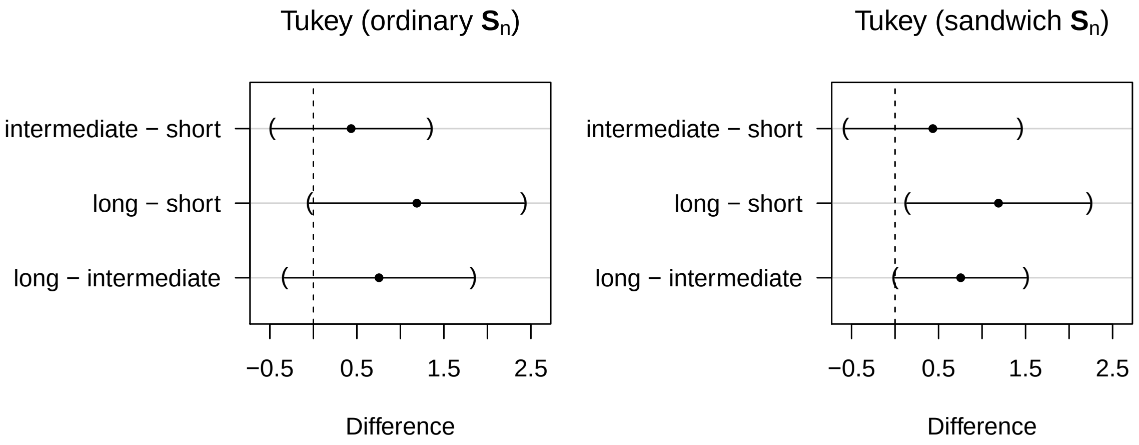

Figure 2: `alpha` data: Simultaneous confidence intervals based on the ordinary covariance matrix (left) and a sandwich estimator (right).

6.2 Prediction of Total Body Fat

Garcia et al. (2005) report on the development of predictive regression equations for body fat content by means of $p = 9$ common anthropometric measurements which were obtained for $n = 71$ healthy German women. In addition, the women's body composition was measured by Dual Energy X-Ray Absorptiometry (DXA). This reference method is very accurate in measuring body fat but finds little applicability in practical environments, mainly because of high costs and the methodological efforts needed. Therefore, a simple regression equation for predicting DXA measurements of body fat is of special interest for the practitioner. Backward-elimination was applied to select important variables from the available anthropometrical measurements and Garcia et al. (2005) report a final linear model utilizing hip circumference, knee breadth and a compound covariate which is defined as the sum of log chin skinfold, log triceps skinfold and log subscapular skinfold. Here, we fit the saturated model to the data and use the max-t test over all t-statistics to select important variables based on adjusted p-values. The linear model including all covariates and the classical unadjusted p-values are given by

```
R> data("bodyfat", package = "TH.data")
R> summary(lmod <- lm(DEXfat ~ ., data = bodyfat))
```

```
Call:
lm(formula = DEXfat ~ ., data = bodyfat)

Residuals:
   Min     1Q Median     3Q    Max
-6.954 -1.949 -0.219  1.169 10.812

Coefficients:
              Estimate Std. Error t value Pr(>|t|)
(Intercept)  -69.02828    7.51686  -9.183 4.18e-13 ***
age            0.01996    0.03221   0.620  0.53777
waistcirc      0.21049    0.06714   3.135  0.00264 **
hipcirc        0.34351    0.08037   4.274 6.85e-05 ***
elbowbreadth  -0.41237    1.02291  -0.403  0.68826
kneebreadth    1.75798    0.72495   2.425  0.01829 *
anthro3a       5.74230    5.20752   1.103  0.27449
anthro3b       9.86643    5.65786   1.744  0.08622 .
anthro3c       0.38743    2.08746   0.186  0.85338
anthro4       -6.57439    6.48918  -1.013  0.31500
---
Signif. codes:  0 '***' 0.001 '**' 0.01 '*' 0.05 '.' 0.1 ' ' 1

Residual standard error: 3.281 on 61 degrees of freedom
Multiple R-squared:  0.9231,        Adjusted R-squared:  0.9117
F-statistic: 81.35 on 9 and 61 DF,  p-value: < 2.2e-16
```

The marix of linear functions **K** is basically the identity matrix, except for the intercept which is omitted. Once the matrix **K** has been defined, it can be used to set up the general linear hypotheses:

```
R> K <- cbind(0, diag(length(coef(lmod)) - 1))
R> rownames(K) <- names(coef(lmod))[-1]
R> lmod_glht <- glht(lmod, linfct = K)
```

Classically, one would perform an F-test to check if any of the regression coefficients is non-zero:

```
R> summary(lmod_glht, test = Ftest())
```

```
         General Linear Hypotheses

Linear Hypotheses:
                  Estimate
age == 0           0.01996
waistcirc == 0     0.21049
hipcirc == 0       0.34351
elbowbreadth == 0 -0.41237
```

```
kneebreadth == 0   1.75798
anthro3a == 0      5.74230
anthro3b == 0      9.86643
anthro3c == 0      0.38743
anthro4 == 0      -6.57439

Global Test:
      F DF1 DF2     Pr(>F)
1 81.35   9  61 1.387e-30
```

but the source of the deviation from the global null hypothesis can only be inspected by the corresponding max-t test, i.e., via

```
R> summary(lmod_glht)

         Simultaneous Tests for General Linear Hypotheses

Fit: lm(formula = DEXfat ~ ., data = bodyfat)

Linear Hypotheses:
                  Estimate Std. Error t value Pr(>|t|)
age == 0           0.01996    0.03221   0.620   0.9959
waistcirc == 0     0.21049    0.06714   3.135   0.0213 *
hipcirc == 0       0.34351    0.08037   4.274   <0.001 ***
elbowbreadth == 0 -0.41237    1.02291  -0.403   0.9998
kneebreadth == 0   1.75798    0.72495   2.425   0.1312
anthro3a == 0      5.74230    5.20752   1.103   0.8948
anthro3b == 0      9.86643    5.65786   1.744   0.4776
anthro3c == 0      0.38743    2.08746   0.186   1.0000
anthro4 == 0      -6.57439    6.48918  -1.013   0.9298
---
Signif. codes:  0 '***' 0.001 '**' 0.01 '*' 0.05 '.' 0.1 ' ' 1
(Adjusted p values reported -- single-step method)
```

Only two covariates, waist and hip circumference, seem to be important and caused the rejection of H_0. Alternatively, an MM-estimator (Yohai, 1987) as implemented by `lmrob()` from package **lmrob** (Todorov et al., 2007) can be used to fit a robust version of the above linear model, the results coincide rather nicely (note that the control arguments to `lmrob()` were changed in **multcomp** version 1.2-6 and thus the results have slightly changed):

```
R> summary(glht(lmrob(DEXfat ~ ., data = bodyfat,
+              control = lmrob.control(setting = "KS2011")), linfct = K))

         Simultaneous Tests for General Linear Hypotheses

Fit: lmrob(formula = DEXfat ~ ., data = bodyfat, control = lmrob.control(setting = "KS20

Linear Hypotheses:
                  Estimate Std. Error z value Pr(>|z|)
age == 0           0.02402    0.02503   0.960    0.949
```

```
waistcirc == 0      0.23332    0.05252   4.443   <0.001 ***
hipcirc == 0        0.32704    0.06285   5.203   <0.001 ***
elbowbreadth == 0 -0.18370    0.80616  -0.228    1.000
kneebreadth == 0    0.93892    0.58126   1.615    0.565
anthro3a == 0       2.39744    4.07016   0.589    0.997
anthro3b == 0      10.43204    4.46917   2.334    0.144
anthro3c == 0       1.51427    1.62754   0.930    0.957
anthro4 == 0       -5.77756    5.11995  -1.128    0.887
---
Signif. codes:  0 '***' 0.001 '**' 0.01 '*' 0.05 '.' 0.1 ' ' 1
(Adjusted p values reported -- single-step method)
```

and the result reported above holds under very mild model assumptions.

6.3 Smoking and Alzheimer's Disease

Salib and Hillier (1997) report results of a case-control study on Alzheimer's disease and smoking behavior of 198 female and male Alzheimer patients and 164 controls. The `alzheimer` data have been re-constructed from Table 4 in Salib and Hillier (1997). The authors conclude that 'cigarette smoking is less frequent in men with Alzheimer's disease.' Originally, one was interested to assess whether there is any association between smoking and Alzheimer's (or other dementia) diseases. Here, we focus on how a potential association can be described (see Hothorn et al., 2006, for a non-parametric approach).

First, we fit a logistic regression model including both main effects and an interaction effect of smoking and gender. The response is a binary variable giving the diagnosis of the patient (either suffering from Alzheimer's disease or other dementias):

```
R> data("alzheimer", package = "coin")
R> y <- factor(alzheimer$disease == "Alzheimer",
+               labels = c("other", "Alzheimer"))
R> gmod <- glm(y ~ smoking * gender, data = alzheimer,
+               family = binomial())
R> summary(gmod)
```

```
Call:
glm(formula = y ~ smoking * gender, family = binomial(), data = alzheimer)

Deviance Residuals:
    Min       1Q   Median       3Q      Max
-1.6120  -1.0151  -0.7897   1.3141   2.0782

Coefficients:
                        Estimate Std. Error z value Pr(>|z|)
(Intercept)             -0.39442    0.13563  -2.908 0.003638 **
smoking<10               0.03774    0.51113   0.074 0.941140
```

```
smoking10-20           -0.61111    0.33084  -1.847 0.064725 .
smoking>20              0.54857    0.34867   1.573 0.115647
genderMale              0.07856    0.26039   0.302 0.762870
smoking<10:genderMale   1.25894    0.87692   1.436 0.151105
smoking10-20:genderMale -0.02855   0.50116  -0.057 0.954568
smoking>20:genderMale  -2.26959    0.59948  -3.786 0.000153 ***
---
Signif. codes:  0 '***' 0.001 '**' 0.01 '*' 0.05 '.' 0.1 ' ' 1

(Dispersion parameter for binomial family taken to be 1)

    Null deviance: 707.90  on 537  degrees of freedom
Residual deviance: 673.55  on 530  degrees of freedom
AIC: 689.55

Number of Fisher Scoring iterations: 4
```

The negative regression coefficient for heavy smoking males indicates that Alzheimer's disease might be less frequent in this group, but the model is still difficult to interpret based on the coefficients and corresponding p-values only. Therefore, confidence intervals on the probability scale for the different 'risk groups' are interesting and can be computed as follows. For each combination of gender and smoking behavior, the probability of suffering from Alzheimer's disease can be estimated by computing the logit function of the linear predictor from model `gmod`. Using the `predict()` method for generalized linear models is a convenient way to compute these probability estimates. Alternatively, we can set up $\mathbf{K}$ such that $\left(1 + \exp(-\hat{\vartheta}_n)\right)^{-1}$ is the vector of estimated probabilities with simultaneous confidence intervals

$$\left(\left(1 + \exp\left(-\left(\hat{\vartheta}_n - q_\alpha \mathbf{D}_n^{1/2}\right)\right)\right)^{-1}, \left(1 + \exp\left(-\left(\hat{\vartheta}_n + q_\alpha \mathbf{D}_n^{1/2}\right)\right)\right)^{-1}\right).$$

For our model, $\mathbf{K}$ is given by the following matrix (essentially the design matrix of `gmod` for eight persons with different smoking behavior from both genders)

```
R> K
```

```
             (Icpt) s<10 s10-20 s>20 gMale s<10:gMale s10-20:gMale s>20:gMale
None:Female       1    0      0    0     0          0            0          0
<10:Female        1    1      0    0     0          0            0          0
10-20:Female      1    0      1    0     0          0            0          0
>20:Female        1    0      0    1     0          0            0          0
None:Male         1    0      0    0     1          0            0          0
<10:Male          1    1      0    0     1          1            0          0
10-20:Male        1    0      1    0     1          0            1          0
>20:Male          1    0      0    1     1          0            0          1
```

and can easily be used to compute the confidence intervals described above

```
R> gmod_ci <- confint(glht(gmod, linfct = K))
R> gmod_ci$confint <- apply(gmod_ci$confint, 2, binomial()$linkinv)
R> plot(gmod_ci, xlab = "Probability of Developing Alzheimer",
+       xlim = c(0, 1))
```

The simultaneous confidence intervals are depicted in Figure 3. Using this representation of the results, it is obvious that Alzheimer's disease is less frequent in heavy smoking men compared to all other configurations of the two covariates.

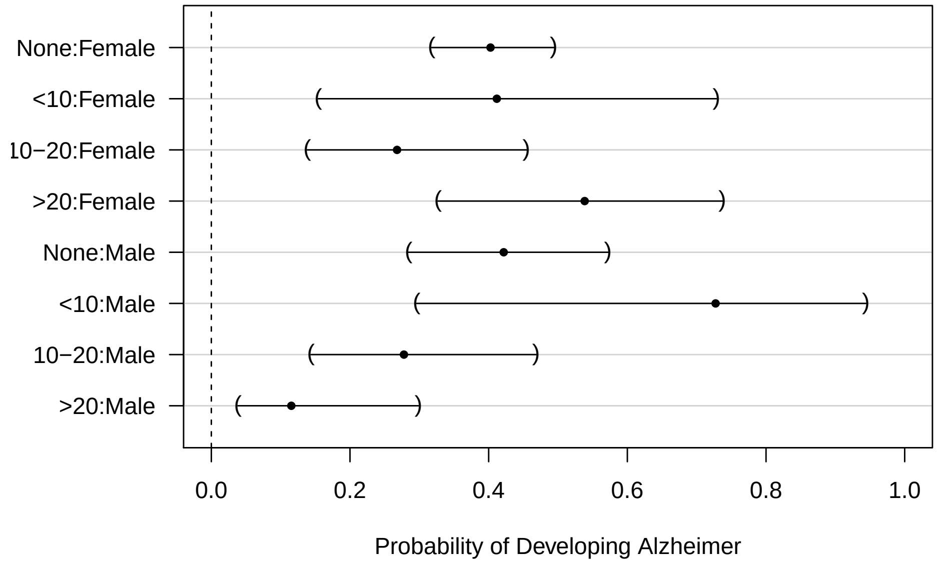

Figure 3: `alzheimer` data: Simultaneous confidence intervals for the probability to suffer from Alzheimer's disease.

6.4 Acute Myeloid Leukemia Survival

The treatment of patients suffering from acute myeloid leukemia (AML) is determined by a tumor classification scheme taking the status of various cytogenetic aberrations into account. Bullinger et al. (2004) investigate an extended tumor classification scheme incorporating molecular subgroups of the disease obtained by gene expression profiling. The analyses reported here are based on clinical data only (thus omitting available gene expression data) published online at `http://www.ncbi.nlm.nih.gov/geo`, accession number GSE425. The overall survival time and censoring indicator as well as the clinical variables age, sex, lactic dehydrogenase level (LDH), white blood cell count (WBC), and treatment group are taken from Supplementary Table 1 in Bullinger et al. (2004). In addition, this

table provides two molecular markers, the fms-like tyrosine kinase 3 (FLT3) and the mixed-lineage leukemia (MLL) gene, as well as cytogenetic information helpful to define a risk score ('low': karyotype t(8;21), t(15;17) and inv(16); 'intermediate': normal karyotype and t(9;11); and 'high': all other forms). One interesting question might be the usefulness of this risk score. Here, we fit a Weibull survival model to the data including all above mentioned covariates as well as their interactions with the patient's gender. Tukey's all-pairwise comparisons highlight that there seems to be a difference between 'high' scores and both 'low' and 'intermediate' ones but the latter two aren't distinguishable:

```
R> smod <- survreg(Surv(time, event) ~ Sex + Age + WBC + LDH + FLT3 + risk,
+                  data = clinical)
R> summary(glht(smod, linfct = mcp(risk = "Tukey")))

         Simultaneous Tests for General Linear Hypotheses

Multiple Comparisons of Means: Tukey Contrasts


Fit: survreg(formula = Surv(time, event) ~ Sex + Age + WBC + LDH +
    FLT3 + risk, data = clinical)

Linear Hypotheses:
                         Estimate Std. Error z value Pr(>|z|)
intermediate - high == 0   1.1101     0.3851   2.882  0.01119 *
low - high == 0            1.4769     0.4583   3.223  0.00359 **
low - intermediate == 0    0.3668     0.4303   0.852  0.66920
---
Signif. codes:  0 '***' 0.001 '**' 0.01 '*' 0.05 '.' 0.1 ' ' 1
(Adjusted p values reported -- single-step method)
```

Again, a sandwich estimator of the covariance matrix S_n can be plugged-in but the results stay very much the same in this case.

6.5 Forest Regeneration

In most parts of Germany, the natural or artificial regeneration of forests is difficult due to a high browsing intensity. Young trees suffer from browsing damage, mostly by roe and red deer. In order to estimate the browsing intensity for several tree species, the Bavarian State Ministry of Agriculture and Forestry conducts a survey every three years. Based on the estimated percentage of damaged trees, suggestions for the implementation or modification of deer management plans are made. The survey takes place in all 756 game management districts ('Hegegemeinschaften') in Bavaria. Here, we focus on the 2006 data of the game management district number 513 'Unterer Aischgrund' (located in Frankonia between Erlangen and Höchstadt). The data of 2700 trees include the species and a binary variable indicating whether or not the tree suffers from damage caused by deer browsing.

We fit a mixed logistic regression model (using package **lme4**, Bates, 2005, 2007) without intercept and with random effects accounting for the spatial variation of the trees. For each plot nested within a set of five plots orientated on a 100m transect (the location of the transect is determined by a pre-defined equally spaced lattice of the area under test), a random intercept is included in the model. We are interested in probability estimates and confidence intervals for each tree species. Each of the six fixed parameters of the model corresponds to one species, therefore, $\mathbf{K} = \text{diag}(6)$ is the linear function we are interested in:

```
R> mmod <- lmer(damage ~ species - 1 + (1 | lattice / plot),
+                data = trees513, family = binomial())
R> K <- diag(length(fixef(mmod)))
```

Based on $\mathbf{K}$, we first compute simultaneous confidence intervals for $\mathbf{K}\theta$ and transform these into probabilities:

```
R> ci <- confint(glht(mmod, linfct = K))
R> ci$confint <- 1 - binomial()$linkinv(ci$confint)
R> ci$confint[,2:3] <- ci$confint[,3:2]
```

The result is shown in Figure 4. Browsing is less frequent in hardwood but especially small oak trees are severely at risk. Consequently, the local authorities increased the number of roe deers to be harvested in the following years. The large confidence interval for ash, maple, elm and lime trees is caused by the small sample size.

7 Conclusion

Multiple comparisons in linear models have been in use for a long time, see Hochberg and Tamhane (1987), Hsu (1996), and Bretz et al. (2008). In this paper we have extended the theory to a broader class of parametric and semi-parametric statistical models, which allows for a unified treatment of multiple comparisons and other simultaneous inference procedures in generalized linear models, mixed models, models for censored data, robust models, etc. In essence, all that is required is a parameter estimate $\hat{\theta}_n$ following an asymptotic multivariate normal distribution, and a consistent estimate of its covariance matrix. Standard software packages can be used to compute these quantities. As shown in this paper, these quantities are sufficient to derive powerful simultaneous inference procedures, which are tailored to the experimental questions under investigation. Therefore, honest decisions based on simultaneous inference procedures maintaining a pre-specified familywise error rate (at least asymptotically) can now be based on almost all classical and modern statistical models.

The examples given in Section 6 illustrate two facts. At first, the presented approach helps to formulate simultaneous inference procedures in situations that were previously hard to

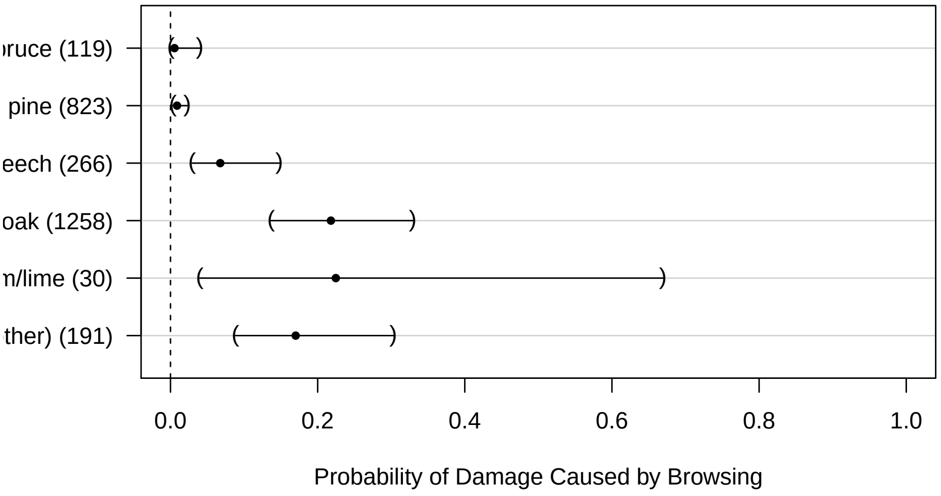

Figure 4: `trees513` data: Probability of damage caused by roe deer browsing for six tree species. Sample sizes are given in brackets.

deal with and, at second, a flexible open-source implementation offers tools to actually perform such procedures rather easily. With the **multcomp** package, freely available from http://CRAN.R-project.org, honest simultaneous inference is only two simple R commands away. The analyses shown in Section 6 are reproducible via the **multcomp** package vignette "`generalsiminf`".

References

Douglas Bates. Fitting linear mixed models in R. *R News*, 5(1):27–30, May 2005. URL http://CRAN.R-project.org/doc/Rnews/.

Douglas Bates. *lme4: Linear mixed-effects models using S4 classes*, 2007. URL http://CRAN.R-project.org. R package version 0.99875-9.

Domenikus Bönsch, Thomas Lederer, Udo Reulbach, Torsten Hothorn, Johannes Kornhuber, and Stefan Bleich. Joint analysis of the NACP-REP1 marker within the alpha synuclein gene concludes association with alcohol dependence. *Human Molecular Genetics*, 14(7):967–971, 2005.

Frank Bretz, Alan Genz, and Ludwig A. Hothorn. On the numerical availability of multiple comparison procedures. *Biometrical Journal*, 43(5):645–656, 2001.

Frank Bretz, Torsten Hothorn, and Peter Westfall. Multiple comparison procedures in linear models. In *International Conference on Computational Statistics*, 2008. submitted.

Lars Bullinger, Konstanze Döhner, Eric Bair, Stefan Fröhlich, Richard F. Schlenk, Robert Tibshirani, Hartmut Döhner, and Jonathan R. Pollack. Use of gene-expression profiling to identify prognostic subclasses in adult acute myloid leukemia. *New England Journal of Medicine*, 350(16):1605–1616, 2004.

Ada L. Garcia, Karen Wagner, Torsten Hothorn, Corinna Koebnick, Hans-Joachim F. Zunft, and Ulrike Trippo. Improved prediction of body fat by measuring skinfold thickness, circumferences, and bone breadths. *Obesity Research*, 13(3):626–634, 2005.

Alan Genz. Numerical computation of multivariate normal probabilities. *Journal of Computational and Graphical Statistics*, 1:141–149, 1992.

Alan Genz and Frank Bretz. Numerical computation of multivariate t-probabilities with application to power calculation of multiple contrasts. *Journal of Statistical Computation and Simulation*, 63:361–378, 1999.

Alan Genz and Frank Bretz. Methods for the computation of multivariate t-probabilities. *Journal of Computational and Graphical Statistics*, 11:950–971, 2002.

Yosef Hochberg and Ajit C. Título Tamhane. *Multiple Comparison Procedures*. John Wiley & Sons, New York, 1987.

Torsten Hothorn, Kurt Hornik, Mark A. van de Wiel, and Achim Zeileis. A Lego system for conditional inference. *The American Statistician*, 60(3):257–263, 2006.

Torsten Hothorn, Frank Bretz, Peter Westfall, and Richard M. Heiberger. *multcomp: Simultaneous Inference in General Parametric Models*, 2008. URL http://CRAN.R-project.org. R package version 1.0-0.

Jason C. Hsu. *Multiple Comparisons: Theory and Methods*. CRC Press, Chapman & Hall, London, 1996.

Ruth Marcus, Peritz Eric, and K. Ruben Gabriel. On closed testing procedures with special reference to ordered analysis of variance. *Biometrika*, 63:655–660, 1976.

R Development Core Team. *R: A Language and Environment for Statistical Computing*. R Foundation for Statistical Computing, Vienna, Austria, 2008. URL http://www.R-project.org. ISBN 3-900051-07-0.

Peter J. Rousseeuw and Annick M. Leroy. *Robust Regression and Outlier Detection*. John Wiley & Sons, New York, 2nd edition, 2003.

Emad Salib and Valerie Hillier. A case-control study of smoking and Alzheimer's disease. *International Journal of Geriatric Psychiatry*, 12:295–300, 1997.

Shayle R. Searle. *Linear Models*. John Wiley & Sons, New York, 1971.

Robert J. Serfling. *Approximation Theorems of Mathematical Statistics*. John Wiley & Sons, New York, 1980.

Juliet P. Shaffer. Modified sequentially rejective multiple test procedures. *Journal of the American Statistical Association*, 81:826–831, 1986.

Leonard A. Stefanski and Dennis D. Boos. The calculus of M-estimation. *The American Statistician*, 56:29–38, 2002.

Valentin Todorov, Andreas Ruckstuhl, Matias Salibian-Barrera, Martin Maechler, and others. *robustbase: Basic Robust Statistics*, 2007. URL http://CRAN.R-project.org. R package version 0.2-8.

Yung Liang Tong. *The Multivariate Normal Distribution*. Springer-Verlag, New York, Berlin, 1990.

Peter H. Westfall. Multiple testing of general contrasts using logical constraints and correlations. *Journal of the American Statistical Association*, 92(437):299–306, 1997.

Peter H. Westfall and Randall D. Tobias. Multiple testing of general contrasts: Truncated closure and the extended Shaffer-Royen method. *Journal of the American Statistical Association*, 102:487–494, 2007.

Peter H. Westfall, Randall D. Tobias, Dror Rom, Russell D. Wolfinger, and Yosef Hochberg. *Multiple Comparisons and Multiple Tests Using the SAS System*. SAS Institute Inc., Cary, NC, 1999.

Victor J. Yohai. High breakdown-point and high efficiency estimates for regression. *The Annals of Statistics*, 15:642–65, 1987.

Achim Zeileis. Econometric computing with HC and HAC covariance matrix estimators. *Journal of Statistical Software*, 11(10):1–17, 2004. URL http://www.jstatsoft.org/v11/i10/.

Achim Zeileis. Object-oriented computation of sandwich estimators. *Journal of Statistical Software*, 16(9):1–16, 2006. URL http://www.jstatsoft.org/v16/i09/.
