# Supplementary material for: Comparing Kaolin and Pinolene to Improve Sustainable Grapevine Production during Drought
Source: PLoS One. 2016 Jun 13;11(6):e0156631. doi: 10.1371/journal.pone.0156631 (PMC4905681; doi:10.1371/journal.pone.0156631)
Supplement: S1 File — (GZ) [file pone.0156631.s003.gz › PONE_Brillante_et_al_2016_Code_and_Data/packrat/src/multcomp/multcomp_1.4-0.tar.gz!/multcomp/inst/doc/chfls1.pdf]

Supplementary Material for *A re-evaluation of the model selection procedure in Pollet & Nettle (2009)*

Esther Herberich, Torsten Hothorn, Daniel Nettle & Thomas Pollet

Summary

In this paper, we first explain the statistical model underlying the ordinal regression technique used by Pollet and Nettle (2009), including the two possible ways of calculating the likelihood function (section 1). We then show that the model fit criteria reported were in fact invalid, and calculate the correct ones, showing that this leads to a different choice of best model (section 2). We then suggest two other strategies of model selection for these data, and show that these also lead to different best-fitting models than that reported by Pollet and Nettle (2009) (sections 3 and 4).

1 Ordinal regression: The cumulative Logit Model

The appropriate model for a dependent variable $Y_i \in \{1, \ldots, R\}$, $i = 1, \ldots, n$, consisting of ranked outcome categories is a cumulative logit model (Agresti, 2002):

$$P(Y_i \leq r|x_i) = \frac{\exp(\beta_{0r} - x_i^\top \beta)}{1 + \exp(\beta_{0r} - x_i^\top \beta)}, \quad r = 1, \ldots, R-1.$$

The model includes intercepts β_{0r} for each category and a global parameter vector $\beta = (\beta_1, \ldots, \beta_p)$ for the p covariates.
To obtain parameter estimates the maximum-likelihood method is used. The responses are conditionally independent and follow a multinomial distribution with

$$\begin{aligned} y_i|x_i &\sim \mathcal{M}(1, \pi_i), \\ y_i &= (y_{i1}, \ldots, y_{iR-1}) = (0, \ldots, 0, \underbrace{1}_{r-\text{th position}}, 0, \ldots, 0) \quad \Leftrightarrow \quad Y_i = r, \\ \pi_i &= (\pi_{i1}, \ldots, \pi_{iR-1}) \quad \text{with} \\ \pi_{ir} &= P(Y_i = r|x_i) = P(Y_i \leq r|x_i) - P(Y_i \leq r-1|x_i), \; r = 1, \ldots, R-1. \end{aligned}$$

The associated likelihood function is

$$\mathcal{L}(\beta_{01}, \ldots \beta_{0R-1}, \beta; x_1, \ldots x_n) = \prod_{i=1}^{n} {\pi_{i1}}^{y_{i1}} \cdot {\pi_{i2}}^{y_{i2}} \cdot \ldots \cdot (1 - \pi_{i1} - \ldots - \pi_{iR-1})^{1 - y_{i1} - \ldots - y_{iR-1}}.$$

To obtain the parameter estimates, the data are often (as by default in SPSS 15.0) pooled in K groups, and the likelihood of the grouped data is maximized, instead of the likelihood of the individual data. Group k, $k = 1, \dots K$, includes all h_k observations with the value $\tilde{x}_k = (\tilde{x}_{k1}, \dots, \tilde{x}_{kp})$ of the covariates $x = (x_1, \dots, x_p)$. The responses again follow a multinomial distribution:

$$\begin{aligned} \tilde{y}_k | \tilde{x}_k &\sim \mathcal{M}(h_k, \tilde{\pi}_k), \\ \tilde{y}_k &= (\tilde{y}_{k1}, \dots, \tilde{y}_{kR-1}), \\ \tilde{\pi}_k &= (\tilde{\pi}_{k1}, \dots, \tilde{\pi}_{kR-1}). \end{aligned}$$

The vector $\tilde{y}_k$ contains the observed frequencies of the categories 1 to $R-1$ in group k. $\tilde{\pi}_{kr}$ is the probability of an individual of group k being in category r. The likelihood function of the grouped data results in

$$\mathcal{L}(\beta_{01}, \dots \beta_{0R-1}, \beta; \tilde{x}_1, \dots \tilde{x}_K) =$$
$$\underbrace{\prod_{k=1}^{K} \frac{h_k!}{\tilde{y}_{k1}! \cdot \ldots \cdot \tilde{y}_{kR-1}}}_{\text{multinomial constant}} \cdot \underbrace{\prod_{k=1}^{K} \tilde{\pi}_{k1}{}^{\tilde{y}_{k1}} \cdot \tilde{\pi}_{k2}{}^{\tilde{y}_{k2}} \cdot \ldots \cdot (1 - \tilde{\pi}_{k1} - \ldots - \tilde{\pi}_{kR-1})^{1 - \tilde{y}_{k1} \cdots - \tilde{y}_{kR-1}}}_{\text{kernel}}.$$

The kernel of the likelihood function of the grouped data equals the likelihood function of the individual data. Both likelihood functions only differ by the multinomial constant in the likelihood for grouped data. Maximization of both likelihood functions results in the same parameter estimates.

2 Variable Selection according to Pollet and Nettle

The analytical strategy of Pollet and Nettle (2009) was as follows:
Start: Inclusion of partner income and partner height as independent variables.
Step 1: Omission of any independent variable not significant in the start model. Significance is assessed by the Wald test without adjusting for multiplicity.
Subsequent steps: Stepwise inclusion of the remaining variables in the order in which they improve model fit the most compared to the start model. The procedure stops, when model fit cannot be improved further by including another covariate.

Model fit was assessed by the criteria AIC and BIC:

$$\begin{aligned} \text{AIC} &= -2 \cdot \ell(\hat{\theta}) + 2 \cdot \dim(\theta), \\ \text{BIC} &= -2 \cdot \ell(\hat{\theta}) + \log(n) \cdot \dim(\theta). \end{aligned}$$

ℓ denotes the logarithmized likelihood function. In the cumulative logit model the parameter vector θ is $\theta = (\beta_{01}, \dots, \beta_{0R-1}, \beta_1, \dots, \beta_p)$.

In SPSS 15.0, the likelihood function for multinomial distributed responses is calculated by pooling the data according to the covariates (see above). Parameter estimates are the same whether they are obtained by maximization of the likelihood function for individual or grouped data. To compare several models, which differ in terms of their

	Start	Step 1	Step 2
Partner income	$\surd$	$\surd$	$\surd$
Partner height	$\surd$[1]	—	—
Happiness	—	—	$\surd$
Calculations by Pollet and Nettle (2009):			
$-2 \cdot \ell(\hat{\theta})$	1868.1	405.6	752.4
$\dim(\theta)$	2	1	4
AIC	1872.1	407.6	760.4[2]
BIC	1882.8	412.9	781.7[2]
Correct calculations:			
$-2 \cdot \ell(\hat{\theta})$	3903.8	3906.7	3880.5
$\dim(\theta)$	6	5	8
AIC	3915.8	3916.7	3896.5
BIC	3947.8	3943.4	3939.2

[1] Coefficient of this variable not significant based on Wald test.
[2] No reduction of AIC and BIC by adding a further variable

Table 1: Summary of variable selection in Pollet and Nettle (2009).

covariates, by the (log) likelihood function or by criteria calculated by the (log) likelihood function (like AIC and BIC), the multinomial constant has to be omitted. As grouping differs among the models due to different covariates in the models, the multinomial constant differs as well and the models cannot be compared by the likelihood which includes the constant.

As SPSS 15.0 provides only $-2 \cdot \ell(\hat{\theta})$, Pollet and Nettle (2009) calculated AIC and BIC by adding the penalization terms $2 \cdot \dim(\theta)$ and $\log(n) \cdot \dim(\theta)$ respectively to -2 log likelihood of the grouped data including the multinomial constant, leading to an invalid model choice.

Table 1 shows the progress of model choice following the strategy of Pollet and Nettle (2009). The invalid model fit criteria used in the paper, as well as the correctly calculated criteria, are shown. The number of model parameters differs, because Pollet and Nettle did not account for the category specific intercepts $\beta_{01}, \ldots, \beta_{0R-1}$.

Start model and step 1 are the same as in table 1. In the subsequent models further variables were added one at a time starting with the variable which improved model fit the most. The selected variables were the same using AIC and BIC to assess model fit except for step 4a/4b. Using BIC the model in step 5 was chosen as the best model including the variables partner income, education, age, happiness and difference in education. Using AIC as model fit criterion, inclusion of region and health could further improve model fit. The start model included partner income and partner height. The variable partner income was significant based on the Wald test and remained in the model while the variable partner height was excluded from the model due to non-significance. In step 2 inclusion of the variable self-reported happiness resulted in the best improvement of model fit compared to the start model. Inclusion of fur-

ther variables did not improve model fit. Therefore the model with partner income and happiness was chosen as the best model with partner income being the only significant variable based on the Wald test.

When using the correctly calculated criteria AIC and BIC, a different model is chosen. In step 2 the variable education instead of happiness is included. The progress of variable selection following to the analytical strategy of Pollet and Nettle (2009) using the correctly calculated criteria, is shown in table 2. Start model and step 1 are the same as in table 1. In the subsequent models further variables were added one at a time starting with the variable which improved model fit the most. The selected variables were the same using AIC and BIC to assess model fit except for step 4a/4b. Using BIC the model in step 5 was chosen as the best model including the variables partner income, education, age, happiness and difference in education. Using AIC as model fit criterion, inclusion of region and health could further improve model fit. In the next section a further method of variable selection based on the AIC is used to determine the important factors for orgasm frequency.

	Start	Step 1	Step 2	Step 3	Step 4a	Step 4b	Step 5	Step 6	Step 7
Partner income	√	√	√	√	√	√	√	√	√
Partner height	√	—	—	—	—	—	—	—	—
Education ♀	—	—	√	√	√	√	√	√	√
Age ♀	—	—	—	√	√	√	√	√	√
Happiness ♀	—	—	—	—	√	—	√	√	√
Difference in Education	—	—	—	—	—	√	√	√	√
Region	—	—	—	—	—	—	—	√	√
Health ♀	—	—	—	—	—	—	—	—	√
AIC	3915.8	3916.7	3837.0	3800.0	3779.4[1]		3764.3	3759.2	3753.9[4]
BIC	3947.8	3943.4	3890.4	3858.7		3848.7[2]	3844.3[3]		

[1] AIC for step 4a.
[2] BIC for step 4b.
[3] No reduction of BIC by adding a further variable.
[4] No reduction of AIC by adding a further variable.

Table 2: Summary of variable selection following the strategy of Pollet and Nettle (2009) using the correctly calculated AIC and BIC.

3 Stepwise Backward Selection

```
   Mode   FALSE    TRUE    NA's
logical       3    1531       0
```

The stepwise backward selection starts with the saturated model, which includes all variables. Variables are omitted one at a time starting with the variable that reduces the AIC most. Variable selection stops, when the AIC cannot be reduced further by removing a variable. Note that the original data contains three missing values in variable `edudiffSD`. The corresponding observations have been removed from the data set before fitting all models presented in Table 3 but only for models involving these variable presented in Table 2 (since we assume the same approach was taken in SPSS).

In our data a stepwise backward selection results in a reduction of the AIC from 3759.2 in the saturated model to 3752.7 in the reduced model. The steps of the backwise selection are shown in table 3. The variable partner income, which was included in all models when following the strategy of Pollet and Nettle (2009), is here dropped in step 2. By stepwise backward selection the same variables except for partner income are chosen as by the strategy of Pollet and Nettle using the correctly calculated AIC.

Model	Start	Step 1	Step 2	Step 3	Step 4
Partner height	√	—	—	—	—
Partner income	√	√	—	—	—
Duration of relationship	√	√	√	—	—
Difference in income	√	√	√	√	—
Age ♀	√	√	√	√	√
Diffference in education	√	√	√	√	√
Education ♀	√	√	√	√	√
Happiness ♀	√	√	√	√	√
Region	√	√	√	√	√
Health ♀	√	√	√	√	√
AIC	3759.2	3757.2	3755.3	3753.8	3752.7

Table 3: Steps of backward variable selection based on the AIC.

4 Variable Selection by Simultaneous Inference

In the following, the relevant factors for orgasm frequency are assessed using the procedure for simultaneous inference introduced by Hothorn *et al.* (2008) instead of using model fit criterions like AIC and BIC. Therefore, we fit a cumulative logit model, which includes all covariates and use the max-t-test to select important variables based on adjusted p-values. The hypotheses are

$$H_j^0 : \beta_j = 0,\ j = 1, \ldots, p,$$

and can be specified as linear hypotheses $K\beta = 0$ with the matrix K being the $p \times p$ identity matrix. Three observations with missings in variable `edudiffSD` have been removed prior to fitting the model.

The parameter estimates and associated adjusted p-values are shown in table 4. The respondant's education is the relevant factor for orgasm frequency with a cumulative odds ratio of $\exp(-1.82) = 0.16$ comparing the categories "No school" and "University". Women with university degree have a higher chance of having an orgasm more frequently than women without school education. Associated with this is the significance of the variable "difference in education" with women having less orgasms the higher their partners' level of education is above their own. Further differences in orgasm frequency exist between two regions of China.

Not only when selecting important variables by simultaneous inference of all parameter estimates the respondent's education was chosen as the relevant factor for orgasm frequency, but also the methods described in sections 2 and 3 selected education as an important variable among others. Therefore we further investigate the effect of education and take a look at the cumulative odds ratios when comparing the levels of the respondent's education. Again we fit a cumulative logit model including all covariates. The matrix of linear functions K, which sets up the linear hypothesis of model parameters, is defined in the form that consecutive levels of education are compared. The estimated log odds ratios and associated p-values of the simultaneous comparisons based on the max-t-test are summarized in table 5.

When comparing levels of education from "No school" to "Upper middle school" women with the respective higher level of education tend to have more frequent orgasms with cumulative odds ratios of 2.32 (Comparison Primary school - No school), 1.70 (Comparison Lower middle school - Primary school) und 1.81 (Comparison Upper middle school - Lower middle school).

References

Agresti A (2002). *Categorical Data Analysis.* 2nd edition. John Wiley and Sons, New York.

Hothorn T, Bretz F, Westfall P (2008). "Simultaneous Inference in General Parametric Models." *Biometrical Journal*, **50**(3), 346–363.

Pollet TV, Nettle D (2009). "Partner Wealth Predicts Self-Reported Orgasm Frequency in a Sample of Chinese Women." *Evolution and Human Behavior*, **30**, 146–151.

Variable	Estimate	Adjusted p-value
Partner income	0.02	1.000
Partner height	0.01	1.000
Duration of relationship	0.09	1.000
Age	-0.37	0.091
Difference in education	-0.17	0.030
Difference in income	-0.03	1.000
Education		
University (reference category)	NA	—
Junior college	0.11	1.000
Upper middle	0.14	1.000
Lower middle	-0.45	0.909
Primary	-0.98	0.078
No school	-1.82	0.000
Health		
Poor (reference category)	NA	—
Not good	1.22	0.526
Fair	1.56	0.165
Good	1.70	0.091
Excellent	1.72	0.089
Happiness		
Very unhappy (reference category)	NA	—
Not too happy	0.17	1.000
Relatively happy	0.64	0.986
Very happy	0.91	0.848
Region		
Central West (reference category)	NA	—
North East	0.40	0.315
North	0.20	0.989
Inland South	0.49	0.224
Coastal East	0.20	0.980
Coastal South	0.59	0.015

Table 4: Parameter estimates of the saturated cumulative logit model with associated adjusted p-values of the max-t-test.

Compared levels of education	Estimated log odds ratio	Adjusted p-value
University - Junior college	-0.11	0.999
Junior college - Upper middle	-0.03	1.000
Upper middle - Lower middle	0.59	0.000
Lower middle - Primary	0.53	0.003
Primary - No school	0.84	0.003

Table 5: Estimated log odds ratios for comparisons of consecutive levels of education and associated adjusted p-values of the simultaneous comparisons.
