# Supplementary material for: Comparing Kaolin and Pinolene to Improve Sustainable Grapevine Production during Drought
Source: PLoS One. 2016 Jun 13;11(6):e0156631. doi: 10.1371/journal.pone.0156631 (PMC4905681; doi:10.1371/journal.pone.0156631)
Supplement: S1 File — (GZ) [file pone.0156631.s003.gz › PONE_Brillante_et_al_2016_Code_and_Data/packrat/src/multcomp/multcomp_1.4-0.tar.gz!/multcomp/inst/doc/multcomp-examples.pdf]

Additional **multcomp** Examples

Torsten Hothorn

March 5, 2015

It is assumed that the reader is familiar with the theory and applications described in the `generalsiminf` vignette.

1 Simple Examples

Example: Simple Linear Model. Consider a simple univariate linear model regressing the distance to stop on speed for 50 cars:

```
R> lm.cars <- lm(dist ~ speed, data = cars)
R> summary(lm.cars)
```

```
Call:
lm(formula = dist ~ speed, data = cars)

Residuals:
   Min     1Q Median     3Q    Max
-29.07  -9.53  -2.27   9.21  43.20

Coefficients:
            Estimate Std. Error t value Pr(>|t|)
(Intercept)  -17.579      6.758   -2.60    0.012 *
speed          3.932      0.416    9.46  1.5e-12 ***
---
Signif. codes:  0 '***' 0.001 '**' 0.01 '*' 0.05 '.' 0.1 ' ' 1

Residual standard error: 15.4 on 48 degrees of freedom
Multiple R-squared:  0.651,        Adjusted R-squared:  0.644
F-statistic: 89.6 on 1 and 48 DF,  p-value: 1.49e-12
```

The estimates of the regression coefficients β and their covariance matrix can be extracted from the fitted model via:

```
R> betahat <- coef(lm.cars)
R> Vbetahat <- vcov(lm.cars)
```

At first, we are interested in the hypothesis $\beta_1 = 0$ and $\beta_2 = 0$. This is equivalent to the linear hypothesis $\mathbf{K}\beta = 0$ where $\mathbf{K} = \text{diag}(2)$, i.e.,

```
R> K <- diag(2)
R> Sigma <- diag(1 / sqrt(diag(K %*% Vbetahat %*% t(K))))
R> z <- Sigma %*% K %*% betahat
R> Cor <- Sigma %*% (K %*% Vbetahat %*% t(K)) %*% t(Sigma)
```

Note that $\mathbf{z} = (-2.6011, 9.464)$ is equal to the t statistics. The multiplicity-adjusted p values can now be computed by means of the multivariate t distribution utilizing the `pmvt` function available in package **mvtnorm**:

```
R> library("mvtnorm")
R> df.cars <- nrow(cars) - length(betahat)
R> sapply(abs(z), function(x) 1 - pmvt(-rep(x, 2), rep(x, 2), corr = Cor, df = df.cars))

[1] 1.661e-02 2.458e-12
```

Note that the p value of the global test is the minimum p value of the partial tests.

The computations above can be performed much more conveniently using the functionality implemented in package **multcomp**. The function `glht` just takes a fitted model and a matrix defining the linear functions, and thus hypotheses, to be tested:

```
R> library("multcomp")
R> cars.ht <- glht(lm.cars, linfct = K)
R> summary(cars.ht)

         Simultaneous Tests for General Linear Hypotheses

Fit: lm(formula = dist ~ speed, data = cars)

Linear Hypotheses:
                 Estimate Std. Error t value Pr(>|t|)
(Intercept) == 0  -17.579      6.758   -2.60    0.017 *
speed == 0          3.932      0.416    9.46   <1e-10 ***
---
Signif. codes:  0 ‘***’ 0.001 ‘**’ 0.01 ‘*’ 0.05 ‘.’ 0.1 ‘ ’ 1
(Adjusted p values reported -- single-step method)
```

Simultaneous confidence intervals corresponding to this multiple testing procedure are available via

```
R> confint(cars.ht)

         Simultaneous Confidence Intervals

Fit: lm(formula = dist ~ speed, data = cars)

Quantile = 2.13
```

```
95% family-wise confidence level


Linear Hypotheses:
                 Estimate lwr     upr
(Intercept) == 0 -17.579  -31.977  -3.181
speed == 0         3.932    3.047   4.818
```

The application of the framework isn't limited to linear models, nonlinear least-squares estimates can be tested as well. Consider constructing simultaneous confidence intervals for the model parameters (example from the manual page of `nls`):

```
R> DNase1 <- subset(DNase, Run == 1)
R> fm1DNase1 <- nls(density ~ SSlogis(log(conc), Asym, xmid, scal), DNase1)
R> K <- diag(3)
R> rownames(K) <- names(coef(fm1DNase1))
R> confint(glht(fm1DNase1, linfct = K))

         Simultaneous Confidence Intervals

Fit: nls(formula = density ~ SSlogis(log(conc), Asym, xmid, scal),
    data = DNase1, algorithm = "default", control = list(maxiter = 50,
        tol = 1e-05, minFactor = 0.0009765625, printEval = FALSE,
        warnOnly = FALSE), trace = FALSE)

Quantile = 2.138
95% family-wise confidence level


Linear Hypotheses:
          Estimate lwr   upr
Asym == 0 2.345    2.178 2.512
xmid == 0 1.483    1.309 1.657
scal == 0 1.041    0.972 1.110
```

which is not totally different from univariate confidence intervals

```
R> confint(fm1DNase1)

       2.5% 97.5%
Asym 2.1935 2.539
xmid 1.3215 1.679
scal 0.9743 1.115
```

because the parameter estimates are highly correlated

```
R> cov2cor(vcov(fm1DNase1))
```

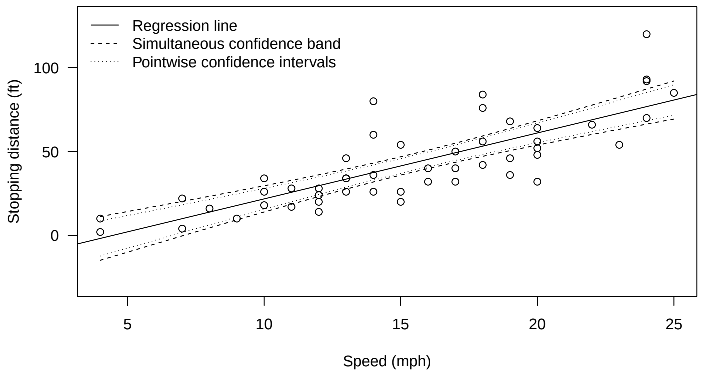

Figure 1: cars data: Regression line with confidence bands (dashed) and intervals (dotted).

```
       Asym   xmid   scal
Asym 1.0000 0.9868 0.9008
xmid 0.9868 1.0000 0.9063
scal 0.9008 0.9063 1.0000
```

Example: Confidence Bands for Regression Line. Suppose we want to plot the linear model fit to the `cars` data including an assessment of the variability of the model fit. This can be based on simultaneous confidence intervals for the regression line $x_i^\top \hat{\beta}$:

```
R> K <- model.matrix(lm.cars)[!duplicated(cars$speed),]
R> ci.cars <- confint(glht(lm.cars, linfct = K), abseps = 0.1)
```

Figure 1 depicts the regression fit together with the confidence band for the regression line and the pointwise confidence intervals as computed by `predict(lm.cars)`.

2 Multiple Comparison Procedures

Multiple comparisons of means, i.e., regression coefficients for groups in AN(C)OVA models, are a special case of the general framework sketched in the previous section. The main difficulty is that the comparisons one is usually interested in, for example all-pairwise differences, can't be directly specified based on model parameters of an AN(C)OVA regression model. We start with a simple one-way ANOVA example and generalize to ANCOVA models in the following.

Consider a one-way ANOVA model, i.e., the only covariate x is a factor at j levels. In the absence of an intercept term only, the elements of the parameter vector $\beta \in \mathbb{R}^j$ correspond to the mean of the response in each of the j groups:

```
R> ex <- data.frame(y = rnorm(12), x = gl(3, 4, labels = LETTERS[1:3]))
R> aov.ex <- aov(y ~ x - 1, data = ex)
R> coef(aov.ex)

     xA      xB      xC 
-0.2784  0.1496  0.5301 
```

Thus, the hypotheses $\beta_2 - \beta_1 = 0$ and $\beta_3 - \beta_1 = 0$ can be written in form of a linear function $\mathbf{K}\beta$ with

```
R> K <- rbind(c(-1, 1, 0),
+            c(-1, 0, 1))
R> rownames(K) <- c("B - A", "C - A")
R> colnames(K) <- names(coef(aov.ex))
R> K

      xA xB xC
B - A -1  1  0
C - A -1  0  1
```

Using the general linear hypothesis function **glht**, this so-called 'many-to-one comparison procedure' [Dunnett, 1955] can be performed via

```
R> summary(glht(aov.ex, linfct = K))

         Simultaneous Tests for General Linear Hypotheses

Fit: aov(formula = y ~ x - 1, data = ex)

Linear Hypotheses:
           Estimate Std. Error t value Pr(>|t|)
B - A == 0    0.428      0.653    0.66     0.74
C - A == 0    0.808      0.653    1.24     0.39
(Adjusted p values reported -- single-step method)
```

Alternatively, a symbolic description of the general linear hypothesis of interest can be supplied to **glht**:

```
R> summary(glht(aov.ex, linfct = c("xB - xA = 0", "xC - xA = 0")))

         Simultaneous Tests for General Linear Hypotheses

Fit: aov(formula = y ~ x - 1, data = ex)

Linear Hypotheses:
```

```
             Estimate Std. Error t value Pr(>|t|)
xB - xA == 0    0.428      0.653    0.66     0.74
xC - xA == 0    0.808      0.653    1.24     0.39
(Adjusted p values reported -- single-step method)
```

However, in the presence of an intercept term, the full parameter vector $\theta = c(\mu, \beta_1, \ldots, \beta_j)$ can't be estimated due to singularities in the corresponding design matrix. Therefore, a vector of *contrasts* $\gamma^\star$ of the original parameter vector γ is fitted. More technically, a contrast matrix $\mathbf{C}$ is included into this model such that $\beta = \mathbf{C}\gamma^\star$ any we only obtain estimates for $\gamma^\star$, but not for γ:

```
R> aov.ex2 <- aov(y ~ x, data = ex)
R> coef(aov.ex2)

(Intercept)          xB          xC
    -0.2784      0.4279      0.8085
```

The default contrasts in R are so-called treatment contrasts, nothing but differences in means for one baseline group (compare the Dunnett contrasts and the estimated regression coefficients):

```
R> contr.treatment(table(ex$x))

  4 4
4 0 0
4 1 0
4 0 1

R> K %*% contr.treatment(table(ex$x)) %*% coef(aov.ex2)[-1]

        [,1]
B - A 0.4279
C - A 0.8085
```

so that $\mathbf{KC}\hat{\beta}^\star = \mathbf{K}\hat{\beta}$.

When the `mcp` function is used to specify linear hypotheses, the `glht` function takes care of contrasts. Within `mcp`, the matrix of linear hypotheses $\mathbf{K}$ can be written in terms of γ, not $\gamma^\star$. Note that the matrix of linear hypotheses only applies to those elements of $\hat{\gamma}^\star$ attached to factor `x` but not to the intercept term:

```
R> summary(glht(aov.ex2, linfct = mcp(x = K)))

         Simultaneous Tests for General Linear Hypotheses

Multiple Comparisons of Means: User-defined Contrasts


Fit: aov(formula = y ~ x, data = ex)
```

```
Linear Hypotheses:
           Estimate Std. Error t value Pr(>|t|)
B - A == 0    0.428      0.653    0.66     0.74
C - A == 0    0.808      0.653    1.24     0.39
(Adjusted p values reported -- single-step method)
```

or, a little bit more convenient in this simple case:

```
R> summary(glht(aov.ex2, linfct = mcp(x = c("B - A = 0", "C - A = 0"))))

         Simultaneous Tests for General Linear Hypotheses

Multiple Comparisons of Means: User-defined Contrasts


Fit: aov(formula = y ~ x, data = ex)

Linear Hypotheses:
           Estimate Std. Error t value Pr(>|t|)
B - A == 0    0.428      0.653    0.66     0.74
C - A == 0    0.808      0.653    1.24     0.39
(Adjusted p values reported -- single-step method)
```

More generally, inference on linear functions of parameters which can be interpreted as 'means' are known as *multiple comparison procedures* (MCP). For some of the more prominent special cases, the corresponding linear functions can be computed by convenience functions part of **multcomp**. For example, Tukey all-pair comparisons for the factor `x` can be set up using

```
R> glht(aov.ex2, linfct = mcp(x = "Tukey"))

         General Linear Hypotheses

Multiple Comparisons of Means: Tukey Contrasts


Linear Hypotheses:
           Estimate
B - A == 0    0.428
C - A == 0    0.808
C - B == 0    0.381
```

The initial parameterization of the model is automatically taken into account:

```
R> glht(aov.ex, linfct = mcp(x = "Tukey"))
```

```
	 General Linear Hypotheses

Multiple Comparisons of Means: Tukey Contrasts


Linear Hypotheses:
           Estimate
B - A == 0    0.428
C - A == 0    0.808
C - B == 0    0.381
```

3 Two-way ANOVA

For two-way ANOVA models, one might be interested in comparing the levels of the two factors simultaneously. For the model

```
R> mod <- lm(breaks ~ wool + tension, data = warpbreaks)
```

one can extract the appropriate contrast matrices for both factors via

```
R> K1 <- glht(mod, mcp(wool = "Tukey"))$linfct
R> K2 <- glht(mod, mcp(tension = "Tukey"))$linfct
```

and we can simultaneously compare the levels of each factor using

```
R> summary(glht(mod, linfct = rbind(K1, K2)))

	 Simultaneous Tests for General Linear Hypotheses

Fit: lm(formula = breaks ~ wool + tension, data = warpbreaks)

Linear Hypotheses:
           Estimate Std. Error t value Pr(>|t|)
B - A == 0    -5.78       3.16   -1.83   0.2304
M - L == 0   -10.00       3.87   -2.58   0.0457 *
H - L == 0   -14.72       3.87   -3.80   0.0015 **
H - M == 0    -4.72       3.87   -1.22   0.5703
---
Signif. codes:  0 ‘***’ 0.001 ‘**’ 0.01 ‘*’ 0.05 ‘.’ 0.1 ‘ ’ 1
(Adjusted p values reported -- single-step method)
```

where the first comparison is with respect to `wool` and the remaining three to `tension`.

For models with interaction term

```
R> mod <- lm(breaks ~ wool * tension, data = warpbreaks)
```

one might be interested in comparing the levels of `tension` within the levels of `wool`. There are two ways to do this. First, we compute the means of the response for all six combinations of the levels of `wool` and `tension`:

```
R> tmp <- expand.grid(tension = unique(warpbreaks$tension),
+                     wool = unique(warpbreaks$wool))
R> X <- model.matrix(~ wool * tension, data = tmp)
R> glht(mod, linfct = X)

	 General Linear Hypotheses

Linear Hypotheses:
       Estimate
1 == 0     44.6
2 == 0     24.0
3 == 0     24.6
4 == 0     28.2
5 == 0     28.8
6 == 0     18.8
```

which is the same as

```
R> predict(mod, newdata = tmp)

    1     2     3     4     5     6 
44.56 24.00 24.56 28.22 28.78 18.78 
```

We now construct a contrast matrix based on Tukey-contrasts for `tension` in a block-diagonal way, i.e., for each level of `wool`:

```
R> Tukey <- contrMat(table(warpbreaks$tension), "Tukey")
R> K1 <- cbind(Tukey, matrix(0, nrow = nrow(Tukey), ncol = ncol(Tukey)))
R> rownames(K1) <- paste(levels(warpbreaks$wool)[1], rownames(K1), sep = ":")
R> K2 <- cbind(matrix(0, nrow = nrow(Tukey), ncol = ncol(Tukey)), Tukey)
R> rownames(K2) <- paste(levels(warpbreaks$wool)[2], rownames(K2), sep = ":")
R> K <- rbind(K1, K2)
R> colnames(K) <- c(colnames(Tukey), colnames(Tukey))
```

and perform the tests via

```
R> summary(glht(mod, linfct = K %*% X))

	 Simultaneous Tests for General Linear Hypotheses

Fit: lm(formula = breaks ~ wool * tension, data = warpbreaks)

Linear Hypotheses:
             Estimate Std. Error t value Pr(>|t|)   
A:M - L == 0  -20.556      5.157   -3.99   0.0013 **
A:H - L == 0  -20.000      5.157   -3.88   0.0018 **
A:H - M == 0    0.556      5.157    0.11   1.0000   
B:M - L == 0    0.556      5.157    0.11   1.0000   
```

```
B:H - L == 0   -9.444      5.157   -1.83   0.3079
B:H - M == 0  -10.000      5.157   -1.94   0.2554
---
Signif. codes:  0 '***' 0.001 '**' 0.01 '*' 0.05 '.' 0.1 ' ' 1
(Adjusted p values reported -- single-step method)
```

where the effects are the same as

```
R> K %*% predict(mod, newdata = tmp)

             [,1]
A:M - L -20.5556
A:H - L -20.0000
A:H - M   0.5556
B:M - L   0.5556
B:H - L  -9.4444
B:H - M -10.0000
```

We see that the groups (M, L) and (H, L) are different, however, only for wool A (in contrast to the additive model above).

Note that the same results can be obtained by fitting the so-called cell-means model based on a new factor derived as the interaction of `wool` and `tension`:

```
R> warpbreaks$tw <- with(warpbreaks, interaction(tension, wool))
R> cell <- lm(breaks ~ tw - 1, data = warpbreaks)
R> summary(glht(cell, linfct = K))

         Simultaneous Tests for General Linear Hypotheses

Fit: lm(formula = breaks ~ tw - 1, data = warpbreaks)

Linear Hypotheses:
             Estimate Std. Error t value Pr(>|t|)
A:M - L == 0  -20.556      5.157   -3.99   0.0013 **
A:H - L == 0  -20.000      5.157   -3.88   0.0018 **
A:H - M == 0    0.556      5.157    0.11   1.0000
B:M - L == 0    0.556      5.157    0.11   1.0000
B:H - L == 0   -9.444      5.157   -1.83   0.3080
B:H - M == 0  -10.000      5.157   -1.94   0.2554
---
Signif. codes:  0 '***' 0.001 '**' 0.01 '*' 0.05 '.' 0.1 ' ' 1
(Adjusted p values reported -- single-step method)
```

4 Test Procedures

Several global and multiple test procedures are available from the `summary` method of `glht` objects and can be specified via its `test` argument:

- `test = univariate()` univariate p values based on either the t or normal distribution are reported. Controls the type I error for each partial hypothesis only.
- `test = Ftest()` global F test for H_0.
- `test = Chisqtest()` global χ^2 test for H_0.
- `test = adjusted()` multiple test procedures as specified by the `type` argument to `adjusted`: `"single-step"` denotes adjusted p values as computed from the joint normal or t distribution of the **z** statistics (default), `"free"` implements multiple testing procedures under free combinations, `"Shaffer"` implements Bonferroni-adjustments taking logical constraints into account Shaffer [1986] and `"Westfall"` takes both logical constraints and correlations among the **z** statistics into account Westfall [1997]. In addition, all adjustment methods implemented in `p.adjust` can be specified as well.

5 Quality Assurance

The analyses shown in Westfall et al. [1999] can be reproduced using **multcomp** by running the R transcript file in `inst/MCMT`.

References

Charles W. Dunnett. A multiple comparison procedure for comparing several treatments with a control. *Journal of the American Statistical Association*, 50(272):1096–1121, 1955.

Juliet P. Shaffer. Modified sequentially rejective multiple test procedures. *Journal of the American Statistical Association*, 81:826–831, 1986.

Peter H. Westfall. Multiple testing of general contrasts using logical constraints and correlations. *Journal of the American Statistical Association*, 92:299–306, 1997.

Peter H. Westfall, Randall D. Tobias, Dror Rom, Russell D. Wolfinger, and Yosef Hochberg. *Multiple Comparisons and Multiple Tests Using the SAS System*. SAS Insitute, Cary, NC, 1999.
