# Supplementary material for: Comparing Kaolin and Pinolene to Improve Sustainable Grapevine Production during Drought
Source: PLoS One. 2016 Jun 13;11(6):e0156631. doi: 10.1371/journal.pone.0156631 (PMC4905681; doi:10.1371/journal.pone.0156631)
Supplement: S1 File — (GZ) [file pone.0156631.s003.gz › PONE_Brillante_et_al_2016_Code_and_Data/packrat/src/mvtnorm/mvtnorm_1.0-2.tar.gz!/mvtnorm/inst/doc/MVT_Rnews.pdf]

ON MULTIVARIATE t AND GAUSS PROBABILITIES IN R

TORSTEN HOTHORN, FRANK BRETZ, AND ALAN GENZ

Introduction

The numerical computation of a multivariate normal or t probability is often a difficult problem. Recent developments resulted in algorithms for the fast computation of those probabilities for arbitrary correlation structures. We refer to the work described in Genz (1992), Genz (1993) and Genz and Bretz (1999). The procedures proposed in those papers are implemented in package `mvtnorm`, available at CRAN. Basically, the package implements two functions: `pmvnorm` for the computation of multivariate normal probabilities and `pmvt` for the computation of multivariate t probabilities, both for arbitrary means (resp. noncentrality parameters), correlation matrices and hyperrectangular integration regions.

We first illustrate the use of the package using a simple example of the multivariate normal distribution in Section 1. A little more details are given in Section 2. The application of `pmvt` in a multiple testing problem is discussed in Section 3.

1. A Simple Example

Assume that $X = (X_1, X_2, X_3)$ is multivariate normal with correlation matrix

$$\mathbf{\Sigma} = \begin{pmatrix} 1 & \frac{3}{5} & \frac{1}{3} \\ \frac{3}{5} & 1 & \frac{11}{15} \\ \frac{1}{3} & \frac{11}{15} & 1 \end{pmatrix}$$

This document is an updated version of the paper published in R News **1**(2).

and expectation $\mu = (0, 0, 0)^\top$. We are interested in the probability

$$P(-\infty < X_1 \leq 1, -\infty < X_2 \leq 4, -\infty < X_3 \leq 2).$$

This is computed as follows:

```
> library(mvtnorm)
> m <- 3
> sigma <- diag(3)
> sigma[2,1] <- 3/5
> sigma[3,1] <- 1/3
> sigma[3,2] <- 11/15
> pmvnorm(mean=rep(0, m), sigma, lower=rep(-Inf, m), upper=c(1,4,2))
[1] 0.8279847
attr(,"error")
[1] 4.712588e-07
attr(,"msg")
[1] "Normal Completion"
```

First, the lower triangular of the correlation matrix `sigma` is needed. The mean vector is passed to `pmvnorm` by the argument `mean`. The region of integration is given by the vectors `lower` and `upper`, both can have elements `-Inf` or `+Inf`. The value of `pmvnorm` is the estimated integral value with two attributes

- `error`: the estimated absolute error and
- `msg`: a status message, indicating wheater or not the algorithm terminated correctly.

From the results above it follows that

$$P(-\infty < X_1 \leq 1, -\infty < X_2 \leq 4, -\infty < X_3 \leq 2) \approx 0.82798$$

with an absolute error estimate of $2.0e-06$.

2. Details

This section outlines the basic ideas of the algorithms used. The multivariate t distribution (MVT) is given by

$$\mathbf{T}(\mathbf{a},\mathbf{b},\boldsymbol{\Sigma},\nu) = \frac{2^{1-\frac{\nu}{2}}}{\Gamma(\frac{\nu}{2})} \int\limits_0^\infty s^{\nu-1} e^{-\frac{s^2}{2}} \boldsymbol{\Phi}(\frac{s\mathbf{a}}{\sqrt{\nu}}, \frac{s\mathbf{b}}{\sqrt{\nu}}, \boldsymbol{\Sigma}) ds,$$

where the multivariate normal distribution function (MVN)

$$\boldsymbol{\Phi}(\mathbf{a},\mathbf{b},\boldsymbol{\Sigma}) = \frac{1}{\sqrt{|\boldsymbol{\Sigma}|(2\pi)^m}} \int\limits_{a_1}^{b_1}\int\limits_{a_2}^{b_2} ... \int\limits_{a_m}^{b_m} e^{-\frac{1}{2}\mathbf{x}^t\boldsymbol{\Sigma}^{-1}\mathbf{x}} d\mathbf{x},$$

$\mathbf{x} = (x_1, x_2, ..., x_m)^t$, $-\infty \leq a_i < b_i \leq \infty$ for all i, and $\boldsymbol{\Sigma}$ is a positive semi-definite symmetric $m \times m$ matrix. The original integral over an arbitrary m-dimensional, possibly unbounded hyper-rectangle is transformed to an integral over the unit hypercube. These transformations are described in Genz (1992) for the MVN case and in Genz and Bretz (1999) for the MVT case. Several suitable standard integration routines can be applied to this transformed integral. For the present implementation randomized lattice rules were used. Such lattice rules seek to fill the integration region evenly in a deterministic process. In principle, they construct regular patterns, such that the projections of the integration points onto each axis produce an equidistant subdivision of the axis. Robust integration error bounds are obtained by introducing additional shifts of the entire set of integration nodes in random directions. Since this additional randomization step is only performed to introduce a robust Monte Carlo error bound, 10 simulation runs are usually sufficient. For a more detailed description Genz and Bretz (1999) might be referred to.

3. Applications

The multivariate t distribution is applicable in a wide field of multiple testing problems. We will illustrate this using a example studied earlier by Edwards and Berry (1987). For short, the effects of 5 different perfusates in

capillary permeability in cats was investigated by Watson et al. (1987). The data met the assumptions of a standard one-factor ANOVA. For experimental reasons, the investigators were interested in a simultaneous confidence intervals for the following pairwise comparisons: $\beta_1-\beta_2, \beta_1-\beta_3, \beta_1-\beta_5, \beta_4-\beta_2$ and $\beta_4-\beta_3$. Therefore, the matrix of contrast is given by

$$\boldsymbol{C} = \begin{pmatrix} 1 & -1 & 0 & 0 & 0 \\ 1 & 0 & -1 & 0 & 0 \\ 1 & 0 & 0 & 0 & -1 \\ 0 & 1 & 0 & -1 & 0 \\ 0 & 0 & 1 & -1 & 0 \end{pmatrix}.$$

Edwards and Berry (1987) assumed that $\beta = (\beta_1, \dots, \beta_5)$ is multivariate normal with mean β and covariance matrix $\sigma^2 \boldsymbol{V}$, where $\boldsymbol{V}$ is known. Under the null hypothesis $\beta = 0$, we need knowledge about the distribution of the statistic

$$W = \max_{1 \le j \le 5} \left\{ \frac{|c_j(\hat{\beta} - \beta)|}{\hat{\sigma}\sqrt{c_j \boldsymbol{V} c_j^\top}} \right\}$$

where c_j is the jth row of $\boldsymbol{C}$. By assumption, $\hat{\sigma}$ is χ_ν distributed, so under hypothesis W the argument to max follows a multivariate t distribution. Confidence intervals can be obtained by $c_j\hat{\beta} \pm w_\alpha \hat{\sigma}\sqrt{c_j \boldsymbol{V} c_i^\top}$, where w_α is the $1-\alpha$ quantile of the null distribution of W. Using `pmvt`, one can easily compute the quantile for the example cited above.

```
> n <- c(26, 24, 20, 33, 32)
> V <- diag(1/n)
> df <- 130
> C <- c(1,1,1,0,0,-1,0,0,1,0,0,-1,0,0,1,0,0,0,-1,-1,0,0,-1,0,0)
> C <- matrix(C, ncol=5)
> ### covariance matrix
> cv <- C %*% V %*% t(C)
> ### correlation matrix
```

```
> dv <- t(1/sqrt(diag(cv)))
> cr <- cv * (t(dv) %*% dv)
> delta <- rep(0,5)
> qmvt(0.95, df = df, delta = delta, corr = cr, abseps = 0.0001,
+      maxpts = 100000, tail = "both")
$quantile
[1] 2.561249

$f.quantile
[1] 3.599266e-05
attr(,"error")
[1] 8.724695e-05
attr(,"msg")
[1] "Normal Completion"

$iter
[1] 9

$init.it
[1] NA

$estim.prec
[1] 6.103516e-05
```

`n` is the sample size vector of each level of the factor, `V` is the covariance matrix of β. With the contrasts $\boldsymbol{C}$ we can compute the correlation matrix `cr` of $\boldsymbol{C}\beta$. Finally, we are interested in the 95% quantile of W. The `alpha` quantile can now be computed easily using `pmvt`. The 95% quantile of W in this example is 2.56 , Edwards and Berry (1987) obtained the same result using 80.000 simulation runs. The computation needs 8.06 seconds total time on a Pentium III 450 MHz with 256 MB memory.

Using package mvtnorm, the efficient computation of multivariate normal or t probabilities is now available in R. We hope that this is helpful to users / programmers who deal with multiple testing problems.

References

Don Edwards and Jack J. Berry. The efficiency of simulation-based multiple comparisons. *Biometrics*, 43:913–928, December 1987.

A. Genz and F. Bretz. Numerical computation of multivariate t-probabilities with application to power calculation of multiple contrasts. *Journal of Statistical Computation and Simulation*, 63:361–378, 1999.

Alan Genz. Numerical computation of multivariate normal probabilities. *Journal of Computational and Graphical Statistics*, 1:141–149, 1992.

Alan Genz. Comparison of methods for the computation of multivariate normal probabilities. *Computing Science and Statistics*, 25:400–405, 1993.

P.D. Watson, M. B. Wolf, and I.S. Beck-Montgemery. Blood and isoproterenol reduce capillary permeability in cat hindlimb. *The American Journal of Physiology*, 252:H47–H53, 1987.

Friedrich-Alexander-Universität Erlangen-Nürnberg, Institut für Medizininformatik, Biometrie und Epidemiologie, Waldstrasse 6, D-91054 Erlangen

E-mail address: Torsten.Hothorn@rzmail.uni-erlangen.de

Universität Hannover, LG Bioinformatik, FB Gartenbau, Herrenhäuser Str. 2, D-30419 Hannover

E-mail address: bretz@ifgb.uni-hannover.de

Department of Mathematics, Washington State University, Pullman, WA 99164-3113 USA

E-mail address: alangenz@wsu.edu
