# Supplementary material for: Comparing Kaolin and Pinolene to Improve Sustainable Grapevine Production during Drought
Source: PLoS One. 2016 Jun 13;11(6):e0156631. doi: 10.1371/journal.pone.0156631 (PMC4905681; doi:10.1371/journal.pone.0156631)
Supplement: S1 File — (GZ) [file pone.0156631.s003.gz › PONE_Brillante_et_al_2016_Code_and_Data/packrat/src/proto/proto_0.3-10.tar.gz!/proto/vignettes/proto-dot.pdf]

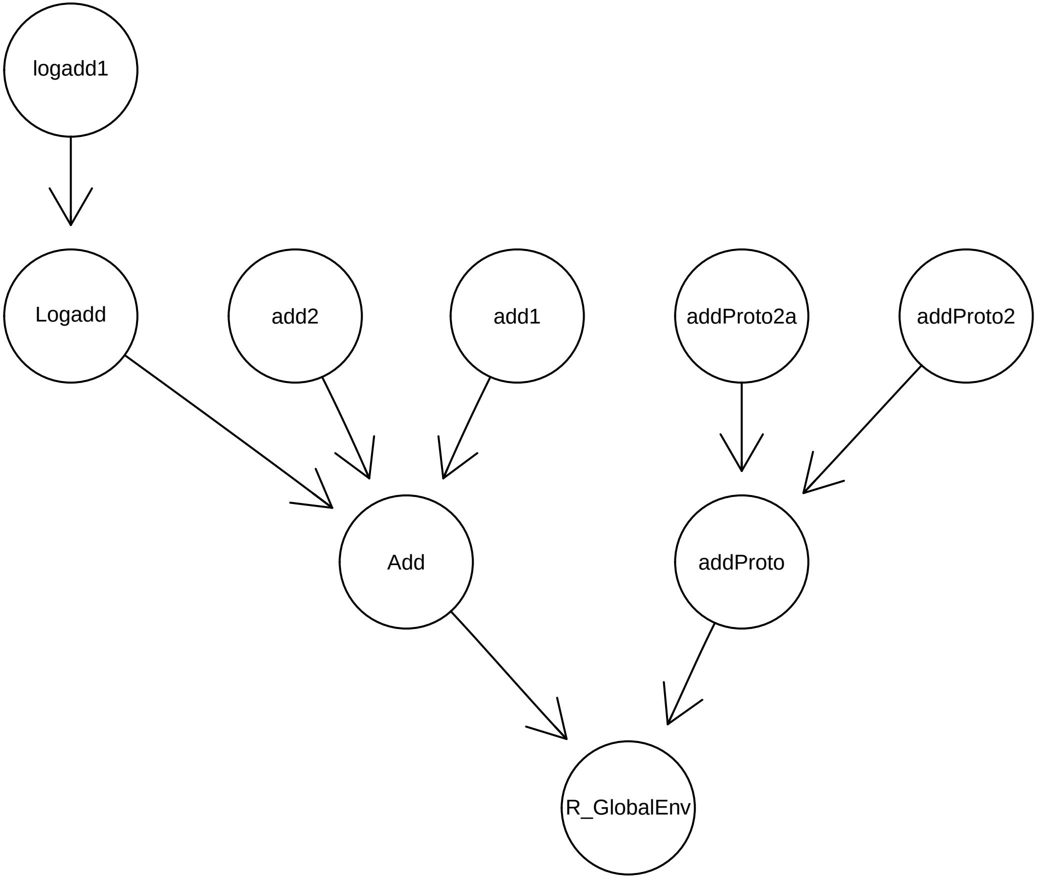

logadd1
Logadd
add2
add1
addProto2a
addProto2
Add
addProto
R_GlobalEnv
