# Supplementary material for: Comparing Kaolin and Pinolene to Improve Sustainable Grapevine Production during Drought
Source: PLoS One. 2016 Jun 13;11(6):e0156631. doi: 10.1371/journal.pone.0156631 (PMC4905681; doi:10.1371/journal.pone.0156631)
Supplement: S1 File — (GZ) [file pone.0156631.s003.gz › PONE_Brillante_et_al_2016_Code_and_Data/packrat/src/proto/proto_0.3-10.tar.gz!/proto/inst/doc/protoref.pdf]

proto reference card

Creation

`proto` — `proto(., expr, envir, ... )` embeds the components specified in `expr` and/or `...` into the `proto` object or environment specified by `envir`. A new object is created if `envir` is omitted. The parent of the object is set to `.` . The parent object, `.`, defaults to the parent of `envir` or the current environment if `envir` is missing. `expr` and `...` default to empty specifications. The returned object will contain `.that` and `.super` variables referring to the object itself and the parent of the object, respectively.

Coercion

`as.proto` — If `x` is a `proto` object or environment then `x` is returned as a `proto` object with the values of `.that` and `.super` inserted in the case of an environment or refreshed in the case of a proto object. If `x` is a list then additional arguments are available: `as.proto(x, envir, parent, FUN, all.names, ...)`. Each component of `x` is copied into `envir`. `envir` may be an `environment` or `proto` object. If it is missing a new `proto` object is created. If `all.names = FALSE` then only list components whose names do not begin with a dot are copied. If `FUN` is specified then, in addition, only list components `v` for which `FUN(v)` is `TRUE` are copied. If `parent` is specified then the resulting `proto` object will have that parent. Otherwise, it will have the parent of `envir` if `envir` was specified. If neither are specified the parent defaults to the current environment.

Standard methods

`$` — `obj$x` searches `proto` object `obj` for `x`. If the name `x` does not begin with two dots then ancestors are searched if the name is not found in `obj`. If `x` is a variable or if `obj` is `.super` or `.that` then `x` is returned. Otherwise, the call `obj$x(...)` is equivalent to the call `get("x", obj)(obj, ...)`. If it is desired to return a method as a value rather than in the context of a call then use `get("x", obj)` (or `obj[["x"]] x` is known to be directly in `obj`) rather than `$` syntax.

`$<-` — `obj$x <- value` sets `x` in `proto` object `obj` to `value` creating `x` if not present. If `obj` is `.super` then a side effect is to set the parent of `obj` to `value`.

`is.proto(x)` — returns `TRUE` if `x` is a `proto` object and othewise returns `FALSE`.

Utilities

`graph.proto` — `graph.proto(e, g, child.to.parent)` adds a graph in the sense of the `graph` package representing an ancestor tree among all `proto` objects in `environment` or `proto` object `e` to graph `g`. `e` defaults to the current environment and `g` defaults to an empty graph. `child.to.parent` is a logical variable specifying the direction of arrows. By default they are displayed from children to parents.
