# Supplementary material for: Comparing Kaolin and Pinolene to Improve Sustainable Grapevine Production during Drought
Source: PLoS One. 2016 Jun 13;11(6):e0156631. doi: 10.1371/journal.pone.0156631 (PMC4905681; doi:10.1371/journal.pone.0156631)
Supplement: S1 File — (GZ) [file pone.0156631.s003.gz › PONE_Brillante_et_al_2016_Code_and_Data/packrat/src/proto/proto_0.3-10.tar.gz!/proto/inst/doc/proto.pdf]

proto: An R Package for Prototype Programming

Louis Kates
GKX Associates Inc.

Thomas Petzoldt
Technische Universität Dresden

Abstract

proto is an R package which facilitates a style of programming known as prototype programming. Prototype programming is a type of object oriented programming in which there are no classes. **proto** is simple yet retains the object oriented features of delegation (the prototype counterpart to inheritance) and object oriented dispatch. `proto` can be used to organize the concrete data and procedures in statistical studies and other applications without the necessity of defining classes while still providing convenient access to an object oriented style of programming. Furthermore, it can be used in a class-based style as well so that incremental design can begin with defining the concrete objects and later transition to abstract classes, once the general case is understood, without having to change to object-oriented frameworks. The key goals of the package are to integrate into R while providing nothing more than a thin layer on top of it.

Keywords: prototype programming, delegation, inheritance, clone, object orientated, S3, R.

1. Introduction

1.1. Object Oriented Programming in R

The R system for statistical computing (R Development Core Team 2005, `http://www.R-project.org/`) ships with two systems for object oriented programming referred to as S3 and S4. With the increased interest in object oriented programming within R over the last years additional object oriented programming packages emerged. These include the **R.oo** package (Bengtsson 2003) and the **OOP** package (Chambers and Lang 2001, `http://www.omegahat.org/OOP/`). The common thread among these packages is that they use classes as the basis of inheritance. When a message is sent to an object the class of the object is examined and that class determines the specific function to be executed. In contrast, in prototype programming there are no classes yet it retains much of the power of class-based programming. In the fact, **proto** is so simple that there are only two significant new routine name, `proto`, and `clone2`. The other routines are just the expected support routines such as `as.proto` to coerce objects to proto objects, `$` to access and set proto object components and `is.proto` to check whether an object is a proto object. In addition, `graph.proto` will generate a graphical ancestor tree showing the parent-child relationships among generated `proto` objects.

The aim of the package is to provide a lightweight layer for prototype programming in R written only in R leveraging the existing facilities of the language rather than adding its own.

1.2. History

The concept of prototype programming (Lieberman 1986; Taivalsaari 1996; Noble, Taivalsaari, and Moore 1999) has developed over a number of years with the Self language (Agesen, Bak, Chambers, Chang, Hölzle, Maloney, Smith, and Ungar 1992) being the key evolved programming language to demonstrate the concept. In statistics, the Lisp-based LispStat programming language (Tierney 1990) was the first and possibly only statistical system to feature prototype programming.

Despite having been developed over 20 years ago, and some attempts to enter the mainstream

(e.g. Newtonscript on the Newton computer, which is no longer available, and Javascript where it is available but whose domain of application largely precludes use of prototype programming) prototype programming is not well known due to lack of language support in popular programming languages such as C and Java. It tends to be the domain of research languages or Lisp.

Thus the availability of a popular language, R [1], that does provide the key infrastructure could be important for developers.

This work grew out of the need to organize multiple scenarios of model simulations in ecological modelling (Petzoldt 2003) and was subsequently generalized to the present package. A number of iterations of the code, some motivated by the ever increasing feature set in R, resulted in a series of utilities and ultimately successive versions of an R package developed over a period of several years. An initial version used R lists as the basis of the package. Subsequently the package was changed to use R environments. The first version to use environments stored the receiver object variable in a proxy parent environment which was created on-the-fly at each method call. The present version of the **proto** package passes the receiver object through the argument list, while hiding this from the caller. It defines the `proto` class as a subclass of the `environment` class so that functionality built into R for the environment class is automatically inherited by the `proto` class.

1.3. Overview

It is assumed that the reader has some general familiarity with object oriented programming concepts and with R.

The paper will proceed primarily by example focusing on illustrating the package `proto` through such demonstration. The remainder of the paper is organized as follows: Section 2 explains how `"proto"` objects are created and illustrates the corresponding methods for setting and getting components. It further discusses how object oriented delegation (the prototype programming analogue of inheritance) is handled and finally discusses the internals of the package. This section uses small examples chosen for their simplicity in illustrating the concepts. In Section 3 we provide additional examples of prototype programming in action. Four examples are shown. The first involves smoothing of data. Secondly we demonstrate the calculation of correlation confidence intervals using classical (Fisher Transform) and modern (bootstrapping) methods. Thirdly we demonstrate the development of a binary tree as would be required for a dendrogram. Fourthly, we use the solution of linear equations to illustrate program evolution from object-based to class-based, all within the **proto** framework. Section 4 gives a few summarizing remarks. Finally, an appendix provides a reference card that summarizes the functionality contained in **proto** in terms of its constituent commands.

2. The class `"proto"` and its methods

2.1. Creation of `"proto"` objects

In this section we shall show, by example, the creation of two prototype objects and related operations. The simple idea is that each `"proto"` object is a set of components: functions (methods) and variables, which are tightly related in some way.

A prototype object is an environment holding the variables and methods of the object. [2]

A prototype object is created using the constructor function `proto` (see Appendix B at the end of this paper or **proto** package help for complete syntax of commands).

[1] Some indications of the popularity of R are the high volume mailing lists, international development team, the existence of over 1000 addon packages, conferences and numerous books and papers devoted to R.

[2] In particular this implies that `"proto"` objects have single inheritance, follow ordinary environment scoping rules and have mutable state as environments do.

```
addProto <- proto( x = rnorm(5), add = function(.) sum(.$x) )
```

In this simple example, the `proto` function defines two components: a variable `x` and a method `add`. The variable `x` is a vector of 5 numbers and the method sums those numbers. The `proto` object `addProto` contains the variable and the method. Thus the `addProto` `proto` object can be used to compute the sum of the values stored in it. As shown with the `add` method in this example, formal argument lists of methods must always have a first argument of dot (i.e. `.`) which signifies the object on which the method is operating. The dot refers to the current object in the same way that a dot refers to the current directory in UNIX. Within the method one must refer to other variables and methods in the object by prefacing each with `.$`. For example, in the above we write `sum(.$x)`. Finally, note that the data and the method are very closely related. Such close coupling is important in order to create an easily maintained system.

To illustrate the usage of `proto`, we first load the package and set the random seed to make the examples in this paper exactly reproducible.

```
> library(proto)
> set.seed(123)
```

Then, we create the `proto` object from above and call its `add` method.

```
> addProto <- proto(x = rnorm(5), add = function(.) sum(.$x))
> addProto$add()

[1] 0.9678513
```

We also create another object, `addProto2` with a different `x` vector and invoke its `add` method too.

```
> addProto2 <- addProto$proto(x = 1:5)
> addProto2$add()

[1] 15
```

In the examples above, we created a prototype object `addProto` and then called its `add` method as just explained. The notation `addProto$add` tells the system to look for the `add` method in the `addProto` object. In the expression `addProto$add`, the `proto` object to the left of the dollar sign, `addProto` here, is referred to as the *receiver* object. This expression also has a second purpose which is to pass the receiver object implicitly as the first argument of `add`. Note that we called `add` as if it had zero arguments but, in fact, it has one argument because the receiver is automatically and implicitly supplied as the first argument. In general, the notation `object$method(arguments)` is used to invoke the indicated method of the receiver object using the object as the implicit first argument along with the indicated arguments as the subsequent arguments. As with the `addProto` example, the receiver object not only determines where to find the method but also is implicitly passed to the method through the first argument. The motivation for this notation is to relieve the user of specifying the receiver object twice: once to locate the method in the object and a second time to pass the object itself to the method. The `$` is overloaded by the `proto` class to automatically do both with one reference to the receiver object. Even though, as with the `addProto` example, the first argument is not listed in the call it still must be listed among the formal arguments in the definition of the method. It is conventional to use a dot `.` as the first formal argument in the method/function definition. That is, we call `add` using `addProto$add()` displaying zero arguments but we define `add` in `addProto` displaying one argument `add <- function(.)`, the dot.

In this example, we also created a second object, `addProto2`, which has the first object, `addProto` as its parent. Any reference to a component in the second object that is unsuccessful will cause search to continue in the parent. Thus the call `addProto2$add()` looks for `add` in `addProto2` and

not finding it there searches its parent, `addProto`, where it is, indeed, found. `add` is invoked with the receiver object, `addProto2`, as the value of dot. The call `addProto2$add()` actually causes the `add` in `addProto` to run but it still uses the `x` from `addProto2` since dot (`.`) is `addProto2` here and `add` references `.$x`. Note that the reference to `.$x` in the `add` found in `addProto` does not refer to the `x` in `addProto` itself. The `x` in `addProto2` has overridden the `x` in its parent. This point is important so the reader should take care to absorb this point.

This simple example already shows the key elements of the system and how *delegation* (the prototype programming term for inheritance) works without classes.

We can add new components or replace components in an object and invoke various methods like this:

```
> addProto2$y <- seq(2, 10, 2)
> addProto2$x <- 1:10
> addProto2$add3 <- function(., z) sum(.$x) + sum(.$y) + sum(z)
> addProto2$add()

[1] 55

> addProto2$add3(c(2, 3, 5))

[1] 95

> addProto2$y

[1]  2  4  6  8 10
```

In this example, we insert variable `y` into the object `addProto2` with a value of `seq(2,10,2)`, reset variable `x` to a new value and insert a new method, `add3`. Then we invoke our two methods and display `y`. Again, note that in the case of `protoAdd2$add` the `add` method is not present in `protoAdd2` and so search continues to the parent `addProto` where it is found.

2.2. Internals

So far, we have used simple examples to illustrate the basic manipulation of objects: construction, getting and setting components and method invocation. We now discuss the internals of the package and how it relates to R constructs. `proto` is actually an S3 class which is a subclass of the `environment` class. Every `proto` object is an environment and its class is `c("proto", "environment")`. The `$` accessor is similar to the same accessor in environments except it will use the R `get` function to search up parent links if it cannot otherwise find the object (unlike environments). When accessing a method, `$` automatically supplies the first argument to the method unless the object is `.that` or `.super`. `.that` is a special variable which `proto` adds to every `proto` object denoting the object itself. `.super` is also added to every proto object and is the parent of `.that`. `.that` and `.super` are normally used within methods of an object to refer to other components of the same or parent object, respectively, as opposed to the receiver (`.`). For example, suppose we want `add` in `addProto2` to add the elements of `x` together and the elements of `y` together and then add these two sums. We could redefine add like this:

```
> addProto2$add <- function(.) .super$add(.) + sum(.$y)
```

making use of the `add` already defined in the parent. One exception should be noted here. When one uses `.super`, as above, or `.that` to specify a method then the receiver object must be explicitly specified in argument one (since in those cases the receiver is possibly different than `.super` or `.that` so the system cannot automatically supply it to the call.)

Setting a value is similar to the corresponding operation for environments except that any function, i.e method, which is inserted has its environment set to the environment of the object into which it is being inserted. This is necessary so that such methods can reference `.that` and `.super` using lexical scoping.

In closing this section a few points should be re-emphasized and expanded upon. A `proto` object is an environment whose parent object is the parent environment of the `proto` object. The methods in the `proto` objects are ordinary functions that have the containing object as their environment.

The R `with` function can be used with environments and therefore can be used with `proto` objects since `proto` objects are environments too. Thus `with(addProto, x)` refers to the variable `x` in `proto` object `addProto` and `with(addProto, add)` refers to the method `add` in the same way. `with(addProto, add)(addProto)` can be used to call `add`. These constructs all follow from their corresponding use in environments from which they are inherited.

Because the `with` expressions are somewhat verbose, two common cases can be shortened using the `$` operator. `addProto$x` can be used to refer to variable `x` in `proto` object `addProto` and has the same meaning as `with(addProto, x)`. In particular like `with` but unlike the the behavior of the `$` operator on environments, when used with `proto` objects, `$` will search not only the object itself but also its ancestors. Similarly `addProto$add()` can be used to call method `add` in `addProto` also searching through ancestors if not found in `addProto`. Note that `addProto$add` returns an object of class

`c("instantiatedProtoMethod", "function")` which is derived from `add` such that the first argument, the `proto` object, is already inserted. Note that there is a `print` method for class `"instantiatedProtoMethod"` so printing such objects will display the underlying function but returning such objects is not the same as returning the function without slot one inserted. Thus, if one wants exactly the original `add` as a value one should use `with(addProto, add)` or `addProto$with(add)`.

Within a method, if a variable is referred to without qualification simply as `x`, say, then its meaning is unchanged from how it is otherwise used in R and follows the same scope rules as any variable to resolve its name. If it is desired that the variable have object scope, i.e. looked up in the receiver object and its ancestors, then `.$x` or similar `with` notation, i.e. `with(., x)`, should be used. Similarly `.$f(x)` calls method `f` automatically inserting the receiver object into argument one and using `x` for argument two. It looks for `f` first in the receiver object and then its ancestors.

2.3. Traits

Let us look at the definition of a child object once again. In the code below, `addProto` is the previously defined parent object and the expression `addProto$proto(x = 1:5)` defines a child object of `addProto` and assigns it to variable `addProto2a`.

```
> addProto2a <- addProto$proto(x = 1:5)
> addProto2a$add()
```

```
[1] 15
```

That is, `proto` can be used to create a new child of an existing object by writing the parent object on the left of the `$` and `proto` on its right. Any contents to be added to the new child are listed in arguments of `proto` as shown.

For example, first let us create a class-like structure. In the following `Add` is an object that behaves very much like a class with an `add` method and a method `new` which constructs new objects. In the line creating object `add1` the expression `Add$new(x = 1:5)` invokes the `new` constructor of the receiver object `Add`. The method `new` has an argument of `x = 1:5` which defines an `x` variable in the `add1` object being instantiated. We similarly create another object `add2`.

```
> Add <- proto(add = function(.) sum(.$x), new = function(., x) .$proto(x = x))
> add1 <- Add$new(x = 1:5)
> add1$add()
```

```
[1] 15
```

```
> add2 <- Add$new(x = 1:10)
> add2$add()
```

```
[1] 55
```

An object which contains only methods and variables that are intended to be shared by all its children (as opposed to an object whose purpose is to have its own methods and variables) is known as a *trait* (Agesen *et al.* 1992). It is similar to a class in class-based object oriented programming. Note that the objects `add1` and `add2` have the trait `Add` as their parent. We could implement subclass-like and superclass-like objects by simply defining similar trait objects to be the parent or child of `Add`. For example, suppose we want a class which calculates the sum of the logarithms of the data. We could define:

```
> Logadd <- Add$proto(logadd = function(.) log(.$add()))
> logadd1 <- Logadd$new(1:5)
> logadd1$logadd()
```

```
[1] 2.70805
```

Here the capitalized objects are traits. `Logadd` is a trait. It is a child of `Add` which is also a trait. `logadd1` is an ordinary object, not a trait. One possible design is to create a tree of traits and other objects in which the leaves are ordinary objects and the remaining nodes are traits. This would closely correspond to class-based object oriented programming.

Note that the delegation of methods from one trait to another as in `new` which is inherited by `Logadd` from `Add` is nothing more than the same mechanism by which traits delegate methods to objects since, of course, traits are just objects no different from any other object other than by the conventions we impose on them. This unification of subclassing and instantiation beautifully shows the simplification that prototype programming represents.

2.4. Utilities

The fact that method calls automatically insert the first argument can be used to good effect in leveraging existing R functions while allowing an object-oriented syntax.

For example, `ls()` can be used to list the components of `proto` objects:

```
> addProto$ls()
```

```
[1] "add" "x"
```

Functions like:

```
> addProto$str()
> addProto$print()
> addProto$as.list()
> addProto2a$parent.env()
```

show additional information about the elements. `eapply` can be used to explore more properties such as the the length of each component of an object:

```
> addProto$eapply(length)
```

Another example of some interest in any object oriented system which allows multiple references to one single object is that object identity can be tested using the respective base function:

```
> addProto$identical(addProto2)
```

```
[1] FALSE
```

`proto` does contain a special purpose `str.proto` function but in the main it is important to notice here, that `proto` has no code that is specific to `ls` or any of the other ordinary R functions listed. We are simply making use of the fact that `obj$fun(...)` is transformed into `get("fun", obj)(obj, ...)` by the proto `$` operator. For example, in the case of `addProto$ls()` the system looks for `ls` in object `addProto`. It cannot find it there so it looks to its parent, which is the global environment. It does not find it there so it searches the remainder of the search path, i.e. the path shown by running the R command `search()`, and finally finds it in the base package, invoking it with an argument of `addProto`. Since all `proto` objects are also environments `ls(addProto)` interprets `addProto` as an environment and runs the `ls` command with it. In the `ls` example there were no arguments other than `addProto`, and even that one was implicit, but if there were additional arguments then they would be passed as shown in the `eapply` and `identical` examples above.

2.5. Plotting

The `graph.proto` function can be used to create graphs that can be rendered by the `Rgraphviz` package creating visual representations of ancestor trees (figure 1). That package provides an interface to the GraphViz `dot` program (Ganser and North 2000).

`graph.proto` takes three arguments, all of which are usually omitted. The first argument is a `proto` object (or an environment) out of which all contained `proto` objects and their parents (but not higher order ancestors) are graphed. If it is omitted, the current environment is assumed. The second argument is a graph (in the sense of the `graph` package) to which the nodes and edges are added. If it is omitted an empty graph is assumed. The last argument is a logical variable that specifies the orientation of arrows. If omitted arrows are drawn from children to their parents.

```
> library(Rgraphviz)
> g <- graph.proto()
> plot(g)
```

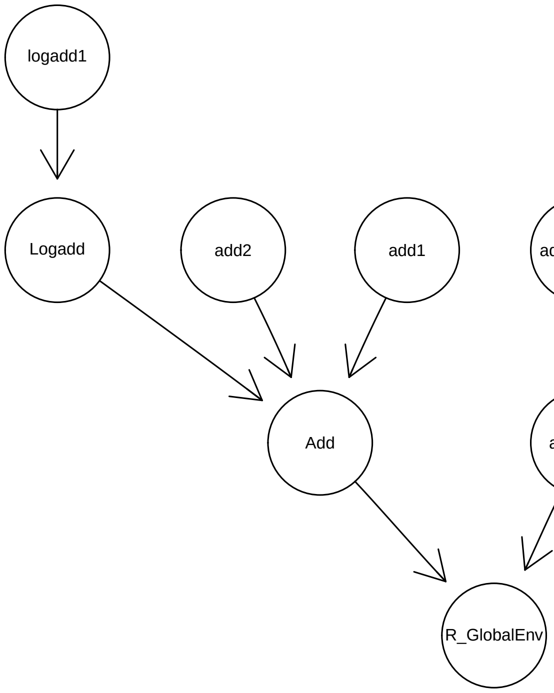

Figure 1: Ancestor tree generated using graph.proto. Edges point from child to parent.

3. Examples

3.1. Smoothing

In the following we create a `proto` object named `oo` containing a vector of data `x` (generated from a simulated autoregressive model) and time points `tt`, an intermediate result `x.smooth`, some plotting parameters `xlab`, `ylab`, `pch`, `col` and three methods `smooth`, `plot` and `residuals` which smooth the data, plot the data and calculate residuals, respectively. We also define `..x.smooth` which holds intermediate results. Names beginning with two dots prevent them from being delegated to children. If we override `x` in a child we would not want an out-of-sync `x.smooth`. Note that the components of an object can be specified using a code block in place of the argument notation we used previously in the `proto` command.

```
> oo <- proto(expr = {
+     x <- rnorm(251, 0, 0.15)
+     x <- filter(x, c(1.2, -0.05, -0.18), method = "recursive")
+     x <- unclass(x[-seq(100)]) * 2 + 20
+     tt <- seq(12200, length = length(x))
+     ..x.smooth <- NA
+     xlab <- "Time (days)"
+     ylab <- "Temp (deg C)"
+     pch <- "."
+     col <- rep("black", 2)
+     smooth <- function(., ...) {
+         .$..x.smooth <- supsmu(.$tt, .$x, ...)$y
+     }
```

```
+     plot <- function(.) with(., {
+         graphics::plot(tt, x, pch = pch, xlab = xlab, ylab = ylab,
+             col = col[1])
+         if (!is.na(..x.smooth[1]))
+             lines(tt, ..x.smooth, col = col[2])
+     })
+     residuals <- function(.) with(., {
+         data.frame(t = tt, y = x - ..x.smooth)
+     })
+ })
```

Having defined our `proto` object we can inspect it, as shown below, using `print` which is automatically invoked if the name of the object, `oo`, is entered on a line by itself. In this case, there is no proto print method so we inherit the environment print method which displays the environment hash code. Although it produces too much output to show here, we could have displayed a list of the entire contents of the object `oo` via `oo$as.list(all.names = TRUE)`. We can get a list of the names of the components of the object using `oo$ls(all.names = TRUE)` and will look at the contents of one component, `oo$pch`.

```
> oo

<environment: 0x04330010>
attr(,"class")
[1] "proto"       "environment"

> oo$ls(all.names = TRUE)

 [1] "..x.smooth" ".super"     ".that"      "col"        "pch"
 [6] "plot"       "residuals"  "smooth"     "tt"         "x"
[11] "xlab"       "ylab"

> oo$pch

[1] "."
```

Let us illustrate a variety of manipulations. We will set up the output to plot 2 plots per screen using `mfrow`. We change the plotting symbol, smooth the data, invoke the `plot` method to display a plot of the data and the smooth and then plot the residuals in the second plot (figure 2).

```
> par(mfrow = c(1, 2))
> oo$pch <- 20
> oo$smooth()
> oo$plot()
> plot(oo$residuals(), type = "l")
```

Now let us illustrate the creation of a child object and delegation. We create a new child object of `oo` called `oo.res`. We will override the `x` value in its parent by setting `x` in the child to the value of the residuals in the parent. We will also override the `pch` and `ylab` plotting parameters. We will return to 1 plot per screen and run `plot` using the `oo.res` object as the receiver invoking the `smooth` and `plot` methods (which are delegated from the parent `oo`) with the data in the child (figure 3).

```
> oo.res <- oo$proto(pch = "-", x = oo$residuals()$y, ylab = "Residuals deg K")
> par(mfrow = c(1, 1))
> oo.res$smooth()
> oo.res$plot()
```

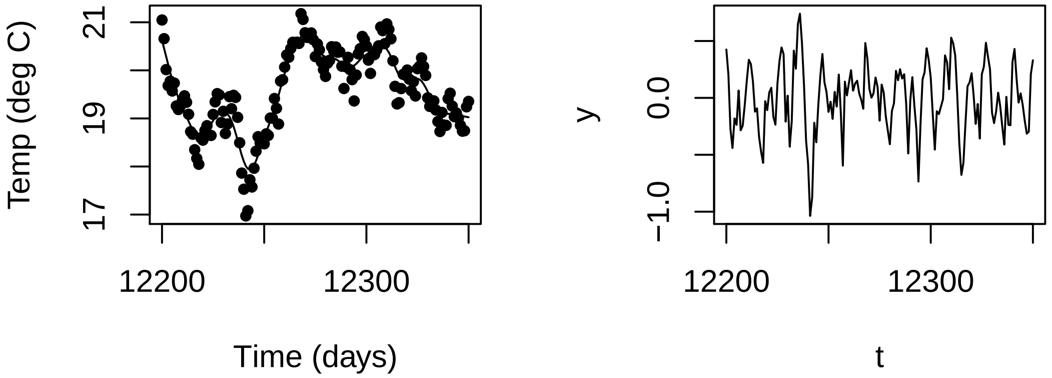

Figure 2: Data and smooth from `oo$plot()` (left) and plot of `oo$residuals()` (right).

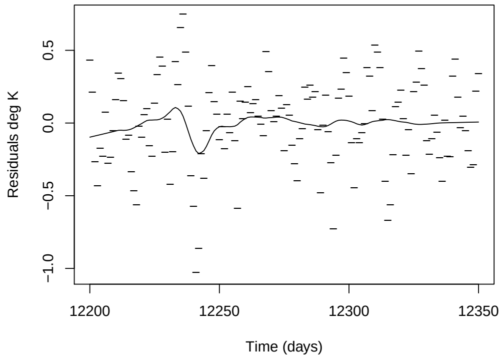

Figure 3: Output of `oo.res$plot()`. `oo.res$x` contains the residuals from `oo`.

Now we make use of delegation to change the parent and child in a consistent way with respect to certain plot characteristics. We have been using a numeric time axis. Let us interpret these numbers as the number of days since the Epoch, January 1, 1970, and let us also change the plot colors.

```
> oo$tt <- oo$tt + as.Date("1970-01-01")
> oo$xlab <- format(oo.res$tt[1], "%Y")
> oo$col <- c("blue", "red")
```

We can introduce a new method, `splot`, into the parent `oo` and have it automatically inherited by its children. In this example it smooths and then plots and we use it with both `oo` and `oo.res` (figure 4).

```
> oo$splot <- function(., ...) {
+     .$smooth(...)
```

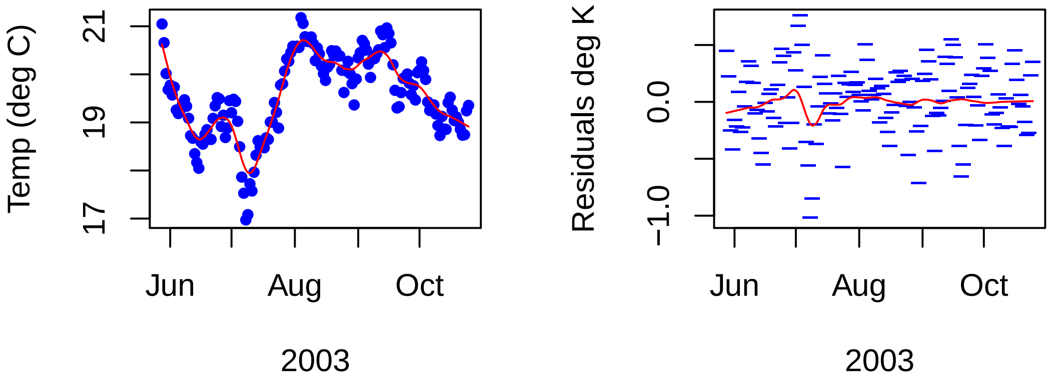

Figure 4: Plotting options and `splot` function applied to both parent (left) and child (right) object

```
+     .$plot()
+ }
> par(mfrow = c(1, 2))
> oo$splot(bass = 2)
> oo.res$splot()
```

Numerous possibilities exist to make use of the mechanisms shown, so one may create different child objects, apply different smoothing parameters, overwrite the smoothing function with a lowess smoother and finally compare fits and residuals.

Now lets change the data and repeat the analysis. Rather than overwrite the data we will preserve it in `oo` and create a child `oos` to hold an analysis with sinusoidal data.

```
> oos <- oo$proto(expr = {
+     tt <- seq(0, 4 * pi, length = 1000)
+     x <- sin(tt) + rnorm(tt, 0, 0.2)
+ })
> oos$splot()
```

Lets perform the residual analysis with `oos`. We will make a deep copy of `oo.res`, i.e. duplicate its contents and not merely delegate it, by copying `oo.res` to a list from which we create the duplicate, or cloned, `proto` object (figure 5 and 6):

```
> oos.res <- as.proto(oo.res$as.list(), parent = oos)
> oos.res$x <- oos$residuals()$y
> oos.res$splot()
```

We have delegated variables and methods and overridden both. Thus, even with such a simple analysis, object orientation and delegation came into play. The reader can plainly see that smoothing and residual analysis were not crucial to the example and this example could be replaced with any statistical analysis including likelihood or other estimation techniques, time series, survival analysis, stochastic processes and so on. The key aspect is just that we are performing one-of

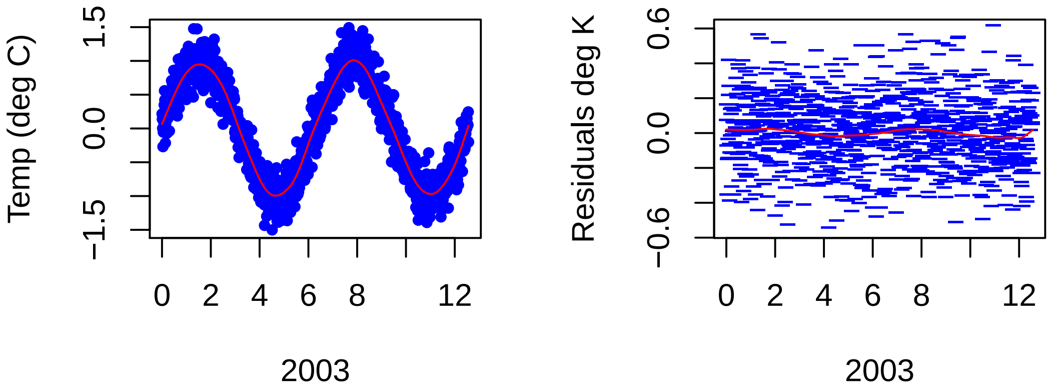

Figure 5: Smoothing of sinusoidal data (left) and of their residuals (right)

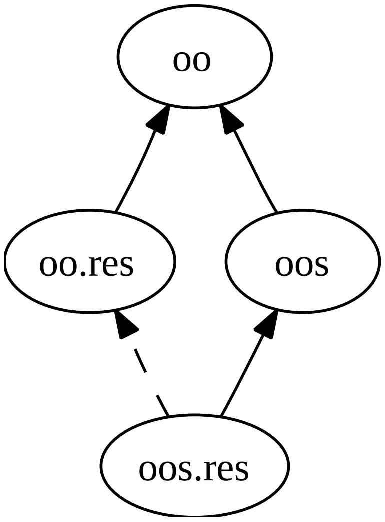

Figure 6: Cloning (dashed line) and delegation (solid line). Edges point from child to parent.

analyses and do not want to set up an elaborate class infrastructure but just want to directly create objects to organize our calculations while relying on delegation and dispatch to eliminate redundancy.

3.2. Correlation, Fisher's Transform and Bootstrapping

The common approach to confidence intervals for the correlation coefficient is to assume normality of the underlying data and then use Fisher's transform to transform the correlation coefficient to an approximately normal random variable. Fisher showed that with the above normality assumption, transforming the correlation coefficient using the hyperbolic arc tangent function yields a random variable approximately distributed with an $\frac{N(p,1)}{\sqrt{(n-3)}}$ distribution. The transformed random variable

can be used to create normal distribution confidence intervals and the procedure can be back transformed to get confidence intervals for the original correlation coefficient.

A more recent approach to confidence intervals for the correlation coefficient is to use bootstrapping. This does not require the assumption of normality of the underlying distribution and requires no special purpose theory devoted solely to the correlation coefficient,

Let us calculate the 95% confidence intervals using Fisher's transform first. We use `GNP` and `Unemployed` from the Longley data set. First we retrieve the data set and extract the required columns into `x`. Then we set `n` to the number of cases and `pp` to the percentiles of interest. Finally we calculate the sample correlation and create a function to calculate the confidence interval using Fisher's Transform. This function not only returns the confidence interval but also stores it in `CI` in the receiver object.

```
> longley.ci <- proto(expr = {
+     data(longley)
+     x <- longley[, c("GNP", "Unemployed")]
+     n <- nrow(x)
+     pp <- c(0.025, 0.975)
+     corx <- cor(x)[1, 2]
+     ci <- function(.) (.$CI <- tanh(atanh(.$corx) + qnorm(.$pp)/sqrt(.$n -
+         3)))
+ })
```

Now let us repeat this analysis using the bootstrapping approach. We derive a new object `longley.ci.boot` as child of `longley.ci`, setting the number of replications, `N`, and defining the procedure, `ci` which does the actual bootstrap calculation.

```
> longley.ci.boot <- longley.ci$proto({
+     N <- 1000
+     ci <- function(.) {
+         corx <- function(idx) cor(.$x[idx, ])[1, 2]
+         samp <- replicate(.$N, corx(sample(.$n, replace = TRUE)))
+         (.$CI <- quantile(samp, .$pp))
+     }
+ })
```

In the example code below the first line runs the Fisher Transform procedure and the second runs the bootstrap procedure. Just to check that we have performed sufficient bootstrap iterations we rerun it in the third line, creating a delegated object on-the-fly running its `ci` method and then immediately throwing the object away. The fact that 4,000 replications give roughly the same result as 1,000 replications satisfies us that we have used a sufficient number of replications.

```
> longley.ci$ci()

[1] 0.1549766 0.8464304

> longley.ci.boot$ci()

     2.5%     97.5% 
0.2299395 0.8211854 

> longley.ci.boot$proto(N = 4000)$ci()

     2.5%     97.5% 
0.2480999 0.8259276 
```

We now have the results stored in two objects nicely organized for the future. Note, again, that despite the simplicity of the example we have used the features of object oriented programming, coupling the data and methods that go together, while relying on delegation and dispatch to avoid duplication.

3.3. Dendrograms

In Gentleman (2002) there is an S4 example of creating a binary tree for use as a dendrogram. Here we directly define a binary tree with no setup at all. To keep it short we will create a binary tree of only two nodes having a root whose left branch points to a leaf. The leaf inherits the `value` and `incr` components from the root. The attractive feature is that the leaf be defined as a child of the parent using `proto` before the parent is even finished being defined. Compared to the cited S4 example where it was necessary to create an extra class to introduce the required level of indirection there is no need to take any similar action.

`tree` is the root node of the tree. It has four components. A method `incr` which increments the `value` component, a `..Name`, the `value` component itself and the left branch `..left`. `..left` is itself a proto object which is a child of `tree`. The leaf inherits the `value` component from its parent, the root. As mentioned, at the time we define `..left` we have not even finished defining `tree` yet we are able to implicitly reference the yet to be defined parent.

```
> tree <- proto(expr = {
+     incr <- function(., val) .$value <- .$value + val
+     ..Name <- "root"
+     value <- 3
+     ..left <- proto(expr = {
+         ..Name = "leaf"
+     })
+ })
```

Although this is a simple structure we could have embedded additional children into `root` and `leaf` and so on recursively making the tree or dendrogram arbitrarily complex.

Let us do some computation with this structure. We display the `value` fields in the two nodes, increment the value field in the root and then display the two nodes again to show .that the leaf changed too.

```
> cat("root:", tree$value, "leaf:", tree$..left$value, "\n")

root: 3 leaf: 3

> tree$incr(1)
> cat("root:", tree$value, "leaf:", tree$..left$value, "\n")

root: 4 leaf: 4
```

If we increment `value` in `leaf` directly (see the example below where we increment it by 10) then it receives its own copy of `value` so from that point on `leaf` no longer inherits `value` from `root`. Thus incrementing the root by 5 no longer increments the `value` field in the leaf.

```
> tree$..left$incr(10)
> cat("root:", tree$value, "leaf:", tree$..left$value, "\n")

root: 4 leaf: 14
```

```
> tree$incr(5)
> cat("root:", tree$value, "leaf:", tree$..left$value, "\n")

root: 9 leaf: 14
```

3.4. From Prototypes to Classes

In many cases we will use **proto** for a design that uses prototypes during the full development cycle. In other cases we may use it in an incremental way starting with prototypes but ultimately transitioning to classes. As shown in Section 2.3 the **proto** package is powerful enough to handle class-based as well as class-free programming. Here we illustrate this process of incremental design starting with concrete objects and then over time classifing them into classes, evolving a class-based program. **proto** provides a smooth transition path since it can handle both the class-free and the class-based phases – there is no need to switch object systems part way through. In this example, we define an object which holds a linear equation, `eq`, represented as a character string in terms of the unknown variable `x` and a `print` and a `solve` method. We execute the `print` method to solve it. We also create child object `lineq2` which overrides `eq` and execute its `print` method.

```
> lineq <- proto(eq = "6*x + 12 - 10*x/4 = 2*x", solve = function(.) {
+     e <- eval(parse(text = paste(sub("=", "-(", .$eq), ")")),
+         list(x = 0+1i))
+     -Re(e)/Im(e)
+ }, print = function(.) cat("Equation:", .$eq, "Solution:", .$solve(),
+     "\n"))
> lineq$print()

Equation: 6*x + 12 - 10*x/4 = 2*x Solution: -8

> lineq2 <- lineq$proto(eq = "2*x = 7*x-12+x")
> lineq2$print()

Equation: 2*x = 7*x-12+x Solution: 2
```

We could continue with enhancements but at this point we decide that we have a general case and so wish to abstract `lineq` into a class. Thus we define a trait, `Lineq`, which is just `lineq` minus `eq` plus a constructor `new`. The key difference between `new` and the usual `proto` function is that with `new` the initialization of `eq` is mandatory. Having completed this definition we instantiate an object of class/trait `Lineq` and execute it.

```
> Lineq <- lineq
> rm(eq, envir = Lineq)
> Lineq$new <- function(., eq) proto(., eq = eq)
> lineq3 <- Lineq$new("3*x=6")
> lineq3$print()

Equation: 3*x=6 Solution: 2
```

Note how we have transitioned from a prototype style of programming to a class-based style of programming all the while staying within the **proto** framework.

4. Summary

4.1. Benefits

The key benefit of the **proto** package is to provide access to a style of programming that has not been conveniently accessible within R or any other mainstream language today.

proto can be used in two key ways: class-free object oriented programming and class-based object oriented programming.

A key application for **proto** in class-free programming is to wrap the code and data for each run of a particular statistical study into an object for purposes of organization and reproducibility. It provides such organization directly and without the need and overhead of class definitions yet still provides the inheritance and dispatch advantages of object oriented programming. We provide examples of this style of programming in Section 3.1 and Section 3.2. A third example in Section 3.3 illustrates a beneficial use of **proto** with recursive data structures.

Another situation where prototype programming is of interest is in the initial development stages of a program. In this case, the design may not be fully clear so it is more convenient to create concrete objects individually rather than premature abstractions through classes. The `graph.proto` function can be used to generate visual representations of the object tree suggesting classifications of objects so that as the program evolves the general case becomes clearer and in a bottom up fashion the objects are incrementally abstracted into classes. In this case, **proto** provides a smooth transition path since it not only supports class-free programming but, as explained in the Section 2.3, is sufficiently powerful to support class-based programming, as well.

4.2. Conclusion

The package **proto** provides an S3 subclass of the `environment` class for constructing and manipulating object oriented systems without classes. It can also emulate classes even though classes are not a primitive structure. Its key design goals are to provide as simple and as thin a layer as practically possible while giving the user convenient access to this alternate object oriented paradigm. This paper describes, by example, how prototype programming can be carried out in R using **proto** and illustrates such usage. Delegation, cloning traits and general manipulation and incremental development are all reviewed by example.

Computational details

The results in this paper were obtained using R 2.1.0 with the package **proto** 0.3–2. R itself and the **proto** package are available from CRAN at `http://CRAN.R-project.org/`. The GraphViz software is available from `http://www.graphviz.org`.

References

Agesen O, Bak L, Chambers C, Chang BW, Hölzle U, Maloney J, Smith RB, Ungar D (1992). *The SELF Programmer's Reference Manual*. 2550 Garcia Avenue, Mountain View, CA 94043, USA. Version 2.0.

Bengtsson H (2003). "The **R.oo** Package – Object-Oriented Programming with References Using Standard R Code." In K Hornik, F Leisch, A Zeileis (eds.), "Proceedings of the 3rd International Workshop on Distributed Statistical Computing," Vienna, Austria. URL `http://www.maths.lth.se/help/R/`.

Chambers JM, Lang DT (2001). "Object-Oriented Programming in R." *R News*, **1**(3), 17–19. URL `http://CRAN.R-project.org/doc/Rnews/`.

Ganser ER, North SC (2000). "An Open Graph Visualization System with Applications to Software Engineering." *Software–Practice and Experience*, **30**(11), 1203–1233. URL `http://www.graphviz.org`.

Gentleman R (2002). "S4 Classes in 15 Pages More or Less." URL http://www.bioconductor.org/develPage/guidelines/programming/S4Objects.pdf.

Kates L, Petzoldt T (2004). "Prototype-Based Programming in Statistical Computation." URL http://r-proto.googlecode.com/files/prototype_approaches.pdf.

Lieberman H (1986). "Using Prototypical Objects to Implement Shared Behavior in Object-Oriented Systems." In N Meyrowitz (ed.), "Proceedings of the Conference on Object-Oriented Programming Systems, Languages, and Applications (OOPSLA)," volume 21(11), pp. 214–223. ACM Press, New York, NY. URL http://citeseer.ist.psu.edu/lieberman86using.html.

Noble J, Taivalsaari A, Moore I (1999). *Prototype-Programming*. Springer-Verlag Singapore Pte. Ltd.

Petzoldt T (2003). "R as a Simulation Platform in Ecological Modelling." *R News*, **3**(3), 8–16. URL http://CRAN.R-project.org/doc/Rnews/.

R Development Core Team (2005). *R: A language and environment for statistical computing*. R Foundation for Statistical Computing, Vienna, Austria. ISBN 3-900051-07-0, URL http://www.R-project.org.

Taivalsaari A (1996). "Classes vs. Prototypes Some Philosophical and Historical Observations." *Journal of Object-Oriented Programming*, **10**(7), 44–50. URL http://www.csee.umbc.edu/331/resources/papers/Inheritance.pdf.

Tierney L (1990). *LISP-STAT: An Object-Oriented Environment for Statistical Computing and Dynamic Graphics*. Wiley, New York, NY.

A. Frequently Asked Questions

1. What scope do unqualified object references within methods use?

   A **proto** object is an environment and that environment is the environment of the methods in it (by default). That is, unqualified object references within a **proto** method look first in the method itself and secondly in the **proto** object containing the method. This is referred to as object scope as opposed to lexical scope or dynamic scope. It allows simple situations, where delegation is not used, to use unqualified names. Thus simple situations remain simple. (Kates and Petzoldt 2004) discusses the fragile base class problem which relates to this question. Also note that if a **proto** object is created via the `proto` function using an argument of `funEnvir = FALSE` then the environment of the function/method will not be set as just described (but rather it will retain its original environment) so the above does not apply. This can be used for instances when non-default processing is desirable.

2. Why does `obj$meth` not return the method, `meth`?

   Conceptually `obj$meth` returns `meth` but with `obj` already inserted into its first argument. This is termed an instantiated **proto** method and is of S3 class `"instantiatedProtoMethod"`.

   In contrast, the method itself (i.e. the uninstantited method) would not have the first argument already inserted. To return the method itself use `with(obj, meth`.

   The main advantage of a design that makes the distinction between instantiated and uninstantiated methods is that uninstantiated methods are never changed so debugging can be more readily carried out (as discussed in the next question and answer).

3. How does one debug a method?

   proto does not dynamically redefine methods. This has the advantage that the ordinary R `debug` and `undebug` commands can be used. When using these be sure that to use them with the uninstantiated method itself and not the instantiated method derived from it. That is, use:

   ```
   with(obj, debug(meth))
   ```

   and not

   ```
   debug(obj$meth) # wrong
   ```

4. Is multiple inheritance supported?

   No. **proto** is just a thin layer on top of R environments and R environments provide single inheritance only. (Kates and Petzoldt 2004) discusses some ways of handling situations which would otherwise require multiple inheritance.

5. Does **proto** support lazy evaluation?

   Since `proto` methods are just R functions they do support lazy evaluation; however, the `proto` function itself does evaluate its arguments. To get the effect of lazy evaluation when using the `proto` function replace any properties with a function.

   If the caller is the parent of the `proto` object then its particularly simple. Note how we got the equivalent of lazy evaluation in the second example where f is a function:

```
# eager evaluation
x <- 0
p <- proto(f = x, g = function(.) $x)
x <- 1
p$f # 0

# versus making f a function

# simulates lazy evaluation
x <- 0
p <- proto(f = function(.) x, g = function(.) .$x)
x <- 1
p$f() # 1
```

If we cannot guarantee that the proto object has the caller as its parent then ensure that the environment of the function has not been reset. If no method needs to reference `.that` or `.super` then we can arrange for that using `funEnvir=FALSE` as seen here in the second example:

```
# does not work as intended
x <- 0
p <- proto(x = 99)
q <- p$proto(f = function(.) x, g = function(.) .$x)
x <- 1
q$f() # 99

# does work
x <- 0
p <- proto(x = 99)
q <- p$proto(f = function(.) x, g = function(.) .$x, funEnvir = FALSE)
x <- 1
q$f() # 1
```

If we wish only to not reset the function used to simulate lazy evaluation then we can do it using either of the two equivalent alternatives below. `g` is an ordinary method whose environment is reset to `q` whereas `f` is a function whose environment is not reset and serves to provide lazy evaluation for `x` found in the caller.

```
x <- 0
p <- proto(x = 99)
# g will use q's y in children of q even if those children
# override y
q <- p$proto(y = 25, g = function(.) .that$y + .$x)
q[["f"]] <- function(.) x
x <- 1
q$f() # 1

# equivalent alternative

x <- 0
p <- proto(x = 99)
q <- proto(f = function(.) x, funEnvir = FALSE,
envir = p$proto(y = 25, g = function(.) .that$y + .$x))
```

```
x <- 1
q$f() # 1
```

B. Reference Card

Creation

`proto`	`proto(., expr, envir, ... )` embeds the components specified in `expr` and/or `...` into the `proto` object or environment specified by `envir`. A new object is created if `envir` is omitted. The parent of the object is set to `.` . The parent object, `.`, defaults to the parent of `envir` or the current environment if `envir` is missing. `expr` and `...` default to empty specifications. The returned object will contain `.that` and `.super` variables referring to the object itself and the parent of the object, respectively.

Coercion

`as.proto`	If `x` is a `proto` object or environment then `x` is returned as a `proto` object with the values of `.that` and `.super` inserted in the case of an environment or refreshed in the case of a proto object. If `x` is a list then additional arguments are available: `as.proto(x, envir, parent, FUN, all.names, ...)`. Each component of `x` is copied into `envir`. `envir` may be an `environment` or `proto` object. If it is missing a new `proto` object is created. If `all.names = FALSE` then only list components whose names do not begin with a dot are copied. If `FUN` is specified then, in addition, only list components `v` for which `FUN(v)` is `TRUE` are copied. If `parent` is specified then the resulting `proto` object will have that parent. Otherwise, it will have the parent of `envir` if `envir` was specified. If neither are specified the parent defaults to the current environment.

Standard methods

`$`	`obj$x` searches `proto` object `obj` for `x`. If the name `x` does not begin with two dots then ancestors are searched if the name is not found in `obj`. If `x` is a variable or if `obj` is `.super` or `.that` then `x` is returned. Otherwise, the call `obj$x(...)` is equivalent to the call `get("x", obj)(obj, ...)`. If it is desired to return a method as a value rather than in the context of a call then use `get("x", obj)` (or `obj[["x"]]` `x` is known to be directly in `obj`) rather than `$` syntax.
`$<-`	`obj$x <- value` sets `x` in `proto` object `obj` to `value` creating `x` if not present. If `obj` is `.super` then a side effect is to set the parent of `obj` to `value`.
`is.proto(x)`	returns `TRUE` if `x` is a `proto` object and othewise returns `FALSE`.

Utilities

`graph.proto`	`graph.proto(e, g, child.to.parent)` adds a graph in the sense of the `graph` package representing an ancestor tree among all `proto` objects in `environment` or `proto` object `e` to graph `g`. `e` defaults to the current environment and `g` defaults to an empty graph. `child.to.parent` is a logical variable specifying the direction of arrows. By default they are displayed from children to parents.
