# Supplementary material for: Comparing Kaolin and Pinolene to Improve Sustainable Grapevine Production during Drought
Source: PLoS One. 2016 Jun 13;11(6):e0156631. doi: 10.1371/journal.pone.0156631 (PMC4905681; doi:10.1371/journal.pone.0156631)
Supplement: S1 File — (GZ) [file pone.0156631.s003.gz › PONE_Brillante_et_al_2016_Code_and_Data/packrat/src/R6/R6_2.0.1.tar.gz!/R6/inst/doc/Debugging.html]

Debugging methods in R6 objects


# Debugging methods in R6 objects

- Enabling debugging for all future instances of a class
- Debugging methods in individual objects

Debugging methods in R6 classes is somewhat different from debugging normal R functions.

RStudio breakpoints don’t work in R6 class methods. The simplest way to debug code is to insert a `browser()` line where you want to open a debugging console, reload the classes, and then step through your code. But this involves modifying your code, reloading it, and re-instantiating any objects you want to test.

## Enabling debugging for all future instances of a class

R6 generator objects have a method called `debug()` which will enable debugging for a method. This will affect all instances of the class that are created after the `debug()` is called.

```
# An example class
Simple <- R6Class("Simple",
  public = list(
    x = 10,
    getx = function() self$x
  )
)

# This will enable debugging the getx() method for objects of the 'Simple'
# class that are instantiated in the future.
Simple$debug("getx")

s <- Simple$new()
s$getx()
# [Debugging prompt]
```

To disable debugging for future instances, use the generator’s `undebug()` method:

```
# Disable debugging for future instances:
Simple$undebug("getx")

s <- Simple$new()
s$getx()
#> [1] 10
```

## Debugging methods in individual objects

To enable debugging for a method in a single instance of an object, use the `debug()` function (not the `debug()` method in the generator object).

```
s <- Simple$new()
debug(s$getx)
s$getx()
# [Debugging prompt]
```

Use `undebug()` to disable debugging on an object’s method.

```
undebug(s$getx)
s$getx()
#> [1] 10
```

You can also use the `trace()` function to specify where in a method you want to drop into the debugging console.
