# Supplementary material for: Comparing Kaolin and Pinolene to Improve Sustainable Grapevine Production during Drought
Source: PLoS One. 2016 Jun 13;11(6):e0156631. doi: 10.1371/journal.pone.0156631 (PMC4905681; doi:10.1371/journal.pone.0156631)
Supplement: S1 File — (GZ) [file pone.0156631.s003.gz › PONE_Brillante_et_al_2016_Code_and_Data/packrat/src/R6/R6_2.0.1.tar.gz!/R6/inst/doc/Portable.html]

Portable and non-portable R6 classes


# Portable and non-portable R6 classes

- The problem
- Portable R6
- Using `self`
- Wrap-up

One limitation to R’s reference classes is that class inheritance across package namespaces is limited. R6 avoids this problem when the `portable` option is enabled.

## The problem

Here is an example of the cross-pacakge inheritance problem with reference classes: Suppose you have ClassA in pkgA, and ClassB in pkgB, which inherits from ClassA. ClassA has a method `foo` which calls a non-exported function `fun` in pkgA.

If ClassB inherits `foo`, it will try to call `fun` – but since ClassB objects are created in pkgB namespace (which is an environment) instead of the pkgA namespace, it won’t be able to find `fun`.

Something similar happens with R6 when the `portable=FALSE` option is used. For example:

```
library(R6)
# Simulate packages by creating environments
pkgA <- new.env()
pkgB <- new.env()

# Create a function in pkgA but not pkgB
pkgA$fun <- function() 10

ClassA <- R6Class("ClassA",
  portable = FALSE,
  public = list(
    foo = function() fun()
  ),
  parent_env = pkgA
)

# ClassB inherits from ClassA
ClassB <- R6Class("ClassB",
  portable = FALSE,
  inherit = ClassA,
  parent_env = pkgB
)
```

When we create an instance of ClassA, it works as expected:

```
a <- ClassA$new()
a$foo()
#> [1] 10
```

But with ClassB, it can’t find the `foo` function:

```
b <- ClassB$new()
b$foo()
#> Error in b$foo() : could not find function "fun"
```

## Portable R6

R6 supports inheritance across different packages, with the default `portable=TRUE` option. In this example, we’ll again simulate different packages by creating separate parent environments for the classes.

```
pkgA <- new.env()
pkgB <- new.env()

pkgA$fun <- function() {
  "This function `fun` in pkgA"
}

ClassA <- R6Class("ClassA",
  portable = TRUE,  # The default
  public = list(
    foo = function() fun()
  ),
  parent_env = pkgA
)

ClassB <- R6Class("ClassB",
  portable = TRUE,
  inherit = ClassA,
  parent_env = pkgB
)


a <- ClassA$new()
a$foo()
#> [1] "This function `fun` in pkgA"

b <- ClassB$new()
b$foo()
#> [1] "This function `fun` in pkgA"
```

When a method is inherited from a superclass, that method also gets that class’s environment. In other words, method “runs in” the superclass’s environment. This makes it possible for inheritance to work across packages.

When a method is defined in the subclass, that method gets the subclass’s environment. For example, here ClassC is a subclass of ClassA, and defines its own `foo` method which overrides the `foo` method from ClassA. It happens that the method looks the same as ClassA’s – it just calls `fun`. But this time it finds `pkgC$fun` instead of `pkgA$fun`. This is in contrast to ClassB, which inherited the `foo` method and environment from ClassA.

```
pkgC <- new.env()
pkgC$fun <- function() {
  "This function `fun` in pkgC"
}

ClassC <- R6Class("ClassC",
  portable = TRUE,
  inherit = ClassA,
  public = list(
    foo = function() fun()
  ),
  parent_env = pkgC
)

cc <- ClassC$new()
# This method is defined in ClassC, so finds pkgC$fun
cc$foo()
#> [1] "This function `fun` in pkgC"
```

## Using `self`

One important difference between non-portable and portable classes is that with non-portable classes, it’s possible to access members with just the name of the member, and with portable classes, member access always requires using `self$` or `private$`. This is a consequence of the inheritance implementation.

Here’s an example of a non-portable class with two methods: `sety`, which sets the private field `y` using the `<<-` operator, and `getxy`, which returns a vector with the values of fields `x` and `y`:

```
NP <- R6Class("NP",
  portable = FALSE,
  public = list(
    x = 1,
    getxy = function() c(x, y),
    sety = function(value) y <<- value
  ),
  private = list(
    y = NA
  )
)

np <- NP$new()

np$sety(20)
np$getxy()
#> [1]  1 20
```

If we attempt the same with a portable class, it results in an error:

```
P <- R6Class("P",
  portable = TRUE,
  public = list(
    x = 1,
    getxy = function() c(x, y),
    sety = function(value) y <<- value
  ),
  private = list(
    y = NA
  )
)

p <- P$new()

# No error, but instead of setting private$y, this sets y in the global
# environment! This is because of the sematics of <<-.
p$sety(20)
y
#> [1] 20

p$getxy()
#> Error in p$getxy() : object 'y' not found
```

To make this work with a portable class, we need to use `self$x` and `private$y`:

```
P2 <- R6Class("P2",
  portable = TRUE,
  public = list(
    x = 1,
    getxy = function() c(self$x, private$y),
    sety = function(value) private$y <- value
  ),
  private = list(
    y = NA
  )
)

p2 <- P2$new()
p2$sety(20)
p2$getxy()
#> [1]  1 20
```

There is a small performance penalty for using `self$x` as opposed to `x`. In most cases, this is negligible, but it can be noticeable in some situations where there are tens of thousands or more accesses per second. For more information, see the Performance vignette.

## Wrap-up

In summary:

- Portable classes allow inheritance across different packages.
- Portable classes always require the use of `self` or `private` to access members. This can incur a small performance penalty, since using `self$x` is slower than just `x`.
