# Supplementary material for: Comparing Kaolin and Pinolene to Improve Sustainable Grapevine Production during Drought
Source: PLoS One. 2016 Jun 13;11(6):e0156631. doi: 10.1371/journal.pone.0156631 (PMC4905681; doi:10.1371/journal.pone.0156631)
Supplement: S1 File — (GZ) [file pone.0156631.s003.gz › PONE_Brillante_et_al_2016_Code_and_Data/packrat/src/R6/R6_2.0.1.tar.gz!/R6/inst/doc/Introduction.html]

Introduction to R6 classes


# Introduction to R6 classes

- R6 classes
  - Basics
  - Private members
  - Active bindings
  - Inheritance
  - Fields containing reference objects
- Portable and non-portable classes
  - Using `self` and `<<-`
- Other topics
  - Adding members to an existing class
  - Printing R6 objects to the screen
  - Finalizers
- Summary

The R6 package provides a type of class which is similar to R’s standard reference classes, but it is more efficient and doesn’t depend on S4 classes and the methods package.

## R6 classes

R6 classes are similar to R’s standard reference classes, but are lighter weight, and avoid some issues that come along with using S4 classes (R’s reference classes are based on S4). For more information about speed and memory footprint, see the Performance vignette.

Unlike many objects in R, instances (objects) of R6 classes have reference semantics. R6 classes also support:

- public and private methods
- active bindings
- inheritance (superclasses) which works across packages

Why the name R6? When R’s reference classes were introduced, some users, following the names of R’s existing class systems S3 and S4, called the new class system R5 in jest. Although reference classes are not actually called R5, the name of this package and its classes takes inspiration from that name.

The name R5 was also a code-name used for a different object system started by Simon Urbanek, meant to solve some issues with S4 relating to syntax and performance. However, the R5 branch was shelved after a little development, and it was never released.

### Basics

Here’s how to create a simple R6 class. The `public` argument is a list of items, which can be functions and fields (non-functions). Functions will be used as methods.

```
library(R6)

Person <- R6Class("Person",
  public = list(
    name = NA,
    hair = NA,
    initialize = function(name, hair) {
      if (!missing(name)) self$name <- name
      if (!missing(hair)) self$hair <- hair
      self$greet()
    },
    set_hair = function(val) {
      self$hair <- val
    },
    greet = function() {
      cat(paste0("Hello, my name is ", self$name, ".\n"))
    }
  )
)
```

To instantiate an object of this class, use `$new()`:

```
ann <- Person$new("Ann", "black")
#> Hello, my name is Ann.
ann
#> <Person>
#>   Public:
#>     greet: function
#>     hair: black
#>     initialize: function
#>     name: Ann
#>     set_hair: function
```

The `$new()` method creates the object and calls the `initialize()` method, if it exists.

Inside methods of the class, `self` refers to the object. Public members of the object (all you’ve seen so far) are accessed with `self$x`, and assignment is done with `self$x <- y`. Note that by default, `self` is required to access members, although for non-portable classes which we’ll see later, it is optional.

Once the object is instantiated, you can access values and methods with `$`:

```
ann$hair
#> [1] "black"
ann$greet()
#> Hello, my name is Ann.
ann$set_hair("red")
ann$hair
#> [1] "red"
```

Implementation note: The R6 object is basically an environment with the public members in it. The `self` object is bound in that environment, and is simply a reference back to that environment.

### Private members

In the previous example, all the members were public. It’s also possible to add private members:

```
Queue <- R6Class("Queue",
  public = list(
    initialize = function(...) {
      for (item in list(...)) {
        self$add(item)
      }
    },
    add = function(x) {
      private$queue <- c(private$queue, list(x))
      invisible(self)
    },
    remove = function() {
      if (private$length() == 0) return(NULL)
      # Can use private$queue for explicit access
      head <- private$queue[[1]]
      private$queue <- private$queue[-1]
      head
    }
  ),
  private = list(
    queue = list(),
    length = function() base::length(private$queue)
  )
)

q <- Queue$new(5, 6, "foo")
```

Whereas public members are accessed with `self`, like `self$add()`, private members are accessed with `private`, like `private$queue`.

The public members can be accessed as usual:

```
# Add and remove items
q$add("something")
q$add("another thing")
q$add(17)
q$remove()
#> [1] 5
q$remove()
#> [1] 6
```

However, private members can’t be accessed directly:

```
q$queue
#> NULL
q$length()
#> Error: attempt to apply non-function
```

Unless there is a compelling reason otherwise, methods should return `self` (invisibly) because it makes them chainable. For example, the `add()` method returns `self` so you can chain them together:

```
q$add(10)$add(11)$add(12)
```

On the other hand, `remove()` returns the value removed, so it’s not chainable:

```
q$remove()
#> [1] "foo"
q$remove()
#> [1] "something"
q$remove()
#> [1] "another thing"
q$remove()
#> [1] 17
```

Implementation note: When private members are used, the public environment is a child of the private environment, and the `private` object points to the private environment. Although public and private methods are bound (that is, they can be found) in their respective environments, the *enclosing* environment for all of those methods is the public environment. This means that private methods “run in” the public environment, so they will find public objects without needing an explicit `self$xx`.

### Active bindings

Active bindings look like fields, but each time they are accessed, they call a function. They are always publicly visible.

```
Numbers <- R6Class("Numbers",
  public = list(
    x = 100
  ),
  active = list(
    x2 = function(value) {
      if (missing(value)) return(self$x * 2)
      else self$x <- value/2
    },
    rand = function() rnorm(1)
  )
)

n <- Numbers$new()
n$x
#> [1] 100
```

When an active binding is accessed as if reading a value, it calls the function with `value` as a missing argument:

```
n$x2
#> [1] 200
```

When it’s accessed as if assigning a value, it uses the assignment value as the `value` argument:

```
n$x2 <- 1000
n$x
#> [1] 500
```

If the function takes no arguments, it’s not possible to use it with `<-`:

```
n$rand
#> [1] 0.2648
n$rand
#> [1] 2.171
n$rand <- 3
#> Error: unused argument (quote(3))
```

Implementation note: Active bindings are bound in the public environment. The enclosing environment for these functions is also the public environment.

### Inheritance

One R6 class can inherit from another. In other words, you can have super- and sub-classes.

Subclasses can have additional methods, and they can also have methods that override the superclass methods. In this example of a queue that retains its history, we’ll add a `show()` method and override the `remove()` method:

```
# Note that this isn't very efficient - it's just for illustrating inheritance.
HistoryQueue <- R6Class("HistoryQueue",
  inherit = Queue,
  public = list(
    show = function() {
      cat("Next item is at index", private$head_idx + 1, "\n")
      for (i in seq_along(private$queue)) {
        cat(i, ": ", private$queue[[i]], "\n", sep = "")
      }
    },
    remove = function() {
      if (private$length() - private$head_idx == 0) return(NULL)
      private$head_idx <<- private$head_idx + 1
      private$queue[[private$head_idx]]
    }
  ),
  private = list(
    head_idx = 0
  )
)

hq <- HistoryQueue$new(5, 6, "foo")
hq$show()
#> Next item is at index 1 
#> 1: 5
#> 2: 6
#> 3: foo
hq$remove()
#> [1] 5
hq$show()
#> Next item is at index 2 
#> 1: 5
#> 2: 6
#> 3: foo
hq$remove()
#> [1] 6
```

Superclass methods can be called with `super$xx()`. The `CountingQueue` (example below) keeps a count of the total number of objects that have ever been added to the queue. It does this by overriding the `add()` method – it increments a counter and then calls the superclass’s `add()` method, with `super$add(x)`:

```
CountingQueue <- R6Class("CountingQueue",
  inherit = Queue,
  public = list(
    add = function(x) {
      private$total <<- private$total + 1
      super$add(x)
    },
    get_total = function() private$total
  ),
  private = list(
    total = 0
  )
)

cq <- CountingQueue$new("x", "y")
cq$get_total()
#> [1] 2
cq$add("z")
cq$remove()
#> [1] "x"
cq$remove()
#> [1] "y"
cq$get_total()
#> [1] 3
```

### Fields containing reference objects

If your R6 class contains any fields that also have reference semantics (e.g., other R6 objects, and environments), those fields should be populated in the `initialize` method. If the field set to the reference object directly in the class definition, that object will be shared across all instances of the R6 objects. Here’s an example:

```
SimpleClass <- R6Class("SimpleClass",
  public = list(x = NULL)
)

SharedField <- R6Class("SharedField",
  public = list(
    e = SimpleClass$new()
  )
)

s1 <- SharedField$new()
s1$e$x <- 1

s2 <- SharedField$new()
s2$e$x <- 2

# Changing s2$e$x has changed the value of s1$e$x
s1$e$x
#> [1] 2
```

To avoid this, populate the field in the `initialize` method:

```
NonSharedField <- R6Class("NonSharedField",
  public = list(
    e = NULL,
    initialize = function() e <<- SimpleClass$new()
  )
)

n1 <- NonSharedField$new()
n1$e$x <- 1

n2 <- NonSharedField$new()
n2$e$x <- 2

# n2$e$x does not affect n1$e$x
n1$e$x
#> [1] 1
```

## Portable and non-portable classes

In R6 version 1.0.1, the default was to create **non-portable** classes. In subsequent versions, the default is to create **portable** classes. The two most noticeable differences are that portable classes:

- Support inheritance across different packages. Non-portable classes do not do this very well.
- Always require the use of `self` and `private` to access members, as in `self$x` and `private$y`. Non-portable classes can access these members with just `x` and `y`, and do assignment to these members with the `<<-` operator.

The implementation of the first point is such that it makes the second point necessary.

### Using `self` and `<<-`

With reference classes, you can access the field without `self`, and assign to fields using `<<-`. For example:

```
RC <- setRefClass("RC",
  fields = list(x = 'ANY'),
  methods = list(
    getx = function() x,
    setx = function(value) x <<- value
  )
)

rc <- RC$new()
rc$setx(10)
rc$getx()
#> [1] 10
```

The same is true for non-portable R6 classes:

```
NP <- R6Class("NP",
  portable = FALSE,
  public = list(
    x = NA,
    getx = function() x,
    setx = function(value) x <<- value
  )
)

np <- NP$new()
np$setx(10)
np$getx()
#> [1] 10
```

But for portable R6 classes (this is the default), you must use `self` and/or `private`, and `<<-` assignment doesn’t work – unless you use `self`, of course:

```
P <- R6Class("P",
  portable = TRUE,  # This is default
  public = list(
    x = NA,
    getx = function() self$x,
    setx = function(value) self$x <- value
  )
)

p <- P$new()
p$setx(10)
p$getx()
#> [1] 10
```

For more information, see the Portable vignette.

## Other topics

### Adding members to an existing class

It is sometimes useful to add members to a class after the class has already been created. This can be done using the `$set()` method on the generator object.

```
Simple <- R6Class("Simple",
  public = list(
    x = 1,
    getx = function() x
  )
)

Simple$set("public", "getx2", function() self$x*2)

# To replace an existing member, use overwrite=TRUE
Simple$set("public", "x", 10, overwrite = TRUE)

s <- Simple$new()
s$x
#> [1] 10
s$getx2()
#> [1] 20
```

The new members will be present only in instances that are created after `$set()` has been called.

### Printing R6 objects to the screen

R6 objects have a default `print` method that lists all members of the object. If a class defines a `print` method, then it overrides the default one.

```
PrettyCountingQueue <- R6Class("PrettyCountingQueue",
  inherit = CountingQueue,
  public = list(
    print = function(...) {
      cat("<PrettyCountingQueue> of ", self$get_total(), " elements\n", sep = "")
      invisible(self)
    }
  )
)
```

```
pq <- PrettyCountingQueue$new(1, 2, "foobar")
pq
#> <PrettyCountingQueue> of 3 elements
```

### Finalizers

Sometimes it’s useful to run a function when the object is garbage collected. For example, you may want to make sure a file or database connection gets closed. To do this, you can call the `reg.finalizer()` function in your `initialize` method, and pass it `self` as the object that will trigger the function when it is garbage collected.

```
A <- R6Class("A", public = list(
  initialize = function() {
    reg.finalizer(self,
                  function(e) print("Finalizer has been called!"),
                  onexit = TRUE)
  }
))


# Instantiate an object, but don't save a reference to it
A$new()
#> <A>
#>   Public:
#>     initialize: function

# Run something else to get rid of any references to the object, because the
# last returned value is saved in .Last.value
1+1
#> [1] 2

# Force immediate garbage collection (normally this will happen automatically
# from time to time)
gc()
#>          used (Mb) gc trigger (Mb) max used (Mb)
#> Ncells 336745 18.0     531268 28.4   467875 25.0
#> Vcells 591747  4.6    1162592  8.9   858809  6.6
```

In the example above, we used `onexit=TRUE`, so that the finalizer will alsobe called when R exits. This is useful in some cases, like database connections, but it isn’t necessary for others, like open files, since they will be closed anyway when the R process exits.

## Summary

R6 classes provide capabilities that are common in other object-oriented programming languages. They’re similar to R’s built-in reference classes, but are simpler, smaller, and faster, and they allow inheritance across packages.
