# Supplementary material for: Comparing Kaolin and Pinolene to Improve Sustainable Grapevine Production during Drought
Source: PLoS One. 2016 Jun 13;11(6):e0156631. doi: 10.1371/journal.pone.0156631 (PMC4905681; doi:10.1371/journal.pone.0156631)
Supplement: S1 File — (GZ) [file pone.0156631.s003.gz › PONE_Brillante_et_al_2016_Code_and_Data/packrat/src/R6/R6_2.0.1.tar.gz!/R6/inst/doc/Performance.html]

R6 and Reference class performance tests


# R6 and Reference class performance tests

- Class definitions
  - R reference class
  - R6 class
  - R6 class, without class attribute
  - R6 class, non-portable
  - R6 class, without class attribute, and non-portable
  - R6 class, with public and private members
  - R6 class, with public and private members, no class attribute, and non-portable
  - Environment created by a closure, with class attribute
  - Environment created by a closure, without class attribute
- Tests
  - Memory footprint
  - Object instantiation speed
  - Field access speed
  - Field setting speed
  - Speed of method call that accesses a field
  - Assignment using `self$x <-` vs. `x <<-`
  - Overhead from using `$` on objects with a class attribute
  - Lists vs. environments, and `$` vs. `[[`
- Wrap-up
- Appendix
  - Functions for calculating object sizes
  - System information

This document compares the memory costs and speed of R’s reference classes against R6 classes and simple environments. For must uses, R6 and reference classes have comparable features, but as we’ll see, R6 classes are faster and lighter weight.

This document tests reference classes against R6 classes (in many variations), as well as against very simple reference objects: environments created by closures.

---

First we’ll load some packages which will be used below:

```
library(microbenchmark)
options(microbenchmark.unit = "us")
library(pryr)  # For object_size function
library(R6)
```

---

# Class definitions

We’ll start by defining a number of classes or class-like entities, using reference classes, R6 classes, and simple environments that are created directly by functions. These classes will be used for the speed and memory tests that follow. This is a lot of boring code, so you may want to skip ahead to the results.

All of these classes have the same basic characteristics:

- A field named `x` that contains a number.
- An way of initializing the value of `x`.
- A method named `getx` for retrieving the value of `x`.
- A method named `inc` for incrementing the value of `x`.

The fields and methods are accessed with the `$` operator, so if we have an object named `obj`, we could use `obj$x` or `obj$getx()`.

## R reference class

```
RC <- setRefClass("RC", 
  fields = list(x = "numeric"),
  methods = list(
    initialize = function(x = 1) .self$x <- x,
    getx = function() x,
    inc = function(n = 1) x <<- x + n
  )
)
```

In reference classes, the binding that points back to the object is named `.self`. Within a method, assignment can be done by using `.self`, as in `.self$x <- 10`, or by using `<<-`, as in `x <<- 10`.

To create an object, simply call `$new()` on the class:

```
RC$new()
#> Reference class object of class "RC"
#> Field "x":
#> [1] 1
```

## R6 class

Creating an R6 class is similar to the reference class, except that there’s no need to separate the fields and methods, and you can’t specify the types of the fields.

```
R6 <- R6Class("R6",
  public = list(
    x = NULL,
    initialize = function(x = 1) self$x <- x,
    getx = function() self$x,
    inc = function(n = 1) self$x <- x + n
  )
)
```

Whereas reference classes use `.self`, R6 classes use `self` (without the leading period). As with reference classes, objects are instantiated by calling `$new()`:

```
R6$new()
#> <R6>
#>   Public:
#>     getx: function
#>     inc: function
#>     initialize: function
#>     x: 1
```

An R6 object essentially just an environment. The methods for an R6 object have bindings (that is, they have names) in that environment, and also have that environment as their enclosing environment (they “run in” that environment).

## R6 class, without class attribute

By default, a class attribute is added to R6 objects. This attribute adds a slight performance penalty because R will attempt to use S3 dispatch when using `$` on the object.

It’s possible generate objects without the class attribute, by using `class=FALSE`:

```
R6NoClass <- R6Class("R6NoClass",
  class = FALSE,
  public = list(
    x = NULL,
    initialize = function(x = 1) self$x <- x,
    getx = function() self$x,
    inc = function(n = 1) self$x <- self$x + n
  )
)
```

Note that without the class attribute, S3 method dispatch on the objects is not possible.

## R6 class, non-portable

By default, R6 objects are *portable*. This means that inheritance can be in classes that are in different packages. However, it also requires the use of `self$` and `private$` to access members, and this incurs a small performance penalty.

If `portable=FALSE` is used, members can be accessed without using `self$`, and assignment can be done with `<<-`:

```
R6NonPortable <- R6Class("R6NonPortable",
  portable = FALSE,
  public = list(
    x = NULL,
    initialize = function(value = 1) x <<- value,
    getx = function() x,
    inc = function(n = 1) x <<- x + n
  )
)
```

## R6 class, without class attribute, and non-portable

For comparison, we’ll use a an R6 class without a class attribute, and which is non-portable.

```
R6NoClassNonPortable <- R6Class("R6NoClassNonPortable",
  portable = FALSE,
  class = FALSE,
  public = list(
    x = NULL,
    initialize = function(value = 1) x <<- value,
    getx = function() x,
    inc = function(n = 1) x <<- x + n
  )
)
```

## R6 class, with public and private members

This is a variant of the previous type of reference class, but this version has public and private members.

```
R6Private <- R6Class("R6Private",
  private = list(x = NULL),
  public = list(
    initialize = function(x = 1) private$x <- x,
    getx = function() private$x,
    inc = function(n = 1) private$x <- private$x + n
  )
)
```

Instead of a single `self` object which refers to all items in an object, these objects have `self` (which refers to the public items) and `private`.

```
R6Private$new()
#> <R6Private>
#>   Public:
#>     getx: function
#>     inc: function
#>     initialize: function
```

## R6 class, with public and private members, no class attribute, and non-portable

For comparison, we’ll add a version without a class attribute, and which is non-portable.

```
R6PrivateNcNp <- R6Class("R6PrivateNcNp",
  portable = FALSE,
  class = FALSE,
  private = list(x = NULL),
  public = list(
    initialize = function(x = 1) private$x <- x,
    getx = function() x,
    inc = function(n = 1) x <<- x + n
  )
)
```

## Environment created by a closure, with class attribute

In R, environments are passed by reference. A simple way to create an object that’s passed by reference is to use the environment created by the invocation of a function. The function below captures that environment, attaches a class to it, and returns it:

```
ClosureEnvClass <- function(x = 1) {
  inc <- function(n = 1) x <<- x + n
  getx <- function() x
  self <- environment()
  class(self) <- "ClosureEnvClass"
  self
}
```

Even though `x` isn’t declared in the function body, it gets captured because it’s an argument to the function.

```
ls(ClosureEnvClass())
#> [1] "getx" "inc"  "self" "x"
```

Objects created this way are very similar to those created by `R6` generator we created above.

## Environment created by a closure, without class attribute

We can make an even simpler type of reference object to the previous one, by not having a a class attribute, and not having `self` object:

```
ClosureEnvNoClass <- function(x = 1) {
  inc <- function(n = 1) x <<- x + n
  getx <- function() x
  environment()
}
```

This is simply an environment with some objects in it.

```
ls(ClosureEnvNoClass())
#> [1] "getx" "inc"  "x"
```

---

# Tests

For all the timings using `microbenchmark()`, the results are reported in microseconds, and the most useful value is probably the median column.

## Memory footprint

How much memory does a single instance of each object take, and how much memory does each additional object take? We’ll use the functions `obj_size` and `obj_sizes` (shown at the bottom of this document) to calculate the sizes.

Sizes of each type of object, in bytes:

```
sizes <- obj_sizes(
  RC$new(),
  R6$new(),
  R6NoClass$new(),
  R6NonPortable$new(),
  R6NoClassNonPortable$new(),
  R6Private$new(),
  R6PrivateNcNp$new(),
  ClosureEnvClass(),
  ClosureEnvNoClass()
)
sizes
#>                               one incremental
#> RC$new()                   472072        1368
#> R6$new()                    12624         840
#> R6NoClass$new()             13328         728
#> R6NonPortable$new()         12160         784
#> R6NoClassNonPortable$new()  12408         672
#> R6Private$new()             13584         952
#> R6PrivateNcNp$new()         13344         784
#> ClosureEnvClass()           10696         624
#> ClosureEnvNoClass()          9288         512
```

The results are plotted below. Note that the plots have very different x scales.

It looks like using a reference class takes up a huge amount of memory, but much of that is shared between reference classes. Adding an object from a different reference class doesn’t require much more memory — around 38KB:

```
RC2 <- setRefClass("RC2",
  fields = list(x = "numeric"),
  methods = list(
    initialize = function(x = 2) .self$x <<- x,
    inc = function(n = 2) x <<- x * n
  )
)

# Calcualte the size of a new RC2 object, over and above an RC object
as.numeric(object_size(RC$new(), RC2$new()) - object_size(RC$new()))
#> [1] 37752
```

## Object instantiation speed

How much time does it take to create one of these objects? This shows the median time, in microseconds:

```
# Function to extract the medians from microbenchmark results
mb_summary <- function(x) {
  res <- summary(x, unit="us")
  data.frame(name = res$expr, median = res$median)
}

speed <- microbenchmark(
  RC$new(),
  R6$new(),
  R6NoClass$new(),
  R6NonPortable$new(),
  R6NoClassNonPortable$new(),
  R6Private$new(),
  R6PrivateNcNp$new(),
  ClosureEnvClass(),
  ClosureEnvNoClass()
)
speed <- mb_summary(speed)
speed
#>                         name   median
#> 1                   RC$new() 377.0455
#> 2                   R6$new()  56.9860
#> 3            R6NoClass$new()  46.4555
#> 4        R6NonPortable$new()  54.4795
#> 5 R6NoClassNonPortable$new()  43.3780
#> 6            R6Private$new()  76.7895
#> 7        R6PrivateNcNp$new()  64.9745
#> 8          ClosureEnvClass()   3.7705
#> 9        ClosureEnvNoClass()   1.7730
```

The plot below shows the median instantiation time.

R reference classes are much slower to instantiate than the other types of classes. Instantiating R6 objects is roughly 10 times faster. Creating an environment with a simple closure is another 10-20 times faster still.

## Field access speed

How much time does it take to access a field in an object? First we’ll make some objects:

```
rc           <- RC$new()
r6           <- R6$new()
r6nc         <- R6NoClass$new()
r6np         <- R6NonPortable$new()
r6nc_np      <- R6NoClassNonPortable$new()
r6priv       <- R6Private$new()
r6priv_nc_np <- R6PrivateNcNp$new()
closure      <- ClosureEnvClass()
closure_nc   <- ClosureEnvNoClass()
```

And then get a value from these objects:

```
speed <- microbenchmark(
  rc$x,
  r6$x,
  r6nc$x,
  r6np$x,
  r6nc_np$x,
  r6priv$x,
  r6priv_nc_np$x,
  closure$x,
  closure_nc$x
)
speed <- mb_summary(speed)
speed
#>             name  median
#> 1           rc$x 12.8565
#> 2           r6$x  2.8815
#> 3         r6nc$x  0.3295
#> 4         r6np$x  3.1125
#> 5      r6nc_np$x  0.3260
#> 6       r6priv$x  3.1055
#> 7 r6priv_nc_np$x  0.3385
#> 8      closure$x  2.4540
#> 9   closure_nc$x  0.3485
```

Accessing the field of a reference class is much slower than the other methods.

There’s also an obvious pattern where accessing the field of an environment (created by R6 or a closure) is slower when there is a class attribute. This is because, for the objects that have a class attribute, R attempts to look up an S3 method for `$`, and this lookup has a performance penalty, as we’ll see below.

## Field setting speed

How much time does it take to set the value of a field in an object?

```
speed <- microbenchmark(
  rc$x <- 4,
  r6$x <- 4,
  r6nc$x <- 4,
  r6np$x <- 4,
  r6nc_np$x <- 4,
  # r6priv$x <- 4,         # Can't set private field directly,
  # r6priv_nc_np$x <- 4,   # so we'll skip these two
  closure$x <- 4,
  closure_nc$x <- 4
)
speed <- mb_summary(speed)
speed
#>                name  median
#> 1         rc$x <- 4 55.7430
#> 2         r6$x <- 4  3.5055
#> 3       r6nc$x <- 4  0.9755
#> 4       r6np$x <- 4  3.8320
#> 5    r6nc_np$x <- 4  0.9925
#> 6    closure$x <- 4  3.1355
#> 7 closure_nc$x <- 4  0.9535
```

Reference classes are significantly slower than the others, again. In this case, there’s additional overhead due to type-checking the value.

Once more, the no-class objects are significantly faster than the others, again probably due to attempted S3 dispatch on the `` `$<-` `` function.

## Speed of method call that accesses a field

How much overhead is there when calling a method from one of these objects? All of these `getx()` methods simply return the value of `x` in the object. When necessary, this method uses `self$x` (for R6 classes, when `portable=TRUE`), and in others, it just uses `x` (when `portable=FALSE`, and in reference classes).

```
speed <- microbenchmark(
  rc$getx(),
  r6$getx(),
  r6nc$getx(),
  r6np$getx(),
  r6nc_np$getx(),
  r6priv$getx(),
  r6priv_nc_np$getx(),
  closure$getx(),
  closure_nc$getx()
)
speed <- mb_summary(speed)
speed
#>                  name  median
#> 1           rc$getx() 21.6015
#> 2           r6$getx()  9.8505
#> 3         r6nc$getx()  1.4525
#> 4         r6np$getx()  5.5735
#> 5      r6nc_np$getx()  0.9170
#> 6       r6priv$getx()  5.9810
#> 7 r6priv_nc_np$getx()  0.9830
#> 8      closure$getx()  4.4595
#> 9   closure_nc$getx()  0.9415
```

The reference class is the slowest.

`r6` is also somewhat slower than the others. There are two reasons for this: first, it uses `self$x` which adds some time, and second, it has a class attribute, which slows down the access of both `r6$getx` and `self$x`.

One might expect `r6priv` to be the same speed as `r6`, but it is faster. Although accessing `r6priv$getx` is slow, because `r6priv` has a class attribute, accessing `private$x` is faster, because it does not have a class attribute.

The objects which can access `x` directly (without `self` or `private`) and which lack a class attribute are the fastest.

## Assignment using `self$x <-` vs. `x <<-`

With reference class objects, you can modify fields using the `<<-` operator, or by using the `.self` object. For example, compare the `setx()` methods of these two classes:

```
RCself <- setRefClass("RCself",
  fields = list(x = "numeric"),
  methods = list(
    initialize = function() .self$x <- 1,
    setx = function(n = 2) .self$x <- n
  )
)

RCnoself <- setRefClass("RCnoself",
  fields = list(x = "numeric"),
  methods = list(
    initialize = function() x <<- 1,
    setx = function(n = 2) x <<- n
  )
)
```

Non-portable R6 classes are similar, except they use `self` instead of `.self`:

```
R6self <- R6Class("R6self",
  portable = FALSE,
  public = list(
    x = 1,
    setx = function(n = 2) self$x <- n
  )
)

R6noself <- R6Class("R6noself",
  portable = FALSE,
  public = list(
    x = 1,
    setx = function(n = 2) x <<- n
  )
)
```

```
rc_self   <- RCself$new()
rc_noself <- RCnoself$new()
r6_self   <- R6self$new()
r6_noself <- R6noself$new()

speed <- microbenchmark(
  rc_self$setx(),
  rc_noself$setx(),
  r6_self$setx(),
  r6_noself$setx()
)
speed <- mb_summary(speed)
speed
#>               name  median
#> 1   rc_self$setx() 64.4125
#> 2 rc_noself$setx() 40.2150
#> 3   r6_self$setx()  8.3895
#> 4 r6_noself$setx()  3.7210
```

For both reference and non-portable R6 classes, assignment using `.self$x <-` is somewhat slower than using `x <<-`.

Bear in mind that, by default, R6 classes are portable, and can’t use assignment with `x <<-`.

## Overhead from using `$` on objects with a class attribute

There is some overhead when using `$` on an object that has a class attribute. In the test below, we’ll create three different kinds of objects:

1. An environment with no class attribute.
2. An environment with a class `"e2"`, but without a `$.e2` S3 method.
3. An environment with a class `"e3"`, which has a `$.e3` S3 method that simply returns `NULL`.

Each one of these environments will contain an object `x`.

```
e1 <- new.env(hash = FALSE, parent = emptyenv())
e2 <- new.env(hash = FALSE, parent = emptyenv())
e3 <- new.env(hash = FALSE, parent = emptyenv())

e1$x <- 1
e2$x <- 1
e3$x <- 1

class(e2) <- "e2"
class(e3) <- "e3"

# Define an S3 method for class e3
`$.e3` <- function(x, name) {
  NULL
}
```

Now we can run timing tests for calling `$` on each type of object. Note that for the `e3` object, the `$` function does nothing — it simply returns `NULL`.

```
speed <- microbenchmark(
  e1$x,
  e2$x,
  e3$x
)
speed <- mb_summary(speed)
speed
#>   name median
#> 1 e1$x  0.267
#> 2 e2$x  2.051
#> 3 e3$x  1.613
```

Using `$` on `e2` and `e3` is much slower than on `e1`. This is because `e2` and `e3` have a class attribute. Even though there’s no `$` method defined for `e2`, doing `e2$x` still about 6 times slower than `e1$x`, simply because R looks for an appropriate S3 method.

`e3$x` is slightly faster than `e2$x`; this is probably because the `$.e3` function doesn’t actually do anything other than return NULL.

If an object has a class attribute, R will attempt to look for a method every time `$` is called. This can slow things down considerably, if `$` is used often.

## Lists vs. environments, and `$` vs. `[[`

Lists could also be used for creating classes. How much time does it take to access items using `$` for lists vs. environments? We’ll also compare using `obj$x` to `obj[['x']]`.

```
lst <- list(x = 10)
env <- new.env()
env$x <- 10

mb_summary(microbenchmark(
  lst = lst$x,
  env = env$x,
  lst[['x']],
  env[['x']]
))
#>         name median
#> 1        lst 0.3085
#> 2        env 0.2810
#> 3 lst[["x"]] 0.2195
#> 4 env[["x"]] 0.2105
```

Performance is comparable across environments and lists.

The `[[` operator is slightly faster than `$`, probably because it doesn’t need to convert the unevaluated symbol to a string.

---

# Wrap-up

R6 objects take less memory and are significantly faster than R’s reference class objects, and they also have some options that provide for even more speed.

In these tests, the biggest speedup for R6 classes comes from not using a class attribute; this speeds up the use of `$`. Non-portable R6 classes can also access fields without `$` at all, which provides another modest speed boost. In most cases, these speed increases are negligible – they are on the order of microseconds and will be noticeable only when tens or even hundreds of thousands of class member accesses are performed.

---

# Appendix

## Functions for calculating object sizes

```
# Utility functions for calculating sizes
obj_size <- function(expr, .env = parent.frame()) {
  size_n <- function(n = 1) {
    objs <- lapply(1:n, function(x) eval(expr, .env))
    as.numeric(do.call(object_size, objs))
  }

  data.frame(one = size_n(1), incremental = size_n(2) - size_n(1))
}

obj_sizes <- function(..., .env = parent.frame()) {
  exprs <- as.list(match.call(expand.dots = FALSE)$...)
  names(exprs) <- lapply(1:length(exprs),
    FUN = function(n) {
      name <- names(exprs)[n]
      if (is.null(name) || name == "") paste(deparse(exprs[[n]]), collapse = " ")
      else name
    })

  sizes <- mapply(obj_size, exprs, MoreArgs = list(.env = .env), SIMPLIFY = FALSE)
  do.call(rbind, sizes)
}
```

## System information

```
sessionInfo()
#> R version 3.1.1 (2014-07-10)
#> Platform: x86_64-pc-linux-gnu (64-bit)
#> 
#> locale:
#>  [1] LC_CTYPE=en_US.UTF-8       LC_NUMERIC=C              
#>  [3] LC_TIME=en_US.UTF-8        LC_COLLATE=C              
#>  [5] LC_MONETARY=en_US.UTF-8    LC_MESSAGES=en_US.UTF-8   
#>  [7] LC_PAPER=en_US.UTF-8       LC_NAME=C                 
#>  [9] LC_ADDRESS=C               LC_TELEPHONE=C            
#> [11] LC_MEASUREMENT=en_US.UTF-8 LC_IDENTIFICATION=C       
#> 
#> attached base packages:
#> [1] stats     graphics  grDevices utils     datasets  methods   base     
#> 
#> other attached packages:
#> [1] scales_0.2.4         ggplot2_1.0.0        pryr_0.1            
#> [4] microbenchmark_1.4-2 R6_2.0.1            
#> 
#> loaded via a namespace (and not attached):
#>  [1] MASS_7.3-35      Rcpp_0.11.3      codetools_0.2-9  colorspace_1.2-4
#>  [5] digest_0.6.4     evaluate_0.5.5   formatR_1.0      grid_3.1.1      
#>  [9] gtable_0.1.2     htmltools_0.2.6  knitr_1.7        labeling_0.3    
#> [13] munsell_0.4.2    plyr_1.8.1       proto_0.3-10     reshape2_1.4    
#> [17] rmarkdown_0.3.10 stringr_0.6.2    tools_3.1.1      yaml_2.1.13
```
