# Supplementary material for: Comparing Kaolin and Pinolene to Improve Sustainable Grapevine Production during Drought
Source: PLoS One. 2016 Jun 13;11(6):e0156631. doi: 10.1371/journal.pone.0156631 (PMC4905681; doi:10.1371/journal.pone.0156631)
Supplement: S1 File — (GZ) [file pone.0156631.s003.gz › PONE_Brillante_et_al_2016_Code_and_Data/packrat/src/Rcpp/Rcpp_0.11.6.tar.gz!/Rcpp/inst/doc/Rcpp-unitTests.pdf]

Rcpp: Unit testing results

Dirk Eddelbuettel   Romain François

Rcpp version 0.11.6 as of May 1, 2015

Test Execution

```
The environment variable 'RunAllRcppTests' was not set to 'yes', so skipping some tests.


Executing test function test.binary.testRcppPackage  ...  done successfully.
```

Test Results

```
RUNIT TEST PROTOCOL -- Fri May  1 09:41:52 2015
***********************************************
Number of test functions: 1
Number of errors: 0
Number of failures: 0


1 Test Suite :
Rcpp unit testing - 1 test function, 0 errors, 0 failures



Details
***************************
Test Suite: Rcpp unit testing
Test function regexp: ^test.+
Test file regexp: ^runit.+\.[rR]$
Involved directory:
/tmp/Rtmp022Df2/Rinst22a35f6b57d9/Rcpp/unitTests
---------------------------
Test file: /tmp/Rtmp022Df2/Rinst22a35f6b57d9/Rcpp/unitTests/runit.binary.package.R
test.binary.testRcppPackage: (0 checks) ... OK (0 seconds)
```
