# Supplementary material for: Comparing Kaolin and Pinolene to Improve Sustainable Grapevine Production during Drought
Source: PLoS One. 2016 Jun 13;11(6):e0156631. doi: 10.1371/journal.pone.0156631 (PMC4905681; doi:10.1371/journal.pone.0156631)
Supplement: S1 File — (GZ) [file pone.0156631.s003.gz › PONE_Brillante_et_al_2016_Code_and_Data/packrat/src/Rcpp/Rcpp_0.11.6.tar.gz!/Rcpp/inst/doc/Rcpp-introduction.pdf]

Rcpp: Seamless R and C++ Integration

Dirk Eddelbuettel
Debian Project

Romain François
R Enthusiasts

Abstract

The **Rcpp** package simplifies integrating C++ code with R. It provides a consistent C++ class hierarchy that maps various types of R objects (vectors, matrices, functions, environments, ...) to dedicated C++ classes. Object interchange between R and C++ is managed by simple, flexible and extensible concepts which include broad support for C++ Standard Template Library idioms. C++ code can both be compiled, linked and loaded on the fly, or added via packages. Flexible error and exception code handling is provided. **Rcpp** substantially lowers the barrier for programmers wanting to combine C++ code with R.

Keywords: R, C++, foreign function interface, `.Call`.

1. Introduction

R (R Development Core Team 2013a) is an extensible system. The 'Writing R Extensions' manual (R Development Core Team 2012) describes in detail how to augment R with compiled code, focusing mostly on the C language, but also mentioning C++ and Fortran. The R application programming interface (API) described in 'Writing R Extensions' is based on a set of functions and macros operating on `SEXP` (pointers to `SEXPREC` or 'S expression' structures, see the 'R Language' manual R Development Core Team 2013c for details) which are the internal representation of R objects. In this article, we discuss the functionality of the **Rcpp** package (Eddelbuettel *et al.* 2015), which simplifies the usage of C++ code in R. Combining R and C++ is not a new idea, so we start with a short review of other approaches and give some historical background on the development of **Rcpp**.

The **Rcpp** package provides a consistent API for seamlessly accessing, extending or modifying R objects at the C++ level. The API is a rewritten and extented version of an earlier API which we refer to as the 'classic **Rcpp** API'. It is still provided in the **RcppClassic** package (Eddelbuettel and François 2015) to ensure compatibility, but its use is otherwise deprecated. All new development should use the richer second API which is enclosed in the **Rcpp** C++ namespace, and corresponds to the redesigned code base. This article highlights some of the key design and implementation choices of the new API: Lightweight encapsulation of R objects in C++ classes, automatic garbage collection strategy, code inlining, data interchange between R and C++, and error handling.

Several examples are included to illustrate the benefits of using **Rcpp** as opposed to the traditional R API. Many more examples are available within the package, both as explicit examples and as part of the numerous unit tests. The **Rcpp** package is available from the Comprehensive R Archive Network (CRAN) at `http://CRAN.R-project.org/package=Rcpp`.

This vignette corresponds to the paper published in the *Journal of Statistical Software*. It is currently still identical to the published paper. Over time, this vignette version may receive minor updates. For citations, please use the Eddelbuettel and François (2011) or Eddelbuettel (2013); details are also provided in R via `citation("Rcpp")`.

This version corresponds to **Rcpp** version 0.11.6 and was typeset on May 01, 2015.

1.1. Historical context

Rcpp first appeared in 2005 as a contribution (by Dominick Samperi) to the **RQuantLib** package (Eddelbuettel and Nguyen 2014) and became a CRAN package in early 2006. Several releases (all by Samperi) followed in quick succession under the name **Rcpp**. The package was then renamed to **RcppTemplate**; several more releases followed during 2006 under the new name. However, no further releases were made during 2007, 2008 or most of 2009. Following a few updates in late 2009, it was withdrawn from CRAN.

Given the continued use of the package, Eddelbuettel decided to revitalize it. New releases, using the initial name **Rcpp**, started in November 2008. These included an improved build and distribution process, additional documentation, and new functionality—while retaining the existing 'classic **Rcpp**' interface. While not described here, this API will be provided for the foreseeable future via the **RcppClassic** package.

Reflecting evolving C++ coding standards (see Meyers 2005), Eddelbuettel and François started a significant redesign of the code base in 2009. This added numerous new features several of which are described in this article as well as in multiple vignettes included with the package. This new API is our current focus, and we intend to both extend and support the API in future development of the **Rcpp** package.

1.2. Related work

Integration of C++ and R has been addressed by several authors; the earliest published reference is probably Bates and DebRoy (2001). An unpublished paper by Java *et al.* (2007) expresses several ideas that are close to some of our approaches, though not yet fully fleshed out. The **Rserve** package (Urbanek 2003, 2013) acts as a socket server for R. On the server side, **Rserve** translates R data structures into a binary serialization format and uses TCP/IP for transfer. On the client side, objects are reconstructed as instances of Java or C++ classes that emulate the structure of R objects.

The packages **rcppbind** (Liang 2008), **RAbstraction** (Armstrong 2009a) and **RObjects** (Armstrong 2009b) are all implemented using C++ templates. None of them have matured to the point of a CRAN release. **CXXR** (Runnalls 2009) approaches this topic from the other direction: Its aim is to completely refactor R on a stronger C++ foundation. **CXXR** is therefore concerned with all aspects of the R interpreter, read-eval-print loop (REPL), and threading; object interchange between R and C++ is but one part. A similar approach is discussed by Temple Lang (2009a) who suggests making low-level internals extensible by package developers in order to facilitate extending R. Temple Lang (2009b), using compiler output for references on the code in order to add bindings and wrappers, offers a slightly different angle.

1.3. Rcpp use cases

The core focus of **Rcpp** has always been on helping the programmer to more easily add C++-

based functions. Here, we use 'function' in the standard mathematical sense of providing results (output) given a set of parameters or data (input). This was facilitated from the earliest releases using C++ classes for receiving various types of R objects, converting them to C++ objects and allowing the programmer to return the results to R with relative ease.

This API therefore supports two typical use cases. First, existing R code may be replaced by equivalent C++ code in order to reap performance gains. This case is conceptually easy when there are (built- or run-time) dependencies on other C or C++ libraries. It typically involves setting up data and parameters—the right-hand side components of a function call—before making the call in order to provide the result that is to be assigned to the left-hand side. Second, **Rcpp** facilitates calling functions provided by other libraries. The use resembles the first case but with an additional level of abstraction: data and parameters are passed via **Rcpp** to a function set-up to call code from an external library.

Apart from this 'vertical mode' of calling C++ from R, additional features in the new API also support a more 'horizontal mode' of directly calling **Rcpp** objects. This was motivated by the needs of other projects such as **RInside** (Eddelbuettel and François 2014c) for easy embedding of R in C++ applications and **RProtoBuf** (François *et al.* 2014b) to interface with the Protocol Buffers library. This use will be touched upon in the next section, but a more detailed discussion is outside the scope of this paper. Lastly, the more recent additions '**Rcpp** modules' and '**Rcpp** sugar' also expand the use cases; see Section 9 below.

2. The Rcpp API

2.1. A first example

We can illustrate the **Rcpp** API by revisiting the convolution example from the 'Writing R Extensions' manual (R Development Core Team 2012, Chapter 5). Using **Rcpp**, this function can be written as follows:

```
#include <Rcpp.h>

RcppExport SEXP convolve3cpp(SEXP a, SEXP b) {
    Rcpp::NumericVector xa(a);
    Rcpp::NumericVector xb(b);
    int n_xa = xa.size(), n_xb = xb.size();
    int nab = n_xa + n_xb - 1;
    Rcpp::NumericVector xab(nab);

    for (int i = 0; i < n_xa; i++)
        for (int j = 0; j < n_xb; j++)
            xab[i + j] += xa[i] * xb[j];

    return xab;
}
```

We can highlight several aspects.

1. Only a single header file `Rcpp.h` is needed to use the **Rcpp** API.

2. `RcppExport` is a convenience macro helping with calling a C function from C++.

3. Given two arguments of type `SEXP`, a third is returned (as using only `SEXP` types for input and output is prescribed by the `.Call()` interface of the R API).

4. Both inputs are converted to C++ vector types provided by **Rcpp** (and we have more to say about these conversions below).

5. The usefulness of these classes can be seen when we query the vectors directly for their size—using the `size()` member function—in order to reserve a new result type of appropriate length, and with the use of the `operator[]` to extract and set individual elements of the vector.

6. The computation itself is straightforward embedded looping just as in the original examples in the 'Writing R Extensions' manual (R Development Core Team 2012).

7. The return conversion from the `NumericVector` to the `SEXP` type is also automatic.

We argue that this **Rcpp**-based usage is much easier to read, write and debug than the C macro-based approach supported by R itself.

2.2. Rcpp class hierarchy

The `Rcpp::RObject` class is the basic class of the new **Rcpp** API. An instance of the `RObject` class encapsulates an R object (itself represented by the R type `SEXP`), exposes methods that are appropriate for all types of objects and transparently manages garbage collection.

The most important aspect of the `RObject` class is that it is a very thin wrapper around the `SEXP` it encapsulates. The `SEXP` is indeed the only data member of an `RObject`. The `RObject` class does not interfere with the way R manages its memory and does not perform copies of the object into a suboptimal C++ representation. Instead, it merely acts as a proxy to the object it encapsulates so that methods applied to the `RObject` instance are relayed back to the `SEXP` in terms of the standard R API.

The `RObject` class takes advantage of the explicit life cycle of C++ objects to manage exposure of the underlying R object to the garbage collector. The `RObject` effectively treats its underlying `SEXP` as a resource. The constructor of the `RObject` class takes the necessary measures to guarantee that the underlying `SEXP` is protected from the garbage collector, and the destructor assumes the responsibility to withdraw that protection.

By assuming the entire responsibility of garbage collection, **Rcpp** relieves the programmer from writing boiler plate code to manage the protection stack with `PROTECT` and `UNPROTECT` macros.

The `RObject` class defines a set of member functions applicable to any R object, regardless of its type. This ranges from querying properties of the object (`isNULL`, `isObject`, `isS4`), management of the attributes (`attributeNames`, `hasAttribute`, `attr`) to handling of slots[1] (`hasSlot`, `slot`).

[1] Member functions dealing with slots are only applicable to S4 objects; otherwise an exception is thrown.

2.3. Derived classes

Internally, an R object must have one type amongst the set of predefined types, commonly referred to as SEXP types. The 'R Internals' manual (R Development Core Team 2013b) documents these various types. **Rcpp** associates a dedicated C++ class for most SEXP types, and therefore only exposes functionality that is relevant to the R object that it encapsulates.

For example `Rcpp::Environment` contains member functions to manage objects in the associated environment. Similarly, classes related to vectors—`IntegerVector`, `NumericVector`, `RawVector`, `LogicalVector`, `CharacterVector`, `GenericVector` (also known as `List`) and `ExpressionVector`—expose functionality to extract and set values from the vectors.

The following sections present typical uses of **Rcpp** classes in comparison with the same code expressed using functions and macros of the R API.

2.4. Numeric vectors

The next code snippet is taken from 'Writing R Extensions' (R Development Core Team 2012, Section 5.9.1). It allocates a `numeric` vector of two elements and assigns some values to it using the R API.

```
SEXP ab;
PROTECT(ab = allocVector(REALSXP, 2));
REAL(ab)[0] = 123.45;
REAL(ab)[1] = 67.89;
UNPROTECT(1);
```

Although this is one of the simplest examples in 'Writing R Extensions', it seems verbose and yet it is not obvious at first sight what is happening. Memory is allocated by `allocVector`; we must also supply it with the type of data (`REALSXP`) and the number of elements. Once allocated, the `ab` object must be protected from garbage collection. Lastly, the `REAL` macro returns a pointer to the beginning of the actual array; its indexing does not resemble either R or C++.

The code can be simplified using the `Rcpp::NumericVector` class:

```
Rcpp::NumericVector ab(2);
ab[0] = 123.45;
ab[1] = 67.89;
```

The code contains fewer idiomatic decorations. The `NumericVector` constructor is given the number of elements the vector contains (2), which hides the call to the `allocVector` in the original code example. Also hidden is protection of the object from garbage collection, which is a behavior that `NumericVector` inherits from `RObject`. Values are assigned to the first and second elements of the vector as `NumericVector` overloads the `operator[]`.

The snippet can also be written more concisely as a single statement using the `create` static member function of the `NumericVector` class:

```
Rcpp::NumericVector ab = Rcpp::NumericVector::create(123.45, 67.89);
```

2.5. Character vectors

A second example deals with character vectors and emulates this R code:

```
R> c("foo", "bar")
```

Using the traditional R API, the vector can be allocated and filled as such:

```
SEXP ab;
PROTECT(ab = allocVector(STRSXP, 2));
SET_STRING_ELT( ab, 0, mkChar("foo") );
SET_STRING_ELT( ab, 1, mkChar("bar") );
UNPROTECT(1);
```

This imposes on the programmer knowledge of `PROTECT`, `UNPROTECT`, `SEXP`, `allocVector`, `SET_STRING_ELT`, and `mkChar`. Using the `Rcpp::CharacterVector` class, we can express the same code more concisely:

```
Rcpp::CharacterVector ab(2);
ab[0] = "foo";
ab[1] = "bar";
```

3. R and C++ data interchange

In addition to classes, the **Rcpp** package contains two functions to perform conversion of C++ objects to R objects and back.

3.1. C++ to R: `wrap`

The C++ to R conversion is performed by the `Rcpp::wrap` templated function. It uses advanced template metaprogramming techniques[2] to convert a wide and extensible set of types and classes to the most appropriate type of R object. The signature of the `wrap` template is as follows:

```
template <typename T> SEXP wrap(const T& object);
```

The templated function takes a reference to a 'wrappable' object and converts this object into a `SEXP`, which is what R expects. Currently wrappable types are:

- primitive types: `int`, `double`, `bool`, ... which are converted into the corresponding atomic R vectors;
- `std::string` objects which are converted to R atomic character vectors;
- Standard Template Library (STL) containers such as `std::vector<T>` or `std::map<T>`, as long as the template parameter type `T` is itself wrappable;

[2] A discussion of template metaprogramming (Vandevoorde and Josuttis 2003; Abrahams and Gurtovoy 2004) is beyond the scope of this article.

- STL maps which use `std::string` for keys (e.g., `std::map<std::string, T>`); as long as the type `T` is wrappable;
- any type that implements implicit conversion to `SEXP` through the `operator SEXP()`;
- any type for which the `wrap` template is fully specialized.

Wrappability of an object type is resolved at compile time using modern techniques of template meta programming and class traits. The `Rcpp-extending` vignette in the **Rcpp** package discusses in depth how to extend `wrap` to third-party types. The **RcppArmadillo** (François, Eddelbuettel, and Bates 2014a) and **RcppGSL** (François and Eddelbuettel 2015) packages feature several examples. The following segment of code illustrates that the design allows composition:

```
RcppExport SEXP someFunction() {
  std::vector<std::map<std::string,int> > v;
  std::map<std::string, int> m1;
  std::map<std::string, int> m2;

  m1["foo"]=1;
  m1["bar"]=2;
  m2["foo"]=1;
  m2["bar"]=2;
  m2["baz"]=3;

  v.push_back( m1 );
  v.push_back( m2 );
  return Rcpp::wrap( v );
}
```

In this example, the STL types `vector` and `map` are used to create a list of two named vectors. The member function `push_back` insert a given element into a vector. This example is equivalent to the result of this R statement:

```
list(c(bar = 2L, foo = 1L), c(bar = 2L, baz = 3L, foo = 1L))
```

3.2. R to C++: as

The reverse conversion from an R object to a C++ object is implemented by variations of the `Rcpp::as` template whose signature is:

```
template <typename T> T as(SEXP x);
```

It offers less flexibility and currently handles conversion of R objects into primitive types (e.g., `bool`, `int`, `std::string`, ...), STL vectors of primitive types (e.g., `std::vector<bool>`, `std::vector<double>`, ...) and arbitrary types that offer a constructor that takes a `SEXP`. In addition `as` can be fully or partially specialized to manage conversion of R data structures

to third-party types as can be seen for example in the **RcppArmadillo** package which eases transfer of R matrices and vectors to the optimised data structures in the **Armadillo** linear algebra library (Sanderson 2010).

3.3. Implicit use of converters

The converters offered by `wrap` and `as` provide a very useful framework to implement code logic in terms of C++ data structures and then explicitly convert data back to R.

In addition, the converters are also used implicitly in various places in the `Rcpp` API. Consider the following code that uses the `Rcpp::Environment` class to interchange data between C++ and R. It accesses a vector `x` from the global environment, creates an STL `map` of string types and pushes this back to R:

```
Rcpp::Environment global = Rcpp::Environment::global_env();
std::vector<double> vx = global["x"];

std::map<std::string, std::string> map;
map["foo"] = "oof";
map["bar"] = "rab";

global["y"] = map;
```

In the first part of the example, the code extracts a `std::vector<double>` from the global environment. In order to achieve this, the `operator[]` of `Environment` uses the proxy pattern (Meyers 1995) to distinguish between left hand side (LHS) and right hand side (RHS) use.

The output of the `operator[]` is an instance of the nested class `Environment::Binding`. This class defines a templated implicit conversion operator. It is this conversion operator which allows a `Binding` object to be assigned to any type that `Rcpp::as` is able to handle.

In the last part of the example, the LHS use of the `Binding` instance is implemented through its assignment operator. This is also templated and uses `Rcpp::wrap` to perform the conversion to a `SEXP` that can be assigned to the requested symbol in the global environment.

The same mechanism is used throughout the API. Examples include access/modification of object attributes, slots, elements of generic vectors (lists), function arguments, nodes of dotted pair lists, language calls and more.

4. Function calls

The next example shows how to use **Rcpp** to emulate the R code `rnorm(10L, sd = 100.0)`. As shown in Table 1, the code can be expressed in several ways in either **Rcpp** or the standard R API. The first version shows the use of the `Environment` and `Function` classes by **Rcpp**. The second version shows the use of the `Language` class, which manages calls (LANGSXP). For comparison, we also show both versions using the standard R API. Finally, we also show a variant using '**Rcpp** sugar', a topic which is discussed in Sections 8 and 9 below.

This example illustrates that the **Rcpp** API permits us to work with code that is easier to read, write and maintain. More examples are available as part of the documentation included in the **Rcpp** package, as well as among its over seven hundred and fifty unit tests.

Environment: Using the **Rcpp** API

```
Environment stats("package:stats");
Function rnorm = stats["rnorm"];
return rnorm(10,
             Named("sd", 100.0));
```

Environment: Using the R API

```
SEXP stats = PROTECT(
  R_FindNamespace(
    mkString("stats")));
SEXP rnorm = PROTECT(
  findVarInFrame(stats,
                 install("rnorm")));
SEXP call  = PROTECT(
  LCONS( rnorm,
    CONS(ScalarInteger(10),
      CONS(ScalarReal(100.0),
           R_NilValue))));
SET_TAG(CDDR(call),install("sd"));
SEXP res = PROTECT(eval(call,
                        R_GlobalEnv));
UNPROTECT(4);
return res;
```

Language: Using the **Rcpp** API

```
Language call("rnorm", 10,
              Named("sd",100.0));
return call.eval();
```

Language: Using the R API

```
SEXP call  = PROTECT(
    LCONS(install("rnorm"),
    CONS(ScalarInteger(10),
      CONS(ScalarReal(100.0),
           R_NilValue))));
SET_TAG(CDDR(call),install("sd"));
SEXP res = PROTECT(eval(call,
                        R_GlobalEnv));
UNPROTECT(2);
return res;
```

Sugar: Using the **Rcpp** API

```
RNGScope scope;
return rnorm(10, 0, 100);
```

Sugar: Using the R API

(not applicable)

Table 1: **Rcpp** versus the R API: Five ways of calling `rnorm(10L, sd = 100)` in C/C++.

Note that we have removed the `Rcpp::` prefix for readability; this corresponds to adding a directive `using namespace Rcpp;` in the code. The versions that use callbacks to R do not require handling of the state of the random number generator. The version that uses **Rcpp** sugar requires it, which is done via the instantiation of the `RNGScope` variable.

5. Using code 'inline'

Extending R with compiled code requires a mechanism for reliably compiling, linking, and loading the code. While using a package is preferable in the long run, it may be too involved for quick explorations. An alternative is provided by the **inline** package (Sklyar *et al.* 2013)

which compiles, links and loads a C, C++ or Fortran function—directly from the R prompt using simple functions `cfunction` and `cxxfunction`. The latter provides an extension which works particularly well with **Rcpp** via so-called 'plugins' which provide information about additional header file and library locations.

The use of **inline** is possible as **Rcpp** can be installed and updated just like any other R package using, for examples, the `install.packages()` function for initial installation as well as `update.packages()` for upgrades. So even though R/C++ interfacing would otherwise require source code, the **Rcpp** library is always provided ready for use as a pre-built library through the CRAN package mechanism.[3]

The library and header files provided by **Rcpp** for use by other packages are installed along with the **Rcpp** package. The `LinkingTo: Rcpp` directive in the `DESCRIPTION` file lets R properly reference the header files. The **Rcpp** package provides appropriate information for the `-L` switch needed for linking via the function `Rcpp:::LdFlags()`. It can be used by `Makevars` files of other packages, and **inline** makes use of it internally so that all of this is done behind the scenes without the need for explicitly setting compiler or linker options.

The convolution example provided above can be rewritten for use by **inline** as shown below. The function body is provided by the R character variable `src`, the function header is defined by the argument `signature`, and we only need to enable `plugin = "Rcpp"` to obtain a new R function `fun` based on the C++ code in `src`:

```
R> src <- '
+   Rcpp::NumericVector xa(a);
+   Rcpp::NumericVector xb(b);
+   int n_xa = xa.size(), n_xb = xb.size();
+
+   Rcpp::NumericVector xab(n_xa + n_xb - 1);
+   for (int i = 0; i < n_xa; i++)
+     for (int j = 0; j < n_xb; j++)
+        xab[i + j] += xa[i] * xb[j];
+   return xab;
+ '
R> fun <- cxxfunction(signature(a = "numeric", b = "numeric"),
+    src, plugin = "Rcpp")
R> fun(1:3, 1:4)

[1]  1  4 10 16 17 12
```

With one assignment to the R variable `src`, and one call of the R function `cxxfunction` (provided by the **inline** package), we have created a new R function `fun` that uses the C++ code we assigned to `src`—and all this functionality can be used directly from the R prompt making prototyping with C++ functions straightforward.

Update: **Rcpp** version 0.10.0 and later contain new and powerful feature called 'Rcpp Attributes' which provides an even more powerful mechanism; see Allaire *et al.* (2013) for more details.

[3]This presumes a platform for which pre-built binaries are provided. **Rcpp** is available in binary form for Windows and OS X users from CRAN, and as a `.deb` package for Debian and Ubuntu users. For other systems, the **Rcpp** library is automatically built from source during installation or upgrades.

6. Using Standard Template Library algorithms

The STL offers a variety of generic algorithms designed to be used on ranges of elements (Plauger *et al.* 2000). A range is any sequence of objects that can be accessed through iterators or pointers. All **Rcpp** classes from the new API representing vectors (including lists) can produce ranges through their member functions `begin()` and `end()`, effectively supporting iterating over elements of an R vector.

The following code illustrates how **Rcpp** might be used to emulate a simpler[4] version of `lapply` using the `transform` algorithm from the STL.

```
R> src <- '
+   Rcpp::List input(data);
+   Rcpp::Function f(fun);
+   Rcpp::List output(input.size());
+   std::transform(input.begin(), input.end(), output.begin(), f);
+   output.names() = input.names();
+   return output;
+   '
R> cpp_lapply <- cxxfunction(signature(data = "list", fun = "function"),
+    src, plugin = "Rcpp")
```

We can now use this `cpp_lapply` function to calculate a summary of each column of the `faithful` data set included with R.

```
R> cpp_lapply(faithful, summary)

$eruptions
 Min. 1st Qu.  Median    Mean 3rd Qu.    Max.
1.600   2.163   4.000   3.488   4.454   5.100

$waiting
 Min. 1st Qu.  Median    Mean 3rd Qu.    Max.
 43.0    58.0    76.0    70.9    82.0    96.0
```

7. Error handling

Code that uses both R and C++ has to deal with two distinct error handling models. **Rcpp** simplifies this and allows both systems to work together.

7.1. C++ exceptions in R

The internals of the R condition mechanism and the implementation of C++ exceptions are both based on a layer above POSIX jumps. These layers both assume total control over the call stack and should not be used together without extra precaution. **Rcpp** contains facilities

[4] The version of `lapply` does not allow use of the ellipsis (...).

to combine both systems so that C++ exceptions are caught and recycled into the R condition mechanism.

Rcpp defines the `BEGIN_RCPP` and `END_RCPP` macros that should be used to bracket code that might throw C++ exceptions.

```
RcppExport SEXP fun( SEXP x ) {
BEGIN_RCPP
    int dx = Rcpp::as<int>(x);
    if( dx > 10 )
        throw std::range_error("too big");
    return Rcpp::wrap( dx * dx);
END_RCPP
}
```

The macros are simply defined to avoid code repetition. They expand to simple `try/catch` blocks so that the above example becomes:

```
RcppExport SEXP fun( SEXP x ) {
    try {
        int dx = Rcpp::as<int>(x);
        if( dx > 10 )
            throw std::range_error("too big");
        return Rcpp::wrap( dx * dx);
    } catch( std::exception& __ex__ ) {
        forward_exception_to_r( __ex__ );
    } catch(...) {
        ::Rf_error( "c++ exception (unknown reason)" );
    }
}
```

Using `BEGIN_RCPP` and `END_RCPP`—or the expanded versions—guarantees that the stack is first unwound in terms of C++ exceptions, before the problem is converted to the standard R error management system using the function `Rf_error` of the R API.

The `forward_exception_to_r` function uses run-time type information to extract information about the class of the C++ exception and its message so that dedicated handlers can be installed on the R side.

```
R> f <- function(x) .Call("fun", x)
R> tryCatch(f(12), "std::range_error" = function(e) { conditionMessage(e) })

[1] "too big"

R> tryCatch(f(12), "std::range_error" = function(e) { class(e) })

[1] "std::range_error" "C++Error"         "error"            "condition"
```

A serious limitation of this approach is the lack of support for calling handlers. R calling handlers are also based on POSIX jumps, and using both calling handlers from the R engine as well C++ exception forwarding might lead to undetermined results. Future versions of **Rcpp** might attempt to to improve this issue.

7.2. R errors in C++

R itself currently does not offer C-level mechanisms to deal with errors. To overcome this problem, **Rcpp** uses the `Rcpp_eval` function to evaluate an R expression in an R-level `tryCatch` block. The error, if any, that occurs while evaluating the function is then translated into an C++ exception that can be dealt with using regular C++ `try/catch` syntax.

An open (and rather hard) problem, however, is posed by the fact that calls into the C API offered by R cannot be reliably protected. Such calls can always encounter an error condition of their own triggering a call to `Rf_error` which will lead to a sudden death of the program. In particular, neither C++ class destructors nor `catch` parts of outer `try/catch` blocks will be called. This leaves the potential for memory or resource leakage. So while newly written code can improve on this situation via use of C++ exception handling, existing code calling into the C API of R cannot be amended just by having an outer layer of exception handling around it.

8. Performance comparison

In this section, we present several different ways to leverage **Rcpp** to rewrite the convolution example from 'Writing R Extensions' (R Development Core Team 2012, Chapter 5) first discussed in Section 2. As part of the redesign of **Rcpp**, data copy is kept to the absolute minimum: The `RObject` class and all its derived classes are just a container for a `SEXP` object. We let R perform all memory management and access data though the macros or functions offered by the standard R API.

The implementation of the `operator[]` is designed to be as efficient as possible, using both inlining and caching, but even this implementation is still less efficient than the reference C implementation described in R Development Core Team (2012).

Rcpp follows design principles from the STL, and classes such as `NumericVector` expose iterators that can be used for sequential scans of the data. Algorithms using iterators are usually more efficient than those that operate on objects using the `operator[]`. The following version of the convolution function illustrates the use of the `NumericVector::iterator`.

```
#include <Rcpp.h>

RcppExport SEXP convolve4cpp(SEXP a, SEXP b) {
    Rcpp::NumericVector xa(a), xb(b);
    int n_xa = xa.size(), n_xb = xb.size();
    Rcpp::NumericVector xab(n_xa + n_xb - 1);

    typedef Rcpp::NumericVector::iterator vec_iterator;
    vec_iterator ia = xa.begin(), ib = xb.begin();
    vec_iterator iab = xab.begin();
```

```
    for (int i = 0; i < n_xa; i++)
        for (int j = 0; j < n_xb; j++)
            iab[i + j] += ia[i] * ib[j];

    return xab;
}
```

One of the focuses of recent developments of **Rcpp** is called '**Rcpp** sugar', and aims to provide R-like syntax in C++. While a fuller discussion of **Rcpp** sugar is beyond the scope of this article, we have included another version of the convolution algorithm based on **Rcpp** sugar for illustrative purposes here:

```
#include <Rcpp.h>

RcppExport SEXP convolve11cpp(SEXP a, SEXP b) {
    Rcpp::NumericVector xa(a), xb(b);
    int n_xa = xa.size(), n_xb = xb.size();
    Rcpp::NumericVector xab(n_xa + n_xb-1, 0.0);

    Rcpp::Range r( 0, n_xb-1 );
    for (int i=0; i<n_xa; i++, r++)
        xab[ r ] += Rcpp::noNA(xa[i]) * Rcpp::noNA(xb);
    return xab ;
}
```

Rcpp sugar allows manipulation of entire subsets of vectors at once, thanks to the `Range` class. **Rcpp** sugar uses techniques such as expression templates, lazy evaluation and loop unrolling to generate very efficient code. The `noNA` template function marks its argument to indicates that it does not contain any missing values—an assumption made implicitly by other versions—allowing sugar to compute the individual operations without having to test for missing values.

We have benchmarked the various implementations by averaging over 5000 calls of each function with `a` and `b` containing 200 elements each.[5] The timings are summarized in Table 2 below.

The first implementation, written in C and using the traditional R API, provides our base case. It takes advantage of pointer arithmetics and therefore does not to pay the price of C++ object encapsulation or operator overloading.

The slowest solution illustrates the price of object encapsulation. Calling an overloaded `operator[]` as opposed to using direct pointer arithmetics as in the reference case costs about 29% in performance.

The next implementation uses iterators rather than indexing. Its performance is indistinguishable from the base case. This also shows that the use of C++ may not necessarily imply any performance penalty. Further, C++ `iterators` can be used to achieve the performance of C pointers, but without the potential dangers of direct memory access via pointers.

[5]The code is contained in the directory `inst/examples/ConvolveBenchmarks` in the **Rcpp** package.

Implementation	Time in millisec.	Relative to R API
R API (as benchmark)	218	
**Rcpp** sugar	145	0.67
`NumericVector::iterator`	217	1.00
`NumericVector::operator[]`	282	1.29

Table 2: Run-time performance of the different convolution examples.

Finally, the fastest implementation uses **Rcpp** sugar. It performs significantly better than the base case. Explicit loop unrolling provides us with vectorization at the C++ level which is responsible for this particular speedup.

9. On-going development

Rcpp is in very active development: Current work in the package (and in packages such as **RcppArmadillo**) focuses on further improving interoperability between R and C++. Two core themes for on-going development are '**Rcpp** sugar' as well as '**Rcpp** modules', both of which are also discussed in more detail in specific vignettes in the **Rcpp** package.

'**Rcpp** sugar' offers syntactic sugar at the C++ level, including optimized binary operators and many R functions such as `ifelse`, `sapply`, `any`, `head`, `tail`, and more. The main technique used in **Rcpp** sugar is expression templates pioneered by the **Blitz++** library (Veldhuizen 1998) and since adopted by projects such as **Armadillo** (Sanderson 2010). Access to most of the d/p/q/r-variants of the statistical distribution functions has also been added, enabling the use of expressions such as `dnorm(X, m, s)` for a numeric vector `X` and scalars `m` and `s`. This was shown in Table 1 in Section 4 above where the R expression `rnorm(10L, sd = 100)` was rewritten in C++ as `rnorm(10, 0, 100)`. Note that C++ semantics require the second parameter to be used here, which is different from the R case.

'**Rcpp** modules' allows programmers to expose C++ functions and classes at the R level. This offers access to C++ code from R using even less interface code than by writing accessor functions. Modules are inspired by the **Boost.Python** library (Abrahams and Grosse-Kunstleve 2003) which provides similar functionality for Python. C++ classes exposed by **Rcpp** modules are shadowed by reference classes which have been introduced in R 2.12.0.

Update: Besides the vignettes for '**Rcpp** Sugar' (Eddelbuettel and François 2014b) and '**Rcpp** Modules' (Eddelbuettel and François 2014a), the aforementioned vignette for '**Rcpp** Attributes' (Allaire *et al.* 2013) describes a new possibility for even more direct integration between Rcpp and C++.

10. Summary

The **Rcpp** package presented in this paper greatly simplifies integration of compiled C++ code with R. **Rcpp** provides a C++ class hierarchy which allows manipulation of R data structures in C++ using member functions and operators directly related to the type of object being used, thereby reducing the level of expertise required to master the various functions and macros offered by the internal R API. The classes assume the entire responsibility of garbage collection

of objects, relieving the programmer from book-keeping operations with the protection stack and enabling him/her to focus on the underlying problem.

Data interchange between R and C++ code is performed by the `wrap()` and `as()` template functions. They allow the programmer to write logic in terms of C++ data structures, and facilitate use of modern libraries such as the Standard Template Library (STL) and its containers and algorithms. The `wrap()` and `as()` template functions are extensible by design. They are also used either explicitly or implicitly throughout the API. By using only thin wrappers around `SEXP` objects and adopting C++ idioms such as iterators, the footprint of the **Rcpp** API is very lightweight, and does not incur a significant performance penalty.

The **Rcpp** API offers opportunities to dramatically reduce the complexity of code, which should lower the initial cost of writing code and improve code readability, maintainability, and reuse—without incurring noticeable penalties in run-time performance.

Acknowledgments

Detailed comments and suggestions by editors as well as anonymous referees are gratefully acknowledged. We are also thankful for code contributions by Douglas Bates and John Chambers, as well as for very helpful suggestions by Uwe Ligges, Brian Ripley and Simon Urbanek concerning the build systems for different platforms. Last but not least, several users provided very fruitful ideas for new or extended features via the `rcpp-devel` mailing list.

References

Abrahams D, Grosse-Kunstleve RW (2003). *Building Hybrid Systems with Boost.Python*. Boost Consulting. URL http://www.boostpro.com/writing/bpl.pdf.

Abrahams D, Gurtovoy A (2004). *C++ Template Metaprogramming: Concepts, Tools and Techniques from Boost and Beyond*. Addison-Wesley, Boston.

Allaire JJ, Eddelbuettel D, François R (2013). *Rcpp Attributes*. Vignette included in R package Rcpp, URL http://CRAN.R-Project.org/package=Rcpp.

Armstrong W (2009a). *RAbstraction: C++ abstraction for R objects*. Code repository last updated 2009-07-22., URL http://github.com/armstrtw/rabstraction.

Armstrong W (2009b). *RObjects: C++ wrapper for R objects (a better implementation of RAbstraction*. Code repository last updated 2009-11-28., URL http://github.com/armstrtw/RObjects.

Bates DM, DebRoy S (2001). "C++ Classes for R Objects." In K Hornik, F Leisch (eds.), *Proceedings of the 2nd International Workshop on Distributed Statistical Computing, March 15–17, 2001, Technische Universität Wien, Vienna, Austria*. ISSN 1609-395X, URL http://www.ci.tuwien.ac.at/Conferences/DSC-2001/Proceedings/.

Eddelbuettel D (2013). *Seamless R and C++ Integration with Rcpp*. Use R! Springer, New York. ISBN 978-1-4614-6867-7.

Eddelbuettel D, François R (2011). "Rcpp: Seamless R and C++ Integration." *Journal of Statistical Software*, **40**(8), 1–18. URL http://www.jstatsoft.org/v40/i08/.

Eddelbuettel D, François R (2014a). *Exposing C++ functions and classes with Rcpp modules.* Vignette included in R package Rcpp, URL http://CRAN.R-Project.org/package=Rcpp.

Eddelbuettel D, François R (2014b). *Rcpp syntactic sugar.* Vignette included in R package Rcpp, URL http://CRAN.R-Project.org/package=Rcpp.

Eddelbuettel D, François R (2014c). *RInside: C++ classes to embed R in C++ applications.* R package version 0.2.11, URL http://CRAN.R-Project.org/package=RInside.

Eddelbuettel D, François R (2015). *RcppClassic: Deprecated 'classic' Rcpp API.* R package version 0.9.6, URL http://CRAN.R-Project.org/package=RcppClassic.

Eddelbuettel D, François R, Allaire J, Chambers J, Bates D, Ushey K (2015). *Rcpp: Seamless R and C++ Integration.* R package version 0.11.4, URL http://CRAN.R-Project.org/package=Rcpp.

Eddelbuettel D, Nguyen K (2014). *RQuantLib: R interface to the QuantLib library.* R package version 0.4.0, URL http://CRAN.R-Project.org/package=RQuantLib.

François R, Eddelbuettel D (2015). *RcppGSL: Rcpp integration for GNU GSL vectors and matrices.* R package version 0.2.3, URL http://CRAN.R-Project.org/package=RcppGSL.

François R, Eddelbuettel D, Bates D (2014a). *RcppArmadillo: Rcpp integration for Armadillo templated linear algebra library.* R package version 0.4.600.0, URL http://CRAN.R-Project.org/package=RcppArmadillo.

François R, Eddelbuettel D, Stokely M, Ooms J (2014b). *RProtoBuf: R Interface to the Protocol Buffers API.* R package version 0.4.2, URL http://CRAN.R-Project.org/package=RProtoBuf.

Java JJ, Gaile DP, Manly KE (2007). "R/Cpp: Interface Classes to Simplify Using R Objects in C++ Extensions." Unpublished manuscript, University at Buffalo, URL http://sphhp.buffalo.edu/biostat/research/techreports/UB_Biostatistics_TR0702.pdf.

Liang G (2008). *rcppbind: A template library for R/C++ developers.* R package version 1.0, URL http://R-Forge.R-Project.org/projects/rcppbind.

Meyers S (1995). *More Effective C++: 35 New Ways to Improve Your Programs and Designs.* Addison-Wesley, Boston. ISBN 020163371X.

Meyers S (2005). *Effective C++: 55 Specific Ways to Improve Your Programs and Designs.* 3rd edition. Addison-Wesley, Boston. ISBN 978-0321334879.

Plauger P, Stepanov A, Lee M, Musser DR (2000). *The C++ Standard Template Library.* Prentice Hall PTR. ISBN 978-0134376332.

R Development Core Team (2012). *Writing R extensions.* R Foundation for Statistical Computing, Vienna, Austria. URL http://CRAN.R-Project.org/doc/manuals/R-exts.html.

R Development Core Team (2013a). *R: A Language and Environment for Statistical Computing*. R Foundation for Statistical Computing, Vienna, Austria. URL http://www.R-project.org/.

R Development Core Team (2013b). *R internals*. R Foundation for Statistical Computing, Vienna, Austria. URL http://CRAN.R-Project.org/doc/manuals/R-ints.html.

R Development Core Team (2013c). *R language*. R Foundation for Statistical Computing, Vienna, Austria. URL http://CRAN.R-Project.org/doc/manuals/R-lang.html.

Runnalls A (2009). "Aspects of CXXR internals." In *Directions in Statistical Computing*. University of Copenhagen, Denmark.

Sanderson C (2010). "Armadillo: An open source C++ Algebra Library for Fast Prototyping and Computationally Intensive Experiments." *Technical report*, NICTA. URL http://arma.sf.net.

Sklyar O, Murdoch D, Smith M, Eddelbuettel D, François R (2013). *inline: Inline C, C++, Fortran function calls from R*. R package version 0.3.13, URL http://CRAN.R-Project.org/package=inline.

Temple Lang D (2009a). "A modest proposal: an approach to making the internal R system extensible." *Computational Statistics*, **24**(2), 271–281.

Temple Lang D (2009b). "Working with meta-data from C/C++ code in R: the RGCCTranslationUnit package." *Computational Statistics*, **24**(2), 283–293.

Urbanek S (2003). "Rserve: A Fast Way to Provide R Functionality to Applications." In K Hornik, F Leisch, A Zeileis (eds.), *Proceedings of the 3rd International Workshop on Distributed Statistical Computing, Vienna, Austria*. ISSN 1609-395X, URL http://www.ci.tuwien.ac.at/Conferences/DSC-2003/Proceedings/.

Urbanek S (2013). *Rserve: Binary R server*. R package version 1.7-3, URL http://CRAN.R-Project.org/package=Rserve.

Vandevoorde D, Josuttis NM (2003). *C++ Templates: The Complete Guide*. Addison-Wesley, Boston.

Veldhuizen TL (1998). "Arrays in Blitz++." In *ISCOPE '98: Proceedings of the Second International Symposium on Computing in Object-Oriented Parallel Environments*, pp. 223–230. Springer-Verlag, London. ISBN 3-540-65387-2.

Affiliation:

Dirk Eddelbuettel
Debian Project
River Forest, IL, United States of America
E-mail: edd@debian.org
URL: http://dirk.eddelbuettel.com/

Romain François
Professional R Enthusiast
1 rue du Puits du Temple
34 000 Montpellier, France
E-mail: romain@r-enthusiasts.com
URL: http://romainfrancois.blog.free.fr/
