# Supplementary material for: Comparing Kaolin and Pinolene to Improve Sustainable Grapevine Production during Drought
Source: PLoS One. 2016 Jun 13;11(6):e0156631. doi: 10.1371/journal.pone.0156631 (PMC4905681; doi:10.1371/journal.pone.0156631)
Supplement: S1 File — (GZ) [file pone.0156631.s003.gz › PONE_Brillante_et_al_2016_Code_and_Data/packrat/src/Rcpp/Rcpp_0.11.6.tar.gz!/Rcpp/inst/doc/Rcpp-attributes.pdf]

Rcpp Attributes

J.J. Allaire Dirk Eddelbuettel Romain François

Rcpp version 0.11.6 as of May 1, 2015

Abstract

Rcpp attributes provide a high-level syntax for declaring C++ functions as callable from R and automatically generating the code required to invoke them. Attributes are intended to facilitate both interactive use of C++ within R sessions as well as to support R package development. The implementation of attributes is based on previous work in the **inline** package (Sklyar, Murdoch, Smith, Eddelbuettel, and François, 2013).

1 Introduction

Attributes are a new feature of **Rcpp** version 0.10.0 (Eddelbuettel, François, Allaire, Chambers, Bates, and Ushey, 2015; Eddelbuettel and François, 2011) that provide infrastructure for seamless language bindings between R and C++. The motivation for attributes is several-fold:

1. Reduce the learning curve associated with using C++ and R together
2. Eliminate boilerplate conversion and marshaling code wherever possible
3. Seamless use of C++ within interactive R sessions
4. Unified syntax for interactive work and package development

The core concept is to add annotations to C++ source files that provide the context required to automatically generate R bindings to C++ functions. Attributes and their supporting functions include:

- `Rcpp::export` attribute to export a C++ function to R
- `sourceCpp` function to source exported functions from a file
- `cppFunction` and `evalCpp` functions for inline declarations and execution
- `Rcpp::depends` attribute for specifying additional build dependencies for `sourceCpp`

Attributes can also be used for package development via the `compileAttributes` function, which automatically generates `extern "C"` and `.Call` wrappers for C++ functions within packages.

2 Using Attributes

Attributes are annotations that are added to C++ source files to provide additional information to the compiler. **Rcpp** supports attributes to indicate that C++ functions should be made available as R functions, as well as to optionally specify additional build dependencies for source files.

C++11 specifies a standard syntax for attributes (Maurer and Wong, 2008). Since this standard isn't yet fully supported across all compilers, **Rcpp** attributes are included in source files using specially formatted comments.

2.1 Exporting C++ Functions

The `sourceCpp` function parses a C++ file and looks for functions marked with the `Rcpp::export` attribute. A shared library is then built and its exported functions are made available as R functions in the specified environment. For example, this source file contains an implementation of convolve (note the `Rcpp::export` attribute in the comment above the function):

```cpp
#include <Rcpp.h>
using namespace Rcpp;

// [[Rcpp::export]]
NumericVector convolveCpp(NumericVector a, NumericVector b) {

    int na = a.size(), nb = b.size();
    int nab = na + nb - 1;
    NumericVector xab(nab);

    for (int i = 0; i < na; i++)
        for (int j = 0; j < nb; j++)
            xab[i + j] += a[i] * b[j];

    return xab;
}
```

The addition of the export attribute allows us to do this from the R prompt:

```r
sourceCpp("convolve.cpp")
convolveCpp(x, y)
```

We can now write C++ functions using built-in C++ types and **Rcpp** wrapper types and then source them just as we would an R script.

The sourceCpp function performs caching based on the last modified date of the source file and it's local dependencies so as long as the source does not change the compilation will occur only once per R session.

2.2 Specifying Argument Defaults

If default argument values are provided in the C++ function definition then these defaults are also used for the exported R function. For example, the following C++ function:

```
DataFrame readData(
    CharacterVector file,
    CharacterVector colNames = CharacterVector::create(),
    std::string comment = "#",
    bool header = true)
```

Will be exported to R as:

```
function(file, colNames=character(), comment="#", header=TRUE)
```

Note that C++ rules for default arguments still apply: they must occur consecutively at the end of the function signature and (unlike R) can't rely on the values of other arguments.

Not all C++ default argument values can be parsed into their R equivalents, however the most common cases are supported, including:

- String literals delimited by quotes (e.g. "foo")
- Decimal numeric values (e.g. 10 or 4.5)
- Pre-defined constants including true, false, R_NilValue, NA_STRING, NA_INTEGER, NA_REAL, and NA_LOGICAL.
- Selected vector types (CharacterVector, IntegerVector, and NumericVector) instantiated using the ::create static member function.
- Matrix types instantiated using the rows, cols constructor.

2.3 Signaling Errors

Within R code the stop function is typically used to signal errors. Within R extensions written in C the Rf_error function is typically used. However, within C++ code you cannot safely use Rf_error because it results in a longjmp over any C++ destructors on the stack.

The correct way to signal errors within C++ functions is to throw an Rcpp::exception. For example:

```
if (unexpectedCondition)
    throw Rcpp::exception("Unexpected condition occurred");
```

There is also an `Rcpp::stop` function that is shorthand for throwing an `Rcpp::exception`. For example:

```
if (unexpectedCondition)
    Rcpp::stop("Unexpected condition occurred");
```

In both cases the C++ exception will be caught by **Rcpp** prior to returning control to R and converted into the correct signal to R that execution should stop with the specified message.

You can similarly also signal warnings with the `Rcpp::warning` function:

```
if (unexpectedCondition)
    Rcpp::warning("Unexpected condition occurred");
```

2.4 Supporting User Interruption

If your function may run for an extended period of time, users will appreciate the ability to interrupt it's processing and return to the REPL. This is handled automatically for R code (as R checks for user interrupts periodically during processing) however requires explicit accounting for in C and C++ extensions to R. To make computations interrupt-able, you should periodically call the `Rcpp::checkUserInterrupt` function, for example:

```
for (int i=0; i<1000000; i++) {

    // check for interrupt every 1000 iterations
    if (i % 1000 == 0)
        Rcpp::checkUserInterrupt();

    // ...do some expensive work...

}
```

A good guideline is to call `Rcpp::checkUserInterrupt` every 1 or 2 seconds that your computation is running. In the above code, if the user requests an interrupt then an exception is thrown and the attributes wrapper code arranges for the user to be returned to the REPL.

Note that R provides a C API for the same purpose (`R_CheckUserInterrupt`) however this API is not safe to use in C++ code as it uses `longjmp` to exit the current scope, bypassing any C++ destructors on the stack. The `Rcpp::checkUserInterrupt` function is provided as a safe alternative for C++ code.

2.5 Embedding R Code

Typically C++ and R code are kept in their own source files. However, it's often convenient to bundle code from both languages into a common source file that can be executed using single call to `sourceCpp`.

To embed chunks of R code within a C++ source file you include the R code within a block comment that has the prefix of `/*** R`. For example:

```
/*** R

# Call the fibonacci function defined in C++
fibonacci(10)

*/
```

Multiple R code chunks can be included in a C++ file. The `sourceCpp` function will first compile the C++ code into a shared library and then source the embedded R code.

2.6 Modifying Function Names

You can change the name of an exported function as it appears to R by adding a name parameter to `Rcpp::export`. For example:

```
// [[Rcpp::export(name = ".convolveCpp")]]
NumericVector convolveCpp(NumericVector a, NumericVector b)
```

Note that in this case since the specified name is prefaced by a `.` the exported R function will be hidden. You can also use this method to provide implementations of S3 methods (which wouldn't otherwise be possible because C++ functions can't contain a '.' in their name).

2.7 Function Requirements

Functions marked with the `Rcpp::export` attribute must meet several requirements to be correctly handled:

- Be defined in the global namespace (i.e. not within a C++ namespace declaration)
- Have a return type that is either void or compatible with `Rcpp::wrap` and parameter types that are compatible with `Rcpp::as` (see sections 3.1 and 3.2 of the '*Rcpp-introduction*' vignette for more details).
- Use fully qualified type names for the return value and all parameters. Rcpp types may however appear without a namespace qualifier (i.e. `DataFrame` is okay as a type name but `std::string` must be specified fully).

2.8 Random Number Generation

R functions implemented in C or C++ need to be careful to surround use of internal random number generation routines (e.g. `unif_rand`) with calls to `GetRNGstate` and `PutRNGstate`.

Within **Rcpp**, this is typically done using the `RNGScope` class. However, this is not necessary for C++ functions exported using attributes because an `RNGScope` is established for them automatically. Note that **Rcpp** implements `RNGScope` using a counter, so it's still safe to execute code that may establish it's own `RNGScope` (such as the **Rcpp** sugar functions that deal with random number generation).

The overhead associated with using `RNGScope` is negligible (only a couple of milliseconds) and it provides a guarantee that all C++ code will inter-operate correctly with R's random number generation. If you are certain that no C++ code will make use of random number generation and the 2ms of execution time is meaningful in your context, you can disable the automatic injection of `RNGScope` using the `rng` parameter of the `Rcpp::export` attribute. For example:

```
// [[Rcpp::export(rng = false)]]
double myFunction(double input) {
    // ...code that never uses R random number generation...
}
```

2.9 Importing Dependencies

It's also possible to use the `Rcpp::depends` attribute to declare dependencies on other packages. For example:

```cpp
// [[Rcpp::depends(RcppArmadillo)]]

#include <RcppArmadillo.h>
using namespace Rcpp;

// [[Rcpp::export]]
List fastLm(NumericVector yr, NumericMatrix Xr) {

    int n = Xr.nrow(), k = Xr.ncol();

    arma::mat X(Xr.begin(), n, k, false);
    arma::colvec y(yr.begin(), yr.size(), false);

    arma::colvec coef = arma::solve(X, y);
    arma::colvec resid = y - X*coef;

    double sig2 = arma::as_scalar(arma::trans(resid)*resid/(n-
k));
    arma::colvec stderrest = arma::sqrt(
          sig2 * arma::diagvec( arma::inv(arma::trans(X)*X)) );

    return List::create(Named("coefficients") = coef,
                        Named("stderr")       = stderrest);
}
```

The inclusion of the `Rcpp::depends` attribute causes `sourceCpp` to configure the build environment to correctly compile and link against the **RcppArmadillo** package. Source files can declare more than one dependency either by using multiple `Rcpp::depends` attributes or with syntax like this:

```cpp
// [[Rcpp::depends(Matrix, RcppArmadillo)]]
```

Dependencies are discovered both by scanning for package include directories and by invoking **inline** plugins if they are available for a package.

Note that while the `Rcpp::depends` attribute establishes dependencies for `sourceCpp`, it's important to note that if you include the same source file in an R package these dependencies must still be listed in the `Imports` and/or `LinkingTo` fields of the package `DESCRIPTION` file.

2.10 Sharing Code

The core use case for `sourceCpp` is the compilation of a single self-contained source file. Code within this file can import other C++ code by using the `Rcpp::depends` attribute as described above.

The recommended practice for sharing C++ code across many uses of `sourceCpp` is therefore to create an R package to wrap the C++ code. This has many benefits not the least of which is easy distribution of shared code. More information on creating packages that contain C++ code is included in the Package Development section below.

2.10.1 Shared Code in Header Files

If you need to share a small amount of C++ code between source files compiled with `sourceCpp` and the option of creating a package isn't practical, then you can also share code using local includes of C++ header files. To do this, create a header file with the definition of shared functions, classes, enums, etc. For example:

```
#ifndef __UTILITIES__
#define __UTILITIES__

inline double timesTwo(double x) {
    return x * 2;
}

#endif // __UTILITIES__
```

Note the use of the `#ifndef` include guard, this is import to ensure that code is not included more than once in a source file. You should use an include guard and be sure to pick a unique name for the corresponding `#define`.

Also note the use of the `inline` keyword preceding the function. This is important to ensure that there are not multiple definitions of functions included from header files. Classes fully defined in header files automatically have inline semantics so don't require this treatment.

To use this code in a source file you'd just include it based on it's relative path (being sure to use `"` as the delimiter to indicate a local file reference). For example:

```
#include "shared/utilities.hpp"

// [[Rcpp::export]]
double transformValue(double x) {
    return timesTwo(x) * 10;
}
```

2.10.2 Shared Code in C++ Files

When scanning for locally included header files `sourceCpp` also checks for a corresponding implementation file and automatically includes it in the compilation if it exists.

This enables you to break the shared code entirely into it's own source file. In terms of the above example, this would mean having only a function declaration in the header:

```
#ifndef __UTILITIES__
#define __UTILITIES__

double timesTwo(double x);

#endif // __UTILITIES__
```

Then actually defining the function in a separate source file with the same base name as the header file but with a .cpp extension (in the above example this would be `utilities.cpp`):

```
#include "utilities.hpp"

double timesTwo(double x) {
    return x * 2;
}
```

It's also possible to use attributes to declare dependencies and exported functions within shared header and source files. This enables you to take a source file that is typically used standalone and include it when compiling another source file.

Note that since additional source files are processed as separate translation units the total compilation time will increase proportional to the number of files processed. From this standpoint it's often preferable to use shared header files with definitions fully inlined as demonstrated above.

Note also that embedded R code is only executed for the main source file not those referenced by local includes.

2.11 Including C++ Inline

Maintaining C++ code in it's own source file provides several benefits including the ability to use C++ aware text-editing tools and straightforward mapping of compilation errors to lines in the source file. However, it's also possible to do inline declaration and execution of C++ code.

There are several ways to accomplish this, including passing a code string to `sourceCpp` or using the shorter-form `cppFunction` or `evalCpp` functions. For example:

```
cppFunction('
    int fibonacci(const int x) {
        if (x < 2)
            return x;
        else
            return (fibonacci(x - 1)) + fibonacci(x - 2);
    }
')

evalCpp('std::numeric_limits<double>::max()')
```

You can also specify a depends parameter to `cppFunction` or `evalCpp`:

```
cppFunction(depends = 'RcppArmadillo', code = '...')
```

3 Package Development

One of the goals of **Rcpp** attributes is to simultaneously facilitate ad-hoc and interactive work with C++ while also making it very easy to migrate that work into an R package. There are several benefits of moving code from a standalone C++ source file to a package:

1. Your code can be made available to users without C++ development tools (at least on Windows or Mac OS X where binary packages are common)
2. Multiple source files and their dependencies are handled automatically by the R package build system
3. Packages provide additional infrastructure for testing, documentation and consistency

3.1 Package Creation

To create a package that is based on **Rcpp** you should follow the guidelines in the '*Rcpp-package*' vignette. For a new package this is most conveniently done using the `Rcpp.package.skeleton` function.

To generate a new package with a simple hello, world function that uses attributes you can do the following:

```
Rcpp.package.skeleton("NewPackage", attributes = TRUE)
```

To generate a package based on C++ files that you've been using with `sourceCpp` you can use the `cpp_files` parameter:

```r
Rcpp.package.skeleton("NewPackage", example_code = FALSE,
                      cpp_files = c("convolve.cpp"))
```

3.2 Specifying Dependencies

Once you've migrated C++ code into a package, the dependencies for source files are derived from the Imports and LinkingTo fields in the package DESCRIPTION file rather than the Rcpp::depends attribute. Some packages also require the addition of an entry to the package NAMESPACE file to ensure that the package's shared library is loaded prior to callers using the package. For every package you import C++ code from (including **Rcpp**) you need to add these entries.

Packages that provide only C++ header files (and no shared library) need only be referred to using LinkingTo. You should consult the documentation for the package you are using for the requirements particular to that package.

For example, if your package depends on **Rcpp** you'd have the following entries in the DESCRIPTION file:

```
Imports: Rcpp (>= 0.11.4)
LinkingTo: Rcpp
```

And the following entry in your NAMESPACE file:

```
importFrom(Rcpp, evalCpp)
```

If your package additionally depended on the **BH** (Boost headers) package you'd just add an entry for **BH** to the LinkingTo field since **BH** is a header-only package:

```
Imports: Rcpp (>= 0.11.4)
LinkingTo: Rcpp, BH
```

3.3 Exporting R Functions

Within interactive sessions you call the sourceCpp function on individual files to export C++ functions into the global environment. However, for packages you call a single utility function to export all C++ functions within the package.

The compileAttributes function scans the source files within a package for export attributes and generates code as required. For example, executing this from within the package working directory:

```r
compileAttributes()
```

Results in the generation of the following two source files:

- `src/RcppExports.cpp` – The `extern "C"` wrappers required to call exported C++ functions within the package.
- `R/RcppExports.R` – The `.Call` wrappers required to call the `extern "C"` functions defined in `RcppExports.cpp`.

You should re-run `compileAttributes` whenever functions are added, removed, or have their signatures changed. Note that if you are using either RStudio or **devtools** to build your package then the `compileAttributes` function is called automatically whenever your package is built.

The `compileAttributes` function deals only with exporting C++ functions to R. If you want the functions to additionally be publicly available from your package's namespace another step may be required. Specifically, if your package `NAMESPACE` file does not use a pattern to export functions then you should add an explicit entry to `NAMESPACE` for each R function you want publicly available.

3.4 Types in Generated Code

In some cases the signatures of the C++ functions that are generated within `RcppExports.cpp` may have additional type requirements beyond the core standard library and **Rcpp** types (e.g. `CharacterVector`, `NumericVector`, etc.). Examples might include convenience typedefs, as/wrap handlers for marshaling between custom types and SEXP, or types wrapped by the Rcpp `XPtr` template.

In this case, you can create a header file that contains these type definitions (either defined inline or by including other headers) and have this header file automatically included in `RcppExports.cpp`. Headers named with the convention `pkgname_types` are automatically included along with the generated C++ code. For example, if your package is named **fastcode** then any of the following header files would be automatically included in `RcppExports.cpp`:

```
src/fastcode_types.h
src/fastcode_types.hpp
inst/include/fastcode_types.h
inst/include/fastcode_types.hpp
```

There is one other mechanism for type visibility in `RcppExports.cpp`. If your package provides a master include file for consumption by C++ clients then this file will also be automatically included. For example, if the **fastcode** package had a C++ API and the following header file:

```
inst/include/fastcode.h
```

This header file will also automatically be included in `RcppExports.cpp`. Note that the convention of using `.h` for header files containing C++ code may seem unnatural, but this

comes from the recommended practices described in '*Writing R Extensions*' (R Development Core Team, 2012).

3.5 Roxygen Comments

The **roxygen2** package (Wickham, Danenberg, and Eugster, 2014) provides a facility for automatically generating R documentation files based on specially formatted comments in R source code.

If you include roxygen comments in your C++ source file with a `//'` prefix then `compileAttributes` will transpose them into R roxygen comments within `R/RcppExports.R`. For example the following code in a C++ source file:

```
//' The length of a string (in characters).
//'
//' @param str input character vector
//' @return characters in each element of the vector
// [[Rcpp::export]]
NumericVector strLength(CharacterVector str)
```

Results in the following code in the generated R source file:

```
#' The length of a string (in characters).
#'
#' @param str input character vector
#' @return characters in each element of the vector
strLength <- function(str)
```

3.6 Providing a C++ Interface

The interface exposed from R packages is most typically a set of R functions. However, the R package system also provides a mechanism to allow the exporting of C and C++ interfaces using package header files. This is based on the `R_RegisterCCallable` and `R_GetCCallable` functions described in '*Writing R Extensions*' (R Development Core Team, 2012).

C++ interfaces to a package are published within the top level `include` directory of the package (which within the package source directory is located at `inst/include`). The R build system automatically adds the required `include` directories for all packages specified in the `LinkingTo` field of the package `DESCRIPTION` file.

3.6.1 Interfaces Attribute

The `Rcpp::interfaces` attribute can be used to automatically generate a header-only interface to your C++ functions within the `include` directory of your package.

The `Rcpp::interfaces` attribute is specified on a per-source file basis, and indicates which interfaces (R, C++, or both) should be provided for exported functions within the file.

For example, the following specifies that both R and C++ interfaces should be generated for a source file:

```
// [[Rcpp::interfaces(r, cpp)]]
```

Note that the default behavior if an `Rcpp::interfaces` attribute is not included in a source file is to generate an R interface only.

3.6.2 Generated Code

If you request a `cpp` interface for a source file then `compileAttributes` generates the following header files (substituting *Package* with the name of the package code is being generated for):

```
inst/include/Package.h
inst/include/Package_RcppExports.h
```

The `Package_RcppExports.h` file has inline definitions for all exported C++ functions that enable calling them using the `R_GetCCallable` mechanism.

The `Package.h` file does nothing other than include the `Package_RcppExports.h` header. This is done so that package authors can replace the `Package.h` header with a custom one and still be able to include the automatically generated exports (details on doing this are provided in the next section).

The exported functions are defined within a C++ namespace that matches the name of the package. For example, an exported C++ function `bar` could be called from package `MyPackage` as follows:

```
// [[Rcpp::depends(MyPackage)]]

#include <MyPackage.h>

void foo() {
    MyPackage::bar();
}
```

3.6.3 Including Additional Code

You might wish to use the Rcpp::interfaces attribute to generate a part of your package's C++ interface but also provide additional custom C++ code. In this case you should replace the generated Package.h file with one of your own.

Note that the way **Rcpp** distinguishes user verses generated files is by checking for the presence a special token in the file (if it's present then it's known to be generated and thus safe to overwrite). You'll see this token at the top of the generated Package.h file, be sure to remove it if you want to provide a custom header.

Once you've established a custom package header file, you need only include the Package_RcppExports.h file within your header to make available the automatically generated code alongside your own.

If you need to include code from your custom header files within the compilation of your package source files, you will also need to add the following entry to Makevars and Makevars.win (both are in the src directory of your package):

```
PKG_CPPFLAGS += -I../inst/include/
```

Note that the R package build system does not automatically force a rebuild when headers in inst/include change, so you should be sure to perform a full rebuild of the package after making changes to these headers.

References

Dirk Eddelbuettel and Romain François. Rcpp: Seamless R and C++ integration. *Journal of Statistical Software*, 40(8):1–18, 2011. URL http://www.jstatsoft.org/v40/i08/.

Dirk Eddelbuettel, Romain François, JJ Allaire, John Chambers, Douglas Bates, and Kevin Ushey. *Rcpp: Seamless R and C++ Integration*, 2015. URL http://CRAN.R-Project.org/package=Rcpp. R package version 0.11.4.

Jens Maurer and Michael Wong. Towards support for attributes in C++ (revision 6). In *JTC1/SC22/WG21 - The C++ Standards Committee*, 2008. URL http://www.open-std.org/jtc1/sc22/wg21/docs/papers/2008/n2761.pdf. N2761=08-0271.

R Development Core Team. *Writing R extensions*. R Foundation for Statistical Computing, Vienna, Austria, 2012. URL http://CRAN.R-Project.org/doc/manuals/R-exts.html.

Oleg Sklyar, Duncan Murdoch, Mike Smith, Dirk Eddelbuettel, and Romain François. *inline: Inline C, C++, Fortran function calls from R*, 2013. URL http://CRAN.R-Project.org/package=inline. R package version 0.3.13.

Hadley Wickham, Peter Danenberg, and Manuel Eugster. *roxygen2: In-source documentation for R*, 2014. URL http://CRAN.R-Project.org/package=roxygen2. R package version 4.1.0.
