# Supplementary material for: Comparing Kaolin and Pinolene to Improve Sustainable Grapevine Production during Drought
Source: PLoS One. 2016 Jun 13;11(6):e0156631. doi: 10.1371/journal.pone.0156631 (PMC4905681; doi:10.1371/journal.pone.0156631)
Supplement: S1 File — (GZ) [file pone.0156631.s003.gz › PONE_Brillante_et_al_2016_Code_and_Data/packrat/src/Rcpp/Rcpp_0.11.6.tar.gz!/Rcpp/inst/doc/Rcpp-extending.pdf]

Extending **Rcpp**

Dirk Eddelbuettel   Romain François

Rcpp version 0.11.6 as of May 1, 2015

Abstract

This note provides an overview of the steps programmers should follow to extend **Rcpp** (Eddelbuettel, François, Allaire, Chambers, Bates, and Ushey, 2015; Eddelbuettel and François, 2011) for use with their own classes. This document is based on our experience in extending **Rcpp** to work with the **Armadillo** (Sanderson, 2010) classes, available in the separate package **RcppArmadillo** (François, Eddelbuettel, and Bates, 2014). This document assumes knowledge of **Rcpp** as well as some knowledge of C++ templates (Abrahams and Gurtovoy, 2004).

1 Introduction

Rcpp facilitates data interchange between R and C++ through the templated functions `Rcpp::as` (for conversion of objects from R to C++) and `Rcpp::wrap` (for conversion from C++ to R). In other words, we convert between the so-called S-expression pointers (in type `SEXP`) to a templated C++ type, and vice versa. The corresponding function declarations are as follows:

```
// conversion from R to C++
template <typename T> T as( SEXP x) ;

// conversion from C++ to R
template <typename T> SEXP wrap(const T& object) ;
```

These converters are often used implicitly, as in the following code chunk:

```
// we get a list from R
List input(input_) ;

// pull std::vector<double> from R list
// this is achieved through an implicit call to Rcpp::as
std::vector<double> x = input["x"] ;

// return an R list
// this is achieved through implicit call to Rcpp::wrap
return List::create(
    _["front"] = x.front(),
    _["back"]  = x.back()
    ) ;
```

```
> fx <- cxxfunction( signature( input_ = "list"),
+         paste( readLines( "code.cpp" ), collapse = "\n" ),
+         plugin = "Rcpp"
+         )
> input <- list( x = seq(1, 10, by = 0.5) )
> fx( input )
$front
[1] 1

$back
[1] 10
```

The **Rcpp** converter function `Rcpp::as` and `Rcpp::wrap` have been designed to be extensible to user-defined types and third-party types.

2 Extending `Rcpp::wrap`

The **Rcpp::wrap** converter is extensible in essentially two ways : intrusive and non-intrusive.

2.1 Intrusive extension

When extending **Rcpp** with your own data type, the recommended way is to implement a conversion to `SEXP`. This lets `Rcpp::wrap` know about the new data type. The template meta programming (or TMP) dispatch is able to recognize that a type is convertible to a `SEXP` and `Rcpp::wrap` will use that conversion.

The caveat is that the type must be declared before the main header file `Rcpp.h` is included.

```
#include <RcppCommon.h>

class Foo {
    public:
        Foo() ;

        // this operator enables implicit Rcpp::wrap
        operator SEXP() ;
}

#include <Rcpp.h>
```

This is called *intrusive* because the conversion to `SEXP` operator has to be declared within the class.

2.2 Non-intrusive extension

It is often desirable to offer automatic conversion to third-party types, over which the developer has no control and can therefore not include a conversion to `SEXP` operator in the class definition.

To provide automatic conversion from C++ to R, one must declare a specialization of the `Rcpp::wrap` template between the includes of `RcppCommon.h` and `Rcpp.h`.

```
#include <RcppCommon.h>

// third party library that declares class Bar
#include <foobar.h>

// declaring the specialization
namespace Rcpp {
    template <> SEXP wrap( const Bar& ) ;
}

// this must appear after the specialization,
// otherwise the specialization will not be seen by Rcpp types
#include <Rcpp.h>
```

It should be noted that only the declaration is required. The implementation can appear after the `Rcpp.h` file is included, and therefore take full advantage of the **Rcpp** type system.

2.3 Templates and partial specialization

It is perfectly valid to declare a partial specialization for the `Rcpp::wrap` template. The compiler will identify the appropriate overload:

```
#include <RcppCommon.h>

// third party library that declares template class Bling<T>
#include <foobar.h>

// declaring the partial specialization
namespace Rcpp {
    namespace traits {

        template <typename T> SEXP wrap( const Bling<T>& ) ;

    }
}

// this must appear after the specialization,
// otherwise the specialization will not be seen by Rcpp types
#include <Rcpp.h>
```

3 Extending `Rcpp::as`

Conversion from R to C++ is also possible in both intrusive and non-intrusive ways.

3.1 Intrusive extension

As part of its template meta programming dispatch logic, **Rcpp::as** will attempt to use the constructor of the target class taking a `SEXP`.

```
#include <RcppCommon.h>

#include <RcppCommon.h>

class Foo{
    public:
        Foo() ;

        // this constructor enables implicit Rcpp::as
        Foo(SEXP) ;
}

#include <Rcpp.h>


// this must appear after the specialization,
// otherwise the specialization will not be seen by Rcpp types
#include <Rcpp.h>
```

3.2 Non intrusive extension

It is also possible to fully specialize `Rcpp::as` to enable non intrusive implicit conversion capabilities.

```
#include <RcppCommon.h>

// third party library that declares class Bar
#include <foobar.h>

// declaring the specialization
namespace Rcpp {
    template <> Bar as( SEXP ) ;
}

// this must appear after the specialization,
// otherwise the specialization will not be seen by Rcpp types
#include <Rcpp.h>
```

3.3 Templates and partial specialization

The signature of `Rcpp::as` does not allow partial specialization. When exposing a templated class to `Rcpp::as`, the programmer must specialize the **Rcpp::traits::Exporter** template class. The TMP dispatch will recognize that a specialization of `Exporter` is available and delegate the conversion to this class. **Rcpp** defines the `Rcpp::traits::Exporter` template class as follows :

```
namespace Rcpp {
    namespace traits {

        template <typename T> class Exporter{
        public:
            Exporter( SEXP x ) : t(x){}
            inline T get(){ return t ; }

        private:
            T t ;
        } ;
    }
}
```

This is the reason why the default behavior of `Rcpp::as` is to invoke the constructor of the type `T` taking a `SEXP`. Since partial specialization of class templates is allowed, we can expose a set of classes as follows:

```
#include <RcppCommon.h>

// third party library that declares template class Bling<T>
#include <foobar.h>

// declaring the partial specialization
namespace Rcpp {
    namespace traits {
        template <typename T> class Exporter< Bling<T> >;
    }
}

// this must appear after the specialization,
// otherwise the specialization will not be seen by Rcpp types
#include <Rcpp.h>
```

Using this approach, the requirements for the `Exporter< Bling<T> >` class are:

- it should have a constructor taking a `SEXP`
- it should have a methods called `get` that returns an instance of the `Bling<T>` type.

4 Summary

The **Rcpp** package greatly facilitates the transfer of objects between R and C++. This note has shown how to extend **Rcpp** to either user-defined or third-party classes via the `Rcpp::as` and `Rcpp::wrap` template functions. Both intrusive and non-intrusive approaches were discussed.

References

David Abrahams and Aleksey Gurtovoy. *C++ Template Metaprogramming: Concepts, Tools and Techniques from Boost and Beyond*. Addison-Wesley, Boston, 2004.

Dirk Eddelbuettel and Romain François. Rcpp: Seamless R and C++ integration. *Journal of Statistical Software*, 40(8): 1–18, 2011. URL http://www.jstatsoft.org/v40/i08/.

Dirk Eddelbuettel, Romain François, JJ Allaire, John Chambers, Douglas Bates, and Kevin Ushey. *Rcpp: Seamless R and C++ Integration*, 2015. URL `http://CRAN.R-Project.org/package=Rcpp`. R package version 0.11.4.

Romain François, Dirk Eddelbuettel, and Douglas Bates. *RcppArmadillo: Rcpp integration for Armadillo templated linear algebra library*, 2014. URL `http://CRAN.R-Project.org/package=RcppArmadillo`. R package version 0.4.600.0.

Conrad Sanderson. Armadillo: An open source C++ algebra library for fast prototyping and computationally intensive experiments. Technical report, NICTA, 2010. URL `http://arma.sf.net`.
