# Supplementary material for: Comparing Kaolin and Pinolene to Improve Sustainable Grapevine Production during Drought
Source: PLoS One. 2016 Jun 13;11(6):e0156631. doi: 10.1371/journal.pone.0156631 (PMC4905681; doi:10.1371/journal.pone.0156631)
Supplement: S1 File — (GZ) [file pone.0156631.s003.gz › PONE_Brillante_et_al_2016_Code_and_Data/packrat/src/Rcpp/Rcpp_0.11.6.tar.gz!/Rcpp/inst/doc/Rcpp-modules.pdf]

# Exposing C++ functions and classes with **Rcpp** modules

Dirk Eddelbuettel    Romain François

**Rcpp** version 0.11.6 as of May 1, 2015

**Abstract**

This note discusses *Rcpp modules*. *Rcpp modules* allow programmers to expose C++ functions and classes to R with relative ease. *Rcpp modules* are inspired from the **Boost.Python** C++ library (Abrahams and Grosse-Kunstleve, 2003) which provides similar features for Python.

## 1 Motivation

Exposing C++ functionality to R is greatly facilitated by the **Rcpp** package and its underlying C++ library (Eddelbuettel, François, Allaire, Chambers, Bates, and Ushey, 2015; Eddelbuettel and François, 2011). **Rcpp** smoothes many of the rough edges in R and C++ integration by replacing the traditional R Application Programming Interface (API) described in '*Writing R Extensions*' (R Development Core Team, 2012) with a consistent set of C++ classes. The '*Rcpp-introduction*' vignette (Eddelbuettel et al., 2015; Eddelbuettel and François, 2011) describes the API and provides an introduction to using **Rcpp**.

These **Rcpp** facilities offer a lot of assistance to the programmer wishing to interface R and C++. At the same time, these facilities are limited as they operate on a function-by-function basis. The programmer has to implement a `.Call` compatible function (to conform to the R API) using classes of the **Rcpp** API as described in the next section.

### 1.1 Exposing functions using Rcpp

Exposing existing C++ functions to R through **Rcpp** usually involves several steps. One approach is to write an additional wrapper function that is responsible for converting input objects to the appropriate types, calling the actual worker function and converting the results back to a suitable type that can be returned to R (`SEXP`). Consider the `norm` function below:

```
double norm( double x, double y ) {
    return sqrt( x*x + y*y );
}
```

This simple function does not meet the requirements set by the `.Call` convention, so it cannot be called directly by R. Exposing the function involves writing a simple wrapper function that does match the `.Call` requirements. **Rcpp** makes this easy.

```
using namespace Rcpp;
RcppExport SEXP norm_wrapper(SEXP x_, SEXP y_) {
    // step 0: convert input to C++ types
    double x = as<double>(x_), y = as<double>(y_);

    // step 1: call the underlying C++ function
    double res = norm( x, y );

    // step 2: return the result as a SEXP
    return wrap( res );
}
```

Here we use the (templated) **Rcpp** converter `as()` which can transform from a `SEXP` to a number of different C++ and **Rcpp** types. The **Rcpp** function `wrap()` offers the opposite functionality and converts many known types to a `SEXP`.

This process is simple enough, and is used by a number of CRAN packages. However, it requires direct involvement from the programmer, which quickly becomes tiresome when many functions are involved. *Rcpp modules* provides a much more elegant and unintrusive way to expose C++ functions such as the `norm` function shown above to R.

We should note that **Rcpp** now has *Rcpp attributes* which extends certain aspect of *Rcpp modules* and makes binding to simple functions such as this one even easier. With *Rcpp attribues* we can just write

```
# include <Rcpp.h>

// [[Rcpp::export]]
double norm(double x, double y) {
    return sqrt(x*x + y*y);
}
```

See the corresponding vignette (Allaire, Eddelbuettel, and François, 2013) for details, but read on for *Rcpp modules* which contains to provide features not covered by *Rcpp attributes*, particularly when it comes to binding entire C++ classes and more.

## 1.2 Exposing classes using Rcpp

Exposing C++ classes or structs is even more of a challenge because it requires writing glue code for each member function that is to be exposed.

Consider the simple `Uniform` class below:

```
class Uniform {
public:
    Uniform(double min_, double max_) : min(min_), max(max_) {}

    NumericVector draw(int n) {
        RNGScope scope;
        return runif( n, min, max );
    }

private:
    double min, max;
};
```

To use this class from R, we at least need to expose the constructor and the `draw` method. External pointers (R Development Core Team, 2012) are the perfect vessel for this, and using the `Rcpp:::XPtr` template from **Rcpp** we can expose the class with these two functions:

```
using namespace Rcpp;

/// create an external pointer to a Uniform object
RcppExport SEXP Uniform__new(SEXP min_, SEXP max_) {
    // convert inputs to appropriate C++ types
    double min = as<double>(min_), max = as<double>(max_);

    // create a pointer to an Uniform object and wrap it
    // as an external pointer
    Rcpp::XPtr<Uniform> ptr( new Uniform( min, max ), true );

    // return the external pointer to the R side
    return ptr;
}

/// invoke the draw method
RcppExport SEXP Uniform__draw( SEXP xp, SEXP n_ ) {
    // grab the object as a XPtr (smart pointer) to Uniform
    Rcpp::XPtr<Uniform> ptr(xp);

    // convert the parameter to int
    int n = as<int>(n_);

    // invoke the function
    NumericVector res = ptr->draw( n );

    // return the result to R
    return res;
}
```

As it is generally a bad idea to expose external pointers 'as is', they usually get wrapped as a slot of an S4 class.

Using `cxxfunction()` from the **inline** package, we can build this example on the fly. Suppose the previous example code assigned to a text variable `unifModcode`, we could then do

```
> f1 <- cxxfunction( , "", includes = unifModCode, plugin = "Rcpp" )
> getDynLib(f1)
```

The following listing shows some *manual* wrapping to access the code, we will see later how this can be automated:

```
> setClass( "Uniform", representation( pointer = "externalptr" ) )
> Uniform_method <- function(name) {
+     paste( "Uniform", name, sep = "__" )
+ }
> setMethod( "$", "Uniform", function(x, name ) {
+     function(...) .Call( Uniform_method(name) , x@pointer, ... )
+ } )
> setMethod( "initialize", "Uniform", function(.Object, ...) {
+     .Object@pointer <- .Call( Uniform_method("new"), ... )
+     .Object
+ } )
> u <- new( "Uniform", 0, 10 )
> u$draw( 10L )
```

**Rcpp** considerably simplifies the code that would be involved for using external pointers with the traditional R API. Yet this still involves a lot of mechanical code that quickly becomes hard to maintain and error prone. *Rcpp modules* offer an elegant way to expose the `Uniform` class in a way that makes both the internal C++ code and the R code easier.

# 2 Rcpp modules

The design of Rcpp modules has been influenced by Python modules which are generated by the `Boost.Python` library (Abrahams and Grosse-Kunstleve, 2003). Rcpp modules provide a convenient and easy-to-use way to expose C++ functions and classes to R, grouped together in a single entity.

A Rcpp module is created in a `cpp` file using the `RCPP_MODULE` macro, which then provides declarative code of what the module exposes to R.

## 2.1 Exposing C++ functions using Rcpp modules

Consider the `norm` function from the previous section. We can expose it to R :

```
using namespace Rcpp;

double norm( double x, double y ) {
    return sqrt( x*x + y*y );
}

RCPP_MODULE(mod) {
    function( "norm", &norm );
}
```

The code creates an Rcpp module called `mod` that exposes the `norm` function. **Rcpp** automatically deduces the conversions that are needed for input and output. This alleviates the need for a wrapper function using either **Rcpp** or the R API.

On the R side, the module is retrieved by using the `Module` function from **Rcpp**

```
> inc <- '
using namespace Rcpp;

double norm( double x, double y ) {
    return sqrt( x*x + y*y );
}

RCPP_MODULE(mod) {
    function( "norm", &norm );
}
'
> fx <- cxxfunction(signature(), plugin="Rcpp", include=inc)
> mod <- Module("mod", getDynLib(fx))
```

Note that this example assumed that the previous code segment defining the module was returned by the `cxxfunction()` (from the **inline** package) as callable R function `fx` from which we can extract the relevant pointer using `getDynLib()`. In the case of using Rcpp modules via a package (which is detailed in Section 3 below), modules are actually loaded differently and we would have used

```
> require(nameOfMyModulePackage)
> mod <- new( mod )
> mod$norm( 3, 4 )
```

where the module is loaded upon startup and we use the constructor directly. More details on this aspect follow below.

A module can contain any number of calls to `function` to register many internal functions to R. For example, these 6 functions :

```
std::string hello() {
    return "hello";
}

int bar( int x) {
    return x*2;
}

double foo( int x, double y) {
    return x * y;
}

void bla( ) {
    Rprintf( "hello\\n" );
}

void bla1( int x) {
    Rprintf( "hello (x = %d)\\n", x );
}

void bla2( int x, double y) {
    Rprintf( "hello (x = %d, y = %5.2f)\\n", x, y );
}
```

can be exposed with the following minimal code:

```
RCPP_MODULE(yada) {
    using namespace Rcpp;

    function("hello" , &hello);
    function("bar"   , &bar  );
    function("foo"   , &foo  );
    function("bla"   , &bla  );
    function("bla1"  , &bla1 );
    function("bla2"  , &bla2 );
}
```

which can then be used from R:

```
> require( Rcpp )
> yd <- Module("yada", getDynLib(fx))
> yd$bar(2L)
> yd$foo(2L, 10.0)
> yd$hello()
> yd$bla()
> yd$bla1(2L)
> yd$bla2(2L, 5.0)
```

In the case of a package (as for example the one created by `Rcpp.package.skeleton()` with argument `module=TRUE`;

more on that below), we can use

```
> require(myModulePackage)
> bar(2L)
> foo(2L, 10.0)
> hello()
> bla()
> bla1(2L)
> bla2(2L, 5.0)
```

The requirements for a function to be exposed to R via Rcpp modules are:

- The function takes between 0 and 65 parameters.
- The type of each input parameter must be manageable by the `Rcpp::as` template.
- The return type of the function must be either `void` or any type that can be managed by the `Rcpp::wrap` template.
- The function name itself has to be unique in the module. In other words, no two functions with the same name but different signatures are allowed. C++ allows overloading functions. This might be added in future versions of modules.

### 2.1.1 Documentation for exposed functions using Rcpp modules

In addition to the name of the function and the function pointer, it is possible to pass a short description of the function as the third parameter of `function`.

```
using namespace Rcpp;

double norm( double x, double y ) {
    return sqrt( x*x + y*y );
}

RCPP_MODULE(mod) {
    function( "norm", &norm, "Provides a simple vector norm" );
}
```

The description is used when displaying the function to the R prompt:

```
> mod <- Module("mod", getDynLib(fx))
> show(mod$norm)
```

### 2.1.2 Formal arguments specification

`function` also gives the possibility to specify the formal arguments of the R function that encapsulates the C++ function, by passing a `Rcpp::List` after the function pointer.

```cpp
using namespace Rcpp;

double norm( double x, double y ) {
    return sqrt( x*x + y*y );
}

RCPP_MODULE(mod_formals) {
    function("norm",
             &norm,
             List::create( _["x"] = 0.0, _["y"] = 0.0 ),
             "Provides a simple vector norm");
}
```

A simple usage example is provided below:

```r
> norm <- mod$norm
> norm()
> norm( y = 2 )
> norm( x = 2, y = 3 )
> args( norm )
```

To set formal arguments without default values, simply omit the rhs.

```cpp
using namespace Rcpp;

double norm( double x, double y ) {
    return sqrt( x*x + y*y );
}

RCPP_MODULE(mod_formals2) {
    function("norm", &norm,
             List::create( _["x"], _["y"] = 0.0 ),
             "Provides a simple vector norm");
}
```

This can be used as follows:

```r
> norm <- mod$norm
> args(norm)
```

The ellipsis (...) can be used to denote that additional arguments are optional; it does not take a default value.

```cpp
using namespace Rcpp;

double norm( double x, double y ) {
    return sqrt( x*x + y*y );
}

RCPP_MODULE(mod_formals3) {
    function( "norm", &norm,
             List::create( _["x"], _["..."] ),
             "documentation for norm");
}
```

```
> norm <- mod$norm
> args( norm )
```

## 2.2 Exposing C++ classes using Rcpp modules

Rcpp modules also provide a mechanism for exposing C++ classes, based on the reference classes introduced in R 2.12.0.

### 2.2.1 Initial example

A class is exposed using the `class_` keyword. The `Uniform` class may be exposed to R as follows:

```
using namespace Rcpp;
class Uniform {
public:
    Uniform(double min_, double max_) : min(min_), max(max_) {}

    NumericVector draw(int n) const {
        RNGScope scope;
        return runif( n, min, max );
    }

    double min, max;
};

double uniformRange( Uniform* w) {
    return w->max - w->min;
}

RCPP_MODULE(unif_module) {

    class_<Uniform>( "Uniform" )

    .constructor<double,double>()

    .field( "min", &Uniform::min )
    .field( "max", &Uniform::max )

    .method( "draw", &Uniform::draw )
    .method( "range", &uniformRange )
    ;

}
```

```
> unif_module <- Module( "unif_module", getDynLib(fx_unif ) )
> Uniform <- unif_module$Uniform
> u <- new( Uniform, 0, 10 )
> u$draw( 10L )
> u$range()
> u$max <- 1
> u$range()
> u$draw( 10 )
```

`class_` is templated by the C++ class or struct that is to be exposed to R. The parameter of the `class_<Uniform>` constructor is the name we will use on the R side. It usually makes sense to use the same name as the class name. While this is not enforced, it might be useful when exposing a class generated from a template.

Then constructors, fields and methods are exposed.

### 2.2.2 Exposing constructors using Rcpp modules

Public constructors that take from 0 and 6 parameters can be exposed to the R level using the `.constuctor` template method of `.class_`.

Optionally, `.constructor` can take a description as the first argument.

```
    .constructor<double,double>("sets the min and max value of the distribution")
```

Also, the second argument can be a function pointer (called validator) matching the following type :

```
typedef bool (*ValidConstructor)(SEXP*,int);
```

The validator can be used to implement dispatch to the appropriate constructor, when multiple constructors taking the same number of arguments are exposed. The default validator always accepts the constructor as valid if it is passed the appropriate number of arguments. For example, with the call above, the default validator accepts any call from R with two `double` arguments (or arguments that can be cast to `double`).

TODO: include validator example here

### 2.2.3 Exposing fields and properties

`class_` has three ways to expose fields and properties, as illustrated in the example below :

```
using namespace Rcpp;
class Foo {
public:
    Foo(double x_, double y_, double z_ ):
        x(x_), y(y_), z(z_) {}

    double x;
    double y;

    double get_z() { return z; }
    void set_z( double z_ ) { z = z_; }

private:
    double z;
};

RCPP_MODULE(mod_foo) {
    class_<Foo>( "Foo" )

    .constructor<double,double,double>()

    .field( "x", &Foo::x )
    .field_readonly( "y", &Foo::y )

    .property( "z", &Foo::get_z, &Foo::set_z )
    ;
}
```

The `.field` method exposes a public field with read/write access from R. `field` accepts an extra parameter to give a short description of the field:

```
    .field( "x", &Foo::x, "documentation for x" )
```

The `.field_readonly` exposes a public field with read-only access from R. It also accepts the description of the field.

```
    .field_readonly( "y", &Foo::y, "documentation for y" )
```

The `.property` method allows indirect access to fields through a getter and a setter. The setter is optional, and the property is considered read-only if the setter is not supplied. A description of the property is also allowed:

```
    // with getter and setter
    .property( "z", &Foo::get_z, &Foo::set_z, "Documentation for z" )

    // with only getter
    .property( "z", &Foo::get_z, "Documentation for z" )
```

The type of the field (**T**) is deduced from the return type of the getter, and if a setter is given its unique parameter should be of the same type.

Getters can be member functions taking no parameter and returning a **T** (for example `get_z` above), or a free function taking a pointer to the exposed class and returning a **T**, for example:

```
double z_get( Foo* foo ) { return foo->get_z(); }
```

Setters can be either a member function taking a `T` and returning void, such as `set_z` above, or a free function taking a pointer to the target class and a **T** :

```
void z_set( Foo* foo, double z ) { foo->set_z(z); }
```

Using properties gives more flexibility in case field access has to be tracked or has impact on other fields. For example, this class keeps track of how many times the `x` field is read and written.

```
class Bar {
public:

    Bar(double x_) : x(x_), nread(0), nwrite(0) {}

    double get_x( ) {
        nread++;
        return x;
    }

    void set_x( double x_) {
        nwrite++;
        x = x_;
    }

    IntegerVector stats() const {
        return IntegerVector::create(_["read"] = nread,
                                     _["write"] = nwrite);
    }

private:
    double x;
    int nread, nwrite;
};

RCPP_MODULE(mod_bar) {
    class_<Bar>( "Bar" )

    .constructor<double>()

    .property( "x", &Bar::get_x, &Bar::set_x )
    .method( "stats", &Bar::stats )
    ;
}
```

Here is a simple usage example:

```
> Bar <- mod_bar$Bar
> b <- new( Bar, 10 )
> b$x + b$x
> b$stats()
> b$x <- 10
> b$stats()
```

### 2.2.4 Exposing methods using Rcpp modules

`class_` has several overloaded and templated `.method` functions allowing the programmer to expose a method associated with the class.

A legitimate method to be exposed by `.method` can be:

- A public member function of the class, either const or non const, that returns void or any type that can be handled by `Rcpp::wrap`, and that takes between 0 and 65 parameters whose types can be handled by `Rcpp::as`.
- A free function that takes a pointer to the target class as its first parameter, followed by 0 or more (up to 65) parameters that can be handled by `Rcpp::as` and returning a type that can be handled by `Rcpp::wrap` or void.

**Documenting methods** `.method` can also include a short documentation of the method, after the method (or free function) pointer.

```
.method("stats", &Bar::stats,
        "vector indicating the number of times x has been read and written" )
```

TODO: mention overloading, need good example.

**Const and non-const member functions** `method` is able to expose both `const` and `non const` member functions of a class. There are however situations where a class defines two versions of the same method, differing only in their signature by the `const`-ness. It is for example the case of the member functions `back` of the `std::vector` template from the STL.

```
reference back ( );
const_reference back ( ) const;
```

To resolve the ambiguity, it is possible to use `const_method` or `nonconst_method` instead of `method` in order to restrict the candidate methods.

**Special methods** **Rcpp** considers the methods `[[` and `[[<-` special, and promotes them to indexing methods on the R side.

### 2.2.5 Object finalizers

The `.finalizer` member function of `class_` can be used to register a finalizer. A finalizer is a free function that takes a pointer to the target class and return `void`. The finalizer is called before the destructor and so operates on a valid object of the target class.

It can be used to perform operations, releasing resources, etc ...

The finalizer is called automatically when the R object that encapsulates the C++ object is garbage collected.

### 2.2.6 S4 dispatch

When a C++ class is exposed by the `class_` template, a new S4 class is registered as well. The name of the S4 class is obfuscated in order to avoid name clashes (i.e. two modules exposing the same class).

This allows implementation of R-level (S4) dispatch. For example, one might implement the `show` method for C++ `World` objects:

```
> setMethod( "show", yada$World , function(object) {
+     msg <- paste( "World object with message : ", object$greet() )
+     writeLines( msg )
+ } )
```

TODO: mention R inheritance (John ?)

### 2.2.7 Full example

The following example illustrates how to use Rcpp modules to expose the class `std::vector<double>` from the STL.

```
typedef std::vector<double> vec;    // convenience typedef
void vec_assign( vec* obj, Rcpp::NumericVector data ) {   // helpers
    obj->assign( data.begin(), data.end() );
}
void vec_insert( vec* obj, int position, Rcpp::NumericVector data) {
    vec::iterator it = obj->begin() + position;
    obj->insert( it, data.begin(), data.end() );
}
Rcpp::NumericVector vec_asR( vec* obj ) { return Rcpp::wrap( *obj ); }
void vec_set( vec* obj, int i, double value ) { obj->at( i ) = value; }

RCPP_MODULE(mod_vec) {
    using namespace Rcpp;

    // we expose the class std::vector<double> as "vec" on the R side
    class_<vec>( "vec")

    // exposing constructors
    .constructor()
    .constructor<int>()

    // exposing member functions
    .method( "size", &vec::size)
    .method( "max_size", &vec::max_size)
    .method( "resize", &vec::resize)
    .method( "capacity", &vec::capacity)
    .method( "empty", &vec::empty)
    .method( "reserve", &vec::reserve)
    .method( "push_back", &vec::push_back )
    .method( "pop_back", &vec::pop_back )
    .method( "clear", &vec::clear )

    // specifically exposing const member functions
    .const_method( "back", &vec::back )
    .const_method( "front", &vec::front )
    .const_method( "at", &vec::at )

    // exposing free functions taking a std::vector<double>*
    // as their first argument
    .method( "assign", &vec_assign )
    .method( "insert", &vec_insert )
    .method( "as.vector", &vec_asR )

    // special methods for indexing
    .const_method( "[[", &vec::at )
    .method( "[[<-", &vec_set )
    ;
}
```

```
> mod_vec <- Module( "mod_vec", getDynLib(fx_vec), mustStart = TRUE )
> vec <- mod_vec$vec
> v <- new( vec )
> v$reserve( 50L )
> v$assign( 1:10 )
> v$push_back( 10 )
> v$size()
> v$capacity()
> v[[ 0L ]]
> v$as.vector()
```

# 3 Using modules in other packages

## 3.1 Namespace import/export

### 3.1.1 Import all functions and classes

When using **Rcpp** modules in a packages, the client package needs to import **Rcpp**'s namespace. This is achieved by adding the following line to the `NAMESPACE` file.

```
import( Rcpp )
```

The simplest way to load all functions and classes from a module directly into a package namespace is to use the `loadRcppModules` function within the `.onLoad` body.

```
.onLoad <- function(libname, pkgname) {
    loadRcppModules()
}
```

This will look in the package's DESCRIPTION file for the `RcppModules` field, load each declared module and populate their contents into the package's namespace. For example, both the **testRcppModule** package (which is part of large unit test suite for **Rcpp**) and the package created via `Rcpp.package.skeleton("somename", module=TRUE)` have this declaration:

```
RcppModules: yada, stdVector, NumEx
```

The `loadRcppModules` function has a single argument `direct` with a default value of `TRUE`. With this default value, all content from the module is exposed directly in the package namespace. If set to `FALSE`, all content is exposed as components of the module.

Starting with release 0.9.11, an alternative is provided by the `loadModule()` function which takes the module name as an argument. It can be placed in any `.R` file in the package. This is useful as it allows to load the module from the same file as some auxiliary R functions using the module. For the example module, the equivalent code to the `.onLoad()` use shown above then becomes

```
loadModule("yada")
loadModule("stdVector")
loadModule("NumEx")
```

This feature is also used in the new Rcpp Classes introduced with Rcpp 0.9.11.

### 3.1.2 Just expose the module

Alternatively, it is possible to just expose the module to the user of the package, and let them extract the functions and classes as needed. This uses lazy loading so that the module is only loaded the first time the user attempts to extract a function or a class with the dollar extractor.

```
yada <- Module( "yada" )
.onLoad <- function(libname, pkgname) {
    # placeholder
}
```

### 3.2 Support for modules in skeleton generator

The `Rcpp.package.skeleton` function has been improved to help **Rcpp** modules. When the `module` argument is set to `TRUE`, the skeleton generator installs code that uses a simple module.

```
> Rcpp.package.skeleton( "testmod", module = TRUE )
```

Creating a new package using *Rcpp modules* is easiest via the call to `Rcpp.package.skeleton()` with argument `module=TRUE` as a working package with three example Modules results.

### 3.3 Module documentation

**Rcpp** defines a `prompt` method for the `Module` class, allowing generation of a skeleton of an Rd file containing some information about the module.

```
> yada <- Module( "yada" )
> prompt( yada, "yada-module.Rd" )
```

We strongly recommend using a package when working with Modules. But in case a manually compiled shared library has to loaded, the return argument of the `getDynLib()` function can be supplied as the `PACKAGE` argument to the `Module()` function as well.

## 4 Future extensions

`Boost.Python` has many more features that we would like to port to Rcpp modules : class inheritance, default arguments, enum types, ...

## 5 Known shortcomings

There are some things *Rcpp modules* is not good at:

- serialization and deserialization of objects: modules are implemented via an external pointer using a memory location, which is non-constant and varies between session. Objects have to be re-created, which is different from the (de-)serialization that R offers. So these objects cannot be save from session to session.
- mulitple inheritance: currently, only simple class structures are representable via *Rcpp modules*.

## 6 Summary

This note introduced *Rcpp modules* and illustrated how to expose C++ function and classes more easily to R. We hope that R and C++ programmers find *Rcpp modules* useful.

## References


David Abrahams and Ralf W. Grosse-Kunstleve. *Building Hybrid Systems with Boost.Python*. Boost Consulting, 2003. URL `http://www.boostpro.com/writing/bpl.pdf`.

J. J. Allaire, Dirk Eddelbuettel, and Romain François. *Rcpp Attributes*, 2013. URL `http://CRAN.R-Project.org/package=Rcpp`. Vignette included in R package Rcpp.

Dirk Eddelbuettel and Romain François. Rcpp: Seamless R and C++ integration. *Journal of Statistical Software*, 40(8): 1–18, 2011. URL `http://www.jstatsoft.org/v40/i08/`.

Dirk Eddelbuettel, Romain François, JJ Allaire, John Chambers, Douglas Bates, and Kevin Ushey. *Rcpp: Seamless R and C++ Integration*, 2015. URL `http://CRAN.R-Project.org/package=Rcpp`. R package version 0.11.4.

R Development Core Team. *Writing R extensions*. R Foundation for Statistical Computing, Vienna, Austria, 2012. URL `http://CRAN.R-Project.org/doc/manuals/R-exts.html`.
