# Supplementary material for: Comparing Kaolin and Pinolene to Improve Sustainable Grapevine Production during Drought
Source: PLoS One. 2016 Jun 13;11(6):e0156631. doi: 10.1371/journal.pone.0156631 (PMC4905681; doi:10.1371/journal.pone.0156631)
Supplement: S1 File — (GZ) [file pone.0156631.s003.gz › PONE_Brillante_et_al_2016_Code_and_Data/packrat/src/Rcpp/Rcpp_0.11.6.tar.gz!/Rcpp/build/Rcpp.pdf]

Package ‘Rcpp’

May 1, 2015

Title Seamless R and C++ Integration

Version 0.11.6

Date 2015-05-01

Author Dirk Eddelbuettel, Romain Francois, JJ Allaire, Kevin Ushey, Douglas Bates, and John Chambers

Maintainer Dirk Eddelbuettel <edd@debian.org>

Description The 'Rcpp' package provides R functions as well as C++ classes which offer a seamless integration of R and C++. Many R data types and objects can be mapped back and forth to C++ equivalents which facilitates both writing of new code as well as easier integration of third-party libraries. Documentation about 'Rcpp' is provided by several vignettes included in this package, via the 'Rcpp Gallery' site at <http://gallery.rcpp.org>, the paper by Eddelbuettel and Francois (2011, JSS), and the book by Eddelbuettel (2013, Springer); see 'citation(``Rcpp'')' for details on these last two.

Depends R (>= 3.0.0)

Imports methods

Suggests RUnit, inline, rbenchmark, highlight, pkgKitten (>= 0.1.2)

VignetteBuilder highlight

URL http://www.rcpp.org, http://dirk.eddelbuettel.com/code/rcpp.html, https://github.com/RcppCore/Rcpp

License GPL (>= 2)

BugReports https://github.com/RcppCore/Rcpp/issues

MailingList
Please send questions and comments regarding Rcpp to rcpp-devel@lists.r-forge.r-project.org

R topics documented:

Rcpp-package 2
.DollarNames-methods 3
C++Class-class 4
C++Constructor-class 4

C++Field-class 5
C++Function-class 6
C++Object-class 6
C++OverloadedMethods-class 7
compileAttributes 7
cppFunction 8
demangle 10
dependsAttribute 12
evalCpp 13
exportAttribute 14
exposeClass 15
formals<–methods 19
interfacesAttribute 19
LdFlags 21
loadModule 21
loadRcppModules 23
Module 23
Module-class 24
pluginsAttribute 25
populate 26
Rcpp.package.skeleton 26
Rcpp.plugin.maker 28
RcppUnitTests 29
registerPlugin 30
setRcppClass 30
sourceCpp 32

Index **35**

`Rcpp-package` *R / C++ interface*

Description

The **Rcpp** package provides C++ classes that greatly facilitate interfacing C or C++ code in R packages using the `.Call` interface provided by R.

Introduction

Rcpp provides C++ classes to facilitate manipulation of a large number of R data structures : vectors, functions, environments, ...

The "Rcpp-introduction" vignette gives an introduction on the package

Usage for package building

The "Rcpp-package" vignette documents how to use Rcpp in client packages.

History

The initial versions of Rcpp were written by Dominick Samperi during 2005 and 2006.

Dirk Eddelbuettel made some additions, and became maintainer in 2008.

Dirk Eddelbuettel and Romain Francois have been extending Rcpp since 2009.

Author(s)

Dirk Eddelbuettel and Romain Francois

References

Dirk Eddelbuettel and Romain Francois (2011). **Rcpp**: Seamless R and C++ Integration. *Journal of Statistical Software*, **40(8)**, 1-18. URL http://www.jstatsoft.org/v40/i08/ and available as `vignette("Rcpp-introduction")`.

Eddelbuettel, Dirk (2013) Seamless R and C++ Integration with **Rcpp**. Springer, New York. ISBN 978-1-4614-6867-7.

See Also

Development for **Rcpp** can be followed via the GitHub repository at http://github.com/RcppCore/Rcpp.

Extensive examples with full documentation are available at http://gallery.rcpp.org.

Examples

```
## Not run:
# introduction to Rcpp
vignette("Rcpp-introduction")

# information on how to build a package that uses Rcpp
vignette("Rcpp-package")

## End(Not run)
```

`.DollarNames-methods` *completion*

Description

completion

Methods

`signature(x = "ANY")`

`signature(x = "C++Object")` completes fields and methods of C++ objects

`signature(x = "Module")` completes functions and classes of modules

`C++Class-class` *Reflection information for an internal c++ class*

Description

Information about an internal c++ class.

Objects from the Class

Objects are usually extracted from a Module using the dollar extractor.

Slots

`.Data`: mangled name of the class

`pointer`: external pointer to the internal infomation

`module`: external pointer to the module

`fields`: list of C++Field objects

`constructors`: list of C++Constructor objects

`methods`: list of C++OverloadedMethods objects

generator the generator object for the class

docstring description of the class

typeid unmangled typeid of the class

enums enums of the class

parents names of the parent classes of this class

Methods

show `signature(object = "C++Class")`: prints the class.

$ `signature(object = "C++Class")`: ...

`C++Constructor-class` *Class "C++Constructor"*

Description

Representation of a C++ constructor

Extends

Class "`envRefClass`", directly. Class "`.environment`", by class "envRefClass", distance 2. Class "`refClass`", by class "envRefClass", distance 2. Class "`environment`", by class "envRefClass", distance 3, with explicit coerce. Class "`refObject`", by class "envRefClass", distance 3.

Fields

`pointer`: pointer to the internal structure that represent the constructor

`class_pointer`: pointer to the internal structure that represent the associated C++ class

`nargs`: Number of arguments the constructor expects

`signature`: C++ signature of the constructor

`docstring`: Short description of the constructor

C++Field-class *Class "C++Field"*

Description

Metadata associated with a field of a class exposed through Rcpp modules

Fields

`pointer`: external pointer to the internal (C++) object that represents fields

`cpp_class`: (demangled) name of the C++ class of the field

`read_only`: Is this field read only

`class_pointer`: external pointer to the class this field is from.

Methods

No methods defined with class "C++Field" in the signature.

See Also

The `fields` slot of the `C++Class` class is a list of `C++Field` objects

Examples

```
showClass("C++Field")
```

C++Function-class *Class "C++Function"*

Description

Internal C++ function

Objects from the Class

Objects can be created by the `Rcpp::InternalFunction` class from the `Rcpp` library

Slots

`.Data`**:** R function that calls back to the internal function

`pointer`**:** External pointer to a C++ object poiting to the function

`docstring`**:** Short documentation for the function

`signature`**:** C++ signature

Extends

Class "`function`", from data part. Class "`OptionalFunction`", by class "function", distance 2. Class "`PossibleMethod`", by class "function", distance 2.

Methods

show `signature(object = "C++Function")`: print the object

Examples

```
showClass("C++Function")
```

C++Object-class *c++ internal objects*

Description

C++ internal objects instanciated from a class exposed in an Rcpp module

Objects from the Class

This is a virtual class. Actual C++ classes are subclasses.

Methods

$ `signature(x = "C++Object")`: invokes a method on the object, or retrieves the value of a property

$<- `signature(x = "C++Object")`: set the value of a property

show `signature(object = "C++Object")`: print the object

`C++OverloadedMethods-class` *Class "C++OverloadedMethods"*

Description

Set of C++ methods

Extends

Class "`envRefClass`", directly. Class "`.environment`", by class "envRefClass", distance 2. Class "`refClass`", by class "envRefClass", distance 2. Class "`environment`", by class "envRefClass", distance 3, with explicit coerce. Class "`refObject`", by class "envRefClass", distance 3.

Fields

`pointer`: Object of class `externalptr` pointer to the internal structure that represents the set of methods

`class_pointer`: Object of class `externalptr` pointer to the internal structure that models the related class

---

`compileAttributes` *Compile Rcpp Attributes for a Package*

Description

Scan the source files within a package for attributes and generate code as required. Generates the bindings required to call C++ functions from R for functions adorned with the `Rcpp::export` attribute.

Usage

```
compileAttributes(pkgdir = ".", verbose = getOption("verbose"))
```

Arguments

`pkgdir`	Directory containing the package to compile attributes for (defaults to the current working directory).
`verbose`	`TRUE` to print detailed information about generated code to the console.

Details

The source files in the package directory given by `pkgdir` are scanned for attributes and code is generated as required based on the attributes.

For C++ functions adorned with the `Rcpp::export` attribute, the C++ and R source code required to bind to the function from R is generated and added (respectively) to `src/RcppExports.cpp` or `R/RcppExports.R`.

In order to access the declarations for custom `Rcpp::as` and `Rcpp::wrap` handlers the `compileAttributes` function will also call any inline plugins available for packages listed in the `LinkingTo` field of the `DESCRIPTION` file.

Value

Returns (invisibly) a character vector with the paths to any files that were updated as a result of the call.

Note

The `compileAttributes` function deals only with exporting C++ functions to R. If you want the functions to additionally be publicly available from your package's namespace another step may be required. Specifically, if your package `NAMESPACE` file does not use a pattern to export functions then you should add an explicit entry to `NAMESPACE` for each R function you want publicly available.

In addition to exporting R bindings for C++ functions, the `compileAttributes` function can also generate a direct C++ interface to the functions using the `Rcpp::interfaces` attribute.

See Also

`Rcpp::export`, `Rcpp::interfaces`

Examples

```
## Not run:

# Compile attributes for package in the current working dir
compileAttributes()

## End(Not run)
```

`cppFunction` *Define an R Function with a C++ Implementation*

Description

Dynamically define an R function with C++ source code. Compiles and links a shared library with bindings to the C++ function then defines an R function that uses `.Call` to invoke the library.

Usage

```
cppFunction(code, depends = character(), plugins = character(),
            includes = character(), env = parent.frame(), rebuild = FALSE,
            showOutput = verbose, verbose = getOption("verbose"))
```

Arguments

`code`	Source code for the function definition.
`depends`	Character vector of packages that the compilation depends on. Each package listed will first be queried for an inline plugin to determine header files to include. If no plugin is defined for the package then a header file based the package's name (e.g. `PkgName.h`) will be included.
`plugins`	Character vector of inline plugins to use for the compliation.
`includes`	Character vector of user includes (inserted after the includes provided by `depends`).
`env`	The environment in which to define the R function. May be `NULL` in which case the defined function can be obtained from the return value of `cppFunction`.
`rebuild`	Force a rebuild of the shared library.
`showOutput`	`TRUE` to print `R CMD SHLIB` output to the console.
`verbose`	`TRUE` to print detailed information about generated code to the console.

Details

Functions defined using `cppFunction` must have return types that are compatible with `Rcpp::wrap` and parameter types that are compatible with `Rcpp::as`.

The shared library will not be rebuilt if the underlying code has not changed since the last compilation.

Value

An R function that uses `.Call` to invoke the underlying C++ function.

Note

You can also define R functions with C++ implementations using the `sourceCpp` function, which allows you to separate the C++ code into it's own source file. For many use cases this is an easier and more maintainable approach.

See Also

`sourceCpp`, `evalCpp`

Examples

```
## Not run:

cppFunction(
    'int fibonacci(const int x) {
        if (x == 0) return(0);
        if (x == 1) return(1);
        return (fibonacci(x - 1)) + fibonacci(x - 2);
    }')

cppFunction(depends = "RcppArmadillo",
    'List fastLm(NumericVector yr, NumericMatrix Xr) {

        int n = Xr.nrow(), k = Xr.ncol();

        arma::mat X(Xr.begin(), n, k, false);
        arma::colvec y(yr.begin(), yr.size(), false);

        arma::colvec coef = arma::solve(X, y);
        arma::colvec resid = y - X*coef;

        double sig2 = arma::as_scalar(arma::trans(resid)*resid/(n-k) );
        arma::colvec stderrest = arma::sqrt(
            sig2 * arma::diagvec(arma::inv(arma::trans(X)*X)));

        return List::create(Named("coefficients") = coef,
            Named("stderr")       = stderrest
        );
    }')

cppFunction(plugins=c("cpp11"), '
    int useCpp11() {
        auto x = 10;
        return x;
    }')


## End(Not run)
```

---

demangle *c++ type information*

Description

`demangle` gives the demangled type, `sizeof` its size (in bytes).

Usage

```
demangle(type = "int", ...)
sizeof(type = "int", ...)
```

Arguments

`type`	The type we want to demangle
`...`	Further argument for `cppFunction`

Details

The following function is compiled and invoked:

```
SEXP demangle_this_type(){
    typedef
    return wrap( DEMANGLE(type) ) ;
}

SEXP sizeof_this_type(){
    typedef
    return wrap( sizeof(type) ) ;
}
```

`DEMANGLE` is a macro in ‘Rcpp’ that does the work.

Value

The demangled type, as a string.

Note

We only know how to demangle with gcc. If you know how to demangle types with your compiler, let us know.

Author(s)

Romain Francois <romain@r-enthusiasts.com>

References

See this chapter from the GNU C++ library manual.

See Also

`cppFunction` is used to compile the function `demangle` creates.

Examples

```
## Not run:
    demangle( "int64_t" )
    demangle( "uint64_t" )

    demangle( "NumericVector" )
```

```
    demangle( "std::map<std::string,double>" )

    sizeof( "long" )
    sizeof( "long long" )


## End(Not run)
```

---

dependsAttribute *Rcpp::depends Attribute*

Description

The Rcpp::depends attribute is added to a C++ source file to indicate that it has a compilation dependency on one or more other packages. For example:

```
// [[Rcpp::depends(RcppArmadillo)]]
```

Arguments

...	Packages which the source file depends on for compilation

Details

The Rcpp::depends attribute is used by the implementation of the sourceCpp function to correctly setup the build environment for R CMD SHLIB.

The include directories of the specified packages are added to the CLINK_CPPFLAGS environment variable. In addition, if the referenced package provides an inline plugin it is called to determine additional environment variables required to successfully build.

Note

The Rcpp::depends attribute is specified using a syntax compatible with the new generalized attributes feature of the C++11 standard. Note however that since this feature is not yet broadly supported by compilers it needs to be specified within a comment (see examples below).

See Also

sourceCpp

Examples

```
## Not run:

// [[Rcpp::depends(RcppArmadillo)]]

// [[Rcpp::depends(Matrix, RcppGSL)]]

## End(Not run)
```

evalCpp *Evaluate a C++ Expression*

Description

Evaluates a C++ expression. This creates a C++ function using cppFunction and calls it to get the result.

Usage

```
evalCpp(code, depends = character(), includes = character(),
        rebuild = FALSE, showOutput = verbose,
        verbose = getOption("verbose"))
areMacrosDefined(names, depends = character(), includes = character(),
        rebuild = FALSE, showOutput = verbose,
        verbose = getOption("verbose"))
```

Arguments

code	C++ expression to evaluate
names	names of the macros we want to test
depends	see cppFunction
includes	see cppFunction
rebuild	see cppFunction
showOutput	see cppFunction
verbose	see cppFunction

Value

The result of the evaluated C++ expression.

Note

The result type of the C++ expression must be compatible with `Rcpp::wrap`.

See Also

sourceCpp, cppFunction

Examples

```
## Not run:

evalCpp( "__cplusplus" )
evalCpp( "std::numeric_limits<double>::max()" )
```

```
areMacrosDefined( c("__cplusplus", "HAS_TR1" ) )

## End(Not run)
```

`exportAttribute` *Rcpp::export Attribute*

Description

The `Rcpp::export` attribute is added to a C++ function definition to indicate that it should be made available as an R function. The `sourceCpp` and `compileAttributes` functions process the `Rcpp::export` attribute by generating the code required to call the C++ function from R.

Arguments

`name`	Specify an alternate name for the generated R function (optional, defaults to the name of the C++ function if not specified).

Details

Functions marked with the `Rcpp::export` attribute must meet several conditions to be correctly handled:

1. Be defined in the global namespace (i.e. not within a C++ `namespace` declaration).
2. Have a return type that is either void or compatible with `Rcpp::wrap` and parameter types that are compatible with `Rcpp::as` (see sections 3.1 and 3.2 of the *Rcpp-introduction* vignette for more details).
3. Use fully qualified type names for the return value and all parameters. However, Rcpp types may appear without the namespace qualifier (i.e. `DataFrame` is okay as a type name but `std::string` must be specified fully).

If default argument values are provided in the C++ function definition then these defaults are also used for the exported R function. For example, the following C++ function:

```
DataFrame readData(
    CharacterVector file,
    CharacterVector exclude = CharacterVector::create(),
    bool fill = true)
```

Will be exported to R as:

```
function (file, exclude = character(0), fill = TRUE)
```

Note that C++ rules for default arguments still apply: they must occur consecutively at the end of the function signature and unlike R can't rely on the values of other arguments.

Note

When a C++ function has export bindings automatically generated by the `compileAttributes` function, it can optionally also have a direct C++ interface generated using the `Rcpp::interfaces` attribute.

The `Rcpp::export` attribute is specified using a syntax compatible with the new generalized attributes feature of the C++11 standard. Note however that since this feature is not yet broadly supported by compilers it needs to be specified within a comment (see examples below).

See Also

`sourceCpp` and `compileAttributes`

Examples

```
## Not run:

#include <Rcpp.h>

using namespace Rcpp;

// [[Rcpp::export]]
int fibonacci(const int x) {

   if (x == 0) return(0);
   if (x == 1) return(1);

   return (fibonacci(x - 1)) + fibonacci(x - 2);
}

// [[Rcpp::export("convolveCpp")]]
NumericVector convolve(NumericVector a, NumericVector b) {

   int na = a.size(), nb = b.size();
   int nab = na + nb - 1;
   NumericVector xab(nab);

   for (int i = 0; i < na; i++)
      for (int j = 0; j < nb; j++)
         xab[i + j] += a[i] * b[j];

   return xab;
}

## End(Not run)
```

`exposeClass` *Create an Rcpp Module to Expose a C++ Class in R*

Description

The arguments specify a C++ class and some combination of constructors, fields and methods to be shared with R by creating a corresponding reference class in R. The information needed in the call to `exposeClass()` is the simplest possible in order to create a C++ module for the class; for example, fields and methods in this class need only be identified by their name. Inherited fields and methods can also be included, but more information is needed. The function writes a C++ source file, containing a module definition to expose the class to R, plus one line of R source to create the corresponding reference class.

Usage

```
exposeClass(class, constructors = , fields = , methods = , file = ,
    header = , module = , CppClass = class, readOnly = , rename = ,
    Rfile = TRUE)
```

Arguments

`class` The name of the class in R. By default, this will be the same as the name of the class in C++, unless argument `CppClass` is supplied.

`constructors` A list of the signatures for any of the class constructors to be called from R. Each element of the list gives the data types in C++ for the arguments to the corresponding constructor. See Details and the example.

`fields, methods` The vector of names for the fields and for the methods to be exposed in R. For inherited fields and methods, type information needs to be supplied; see the section "Inherited Fields and Methods".

`file` Usually, the name for the file on which to write the C++ code, by default `paste0(CppClass, "Module.cp`
If the current working directory in R is the top-level directory for a package, the function writes the file in the `"src"` subdirectory. Otherwise the file is written in the working directory.

The argument may also be a connection, already open for writing.

`header` Whatever lines of C++ header information are needed to include the definition of the class. Typically this includes a file from the package where we are writing the module definition, as in the example below.

`module` The name for the Rcpp module, by default `paste0("class_",CppClass)`.

`CppClass` The name for the class in C++. By default and usually, the intended class name in R.

`readOnly` Optional vector of field names. These fields will be created as read-only in the interface.

`rename` Optional named character vector, used to name fields or methods differently in R from their C++ name. The elements of the vector are the C++ names and the corresponding elements of `names(rename)` the desired names in R. So `c(.age = "age")` renames the C++ field or method `age` as `.age`.

`Rfile` Controls the writing of a one-line R command to create the reference class corresponding to the C++ module information. By default, this will be a file `paste0(class, "Class.R")`. If the working directory is an R package source

directory, the file will be written in the `R` subdirectory, otherwise in the working directory itself.

Supplying a character string substitutes that file name for the default.

The argument may also be a connection open for writing or `FALSE` to suppress writing the R source altogether.

Details

The file created by the call to these functions only depends on the information in the C++ class supplied. This file is intended to be part of the C++ source for an R package. The file only needs to modified when the information changes, either because the class has changed or because you want to expose different information to R. In that case you can either recall `exposeClass()` or edit the C++ file created.

The Rcpp Module mechanism has a number of other optional techniques, not covered by `exposeClass()`. These should be entered into the C++ file created. See the "rcpp-modules" vignette with the package for current possibilities.

For fields and methods specified directly in the C++ class, the fields and method arguments to `exposeClass()` are character vectors naming the corresponding members of the class. For module construction, the data types of directly specified fields and of the arguments for the methods are not needed.

For *inherited* fields or methods, data type information is needed. See the section "Inherited Fields and Methods".

For exposing class constructors, the module needs to know the signatures of the constructors to be exposed; each signature is a character vector of the corresponding C++ data types.

Value

Nothing, called for its side effect.

Inherited Fields and Methods

If the C++ class inherits from one or more other classes, the standard Rcpp `Module` mechanism can not be used to expose inherited fields or methods. An indirect mechanism is used, generating free functions in C++ to expose the inherited members in R.

This mechanism requires data type information in the call to `exposeClass()`. This is provided by naming the corresponding element of the `fields` or `methods` argument with the name of the member. The actual element of the `fields` argument is then the single data type of the field.

For the `methods` argument the argument will generally need to be a named list. The corresponding element of the list is the vector of data types for the return value and for the arguments, if any, to the method. For example, if C++ method `foo()` took a single argument of type `NumericVector` and returned a value of type `long`, the `methods` argument would be `list(foo = c("long", "NumericVector"))`.

See the second example below.

Author(s)

John Chambers

See Also

setRcppClass, which must be called from some R source in the package.

Examples

```
## Not run:
### Given the following C++ class, defined in file PopBD.h,
### the call to exposeClass() shown below will write a file
### src/PopBDModule.cpp containing a corresponding module definition.
###   class PopBD {
###     public:
###       PopBD(void);
###       PopBD(NumericVector initBirth, NumericVector initDeath);
###
###       std::vector<double> birth;
###       std::vector<double> death;
###       std::vector<int> lineage;
###       std::vector<long> size;
###       void evolve(int);
###
###   };
### A file R/PopBDClass.R will be written containing the one line:
###   PopBD <- setRcppClass("PopBD")
###
### The call below exposes the lineage and size fields, read-only,
### and the evolve() method.

exposeClass("PopBD",
      constructors =
        list("", c("NumericVector", "NumericVector")),
      fields = c("lineage", "size"),
      methods = "evolve",
      header = '#include "PopBD.h"',
      readOnly = c("lineage", "size"))

### Example with inheritance:  the class PopCount inherits from
### the previous class, and adds a method table().  It has the same
### constructors as the previous class.
### To expose the table() method, and the inherited evolve() method and size field:

exposeClass("PopCount",
      constructors =
        list("", c("NumericVector", "NumericVector")),
      fields = c(size = "std::vector<long>"),
      methods = list("table", evolve = c("void", "int")),
      header = '#include "PopCount.h"',
      readOnly = "size")

## End(Not run)
```

`formals<--methods` *Set the formal arguments of a C++ function*

Description

Set the formal arguments of a C++ function

Methods

`signature(fun = "C++Function")` Set the formal arguments of a C++ function

`interfacesAttribute` *Rcpp::interfaces Attribute*

Description

The `Rcpp::interfaces` attribute is added to a C++ source file to specify which languages to generate bindings for from exported functions. For example:

```
// [[Rcpp::interfaces(r, cpp)]]
```

Arguments

`...`	Interfaces to generate for exported functions within the source file. Valid values are `r` and `cpp`, and more than one interface can be specified.

Details

The `Rcpp::interfaces` attribute is used to determine which bindings to generate for exported functions. The default behavior if no `Rcpp::interfaces` attribute is specified is to generate only an R interface.

When `cpp` bindings are requested code is generated as follows:

1. Bindings are generated into a header file located in the `inst/include` directory of the package using the naming convention *PackageName_RcppExports.h*
2. If not already present, an additional header file named *PackageName.h* is also generated which in turn includes the Rcpp exports header.

   In the case that you already have a *PackageName.h* header for your package then you can manually add an include of the Rcpp exports header to it to make the exported functions available to users of your package.
3. The generated header file allows calling the exported C++ functions without any linking dependency on the package (this is based on using the `R_RegisterCCallable` and `R_GetCCallable` functions).

4. The exported functions are defined within a C++ namespace that matches the name of the package.

For example, an exported C++ function `foo` could be called from package `MyPackage` as follows:

```
// [[Rcpp::depends(MyPackage)]]

#include <MyPackage.h>

void foo() {
   MyPackage::bar();
}
```

The above example assumes that the `sourceCpp` function will be used to compile the code. If rather than that you are building a package then you don't need to include the `Rcpp::depends` attribute, but instead should add an entry for the referenced package in the `Depends` and `LinkingTo` fields of your package's `DESCRIPTION` file.

Note

If a file by the name of *PackageName.h* that wasn't generated by `compileAttributes` already exists in in the `inst/include` directory then it will not be overwritten (rather, an error will occur).

A static naming scheme for generated header files and namespaces is used to ensure consistent usage semantics for clients of exported `cpp` interfaces. Packages that wish to export more complex interfaces or additional C++ types are therefore typically better off not using this mechanism.

The `Rcpp::interfaces` attribute is specified using a syntax compatible with the new generalized attributes feature of the C++11 standard. Note however that since this feature is not yet broadly supported by compilers it needs to be specified within a comment (see examples below).

See Also

`compileAttributes`, `Rcpp::export`, `Rcpp::depends`

Examples

```
## Not run:

// [[Rcpp::interfaces(r, cpp)]]

## End(Not run)
```

`LdFlags` *(Deprecated) Rcpp Linker Flags*

Description

In previous versions of Rcpp, `LdFlags` and `RcppLdFlags` were used to return the required flags and options for the system linker to link to the Rcpp user library. Since we no longer build or ship a user library, these functions now return an empty string.

Usage

```
LdFlags()
RcppLdFlags()
```

Value

An empty string.

Author(s)

Dirk Eddelbuettel and Romain Francois

References

Dirk Eddelbuettel and Romain Francois (2011). **Rcpp**: Seamless R and C++ Integration. *Journal of Statistical Software*, **40(8)**, 1-18. URL http://www.jstatsoft.org/v40/i08/ and available as `vignette("Rcpp-introduction")`.

`loadModule` *Load an Rcpp Module into a Package*

Description

One or more calls to `loadModule` will be included in the source code for a package to load modules and optionally expose objects from them. The actual extraction of the module takes place at load time.

Usage

```
loadModule(module, what = , loadNow, env =)
```

Arguments

- `module` — The name of the C++ module to load. The code for the module should be in the same package as the R call to `loadModule`.
- `what` — The objects to expose in the package's namespace corresponding to objects in the module. By default, nothing is exposed.

  The special value `TRUE` says to load all the objects in the module that have syntactically standard R names (which all objects in a module will normally have).

  Otherwise, if supplied this should be a character vector, the elements being objects defined in the module. The vector can have a names attribute, in which case the non-empty names will be used to rename the objects; otherwise, the name of the object in the package namespace will be the same as the name in the C++ module.
- `loadNow, env` — A logical flag to say whether the load actions should happen now, and the environment into which the objects should be inserted. When called from the source of a package, both of these arguments should usually be omitted.

  The value of `loadNow` will be set by checking the module's status. At package installation time, the module cannot be started, in which case a load action (see `setLoadAction`) is scheduled to do the actual module load.

  The value of `env` will default to the package's namespace.

Details

If the purpose of loading the module is to define classes based on C++ classes, see `setRcppClass()`, which does the necessary module loading for you.

When the module can be started (at namespace load time), the function `Module()` returns an environment with a description of the module's contents. Function `loadModule()` saves this as a metadata object in the package namespace. Therefore multiple calls to `loadModule()` are an efficient way to extract different objects from the module.

Requesting an object that does not exist in the module produces a warning.

Since assignments from the call cannot take place until namespace loading time, any computations using the objects must also be postponed until this time. Use load actions (`setLoadAction`) and make sure that the load action is specified after the call to `loadModule()`.

Value

If the load takes place, the module environment is returned. Usually however the function is called for its side effects.

Note

This function requires version 2.15.0 of R or later, in order to use load actions, introduced in that version. See the note in the help page for `setRcppClass()` for details.

Author(s)

John Chambers

See Also

setRcppClass() to avoid the explicit call.

loadRcppModules() for a shotgun procedure to load all modules.

Examples

```
## Not run:
loadModule("yada", TRUE) # load all the objects from module "yada"

## End(Not run)
```

---

loadRcppModules — *Loads Rcpp modules on package startup*

Description

Function to simplify loading Rcpp modules contained in a package. This function must be called from the `.onLoad` function of a package. It uses the `RcppModules` field of the package `DESCRIPTION` file to query the names of the modules that the package should export, loads each module, and populate each module into the package NAMESPACE.

Usage

```
loadRcppModules(direct=TRUE)
```

Arguments

direct	if `TRUE` the content of the module is exposed in the namespace. Otherwise, the module is exposed.

See Also

populate

Module — *Retrieves an Rcpp module*

Description

Retrieves an Rcpp module from a dynamic library, usually associated with a package.

Usage

```
Module(module, PACKAGE = , where = , mustStart =  )
```

Arguments

`module`	Name of the module, as declared in the `RCPP_MODULE` macro internally
`PACKAGE`	Passed to `getNativeSymbolInfo`
`where`	When the module is loaded, S4 classes are defined based on the internal classes. This argument is passed to `setClass`
`mustStart`	TODO

Value

An object of class Module collecting functions and classes declared in the module.

`Module-class` *Rcpp modules*

Description

Collection of internal c++ functions and classes exposed to R

Objects from the Class

modules are created by the `link{Module}` function

Methods

$ `signature(x = "Module")`: extract a function or a class from the module.

prompt `signature(object = "Module")`: generates skeleton of a documentation for a Module.

show `signature(object = "Module")`: summary information about the module.

initialize `signature(.Object = "Module")`: ...

See Also

The `Module` function

pluginsAttribute *Rcpp::plugins Attribute*

Description

The `Rcpp::plugins` attribute is added to a C++ source file to specify the inline plugins that should be used in the compilation.

```
// [[Rcpp::plugins(plugin1, plugin2)]]
```

Arguments

`...`	Plugins to add to the compilation.

Details

Plugins must be registered using the `registerPlugin` function.

When included within a `sourceCpp` translation unit, the configuration-related fields of the plugin (e.g. `env` and `LinkingTo`) are utilized, however the code-generation fields (e.g. `includes` and `body`) are not.

Note

Rcpp includes a built-in `cpp11` plugin that adds the flags required to enable `C++11` features in the compiler.

See Also

`registerPlugin`

Examples

```
## Not run:

// [[Rcpp::plugins(cpp11)]]

// [[Rcpp::export]]
int useCpp11() {
    auto x = 10;
    return x;
}

## End(Not run)
```

populate — *Populates a namespace or an environment with the content of a module*

Description

Populates a namespace or an environment with the content of a module

Usage

```
populate(module, env)
```

Arguments

module	Rcpp module
env	environment or namespace

Rcpp.package.skeleton — *Create a skeleton for a new package depending on Rcpp*

Description

Rcpp.package.skeleton automates the creation of a new source package that intends to use features of Rcpp.

It is based on the package.skeleton function which it executes first.

Usage

```
Rcpp.package.skeleton(name = "anRpackage", list = character(),
environment = .GlobalEnv, path = ".", force = FALSE,
code_files = character(), cpp_files = character(),
example_code = TRUE, attributes = TRUE, module = FALSE,
author = "Your Name",
maintainer = if(missing( author)) "Your Name" else author,
email = "your@email.com",
license = "GPL (>= 2)"
)
```

Arguments

name	See package.skeleton
list	See package.skeleton
environment	See package.skeleton
path	See package.skeleton
force	See package.skeleton

`code_files`	See package.skeleton
`cpp_files`	A character vector with the paths to C++ source files to add to the package.
`example_code`	If TRUE, example c++ code using Rcpp is added to the package.
`attributes`	If TRUE, example code makes use of Rcpp attributes.
`module`	If TRUE, an example `Module` is added to the skeleton.
`author`	Author of the package.
`maintainer`	Maintainer of the package.
`email`	Email of the package maintainer.
`license`	License of the package.

Details

In addition to package.skeleton :

The 'DESCRIPTION' file gains an Imports line requesting that the package depends on Rcpp and a LinkingTo line so that the package finds Rcpp header files.

The 'NAMESPACE' gains a `useDynLib` directive as well as an `importFrom(Rcpp, evalCpp` to ensure instantiation of Rcpp.

The 'src' directory is created if it does not exists and a 'Makevars' file is added setting the environment variables 'PKG_LIBS' to accomodate the necessary flags to link with the Rcpp library.

If `cpp_files` are provided then they will be copied to the 'src' directory.

If the `example_code` argument is set to `TRUE`, example files 'rcpp_hello_world.h' and 'rcpp_hello_world.cpp' are also created in the 'src'. An R file 'rcpp_hello_world.R' is expanded in the 'R' directory, the `rcpp_hello_world` function defined in this files makes use of the C++ function 'rcpp_hello_world' defined in the C++ file. These files are given as an example and should eventually by removed from the generated package.

If the `attributes` argument is `TRUE`, then rather than generate the example files as described above, a single 'rcpp_hello_world.cpp' file is created in the 'src' directory and it's attributes are compiled using the `compileAttributes` function, so files 'RcppExports.R' and 'RcppExports.cpp' are generated as well.

If the `module` argument is `TRUE`, a sample Rcpp module will be generated as well.

Value

Nothing, used for its side effects

References

Read the *Writing R Extensions* manual for more details.

Once you have created a *source* package you need to install it: see the *R Installation and Administration* manual, `INSTALL` and `install.packages`.

See Also

package.skeleton

Examples

```
## Not run:
# simple package
Rcpp.package.skeleton( "foobar" )

# package using attributes
Rcpp.package.skeleton( "foobar", attributes = TRUE )

# package with a module
Rcpp.package.skeleton( "testmod", module = TRUE )

# the Rcpp-package vignette
vignette( "Rcpp-package" )

# the Rcpp-modules vignette for information about modules
vignette( "Rcpp-modules" )


## End(Not run)
```

`Rcpp.plugin.maker` *Facilitating making package plugins*

Description

This function helps packages making inline plugins.

Usage

```
Rcpp.plugin.maker(
  include.before = "",
  include.after = "",
  LinkingTo = unique(c(package, "Rcpp")),
  Depends = unique(c(package, "Rcpp")),
  libs = "",
  Makevars = NULL,
  Makevars.win = NULL,
  package = "Rcpp"
)
```

Arguments

`include.before`	Code to be included before the ‘`Rcpp.h`’ file
`include.after`	Code to be included after the ‘`Rcpp.h`’ file
`LinkingTo`	Packages to be added to the ‘`LinkingTo`’ field
`Depends`	Packages to be added to the ‘`Depends`’ field
`libs`	library flags

`Makevars`	content for a '`Makevars`' file, or `NULL`
`Makevars.win`	content for a '`Makevars.win`' file, or `NULL`
`package`	The package this plugin is for.

Value

A function that is suitable as a plugin. See for example the '`RcppArmadillo`' package that uses this to create its inline plugin.

`RcppUnitTests` *Rcpp : unit tests results*

Description

Unit tests results for package Rcpp.

Unit tests are run automatically at build time and reports are included in the '`doc`' directory as html or text.

Details

See Also

Examples

```
# unit tests are in the unitTests directory of the package
list.files( system.file("unitTests", package = "Rcpp" ),
pattern = "^runit", full = TRUE )

# trigger the unit tests preparation, follow printed instructions
# on how to run them
## Not run:
source( system.file("unitTests", "runTests.R", package = "Rcpp" ) )

## End(Not run)
```

registerPlugin *Register an inline plugin*

Description

Register an inline plugin for use with `sourceCpp` or `cppFunction`. Inline plugins are functions that return a list with additional includes, environment variables, and other compilation context.

Usage

```
registerPlugin(name, plugin)
```

Arguments

`name`	Name of the inline plugin
`plugin`	Inline plugin function

Details

Plugins can be added to `sourceCpp` compilations using the `Rcpp::plugins` attribute.

See Also

`Rcpp::plugins`

setRcppClass *Create a Class Extending a C++ Class*

Description

These routines create a class definition in R for an exposed C++ class, setting up and executing a load action to incorporate the C++ pointer information. Neither function should normally need to be called directly; for most applications, a call to `exposeClass()` will create both C++ and R code files to expose the C++ class.

Usage

```
setRcppClass(Class, CppClass = , module = , fields = list(), contains = ,
             methods = , saveAs = Class, where = , ...)
loadRcppClass(Class, CppClass = , module = , fields = character(),
              contains = character(),
              methods = , saveAs = Class, where = , ...)
```

Arguments

- `Class` — The name for the new class.
- `CppClass` — The C++ class defined in the C++ code for the package that this class extends. By default, the same as `Class`.
- `module` — The Rcpp module in which the class is defined. The module does not have to be loaded separately; `setRcppClass()` will arrange to load the module. By default, `"class_"` followed by the C++ class name.

  If `exposeClass()` has been called, the necessary module code will have been written in the `src` directory of the package.
- `fields, contains, methods` — Additional fields, superclasses and method definitions in R that extend the C++ class. These arguments are passed on to `setRefClass()`.
- `saveAs` — Save a generator object for the class in the package's namespace under this name. By default, the generator object has the name of the class. To avoid saving any generator object, supply this argument as `NULL`.

  (This argument is currently needed because the actual class definition must take place at package load time, to include C++ pointer information. Therefore the value returned by `setRcppClass()` when called during package installation is not the generator object returned by `setRefClass()`. We may be able to hack around this problem in the future.)
- `where` — The environment in which to save the class definition. By default, will be the namespace of the package in which the `setRcppClass()` call is included.
- `...` — Arguments, if any, to pass on to `setRefClass()`.

Details

The call to these functions normally appears in the source code for a package; in particular, a call is written in an R source file when `exposeClass()` is called.

R code for this class or (preferably) a subclass can define new fields and methods for the class. Methods for the R class can refer to methods and fields defined in C++ for the C++ class, if those have been exposed.

The fields and methods defined can include overriding C++ fields or methods. Keep in mind, however, that R methods can refer to C++ fields and methods, but not the reverse. If you override a C++ field or method, you essentially need to revise all code that refers to that field or method. Otherwise, the C++ code will continue to use the old C++ definition.

Value

At load time, a generator for the new class is created and stored according to the `saveAs` argument, typically under the name of the class.

The value returned at installation time is a dummy. Future revisions of the function may allow us to return a valid generator at install time. We recommend using the standard style of assigning the value to the name of the class, as one would do with `setRefClass`.

Note

This function and function loadModule() require version 2.15.0 of R or later, in order to use load actions, introduced in that version.

A subtle way this can fail is by somehow loading a legitimate binary version of your package (installed under a valid version of R) into a session with an older R. In this case the load actions created in the binary package will simply not be called. None of the modules will be loaded and none of the classes created.

If your symptom is that classes or other objects from modules don't exist, check the R version.

Author(s)

John Chambers

Examples

```
## Not run:
setRcppClass("World",
    module = "yada",
    fields = list(more = "character"),
    methods = list(
        test = function(what) message("Testing: ", what, "; ", more)),
    saveAs = "genWorld"
         )

## End(Not run)
```

---

sourceCpp *Source C++ Code from a File or String*

Description

sourceCpp parses the specified C++ file or source code and looks for functions marked with the Rcpp::export attribute and RCPP_MODULE declarations. A shared library is then built and its exported functions and Rcpp modules are made available in the specified environment.

Usage

```
sourceCpp(file = "", code = NULL, env = globalenv(),
          embeddedR = TRUE, rebuild = FALSE,
          showOutput = verbose, verbose = getOption("verbose"),
          dryRun = FALSE)
```

Arguments

`file`	A character string giving the path name of a file
`code`	A character string with source code. If supplied, the code is taken from this string instead of a file.
`env`	Environment where the R functions and modules should be made available.
`embeddedR`	`TRUE` to run embedded R code chunks.
`rebuild`	Force a rebuild of the shared library.
`showOutput`	`TRUE` to print `R CMD SHLIB` output to the console.
`verbose`	`TRUE` to print detailed information about generated code to the console.
`dryRun`	`TRUE` to do a dry run (showing commands that would be used rather than actually executing the commands).

Details

If the code parameter is provided then the `file` parameter is ignored.

Functions exported using `sourceCpp` must meet several conditions, including being defined in the global namespace and having return types that are compatible with `Rcpp::wrap` and parameter types that are compatible with `Rcpp::as`. See the `Rcpp::export` documentation for more details.

Content of Rcpp Modules will be automatically loaded into the specified environment using the `Module` and `populate` functions.

If the source file has compilation dependencies on other packages (e.g. **Matrix**, **RcppArmadillo**) then an `Rcpp::depends` attribute should be provided naming these dependencies.

It's possible to embed chunks of R code within a C++ source file by including the R code within a block comment with the prefix of `/*** R`. For example:

```
/*** R

# Call the fibonacci function defined in C++
fibonacci(10)

*/
```

Multiple R code chunks can be included in a C++ file. R code is sourced after the C++ compliation is completed so all functions and modules will be available to the R code.

Value

Returns (invisibly) a list with two elements:

`functions`	Names of exported functions
`modules`	Names of Rcpp modules

Note

The sourceCpp function will not rebuild the shared library if the source file has not changed since the last compilation.

The sourceCpp function is designed for compiling a standalone source file whose only dependencies are R packages. If you are compiling more than one source file or have external dependencies then you should create an R package rather than using sourceCpp. Note that the Rcpp::export attribute can also be used within packages via the compileAttributes function.

If you are sourcing a C++ file from within the src directory of a package then the package's LinkingTo dependencies, inst/include, and src directories are automatically included in the compilation.

If no Rcpp::export attributes or RCPP_MODULE declarations are found within the source file then a warning is printed to the console. You can disable this warning by setting the rcpp.warnNoExports option to FALSE.

See Also

Rcpp::export, Rcpp::depends, cppFunction, evalCpp

Examples

```
## Not run:

sourceCpp("fibonacci.cpp")

sourceCpp(code='
  #include <Rcpp.h>

  // [[Rcpp::export]]
  int fibonacci(const int x) {
    if (x == 0) return(0);
    if (x == 1) return(1);
    return (fibonacci(x - 1)) + fibonacci(x - 2);
  }'
)


## End(Not run)
```

Index

∗Topic **classes**
C++Class-class, 4
C++Constructor-class, 4
C++Field-class, 5
C++Function-class, 6
C++Object-class, 6
C++OverloadedMethods-class, 7
exposeClass, 15
Module-class, 24
setRcppClass, 30
∗Topic **interface**
LdFlags, 21
loadRcppModules, 23
Rcpp-package, 2
Rcpp.plugin.maker, 28
∗Topic **manip**
populate, 26
∗Topic **methods**
.DollarNames-methods, 3
formals<--methods, 19
∗Topic **programming**
demangle, 10
exposeClass, 15
LdFlags, 21
loadModule, 21
Module, 23
Rcpp-package, 2
Rcpp.package.skeleton, 26
RcppUnitTests, 29
.DollarNames,ANY-method (.DollarNames-methods), 3
.DollarNames,C++Object-method (.DollarNames-methods), 3
.DollarNames,Module-method (.DollarNames-methods), 3
.DollarNames-methods, 3
.environment, *4*, *7*
$,C++Class-method (C++Class-class), 4
$,C++Object-method (C++Object-class), 6
$,Module-method (Module-class), 24
$<-,C++Object-method (C++Object-class), 6

areMacrosDefined (evalCpp), 13

C++Class, *5*
C++Class-class, 4
C++Constructor, *4*
C++Constructor-class, 4
C++Field, *4*
C++Field-class, 5
C++Function-class, 6
C++Object-class, 6
C++OverloadedMethods, *4*
C++OverloadedMethods-class, 7
compileAttributes, 7, *14, 15*, *20*, *27*, *34*
cppFunction, 8, *11*, *13*, *30*, *34*

demangle, 10
dependsAttribute, 12

environment, *4*, *7*, *9*
envRefClass, *4*, *7*
evalCpp, *9*, 13, *34*
exportAttribute, 14
exposeClass, 15, *30, 31*

formals<-,C++Function-method (formals<--methods), 19
formals<--methods, 19
function, *6*

getNativeSymbolInfo, *24*

initialize,Module-method (Module-class), 24
inline plugin, *9*, *12*, *30*
inline plugins, *8, 9*, *25*
INSTALL, *27*
install.packages, *27*

interfacesAttribute, 19

LdFlags, 21
loadModule, 21, *32*
loadRcppClass *(setRcppClass)*, 30
loadRcppModules, *23*, 23

Module, *4*, *22*, 23, *24*, *27*, *33*
Module-class, 24

OptionalFunction, *6*

package.skeleton, *26, 27*
pluginsAttribute, 25
populate, *23*, 26, *33*
PossibleMethod, *6*
print.bytes *(demangle)*, 10
prompt,Module-method *(Module-class)*, 24

Rcpp *(Rcpp-package)*, 2
Rcpp-package, 2
Rcpp.package.skeleton, 26
Rcpp.plugin.maker, 28
Rcpp::depends, *20*, *33, 34*
Rcpp::export, *8*, *20*, *32–34*
Rcpp::interfaces, *8*, *15*
Rcpp::plugins, *30*
RcppClass-class *(setRcppClass)*, 30
RcppLdFlags *(LdFlags)*, 21
RcppUnitTests, 29
refClass, *4*, *7*
refObject, *4*, *7*
registerPlugin, *25*, 30

setClass, *24*
setLoadAction, *22*
setRcppClass, *18*, *22, 23*, 30
setRefClass, *31*
show,C++Class-method *(C++Class-class)*, 4
show,C++Function-method *(C++Function-class)*, 6
show,C++Object-method *(C++Object-class)*, 6
show,Module-method *(Module-class)*, 24
sizeof *(demangle)*, 10
sourceCpp, *9*, *12–15*, *25*, *30*, 32
