# Supplementary material for: Comparing Kaolin and Pinolene to Improve Sustainable Grapevine Production during Drought
Source: PLoS One. 2016 Jun 13;11(6):e0156631. doi: 10.1371/journal.pone.0156631 (PMC4905681; doi:10.1371/journal.pone.0156631)
Supplement: S1 File — (GZ) [file pone.0156631.s003.gz › PONE_Brillante_et_al_2016_Code_and_Data/packrat/src/sandwich/sandwich_2.3-3.tar.gz!/sandwich/inst/doc/sandwich-OOP.pdf]

Object-Oriented Computation of Sandwich Estimators

Achim Zeileis
Universität Innsbruck

Abstract

This introduction to the object-orientation features of the R package **sandwich** is a (slightly) modified version of Zeileis (2006), published in the *Journal of Statistical Software*.

Sandwich covariance matrix estimators are a popular tool in applied regression modeling for performing inference that is robust to certain types of model misspecification. Suitable implementations are available in the R system for statistical computing for certain model fitting functions only (in particular `lm()`), but not for other standard regression functions, such as `glm()`, `nls()`, or `survreg()`.

Therefore, conceptual tools and their translation to computational tools in the package **sandwich** are discussed, enabling the computation of sandwich estimators in general parametric models. Object orientation can be achieved by providing a few extractor functions—most importantly for the empirical estimating functions—from which various types of sandwich estimators can be computed.

Keywords: covariance matrix estimators, estimating functions, object orientation, R.

1. Introduction

A popular approach to applied parametric regression modeling is to derive estimates of the unknown parameters via a set of estimating functions (including least squares and maximum likelihood scores). Inference for these models is typically based on a central limit theorem in which the covariance matrix is of a sandwich type: a slice of meat between two slices of bread, pictorially speaking. Employing estimators for the covariance matrix based on this sandwich form can make inference for the parameters more robust against certain model misspecifications (provided the estimating functions still hold and yield consistent estimates). Therefore, sandwich estimators such as heteroskedasticy consistent (HC) estimators for cross-section data and heteroskedasitcity and autocorrelation consistent (HAC) estimators for time-series data are commonly used in applied regression, in particular in linear regression models.

Zeileis (2004) discusses a set of computational tools provided by the **sandwich** package for the R system for statistical computing (R Development Core Team 2008) which allows for computing HC and HAC estimators in linear regression models fitted by `lm()`. Here, we set out where the discussion of Zeileis (2004) ends and generalize the tools from linear to general parametric models fitted by estimating functions. This generalization is achieved by providing an object-oriented implementation for the building blocks of the sandwich that rely only on a small set of extractor functions for fitted model objects. The most important of these is

a method for extracting the empirical estimating functions—based on this a wide variety of meat fillings for sandwiches is provided.

The paper is organized as follows: Section 2 discusses the model frame and reviews some of the underlying theory. Section 3 presents some existing R infrastructure which can be re-used for the computation of sandwich covariance matrices in Section 4. Section 5 gives a brief illustration of the computational tools before Section 6 concludes the paper.

2. Model frame

To fix notations, let us assume we have data in a regression setup, i.e., (y_i, x_i) for $i = 1, \dots, n$, that follow some distribution that is controlled by a k-dimensional parameter vector θ. In many situations, an estimating function $\psi(\cdot)$ is available for this type of models such that $\mathsf{E}[\psi(y, x, \theta)] = 0$. Then, under certain weak regularity conditions (see e.g., White 1994), θ can be estimated using an M-estimator $\hat{\theta}$ implicitely defined as

$$\sum_{i=1}^{n} \psi(y_i, x_i, \hat{\theta}) \quad = \quad 0. \tag{1}$$

This includes cases where the estimating function $\psi(\cdot)$ is the derivative of an objective function $\Psi(\cdot)$:

$$\psi(y, x, \theta) \quad = \quad \frac{\partial \Psi(y, x, \theta)}{\partial \theta}. \tag{2}$$

Examples for estimation techniques included in this framework are maximum likelihood (ML) and ordinary and nonlinear least squares (OLS and NLS) estimation, where the estimator is usually written in terms of the objective function as $\hat{\theta} = \text{argmin}_\theta \sum_i \Psi(y_i, x_i, \theta)$. Other techniques—often expressed in terms of the estimating function rather than the objective function—include quasi ML, robust M-estimation and generalized estimating equations (GEE).

Inference about θ is typically performed relying on a central limit theorem (CLT) of type

$$\sqrt{n}\,(\hat{\theta} - \theta) \quad \xrightarrow{\text{d}} \quad N(0, S(\theta)), \tag{3}$$

where $\xrightarrow{\text{d}}$ denotes convergence in distribution. For the covariance matrix $S(\theta)$, a sandwich formula can be given

$$S(\theta) \quad = \quad B(\theta)\, M(\theta)\, B(\theta) \tag{4}$$

$$B(\theta) \quad = \quad \left(\mathsf{E}[-\psi'(y, x, \theta)]\right)^{-1} \tag{5}$$

$$M(\theta) \quad = \quad \mathsf{VAR}[\psi(y, x, \theta)] \tag{6}$$

see Theorem 6.10 in White (1994), Chapter 5 in Cameron and Trivedi (2005), or Stefanski and Boos (2002) for further details. The "meat" of the sandwich $M(\theta)$ is the variance of the estimating function and the "bread" is the inverse of the expectation of its first derivative ψ' (again with respect to θ). Note that we use the more evocative names S, B and M instead of the more conventional notation $V(\theta) = A(\theta)^{-1} B(\theta) A(\theta)^{-1}$.

In correctly specified models estimated by ML (or OLS and NLS with homoskedastic errors), this sandwich expression for $S(\theta)$ can be simplified because $M(\theta) = B(\theta)^{-1}$, corresponding

to the Fisher information matrix. Hence, the variance $S(\theta)$ in the CLT from Equation 3 is typically estimated by an empirical version of $B(\theta)$. However, more robust covariance matrices can be obtained by employing estimates for $M(\theta)$ that are consistent under weaker assumptions (see e.g., Lumley and Heagerty 1999) and plugging these into the sandwich formula for $S(\theta)$ from Equation 4. Robustness can be achieved with respect to various types of misspecification, e.g., heteroskedasticity—however, consistency of $\hat{\theta}$ has to be assured, which implies that at least the estimating functions have to be correctly specified.

Many of the models of interest to us, provide some more structure: the objective function $\Psi(y, x, \theta)$ depends on x and θ in a special way, namely it does only depend on the univariate linear predictor $\eta = x^\top \theta$. Then, the estimating function is of type

$$\psi(y, x, \theta) \quad = \quad \frac{\partial \Psi}{\partial \eta} \cdot \frac{\partial \eta}{\partial \theta} \quad = \quad \frac{\partial \Psi}{\partial \eta} \cdot x. \tag{7}$$

The partial derivative $r(y, \eta) = \partial \Psi(y, \eta) / \partial \eta$ is in some models also called "working residual" corresponding to the usual residuals in linear regression models. In such linear-predictor-based models, the meat of the sandwich can also be sloppily written as

$$M(\theta) \quad = \quad x \, \mathsf{VAR}[r(y, x^\top \theta)] \, x^\top. \tag{8}$$

Whereas employing this structure for computing HC covariance matrix estimates is well-established practice for linear regression models (see MacKinnon and White 1985; Long and Ervin 2000, among others), it is less commonly applied in other regression models such as GLMs.

3. Existing R infrastructure

To make use of the theory outlined in the previous section, some computational infrastructure is required translating the conceptual to computational tools. R comes with a multitude of model-fitting functions that compute estimates $\hat{\theta}$ and can be seen as special cases of the framework above. They are typically accompanied by extractor and summary methods providing inference based on the CLT from Equation 3. For extracting the estimated parameter vector $\hat{\theta}$ and some estimate of the covariance matrix $S(\theta)$, there are usually a `coef()` and a `vcov()` method, respectively. Based on these estimates, inference can typically be performed by the `summary()` and `anova()` methods. By convention, the `summary()` method performs partial t or z tests and the `anova()` method performs F or χ^2 tests for nested models. The covariance estimate used in these tests (and returned by `vcov()`) usually relies on the assumption of correctly specified models and hence is simply an empirical version of the bread $B(\theta)$ only (divided by n).

For extending these tools to inference based on sandwich covariance matrix estimators, two things are needed: 1. generalizations of `vcov()` that enable computations of sandwich estimates, 2. inference functions corresponding to the `summary()` and `anova()` methods which allow other covariance matrices to be plugged in. As for the latter, the package **lmtest** (Zeileis and Hothorn 2002) provides `coeftest()` and `waldtest()` and **car** (Fox 2002) provides `linear.hypothesis()`—all of these can perform model comparisons in rather general parametric models, employing user-specified covariance matrices. As for the former, only specialized solutions of sandwich covariances matrices are currently available in R packages,

e.g., HAC estimators for linear models in previous versions of **sandwich** and HC estimators for linear models in **car** and **sandwich**. Therefore, we aim at providing a tool kit for plugging together sandwich matrices (including HC and HAC estimators and potentially others) in general parametric models, re-using the functionality that is already provided.

4. Covariance matrix estimators

In the following, the conceptual tools outlined in Section 2 are translated to computational tools preserving their flexibility through the use of the estimating functions framework and re-using the computational infrastructure that is already available in R. Separate methods are suggested for computing estimates for the bread $B(\theta)$ and the meat $M(\theta)$, along with some convenience functions and wrapper interfaces that build sandwiches from bread and meat.

4.1. The bread

Estimating the bread $B(\theta)$ is usually relatively easy and the most popular estimate is the Hessian, i.e., the mean crossproduct of the derivative of the estimating function evaluated at the data and estimated parameters:

$$\hat{B} \quad = \quad \left(\frac{1}{n} \sum_{i=1}^{n} -\psi'(y_i, x_i, \hat{\theta}) \right)^{-1}. \tag{9}$$

If an objective function $\Psi(\cdot)$ is used, this is the crossproduct of its second derivative, hence the name Hessian.

This estimator is what the `vcov()` method is typically based on and therefore it can usually be extracted easily from the fitted model objects, e.g., for "`lm`" and "`glm`" it is essentially the `cov.unscaled` element returned by the `summary()` method. To unify the extraction of a suitable estimate for the bread, **sandwich** provides a new `bread()` generic that should by default return the bread estimate that is also used in `vcov()`. This will usually be the Hessian estimate, but might also be the expected Hessian (Cameron and Trivedi 2005, Equation 5.36) in some models.

The package **sandwich** provides `bread()` methods for "`lm`" (including "`glm`" by inheritance), "`coxph`", "`survreg`" and "`nls`" objects. All of them simply re-use the information provided in the fitted models (or their summaries) and perform hardly any computations, e.g., for "`lm`" objects:

```
bread.lm <- function(obj, ...)
{
  so <- summary(obj)
  so$cov.unscaled * as.vector(sum(so$df[1:2]))
}
```

4.2. The meat

While the bread $B(\theta)$ is typically estimated by the Hessian matrix $\hat{B}$ from Equation 9, various different types of estimators are available for the meat $M(\theta)$, usually offering certain robustness properties. Most of these estimators are based on the empirical values of estimating

functions. Hence, a natural idea for object-oriented implementation of such estimators is the following: provide various functions that compute different estimators for the meat based on an `estfun()` extractor function that extracts the empirical estimating functions from a fitted model object. This is what **sandwich** does: the functions `meat()`, `meatHAC()` and `meatHC()` compute outer product, HAC and HC estimators for $M(\theta)$, respectively, relying on the existence of an `estfun()` method (and potentially a few other methods). Their design is described in the following.

Estimating functions

Whereas (different types of) residuals are typically available as discrepancy measure for a model fit via the `residuals()` method, the empirical values of the estimating functions $\psi(y_i, x_i, \hat{\theta})$ are often not readily implemented in R. Hence, **sandwich** provides a new `estfun()` generic whose methods should return an $n \times k$ matrix with the empirical estimating functions:

$$\begin{pmatrix} \psi(y_1, x_1, \hat{\theta}) \\ \vdots \\ \psi(y_n, x_n, \hat{\theta}) \end{pmatrix}.$$

Suitable methods are provided for "`lm`", "`glm`", "`rlm`", "`nls`", "`survreg`" and "`coxph`" objects. Usually, these can easily re-use existing methods, in particular `residuals()` and `model.matrix()` if the model is of type (7). As a simple example, the most important steps of the "`lm`" method are

```
estfun.lm <- function (obj, ...)
{
  wts <- weights(obj)
  if(is.null(wts)) wts <- 1
  residuals(obj) * wts * model.matrix(obj)
}
```

Outer product estimators

A simple and natural estimator for the meat matrix $M(\theta) = \mathsf{VAR}[\psi(y, x, \theta)]$ is the outer product of the empirical estimating functions:

$$\hat{M} = \frac{1}{n} \sum_{i=1}^{n} \psi(y_i, x_i, \hat{\theta}) \psi(y_i, x_i, \hat{\theta})^\top \qquad (10)$$

This corresponds to the Eicker-Huber-White estimator (Eicker 1963; Huber 1967; White 1980) and is sometimes also called outer product of gradients estimator. In practice, a degrees of freedom adjustment is often used, i.e., the sum is scaled by $n - k$ instead of n, corresponding to the HC1 estimator from MacKinnon and White (1985). In non-linear models this has no theoretical justification, but has been found to have better finite sample performance in some simulation studies.

In **sandwich**, these two estimators are provided by the function `meat()` which only relies on the existence of an `estfun()` method. A simplified version of the R code is

```
meat <- function(obj, adjust = FALSE, ...)
{
  psi <- estfun(obj)
  k <- NCOL(psi)
  n <- NROW(psi)
  rval <- crossprod(as.matrix(psi))/n
  if(adjust) rval <- n/(n - k) * rval
  rval
}
```

HAC estimators

More elaborate methods for deriving consistent covariance matrix estimates in the presence of autocorrelation in time-series data are also available. Such HAC estimators $\hat{M}_{\text{HAC}}$ are based on the weighted empirical autocorrelations of the empirical estimating functions:

$$\hat{M}_{\text{HAC}} \quad = \quad \frac{1}{n} \sum_{i,j=1}^{n} w_{|i-j|} \, \psi(y_i, x_i, \hat{\theta}) \psi(y_j, x_j, \hat{\theta})^\top \tag{11}$$

where different strategies are available for the choice of the weights w_ℓ at lag $\ell = 0, \dots, n-1$ (Andrews 1991; Newey and West 1994; Lumley and Heagerty 1999). Again, an additional finite sample adjustment can be applied by multiplication with $n/(n-k)$.

Once a vector of weights is chosen, the computation of $\hat{M}_{\text{HAC}}$ in R is easy, the most important steps are given by

```
meatHAC <- function(obj, weights, ...)
{
  psi <- estfun(obj)
  n <- NROW(psi)

  rval <- 0.5 * crossprod(psi) * weights[1]
  for(i in 2:length(weights))
    rval <- rval + weights[i] * crossprod(psi[1:(n-i+1),], psi[i:n,])

  (rval + t(rval))/n
}
```

The actual function `meatHAC()` in **sandwich** is much more complex as it also interfaces different weighting and bandwidth selection functions. The details are the same compared to Zeileis (2004) where the selection of weights had been discussed for fitted "`lm`" objects.

HC estimators

In addition to the two HC estimators that can be written as outer product estimators (also called HC0 and HC1), various other HC estimators (usually called HC2–HC4) have been suggested, in particular for the linear regression model (MacKinnon and White 1985; Long and Ervin 2000; Cribari-Neto 2004). In fact, they can be applied to more general models

provided the estimating function depends on the parameters only through a linear predictor as described in Equation 7. Then, the meat matrix $M(\theta)$ is of type (8) which naturally leads to HC estimators of the form $\hat{M}_{\mathrm{HC}} = 1/n\, X^\top \hat{\Omega} X$, where X is the regressor matrix and $\hat{\Omega}$ is a diagonal matrix estimating the variance of $r(y, \eta)$. Various functions $\omega(\cdot)$ have been suggested that derive estimates of the variances from the observed working residuals $(r(y_1, x_1^\top \hat{\theta}), \dots, r(y_n, x_n^\top \hat{\theta}))^\top$—possibly also depending on the hat values and the degrees of freedom. Thus, the HC estimators are of the form

$$\hat{M}_{\mathrm{HC}} = \frac{1}{n} X^\top \left(\begin{array}{ccc} \omega(r(y_1, x_1^\top \theta)) & \cdots & 0 \\ \vdots & \ddots & \vdots \\ 0 & \cdots & \omega(r(y, x^\top \theta)) \end{array} \right) X. \quad (12)$$

To transfer these tools into software in the function `meatHC()`, we need infrastructure for three elements in Equation 12: 1. the model matrix X, 2. the function $\omega(\cdot)$, and 3. the empirical working residuals $r(y_i, x_i^\top \hat{\theta})$. As for 1, the model matrix X can easily be accessed via the `model.matrix()` method. Concerning 2, the specification of $\omega(\cdot)$ is discussed in detail in Zeileis (2004). Hence, we omit the details here and only assume that we have either a vector `omega` of diagonal elements or a function `omega` that computes the diagonal elements from the residuals, diagonal values of the hat matrix (provided by the `hatvalues()` method) and the degrees of freedom $n - k$. For 3, the working residuals, some fitted model classes provide infrastructure in their `residuals()` method. However, there is no unified interface available for this and instead of setting up a new separate generic, it is also possible to recover this information from the estimating function. As $\psi(y_i, x_i, \hat{\theta}) = r(y_i, x_i^\top \hat{\theta}) \cdot x_i$, we can simply divide the empirical estimating function by x_i to obtain the working residual.

Based on these functions, all necessary information can be extracted from fitted model objects and a condensed version of `meatHC()` can then be written as

```
meatHC <- function(obj, omega, ...)
{
  X <- model.matrix(obj)
  res <- rowMeans(estfun(obj)/X, na.rm = TRUE)
  diaghat <- hatvalues(obj)
  df <- NROW(X) - NCOL(X)

  if(is.function(omega)) omega <- omega(res, diaghat, df)
  rval <- sqrt(omega) * X

  crossprod(rval)/NROW(X)
}
```

4.3. The sandwich

Based on the building blocks described in the previous sections, computing a sandwich estimate from a fitted model object is easy: the function `sandwich()` computes an estimate (by default the Eicker-Huber-White outer product estimate) for $1/n\, S(\theta)$ via

```
sandwich <- function(obj, bread. = bread, meat. = meat, ...)
{
  if(is.function(bread.)) bread. <- bread.(obj)
  if(is.function(meat.)) meat. <- meat.(obj, ...)
  1/NROW(estfun(obj)) * (bread. %*% meat. %*% bread.)
}
```

For computing other estimates, the argument `meat.` could also be set to `meatHAC` or `meatHC`.

Therefore, all that an R user/developer would have to do to make a new class of fitted models, "`foo`" say, fit for this framework is: provide an `estfun()` method `estfun.`*foo*`()` and a `bread()` method `bread.`*foo*`()`. See also Figure 1.

Only for HC estimators (other than HC0 and HC1 which are available via `meat()`), it has to be assured in addition that

- the model only depends on a linear predictor (this cannot be easily checked by the software, but has to be done by the user),
- the model matrix X is available via a `model.matrix.`*foo*`()` method,
- a `hatvalues.`*foo*`()` method exists (for HC2–HC4).

For both, HAC and HC estimators, the complexity of the meat functions was reduced for exposition in the paper: choosing the `weights` in `meatHAC` and the diagonal elements `omega` in `meatHC` can be controlled by a number of further arguments. To make these explicit for the user, wrapper functions `vcovHAC()` and `vcovHC()` with suitable default methods are provided in **sandwich** which work as advertised in Zeileis (2004) and are the recommended interfaces for computing HAC and HC estimators, respectively. Furthermore, the convenience interfaces `kernHAC()`, `NeweyWest()` and `weave()` setting the right defaults for (Andrews 1991), Newey and West (1994), and Lumley and Heagerty (1999), respectively, continue to be provided by **sandwich**.

5. Illustrations

This section briefly illustrates how the tools provided by **sandwich** can be applied to various models and re-used in other functions. Predominantly, sandwich estimators are used for inference, such as partial t or z tests of regression coefficients or restriction testing in nested regression models. As pointed out in Section 3, the packages **lmtest** (Zeileis and Hothorn 2002) and **car** (Fox 2002) provide some functions for this type of inference.

The model for which sandwich estimators are employed most often is surely the linear regression model. Part of the reason for this is (together with the ubiquity of linear regression) that in linear regression mean and variance can be specified independently from each other. Thus, the model can be seen as a model for the conditional mean of the response with the variance left unspecified and captured only for inference by a robust sandwich estimator. Zeileis (2004) presents a collection of applications of sandwich estimators to linear regression, both for cross-section and time-series data. These examples are not affected by making **sandwich** object oriented, therefore, we do not present any examples for linear regression models here.

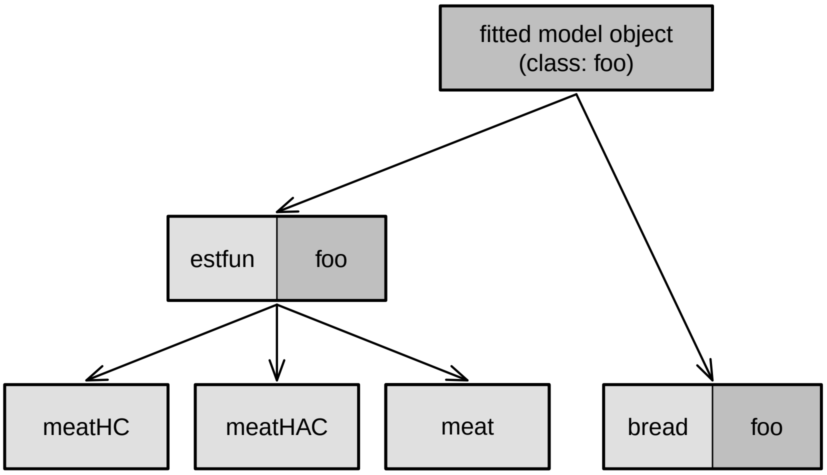

Figure 1: Structure of sandwich estimators

To show that with the new object-oriented tools in **sandwich**, the functions can be applied as easily to other models we consider some models from microeconometrics: count data regression and probit and tobit models. In all examples, we compare the usual summary (coefficients, standard errors and partial z tests) based on `vcov()` with the corresponding summary based on HC standard errors as provided by `sandwich()`. `coeftest()` from **lmtest** is always used for computing the summaries.

5.1. Count data regression

To illustrate the usage of sandwich estimators in count data regressions, we use artifical data simulated from a negative binomial model. The mean of the response `y` depends on a regressor `x` through a log link, the size parameter of the negative binomial distribution is 1, and the regressor is simply drawn from a standard normal distribution. After setting the random seed for reproducibility, we draw 250 observations from this model:

```
R> set.seed(123)
R> x <- rnorm(250)
R> y <- rnbinom(250, mu = exp(1 + x), size = 1)
```

In the following, we will fit various count models to this data employing the overspecification `y ~ x + I(x^2)` and assessing the significance of `I(x^2)`. First, we use `glm()` with `family = poisson` to fit a poisson regression as the simplest model for count data. Of course, this model is not correctly specified as `y` is from a negative binomial distribution. Hence, we are not surprised that the resulting test of `I(x^2)` is spuriously significant:

```
R> fm_pois <- glm(y ~ x + I(x^2), family = poisson)
R> coeftest(fm_pois)

z test of coefficients:
```

```
             Estimate Std. Error z value Pr(>|z|)
(Intercept)  1.063268   0.041357 25.7094  < 2e-16 ***
x            0.996072   0.053534 18.6062  < 2e-16 ***
I(x^2)      -0.049124   0.023146 -2.1223  0.03381 *
---
Signif. codes:  0 '***' 0.001 '**' 0.01 '*' 0.05 '.' 0.1 ' ' 1
```

However, the specification of the conditional mean of `y` is correct in this model which is reflected by the coefficient estimates that are close to their true value. Only the dispersion which is fixed at 1 in the `poisson` family is misspecified. In this situation, the problem can be alleviated by employing sandwich standard errors in the partial z tests, capturing the overdispersion in `y`.

```
R> coeftest(fm_pois, vcov = sandwich)

z test of coefficients:

             Estimate Std. Error z value Pr(>|z|)
(Intercept)  1.063268   0.083776 12.6918   <2e-16 ***
x            0.996072   0.105217  9.4668   <2e-16 ***
I(x^2)      -0.049124   0.036284 -1.3539   0.1758
---
Signif. codes:  0 '***' 0.001 '**' 0.01 '*' 0.05 '.' 0.1 ' ' 1
```

Clearly, sandwich standard errors are not the only way of dealing with this situation. Other obvious candidates would be to use a quasi-poisson or, of course, a negative binomial model (McCullagh and Nelder 1989). The former is available through the `quasipoisson` family for `glm()` that leads to the same coefficient estimates as `poisson` but additionally estimates the dispersion for inference. The associated model summary is very similar to that based on the sandwich standard errors, leading to qualitatively identical results.

```
R> fm_qpois <- glm(y ~ x + I(x^2), family = quasipoisson)
R> coeftest(fm_qpois)

z test of coefficients:

             Estimate Std. Error z value Pr(>|z|)
(Intercept)  1.063268   0.090435 11.7572   <2e-16 ***
x            0.996072   0.117063  8.5088   <2e-16 ***
I(x^2)      -0.049124   0.050613 -0.9706   0.3318
---
Signif. codes:  0 '***' 0.001 '**' 0.01 '*' 0.05 '.' 0.1 ' ' 1
```

Negative binomial models can be fitted by `glm.nb()` from **MASS** (Venables and Ripley 2002).

```
R> fm_nbin <- glm.nb(y ~ x + I(x^2))
R> coeftest(fm_nbin)
```

```
z test of coefficients:

             Estimate Std. Error z value Pr(>|z|)
(Intercept)  1.066057   0.088574 12.0358   <2e-16 ***
x            0.999616   0.094894 10.5340   <2e-16 ***
I(x^2)      -0.052652   0.064883 -0.8115   0.4171
---
Signif. codes:  0 '***' 0.001 '**' 0.01 '*' 0.05 '.' 0.1 ' ' 1
```

Here, the estimated parameters are very similar to those from the poisson regression and the z tests lead to the same conclusions as in the previous two examples.

More details on various techniques for count data regression in R are provided in Zeileis, Kleiber, and Jackman (2008).

5.2. Probit and tobit models

In this section, we consider an example from Greene (2003, Section 22.3.6) that reproduces the analysis of extramarital affairs by Fair (1978). The data, famously known as Fair's affairs, is available in the **AER** package (Kleiber and Zeileis 2008) and provides cross-section information on the number of extramarital affairs of 601 individuals along with several covariates such as `age`, `yearsmarried`, `religiousness`, `occupation` and a self-`rating` of the marriage. Table 22.3 in Greene (2003) provides the parameter estimates and corresponding standard errors of a tobit model (for the number of affairs) and a probit model (for infidelity as a binary variable). In R, these models can be fitted using `tobit()` from **AER** (a convenience interface to `survreg()` from the **survival** package by Therneau and Lumley 2008) and `glm()`, respectively:

```
R> library("AER")
R> data("Affairs", package = "AER")
R> fm_tobit <- tobit(affairs ~ age + yearsmarried + religiousness + occupation + rating, d
R> fm_probit <- glm(I(affairs > 0) ~ age + yearsmarried + religiousness + occupation + rat
+     data = Affairs, family = binomial(link = "probit"))
```

Using `coeftest()`, we compare the usual summary based on the standard errors as computed by `vcov()` (which reproduces the results in Greene 2003) and compare them to the HC standard errors provided by `sandwich()`.

```
R> coeftest(fm_tobit)

z test of coefficients:

               Estimate Std. Error z value  Pr(>|z|)
(Intercept)    8.174197   2.741446  2.9817  0.002866 **
age           -0.179333   0.079093 -2.2674  0.023368 *
yearsmarried   0.554142   0.134518  4.1195 3.798e-05 ***
religiousness -1.686220   0.403752 -4.1764 2.962e-05 ***
```

```
occupation     0.326053   0.254425  1.2815  0.200007    
rating        -2.284973   0.407828 -5.6028 2.109e-08 ***
Log(scale)     2.109859   0.067098 31.4444 < 2.2e-16 ***
---
Signif. codes:  0 '***' 0.001 '**' 0.01 '*' 0.05 '.' 0.1 ' ' 1
```

```
R> coeftest(fm_tobit, vcov = sandwich)

z test of coefficients:

               Estimate Std. Error z value  Pr(>|z|)    
(Intercept)    8.174197   3.077933  2.6557  0.007913 ** 
age           -0.179333   0.088915 -2.0169  0.043706 *  
yearsmarried   0.554142   0.137162  4.0400 5.344e-05 ***
religiousness -1.686220   0.399854 -4.2171 2.475e-05 ***
occupation     0.326053   0.245978  1.3255  0.184993    
rating        -2.284973   0.393479 -5.8071 6.356e-09 ***
Log(scale)     2.109859   0.054837 38.4754 < 2.2e-16 ***
---
Signif. codes:  0 '***' 0.001 '**' 0.01 '*' 0.05 '.' 0.1 ' ' 1
```

For the tobit model `fm_tobit`, the HC standard errors are only slightly different and yield qualitatively identical results. The picture is similar for the probit model `fm_probit` which leads to the same interpretations, both for the standard and the HC estimate.

```
R> coeftest(fm_probit)

z test of coefficients:

               Estimate Std. Error z value  Pr(>|z|)    
(Intercept)    0.976668   0.365375  2.6731 0.0075163 ** 
age           -0.022024   0.010319 -2.1343 0.0328214 *  
yearsmarried   0.059901   0.017121  3.4986 0.0004677 ***
religiousness -0.183646   0.051715 -3.5511 0.0003836 ***
occupation     0.037513   0.032845  1.1421 0.2533995    
rating        -0.272983   0.052574 -5.1923 2.077e-07 ***
---
Signif. codes:  0 '***' 0.001 '**' 0.01 '*' 0.05 '.' 0.1 ' ' 1
```

```
R> coeftest(fm_probit, vcov = sandwich)

z test of coefficients:

               Estimate Std. Error z value  Pr(>|z|)    
(Intercept)    0.976668   0.393033  2.4850 0.0129569 *  
age           -0.022024   0.011274 -1.9534 0.0507678 .  
```

```
yearsmarried   0.059901   0.017557  3.4119 0.0006452 ***
religiousness -0.183646   0.053047 -3.4620 0.0005363 ***
occupation     0.037513   0.032922  1.1395 0.2545089
rating        -0.272983   0.053327 -5.1190 3.071e-07 ***
---
Signif. codes:  0 '***' 0.001 '**' 0.01 '*' 0.05 '.' 0.1 ' ' 1
```

See Greene (2003) for a more detailed discussion of these and other regression models for Fair's affairs data.

6. Discussion

Object-oriented computational infrastructure in the R package **sandwich** for estimating sandwich covariance matrices in a wide class of parametric models is suggested. Re-using existing building blocks, all an R developer has to implement for adapting a new fitted model class to the sandwich estimators are methods for extracting a bread estimator and the empirical estimating functions (and possibly model matrix and hat values).

Although the most important area of application of sandwich covariance matrices is inference, particularly restriction testing, the package **sandwich** does not contain any inference functions but rather aims at providing modular building blocks that can be re-used in or supplied to other computational tools. In this paper, we show how the **sandwich** functions can be plugged into some functions made available by other packages that implement tools for Wald tests. However, it should be pointed out that this is not the only strategy for employing sandwich covariances for restriction testing; recent research provides us with at least two other promising strategies: For cross-section data, Godfrey (2006) shows that the finite sample performance of quasi t or z tests can be improved by computing HC estimators based on the residuals of the restricted model and assessing their significance based on their bootstrap distribution. For time-series data, Kiefer and Vogelsang (2002) consider t-type statistics based on HAC estimators where the bandwidth is equal to the sample size, leading to a non-normal asymptotic distribution of the t statistic. For both strategies, some tools from **sandwich** could be easily re-used but further infrastructure, in particular for the inference, is required. As this is beyond the scope of the **sandwich** package, we leave this for future developments in packages focused on inference in regression models.

As the new tools in **sandwich** provide "robust" covariances for a wide class of parametric models, it is worth pointing out that this should *not* encourage the user to employ them automatically for every model in every analysis. First, the use of sandwich estimators when the model is correctly specified leads to a loss of power. Second, if the model is not correctly specified, the sandwich estimators are only useful if the parameters estimates are still consistent, i.e., if the misspecification does not result in bias. Whereas it is well understood what types of misspecification can be dealt with in linear regression models, the situation is less obvious for general regression models. Some further expository discussion of this issue for ML and quasi ML estimators can be found in Freedman (2006) and Koenker (2006).

Acknowledgments

The extensions of **sandwich**, in particular to microeconometric models, was motivated by the joint work with Christian Kleiber on Kleiber and Zeileis (2008). We would also like to thank Henric Nilsson for helpful feedback and discussions that helped to improve and generalize the functions in the package. Furthermore, we gratefully acknowledge the valuable comments of the associate editor and two referees which led to an improvement of the paper.

References

Andrews DWK (1991). "Heteroskedasticity and Autocorrelation Consistent Covariance Matrix Estimation." *Econometrica*, **59**, 817–858.

Cameron AC, Trivedi PK (2005). *Microeconometrics: Methods and Applications.* Cambridge University Press, Cambridge.

Cribari-Neto F (2004). "Asymptotic Inference Under Heteroskedasticity of Unknown Form." *Computational Statistics & Data Analysis*, **45**, 215–233.

Eicker F (1963). "Asymptotic Normality and Consistency of the Least Squares Estimator for Families of Linear Regressions." *Annals of Mathematical Statistics*, **34**, 447–456.

Fair RC (1978). "A Theory of Extramarital Affairs." *Journal of Political Economy*, **86**, 45–61.

Fox J (2002). *An R and S-PLUS Companion to Applied Regression.* Sage Publications, Thousand Oaks, CA.

Freedman DA (2006). "On the So-Called 'Huber Sandwich Estimator' and 'Robust Standard Errors'." *The American Statistician*, **60**(4), 299–302.

Godfrey LG (2006). "Tests for Regression Models with Heteroskedasticity of Unknown Form." *Computational Statistics & Data Analysis*, **50**, 2715–2733.

Greene WH (2003). *Econometric Analysis.* 5th edition. Prentice Hall, Upper Saddle River, NJ.

Huber PJ (1967). "The Behavior of Maximum Likelihood Estimation Under Nonstandard Conditions." In LM LeCam, J Neyman (eds.), *Proceedings of the Fifth Berkeley Symposium on Mathematical Statistics and Probability.* University of California Press, Berkeley, CA.

Kiefer NM, Vogelsang TJ (2002). "Heteroskedasticity-Autocorrelation Robust Testing Using Bandwidth Equal to Sample Size." *Econometric Theory*, **18**, 1350–1366.

Kleiber C, Zeileis A (2008). *Applied Econometrics with R.* Springer-Verlag, New York. ISBN 978-0-387-77316-2, URL http://CRAN.R-project.org/package=AER.

Koenker R (2006). "Maximum Likelihood Asymptotics Under Non-standard Conditions: A Heuristic Introduction to Sandwiches." Lecture Notes. URL http://www.econ.uiuc.edu/~roger/courses/476/lectures/L10.pdf.

Long JS, Ervin LH (2000). "Using Heteroscedasticity Consistent Standard Errors in the Linear Regression Model." *The American Statistician*, **54**, 217–224.

Lumley T, Heagerty P (1999). "Weighted Empirical Adaptive Variance Estimators for Correlated Data Regression." *Journal of the Royal Statistical Society B*, **61**, 459–477.

MacKinnon JG, White H (1985). "Some Heteroskedasticity-Consistent Covariance Matrix Estimators with Improved Finite Sample Properties." *Journal of Econometrics*, **29**, 305–325.

McCullagh P, Nelder JA (1989). *Generalized Linear Models*. 2nd edition. Chapman & Hall, London.

Newey WK, West KD (1994). "Automatic Lag Selection in Covariance Matrix Estimation." *Review of Economic Studies*, **61**, 631–653.

R Development Core Team (2008). *R: A Language and Environment for Statistical Computing*. R Foundation for Statistical Computing, Vienna, Austria. ISBN 3-900051-07-0, URL http://www.R-project.org/.

Stefanski LA, Boos DD (2002). "The Calculus of M-Estimation." *The American Statistician*, **56**, 29–38.

Therneau TM, Lumley T (2008). ***survival**: Survival Analysis*. R package version 2.34-1. S original by Terry M. Therneau, ported to R by Thomas Lumley, URL http://CRAN.R-project.org/package=survival.

Venables WN, Ripley BD (2002). *Modern Applied Statistics with S*. 4th edition. Springer-Verlag, New York.

White H (1980). "A Heteroskedasticity-Consistent Covariance Matrix and a Direct Test for Heteroskedasticity." *Econometrica*, **48**, 817–838.

White H (1994). *Estimation, Inference and Specification Analysis*. Cambridge University Press, Cambridge.

Zeileis A (2004). "Econometric Computing with HC and HAC Covariance Matrix Estimators." *Journal of Statistical Software*, **11**(10), 1–17. URL http://www.jstatsoft.org/v11/i10/.

Zeileis A (2006). "Object-oriented Computation of Sandwich Estimators." *Journal of Statistical Software*, **16**(9), 1–16. URL http://www.jstatsoft.org/v16/i09/.

Zeileis A, Hothorn T (2002). "Diagnostic Checking in Regression Relationships." *R News*, **2**(3), 7–10. URL http://CRAN.R-project.org/doc/Rnews/.

Zeileis A, Kleiber C, Jackman S (2008). "Regression Models for Count Data in R." *Journal of Statistical Software*, **27**(8), 1–25. URL http://www.jstatsoft.org/v27/i08/.

Affiliation:

Achim Zeileis
Department of Statistics

Faculty of Economics and Statistics
Universität Innsbruck
Universitätsstr. 15
6020 Innsbruck, Austria
E-mail: Achim.Zeileis@R-project.org
URL: http://eeecon.uibk.ac.at/~zeileis/
