# Supplementary material for: Comparing Kaolin and Pinolene to Improve Sustainable Grapevine Production during Drought
Source: PLoS One. 2016 Jun 13;11(6):e0156631. doi: 10.1371/journal.pone.0156631 (PMC4905681; doi:10.1371/journal.pone.0156631)
Supplement: S1 File — (GZ) [file pone.0156631.s003.gz › PONE_Brillante_et_al_2016_Code_and_Data/packrat/src/sandwich/sandwich_2.3-3.tar.gz!/sandwich/inst/doc/sandwich.pdf]

Econometric Computing with HC and HAC Covariance Matrix Estimators

Achim Zeileis
Universität Innsbruck

Abstract

This introduction to the R package **sandwich** is a (slightly) modified version of Zeileis (2004), published in the *Journal of Statistical Software*. A follow-up paper on object object-oriented computation of sandwich estimators is available in (Zeileis 2006b).

Data described by econometric models typically contains autocorrelation and/or heteroskedasticity of unknown form and for inference in such models it is essential to use covariance matrix estimators that can consistently estimate the covariance of the model parameters. Hence, suitable heteroskedasticity-consistent (HC) and heteroskedasticity and autocorrelation consistent (HAC) estimators have been receiving attention in the econometric literature over the last 20 years. To apply these estimators in practice, an implementation is needed that preferably translates the conceptual properties of the underlying theoretical frameworks into computational tools. In this paper, such an implementation in the package **sandwich** in the R system for statistical computing is described and it is shown how the suggested functions provide reusable components that build on readily existing functionality and how they can be integrated easily into new inferential procedures or applications. The toolbox contained in **sandwich** is extremely flexible and comprehensive, including specific functions for the most important HC and HAC estimators from the econometric literature. Several real-world data sets are used to illustrate how the functionality can be integrated into applications.

Keywords: covariance matrix estimators, heteroskedasticity, autocorrelation, estimating functions, econometric computing, R.

1. Introduction

This paper combines two topics that play an important role in applied econometrics: computational tools and robust covariance estimation.

Without the aid of statistical and econometric software modern data analysis would not be possible: hence, both practitioners and (applied) researchers rely on computational tools that should preferably implement state-of-the-art methodology and be numerically reliable, easy to use, flexible and extensible.

In many situations, economic data arises from time-series or cross-sectional studies which typically exhibit some form of autocorrelation and/or heteroskedasticity. If the covariance structure were known, it could be taken into account in a (parametric) model, but more often than not the form of autocorrelation and heteroskedasticity is unknown. In such cases, model parameters can typically still be estimated consistently using the usual estimating

functions, but for valid inference in such models a consistent covariance matrix estimate is essential. Over the last 20 years several procedures for heteroskedasticity consistent (HC) and for heteroskedasticity and autocorrelation consistent (HAC) covariance estimation have been suggested in the econometrics literature (White 1980; MacKinnon and White 1985; Newey and West 1987, 1994; Andrews 1991, among others) and are now routinely used in econometric analyses.

Many statistical and econometric software packages implement various HC and HAC estimators for certain inference procedures, so why is there a need for a paper about econometric computing with HC and HAC estimators? Typically, only certain special cases of such estimators—and not the general framework they are taken from—are implemented in statistical and econometric software packages and sometimes they are only available as options to certain inference functions. It is desirable to improve on this for two reasons: First, the literature suggested conceptual frameworks for HC and HAC estimation and it would only be natural to translate these conceptual properties into computational tools that reflect the flexibility of the general framework. Second, it is important, particularly for applied research, to have covariance matrices not only as options to certain tests but as stand-alone functions which can be used as modular building blocks and plugged into various inference procedures. This is becoming more and more relevant, because today, as Cribari-Neto and Zarkos (2003) point out, applied researchers typically cannot wait until a certain procedure becomes available in the software package of their choice but are often forced to program new techniques themselves. Thus, just as suitable covariance estimators are routinely plugged into formulas in theoretical work, programmers should be enabled to plug in implementations of such estimators in computational work. Hence, the aim of this paper is to present an econometric computing approach to HC and HAC estimation that provides reusable components that can be used as modular building blocks in implementing new inferential techniques and in applications.

All functions described are available in the package **sandwich** implemented in the R system for statistical computing (R Development Core Team 2008) which is currently not the most popular environment for econometric computing but which is finding increasing attention among econometricians (Cribari-Neto and Zarkos 1999; Racine and Hyndman 2002). Both R itself and the **sandwich** package (as well as all other packages used in this paper) are available at no cost under the terms of the general public licence (GPL) from the comprehensive R archive network (CRAN, `http://CRAN.R-project.org/`). R has no built-in support for HC and HAC estimation and at the time we started writing **sandwich** there was only one package that implements HC (but not HAC) estimators (the **car** package Fox 2002) but which does not allow for as much flexibility as the tools presented here. **sandwich** provides the functions `vcovHC` and `vcovHAC` implementing general classes of HC and HAC estimators. The names of the functions are chosen to correspond to `vcov`, R's generic function for extracting covariance matrices from fitted model objects.

Below, we focus on the general linear regression model estimated by ordinary least squares (OLS), which is typically fitted in R using the function `lm` from which the standard covariance matrix (assuming spherical errors) can be extracted by `vcov`. Using the tools from **sandwich**, HC and HAC covariances matrices can now be extracted from the same fitted models using `vcovHC` and `vcovHAC`. Due to the object orientation of R, these functions are not only limited to the linear regression model but can be easily extended to other models. The HAC estimators are already available for generalized linear models (fitted by `glm`) and robust regression (fitted

by `rlm` in package **MASS**). Another important feature of R that is used repeatedly below is that functions are first-level objects—i.e., functions can take functions as arguments and return functions—which is particularly useful for defining certain procedures for data-driven computations such as the definition of the structure of covariance matrices in HC estimation and weighting schemes for HAC estimation.

The remainder of this paper is structured as follows: To fix notations, Section 2 describes the linear regression model used and motivates the following sections. Section 3 gives brief literature reviews and describes the conceptual frameworks for HC and HAC estimation respectively and then shows how the conceptual properties are turned into computational tools in **sandwich**. Section 4 provides some illustrations and applications of these tools before a summary is given in Section 5. More details about the R code used are provided in an appendix.

2. The linear regression model

To fix notations, we consider the linear regression model

$$y_i \quad = \quad x_i^\top \beta \; + \; u_i \qquad (i = 1, \dots, n), \tag{1}$$

with dependent variable y_i, k-dimensional regressor x_i with coefficient vector β and error term u_i. In the usual matrix notation comprising all n observations this can be formulated as $y = X\beta + u$.

In the general linear model, it is typically assumed that the errors have zero mean and variance $\mathsf{VAR}[u] = \Omega$. Under suitable regularity conditions (see e.g., Greene 1993; White 2000), the coefficients β can be consistently estimated by OLS giving the well-known OLS estimator $\hat\beta$ with corresponding OLS residuals $\hat u_i$:

$$\hat\beta \quad = \quad \left(X^\top X\right)^{-1} X^\top y \tag{2}$$

$$\hat u \quad = \quad (I_n - H)\, y \; = \; (I_n - X \left(X^\top X\right)^{-1} X^\top)\, y \tag{3}$$

where I_n is the n-dimensional identity matrix and H is usually called hat matrix. The estimates $\hat\beta$ are unbiased and asymptotically normal (White 2000). Their covariance matrix Ψ is usually denoted in one of the two following ways:

$$\Psi \; = \; \mathsf{VAR}[\hat\beta] \quad = \quad \left(X^\top X\right)^{-1} X^\top \Omega X \left(X^\top X\right)^{-1} \tag{4}$$

$$= \quad \left(\frac{1}{n} X^\top X\right)^{-1} \frac{1}{n} \Phi \left(\frac{1}{n} X^\top X\right)^{-1} \tag{5}$$

where $\Phi = n^{-1} X^\top \Omega X$ is essentially the covariance matrix of the scores or estimating functions $V_i(\beta) = x_i(y_i - x_i^\top \beta)$. The estimating functions evaluated at the parameter estimates $\hat V_i = V_i(\hat\beta)$ have then sum zero.

For inference in the linear regression model, it is essential to have a consistent estimator for Ψ. What kind of estimator should be used for Ψ depends on the assumptions about Ω: In the classical linear model independent and homoskedastic errors with variance σ^2 are assumed

yielding $\Omega = \sigma^2 I_n$ and $\Psi = \sigma^2(X^\top X)^{-1}$ which can be consistently estimated by plugging in the usual OLS estimator $\hat{\sigma}^2 = (n-k)^{-1} \sum_{i=1}^n \hat{u}_i^2$. But if the independence and/or homoskedasticity assumption is violated, inference based on this estimator $\hat{\Psi}_{\mathrm{const}} = \hat{\sigma}(X^\top X)^{-1}$ will be biased. HC and HAC estimators tackle this problem by plugging an estimate $\hat{\Omega}$ or $\hat{\Phi}$ into (4) or (5) respectively which are consistent in the presence of heteroskedasticity and autocorrelation respectively. Such estimators and their implementation are described in the following section.

3. Estimating the covariance matrix Ψ

3.1. Dealing with heteroskedasticity

If it is assumed that the errors u_i are independent but potentially heteroskedastic—a situation which typically arises with cross-sectional data—their covariance matrix Ω is diagonal but has nonconstant diagonal elements. Therefore, various HC estimators $\hat{\Psi}_{\mathrm{HC}}$ have been suggested which are constructed by plugging an estimate of type $\hat{\Omega} = \mathrm{diag}(\omega_1, \dots, \omega_n)$ into Equation (4). These estimators differ in their choice of the ω_i, an overview of the most important cases is given in the following:

$$\begin{aligned}
\text{const}: \quad \omega_i &= \hat{\sigma}^2 \\
\text{HC0}: \quad \omega_i &= \hat{u}_i^2 \\
\text{HC1}: \quad \omega_i &= \frac{n}{n-k}\hat{u}_i^2 \\
\text{HC2}: \quad \omega_i &= \frac{\hat{u}_i^2}{1-h_i} \\
\text{HC3}: \quad \omega_i &= \frac{\hat{u}_i^2}{(1-h_i)^2} \\
\text{HC4}: \quad \omega_i &= \frac{\hat{u}_i^2}{(1-h_i)^{\delta_i}}
\end{aligned}$$

where $h_i = H_{ii}$ are the diagonal elements of the hat matrix, $\bar{h}$ is their mean and $\delta_i = \min\{4, h_i/\bar{h}\}$.

The first equation above yields the standard estimator $\hat{\Psi}_{\mathrm{const}}$ for homoskedastic errors. All others produce different kinds of HC estimators. The estimator HC0 was suggested in the econometrics literature by White (1980) and is justified by asymptotic arguments. The estimators HC1, HC2 and HC3 were suggested by MacKinnon and White (1985) to improve the performance in small samples. A more extensive study of small sample behaviour was carried out by Long and Ervin (2000) which arrive at the conclusion that HC3 provides the best performance in small samples as it gives less weight to influential observations. Recently, Cribari-Neto (2004) suggested the estimator HC4 to further improve small sample performance, especially in the presence of influential observations.

All of these HC estimators $\hat{\Psi}_{\mathrm{HC}}$ have in common that they are determined by $\omega = (\omega_1, \dots, \omega_n)^\top$ which in turn can be computed based on the residuals $\hat{u}$, the diagonal of the hat matrix h and the degrees of freedom $n-k$. To translate these conceptual properties of this class of HC estimators into a computational tool, a function is required which takes a fitted regres-

sion model and the diagonal elements ω as inputs and returns the corresponding $\hat{\Psi}_{\mathrm{HC}}$. In **sandwich**, this is implemented in the function `vcovHC` which takes the following arguments:

```
vcovHC(lmobj, omega = NULL, type = "HC3", ...)
```

The first argument `lmobj` is an object as returned by `lm`, R's standard function for fitting linear regression models. The argument `omega` can either be the vector ω or a function for data-driven computation of ω based on the residuals $\hat{u}$, the diagonal of the hat matrix h and the residual degrees of freedom $n-k$. Thus, it has to be of the form `omega(residuals, diaghat, df)`: e.g., for computing HC3 `omega` is set to `function(residuals, diaghat, df) residuals^2/(1 - diaghat)^2`.

As a convenience option, a `type` argument can be set to `"const"`, `"HC0"` (or equivalently `"HC"`), `"HC1"`, `"HC2"`, `"HC3"` (the default) or `"HC4"` and then `vcovHC` uses the corresponding `omega` function. As soon as `omega` is specified by the user, `type` is ignored.

In summary, by specfying ω—either as a vector or as a function—`vcovHC` can compute arbitrary HC covariance matrix estimates from the class of estimators outlined above. In Section 4, it will be illustrated how this function can be used as a building block when doing inference in linear regression models.

3.2. Dealing with autocorrelation

If the error terms u_i are not independent, Ω is not diagonal and without further specification of a parametic model for the type of dependence it is typically burdensome to estimate Ω directly. However, if the form of heteroskedasticity and autocorrelation is unknown, a solution to this problem is to estimate Φ instead which is essentially the covariance matrix of the estimating functions[1]. This is what HAC estimators do: $\hat{\Psi}_{\mathrm{HAC}}$ is computed by plugging an estimate $\hat{\Phi}$ into Equation (5) with

$$\hat{\Phi} \quad = \quad \frac{1}{n} \sum_{i,j=1}^{n} w_{|i-j|} \, \hat{V}_i \hat{V}_j^\top \tag{6}$$

where $w = (w_0, \dots, w_{n-1})^\top$ is a vector of weights. An additional finite sample adjustment can be applied by multiplication with $n/(n-k)$. For many data structures, it is a reasonable assumption that the autocorrelations should decrease with increasing lag $\ell = |i-j|$—otherwise β can typically not be estimated consistently by OLS—so that it is rather intuitive that the weights w_ℓ should also decrease. Starting from White and Domowitz (1984) and Newey and West (1987), different choices for the vector of weights w have been suggested in the econometrics literature which have been placed by Andrews (1991) in a more general framework of choosing the weights by kernel functions with automatic bandwidth selection. Andrews and Monahan (1992) show that the bias of the estimators can be reduced by prewhitening the estimating functions $\hat{V}_i$ using a vector autoregression (VAR) of order p and applying the estimator in Equation (6) to the VAR(p) residuals subsequently. Lumley and Heagerty (1999) suggest an adaptive weighting scheme where the weights are chosen based on the estimated autocorrelations of the residuals $\hat{u}$.

[1]Due to the use of estimating functions, this approach is not only feasible in linear models estimated by OLS, but also in nonlinear models using other estimating functions such as maximum likelihood (ML), generalized methods of moments (GMM) or Quasi-ML.

All the estimators mentioned above are of the form (6), i.e., a weighted sum of lagged products of the estimating functions corresponding to a fitted regression model. Therefore, a natural implementation for this class of HAC estimators is the following:

```
vcovHAC(lmobj, weights,
  prewhite = FALSE, adjust = TRUE, sandwich = TRUE,
  order.by, ar.method, data)
```

The most important arguments are again the fitted linear model[2] `lmobj`—from which the estimating functions $\hat{V}_i$ can easily be extracted using the generic function `estfun(lmobj)`—and the argument `weights` which specifys w. The latter can be either the vector w directly or a function to compute it from `lmobj`.[3] The argument `prewhite` specifies wether prewhitening should be used or not[4] and `adjust` determines wether a finite sample correction by multiplication with $n/(n-k)$ should be made or not. By setting `sandwich` it can be controlled wether the full sandwich estimator $\hat{\Psi}_{\text{HAC}}$ or only the "meat" $\hat{\Phi}/n$ of the sandwich should be returned. The remaining arguments are a bit more technical: `order.by` specifies by which variable the data should be ordered (the default is that they are already ordered, as is natural with time series data), which `ar.method` should be used for fitting the VAR(p) model (the default is OLS) and `data` provides a data frame from which `order.by` can be taken (the default is the environment from which `vcovHAC` is called).[5]

As already pointed out above, all that is required for specifying an estimator $\hat{\Psi}_{\text{HAC}}$ is the appropriate vector of weights (or a function for data-driven computation of the weights). For the most important estimators from the literature mentioned above there are functions for computing the corresponding weights readily available in **sandwich**. They are all of the form `weights(lmobj, order.by, prewhite, ar.method, data)`, i.e., functions that compute the weights depending on the fitted model object `lmobj` and the arguments `order.by`, `prewhite`, `data` which are only needed for ordering and prewhitening. The function `weightsAndrews` implements the class of weights of Andrews (1991) and `weightsLumley` implements the class of weights of Lumley and Heagerty (1999). Both functions have convenience interfaces: `kernHAC` calls `vcovHAC` with `weightsAndrews` (and different defaults for some parameters) and `weave` calls `vcovHAC` with `weightsLumley`. Finally, a third convenience interface to `vcovHAC` is available for computing the estimator(s) of Newey and West (1987, 1994).

- Newey and West (1987) suggested to use linearly decaying weights

$$w_\ell = 1 - \frac{\ell}{L+1} \qquad (7)$$

  where L is the maximum lag, all other weights are zero. This is implemented in the function `NeweyWest(lmobj, lag = NULL, ...)` where `lag` specifies L and `...` are

[2] Note, that not only HAC estimators for fitted *linear* models can be computed with `vcovHAC`. See Zeileis (2006b) for details.

[3] If `weights` is a vector with less than n elements, the remaining weights are assumed to be zero.

[4] The order p is set to `as.integer(prewhite)`, hence both `prewhite = 1` and `prewhite = TRUE` lead to a VAR(1) model, but also `prewhite = 2` is possible.

[5] More detailed technical documentation of these and other arguments of the functions described are available in the reference manual included in **sandwich**.

(here, and in the following) further arguments passed to other functions, detailed information is always available in the reference manual. If `lag` is set to `NULL` (the default) the non-parametric bandwidth selection procedure of Newey and West (1994) is used. This is also available in a stand-alone function `bwNeweyWest`, see also below.

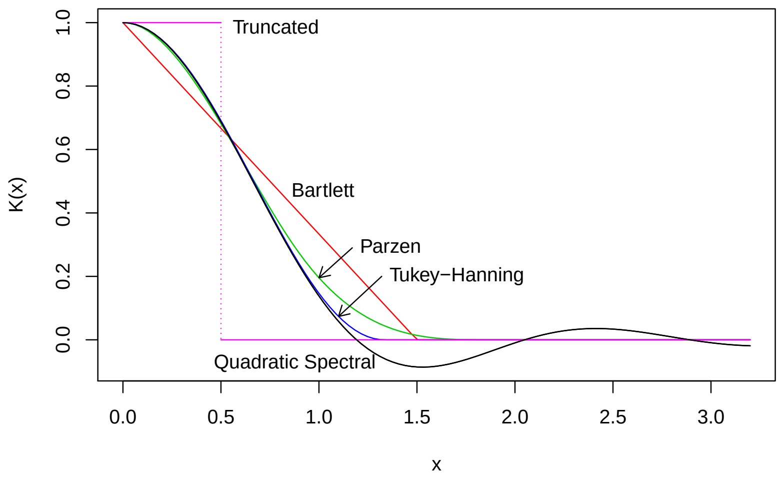

Figure 1: Kernel functions for kernel-based HAC estimation.

- Andrews (1991) placed this and other estimators in a more general class of kernel-based HAC estimators with weights of the form $w_\ell = K(\ell/B)$ where $K(\cdot)$ is a kernel function and B the bandwidth parameter used. The kernel functions considered are the truncated, Bartlett, Parzen, Tukey-Hanning and quadratic spectral kernel which are depicted in Figure 1. The Bartlett kernel leads to the weights used by Newey and West (1987) in Equation (7) when the bandwidth B is set to $L+1$. The kernel recommended by Andrews (1991) and probably most used in the literature is the quadratic spectral kernel which leads to the following weights:

$$w_\ell \quad = \quad \frac{3}{z^2}\left(\frac{\sin(z)}{z} - \cos(z)\right), \qquad (8)$$

where $z = 6\pi/5 \cdot \ell/B$. The definitions for the remaining kernels can be found in Andrews (1991). All kernel weights mentioned above are available in `weightsAndrews(lmobj, kernel, bw, ...)` where `kernel` specifies one of the kernels via a character string (`"Truncated"`, `"Bartlett"`, `"Parzen"`, `"Tukey-Hanning"` or `"Quadratic Spectral"`) and `bw` the bandwidth either as a scalar or as a function. The automatic bandwidth selection described in Andrews (1991) via AR(1) or ARMA(1,1) approximations is implemented in a function `bwAndrews` which is set as the default in `weightsAndrews`. For the Bartlett, Parzen and quadratic spectral kernels, Newey and West (1994) suggested a different nonparametric bandwidth selection procedure, which is implemented in `bwNeweyWest` and which can also be passed to `weightsAndrews`. As the flexibility of

this conceptual framework of estimators leads to a lot of knobs and switches in the computational tools, a convenience function `kernHAC` for kernel-based HAC estimation has been added to **sandwich** that calls `vcovHAC` based on `weightsAndrews` and `bwAndrews` with defaults as motivated by Andrews (1991) and Andrews and Monahan (1992): by default, it computes a quadratic spectral kernel HAC estimator with VAR(1) prewhitening and automatic bandwidth selection based on an AR(1) approximation. But of course, all the options described above can also be changed by the user when calling `kernHAC`.

- Lumley and Heagerty (1999) suggested a different approach for specifying the weights in (6) based on some estimate $\hat{\varrho}_\ell$ of the autocorrelation of the residuals $\hat{u}_i$ at lag $0 = 1, \dots, n-1$. They suggest either to use truncated weights $w_\ell = I\{n\,\hat{\varrho}_\ell^2 > C\}$ (where $I(\cdot)$ is the indicator function) or smoothed weights $w_\ell = \min\{1, C\,n\,\hat{\varrho}_\ell^2\}$, where for both a suitable constant C has to be specified. Lumley and Heagerty (1999) suggest using a default of $C = 4$ and $C = 1$ for the truncated and smoothed weights respectively. Note, that the truncated weights are equivalent to the truncated kernel from the framework of Andrews (1991) but using a different method for computing the truncation lag. To ensure that the weights $|w_\ell|$ are decreasing, the autocorrelations have to be decreasing for increasing lag ℓ which can be achieved by using isotonic regression methods. In **sandwich**, these two weighting schemes are implemented in a function `weightsLumley` with a convenience interface `weave` (which stands for weighted empirical adaptive variance estimators) which again sets up the weights and then calls `vcovHAC`. Its most important arguments are `weave(lmobj, method, C, ...)` where `method` can be either `"truncate"` or `"smooth"` and `C` is by default 4 or 1 respectively.

To sum up, `vcovHAC` provides a simple yet flexible interface for general HAC estimation as defined in Equation (6). Arbitrary weights can be supplied either as vectors or functions for data-driven computation of the weights. As the latter might easily become rather complex, in particular due to the automatic choice of bandwidth or lag truncation parameters, three strategies suggested in the literature are readily available in **sandwich**: First, the Bartlett kernel weights suggested by Newey and West (1987, 1994) are used in `NeweyWest` which by default uses the bandwidth selection function `bwNeweyWest`. Second, the weighting scheme introduced by Andrews (1991) for kernel-based HAC estimation with automatic bandwidth selection is implemented in `weightsAndrews` and `bwAndrews` with corresponding convenience interface `kernHAC`. Third, the weighted empirical adaptive variance estimation scheme suggested by Lumley and Heagerty (1999) is available in `weightsLumley` with convenience interface `weave`.

It is illustrated in the following section how these functions can be easily used in applications.

4. Applications and illustrations

In econometric analyses, the practitioner is only seldom interested in the covariance matrix $\hat{\Psi}$ (or $\hat{\Omega}$ or $\hat{\Phi}$) *per se*, but mainly wants to compute them to use them for inferential procedures. Therefore, it is important that the functions `vcovHC` and `vcovHAC` described in the previous section can be easily supplied to other procedures such that the user does not necessarily have to compute the variances in advance.

A typical field of application for HC and HAC covariances are partial t or z tests for assessing whether a parameter β_j is significantly different from zero. Exploiting the (asymptotic)

normality of the estimates, these tests are based on the t ratio $\hat{\beta}_j/\sqrt{\hat{\Psi}_{jj}}$ and either use the asymptotic normal distribution or the t distribution with $n-k$ degrees of freedom for computing p values (White 2000). This procedure is available in the R package **lmtest** (Zeileis and Hothorn 2002) in the generic function `coeftest` which has a default method applicable to fitted `"lm"` objects.

```
coeftest(lmobj, vcov = NULL, df = NULL, ...)
```

where `vcov` specifies the covariances either as a matrix (corresponding to the covariance matrix estimate) or as a function computing it from `lmobj` (corresponding to the covariance matrix estimator). By default, it uses the `vcov` method which computes $\hat{\Psi}_{\mathrm{const}}$ assuming spherical errors. The `df` argument determines the degrees of freedom: if `df` is finite and positive, a t distribution with `df` degrees of freedom is used, otherwise a normal approximation is employed. The default is to set `df` to $n-k$.

Inference based on HC and HAC estimators is illustrated in the following using three real-world data sets: testing coefficients in two models from Greene (1993) and a structural change problem from Bai and Perron (2003).

To make the results exactly reproducible for the reader, the commands for the inferential procedures is given along with their output within the text. A full list of commands, including those which produce the figures in the text, are provided (without output) in the appendix along with the versions of R and the packages used. Before we start with the examples, the **sandwich** and **lmtest** package have to be loaded:

```
R> library("sandwich")
R> library("lmtest")
```

4.1. Testing coefficients in cross-sectional data

A quadratic regression model for per capita expenditures on public schools explained by per capita income in the United States in 1979 has been analyzed by Greene (1993) and re-analyzed in Cribari-Neto (2004). The corresponding cross-sectional data for the 51 US states is given in Table 14.1 in Greene (1993) and available in **sandwich** in the data frame `PublicSchools` which can be loaded by:

```
R> data("PublicSchools")
R> ps <- na.omit(PublicSchools)
R> ps$Income <- ps$Income * 0.0001
```

where the second line omits a missing value (`NA`) in Wisconsin and assigns the result to a new data frame `ps` and the third line transforms the income to be in USD $10,000$. The quadratic regression can now easily be fit using the function `lm` which fits linear regression models specified by a symbolic formula via OLS.

```
R> fm.ps <- lm(Expenditure ~ Income + I(Income^2), data = ps)
```

The fitted `"lm"` object `fm.ps` now contains the regression of the variable `Expenditure` on the variable `Income` and its sqared value, both variables are taken from the data frame `ps`.

The question in this data set is whether the quadratic term is really needed, i.e., whether the coefficient of `I(Income^2)` is significantly different from zero. The partial quasi-t tests (or z tests) for all coefficients can be computed using the function `coeftest`. Greene (1993) assesses the significance using the HC0 estimator of White (1980).

```
R> coeftest(fm.ps, df = Inf, vcov = vcovHC(fm.ps, type = "HC0"))

z test of coefficients:

            Estimate Std. Error z value Pr(>|z|)
(Intercept)   832.91     460.89  1.8072  0.07073 .
Income      -1834.20    1243.04 -1.4756  0.14006
I(Income^2)  1587.04     829.99  1.9121  0.05586 .
---
Signif. codes:  0 '***' 0.001 '**' 0.01 '*' 0.05 '.' 0.1 ' ' 1
```

The `vcov` argument specifies the covariance matrix as a matrix (as opposed to a function) which is returned by `vcovHC(fm.ps, type = "HC0")`. As `df` is set to infinity (`Inf`) a normal approximation is used for computing the p values which seem to suggest that the quadratic term might be weakly significant. In his analysis, Cribari-Neto (2004) uses his HC4 estimator (among others) giving the following result:

```
R> coeftest(fm.ps, df = Inf, vcov = vcovHC(fm.ps, type = "HC4"))

z test of coefficients:

            Estimate Std. Error z value Pr(>|z|)
(Intercept)   832.91    3008.01  0.2769   0.7819
Income      -1834.20    8183.19 -0.2241   0.8226
I(Income^2)  1587.04    5488.93  0.2891   0.7725
```

The quadratic term is clearly non-significant. The reason for this result is depicted in Figure 2 which shows the data along with the fitted linear and quadratic model—the latter being obviously heavily influenced by a single outlier: Alaska. Thus, the improved performance of the HC4 as compared to the HC0 estimator is due to the correction for high leverage points.

4.2. Testing coefficients in time-series data

Greene (1993) also anayzes a time-series regression model based on robust covariance matrix estimates: his Table 15.1 provides data on the nominal gross national product (GNP), nominal gross private domestic investment, a price index and an interest rate which is used to formulate a model that explains real investment by real GNP and real interest. The corresponding transformed variables `RealInv`, `RealGNP` and `RealInt` are stored in the data frame `Investment` in **sandwich** which can be loaded by:

```
R> data("Investment")
```

Subsequently, the fitted linear regression model is computed by:

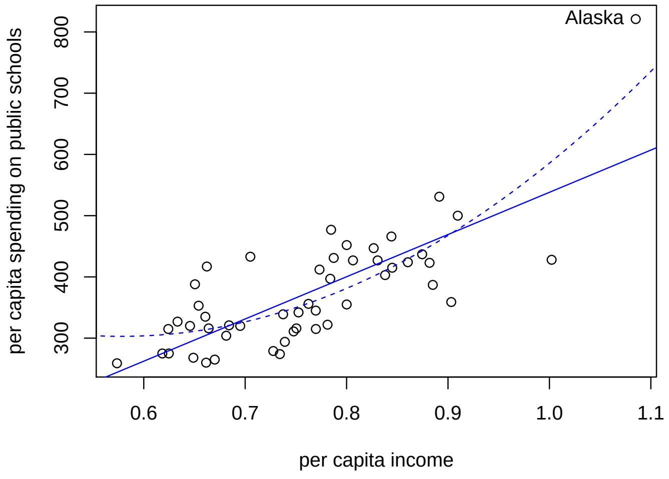

Figure 2: Expenditure on public schools and income with fitted models.

```
R> fm.inv <- lm(RealInv ~ RealGNP + RealInt, data = Investment)
```

and the significance of the coefficients can again be assessed by partial z tests using `coeftest`. Greene (1993) uses the estimator of Newey and West (1987) without prewhitening and with lag $L = 4$ for this purpose which is here passed as a matrix (as opposed to a function) to `coeftest`.

```
R> coeftest(fm.inv, df = Inf, vcov = NeweyWest(fm.inv, lag = 4, prewhite = FALSE))

z test of coefficients:

              Estimate Std. Error z value Pr(>|z|)
(Intercept) -12.533601  18.958298 -0.6611   0.5085
RealGNP       0.169136   0.016751 10.0972   <2e-16 ***
RealInt      -1.001438   3.342375 -0.2996   0.7645
---
Signif. codes:  0 '***' 0.001 '**' 0.01 '*' 0.05 '.' 0.1 ' ' 1
```

If alternatively the automatic bandwidth selection procedure of Newey and West (1994) with prewhitening should be used, this can be passed as a function to `coeftest`.

```
R> coeftest(fm.inv, df = Inf, vcov = NeweyWest)

z test of coefficients:

              Estimate Std. Error z value  Pr(>|z|)
(Intercept) -12.533601  24.374177 -0.5142    0.6071
```

```
RealGNP        0.169136   0.023586  7.1709 7.449e-13 ***
RealInt       -1.001438   3.639935 -0.2751    0.7832
---
Signif. codes:  0 '***' 0.001 '**' 0.01 '*' 0.05 '.' 0.1 ' ' 1
```

For illustration purposes, we show how a new function implementing a particular HAC estimator can be easily set up using the tools provided by **sandwich**. This is particularly helpful if the same estimator is to be applied several times in the course of an analysis. Suppose, we want to use a Parzen kernel with VAR(2) prewhitening, no finite sample adjustment and automatic bandwidth selection according to Newey and West (1994). First, we set up the function `parzenHAC` and then pass this function to `coeftest`.

```
R> parzenHAC <- function(x, ...) kernHAC(x, kernel = "Parzen", prewhite = 2,
+     adjust = FALSE, bw = bwNeweyWest, ...)
R> coeftest(fm.inv, df = Inf, vcov = parzenHAC)


z test of coefficients:

              Estimate Std. Error z value  Pr(>|z|)
(Intercept) -12.533601  24.663944 -0.5082    0.6113
RealGNP       0.169136   0.020835  8.1181 4.737e-16 ***
RealInt      -1.001438   3.947469 -0.2537    0.7997
---
Signif. codes:  0 '***' 0.001 '**' 0.01 '*' 0.05 '.' 0.1 ' ' 1
```

The three estimators leads to slightly different standard errors, but all tests agree that real GNP has a highly significant influence while the real interest rate has not. The data along with the fitted regression are depicted in Figure 3.

4.3. Testing and dating structural changes in the presence of heteroskedasticity and autocorrelation

To illustrate that the functionality provided by the covariance estimators implemented in **sandwich** cannot only be used in simple settings, such as partial quasi-t tests, but also for more complicated tasks, we employ the real interest time series analyzed by Bai and Perron (2003). This series contains changes in the mean (see Figure 4, right panel) which Bai and Perron (2003) detect using several structural change tests based on F statistics and date using a dynamic programming algorithm. As the visualization suggests, this series exhibits both heteroskedasticity and autocorrelation, hence Bai and Perron (2003) use a quadratic spectral kernel HAC estimator in their analysis. Here, we use the same dating procedure but assess the significance using an OLS-based CUSUM test (Ploberger and Krämer 1992) based on the same HAC estimator. The data are available in the package **strucchange** as the quarterly time series `RealInt` containing the US ex-post real interest rate from 1961(1) to 1986(3) and they are analyzed by a simple regression on the mean.

Under the assumptions in the classical linear model with spherical errors, the test statistic of

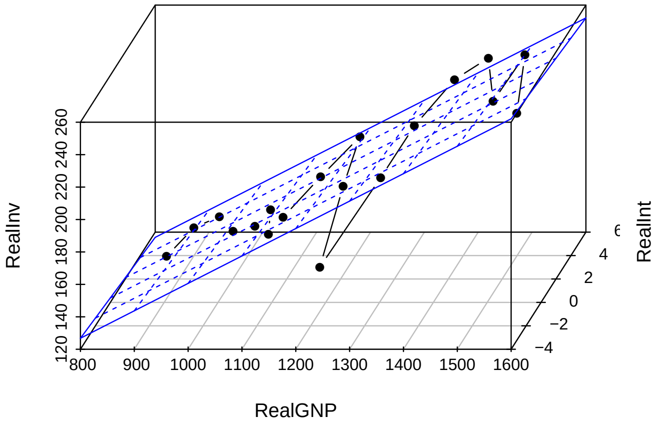

Figure 3: Investment equation data with fitted model.

the OLS-based CUSUM test is

$$\sup_{j=1,\dots,n} \left| \frac{1}{\sqrt{n\,\hat{\sigma}^2}} \sum_{i=1}^{j} \hat{u}_i \right|. \tag{9}$$

If autocorrelation and heteroskedasticity are present in the data, a robust variance estimator should be used: if x_i is equal to unity, this can simply be achieved by replacing $\hat{\sigma}^2$ with $\hat{\Phi}$ or $n\hat{\Psi}$ respectively. Here, we use the quadratic spectral kernel HAC estimator of Andrews (1991) with VAR(1) prewhitening and automatic bandwidth selection based on an AR(1) approximation as implemented in the function `kernHAC`. The p values for the OLS-based CUSUM test can be computed from the distribution of the supremum of a Brownian bridge (see e.g., Ploberger and Krämer 1992). This and other methods for testing, dating and monitoring structural changes are implemented in the R package **strucchange** (Zeileis, Leisch, Hornik, and Kleiber 2002) which contains the function `gefp` for fitting and assessing fluctuation processes including OLS-based CUSUM processes (see Zeileis 2006a, for more details).

After loading the package and the data,

```
R> library("strucchange")
R> data("RealInt")
```

the command

```
R> ocus <- gefp(RealInt ~ 1, fit = lm, vcov = kernHAC)
```

fits the OLS-based CUSUM process for a regression on the mean (`RealInt ~ 1`), using the function `lm` and estimating the variance using the function `kernHAC`. The fitted OLS-based CUSUM process can then be visualized together with its 5% critical value (horizontal lines)

by `plot(scus)` which leads to a similar plot as in the left panel of Figure 4 (see the appendix for more details). As the process crosses its boundary, there is a significant change in the mean, while the clear peak in the process conveys that there is at least one strong break in the early 1980s. A formal significance test can also be carried out by `sctest(ocus)` which leads to a highly significant p value of 0.0082. Similarly, the same quadratic spectral kernel HAC estimator could also be used for computing and visualizing the sup$F$ test of Andrews (1993), the code is provided in the appendix.

Finally, the breakpoints in this model along with their confidence intervals can be computed by:

```
R> bp <- breakpoints(RealInt ~ 1)
R> confint(bp, vcov = kernHAC)

         Confidence intervals for breakpoints
         of optimal 3-segment partition:

Call:
confint.breakpointsfull(object = bp, vcov. = kernHAC)

Breakpoints at observation number:
  2.5 % breakpoints 97.5 %
1    37          47     48
2    77          79     81

Corresponding to breakdates:
  2.5 %   breakpoints 97.5 %
1 1970(1) 1972(3)     1972(4)
2 1980(1) 1980(3)     1981(1)
```

The dating algorithm `breakpoints` implements the procedure described in Bai and Perron (2003) and estimates the timing of the structural changes by OLS. Therefore, in this step no covariance matrix estimate is required, but for computing the confidence intervals using a consistent covariance matrix estimator is again essential. The `confint` method for computing confidence intervals takes again a `vcov` argument which has to be a function (and not a matrix) because it has to be applied to several segments of the data. By default, it computes the breakpoints for the minimum BIC partition which gives in this case two breaks.[6] The fitted three-segment model along with the breakpoints and their confidence intervals is depicted in the right panel of Figure 4.

5. Summary

This paper briefly reviews a class of heteroskedasticity-consistent (HC) and a class of heteroskedasticity and autocorrelation consistent (HAC) covariance matrix estimators suggested

[6] By choosing the number of breakpoints with sequential tests and not the BIC, Bai and Perron (2003) arrive at a model with an additional breakpoint which has rather wide confidence intervals (see also Zeileis and Kleiber 2005)

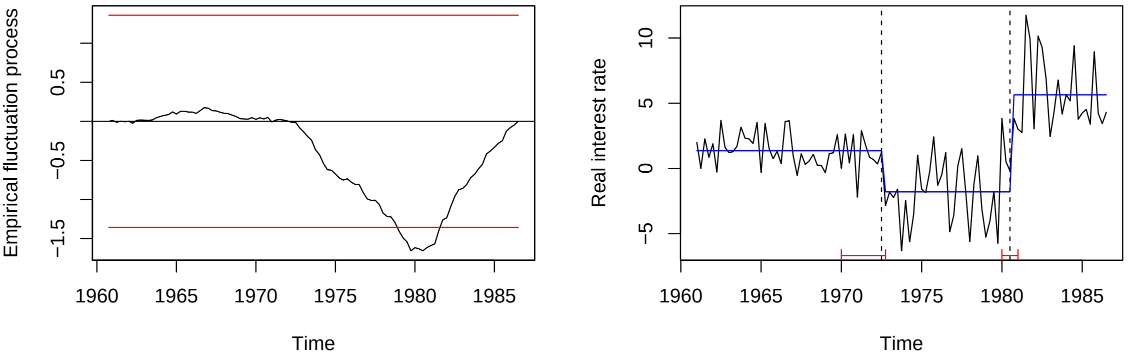

Figure 4: OLS-based CUSUM test (left) and fitted model (right) for real interest data.

in the econometric literature over the last 20 years and introduces unified computational tools that reflect the flexibility and the conceptual ideas of the underlying theoretical frameworks. Based on these general tools, a number of special cases of HC and HAC estimators is provided including the most popular in applied econometric research. All the functions suggested are implemented in the package **sandwich** in the R system for statistical computing and designed in such a way that they build on readily available model fitting functions and provide building blocks that can be easily integrated into other programs or applications. To achieve this flexibility, the object orientation mechanism of R and the fact that functions are first-level objects are of prime importance.

Acknowledgments

We are grateful to Thomas Lumley for putting his code in the **weave** package at disposal and for advice in the design of **sandwich**, and to Christian Kleiber for helpful suggestions in the development of **sandwich**.

References

Andrews DWK (1991). "Heteroskedasticity and Autocorrelation Consistent Covariance Matrix Estimation." *Econometrica*, **59**, 817–858.

Andrews DWK (1993). "Tests for Parameter Instability and Structural Change With Unknown Change Point." *Econometrica*, **61**, 821–856.

Andrews DWK, Monahan JC (1992). "An Improved Heteroskedasticity and Autocorrelation Consistent Covariance Matrix Estimator." *Econometrica*, **60**(4), 953–966.

Bai J, Perron P (2003). "Computation and Analysis of Multiple Structural Change Models." *Journal of Applied Econometrics*, **18**, 1–22.

Cribari-Neto F (2004). "Asymptotic Inference Under Heteroskedasticity of Unknown Form." *Computational Statistics & Data Analysis*, **45**, 215–233.

Cribari-Neto F, Zarkos SG (1999). "R: Yet Another Econometric Programming Environment." *Journal of Applied Econometrics*, **14**, 319–329.

Cribari-Neto F, Zarkos SG (2003). "Econometric and Statistical Computing Using Ox." *Computational Economics*, **21**, 277–295.

Fox J (2002). *An R and S-PLUS Companion to Applied Regression.* Sage Publications, Thousand Oaks, CA.

Greene WH (1993). *Econometric Analysis.* 2nd edition. Macmillan Publishing Company, New York.

Long JS, Ervin LH (2000). "Using Heteroscedasticity Consistent Standard Errors in the Linear Regression Model." *The American Statistician*, **54**, 217–224.

Lumley T, Heagerty P (1999). "Weighted Empirical Adaptive Variance Estimators for Correlated Data Regression." *Journal of the Royal Statistical Society B*, **61**, 459–477.

MacKinnon JG, White H (1985). "Some Heteroskedasticity-Consistent Covariance Matrix Estimators with Improved Finite Sample Properties." *Journal of Econometrics*, **29**, 305–325.

Newey WK, West KD (1987). "A Simple, Positive-Definite, Heteroskedasticity and Autocorrelation Consistent Covariance Matrix." *Econometrica*, **55**, 703–708.

Newey WK, West KD (1994). "Automatic Lag Selection in Covariance Matrix Estimation." *Review of Economic Studies*, **61**, 631–653.

Ploberger W, Krämer W (1992). "The CUSUM Test With OLS Residuals." *Econometrica*, **60**, 271–285.

Racine J, Hyndman R (2002). "Using R to Teach Econometrics." *Journal of Applied Econometrics*, **17**, 175–189.

R Development Core Team (2008). *R: A Language and Environment for Statistical Computing.* R Foundation for Statistical Computing, Vienna, Austria. ISBN 3-900051-07-0, URL http://www.R-project.org/.

White H (1980). "A Heteroskedasticity-Consistent Covariance Matrix and a Direct Test for Heteroskedasticity." *Econometrica*, **48**, 817–838.

White H (2000). *Asymptotic Theory for Econometricians.* Revised edition. Academic Press, New York.

White H, Domowitz I (1984). "Nonlinear Regression with Dependent Observations." *Econometrica*, **52**, 143–161.

Zeileis A (2004). "Econometric Computing with HC and HAC Covariance Matrix Estimators." *Journal of Statistical Software*, **11**(10), 1–17. URL http://www.jstatsoft.org/v11/i10/.

Zeileis A (2006a). "Implementing a Class of Structural Change Tests: An Econometric Computing Approach." *Computational Statistics & Data Analysis*, **50**, 2987–3008. doi:10.1016/j.csda.2005.07.001.

Zeileis A (2006b). "Object-oriented Computation of Sandwich Estimators." *Journal of Statistical Software*, **16**(9), 1–16. URL http://www.jstatsoft.org/v16/i09/.

Zeileis A, Hothorn T (2002). "Diagnostic Checking in Regression Relationships." *R News*, **2**(3), 7–10. URL http://CRAN.R-project.org/doc/Rnews/.

Zeileis A, Kleiber C (2005). "Validating Multiple Structural Change Models – A Case Study." *Journal of Applied Econometrics*, **20**, 685–690. doi:10.1002/jae.856.

Zeileis A, Leisch F, Hornik K, Kleiber C (2002). "**strucchange**: An R Package for Testing for Structural Change in Linear Regression Models." *Journal of Statistical Software*, **7**(2), 1–38. URL http://www.jstatsoft.org/v07/i02/.

A. R code

The packages **sandwich**, **lmtest** and **strucchange** are required for the applications in this paper. Furthermore, the packages depend on **zoo**. For the computations in this paper R 3.1.2 and **sandwich** 2.3–3, **lmtest** 0.9–33, **strucchange** 1.5–0 and **zoo** 1.7–12 have been used. R itself and all packages used are available from CRAN at `http://CRAN.R-project.org/`.

To make the packages available for the examples the following commands are necessary:

```
library("sandwich")
library("lmtest")
library("strucchange")
```

A.1. Testing coefficients in cross-sectional data

Load public schools data, omit `NA` in Wisconsin and scale income:

```
data("PublicSchools")
ps <- na.omit(PublicSchools)
ps$Income <- ps$Income * 0.0001
```

Fit quadratic regression model:

```
fm.ps <- lm(Expenditure ~ Income + I(Income^2), data = ps)
```

Compare standard errors:

```
sqrt(diag(vcov(fm.ps)))
sqrt(diag(vcovHC(fm.ps, type = "const")))
sqrt(diag(vcovHC(fm.ps, type = "HC0")))
sqrt(diag(vcovHC(fm.ps, type = "HC3")))
sqrt(diag(vcovHC(fm.ps, type = "HC4")))
```

Test coefficient of quadratic term:

```
coeftest(fm.ps, df = Inf, vcov = vcovHC(fm.ps, type = "HC0"))
coeftest(fm.ps, df = Inf, vcov = vcovHC(fm.ps, type = "HC4"))
```

Visualization:

```
plot(Expenditure ~ Income, data = ps,
  xlab = "per capita income",
  ylab = "per capita spending on public schools")
inc <- seq(0.5, 1.2, by = 0.001)
lines(inc, predict(fm.ps, data.frame(Income = inc)), col = 4, lty = 2)
fm.ps2 <- lm(Expenditure ~ Income, data = ps)
abline(fm.ps2, col = 4)
text(ps[2,2], ps[2,1], rownames(ps)[2], pos = 2)
```

A.2. Testing coefficients in time-series data

Load investment equation data:

```
data("Investment")
```

Fit regression model:

```
fm.inv <- lm(RealInv ~ RealGNP + RealInt, data = Investment)
```

Test coefficients using Newey-West HAC estimator with user-defined and data-driven bandwidth and with Parzen kernel:

```
coeftest(fm.inv, df = Inf, vcov = NeweyWest(fm.inv, lag = 4, prewhite = FALSE))
coeftest(fm.inv, df = Inf, vcov = NeweyWest)

parzenHAC <- function(x, ...) kernHAC(x, kernel = "Parzen", prewhite = 2,
  adjust = FALSE, bw = bwNeweyWest, ...)
coeftest(fm.inv, df = Inf, vcov = parzenHAC)
```

Time-series visualization:

```
plot(Investment[, "RealInv"], type = "b", pch = 19, ylab = "Real investment")
lines(ts(fitted(fm.inv), start = 1964), col = 4)
```

3-dimensional visualization:

```
library("scatterplot3d")
s3d <- scatterplot3d(Investment[,c(5,7,6)],
  type = "b", angle = 65, scale.y = 1, pch = 16)
s3d$plane3d(fm.inv, lty.box = "solid", col = 4)
```

A.3. Testing and dating structural changes in the presence of heteroskedasticity and autocorrelation

Load real interest series:

```
data("RealInt")
```

OLS-based CUSUM test with quadratic spectral kernel HAC estimate:

```
ocus <- gefp(RealInt ~ 1, fit = lm, vcov = kernHAC)
plot(ocus, aggregate = FALSE)
sctest(ocus)
```

sup$F$ test with quadratic spectral kernel HAC estimate:

```
fs <- Fstats(RealInt ~ 1, vcov = kernHAC)
plot(fs)
sctest(fs)
```

Breakpoint estimation and confidence intervals with quadratic spectral kernel HAC estimate:

```
bp <- breakpoints(RealInt ~ 1)
confint(bp, vcov = kernHAC)
plot(bp)
```

Visualization:

```
plot(RealInt, ylab = "Real interest rate")
lines(ts(fitted(bp), start = start(RealInt), freq = 4), col = 4)
lines(confint(bp, vcov = kernHAC))
```

A.4. Integrating covariance matrix estimators in other functions

If programmers want to allow for the same flexibility regarding the specification of covariance matrices in their own functions as illustrated in `coeftest`, only a few simple additions have to be made which are illustrated in the following. Say, a function `foo(lmobj, vcov = NULL, ...)` wants to compute some quantity involving the standard errors associated with the `"lm"` object `lmobj`. Then, `vcov` should use by default the standard `vcov` method for `"lm"` objects, otherwise `vcov` is assumed to be either a function returning the covariance matrix estimate or the estimate itself. The following piece of code is sufficient for computing the standard errors.

```
if(is.null(vcov)) {
  se <- vcov(lmobj)
} else {
  if (is.function(vcov))
    se <- vcov(lmobj)
  else
    se <- vcov
}
se <- sqrt(diag(se))
```

In the first step the default method is called: note, that R can automatically distinguish between the variable `vcov` (which is `NULL`) and the generic function `vcov` (from the **stats** package which dispatches to the `"lm"` method) that is called here. Otherwise, it is just distinguished between a function or non-function. In the final step the square root of the diagonal elements is computed and stored in the vector `se` which can subsequently used for further computation in `foo()`.

Affiliation:

Achim Zeileis
Department of Statistics
Faculty of Economics and Statistics
Universität Innsbruck

Universitätsstr. 15
6020 Innsbruck, Austria
E-mail: Achim.Zeileis@R-project.org
URL: http://eeecon.uibk.ac.at/~zeileis/
