# Supplementary material for: Comparing Kaolin and Pinolene to Improve Sustainable Grapevine Production during Drought
Source: PLoS One. 2016 Jun 13;11(6):e0156631. doi: 10.1371/journal.pone.0156631 (PMC4905681; doi:10.1371/journal.pone.0156631)
Supplement: S1 File — (GZ) [file pone.0156631.s003.gz › PONE_Brillante_et_al_2016_Code_and_Data/packrat/src/stringi/stringi_0.5-5.tar.gz!/stringi/src/icu55/license.html]

ICU License - ICU 1.8.1 and later


## ICU License - ICU 1.8.1 and later

COPYRIGHT AND PERMISSION NOTICE

Copyright (c) 1995-2015 International Business Machines Corporation and others

All rights reserved.

Permission is hereby granted, free of charge, to any person obtaining a copy
of this software and associated documentation files (the "Software"),
to deal in the Software without restriction, including without limitation
the rights to use, copy, modify, merge, publish, distribute, and/or sell
copies of the Software, and to permit persons
to whom the Software is furnished to do so, provided that the above
copyright notice(s) and this permission notice appear in all copies
of the Software and that both the above copyright notice(s) and this
permission notice appear in supporting documentation.

THE SOFTWARE IS PROVIDED "AS IS", WITHOUT WARRANTY OF ANY KIND, EXPRESS OR IMPLIED,
INCLUDING BUT NOT LIMITED TO THE WARRANTIES OF MERCHANTABILITY, FITNESS FOR A
PARTICULAR PURPOSE AND NONINFRINGEMENT OF THIRD PARTY RIGHTS. IN NO EVENT SHALL
THE COPYRIGHT HOLDER OR HOLDERS INCLUDED IN THIS NOTICE BE LIABLE FOR ANY CLAIM,
OR ANY SPECIAL INDIRECT OR CONSEQUENTIAL DAMAGES, OR ANY DAMAGES WHATSOEVER
RESULTING FROM LOSS OF USE, DATA OR PROFITS, WHETHER IN AN ACTION OF CONTRACT,
NEGLIGENCE OR OTHER TORTIOUS ACTION, ARISING OUT OF OR IN CONNECTION WITH THE
USE OR PERFORMANCE OF THIS SOFTWARE.

Except as contained in this notice, the name of a copyright holder shall not be
used in advertising or otherwise to promote the sale, use or other dealings in
this Software without prior written authorization of the copyright holder.

---

All trademarks and registered trademarks mentioned herein are the property of their respective owners.

---

## Third-Party Software Licenses

This section contains third-party software notices and/or additional terms for licensed
third-party software components included within ICU libraries.

### 1. Unicode Data Files and Software

```
COPYRIGHT AND PERMISSION NOTICE

Copyright © 1991-2015 Unicode, Inc. All rights reserved.
Distributed under the Terms of Use in 
http://www.unicode.org/copyright.html.

Permission is hereby granted, free of charge, to any person obtaining
a copy of the Unicode data files and any associated documentation
(the "Data Files") or Unicode software and any associated documentation
(the "Software") to deal in the Data Files or Software
without restriction, including without limitation the rights to use,
copy, modify, merge, publish, distribute, and/or sell copies of
the Data Files or Software, and to permit persons to whom the Data Files
or Software are furnished to do so, provided that
(a) this copyright and permission notice appear with all copies 
of the Data Files or Software,
(b) this copyright and permission notice appear in associated 
documentation, and
(c) there is clear notice in each modified Data File or in the Software
as well as in the documentation associated with the Data File(s) or
Software that the data or software has been modified.

THE DATA FILES AND SOFTWARE ARE PROVIDED "AS IS", WITHOUT WARRANTY OF
ANY KIND, EXPRESS OR IMPLIED, INCLUDING BUT NOT LIMITED TO THE
WARRANTIES OF MERCHANTABILITY, FITNESS FOR A PARTICULAR PURPOSE AND
NONINFRINGEMENT OF THIRD PARTY RIGHTS.
IN NO EVENT SHALL THE COPYRIGHT HOLDER OR HOLDERS INCLUDED IN THIS
NOTICE BE LIABLE FOR ANY CLAIM, OR ANY SPECIAL INDIRECT OR CONSEQUENTIAL
DAMAGES, OR ANY DAMAGES WHATSOEVER RESULTING FROM LOSS OF USE,
DATA OR PROFITS, WHETHER IN AN ACTION OF CONTRACT, NEGLIGENCE OR OTHER
TORTIOUS ACTION, ARISING OUT OF OR IN CONNECTION WITH THE USE OR
PERFORMANCE OF THE DATA FILES OR SOFTWARE.

Except as contained in this notice, the name of a copyright holder
shall not be used in advertising or otherwise to promote the sale,
use or other dealings in these Data Files or Software without prior
written authorization of the copyright holder.
```

### 2. Chinese/Japanese Word Break Dictionary Data (cjdict.txt)

```
 #    The Google Chrome software developed by Google is licensed under the BSD license. Other software included in this distribution is provided under other licenses, as set forth below.
 #	
 #	The BSD License
 #	http://opensource.org/licenses/bsd-license.php 
 #	Copyright (C) 2006-2008, Google Inc.
 #	
 #	All rights reserved.
 #	
 #	Redistribution and use in source and binary forms, with or without modification, are permitted provided that the following conditions are met:
 #	
 #	Redistributions of source code must retain the above copyright notice, this list of conditions and the following disclaimer.
 #	Redistributions in binary form must reproduce the above copyright notice, this list of conditions and the following disclaimer in the documentation and/or other materials provided with the distribution.
 #	Neither the name of  Google Inc. nor the names of its contributors may be used to endorse or promote products derived from this software without specific prior written permission.
 #	 
 #	
 #	THIS SOFTWARE IS PROVIDED BY THE COPYRIGHT HOLDERS AND CONTRIBUTORS "AS IS" AND ANY EXPRESS OR IMPLIED WARRANTIES, INCLUDING, BUT NOT LIMITED TO, THE IMPLIED WARRANTIES OF MERCHANTABILITY AND FITNESS FOR A PARTICULAR PURPOSE ARE DISCLAIMED. IN NO EVENT SHALL THE COPYRIGHT OWNER OR CONTRIBUTORS BE LIABLE FOR ANY DIRECT, INDIRECT, INCIDENTAL, SPECIAL, EXEMPLARY, OR CONSEQUENTIAL DAMAGES (INCLUDING, BUT NOT LIMITED TO, PROCUREMENT OF SUBSTITUTE GOODS OR SERVICES; LOSS OF USE, DATA, OR PROFITS; OR BUSINESS INTERRUPTION) HOWEVER CAUSED AND ON ANY THEORY OF LIABILITY, WHETHER IN CONTRACT, STRICT LIABILITY, OR TORT (INCLUDING NEGLIGENCE OR OTHERWISE) ARISING IN ANY WAY OUT OF THE USE OF THIS SOFTWARE, EVEN IF ADVISED OF THE POSSIBILITY OF SUCH DAMAGE.
 #	
 #	                                             
 #	The word list in cjdict.txt are generated by combining three word lists listed
 #	below with further processing for compound word breaking. The frequency is generated
 #	with an iterative training against Google web corpora. 
 #	
 #	* Libtabe (Chinese)
 #	  - https://sourceforge.net/project/?group_id=1519
 #	  - Its license terms and conditions are shown below.
 #	
 #	* IPADIC (Japanese)
 #	  - http://chasen.aist-nara.ac.jp/chasen/distribution.html
 #	  - Its license terms and conditions are shown below.
 #	
 #	---------COPYING.libtabe ---- BEGIN--------------------
 #	
 #	/*
 #	 * Copyrighy (c) 1999 TaBE Project.
 #	 * Copyright (c) 1999 Pai-Hsiang Hsiao.
 #	 * All rights reserved.
 #	 *
 #	 * Redistribution and use in source and binary forms, with or without
 #	 * modification, are permitted provided that the following conditions
 #	 * are met:
 #	 *
 #	 * . Redistributions of source code must retain the above copyright
 #	 *   notice, this list of conditions and the following disclaimer.
 #	 * . Redistributions in binary form must reproduce the above copyright
 #	 *   notice, this list of conditions and the following disclaimer in
 #	 *   the documentation and/or other materials provided with the
 #	 *   distribution.
 #	 * . Neither the name of the TaBE Project nor the names of its
 #	 *   contributors may be used to endorse or promote products derived
 #	 *   from this software without specific prior written permission.
 #	 *
 #	 * THIS SOFTWARE IS PROVIDED BY THE COPYRIGHT HOLDERS AND CONTRIBUTORS
 #	 * "AS IS" AND ANY EXPRESS OR IMPLIED WARRANTIES, INCLUDING, BUT NOT
 #	 * LIMITED TO, THE IMPLIED WARRANTIES OF MERCHANTABILITY AND FITNESS
 #	 * FOR A PARTICULAR PURPOSE ARE DISCLAIMED. IN NO EVENT SHALL THE
 #	 * REGENTS OR CONTRIBUTORS BE LIABLE FOR ANY DIRECT, INDIRECT,
 #	 * INCIDENTAL, SPECIAL, EXEMPLARY, OR CONSEQUENTIAL DAMAGES
 #	 * (INCLUDING, BUT NOT LIMITED TO, PROCUREMENT OF SUBSTITUTE GOODS OR
 #	 * SERVICES; LOSS OF USE, DATA, OR PROFITS; OR BUSINESS INTERRUPTION)
 #	 * HOWEVER CAUSED AND ON ANY THEORY OF LIABILITY, WHETHER IN CONTRACT,
 #	 * STRICT LIABILITY, OR TORT (INCLUDING NEGLIGENCE OR OTHERWISE)
 #	 * ARISING IN ANY WAY OUT OF THE USE OF THIS SOFTWARE, EVEN IF ADVISED
 #	 * OF THE POSSIBILITY OF SUCH DAMAGE.
 #	 */
 #	
 #	/*
 #	 * Copyright (c) 1999 Computer Systems and Communication Lab,
 #	 *                    Institute of Information Science, Academia Sinica.
 #	 * All rights reserved.
 #	 *
 #	 * Redistribution and use in source and binary forms, with or without
 #	 * modification, are permitted provided that the following conditions
 #	 * are met:
 #	 *
 #	 * . Redistributions of source code must retain the above copyright
 #	 *   notice, this list of conditions and the following disclaimer.
 #	 * . Redistributions in binary form must reproduce the above copyright
 #	 *   notice, this list of conditions and the following disclaimer in
 #	 *   the documentation and/or other materials provided with the
 #	 *   distribution.
 #	 * . Neither the name of the Computer Systems and Communication Lab
 #	 *   nor the names of its contributors may be used to endorse or
 #	 *   promote products derived from this software without specific
 #	 *   prior written permission.
 #	 *
 #	 * THIS SOFTWARE IS PROVIDED BY THE COPYRIGHT HOLDERS AND CONTRIBUTORS
 #	 * "AS IS" AND ANY EXPRESS OR IMPLIED WARRANTIES, INCLUDING, BUT NOT
 #	 * LIMITED TO, THE IMPLIED WARRANTIES OF MERCHANTABILITY AND FITNESS
 #	 * FOR A PARTICULAR PURPOSE ARE DISCLAIMED. IN NO EVENT SHALL THE
 #	 * REGENTS OR CONTRIBUTORS BE LIABLE FOR ANY DIRECT, INDIRECT,
 #	 * INCIDENTAL, SPECIAL, EXEMPLARY, OR CONSEQUENTIAL DAMAGES
 #	 * (INCLUDING, BUT NOT LIMITED TO, PROCUREMENT OF SUBSTITUTE GOODS OR
 #	 * SERVICES; LOSS OF USE, DATA, OR PROFITS; OR BUSINESS INTERRUPTION)
 #	 * HOWEVER CAUSED AND ON ANY THEORY OF LIABILITY, WHETHER IN CONTRACT,
 #	 * STRICT LIABILITY, OR TORT (INCLUDING NEGLIGENCE OR OTHERWISE)
 #	 * ARISING IN ANY WAY OUT OF THE USE OF THIS SOFTWARE, EVEN IF ADVISED
 #	 * OF THE POSSIBILITY OF SUCH DAMAGE.
 #	 */
 #	
 #	Copyright 1996 Chih-Hao Tsai @ Beckman Institute, University of Illinois
 #	c-tsai4@uiuc.edu  http://casper.beckman.uiuc.edu/~c-tsai4
 #	
 #	---------------COPYING.libtabe-----END------------------------------------
 #	
 #	
 #	---------------COPYING.ipadic-----BEGIN------------------------------------
 #	
 #	Copyright 2000, 2001, 2002, 2003 Nara Institute of Science
 #	and Technology.  All Rights Reserved.
 #	
 #	Use, reproduction, and distribution of this software is permitted.
 #	Any copy of this software, whether in its original form or modified,
 #	must include both the above copyright notice and the following
 #	paragraphs.
 #	
 #	Nara Institute of Science and Technology (NAIST),
 #	the copyright holders, disclaims all warranties with regard to this
 #	software, including all implied warranties of merchantability and
 #	fitness, in no event shall NAIST be liable for
 #	any special, indirect or consequential damages or any damages
 #	whatsoever resulting from loss of use, data or profits, whether in an
 #	action of contract, negligence or other tortuous action, arising out
 #	of or in connection with the use or performance of this software.
 #	
 #	A large portion of the dictionary entries
 #	originate from ICOT Free Software.  The following conditions for ICOT
 #	Free Software applies to the current dictionary as well.
 #	
 #	Each User may also freely distribute the Program, whether in its
 #	original form or modified, to any third party or parties, PROVIDED
 #	that the provisions of Section 3 ("NO WARRANTY") will ALWAYS appear
 #	on, or be attached to, the Program, which is distributed substantially
 #	in the same form as set out herein and that such intended
 #	distribution, if actually made, will neither violate or otherwise
 #	contravene any of the laws and regulations of the countries having
 #	jurisdiction over the User or the intended distribution itself.
 #	
 #	NO WARRANTY
 #	
 #	The program was produced on an experimental basis in the course of the
 #	research and development conducted during the project and is provided
 #	to users as so produced on an experimental basis.  Accordingly, the
 #	program is provided without any warranty whatsoever, whether express,
 #	implied, statutory or otherwise.  The term "warranty" used herein
 #	includes, but is not limited to, any warranty of the quality,
 #	performance, merchantability and fitness for a particular purpose of
 #	the program and the nonexistence of any infringement or violation of
 #	any right of any third party.
 #	
 #	Each user of the program will agree and understand, and be deemed to
 #	have agreed and understood, that there is no warranty whatsoever for
 #	the program and, accordingly, the entire risk arising from or
 #	otherwise connected with the program is assumed by the user.
 #	
 #	Therefore, neither ICOT, the copyright holder, or any other
 #	organization that participated in or was otherwise related to the
 #	development of the program and their respective officials, directors,
 #	officers and other employees shall be held liable for any and all
 #	damages, including, without limitation, general, special, incidental
 #	and consequential damages, arising out of or otherwise in connection
 #	with the use or inability to use the program or any product, material
 #	or result produced or otherwise obtained by using the program,
 #	regardless of whether they have been advised of, or otherwise had
 #	knowledge of, the possibility of such damages at any time during the
 #	project or thereafter.  Each user will be deemed to have agreed to the
 #	foregoing by his or her commencement of use of the program.  The term
 #	"use" as used herein includes, but is not limited to, the use,
 #	modification, copying and distribution of the program and the
 #	production of secondary products from the program.
 #	
 #	In the case where the program, whether in its original form or
 #	modified, was distributed or delivered to or received by a user from
 #	any person, organization or entity other than ICOT, unless it makes or
 #	grants independently of ICOT any specific warranty to the user in
 #	writing, such person, organization or entity, will also be exempted
 #	from and not be held liable to the user for any such damages as noted
 #	above as far as the program is concerned.
 #	
 #	---------------COPYING.ipadic-----END------------------------------------
```

### 3. Lao Word Break Dictionary Data (laodict.txt)

```
 #	Copyright (c) 2013 International Business Machines Corporation
 #	and others. All Rights Reserved.
 #
 #	Project:    http://code.google.com/p/lao-dictionary/
 #	Dictionary: http://lao-dictionary.googlecode.com/git/Lao-Dictionary.txt
 #	License:    http://lao-dictionary.googlecode.com/git/Lao-Dictionary-LICENSE.txt
 #	            (copied below)
 #
 #	This file is derived from the above dictionary, with slight modifications.
 #	--------------------------------------------------------------------------------
 #	Copyright (C) 2013 Brian Eugene Wilson, Robert Martin Campbell.
 #	All rights reserved.
 #
 #	Redistribution and use in source and binary forms, with or without modification,
 #	are permitted provided that the following conditions are met:
 #
 #		Redistributions of source code must retain the above copyright notice, this
 #		list of conditions and the following disclaimer. Redistributions in binary
 #		form must reproduce the above copyright notice, this list of conditions and
 #		the following disclaimer in the documentation and/or other materials
 #		provided with the distribution.
 #
 #	THIS SOFTWARE IS PROVIDED BY THE COPYRIGHT HOLDERS AND CONTRIBUTORS "AS IS" AND
 #	ANY EXPRESS OR IMPLIED WARRANTIES, INCLUDING, BUT NOT LIMITED TO, THE IMPLIED
 #	WARRANTIES OF MERCHANTABILITY AND FITNESS FOR A PARTICULAR PURPOSE ARE
 #	DISCLAIMED. IN NO EVENT SHALL THE COPYRIGHT HOLDER OR CONTRIBUTORS BE LIABLE FOR
 #	ANY DIRECT, INDIRECT, INCIDENTAL, SPECIAL, EXEMPLARY, OR CONSEQUENTIAL DAMAGES
 #	(INCLUDING, BUT NOT LIMITED TO, PROCUREMENT OF SUBSTITUTE GOODS OR SERVICES;
 #	LOSS OF USE, DATA, OR PROFITS; OR BUSINESS INTERRUPTION) HOWEVER CAUSED AND ON
 #	ANY THEORY OF LIABILITY, WHETHER IN CONTRACT, STRICT LIABILITY, OR TORT
 #	(INCLUDING NEGLIGENCE OR OTHERWISE) ARISING IN ANY WAY OUT OF THE USE OF THIS
 #	SOFTWARE, EVEN IF ADVISED OF THE POSSIBILITY OF SUCH DAMAGE.
 #	--------------------------------------------------------------------------------
```

### 4. Burmese Word Break Dictionary Data (burmesedict.txt)

```
 #	Copyright (c) 2014 International Business Machines Corporation
 #	and others. All Rights Reserved.
 #
 #	This list is part of a project hosted at:
 #	  github.com/kanyawtech/myanmar-karen-word-lists
 #
 #	--------------------------------------------------------------------------------
 #	Copyright (c) 2013, LeRoy Benjamin Sharon
 #	All rights reserved.
 #
 #	Redistribution and use in source and binary forms, with or without modification,
 #	are permitted provided that the following conditions are met:
 #
 #	  Redistributions of source code must retain the above copyright notice, this
 #	  list of conditions and the following disclaimer.
 #
 #	  Redistributions in binary form must reproduce the above copyright notice, this
 #	  list of conditions and the following disclaimer in the documentation and/or
 #	  other materials provided with the distribution.
 #
 #	  Neither the name Myanmar Karen Word Lists, nor the names of its
 #	  contributors may be used to endorse or promote products derived from
 #	  this software without specific prior written permission.
 #
 #	THIS SOFTWARE IS PROVIDED BY THE COPYRIGHT HOLDERS AND CONTRIBUTORS "AS IS" AND
 #	ANY EXPRESS OR IMPLIED WARRANTIES, INCLUDING, BUT NOT LIMITED TO, THE IMPLIED
 #	WARRANTIES OF MERCHANTABILITY AND FITNESS FOR A PARTICULAR PURPOSE ARE
 #	DISCLAIMED. IN NO EVENT SHALL THE COPYRIGHT HOLDER OR CONTRIBUTORS BE LIABLE FOR
 #	ANY DIRECT, INDIRECT, INCIDENTAL, SPECIAL, EXEMPLARY, OR CONSEQUENTIAL DAMAGES
 #	(INCLUDING, BUT NOT LIMITED TO, PROCUREMENT OF SUBSTITUTE GOODS OR SERVICES;
 #	LOSS OF USE, DATA, OR PROFITS; OR BUSINESS INTERRUPTION) HOWEVER CAUSED AND ON
 #	ANY THEORY OF LIABILITY, WHETHER IN CONTRACT, STRICT LIABILITY, OR TORT
 #	(INCLUDING NEGLIGENCE OR OTHERWISE) ARISING IN ANY WAY OUT OF THE USE OF THIS
 #	SOFTWARE, EVEN IF ADVISED OF THE POSSIBILITY OF SUCH DAMAGE.
 #	--------------------------------------------------------------------------------
```

### 5. Time Zone Database

ICU uses the public domain data and code derived from 
Time Zone Database for its time zone support. The ownership of the TZ database is explained
in BCP 175: Procedure for Maintaining the Time Zone
Database section 7.

```
7.  Database Ownership

   The TZ database itself is not an IETF Contribution or an IETF
   document.  Rather it is a pre-existing and regularly updated work
   that is in the public domain, and is intended to remain in the public
   domain.  Therefore, BCPs 78 [RFC5378] and 79 [RFC3979] do not apply
   to the TZ Database or contributions that individuals make to it.
   Should any claims be made and substantiated against the TZ Database,
   the organization that is providing the IANA Considerations defined in
   this RFC, under the memorandum of understanding with the IETF,
   currently ICANN, may act in accordance with all competent court
   orders.  No ownership claims will be made by ICANN or the IETF Trust
   on the database or the code.  Any person making a contribution to the
   database or code waives all rights to future claims in that
   contribution or in the TZ Database.
```
