# Supplementary material for: Comparing Kaolin and Pinolene to Improve Sustainable Grapevine Production during Drought
Source: PLoS One. 2016 Jun 13;11(6):e0156631. doi: 10.1371/journal.pone.0156631 (PMC4905681; doi:10.1371/journal.pone.0156631)
Supplement: S1 File — (GZ) [file pone.0156631.s003.gz › PONE_Brillante_et_al_2016_Code_and_Data/packrat/src/stringr/stringr_1.0.0.tar.gz!/stringr/inst/doc/stringr.html]

Introduction to stringr


# Introduction to stringr

#### *2015-04-29*

Strings are not glamorous, high-profile components of R, but they do play a big role in many data cleaning and preparations tasks. R provides a solid set of string operations, but because they have grown organically over time, they can be inconsistent and a little hard to learn. Additionally, they lag behind the string operations in other programming languages, so that some things that are easy to do in languages like Ruby or Python are rather hard to do in R. The **stringr** package aims to remedy these problems by providing a clean, modern interface to common string operations.

More concretely, stringr:

- Simplifies string operations by eliminating options that you don’t need 95% of the time (the other 5% of the time you can functions from base R or stringi).
- Uses consistent function names and arguments.
- Produces outputs than can easily be used as inputs. This includes ensuring that missing inputs result in missing outputs, and zero length inputs result in zero length outputs. It also processes factors and character vectors in the same way.
- Completes R’s string handling functions with useful functions from other programming languages.

To meet these goals, stringr provides two basic families of functions:

- basic string operations, and
- pattern matching functions which use regular expressions to detect, locate, match, replace, extract, and split strings.

As of version 1.0, stringr is a thin wrapper around stringi, which implements all the functions in stringr with efficient C code based on the ICU library. Compared to stringi, stringr is considerably simpler: it provides fewer options and fewer functions. This is great when you’re getting started learning string functions, and if you do need more of stringi’s power, you should find the interface similar.

These are described in more detail in the following sections.

## Basic string operations

There are three string functions that are closely related to their base R equivalents, but with a few enhancements:

- `str_c()` is equivalent to `paste()`, but it uses the empty string (“”) as the default separator and silently removes `NULL` inputs.
- `str_length()` is equivalent to `nchar()`, but it preserves NA’s (rather than giving them length 2) and converts factors to characters (not integers).
- `str_sub()` is equivalent to `substr()` but it returns a zero length vector if any of its inputs are zero length, and otherwise expands each argument to match the longest. It also accepts negative positions, which are calculated from the left of the last character. The end position defaults to `-1`, which corresponds to the last character.
- `str_str<-` is equivalent to `substr<-`, but like `str_sub` it understands negative indices, and replacement strings not do need to be the same length as the string they are replacing.

Three functions add new functionality:

- `str_dup()` to duplicate the characters within a string.
- `str_trim()` to remove leading and trailing whitespace.
- `str_pad()` to pad a string with extra whitespace on the left, right, or both sides.

## Pattern matching

stringr provides pattern matching functions to **detect**, **locate**, **extract**, **match**, **replace**, and **split** strings. I’ll illustrate how they work with some strings and a regular expression designed to match (US) phone numbers:

```
strings <- c(
  "apple", 
  "219 733 8965", 
  "329-293-8753", 
  "Work: 579-499-7527; Home: 543.355.3679"
)
phone <- "([2-9][0-9]{2})[- .]([0-9]{3})[- .]([0-9]{4})"
```

- `str_detect()` detects the presence or absence of a pattern and returns a logical vector (similar to `grepl()`). `str_subset()` returns the elements of a character vector that match a regular expression (similar to `grep()` with `value = TRUE`)`.

  ```
  # Which strings contain phone numbers?
  str_detect(strings, phone)
  #> [1] FALSE  TRUE  TRUE  TRUE
  str_subset(strings, phone)
  #> [1] "219 733 8965"                          
  #> [2] "329-293-8753"                          
  #> [3] "Work: 579-499-7527; Home: 543.355.3679"
  ```
- `str_locate()` locates the first position of a pattern and returns a numeric matrix with columns start and end. `str_locate_all()` locates all matches, returning a list of numeric matrices. Similar to `regexpr()` and `gregexpr()`.

  ```
  # Where in the string is the phone number located?
  (loc <- str_locate(strings, phone))
  #>      start end
  #> [1,]    NA  NA
  #> [2,]     1  12
  #> [3,]     1  12
  #> [4,]     7  18
  str_locate_all(strings, phone)
  #> [[1]]
  #>      start end
  #> 
  #> [[2]]
  #>      start end
  #> [1,]     1  12
  #> 
  #> [[3]]
  #>      start end
  #> [1,]     1  12
  #> 
  #> [[4]]
  #>      start end
  #> [1,]     7  18
  #> [2,]    27  38
  ```
- `str_extract()` extracts text corresponding to the first match, returning a character vector. `str_extract_all()` extracts all matches and returns a list of character vectors.

  ```
  # What are the phone numbers?
  str_extract(strings, phone)
  #> [1] NA             "219 733 8965" "329-293-8753" "579-499-7527"
  str_extract_all(strings, phone)
  #> [[1]]
  #> character(0)
  #> 
  #> [[2]]
  #> [1] "219 733 8965"
  #> 
  #> [[3]]
  #> [1] "329-293-8753"
  #> 
  #> [[4]]
  #> [1] "579-499-7527" "543.355.3679"
  str_extract_all(strings, phone, simplify = TRUE)
  #>      [,1]           [,2]          
  #> [1,] ""             ""            
  #> [2,] "219 733 8965" ""            
  #> [3,] "329-293-8753" ""            
  #> [4,] "579-499-7527" "543.355.3679"
  ```
- `str_match()` extracts capture groups formed by `()` from the first match. It returns a character matrix with one column for the complete match and one column for each group. `str_match_all()` extracts capture groups from all matches and returns a list of character matrices. Similar to `regmatches()`.

  ```
  # Pull out the three components of the match
  str_match(strings, phone)
  #>      [,1]           [,2]  [,3]  [,4]  
  #> [1,] NA             NA    NA    NA    
  #> [2,] "219 733 8965" "219" "733" "8965"
  #> [3,] "329-293-8753" "329" "293" "8753"
  #> [4,] "579-499-7527" "579" "499" "7527"
  str_match_all(strings, phone)
  #> [[1]]
  #>      [,1] [,2] [,3] [,4]
  #> 
  #> [[2]]
  #>      [,1]           [,2]  [,3]  [,4]  
  #> [1,] "219 733 8965" "219" "733" "8965"
  #> 
  #> [[3]]
  #>      [,1]           [,2]  [,3]  [,4]  
  #> [1,] "329-293-8753" "329" "293" "8753"
  #> 
  #> [[4]]
  #>      [,1]           [,2]  [,3]  [,4]  
  #> [1,] "579-499-7527" "579" "499" "7527"
  #> [2,] "543.355.3679" "543" "355" "3679"
  ```
- `str_replace()` replaces the first matched pattern and returns a character vector. `str_replace_all()` replaces all matches. Similar to `sub()` and `gsub()`.

  ```
  str_replace(strings, phone, "XXX-XXX-XXXX")
  #> [1] "apple"                                 
  #> [2] "XXX-XXX-XXXX"                          
  #> [3] "XXX-XXX-XXXX"                          
  #> [4] "Work: XXX-XXX-XXXX; Home: 543.355.3679"
  str_replace_all(strings, phone, "XXX-XXX-XXXX")
  #> [1] "apple"                                 
  #> [2] "XXX-XXX-XXXX"                          
  #> [3] "XXX-XXX-XXXX"                          
  #> [4] "Work: XXX-XXX-XXXX; Home: XXX-XXX-XXXX"
  ```
- `str_split_fixed()` splits the string into a fixed number of pieces based on a pattern and returns a character matrix. `str_split()` splits a string into a variable number of pieces and returns a list of character vectors.

### Arguments

Each pattern matching function has the same first two arguments, a character vector of `string`s to process and a single `pattern` (regular expression) to match. The replace functions have an additional argument specifying the replacement string, and the split functions have an argument to specify the number of pieces.

Unlike base string functions, stringr offers control over matching not through arguments, but through modifier functions, `regexp()`, `coll()` and `fixed()`. This is a deliberate choice made to simplify these functions. For example, while `grepl` has six arguments, `str_detect()` only has two.

### Regular expressions

To be able to use these functions effectively, you’ll need a good knowledge of regular expressions, which this vignette is not going to teach you. Some useful tools to get you started:

- A good reference sheet.
- A tool that allows you to interactively test what a regular expression will match.
- A tool to build a regular expression from an input string.

When writing regular expressions, I strongly recommend generating a list of positive (pattern should match) and negative (pattern shouldn’t match) test cases to ensure that you are matching the correct components.

### Functions that return lists

Many of the functions return a list of vectors or matrices. To work with each element of the list there are two strategies: iterate through a common set of indices, or use `Map()` to iterate through the vectors simultaneously. The second strategy is illustrated below:

```
col2hex <- function(col) {
  rgb <- col2rgb(col)
  rgb(rgb["red", ], rgb["green", ], rgb["blue", ], max = 255)
}

# Goal replace colour names in a string with their hex equivalent
strings <- c("Roses are red, violets are blue", "My favourite colour is green")

colours <- str_c("\\b", colors(), "\\b", collapse="|")
# This gets us the colours, but we have no way of replacing them
str_extract_all(strings, colours)
#> [[1]]
#> [1] "red"  "blue"
#> 
#> [[2]]
#> [1] "green"

# Instead, let's work with locations
locs <- str_locate_all(strings, colours)
Map(function(string, loc) {
  hex <- col2hex(str_sub(string, loc))
  str_sub(string, loc) <- hex
  string
}, strings, locs)
#> $`Roses are red, violets are blue`
#> [1] "Roses are #FF0000, violets are blue"
#> [2] "Roses are red, violets are #0000FF" 
#> 
#> $`My favourite colour is green`
#> [1] "My favourite colour is #00FF00"
```

Another approach is to use the second form of `str_replace_all()`: if you give it a named vector, it applies each `pattern = replacement` in turn:

```
matches <- col2hex(colors())
names(matches) <- str_c("\\b", colors(), "\\b")

str_replace_all(strings, matches)
#> [1] "Roses are #FF0000, violets are #0000FF"
#> [2] "My favourite colour is #00FF00"
```

## Conclusion

stringr provides an opinionated interface to strings in R. It makes string processing simpler by removing uncommon options, and by vigorously enforcing consistency across functions. I have also added new functions that I have found useful from Ruby, and over time, I hope users will suggest useful functions from other programming languages. I will continue to build on the included test suite to ensure that the package behaves as expected and remains bug free.
