# Supplementary material for: Comparing Kaolin and Pinolene to Improve Sustainable Grapevine Production during Drought
Source: PLoS One. 2016 Jun 13;11(6):e0156631. doi: 10.1371/journal.pone.0156631 (PMC4905681; doi:10.1371/journal.pone.0156631)
Supplement: S1 File — (GZ) [file pone.0156631.s003.gz › PONE_Brillante_et_al_2016_Code_and_Data/packrat/src/zoo/zoo_1.7-12.tar.gz!/zoo/inst/doc/zoo-quickref.pdf]

zoo Quick Reference

Ajay Shah
National Institute of Public Finance and Policy, India

Achim Zeileis
Universität Innsbruck

Gabor Grothendieck
GKX Associates Inc.

Abstract

This vignette gives a brief overview of (some of) the functionality contained in **zoo** including several nifty code snippets when dealing with (daily) financial data. For a more complete overview of the package's functionality and extensibility see Zeileis and Grothendieck (2005) (contained as vignette "zoo" in the package), the manual pages and the reference card.

Keywords: irregular time series, daily data, weekly data, returns.

Read a series from a text file

To read in data in a text file, `read.table()` and associated functions can be used as usual with `zoo()` being called subsequently. The convenience function `read.zoo` is a simple wrapper to these functions that assumes the index is in the first column of the file and the remaining columns are data.

Data in `demo1.txt`, where each row looks like

```
23 Feb 2005|43.72
```

can be read in via

```
R> Sys.setlocale("LC_TIME", "C")

[1] "C"

R> inrusd <- read.zoo("demo1.txt", sep = "|", format="%d %b %Y")
```

The `format` argument causes the first column to be transformed to an index of class `"Date"`. The `Sys.setlocale` is set to `"C"` here to assure that the English month abbreviations are read correctly. (It is unnecessary if the system is already set to a locale that understands English month abbreviations.)

The data in `demo2.txt` look like

```
Daily,24 Feb 2005,2055.30,4337.00
```

and requires more attention because of the format of the first column.

```
R> tmp <- read.table("demo2.txt", sep = ",")
R> z <- zoo(tmp[, 3:4], as.Date(as.character(tmp[, 2]), format="%d %b %Y"))
R> colnames(z) <- c("Nifty", "Junior")
```

Query dates

To return all dates corresponding to a series `index(z)` or equivalently

```
R> time(z)
```

```
 [1] "2005-02-10" "2005-02-11" "2005-02-14" "2005-02-15" "2005-02-17"
 [6] "2005-02-18" "2005-02-21" "2005-02-22" "2005-02-23" "2005-02-24"
[11] "2005-02-25" "2005-02-28" "2005-03-01" "2005-03-02" "2005-03-03"
[16] "2005-03-04" "2005-03-07" "2005-03-08" "2005-03-09" "2005-03-10"
```

can be used. The first and last date can be obtained by

```
R> start(z)
```

```
[1] "2005-02-10"
```

```
R> end(inrusd)
```

```
[1] "2005-03-10"
```

Convert back into a plain matrix

To strip off the dates and just return a plain vector/matrix `coredata` can be used

```
R> plain <- coredata(z)
R> str(plain)
```

```
 num [1:20, 1:2] 2063 2082 2098 2090 2062 ...
 - attr(*, "dimnames")=List of 2
  ..$ : NULL
  ..$ : chr [1:2] "Nifty" "Junior"
```

Union and intersection

Unions and intersections of series can be computed by `merge`. The intersection are those days where both series have time points:

```
R> m <- merge(inrusd, z, all = FALSE)
```

whereas the union uses all dates and fills the gaps where one series has a time point but the other does not with `NA`s (by default):

```
R> m <- merge(inrusd, z)
```

`cbind(inrusd, z)` is almost equivalent to the `merge` call, but may lead to inferior naming in some situations hence `merge` is preferred

To combine a series with its lag, use

```
R> merge(inrusd, lag(inrusd, -1))
```

```
           inrusd lag(inrusd, -1)
2005-02-10  43.78              NA
2005-02-11  43.79           43.78
2005-02-14  43.72           43.79
2005-02-15  43.76           43.72
2005-02-16  43.82           43.76
2005-02-17  43.74           43.82
2005-02-18  43.84           43.74
2005-02-21  43.82           43.84
2005-02-22  43.72           43.82
2005-02-23  43.72           43.72
2005-02-24  43.70           43.72
2005-02-25  43.69           43.70
2005-02-28  43.64           43.69
2005-03-01  43.72           43.64
2005-03-02  43.70           43.72
2005-03-03  43.65           43.70
2005-03-04  43.71           43.65
2005-03-07  43.69           43.71
2005-03-09  43.67           43.69
2005-03-10  43.58           43.67
```

Visualization

By default, the `plot` method generates a graph for each series in `m`

```
R> plot(m)
```

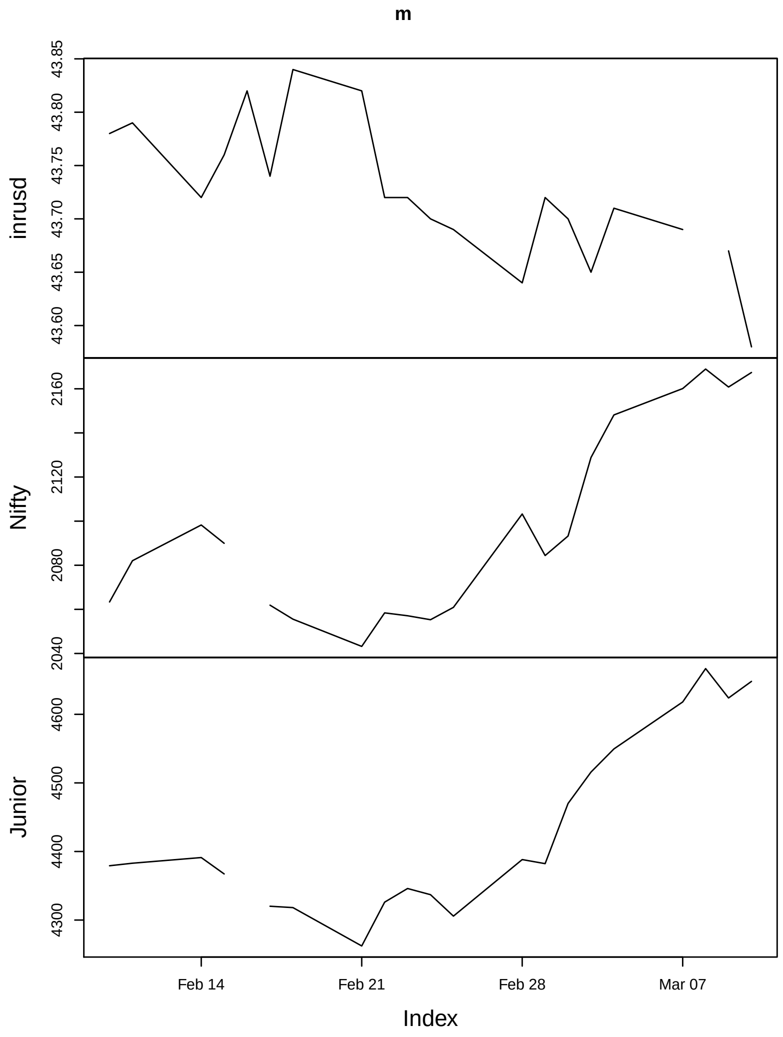

but several series can also be plotted in a single window.

```
R> plot(m[, 2:3], plot.type = "single", col = c("red", "blue"), lwd = 2)
```

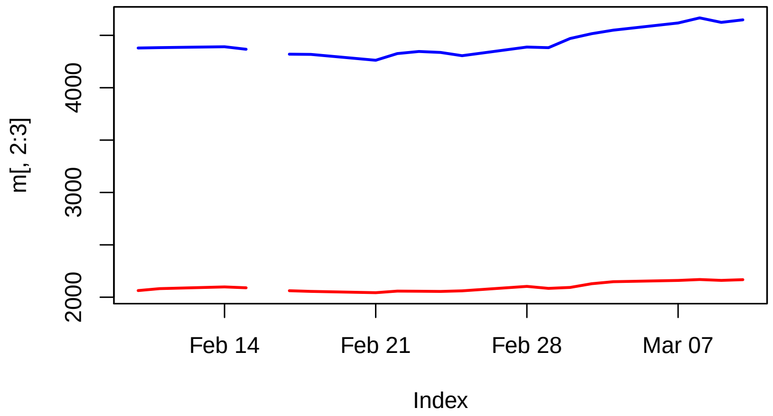

Select (a few) observations

Selections can be made for a range of dates of interest

```
R> window(z, start = as.Date("2005-02-15"), end = as.Date("2005-02-28"))

             Nifty  Junior
2005-02-15 2089.95 4367.25
2005-02-17 2061.90 4320.15
2005-02-18 2055.55 4318.15
2005-02-21 2043.20 4262.25
2005-02-22 2058.40 4326.10
2005-02-23 2057.10 4346.00
2005-02-24 2055.30 4337.00
2005-02-25 2060.90 4305.75
2005-02-28 2103.25 4388.20
```

and also just for a single date

```
R> m[as.Date("2005-03-10")]

           inrusd  Nifty  Junior
2005-03-10  43.58 2167.4 4648.05
```

Handle missing data

Various methods for dealing with `NA`s are available, including linear interpolation

```
R> interpolated <- na.approx(m)
```

'last observation carried forward',

```
R> m <- na.locf(m)
R> m

           inrusd   Nifty  Junior
2005-02-10  43.78 2063.35 4379.20
2005-02-11  43.79 2082.05 4382.90
2005-02-14  43.72 2098.25 4391.15
2005-02-15  43.76 2089.95 4367.25
2005-02-16  43.82 2089.95 4367.25
2005-02-17  43.74 2061.90 4320.15
2005-02-18  43.84 2055.55 4318.15
2005-02-21  43.82 2043.20 4262.25
2005-02-22  43.72 2058.40 4326.10
2005-02-23  43.72 2057.10 4346.00
2005-02-24  43.70 2055.30 4337.00
2005-02-25  43.69 2060.90 4305.75
2005-02-28  43.64 2103.25 4388.20
2005-03-01  43.72 2084.40 4382.25
2005-03-02  43.70 2093.25 4470.00
2005-03-03  43.65 2128.85 4515.80
2005-03-04  43.71 2148.15 4549.55
2005-03-07  43.69 2160.10 4618.05
2005-03-08  43.69 2168.95 4666.70
2005-03-09  43.67 2160.80 4623.85
2005-03-10  43.58 2167.40 4648.05
```

and others.

Prices and returns

To compute log-difference returns in %, the following convenience function is defined

```
R> prices2returns <- function(x) 100*diff(log(x))
```

which can be used to convert all columns (of prices) into returns.

```
R> r <- prices2returns(m)
```

A 10-day rolling window standard deviations (for all columns) can be computed by

```
R> rollapply(r, 10, sd)

               inrusd     Nifty    Junior
2005-02-17 0.14599121 0.6993355 0.7878843
2005-02-18 0.14527421 0.6300543 0.8083622
2005-02-21 0.14115862 0.8949318 1.0412806
2005-02-22 0.15166883 0.9345299 1.0256508
2005-02-23 0.14285470 0.9454103 1.1957959
```

```
2005-02-24 0.13607992 0.9453855 1.1210963
2005-02-25 0.11962991 0.9334899 1.1105966
2005-02-28 0.11963193 0.8585071 0.9388661
2005-03-01 0.09716262 0.8569891 0.9131822
2005-03-02 0.09787943 0.8860388 1.0566389
2005-03-03 0.11568119 0.8659890 1.0176645
```

To go from a daily series to the series of just the last-traded-day of each month `aggregate` can be used

```
R> prices2returns(aggregate(m, as.yearmon, tail, 1))

             inrusd    Nifty   Junior
Mar 2005 -0.1375831 3.004453 5.752866
```

Analogously, the series can be aggregated to the last-traded-day of each week employing a convenience function `nextfri` that computes for each `"Date"` the next friday.

```
R> nextfri <- function(x) 7 * ceiling(as.numeric(x-5+4) / 7) + as.Date(5-4)
R> prices2returns(aggregate(na.locf(m), nextfri, tail, 1))

                inrusd      Nifty     Junior
2005-02-18  0.11411618 -1.2809533 -1.4883536
2005-02-25 -0.34273997  0.2599329 -0.2875731
2005-03-04  0.04576659  4.1464226  5.5076988
2005-03-11 -0.29785794  0.8921286  2.1419450
```

Here is a similar example of `aggregate` where we define `to4sec` analogously to `nextfri` in order to aggregate the `zoo` object `zsec` every 4 seconds.

```
R> zsec <- structure(1:10, index = structure(c(1234760403.968, 1234760403.969,
+ 1234760403.969, 1234760405.029, 1234760405.029, 1234760405.03,
+ 1234760405.03, 1234760405.072, 1234760405.073, 1234760405.073
+ ), class = c("POSIXt", "POSIXct"), tzone = ""), class = "zoo")
R> to4sec <- function(x) as.POSIXct(4*ceiling(as.numeric(x)/4), origin = "1970-01-01")
R> aggregate(zsec, to4sec, tail, 1)

2009-02-16 05:00:04 2009-02-16 05:00:08
                  3                  10
```

Here is another example using the same `zsec` zoo object but this time rather than aggregating we truncate times to the second using the last data value for each such second. For large objects this will be much faster than using `aggregate.zoo` .

```
R> # tmp is zsec with time discretized into one second bins
R> tmp <- zsec
R> st <- start(tmp)
```

```
R> Epoch <- st - as.numeric(st)
R> time(tmp) <- as.integer(time(tmp) + 1e-7) + Epoch
R> # find index of last value in each one second interval
R> ix <- !duplicated(time(tmp), fromLast = TRUE)
R> # merge with grid
R> merge(tmp[ix], zoo(, seq(start(tmp), end(tmp), "sec")))

2009-02-16 05:00:03 2009-02-16 05:00:04 2009-02-16 05:00:05
                  3                  NA                  10

R> # Here is a function which generalizes the above:
R>
R> intraday.discretise <- function(b, Nsec) {
+  st <- start(b)
+  time(b) <- Nsec * as.integer(time(b)+1e-7) %/% Nsec + st -
+  as.numeric(st)
+  ix <- !duplicated(time(b), fromLast = TRUE)
+  merge(b[ix], zoo(, seq(start(b), end(b), paste(Nsec, "sec"))))
+ }
R> intraday.discretise(zsec, 1)

2009-02-16 05:00:03 2009-02-16 05:00:04 2009-02-16 05:00:05
                  3                  NA                  10

R>
```

Query Yahoo! Finance

When connected to the internet, Yahoo! Finance can be easily queried using the `get.hist.quote` function in

```
R> library("tseries")
```

From version 0.9-30 on, `get.hist.quote` by default returns `"zoo"` series with a `"Date"` attribute (in previous versions these had to be transformed from `"ts"` 'by hand').
A daily series can be obtained by:

```
R> sunw <- get.hist.quote(instrument = "SUNW", start = "2004-01-01", end = "2004-12-31")
```

A monthly series can be obtained and transformed by

```
R> sunw2 <- get.hist.quote(instrument = "SUNW", start = "2004-01-01", end = "2004-12-31",
+   compression = "m", quote = "Close")
```

Here, `"yearmon"` dates might be even more useful:

```
R> time(sunw2) <- as.yearmon(time(sunw2))
```

The same series can equivalently be computed from the daily series via

```
R> sunw3 <- aggregate(sunw[, "Close"], as.yearmon, tail, 1)
```

The corresponding returns can be computed via

```
R> r <- prices2returns(sunw3)
```

where `r` is still a `"zoo"` series.

Query Oanda

Similarly you can obtain historical exchange rates from http://www.oanda.com/ using `get.hist.quote`. A daily series of EUR/USD exchange rates can be queried by

```
R> eur.usd <- get.hist.quote(instrument = "EUR/USD", provider = "oanda", start = "2004-01-
```

This contains the exchange rates for every day in 2004. However, it is common practice in many situations to exclude the observations from weekends. To do so, we write a little convenience function which can determine for a vector of `"Date"` observations whether it is a weekend or not

```
R> is.weekend <- function(x) ((as.numeric(x)-2) %% 7) < 2
```

Based on this we can omit all observations from weekends

```
R> eur.usd <- eur.usd[!is.weekend(time(eur.usd))]
```

The function `is.weekend` introduced above exploits the fact that a `"Date"` is essentially the number of days since 1970-01-01, a Thursday. A more intelligible function which yields identical results could be based on the `"POSIXlt"` class

```
R> is.weekend <- function(x) {
+   x <- as.POSIXlt(x)
+   x$wday > 5 | x$wday < 1
+ }
```

Summaries

Here we create a daily series and then find the series of quarterly means and standard deviations and also for weekly means and standard deviations where we define weeks to end on Tuesay.

We do the above separately for mean and standard deviation, binding the two results together and then show a different approach in which we define a custom `ag` function that can accept multiple function names as a vector argument.

```
R> date1 <- seq(as.Date("2001-01-01"), as.Date("2002-12-1"), by = "day")
R> len1 <- length(date1)
R> set.seed(1) # to make it reproducible
R> data1 <- zoo(rnorm(len1), date1)
R> # quarterly summary
R>
R> data1q.mean <- aggregate(data1, as.yearqtr, mean)
R> data1q.sd <- aggregate(data1, as.yearqtr, sd)
R> head(cbind(mean = data1q.mean, sd = data1q.sd), main = "Quarterly")

                mean        sd
2001 Q1  0.108503596 0.8861821
2001 Q2 -0.006836172 0.9800027
2001 Q3 -0.042260559 0.9954100
2001 Q4  0.096188411 1.0336234
2002 Q1 -0.092684201 1.0337850
2002 Q2  0.049217429 1.0860119

R> # weekly summary - week ends on tuesday
R>
R> # Given a date find the next Tuesday.
R> # Based on formula in Prices and Returns section.
R> nexttue <- function(x) 7 * ceiling(as.numeric(x - 2 + 4)/7) + as.Date(2 - 4)
R> data1w <- cbind(
+         mean = aggregate(data1, nexttue, mean),
+         sd = aggregate(data1, nexttue, sd)
+ )
R> head(data1w)

                  mean        sd
2001-01-02 -0.22140524 0.5728252
2001-01-09  0.29574667 0.8686111
2001-01-16 -0.02281531 1.2305420
2001-01-23  0.58834959 0.3993624
2001-01-30 -0.44463015 0.9619139
2001-02-06 -0.08522653 0.8349544

R> ### ALTERNATIVE ###
R>
R> # Create function ag like aggregate but takes vector of
R> # function names.
R>
R> FUNs <- c(mean, sd)
R> ag <- function(z, by, FUNs) {
+         f <- function(f) aggregate(z, by, f)
+         do.call(cbind, sapply(FUNs, f, simplify = FALSE))
+ }
```

```
R> data1q <- ag(data1, as.yearqtr, c("mean", "sd"))
R> data1w <- ag(data1, nexttue, c("mean", "sd"))
R> head(data1q)
```

```
                mean        sd
2001 Q1  0.108503596 0.8861821
2001 Q2 -0.006836172 0.9800027
2001 Q3 -0.042260559 0.9954100
2001 Q4  0.096188411 1.0336234
2002 Q1 -0.092684201 1.0337850
2002 Q2  0.049217429 1.0860119
```

```
R> head(data1w)
```

```
                  mean        sd
2001-01-02 -0.22140524 0.5728252
2001-01-09  0.29574667 0.8686111
2001-01-16 -0.02281531 1.2305420
2001-01-23  0.58834959 0.3993624
2001-01-30 -0.44463015 0.9619139
2001-02-06 -0.08522653 0.8349544
```

References

Zeileis A, Grothendieck G (2005). "**zoo**: S3 Infrastructure for Regular and Irregular Time Series." *Journal of Statistical Software*, **14**(6), 1–27. URL http://www.jstatsoft.org/v14/i06/.

Affiliation:

Ajay Shah
National Institute of Public Finance and Policy, India
E-mail: ajayshah@mayin.org

Achim Zeileis
Universität Innsbruck
E-mail: Achim.Zeileis@R-project.org

Gabor Grothendieck
GKX Associates Inc.
E-mail: ggrothendieck@gmail.com
