# Supplementary material for: Comparing Kaolin and Pinolene to Improve Sustainable Grapevine Production during Drought
Source: PLoS One. 2016 Jun 13;11(6):e0156631. doi: 10.1371/journal.pone.0156631 (PMC4905681; doi:10.1371/journal.pone.0156631)
Supplement: S1 File — (GZ) [file pone.0156631.s003.gz › PONE_Brillante_et_al_2016_Code_and_Data/packrat/src/zoo/zoo_1.7-12.tar.gz!/zoo/inst/doc/zoo-faq.pdf]

zoo FAQ

zoo Development Team

Abstract

This is a collection of frequently asked questions (FAQ) about the **zoo** package together with their answers.

Keywords: irregular time series, ordered observations, time index, daily data, weekly data, returns.

1. I know that duplicate times are not allowed but my data has them. What do I do?

zoo objects should not normally contain duplicate times. If you try to create such an object using **zoo** or `read.zoo` then warnings will be issued but the objects will be created. The user then has the opportunity to fix them up – typically by using `aggregate.zoo` or `duplicated`.

Merging is not well defined for duplicate series with duplicate times and rather than give an undesired or unexpected result, `merge.zoo` issues an error message if it encounters such illegal objects. Since `merge.zoo` is the workhorse behind many **zoo** functions, a significant portion of **zoo** will not accept duplicates among the times.

Typically duplicates are eliminated by (1) averaging over them, (2) taking the last among each run of duplicates or (3) interpolating the duplicates and deleting ones on the end that cannot be interpolated. These three approaches are shown here using the `aggregate.zoo` function. Another way to do this is to use the `aggregate` argument of `read.zoo` which will aggregate the zoo object read in by `read.zoo` all in one step.

Note that in the example code below that `identity` is the identity function (i.e. it just returns its argument). It is an R core function:

A `"zoo"` series with duplicated indexes

```
> z <- suppressWarnings(zoo(1:8, c(1, 2, 2, 2, 3, 4, 5, 5)))
> z

1 2 2 2 3 4 5 5
1 2 3 4 5 6 7 8
```

Fix it up by averaging duplicates:

```
> aggregate(z, identity, mean)

  1   2   3   4   5
1.0 3.0 5.0 6.0 7.5
```

Or, fix it up by taking last in each set of duplicates:

```
> aggregate(z, identity, tail, 1)
```

```
1 2 3 4 5
1 4 5 6 8
```

Fix it up via interpolation of duplicate times

```
> time(z) <- na.approx(ifelse(duplicated(time(z)), NA, time(z)), na.rm = FALSE)
```

If there is a run of equal times at end they wind up as `NA`s and we cannot have `NA` times.

```
> z[!is.na(time(z))]
```

```
     1      2 2.3333 2.6667      3      4      5 
     1      2      3      4      5      6      7 
```

The `read.zoo` command has an `aggregate` argument that supports arbitrary summarization. For example, in the following we take the last value among any duplicate times and sum the volumes among all duplicate times. We do this by reading the data twice, once for each aggregate function. In this example, the first three columns are junk that we wish to suppress which is why we specified `colClasses`; however, in most cases that argument would not be necessary.

```
> Lines <- "1|BHARTIARTL|EQ|18:15:05|600|1
+ 2|BHARTIARTL|EQ|18:15:05|600|99
+ 3|GLENMARK|EQ|18:15:05|238.1|5
+ 4|HINDALCO|EQ|18:15:05|43.75|100
+ 5|BHARTIARTL|EQ|18:15:05|600|1
+ 6|BHEL|EQ|18:15:05|1100|11
+ 7|HINDALCO|EQ|18:15:06|43.2|1
+ 8|CHAMBLFERT|EQ|18:15:06|46|10
+ 9|CHAMBLFERT|EQ|18:15:06|46|90
+ 10|BAJAUTOFIN|EQ|18:15:06|80|100"
> library("zoo")
> library("chron")
> tail1 <- function(x) tail(x, 1)
> cls <- c("NULL", "NULL", "NULL", "character", "numeric", "numeric")
> nms <- c("", "", "", "time", "value", "volume")
> z <- read.zoo(textConnection(Lines), aggregate = tail1,
+   FUN = times, sep = "|", colClasses = cls, col.names = nms)
> z2 <- read.zoo(textConnection(Lines), aggregate = sum,
+   FUN = times, sep = "|", colClasses = cls, col.names = nms)
> z$volume <- z2$volume
> z
```

```
         value volume
18:15:05  1100    217
18:15:06    80    201
```

If the reason for the duplicate times is that the data is stored in long format then use `read.zoo` (particlarly the `split` argument) to convert it to wide format. Wide format is typically a time series whereas long format is not so wide format is the suitable one for zoo.

```
> Lines <- "Date Stock Price
+ 2000-01-01 IBM 10
+ 2000-01-02 IBM 11
+ 2000-01-01 ORCL 12
+ 2000-01-02 ORCL 13"
> stocks <- read.zoo(textConnection(Lines), header = TRUE, split = "Stock")
> stocks
```

```
           IBM ORCL
2000-01-01  10   12
2000-01-02  11   13
```

2. When I try to specify a log axis to `plot.zoo` *a warning is issued. What is wrong?*

Arguments that are part of `...` are passed to the `panel` function and the default `panel` function, `lines`, does not accept `log`. Either ignore the warning, use `suppressWarnings` (see `?suppressWarnings`) or create your own panel function which excludes the `log`:

```
> z <- zoo(1:100)
> plot(z, log = "y", panel = function(..., log) lines(...))
```

3. How do I create right and a left vertical axes in `plot.zoo`*?*

The following shows an example of creating a plot containing a single panel and both left and right axes.

```
> set.seed(1)
> z.Date <- as.Date(paste(2003, 02, c(1, 3, 7, 9, 14), sep = "-"))
> z <- zoo(cbind(left = rnorm(5), right = rnorm(5, sd = 0.2)), z.Date)
> plot(z[,1], xlab = "Time", ylab = "")
> opar <- par(usr = c(par("usr")[1:2], range(z[,2])))
> lines(z[,2], lty = 2)
> axis(side = 4)
> legend("bottomright", lty = 1:2, legend = colnames(z), bty="n")
> par(opar)
```

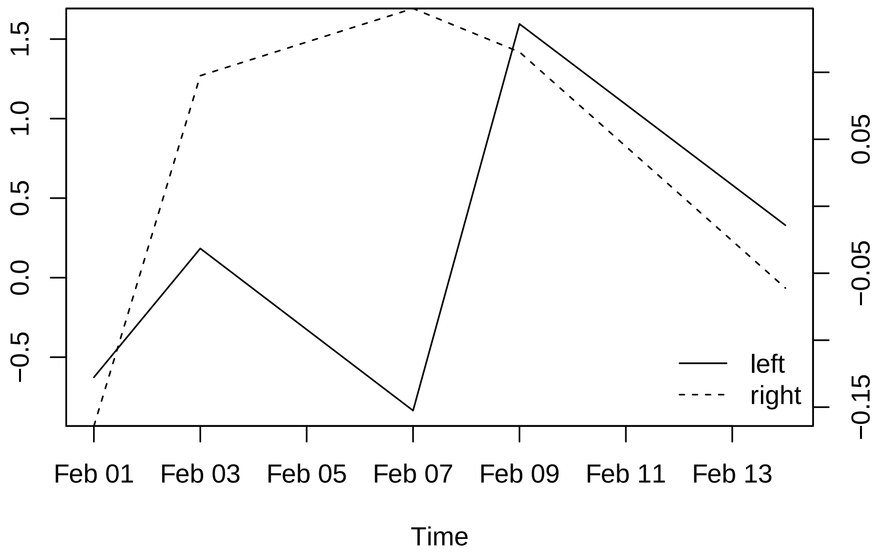

Figure 1: Left and right `plot.zoo` axes.

4. I have data frame with both numeric and factor columns. How do I convert that to a `"zoo"` *object?*

A `"zoo"` object may be (1) a numeric vector, (2) a numeric matrix or (3) a factor but may not contain both a numeric vector and factor. The underlying reason for this constraint is that `"zoo"` was intended to generalize R's `"ts"` class, which is also based on matrices, to irregularly spaced series with an arbitrary index class. The main reason to stick to matrices is that operations on matrices in R are much faster than on data frames.

If you have a data frame with both numeric and factor variables that you want to convert to `"zoo"`, you can do one of the following.

Use two `"zoo"` variables instead:

```
> DF <- data.frame(time = 1:4, x = 1:4, f = factor(letters[c(1, 1, 2, 2)]))
> zx <- zoo(DF$x, DF$time)
> zf <- zoo(DF$f, DF$time)
```

These could also be held in a `"data.frame"` again:

```
> DF2 <- data.frame(x = zx, f = zf)
```

Or convert the factor to numeric and create a single `"zoo"` series:

```
> z <- zoo(data.matrix(DF[-1]), DF$time)
```

5. Why does lag give slightly different results on a "zoo" and a "zooreg" series which are otherwise the same?

To be definite let us consider the following examples, noting how both `lag` and `diff` give a different answer with the same input except its class is `"zoo"` in one case and `"zooreg"` in another:

```
> z <- zoo(11:15, as.Date("2008-01-01") + c(-4, 1, 2, 3, 6))
> zr <- as.zooreg(z)
> lag(z)

2007-12-28 2008-01-02 2008-01-03 2008-01-04
        12         13         14         15

> lag(zr)

2007-12-27 2008-01-01 2008-01-02 2008-01-03 2008-01-06
        11         12         13         14         15

> diff(log(z))

2008-01-02 2008-01-03 2008-01-04 2008-01-07
0.08701138 0.08004271 0.07410797 0.06899287

> diff(log(zr))

2008-01-03 2008-01-04
0.08004271 0.07410797
```

`lag.zoo` and `lag.zooreg` work differently. For `"zoo"` objects the lagged version is obtained by moving values to the adjacent time point that exists in the series but for `"zooreg"` objects the time is lagged by `deltat`, the time between adjacent regular times.

A key implication is that `"zooreg"` can lag a point to a time point that did not previously exist in the series and, in particular, can lag a series outside of the original time range whereas that is not possible in a `"zoo"` series.

Note that `lag.zoo` has an `na.pad=` argument which in some cases may be what is being sought here.

The difference between `diff.zoo` and `diff.zooreg` stems from the fact that `diff(x)` is defined in terms of `lag` like this: `x-lag(x,-1)`.

6. How do I subtract the mean of each month from a "zoo" series?

Suppose we have a daily series. To subtract the mean of Jan 2007 from each day in that month, subtract the mean of Feb 2007 from each day in that month, etc. try this:

```
> set.seed(123)
> z <- zoo(rnorm(100), as.Date("2007-01-01") + seq(0, by = 10, length = 100))
> z.demean1 <- z - ave(z, as.yearmon(time(z)))
```

This first generates some artificial data and then employs `ave` to compute monthly means. To subtract the mean of all Januaries from each January, etc. try this:

```
> z.demean2 <- z - ave(z, format(time(z), "%m"))
```

7. How do I create a monthly series but still keep track of the dates?

Create a S3 subclass of `"yearmon"` called `"yearmon2"` that stores the dates as names on the time vector. It will be sufficient to create an `as.yearmon2` generic together with an `as.yearmon2.Date` methods as well as the inverse: `as.Date.yearmon2`.

```
> as.yearmon2 <- function(x, ...) UseMethod("as.yearmon2")
> as.yearmon2.Date <- function(x, ...) {
+   y <- as.yearmon(with(as.POSIXlt(x, tz = "GMT"), 1900 + year + mon/12))
+   names(y) <- x
+   structure(y, class = c("yearmon2", class(y)))
+ }
```

`as.Date.yearmon2` is inverse of `as.yearmon2.Date`

```
> as.Date.yearmon2 <- function(x, frac = 0, ...) {
+   if (!is.null(names(x))) return(as.Date(names(x)))
+   x <- unclass(x)
+   year <- floor(x + .001)
+   month <- floor(12 * (x - year) + 1 + .5 + .001)
+   dd.start <- as.Date(paste(year, month, 1, sep = "-"))
+   dd.end <- dd.start + 32 - as.numeric(format(dd.start + 32, "%d"))
+   as.Date((1-frac) * as.numeric(dd.start) + frac * as.numeric(dd.end),
+     origin = "1970-01-01")
+ }
```

This new class will act the same as `"yearmon"` stores and allows recovery of the dates using `as.Date` and `aggregate.zoo`.

```
> dd <- seq(as.Date("2000-01-01"), length = 5, by = 32)
> z <- zoo(1:5, as.yearmon2(dd))
> z

Jan 2000 Feb 2000 Mar 2000 Apr 2000 May 2000
       1        2        3        4        5

> aggregate(z, as.Date, identity)

2000-01-01 2000-02-02 2000-03-05 2000-04-06 2000-05-08
         1          2          3          4          5
```

8. How are axes added to a plot created using `plot.zoo`*?*

On single panel plots `axis` or `Axis` can be used just as with any classic graphics plot in R. The following example adds custom axis for single panel plot. It labels months but uses the larger year for January. Months, quarters and years should have successively larger ticks.

```
> z <- zoo(0:500, as.Date(0:500))
> plot(z, xaxt = "n")
> tt <- time(z)
> m <- unique(as.Date(as.yearmon(tt)))
> jan <- format(m, "%m") == "01"
> mlab <- substr(months(m[!jan]), 1, 1)
> axis(side = 1, at = m[!jan], labels = mlab, tcl = -0.3, cex.axis = 0.7)
> axis(side = 1, at = m[jan], labels = format(m[jan], "%y"), tcl = -0.7)
> axis(side = 1, at = unique(as.Date(as.yearqtr(tt))), labels = FALSE)
> abline(v = m, col = grey(0.8), lty = 2)
```

A multivariate series can either be generated as (1) multiple single panel plots:

```
> z3 <- cbind(z1 = z, z2 = 2*z, z3 = 3*z)
> opar <- par(mfrow = c(2, 2))
> tt <- time(z)
> m <- unique(as.Date(as.yearmon(tt)))
> jan <- format(m, "%m") == "01"
> mlab <- substr(months(m[!jan]), 1, 1)
> for(i in 1:ncol(z3)) {
+   plot(z3[,i], xaxt = "n", ylab = colnames(z3)[i], ylim = range(z3))
+   axis(side = 1, at = m[!jan], labels = mlab, tcl = -0.3, cex.axis = 0.7)
+   axis(side = 1, at = m[jan], labels = format(m[jan], "%y"), tcl = -0.7)
+   axis(side = 1, at = unique(as.Date(as.yearqtr(tt))), labels = FALSE)
+ }
> par(opar)
```

or (2) as a multipanel plot. In this case any custom axis must be placed in a panel function.

```
> plot(z3, screen = 1:3, xaxt = "n", nc = 2, ylim = range(z3),
+   panel = function(...) {
+     lines(...)
+     panel.number <- parent.frame()$panel.number
+     nser <- parent.frame()$nser
+     # place axis on bottom panel of each column only
+     if (panel.number %% 2 == 0 || panel.number == nser) {
+       tt <- list(...)[[1]]
+       m <- unique(as.Date(as.yearmon(tt)))
+       jan <- format(m, "%m") == "01"
+       mlab <- substr(months(m[!jan]), 1, 1)
+       axis(side = 1, at = m[!jan], labels = mlab, tcl = -0.3, cex.axis = 0.7)
```

```
+       axis(side = 1, at = m[jan], labels = format(m[jan], "%y"), tcl = -0.7)
+       axis(side = 1, at = unique(as.Date(as.yearqtr(tt))), labels = FALSE)
+     }
+ })
```

9. Why is nothing plotted except axes when I plot an object with many `NA`s?

Isolated points surrounded by `NA` values do not form lines:

```
> z <- zoo(c(1, NA, 2, NA, 3))
> plot(z)
```

So try one of the following:
Plot points rather than lines.

```
> plot(z, type = "p")
```

Omit `NA`s and plot that.

```
> plot(na.omit(z))
```

Fill in the `NA`s with interpolated values.

```
> plot(na.approx(z))
```

Plot points with lines superimposed.

```
> plot(z, type = "p")
> lines(na.omit(z))
```

Note that this is not specific to **zoo.** If we plot in R without **zoo** we get the same behavior.

10. Does zoo work with Rmetrics?

Yes. `timeDate` class objects from the **timeDate** package can be used directly as the index of a `zoo` series and `as.timeSeries.zoo` and `as.zoo.timeSeries` can convert back and forth between objects of class `zoo` and class `timeSeries` from the **timeSeries** package.

```
> library("timeDate")
> dts <- c("1989-09-28", "2001-01-15", "2004-08-30", "1990-02-09")
> tms <- c(  "23:12:55",   "10:34:02",   "08:30:00",   "11:18:23")
> td <- timeDate(paste(dts, tms), format = "%Y-%m-%d %H:%M:%S")
> library("zoo")
> z <- zoo(1:4, td)
> zz <- merge(z, lag(z))
> plot(zz)
> library("timeSeries")
> zz
```

```
                    z lag(z)
1989-09-28 23:12:55 1      4
1990-02-09 11:18:23 4      2
2001-01-15 10:34:02 2      3
2004-08-30 08:30:00 3     NA
```

```
> as.timeSeries(zz)
```

```
GMT
                    z lag(z)
1989-09-28 23:12:55 1      4
1990-02-09 11:18:23 4      2
2001-01-15 10:34:02 2      3
2004-08-30 08:30:00 3     NA
```

```
> as.zoo(as.timeSeries(zz))
```

```
                    z lag(z)
1989-09-28 23:12:55 1      4
1990-02-09 11:18:23 4      2
2001-01-15 10:34:02 2      3
2004-08-30 08:30:00 3     NA
```

11. What other packages use zoo?

A DEIS dependency means that a package lists **zoo** in the Depends, Enhances, Imports or Suggests clause of their DESCRIPTION file. As of September 27, 2011 there are 65 packages on CRAN with DEIS dependencies on zoo and 207 packages which either have direct DEIS dependencies or a DEIS dependency on a package which in turn has a DEIS dependency on zoo. This suggests that packages that have a DEIS dependency on zoo are themselves popular. If one recursively calculates DEIS dependencies to all depths then 2127 packages on CRAN have direct or indirect DEIS dependencies on zoo. That is over half of CRAN. Below are 74 packages which include those with direct DEIS dependencies as well as packages that are often used with zoo:

Some packages depend on zoo indirectly listing such a relationship to a package which in turn has such a dependency on zoo. There are 207 packages which There are 74 other CRAN packages that are or can be used with **zoo** (and possibly more in other repositories):

Depends	
**AER**	Applied Econometrics with R
**BootPR**	Bootstrap Prediction Intervals and Bias-Corrected Forecasting
**DMwR**	Functions and data for "Data Mining with R"
**FinTS**	Companion to Tsay (2005) Analysis of Financial Time Series
**MFDF**	Modeling Functional Data in Finance
**Modalclust**	Hierarchical Modal Clustering
**PerformanceAnalytics**	Econometric tools for performance and risk analysis
**RBloomberg**	R/Bloomberg
**RghcnV3**	Global Historical Climate Network Version 3
**StreamMetabolism**	Stream Metabolism-A package for calculating single station metabolism from diurnal Oxygen curves
**TSfame**	Time Series Database Interface extensions for fame
**TShistQuote**	Time Series Database Interface extensions for get.hist.quote
**TSxls**	Time Series Database Interface extension to connect to spreadsheets
**VhayuR**	Vhayu R Interface
**delftfews**	delftfews R extensions
**dyn**	Time Series Regression
**dynlm**	Dynamic Linear Regression
**fda**	Functional Data Analysis
**forecast**	Forecasting functions for time series
**fractalrock**	Generate fractal time series with non-normal returns distribution
**fxregime**	Exchange Rate Regime Analysis
**glogis**	Fitting and Testing Generalized Logistic Distributions
**hydroTSM**	Time series management, analysis and interpolation for hydrological modelling
**lmtest**	Testing Linear Regression Models
**meboot**	Maximum Entropy Bootstrap for Time Series
**mlogit**	multinomial logit model
**party**	A Laboratory for Recursive Partytioning
**quantmod**	Quantitative Financial Modelling Framework
**rdatamarket**	Data access API for DataMarket.com
**sandwich**	Robust Covariance Matrix Estimators
**sde**	Simulation and Inference for Stochastic Differential Equations
**solaR**	Solar Photovoltaic Systems
**spacetime**	classes and methods for spatio-temporal data
**strucchange**	Testing, Monitoring, and Dating Structural Changes
**tawny**	Provides various portfolio optimization strategies including random matrix theory and shrinkage estimators
**termstrc**	Zero-coupon Yield Curve Estimation
**tgram**	Functions to compute and plot tracheidograms
**tripEstimation**	Metropolis sampler and supporting functions for estimating animal movement from archival tags and satellite fixes
**tseries**	Time series analysis and computational finance
**wq**	Exploring water quality monitoring data
**xts**	eXtensible Time Series

Enhances	
**chron**	Chronological objects which can handle dates and times
**hydroTSM**	Time series management, analysis and interpolation for hydrological modelling
**lubridate**	Make dealing with dates a little easier
**tis**	Time Indexes and Time Indexed Series

Imports	
**fxregime**	Exchange Rate Regime Analysis
**glogis**	Fitting and Testing Generalized Logistic Distributions
**hydroGOF**	Goodness-of-fit functions for comparison of simulated and observed hydrological time series
**openair**	Tools for the analysis of air pollution data
**rasterVis**	Visualization methods for the raster package

Suggests	
**MeDiChI**	MeDiChI ChIP-chip deconvolution library
**RQuantLib**	R interface to the QuantLib library
**TSAgg**	Time series Aggregation
**TSMySQL**	Time Series Database Interface extensions for MySQL
**TSPostgreSQL**	Time Series Database Interface extensions for PostgreSQL
**TSSQLite**	Time Series Database Interface extentions for SQLite
**TSdbi**	Time Series Database Interface
**TSodbc**	Time Series Database Interface extensions for ODBC
**TSzip**	Time Series Database Interface extension to connect to zip files
**UsingR**	Data sets for the text ”Using R for Introductory Statistics”
**Zelig**	Everyone's Statistical Software
**gsubfn**	Utilities for strings and function arguments
**latticeExtra**	Extra Graphical Utilities Based on Lattice
**mondate**	Keep track of dates in terms of months
**playwith**	A GUI for interactive plots using GTK+
**pscl**	Political Science Computational Laboratory, Stanford University
**quantreg**	Quantile Regression
**tframePlus**	Time Frame coding kernel extensions

Uses or Used with	
**timeDate**	**Rmetrics** date and time functions: `timeDate` usable with `zoo`
**grid**	Graphics infrastructure: use with `xyplot.zoo`
**its**	Irregular time series: `as.its.zoo`, `as.zoo.its`
**lattice**	**grid**-based graphics: use with `xyplot.zoo`
**timeSeries**	**Rmetrics** time series functions: `as.timeSeries.zoo`, `as.zoo.timeSeries`
**YaleToolkit**	Data exploration tools from Yale University: accepts `"zoo"` input

12. Why does `ifelse` *not work as I expect?*

The ordinary R `ifelse` function only works with zoo objects if all three arguments are zoo

objects with the same time index. **zoo** provides an `ifelse.zoo` function that should be used instead. The `.zoo` part must be written out since `ifelse` is not generic.

```
> z <- zoo(c(1, 5, 10, 15))
> # wrong !!!
> ifelse(diff(z) > 4, -z, z)

  2   3   4
  1  -5 -10

> # ok
> ifelse.zoo(diff(z) > 4, -z, z)

  1   2   3   4
 NA   5 -10 -15

> # or if we merge first we can use ordinary ifelse
> xm <- merge(z, dif = diff(z))
> with(xm, ifelse(dif > 4, -z, z))

  1   2   3   4
 NA   5 -10 -15

> # or in this case we could also use orindary ifelse if we
> # use fill = NA to ensure all three have same index
> ifelse(diff(z, fill = NA) > 4, -z, z)

  2   3   4
  1  -5 -10
```

13. In a series which is regular except for a few missing times or for which we wish to align to a grid how is it filled or aligned?

```
> # April is missing
> zym <- zoo(1:5, as.yearmon("2000-01-01") + c(0, 1, 2, 4, 5)/12)
> g <- seq(start(zym), end(zym), by = 1/12)
> na.locf(zym, xout = g)

Jan 2000 Feb 2000 Mar 2000 Apr 2000 May 2000 Jun 2000
       1        2        3        3        4        5
```

A variation of this is where the grid is of a different date/time class than the original series. In that case use the `x` argument. In the example that follows the series `z` is of `"Date"` class whereas the grid is of `"yearmon"` class:

```
> z <- zoo(1:3, as.Date(c("2000-01-15", "2000-03-3", "2000-04-29")))
> g <- seq(as.yearmon(start(z)), as.yearmon(end(z)), by = 1/12)
> na.locf(z, x = as.yearmon, xout = g)

Jan 2000 Feb 2000 Mar 2000 Apr 2000
       1        1        2        3
```

Here is a `chron` example where we wish to create a 10 minute grid:

```
> Lines <- "Time,Value
+ 2009-10-09 5:00:00,210
+ 2009-10-09 5:05:00,207
+ 2009-10-09 5:17:00,250
+ 2009-10-09 5:30:00,193
+ 2009-10-09 5:41:00,205
+ 2009-10-09 6:00:00,185"
> library("chron")
> z <- read.zoo(textConnection(Lines), FUN = as.chron, sep = ",", header = TRUE)
> g <- seq(start(z), end(z), by = times("00:10:00"))
> na.locf(z, xout = g)

(10/09/09 05:00:00) (10/09/09 05:10:00) (10/09/09 05:20:00) (10/09/09 05:30:00)
                210                 207                 250                 193
(10/09/09 05:40:00) (10/09/09 05:50:00) (10/09/09 06:00:00)
                193                 205                 185
```

What is the difference between `as.Date` *in zoo and* `as.Date` *in the core of R?*

zoo has extended the `origin` argument of `as.Date.numeric` so that it has a default of `origin="1970-01-01"` (whereas in the core of R it has no default and must always be specified). Note that this is a strictly upwardly compatible extensions to R and any usage of `as.Date` in R will also work in zoo.

This makes it more convenient to use as.Date as a function input. For example, one can shorten this:

```
> z <- zoo(1:2, c("2000-01-01", "2000-01-02"))
> aggregate(z, function(x) as.Date(x, origin = "1970-01-01"))

2000-01-01 2000-01-02
         1          2
```

to just this:

```
> aggregate(z, as.Date)
```

```
2000-01-01 2000-01-02
         1          2
```

As another example, one can shorten

```
> Lines <- "2000-01-01 12:00:00,12
+ 2000-01-02 12:00:00,13"
> read.zoo(text = Lines, sep = ",", FUN = function(x) as.Date(x, origin = "1970-01-01"))
```

```
2000-01-01 2000-01-02
        12         13
```

to this:

```
> read.zoo(text = Lines, sep = ",", FUN = as.Date)
```

```
2000-01-01 2000-01-02
        12         13
```

Note to package developers of packages that use zoo: Other packages that work with zoo and define `as.Date` methods should either import **zoo** or else should fully export their `as.Date` methods in their `NAMESPACE` file, e.g. `export(as.Date.X)`, in order that those methods be registered with **zoo**'s `as.Date` generic and not just the `as.Date` generic in **base**.

15. How can I speed up zoo?

The main area where you might notice slowness is if you do indexing of zoo objects in an inner loop. In that case extract the data and time components prior to the loop. Since most calculations in R use the whole object approach there are relatively few instances of this.

For example, the following shows two ways of performing a rolling sum using only times nearer than 3 before the current time. The second one eliminates the zoo indexing to get a speedup:

```
> n <- 50
> z <- zoo(1:n, c(1:3, seq(4, by = 2, length = n-3)))
> system.time({
+         zz <- sapply(seq_along(z),
+                 function(i) sum(z[time(z) <= time(z)[i] & time(z) > time(z)[i] - 3]))
+         z1 <- zoo(zz, time(z))
+ })
```

```
   user  system elapsed
  0.016   0.000   0.017
```

```
> system.time({
+         zc <- coredata(z)
+         tt <- time(z)
+         zr <- sapply(seq_along(zc),
+                 function(i) sum(zc[tt <= tt[i] & tt > tt[i] - 3]))
+         z2 <- zoo(zr, tt)
+ })
```

```
   user  system elapsed 
  0.004   0.000   0.001 
```

```
> identical(z1, z2)
```

```
[1] TRUE
```


Affiliation:

zoo Development Team
R-Forge: http://R-Forge.R-project.org/projects/zoo/
Comprehensive R Archive Network: http://CRAN.R-project.org/package=zoo
