# Supplementary material for: Comparing Kaolin and Pinolene to Improve Sustainable Grapevine Production during Drought
Source: PLoS One. 2016 Jun 13;11(6):e0156631. doi: 10.1371/journal.pone.0156631 (PMC4905681; doi:10.1371/journal.pone.0156631)
Supplement: S1 File — (GZ) [file pone.0156631.s003.gz › PONE_Brillante_et_al_2016_Code_and_Data/packrat/src/zoo/zoo_1.7-12.tar.gz!/zoo/inst/doc/zoo-design.pdf]

zoo Design

zoo Development Team

Abstract

This is a set of design principles that – albeit not having been explicitly set out initially – have guided the development of the R **zoo** package.

Keywords: irregular time series, ordered observations, time index.

***zoo** works with any ordered index class* having the prescribed methods, see `?zoo` and Zeileis and Grothendieck (2005). Specific index classes are not hard-coded in functions (with a few exceptions necessitated by external interfaces such as reading and interfacing with '`ts`') but rather all index operations are only done via the allowed methods.

***zoo** is invisible.* As far as possible new names (e.g., for functions or methods and their arguments) are not introduced. New functionality is added as methods to generics from base R allowing reuse of those names. When names are added, thought is put into the name since **zoo** is so miserly about adding them. For example, the `read.zoo` name is obviously derived from `read.table` and the names `na.locf` or `na.approx` are in analogy to other `na.*` functions frome base R.

***zoo** is consistent with base R.* The idea is to make the usage of **zoo** completely natural to someone who already has experience with R (in particular with the '`ts`' class). Certainly, **zoo** contains extensions to base R, but they should be as consistent as possible and have a natural 'look and feel'.

***zoo** is infrastructure.* **zoo** tries to do basic things well, e.g., data reading, handling, aggregation, transformation, etc. However, it does not provide time series modeling functionality – **zoo** rather encourages add-on packages. A list of current packages employing **zoo** can be found on the Comprehensive R Archive Network (CRAN) and in the **zoo** FAQ, see `vignette("zoo-faq", package = "zoo")`.

***zoo** interfaces to all other time series packages* on CRAN (or at least all of the more popular ones and some of the relatively unknown ones as well). Thus, time series information can be passed back and forth relatively easily between '`zoo`' and other time series classes and hence combinations of various functionalities are facilitated.

***zoo** includes extensive documentation.* In addition to the standard `help()` pages, **zoo** provides a set of vignettes, see `vignette(package = "zoo")` for a list. Furthermore, the **zoo** Development Team members also post frequently on the mailing lists (especially "R-help", "R-devel", and "R-SIG-Finance") whose archives hence contain many useful code snippets etc.

***zoo** has no bug list since all bugs are fixed (almost) immediately.* Over the last years, **zoo** has grown into a mature package. Nevertheless, there are always still bugs (or border cases that are not handled well). When reported to the development team by e-mail, these typically get fixed immediately in the Subversion (SVN) repository on R-Forge. (As of this writing, there

are a couple of entries in the **zoo** R-Forge bug list, however typically this is not the situation.)

zoo *includes formal regression testing.* We have started developing formal regression tests employing R's own system (in `zoo/tests/`) as well as the **RUnit** package (Burger, Jünemann, and König 2009).

References

Burger M, Jünemann K, König T (2009). ***RUnit**: R Unit Test Framework*. R package version 0.4.25, URL `http://CRAN.R-project.org/package=RUnit`.

Zeileis A, Grothendieck G (2005). "**zoo**: S3 Infrastructure for Regular and Irregular Time Series." *Journal of Statistical Software*, **14**(6), 1–27. URL `http://www.jstatsoft.org/v14/i06/`.

Affiliation:

zoo Development Team
R-Forge: `http://R-Forge.R-project.org/projects/zoo/`
Comprehensive R Archive Network: `http://CRAN.R-project.org/package=zoo`
