# Supplementary material for: Comparing Kaolin and Pinolene to Improve Sustainable Grapevine Production during Drought
Source: PLoS One. 2016 Jun 13;11(6):e0156631. doi: 10.1371/journal.pone.0156631 (PMC4905681; doi:10.1371/journal.pone.0156631)
Supplement: S1 File — (GZ) [file pone.0156631.s003.gz › PONE_Brillante_et_al_2016_Code_and_Data/packrat/src/zoo/zoo_1.7-12.tar.gz!/zoo/inst/doc/zoo-read.pdf]

Reading Data in zoo

Gabor Grothendieck
GKX Associates Inc.

Achim Zeileis
Universität Innsbruck

Abstract

This vignette gives examples of how to read data in various formats in the **zoo** package using the `read.zoo()` function. The function `read.zoo()` function expects either a text `file` (or text connection) as input or data frame. The former case is handled by first using `read.table()` to produce the data frame. (Instead of a text `file`, the `text` argument can be used to read a text string that is already stored in R which is used in the examples of this vignette.) Subsequently, `read.zoo()` provides a wide collection of convenience functionality to turn that data frame into a '`zoo`' series with a specific structure and a specific time index. In this vignette, an overview is provided of the wide variety of cases that can be handled with `read.zoo()`. All examples assume that **zoo** is already loaded and (if necessary) that the **chron** package has been loaded as well.

Keywords: irregular time series, daily data, weekly data, data frame, text file.

Example 1

Input class: Text file/connection (space-separated with header).

Input index: '`integer`'.

Output class: Multivariate '`zoo`' series.

Output index: '`integer`'.

Strategy: No transformation of time index needed, hence only a simple call to `read.zoo()`.

```
R> Lines <- "
+ time latitude longitude altitude  distance heartrate
+ 1277648884 0.304048 -0.793819      260  0.000000        94
+ 1277648885 0.304056 -0.793772      262  4.307615        95
+ 1277648894 0.304075 -0.793544      263 25.237911       103
+ 1277648902 0.304064 -0.793387      256 40.042988       115
+ "
R> z <- read.zoo(text = Lines, header = TRUE)
R> z
```

```
           latitude longitude altitude  distance heartrate
1277648884 0.304048 -0.793819      260  0.000000        94
1277648885 0.304056 -0.793772      262  4.307615        95
1277648894 0.304075 -0.793544      263 25.237911       103
1277648902 0.304064 -0.793387      256 40.042988       115
```

Example 2

Input class: ‘`data.frame`’.

Input index: ‘`factor`’ with labels indicating AM/PM times but no date.

Output class: Multivariate ‘`zoo`’ series.

Output index: ‘`times`’ (from **chron**).

Strategy: The idea is to add some dummy date (here 1970-01-01) to the ‘`character`’ lables, then transform to ‘`chron`’ and extract the ‘`times`’.

```
R> DF <- structure(list(
+   Time = structure(1:5, .Label = c("7:10:03 AM", "7:10:36 AM",
+     "7:11:07 AM", "7:11:48 AM", "7:12:25 AM"), class = "factor"),
+   Bid = c(6118.5, 6118.5, 6119.5, 6119, 6119),
+   Offer = c(6119.5, 6119.5, 6119.5, 6120, 6119.5)),
+   .Names = c("Time", "Bid", "Offer"), row.names = c(NA, -5L),
+   class = "data.frame")
R> DF

        Time    Bid  Offer
1 7:10:03 AM 6118.5 6119.5
2 7:10:36 AM 6118.5 6119.5
3 7:11:07 AM 6119.5 6119.5
4 7:11:48 AM 6119.0 6120.0
5 7:12:25 AM 6119.0 6119.5

R> z <- read.zoo(DF, FUN = function(x)
+   times(as.chron(paste("1970-01-01", x), format = "%Y-%m-%d %H:%M:%S %p")))
R> z

            Bid  Offer
07:10:03 6118.5 6119.5
07:10:36 6118.5 6119.5
07:11:07 6119.5 6119.5
07:11:48 6119.0 6120.0
07:12:25 6119.0 6119.5
```

Example 3

Input class: Text file/connection (semicolon-separated with header).

Input index: '`factor`'s with labels indicating dates (column 1) and times (column 2).

Output class: Multivariate '`zoo`' series, with separate columns for each date.

Output index: '`times`' (from **chron**).

Strategy: Split the data based on date (column 1) and process times (column 2) to '`times`'. Enhance column names at the end.

```
R> Lines <- "
+ Date;Time;Close
+ 01/09/2009;10:00;56567
+ 01/09/2009;10:05;56463
+ 01/09/2009;10:10;56370
+ 01/09/2009;16:45;55771
+ 01/09/2009;16:50;55823
+ 01/09/2009;16:55;55814
+ 02/09/2009;10:00;55626
+ 02/09/2009;10:05;55723
+ 02/09/2009;10:10;55659
+ 02/09/2009;16:45;55742
+ 02/09/2009;16:50;55717
+ 02/09/2009;16:55;55385
+ "
R> f <- function(x) times(paste(x, 0, sep = ":"))
R> z <- read.zoo(text = Lines, header = TRUE, sep = ";",
+   split = 1, index = 2, FUN = f)
R> colnames(z) <- sub("X(..).(..).(....)", "\\3-\\2-\\1", colnames(z))
R> z
```

```
         01/09/2009 02/09/2009
10:00:00      56567      55626
10:05:00      56463      55723
10:10:00      56370      55659
16:45:00      55771      55742
16:50:00      55823      55717
16:55:00      55814      55385
```

Example 4

Input class: Text file/connection (space-separated with header).

Input index: '`factor`'s with labels indicating dates (column 1) and times (column 2).

Output class: Multivariate '`zoo`' series.

Output index: '`chron`' (from **chron**).

Strategy: Indicate vector of two columns in `index`, which is subsequently processed by a `FUN` taking two arguments and returning a '`chron`' time/date.

```
R> Lines <- "
+ Date Time O H L C
+ 1/2/2005 17:05 1.3546 1.3553 1.3546 1.35495
+ 1/2/2005 17:10 1.3553 1.3556 1.3549 1.35525
+ 1/2/2005 17:15 1.3556 1.35565 1.35515 1.3553
+ 1/2/2005 17:25 1.355 1.3556 1.355 1.3555
+ 1/2/2005 17:30 1.3556 1.3564 1.35535 1.3563
+ "
R> f <- function(d, t) as.chron(paste(as.Date(chron(d)), t))
R> z <- read.zoo(text = Lines, header = TRUE, index = 1:2, FUN = f)
R> z
```

```
                          O       H       L       C
(01/02/05 17:05:00) 1.3546 1.35530 1.35460 1.35495
(01/02/05 17:10:00) 1.3553 1.35560 1.35490 1.35525
(01/02/05 17:15:00) 1.3556 1.35565 1.35515 1.35530
(01/02/05 17:25:00) 1.3550 1.35560 1.35500 1.35550
(01/02/05 17:30:00) 1.3556 1.35640 1.35535 1.35630
```

Example 5

Input class: Text file/connection (space-separated with non-matching header).

Input index: 'factor''s with labels indicating dates (column 6) and unneeded weekdays (column 5) and times (column 7).

Output class: Multivariate 'zoo' series.

Output index: 'Date'.

Strategy: First, skip the header line, remove unneeded columns by setting colClasses to "NULL", and set suitable col.names. Second, convert the date column to a 'Date' index using format. Finally, aggregate over duplicate dates, keeping only the last observation.

```
R> Lines <-
+ "  views  number  timestamp day            time
+ 1  views  910401 1246192687 Sun 6/28/2009 12:38
+ 2  views  921537 1246278917 Mon 6/29/2009 12:35
+ 3  views  934280 1246365403 Tue 6/30/2009 12:36
+ 4  views  986463 1246888699 Mon  7/6/2009 13:58
+ 5  views  995002 1246970243 Tue  7/7/2009 12:37
+ 6  views 1005211 1247079398 Wed  7/8/2009 18:56
+ 7  views 1011144 1247135553 Thu  7/9/2009 10:32
+ 8  views 1026765 1247308591 Sat 7/11/2009 10:36
+ 9  views 1036856 1247436951 Sun 7/12/2009 22:15
+ 10 views 1040909 1247481564 Mon 7/13/2009 10:39
+ 11 views 1057337 1247568387 Tue 7/14/2009 10:46
+ 12 views 1066999 1247665787 Wed 7/15/2009 13:49
+ 13 views 1077726 1247778752 Thu 7/16/2009 21:12
+ 14 views 1083059 1247845413 Fri 7/17/2009 15:43
+ 15 views 1083059 1247845824 Fri 7/17/2009 18:45
+ 16 views 1089529 1247914194 Sat 7/18/2009 10:49
+ "
R> cl <- c("NULL", "numeric", "character")[c(1, 1, 2, 2, 1, 3, 1)]
R> cn <- c(NA, NA, "views", "number", NA, NA, NA)
R> z <- read.zoo(text = Lines, skip = 1, col.names = cn, colClasses = cl,
+   index = 3, format = "%m/%d/%Y",
+   aggregate = function(x) tail(x, 1))
R> z

             views     number
2009-06-28  910401 1246192687
2009-06-29  921537 1246278917
2009-06-30  934280 1246365403
2009-07-06  986463 1246888699
2009-07-07  995002 1246970243
2009-07-08 1005211 1247079398
2009-07-09 1011144 1247135553
2009-07-11 1026765 1247308591
```

```
2009-07-12 1036856 1247436951
2009-07-13 1040909 1247481564
2009-07-14 1057337 1247568387
2009-07-15 1066999 1247665787
2009-07-16 1077726 1247778752
2009-07-17 1083059 1247845824
2009-07-18 1089529 1247914194
```

Extract all Thursdays and Fridays.

```
R> (z45 <- z[format(time(z), "%w") %in% 4:5,])

             views     number
2009-07-09 1011144 1247135553
2009-07-16 1077726 1247778752
2009-07-17 1083059 1247845824
```

Keep last entry in each week.

```
R> z45[!duplicated(format(time(z45), "%U"), fromLast = TRUE), ]

             views     number
2009-07-09 1011144 1247135553
2009-07-17 1083059 1247845824
```

Alternative approach: Above approach labels each point as it was originally labeled, i.e., if Thursday is used it gets the date of that Thursday. Another approach is to always label the resulting point as Friday and also use the last available value even if its not Thursday.

Create daily grid and fill in so Friday is filled in with prior value if Friday is `NA`.

```
R> g <- seq(start(z), end(z), by = "day")
R> z.filled <- na.locf(z, xout = g)
```

Extract Fridays, including those filled in from previous day.

```
R> z.filled[format(time(z.filled), "%w") == "5", ]

             views     number
2009-07-03  934280 1246365403
2009-07-10 1011144 1247135553
2009-07-17 1083059 1247845824
```

Example 6

Input class: Text file/connection (comma-separated with header).

Input index: `factor`'s with labels indicating dates (column 1) and times (column 2).

Output class: Multivariate `zoo` series.

Output index: `chron` (from **chron**) or `POSIXct`.

Strategy: Three versions, all using vector `index = 1:2`.

```
R> Lines <- "
+ Date,Time,Open,High,Low,Close,Up,Down
+ 05.02.2001,00:30,421.20,421.20,421.20,421.20,11,0
+ 05.02.2001,01:30,421.20,421.40,421.20,421.40,7,0
+ 05.02.2001,02:00,421.30,421.30,421.30,421.30,0,5"
```

With custom `FUN` using `chron()` after appending seconds.

```
R> f <- function(d, t) chron(d, paste(t, "00", sep = ":"),
+   format = c("m.d.y", "h:m:s"))
R> z <- read.zoo(text = Lines, sep = ",", header = TRUE,
+   index = 1:2, FUN  = f)
R> z

                     Open  High   Low Close Up Down
(05.02.01 00:30:00) 421.2 421.2 421.2 421.2 11    0
(05.02.01 01:30:00) 421.2 421.4 421.2 421.4  7    0
(05.02.01 02:00:00) 421.3 421.3 421.3 421.3  0    5
```

With custom `FUN` using `as.chron()` with suitable `format`.

```
R> f2 <- function(d, t) as.chron(paste(d, t), format = "%d.%m.%Y %H:%M")
R> z2 <- read.zoo(text = Lines, sep = ",", header = TRUE,
+   index = 1:2, FUN  = f2)
R> z2

                     Open  High   Low Close Up Down
(02/05/01 00:30:00) 421.2 421.2 421.2 421.2 11    0
(02/05/01 01:30:00) 421.2 421.4 421.2 421.4  7    0
(02/05/01 02:00:00) 421.3 421.3 421.3 421.3  0    5
```

Without `FUN`, hence the `index` columns are pasted together and then passt do `as.POSIXct()` because `tz` and `format` are specified.

```
R> z3 <- read.zoo(text = Lines, sep = ",", header = TRUE,
+   index = 1:2, tz = "", format = "%d.%m.%Y %H:%M")
R> z3

                     Open  High   Low Close Up Down
2001-02-05 00:30:00 421.2 421.2 421.2 421.2 11    0
2001-02-05 01:30:00 421.2 421.4 421.2 421.4  7    0
2001-02-05 02:00:00 421.3 421.3 421.3 421.3  0    5
```

Example 7

Input class: Text file/connection (space-separated with header).

Input index: '`factor`'s with labels indicating dates (column 1) and times (column 2).

Output class: Multivariate '`zoo`' series.

Output index: '`POSIXct`'.

Strategy: Due to standard date/time formats, only `index = 1:2` and `tz = ""` need to be specified to produce '`POSIXct`' index.

```
R> Lines <- "Date Time V2   V3   V4   V5
+ 2010-10-15 13:43:54 73.8 73.8 73.8 73.8
+ 2010-10-15 13:44:15 73.8 73.8 73.8 73.8
+ 2010-10-15 13:45:51 73.8 73.8 73.8 73.8
+ 2010-10-15 13:46:21 73.8 73.8 73.8 73.8
+ 2010-10-15 13:47:27 73.8 73.8 73.8 73.8
+ 2010-10-15 13:47:54 73.8 73.8 73.8 73.8
+ 2010-10-15 13:49:51 73.7 73.7 73.7 73.7
+ "
R> z <- read.zoo(text = Lines, header = TRUE, index = 1:2, tz = "")
R> z

                      V2   V3   V4   V5
2010-10-15 13:43:54 73.8 73.8 73.8 73.8
2010-10-15 13:44:15 73.8 73.8 73.8 73.8
2010-10-15 13:45:51 73.8 73.8 73.8 73.8
2010-10-15 13:46:21 73.8 73.8 73.8 73.8
2010-10-15 13:47:27 73.8 73.8 73.8 73.8
2010-10-15 13:47:54 73.8 73.8 73.8 73.8
2010-10-15 13:49:51 73.7 73.7 73.7 73.7
```

Example 8

Input class: Text file/connection (space-separated without header).

Input index: 'factor' with labels indicating dates.

Output class: Multivariate 'zoo' series, with separate columns depending on column 2.

Output index: 'Date'.

Strategy: Non-standard na.strings format needs to be specified, series is split based on second column, and date format (in column 1, default) needs to be specified.

```
R> Lines <- "
+ 13/10/2010      A       23
+ 13/10/2010      B       12
+ 13/10/2010      C       124
+ 14/10/2010      A       43
+ 14/10/2010      B       54
+ 14/10/2010      C       65
+ 15/10/2010      A       43
+ 15/10/2010      B       N.A.
+ 15/10/2010      C       65
+ "
R> z <- read.zoo(text = Lines, na.strings = "N.A.",
+   format = "%d/%m/%Y", split = 2)
R> z
```

```
            A  B   C
2010-10-13 23 12 124
2010-10-14 43 54  65
2010-10-15 43 NA  65
```

Example 9

Input class: Text file/connection (comma-separated with header).

Input index: '`factor`' with labels indicating date/time.

Output class: Univariate '`zoo`' series.

Output index: '`chron`' (from **chron**) or '`POSIXct`'.

Strategy: Ignore first two columns by setting `colClasses` to `"NULL"`. Either produce '`chron`' index via `as.chron()` or use all defaults to produce '`POSIXct`' by setting `tz`.

```
R> Lines <- '
+ "","Fish_ID","Date","R2sqrt"
+ "1",1646,2006-08-18 08:48:59,0
+ "2",1646,2006-08-18 09:53:20,100
+ '
R> z <- read.zoo(text = Lines, header = TRUE, sep = ",",
+   colClasses = c("NULL", "NULL", "character", "numeric"),
+   FUN = as.chron)
R> z

(08/18/06 08:48:59) (08/18/06 09:53:20)
                  0                 100

R> z2 <- read.zoo(text = Lines, header = TRUE, sep = ",",
+   colClasses = c("NULL", "NULL", "character", "numeric"),
+   tz = "")
R> z2

2006-08-18 08:48:59 2006-08-18 09:53:20
                  0                 100
```

Example 10

Input class: Text file/connection (space-separated with non-matching header).

Input index: '`factor`' with labels indicating date (column 3) and time (column 4).

Output class: Multivariate '`zoo`' series.

Output index: '`chron`' (from **chron**) or '`POSIXct`'.

Strategy: `skip` non-matching header and extract date/time from two columns `index = 3:4`. Either using sequence of two functions `FUN` and `FUN2` or employ defaults yielding '`POSIXct`'.

```
R> Lines <-
+ " iteration         Datetime    VIC1    NSW1     SA1    QLD1
+ 1         1 2011-01-01 00:30 5482.09 7670.81 2316.22 5465.13
+ 2         1 2011-01-01 01:00 5178.33 7474.04 2130.30 5218.61
+ 3         1 2011-01-01 01:30 4975.51 7163.73 2042.39 5058.19
+ 4         1 2011-01-01 02:00 5295.36 6850.14 1940.19 4897.96
+ 5         1 2011-01-01 02:30 5042.64 6587.94 1836.19 4749.05
+ 6         1 2011-01-01 03:00 4799.89 6388.51 1786.32 4672.92
+ "
R> z <- read.zoo(text = Lines, skip = 1, index = 3:4,
+   FUN = paste, FUN2 = as.chron)
R> z

                    V1 V2      V5      V6      V7      V8
(01/01/11 00:30:00)  1  1 5482.09 7670.81 2316.22 5465.13
(01/01/11 01:00:00)  2  1 5178.33 7474.04 2130.30 5218.61
(01/01/11 01:30:00)  3  1 4975.51 7163.73 2042.39 5058.19
(01/01/11 02:00:00)  4  1 5295.36 6850.14 1940.19 4897.96
(01/01/11 02:30:00)  5  1 5042.64 6587.94 1836.19 4749.05
(01/01/11 03:00:00)  6  1 4799.89 6388.51 1786.32 4672.92

R> z2 <- read.zoo(text = Lines, skip = 1, index = 3:4, tz = "")
R> z2

                    V1 V2      V5      V6      V7      V8
2011-01-01 00:30:00  1  1 5482.09 7670.81 2316.22 5465.13
2011-01-01 01:00:00  2  1 5178.33 7474.04 2130.30 5218.61
2011-01-01 01:30:00  3  1 4975.51 7163.73 2042.39 5058.19
2011-01-01 02:00:00  4  1 5295.36 6850.14 1940.19 4897.96
2011-01-01 02:30:00  5  1 5042.64 6587.94 1836.19 4749.05
2011-01-01 03:00:00  6  1 4799.89 6388.51 1786.32 4672.92
```

Example 11

Input class: ‘data.frame’.

Input index: ‘Date’.

Output class: Multivariate ‘zoo’ series.

Output index: ‘Date’.

Strategy: Given a ‘data.frame’ only keep last row in each month. Use `read.zoo()` to convert to ‘zoo’ and then `na.locf()` and `duplicated()`.

```
R> DF <- structure(list(
+   Date = structure(c(14609, 14638, 14640, 14666, 14668, 14699,
+     14729, 14757, 14759, 14760), class = "Date"),
+   A = c(4.9, 5.1, 5, 4.8, 4.7, 5.3, 5.2, 5.4, NA, 4.6),
+   B = c(18.4, 17.7, NA, NA, 18.3, 19.4, 19.7, NA, NA, 18.1),
+   C = c(32.6, NA, 32.8, NA, 33.7, 32.4, 33.6, NA, 34.5, NA),
+   D = c(77, NA, 78.7, NA, 79, 77.8, 79, 81.7, NA, NA)),
+   .Names = c("Date", "A", "B", "C", "D"), row.names = c(NA, -10L),
+   class = "data.frame")
R> DF
```

```
         Date   A    B    C    D
1  2009-12-31 4.9 18.4 32.6 77.0
2  2010-01-29 5.1 17.7   NA   NA
3  2010-01-31 5.0   NA 32.8 78.7
4  2010-02-26 4.8   NA   NA   NA
5  2010-02-28 4.7 18.3 33.7 79.0
6  2010-03-31 5.3 19.4 32.4 77.8
7  2010-04-30 5.2 19.7 33.6 79.0
8  2010-05-28 5.4   NA   NA 81.7
9  2010-05-30  NA   NA 34.5   NA
10 2010-05-31 4.6 18.1   NA   NA
```

```
R> z <- read.zoo(DF)
R> na.locf(z)[!duplicated(as.yearmon(time(z)), fromLast = TRUE)]
```

```
             A    B    C    D
2009-12-31 4.9 18.4 32.6 77.0
2010-01-31 5.0 17.7 32.8 78.7
2010-02-28 4.7 18.3 33.7 79.0
2010-03-31 5.3 19.4 32.4 77.8
2010-04-30 5.2 19.7 33.6 79.0
2010-05-31 4.6 18.1 34.5 81.7
```

Example 12

Input class: Text file/connection (space-separated without header).

Input index: ‘`factor`’ with labels indicating dates.

Output class: Univariate ‘`zoo`’ series.

Output index: ‘`Date`’.

Strategy: Only keep last point in case of duplicate dates.

```
R> Lines <- "
+ 2009-10-07      0.009378
+ 2009-10-19      0.014790
+ 2009-10-23      -0.005946
+ 2009-10-23      0.009096
+ 2009-11-08      0.004189
+ 2009-11-10      -0.004592
+ 2009-11-17      0.009397
+ 2009-11-24      0.003411
+ 2009-12-02      0.003300
+ 2010-01-15      0.010873
+ 2010-01-20      0.010712
+ 2010-01-20      0.022237
+ "
R> z <- read.zoo(text = Lines, aggregate = function(x) tail(x, 1))
R> z
```

```
2009-10-07 2009-10-19 2009-10-23 2009-11-08 2009-11-10 2009-11-17 2009-11-24
  0.009378   0.014790   0.009096   0.004189  -0.004592   0.009397   0.003411
2009-12-02 2010-01-15 2010-01-20
  0.003300   0.010873   0.022237
```

Example 13

Input class: Text file/connection (comma-separated with header).

Input index: ‘`factor`’ with labels indicating date/time.

Output class: Multivariate ‘`zoo`’ series.

Output index: ‘`POSIXct`’ or ‘`chron`’ (from **chron**).

Strategy: Dates and times are in standard format, hence the default ‘`POSIXct`’ can be produced by setting `tz` or, alternatively, ‘`chron`’ can be produced by setting `as.chron()` as `FUN`.

```
R> Lines <- "
+ timestamp,time-step-index,value
+ 2009-11-23 15:58:21,23301,800
+ 2009-11-23 15:58:29,23309,950
+ "
R> z <- read.zoo(text = Lines, header = TRUE, sep = ",", tz = "")
R> z

                    time.step.index value
2009-11-23 15:58:21           23301   800
2009-11-23 15:58:29           23309   950

R> z2 <- read.zoo(text = Lines, header = TRUE, sep = ",", FUN = as.chron)
R> z2

                    time.step.index value
(11/23/09 15:58:21)           23301   800
(11/23/09 15:58:29)           23309   950
```

Example 14

Input class: Text file/connection (space-separated with header).

Input index: '`factor`'s with labels indicating dates (column 1) times (column 2).

Output class: Univariate '`zoo`' series.

Output index: '`chron`' (from **chron**).

Strategy: Indicate vector `index = 1:2` and use `chron()` (which takes two separate arguments for dates and times) to produce '`chron`' index.

```
R> Lines <- "
+ Date Time Value
+ 01/23/2000 10:12:15 12.12
+ 01/24/2000 11:10:00 15.00
+ "
R> z <- read.zoo(text = Lines, header = TRUE, index = 1:2, FUN = chron)
R> z

(01/23/00 10:12:15) (01/24/00 11:10:00)
              12.12               15.00
```

Example 15

Input class: Text file/connection (space-separated with header).

Input index: 'numeric' year with quarters represented by separate columns.

Output class: Univariate 'zoo' series.

Output index: 'yearqtr'.

Strategy: First, create a multivariate annual time series using the year index. Then, create a regular univariate quarterly series by collapsing the annual series to a vector and adding a new 'yearqtr' index from scratch.

```
R> Lines <- "
+ Year   Qtr1  Qtr2  Qtr3  Qtr4
+ 1992    566   443   329   341
+ 1993    344   212   133   112
+ 1994    252   252   199   207
+ "
R> za <- read.zoo(text = Lines, header = TRUE)
R> za

     Qtr1 Qtr2 Qtr3 Qtr4
1992  566  443  329  341
1993  344  212  133  112
1994  252  252  199  207

R> zq <- zooreg(as.vector(t(za)), start = yearqtr(start(za)), freq = 4)
R> zq

1992 Q1 1992 Q2 1992 Q3 1992 Q4 1993 Q1 1993 Q2 1993 Q3 1993 Q4 1994 Q1 1994 Q2
    566     443     329     341     344     212     133     112     252     252
1994 Q3 1994 Q4
    199     207
```

Further comments

Multiple files can be read and subsequently merged.

```
R> filenames <- dir(pattern = "csv$")
R> z <- read.zoo(filenames, header = TRUE, sep = ",", fixed = FALSE)
```

Affiliation:

Gabor Grothendieck
GKX Associates Inc.
E-mail: ggrothendieck@gmail.com

Achim Zeileis
Universität Innsbruck
E-mail: Achim.Zeileis@R-project.org
