# Supplementary material for: Comparing Kaolin and Pinolene to Improve Sustainable Grapevine Production during Drought
Source: PLoS One. 2016 Jun 13;11(6):e0156631. doi: 10.1371/journal.pone.0156631 (PMC4905681; doi:10.1371/journal.pone.0156631)
Supplement: S1 File — (GZ) [file pone.0156631.s003.gz › PONE_Brillante_et_al_2016_Code_and_Data/packrat/src/zoo/zoo_1.7-12.tar.gz!/zoo/inst/doc/zoo.pdf]

zoo: An S3 Class and Methods for Indexed Totally Ordered Observations

Achim Zeileis
Universität Innsbruck

Gabor Grothendieck
GKX Associates Inc.

Abstract

A previous version to this introduction to the R package **zoo** has been published as Zeileis and Grothendieck (2005) in the *Journal of Statistical Software*.

zoo is an R package providing an S3 class with methods for indexed totally ordered observations, such as discrete irregular time series. Its key design goals are independence of a particular index/time/date class and consistency with base R and the "ts" class for regular time series. This paper describes how these are achieved within **zoo** and provides several illustrations of the available methods for "zoo" objects which include plotting, merging and binding, several mathematical operations, extracting and replacing data and index, coercion and NA handling. A subclass "zooreg" embeds regular time series into the "zoo" framework and thus bridges the gap between regular and irregular time series classes in R.

Keywords: totally ordered observations, irregular time series, regular time series, S3, R.

1. Introduction

The R system for statistical computing (R Development Core Team 2008, http://www.R-project.org/) ships with a class for regularly spaced time series, "ts" in package **stats**, but has no native class for irregularly spaced time series. With the increased interest in computational finance with R over the last years several implementations of classes for irregular time series emerged which are aimed particularly at finance applications. These include the S4 classes "timeSeries" in package **timeSeries** (previously **fSeries**) from the **Rmetrics** suite (Wuertz 2010), "its" in package **its** (Heywood 2009) and the S3 class "irts" in package **tseries** (Trapletti 2009). With these packages available, why would anybody want yet another package providing infrastructure for irregular time series? The above mentioned implementations have in common that they are restricted to a particular class for the time scale: the former implementation comes with its own time class "timeDate" from package **timeDate** (previously **fCalendar**) built on top of the "POSIXct" class available in base R whereas the latter two use "POSIXct" directly. And this was the starting point for the **zoo** project: the first author of the present paper needed more general support for ordered observations, independent of a particular index class, for the package **strucchange** (Zeileis, Leisch, Hornik, and Kleiber 2002). Hence, the package was called **zoo** which stands for Z's ordered observations. Since the first release, a major part of the additions to **zoo** were provided by the second author of this paper, so that the name of the package does not really reflect the authorship anymore. Nevertheless, independence of a particular index class remained the most important design

goal. While the package evolved to its current status, a second key design goal became more and more clear: to provide methods to standard generic functions for the `"zoo"` class that are similar to those for the `"ts"` class (and base R in general) such that the usage of **zoo** is very intuitive because few additional commands have to be learned. This paper describes how these design goals are implemented in **zoo**. The resulting package provides the `"zoo"` class which offers an extensive (and still growing) set of standard and new methods for working with indexed observations and 'talks' to the classes `"ts"`, `"its"`, `"irts"` and `"timeSeries"`. (In addition to these independent approaches, the class `"xts"` built upon `"zoo"` was recently introduced by Ryan and Ulrich 2010, .). **zoo** also bridges the gap between regular and irregular time series by providing coercion with (virtually) no loss of information between `"ts"` and `"zoo"`. With these tools **zoo** provides the basic infrastructure for working with indexed totally ordered observations and the package can be either employed by users directly or can be a basic ingredient on top of which other more specialized applications can be built.

The remainder of the paper is organized as follows: Section 2 explains how `"zoo"` objects are created and illustrates how the corresponding methods for plotting, merging and binding, several mathematical operations, extracting and replacing data and index, coercion and `NA` handling can be used. Section 3 outlines how other packages can build on this basic infrastructure. Section 4 gives a few summarizing remarks and an outlook on future developments. Finally, an appendix provides a reference card that gives an overview of the functionality contained in **zoo**.

2. The class "zoo" and its methods

This section describes how `"zoo"` series can be created and subsequently manipulated, visualized, combined or coerced to other classes. In Section 2.1, the general class `"zoo"` for totally ordered series is described. Subsequently, in Section 2.2, the subclass `"zooreg"` for regular `"zoo"` series, i.e., series which have an index with a specified frequency, is discussed. The methods illustrated in the remainder of the section are mostly the same for both `"zoo"` and `"zooreg"` objects and hence do not have to be discussed separately. The few differences in merging and binding are briefly highlighted in Section 2.4.

2.1. Creation of "zoo" objects

The simple idea for the creation of `"zoo"` objects is to have some vector or matrix of observations `x` which are totally ordered by some index vector. In time series applications, this index is a measure of time but every other numeric, character or even more abstract vector that provides a total ordering of the observations is also suitable. Objects of class `"zoo"` are created by the function

```
zoo(x, order.by)
```

where `x` is the vector or matrix of observations[1] and `order.by` is the index by which the observations should be ordered. It has to be of the same length as `NROW(x)`, i.e., either the same

[1]In principle, more general objects can be indexed, but currently **zoo** does not support this. Development plans are that **zoo** should eventually support indexed factors, data frames and lists.

length as `x` for vectors or the same number of rows for matrices.[2] The `"zoo"` object created is essentially the vector/matrix as before but has an additional `"index"` attribute in which the index is stored.[3] Both the observations in the vector/matrix `x` and the index `order.by` can, in principle, be of arbitrary classes. However, most of the following methods (plotting, aggregating, mathematical operations) for `"zoo"` objects are typically only useful for numeric observations `x`. Special effort in the design was put into independence from a particular class for the index vector. In **zoo**, it is assumed that combination `c()`, querying the `length()`, value matching `MATCH()`, subsetting `[`, and, of course, ordering `ORDER()` work when applied to the index. In addition, an `as.character()` method might improve printed output[4] and `as.numeric()` could be used for computing distances between indexes, e.g., in interpolation. Both methods are not necessary for working with `"zoo"` objects but could be used if available. All these methods are available, e.g., for standard numeric and character vectors and for vectors of classes `"Date"`, `"POSIXct"` or `"times"` from package **chron** and `"timeDate"` in **timeDate**. Because not all required methods used to be available for `"timeDate"` in older versions of **fCalendar**, Section 3.3 has a rather outdated example how to provide such methods so that `"zoo"` objects work with `"timeDate"` indexes. To achieve this independence of the index class, new generic functions for ordering (`ORDER()`) and value matching (`MATCH()`) are introduced as the corresponding base functions `order()` and `match()` are non-generic. The default methods simply call the corresponding base functions, i.e., no new method needs to be introduced for a particular index class if the non-generic functions `order()` and `match()` work for this class. *R now also provides a new generic* `xtfrm()` *which was not available when the new generic* `ORDER()` *was introduced. If there is a* `xtfrm()` *for a class, the default* `ORDER()` *method typically works.*

To illustrate the usage of `zoo()`, we first load the package and set the random seed to make the examples in this paper exactly reproducible.

```
R> library("zoo")
R> set.seed(1071)
```

Then, we create two vectors `z1` and `z2` with `"POSIXct"` indexes, one with random observations

```
R> z1.index <- ISOdatetime(2004, rep(1:2,5), sample(28,10), 0, 0, 0)
R> z1.data <- rnorm(10)
R> z1 <- zoo(z1.data, z1.index)
```

and one with a sine wave

```
R> z2.index <- as.POSIXct(paste(2004, rep(1:2, 5), sample(1:28, 10),
+   sep = "-"))
R> z2.data <- sin(2*1:10/pi)
R> z2 <- zoo(z2.data, z2.index)
```

[2]The only case where this restriction is not imposed is for zero-length vectors, i.e., vectors that only have an index but no data.

[3]There is some limited support for indexed factors available in which case the `"zoo"` object also has an attribute `"oclass"` with the original class of `x`. This feature is still under development and might change in future versions.

[4]If an `as.character()` method is already defined, but gives not the desired output for printing, then an `index2char()` method can be defined. This is a generic convenience function used for creating character representations of the index vector and it defaults to using `as.character()`.

Furthermore, we create a matrix `Z` with random observations and a `"Date"` index

```
R> Z.index <- as.Date(sample(12450:12500, 10))
R> Z.data <- matrix(rnorm(30), ncol = 3)
R> colnames(Z.data) <- c("Aa", "Bb", "Cc")
R> Z <- zoo(Z.data, Z.index)
```

In the examples above, the generation of indexes looks a bit awkward due to the fact the indexes need to be randomly generated (and there are no special functions for random indexes because these are rarely needed in practice). In "real world" applications, the indexes are typically part of the raw data set read into R so the code would be even simpler. See Section 3 for such examples.[5]

Methods to several standard generic functions are available for `"zoo"` objects, such as `print`, `summary`, `str`, `head`, `tail` and `[` (subsetting), a few of which are illustrated in the following.

There are three printing code styles for `"zoo"` objects: vectors are by default printed in `"horizontal"` style

```
R> z1

 2004-01-05  2004-01-14  2004-01-19  2004-01-25  2004-01-27  2004-02-07
 0.74675994  0.02107873 -0.29823529  0.68625772  1.94078850  1.27384445
 2004-02-12  2004-02-16  2004-02-20  2004-02-24
 0.22170438 -2.07607585 -1.78439244 -0.19533304
```

```
R> z1[3:7]

2004-01-19 2004-01-25 2004-01-27 2004-02-07 2004-02-12
-0.2982353  0.6862577  1.9407885  1.2738445  0.2217044
```

and matrices in `"vertical"` style

```
R> Z

                   Aa         Bb          Cc
2004-02-02  1.2554339  0.6815732 -0.63292049
2004-02-08 -1.4945833  1.3234122 -1.49442269
2004-02-09 -1.8746225 -0.8732929  0.62733971
2004-02-21 -0.1453861  0.4523490 -0.14597401
2004-02-22  0.2254242  0.5383894  0.23136133
2004-02-29  1.2069552  0.3181422 -0.01129202
2004-03-05 -1.2086102  1.4237978 -0.81614483
2004-03-10 -0.1103956  1.3477425  0.95522468
2004-03-14  0.8420238 -2.7384202  0.23150695
2004-03-20 -0.1901910  0.1230887 -1.51862157
```

[5]Note, that in the code above a new `as.Date` method, provided in **zoo**, is used to convert days since 1970-01-01 to class `"Date"`. See the respective help page for more details.

```
R> Z[1:3, 2:3]

                   Bb         Cc
2004-02-02  0.6815732 -0.6329205
2004-02-08  1.3234122 -1.4944227
2004-02-09 -0.8732929  0.6273397
```

Additionally, there is a "plain" style which simply first prints the data and then the index.

Above, we have illustrated that "zoo" series can be indexed like vectors or matrices respectively, i.e., with integers correponding to their observation number (and column number). But for indexed observations, one would obviously also like to be able to index with the index class. This is also available in [which only uses vector/matrix-type subsetting if its first argument is of class "numeric", "integer" or "logical".

```
R> z1[ISOdatetime(2004, 1, c(14, 25), 0, 0, 0)]

2004-01-14 2004-01-25
0.02107873 0.68625772
```

If the index class happens to be "numeric", the index has to be either insulated in I() like z[I(i)] or the window() method can be used (see Section 2.6).

Summaries and most other methods for "zoo" objects are carried out column wise, reflecting the rectangular structure. In addition, a summary of the index is provided.

```
R> summary(z1)

     Index                           z1
 Min.   :2004-01-05 00:00:00   Min.   :-2.07608
 1st Qu.:2004-01-20 12:00:00   1st Qu.:-0.27251
 Median :2004-02-01 12:00:00   Median : 0.12139
 Mean   :2004-02-01 09:36:00   Mean   : 0.05364
 3rd Qu.:2004-02-15 00:00:00   3rd Qu.: 0.73163
 Max.   :2004-02-24 00:00:00   Max.   : 1.94079
```

```
R> summary(Z)

     Index                  Aa                Bb                Cc
 Min.   :2004-02-02   Min.   :-1.8746   Min.   :-2.7384   Min.   :-1.51862
 1st Qu.:2004-02-12   1st Qu.:-0.9540   1st Qu.: 0.1719   1st Qu.:-0.77034
 Median :2004-02-25   Median :-0.1279   Median : 0.4954   Median :-0.07863
 Mean   :2004-02-25   Mean   :-0.1494   Mean   : 0.2597   Mean   :-0.25739
 3rd Qu.:2004-03-08   3rd Qu.: 0.6879   3rd Qu.: 1.1630   3rd Qu.: 0.23147
 Max.   :2004-03-20   Max.   : 1.2554   Max.   : 1.4238   Max.   : 0.95522
```

2.2. Creation of "zooreg" objects

Strictly regular series are such series observations where the distance between the indexes of every two adjacent observations is the same. Such series can also be described by their frequency, i.e., the reciprocal value of the distance between two observations. As "zoo" can be used to store series with arbitrary type of index, it can, of course, also be used to store series with regular indexes. So why should this case be given special attention, in particular as there is already the "ts" class devoted entirely to regular series? There are two reasons: First, to be able to convert back and forth between "ts" and "zoo", the frequency of a certain series needs to be stored on the "zoo" side. Second, "ts" is limited to strictly regular series and the regularity is lost if some internal observations are omitted. Series that can be created by omitting some internal observations from strictly regular series will in the following be refered to as being (weakly) regular. Therefore, a class that bridges the gap between irregular and strictly regular series is needed and "zooreg" fills this gap. Objects of class "zooreg" inherit from class "zoo" but have an additional attribute "frequency" in which the frequency of the series is stored. Therefore, they can be employed to represent both strictly and weakly regular series.

To create a "zooreg" object, either the command `zoo()` can be used or the command `zooreg()`.

```
zoo(x, order.by, frequency)
zooreg(data, start, end, frequency, deltat, ts.eps, order.by)
```

If `zoo()` is called as in the previous section but with an additional `frequency` argument, it is checked whether `frequency` complies with the index `order.by`: if it does an object of class "zooreg" inheriting from "zoo" is returned. The command `zooreg()` takes mostly the same arguments as `ts()`.[6] In both cases, the index class is more restricted than in the plain "zoo" case. The index must be of a class which can be coerced to "numeric" (for checking its regularity) and when converted to numeric the index must be expressable as multiples of 1/frequency. Furthermore, adding/substracting a numeric to/from an observation of the index class, should return the correct value of the index class again, i.e., group generic functions `Ops` should be defined.[7]

The following calls yield equivalent series

```
R> zr1 <- zooreg(sin(1:9), start = 2000, frequency = 4)
R> zr2 <- zoo(sin(1:9), seq(2000, 2002, by = 1/4), 4)
R> zr1

   2000(1)    2000(2)    2000(3)    2000(4)    2001(1)    2001(2)    2001(3) 
 0.8414710  0.9092974  0.1411200 -0.7568025 -0.9589243 -0.2794155  0.6569866 
   2001(4)    2002(1) 
 0.9893582  0.4121185 

R> zr2
```

[6]Only if `order.by` is specified in the `zooreg()` call, then `zoo(x, order.by, frequency)` is called.

[7]An application of non-numeric indexes for regular series are the classes "yearmon" and "yearqtr" which are designed for monthly and quarterly series respectively and are discussed in Section 3.4.

```
  2000(1)    2000(2)    2000(3)    2000(4)    2001(1)    2001(2)    2001(3) 
0.8414710  0.9092974  0.1411200 -0.7568025 -0.9589243 -0.2794155  0.6569866 
  2001(4)    2002(1) 
0.9893582  0.4121185 
```

to which methods to standard generic functions for regular series can be applied, such as `frequency`, `deltat`, `cycle`.

As stated above, the advantage of `"zooreg"` series is that they remain regular even if an internal observation is dropped:

```
R> zr1 <- zr1[-c(3, 5)]
R> zr1
```

```
  2000(1)    2000(2)    2000(4)    2001(2)    2001(3)    2001(4)    2002(1) 
0.8414710  0.9092974 -0.7568025 -0.2794155  0.6569866  0.9893582  0.4121185 
```

```
R> class(zr1)
```

```
[1] "zooreg" "zoo"
```

```
R> frequency(zr1)
```

```
[1] 4
```

This facilitates `NA` handling significantly compared to `"ts"` and makes `"zooreg"` a much more attractive data type, e.g., for time series regression.

`zooreg()` can also deal with non-numeric indexes provided that adding `"numeric"` observations to the index class preserves the class and does not coerce to `"numeric"`.

```
R> zooreg(1:5, start = as.Date("2005-01-01"))
```

```
2005-01-01 2005-01-02 2005-01-03 2005-01-04 2005-01-05 
         1          2          3          4          5 
```

To check whether a certain series is (strictly) regular, the new generic function `is.regular(x, strict = FALSE)` can be used:

```
R> is.regular(zr1)
```

```
[1] TRUE
```

```
R> is.regular(zr1, strict = TRUE)
```

```
[1] FALSE
```

This function (and also the `frequency`, `deltat` and `cycle`) also work for `"zoo"` objects if the regularity can still be inferred from the data:

```
R> zr1 <- as.zoo(zr1)
R> zr1

     2000   2000.25   2000.75   2001.25    2001.5   2001.75      2002 
0.8414710 0.9092974 -0.7568025 -0.2794155 0.6569866 0.9893582 0.4121185 

R> class(zr1)

[1] "zoo"

R> is.regular(zr1)

[1] TRUE

R> frequency(zr1)

[1] 4
```

Of course, inferring the underlying regularity is not always reliable and it is safer to store a regular series as a `"zooreg"` object if it is intended to be a regular series.

If a weakly regular series is coerced to `"ts"` the missing observations are filled with `NA`s (see also Section 2.8). For strictly regular series with numeric index, the class can be switched between `"zoo"` and `"ts"` without loss of information.

```
R> as.ts(zr1)

          Qtr1       Qtr2       Qtr3       Qtr4
2000  0.8414710  0.9092974         NA -0.7568025
2001         NA -0.2794155  0.6569866  0.9893582
2002  0.4121185                                 

R> identical(zr2, as.zoo(as.ts(zr2)))

[1] TRUE
```

This enables direct use of functions such as `acf`, `arima`, `stl` etc. on `"zooreg"` objects as these methods coerce to `"ts"` first. The result only has to be coerced back to `"zoo"`, if appropriate.

2.3. Plotting

The `plot` method for `"zoo"` objects, in particular for multivariate `"zoo"` series, is based on the corresponding method for (multivariate) regular time series. It relies on `plot` and `lines` methods being available for the index class which can plot the index against the observations.

By default the `plot` method creates a panel for each series

```
R> plot(Z)
```

but can also display all series in a single panel

```
R> plot(Z, plot.type = "single", col = 2:4)
```

In both cases additional graphical parameters like color `col`, plotting character `pch` and line type `lty` can be expanded to the number of series. But the `plot` method for `"zoo"` objects offers some more flexibility in specification of graphical parameters as in

```
R> plot(Z, type = "b", lty = 1:3, pch = list(Aa = 1:5, Bb = 2, Cc = 4),
+    col = list(Bb = 2, 4))
```

The argument `lty` behaves as before and sets every series in another line type. The `pch` argument is a named list that assigns to each series a different vector of plotting characters each of which is expanded to the number of observations. Such a list does not necessarily have to include the names of all series, but can also specify a subset. For the remaining series the default parameter is then used which can again be changed: e.g., in the above example the `col` argument is set to display the series `"Bb"` in red and all remaining series in blue. The results of the multiple panel plots are depicted in Figure 2 and the single panel plot in Figure 1.

2.4. Merging and binding

As for many rectangular data formats in R, there are both methods for combining the rows and columns of `"zoo"` objects respectively. For the `rbind` method the number of columns of the combined objects has to be identical and the indexes may not overlap.

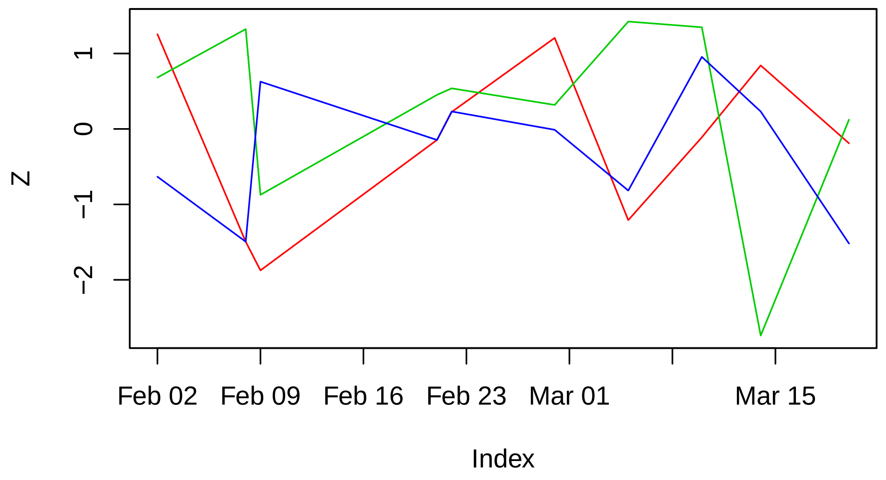

Figure 1: Example of a single panel plot

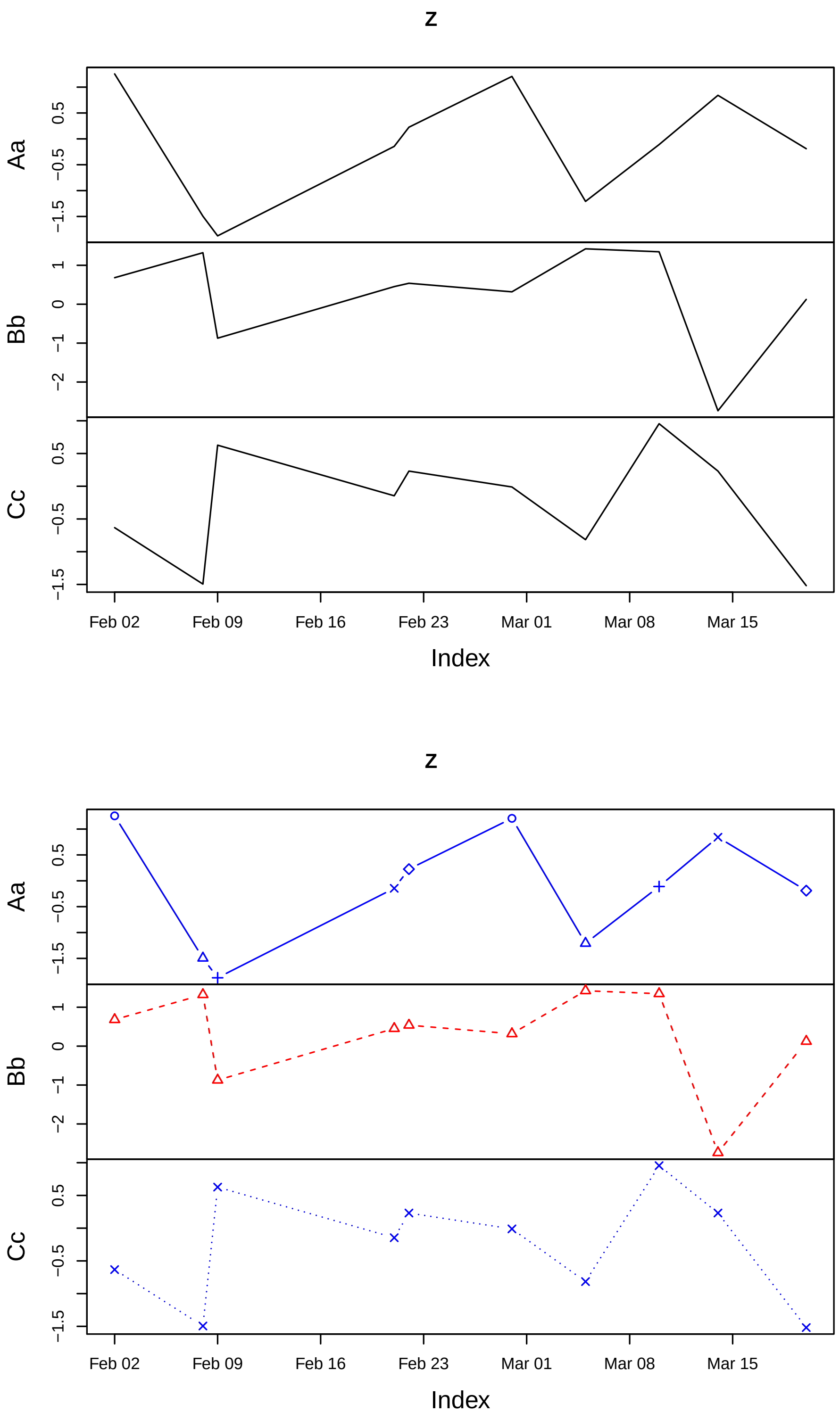

Figure 2: Examples of multiple panel plots

```
R> rbind(z1[5:10], z1[2:3])

 2004-01-14  2004-01-19  2004-01-27  2004-02-07  2004-02-12  2004-02-16
 0.02107873 -0.29823529  1.94078850  1.27384445  0.22170438 -2.07607585
 2004-02-20  2004-02-24
-1.78439244 -0.19533304
```

The `c` method simply calls `rbind` and hence behaves in the same way.

The `cbind` method by default combines the columns by the union of the indexes and fills the created gaps by `NA`s.

```
R> cbind(z1, z2)

                    z1          z2
2004-01-03          NA  0.94306673
2004-01-05  0.74675994 -0.04149429
2004-01-14  0.02107873          NA
2004-01-17          NA  0.59448077
2004-01-19 -0.29823529 -0.52575918
2004-01-24          NA -0.96739776
2004-01-25  0.68625772          NA
2004-01-27  1.94078850          NA
2004-02-07  1.27384445          NA
2004-02-08          NA  0.95605566
2004-02-12  0.22170438 -0.62733473
2004-02-13          NA -0.92845336
2004-02-16 -2.07607585          NA
2004-02-20 -1.78439244          NA
2004-02-24 -0.19533304          NA
2004-02-25          NA  0.56060280
2004-02-26          NA  0.08291711
```

In fact, the `cbind` method is synonymous with the `merge` method[8] except that the latter provides additional arguments which allow for combining the columns by the intersection of the indexes using the argument `all = FALSE`

```
R> merge(z1, z2, all = FALSE)

                   z1          z2
2004-01-05  0.7467599 -0.04149429
2004-01-19 -0.2982353 -0.52575918
2004-02-12  0.2217044 -0.62733473
```

[8]Note, that in some situations the column naming in the resulting object is somewhat problematic in the `cbind` method and the `merge` method might provide better formatting of the column names.

Additionally, the filling pattern can be changed in `merge`, the naming of the columns can be modified and the return class of the result can be specified. In the case of merging of objects with different index classes, R gives a warning and tries to coerce the indexes. Merging objects with different index classes is generally discouraged—if it is used nevertheless, it is the responsibility of the user to ensure that the result is as intended. If at least one of the merged/binded objects was a `"zooreg"` object, then `merge` tries to return a `"zooreg"` object. This is done by assessing whether there is a common maximal frequency and by checking whether the resulting index is still (weakly) regular.

If non-`"zoo"` objects are included in merging, then `merge` gives plain vectors/factors/matrices the index of the first argument (if it is of the same length). Scalars are always added for the full index without missing values.

```
R> merge(z1, pi, 1:10)

                   z1       pi 1:10
2004-01-05  0.74675994 3.141593    1
2004-01-14  0.02107873 3.141593    2
2004-01-19 -0.29823529 3.141593    3
2004-01-25  0.68625772 3.141593    4
2004-01-27  1.94078850 3.141593    5
2004-02-07  1.27384445 3.141593    6
2004-02-12  0.22170438 3.141593    7
2004-02-16 -2.07607585 3.141593    8
2004-02-20 -1.78439244 3.141593    9
2004-02-24 -0.19533304 3.141593   10
```

Another function which performs operations along a subset of indexes is `aggregate`, which is discussed in this section although it does not combine several objects. Using the `aggregate` method, `"zoo"` objects are split into subsets along a coarser index grid, summary statistics are computed for each and then the reduced object is returned. In the following example, first a function is set up which returns for a given `"Date"` value the corresponding first of the month. This function is then used to compute the coarser grid for the `aggregate` call: in the first example, the grouping is computed explicitely by `firstofmonth(index(Z))` and the mean of the observations in the month is returned—in the second example, only the function that computes the grouping (when applied to `index(Z)`) is supplied and the first observation is used for aggregation.

```
R> firstofmonth <- function(x) as.Date(sub("..$", "01", format(x)))
R> aggregate(Z, firstofmonth(index(Z)), mean)

                  Aa         Bb         Cc
2004-02-01 -0.1377964 0.40676219 -0.2376514
2004-03-01 -0.1667933 0.03905223 -0.2870087
```

```
R> aggregate(Z, firstofmonth, head, 1)
```

```
                  Aa        Bb         Cc
2004-02-01  1.255434 0.6815732 -0.6329205
2004-03-01 -1.208610 1.4237978 -0.8161448
```

The opposite of aggregation is disaggregation. For example, the `Nile` dataset is an annual `"ts"` class series. To disaggregate it into a quarterly series, convert it to a `"zoo` class series, insert intermediate quarterly points containing `NA` values and then fill the `NA` values using `na.approx`, `na.locf` or `na.spline`. (More details on `NA` handling in general can be found in Section 2.8.)

```
R> Nile.na <- merge(as.zoo(Nile),
+   zoo(, seq(start(Nile)[1], end(Nile)[1], 1/4)))
R> head(as.zoo(Nile))

1871 1872 1873 1874 1875 1876
1120 1160  963 1210 1160 1160

R> head(na.approx(Nile.na))

1871(1) 1871(2) 1871(3) 1871(4) 1872(1) 1872(2)
1120.00 1130.00 1140.00 1150.00 1160.00 1110.75

R> head(na.locf(Nile.na))

1871(1) 1871(2) 1871(3) 1871(4) 1872(1) 1872(2)
   1120    1120    1120    1120    1160    1160

R> head(na.spline(Nile.na))

 1871(1)  1871(2)  1871(3)  1871(4)  1872(1)  1872(2)
1120.000 1199.059 1224.985 1208.419 1160.000 1091.970
```

2.5. Mathematical operations

To allow for standard mathematical operations among `"zoo"` objects, **zoo** extends group generic functions `Ops`. These perform the operations only for the intersection of the indexes of the objects. As an example, the summation and logical comparison with $<$ of `z1` and `z2` yield

```
R> z1 + z2

2004-01-05 2004-01-19 2004-02-12
 0.7052657 -0.8239945 -0.4056304

R> z1 < z2
```

```
2004-01-05 2004-01-19 2004-02-12
     FALSE      FALSE      FALSE
```

Additionally, methods for transposing `t` of `"zoo"` objects—which coerces to a matrix before—and computing cumulative quantities such as `cumsum`, `cumprod`, `cummin`, `cummax` which are all applied column wise.

```
R> cumsum(Z)
```

```
                   Aa        Bb         Cc
2004-02-02  1.2554339 0.6815732 -0.6329205
2004-02-08 -0.2391494 2.0049854 -2.1273432
2004-02-09 -2.1137718 1.1316925 -1.5000035
2004-02-21 -2.2591579 1.5840415 -1.6459775
2004-02-22 -2.0337337 2.1224309 -1.4146162
2004-02-29 -0.8267785 2.4405731 -1.4259082
2004-03-05 -2.0353888 3.8643710 -2.2420530
2004-03-10 -2.1457844 5.2121135 -1.2868283
2004-03-14 -1.3037606 2.4736933 -1.0553214
2004-03-20 -1.4939516 2.5967820 -2.5739429
```

2.6. Extracting and replacing the data and the index

zoo provides several generic functions and methods to work on the data contained in a `"zoo"` object, the index (or time) attribute associated to it, and on both data and index.

The data stored in `"zoo"` objects can be extracted by `coredata` which strips off all `"zoo"`-specific attributes and it can be replaced using `coredata<-`. Both are new generic functions[9] with methods for `"zoo"` objects as illustrated in the following example.

```
R> coredata(z1)
```

```
 [1]  0.74675994  0.02107873 -0.29823529  0.68625772  1.94078850  1.27384445
 [7]  0.22170438 -2.07607585 -1.78439244 -0.19533304
```

```
R> coredata(z1) <- 1:10
R> z1
```

```
2004-01-05 2004-01-14 2004-01-19 2004-01-25 2004-01-27 2004-02-07 2004-02-12
         1          2          3          4          5          6          7
2004-02-16 2004-02-20 2004-02-24
         8          9         10
```

[9]The `coredata` functionality is similar in spirit to the `core` function in **its** and `value` in **tseries**. However, the focus of those functions is somewhat narrower and we try to provide more general purpose generic functions. See the respective manual page for more details.

The index associated with a "zoo" object can be extracted by index and modified by index<-. As the interpretation of the index as "time" in time series applications is natural, there are also synonymous methods time and time<-. Hence, the commands index(z2) and time(z2) return equivalent results.

```
R> index(z2)

 [1] "2004-01-03 GMT" "2004-01-05 GMT" "2004-01-17 GMT" "2004-01-19 GMT"
 [5] "2004-01-24 GMT" "2004-02-08 GMT" "2004-02-12 GMT" "2004-02-13 GMT"
 [9] "2004-02-25 GMT" "2004-02-26 GMT"
```

The index scale of z2 can be changed to that of z1 by

```
R> index(z2) <- index(z1)
R> z2

 2004-01-05  2004-01-14  2004-01-19  2004-01-25  2004-01-27  2004-02-07
 0.94306673 -0.04149429  0.59448077 -0.52575918 -0.96739776  0.95605566
 2004-02-12  2004-02-16  2004-02-20  2004-02-24
-0.62733473 -0.92845336  0.56060280  0.08291711
```

The start and the end of the index/time vector can be queried by start and end:

```
R> start(z1)

[1] "2004-01-05 GMT"

R> end(z1)

[1] "2004-02-24 GMT"
```

To work on both data and index/time, **zoo** provides window and window<- methods for "zoo" objects. In both cases the window is specified by

```
window(x, index, start, end)
```

where x is the "zoo" object, index is a set of indexes to be selected (by default the full index of x) and start and end can be used to restrict the index set.

```
R> window(Z, start = as.Date("2004-03-01"))

                   Aa         Bb         Cc
2004-03-05 -1.2086102  1.4237978 -0.8161448
2004-03-10 -0.1103956  1.3477425  0.9552247
2004-03-14  0.8420238 -2.7384202  0.2315069
2004-03-20 -0.1901910  0.1230887 -1.5186216
```

```
R> window(Z, index = index(Z)[5:8], end = as.Date("2004-03-01"))

                  Aa        Bb          Cc
2004-02-22 0.2254242 0.5383894  0.23136133
2004-02-29 1.2069552 0.3181422 -0.01129202
```

The first example selects all observations starting from 2004-03-01 whereas the second selects from the from the 5th to 8th observation those up to 2004-03-01.

The same syntax can be used for the corresponding replacement function.

```
R> window(z1, end = as.POSIXct("2004-02-01")) <- 9:5
R> z1

2004-01-05 2004-01-14 2004-01-19 2004-01-25 2004-01-27 2004-02-07 2004-02-12
         9          8          7          6          5          6          7
2004-02-16 2004-02-20 2004-02-24
         8          9         10
```

Two methods that are standard in time series applications are `lag` and `diff`. These are available with the same arguments as the `"ts"` methods.[10]

```
R> lag(z1, k = -1)

2004-01-14 2004-01-19 2004-01-25 2004-01-27 2004-02-07 2004-02-12 2004-02-16
         9          8          7          6          5          6          7
2004-02-20 2004-02-24
         8          9

R> merge(z1, lag(z1, k = 1))

           z1 lag(z1, k = 1)
2004-01-05  9              8
2004-01-14  8              7
2004-01-19  7              6
2004-01-25  6              5
2004-01-27  5              6
2004-02-07  6              7
2004-02-12  7              8
2004-02-16  8              9
2004-02-20  9             10
2004-02-24 10             NA

R> diff(z1)
```

[10] `diff` also has an additional argument that also allows for geometric and not only allows arithmetic differences. Furthermore, note the sign of the lag in `lag` which behaves like the `"ts"` method, i.e., by default it is positive and shifts the observations *forward*, to obtain the more standard *backward* shift the lag has to be negative.

```
2004-01-14 2004-01-19 2004-01-25 2004-01-27 2004-02-07 2004-02-12 2004-02-16
        -1         -1         -1         -1          1          1          1
2004-02-20 2004-02-24
         1          1
```

2.7. Coercion to and from "zoo"

Coercion to and from "zoo" objects is available for objects of various classes, in particular "ts", "irts" and "its" objects can be coerced to "zoo" and back if the index is of the appropriate class.[11]

Coercion between "zooreg" and "zoo" is also available and is essentially dropping the "frequency" attribute or trying to add one, respectively.

Furthermore, "zoo" objects can be coerced to vectors, matrices, lists and data frames (the latter dropping the index/time attribute). A simple example is

```
R> as.data.frame(Z)
```

```
                   Aa         Bb          Cc
2004-02-02  1.2554339  0.6815732 -0.63292049
2004-02-08 -1.4945833  1.3234122 -1.49442269
2004-02-09 -1.8746225 -0.8732929  0.62733971
2004-02-21 -0.1453861  0.4523490 -0.14597401
2004-02-22  0.2254242  0.5383894  0.23136133
2004-02-29  1.2069552  0.3181422 -0.01129202
2004-03-05 -1.2086102  1.4237978 -0.81614483
2004-03-10 -0.1103956  1.3477425  0.95522468
2004-03-14  0.8420238 -2.7384202  0.23150695
2004-03-20 -0.1901910  0.1230887 -1.51862157
```

2.8. NA handling

A wide range of methods for dealing with NAs (missing observations) in the observations are applicable to "zoo" objects including na.omit, na.contiguous, na.approx, na.spline, and na.locf among others. na.omit—or its default method to be more precise—returns a "zoo" object with incomplete observations removed. na.contiguous extracts the longest consecutive stretch of non-missing values. Furthermore, new generic functions na.approx, na.spline, and na.locf and corresponding default methods are introduced in **zoo**. The former two replace NAs by interpolation (using the function approx and spline, respectively) and the name of the latter stands for last observation carried forward. It replaces missing observations by the most recent non-NA prior to it. Leading NAs, which cannot be replaced by previous observations, are removed in both functions by default.

```
R> z1[sample(1:10, 3)] <- NA
R> z1
```

[11] Coercion from "zoo" to "irts" is contained in the **tseries** package.

```
2004-01-05 2004-01-14 2004-01-19 2004-01-25 2004-01-27 2004-02-07 2004-02-12 
         9         NA          7          6          5          6         NA 
2004-02-16 2004-02-20 2004-02-24 
         8          9         NA 
```

```
R> na.omit(z1)
```

```
2004-01-05 2004-01-19 2004-01-25 2004-01-27 2004-02-07 2004-02-16 2004-02-20 
         9          7          6          5          6          8          9 
```

```
R> na.contiguous(z1)
```

```
2004-01-19 2004-01-25 2004-01-27 2004-02-07 
         7          6          5          6 
```

```
R> na.approx(z1)
```

```
2004-01-05 2004-01-14 2004-01-19 2004-01-25 2004-01-27 2004-02-07 2004-02-12 
  9.000000   7.714286   7.000000   6.000000   5.000000   6.000000   7.111111 
2004-02-16 2004-02-20 
  8.000000   9.000000 
```

```
R> na.approx(z1, 1:NROW(z1))
```

```
2004-01-05 2004-01-14 2004-01-19 2004-01-25 2004-01-27 2004-02-07 2004-02-12 
         9          8          7          6          5          6          7 
2004-02-16 2004-02-20 
         8          9 
```

```
R> na.spline(z1)
```

```
2004-01-05 2004-01-14 2004-01-19 2004-01-25 2004-01-27 2004-02-07 2004-02-12 
  9.000000   6.766410   7.000000   6.000000   5.000000   6.000000   7.167209 
2004-02-16 2004-02-20 2004-02-24 
  8.000000   9.000000  10.157026 
```

```
R> na.locf(z1)
```

```
2004-01-05 2004-01-14 2004-01-19 2004-01-25 2004-01-27 2004-02-07 2004-02-12 
         9          9          7          6          5          6          6 
2004-02-16 2004-02-20 2004-02-24 
         8          9          9 
```

As the above example illustrates, `na.approx` (and also `na.spline`) use by default the underlying time scale for interpolation. This can be changed, e.g., to an equidistant spacing, by

setting the second argument of `na.approx`. Furthermore, a different output time index can be supplied as well.

In addition to the methods discussed above, there are also other methods for dealing with missing values in **zoo** such as `na.aggregate`, `na.fill`, `na.trim`, and `na.StructTS`.

2.9. Rolling functions

A typical task to be performed on ordered observations is to evaluate some function, e.g., computing the mean, in a window of observations that is moved over the full sample period. The resulting statistics are usually synonymously referred to as rolling/running/moving statistics. In **zoo**, the generic function `rollapply`[12] is provided along with a `"zoo"` and a `"ts"` method. The most important arguments are

```
rollapply(data, width, FUN)
```

where the function `FUN` is applied to a rolling window of size `width` of the observations `data`. The function `rollapply` by default only evaluates the function for windows of full size `width` and then the result has `width - 1` fewer observations than the original series and is aligned at the center of the rolling window. Setting further arguments such as `partial`, `align`, or `fill` also allows for rolling computations on partial windows with arbitrary aligning and flexible filling. For example, without partial evaluation the 'lost' observations could be filled with `NA`s and aligned at the left of the sample.

```
R> rollapply(Z, 5, sd)

                  Aa        Bb        Cc
2004-02-09 1.2814876 0.8018950 0.8218959
2004-02-21 1.2658555 0.7891358 0.8025043
2004-02-22 1.2102011 0.8206819 0.5319727
2004-02-29 0.8662296 0.5266261 0.6411751
2004-03-05 0.9363400 1.7011273 0.6356144
2004-03-10 0.9508642 1.6892246 0.9578196

R> rollapply(Z, 5, sd, fill = NA, align = "left")

                  Aa        Bb        Cc
2004-02-02 1.2814876 0.8018950 0.8218959
2004-02-08 1.2658555 0.7891358 0.8025043
2004-02-09 1.2102011 0.8206819 0.5319727
2004-02-21 0.8662296 0.5266261 0.6411751
2004-02-22 0.9363400 1.7011273 0.6356144
2004-02-29 0.9508642 1.6892246 0.9578196
2004-03-05        NA        NA        NA
```

[12]In previous versions of **zoo**, this function was called `rapply`. It was renamed because from R 2.4.0 on, base R provides a different function `rapply` for recursive (and not rolling) application of functions. The function `zoo::rapply` is still provided for backward compatibility, however it dispatches now to `rollapply` methods.

```
2004-03-10         NA         NA         NA
2004-03-14         NA         NA         NA
2004-03-20         NA         NA         NA
```

To improve the performance of `rollapply(x, k, `*foo*`)` for some frequently used functions *foo*, more efficient implementations `roll`*foo*`(x, k)` are available (and also called by `rollapply`). Currently, these are the generic functions `rollmean`, `rollmedian` and `rollmax` which have methods for `"zoo"` and `"ts"` series and a default method for plain vectors.

```
R> rollmean(z2, 5, fill = NA)

   2004-01-05    2004-01-14    2004-01-19    2004-01-25    2004-01-27 
           NA            NA  0.0005792538  0.0031770388 -0.1139910497 
   2004-02-07    2004-02-12    2004-02-16    2004-02-20    2004-02-24 
-0.4185778750 -0.2013054791  0.0087574946            NA            NA 
```

3. Combining zoo with other packages

The main purpose of the package **zoo** is to provide basic infrastructure for working with indexed totally ordered observations that can be either employed by users directly or can be a basic ingredient on top of which other packages can build. The latter is illustrated with a few brief examples involving the packages **strucchange**, **tseries** and **timeDate**/**fCalendar** in this section. Finally, the classes `"yearmon"` and `"yearqtr"` (provided in **zoo**) are used for illustrating how **zoo** can be extended by creating a new index class.

3.1. strucchange: Empirical fluctuation processes

The package **strucchange** provides a collection of methods for testing, monitoring and dating structural changes, in particular in linear regression models. Tests for structural change assess whether the parameters of a model remain constant over an ordering with respect to a specified variable, usually time. To adequately store and visualize empirical fluctuation processes which capture instabilities over this ordering, a data type for indexed ordered observations is required. This was the motivation for starting the **zoo** project.

A simple example for the need of `"zoo"` objects in **strucchange** which can not be (easily) implemented by other irregular time series classes available in R is described in the following. We assess the constancy of the electrical resistance over the apparent juice content of kiwi fruits.[13] The data set `fruitohms` is contained in the **DAAG** package (Maindonald and Braun 2009). The fitted `ocus` object contains the OLS-based CUSUM process for the mean of the electrical resistance (variable `ohms`) indexed by the juice content (variable `juice`).

```
R> library("strucchange")
R> library("DAAG")
R> data("fruitohms")
R> ocus <- gefp(ohms ~ 1, order.by = ~ juice, data = fruitohms)
```

[13]A different approach would be to test whether the slope of a regression of electrical resistance on juice content changes with increasing juice content, i.e., to test for instabilities in `ohms ~ juice` instead of `ohms ~ 1`. Both lead to similar results.

```
R> plot(ocus)
```

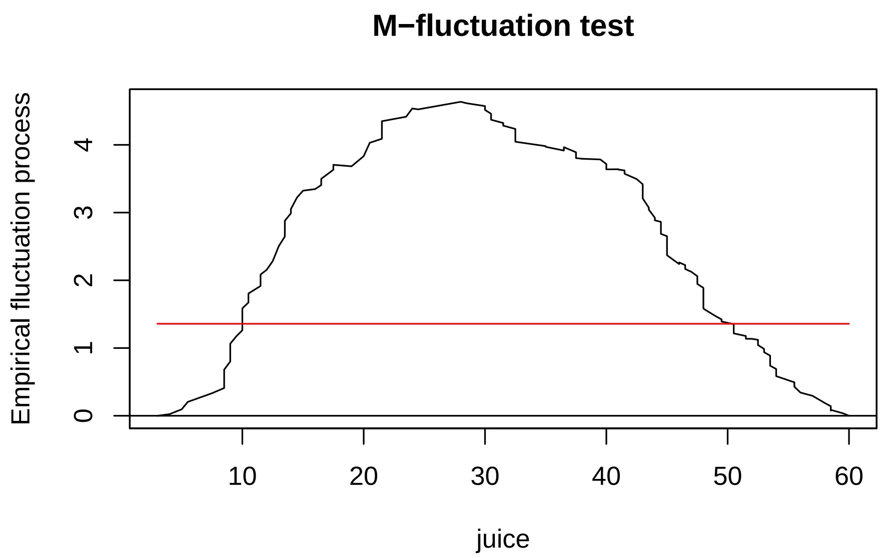

Figure 3: Empirical M-fluctuation process for `fruitohms` data

This OLS-based CUSUM process can be visualized using the `plot` method for `"gefp"` objects which builds on the `"zoo"` method and yields in this case the plot in Figure 3 showing the process which crosses its 5% critical value and thus signals a significant decrease in the mean electrical resistance over the juice content. For more information on the package **strucchange** and the function `gefp` see Zeileis *et al.* (2002) and Zeileis (2006).

3.2. tseries: Historical financial data

This section was written when ***tseries*** *did not yet support* `"zoo"` *series directly. For historical reasons and completeness, the example is still included but for practical purposes it is not relevant anymore because, from version 0.9-30 on,* `get.hist.quote` *returns a* `"zoo"` *series by default.*

A typical application for irregular time series which became increasingly important over the last years in computational statistics and finance is daily (or higher frequency) financial data. The package **tseries** provides the function `get.hist.quote` for obtaining historical financial data by querying Yahoo! Finance at `http://finance.yahoo.com/`, an online portal quoting data provided by Reuters. The following code queries the quotes of Microsoft Corp. starting from 2001-01-01 until 2004-09-30:

```
R> library("tseries")
R> MSFT <- get.hist.quote(instrument = "MSFT", start = "2001-01-01",
+   end = "2004-09-30", origin = "1970-01-01", retclass = "ts")
```

In the returned `MSFT` object the irregular data is stored by extending it in a regular grid and filling the gaps with `NA`s. The time is stored in days starting from an `origin`, in this case

specified to be 1970-01-01, the origin used by the "Date" class. This series can be transformed easily into a "zoo" series using a "Date" index.

```
R> MSFT <- as.zoo(MSFT)
R> index(MSFT) <- as.Date(index(MSFT))
R> MSFT <- na.omit(MSFT)
```

Because this is daily data, the series has a natural underlying regularity. Thus, `as.zoo()` returns a "zooreg" object by default. To treat it as an irregular series `as.zoo()` can be applied a second time, yielding a "zoo" series. The corresponding log-difference returns are depicted in Figure 4.

```
R> MSFT <- as.zoo(MSFT)
```

3.3. timeDate/fCalendar: Indexes of class "timeDate"

The original version of this section was written when ***fCalendar*** *(now:* ***timeDate****) and* ***zoo*** *did not yet include enough methods to attach* "timeDate" *indexes to* "zoo" *series. For historical reasons and completeness, we still briefly comment on the communcation between the packages and their classes.*

Although the methods in **zoo** work out of the box for many index classes, it might be necessary for some index classes to provide `c()`, `length()`, `[`, `ORDER()` and `MATCH()` methods such that the methods in **zoo** work properly. Previously, this was the case "timeDate" from the **fCalendar** package which is why it was used as an example in this vigntte. Meanwhile however, both **zoo** and **fCalendar**/**timeDate** have been enhanced: The latter contains the methods for `c()`, `length()`, and `[`, while **zoo** has methods for `ORDER()` and `MATCH()` for class "timeDate". The last two functions essentially work by coercing to the underlying "POSIXct" and then using the associated methods.

The following example illustrates how `z2` can be transformed to use the "timeDate" class.

```
R> library("timeDate")
R> z2td <- zoo(coredata(z2), timeDate(index(z2), FinCenter = "GMT"))
R> z2td

 2004-01-05  2004-01-14  2004-01-19  2004-01-25  2004-01-27  2004-02-07
 0.94306673 -0.04149429  0.59448077 -0.52575918 -0.96739776  0.95605566
 2004-02-12  2004-02-16  2004-02-20  2004-02-24
-0.62733473 -0.92845336  0.56060280  0.08291711
```

3.4. The classes "yearmon" and "yearqtr": Roll your own index

One of the strengths of the **zoo** package is its independence of the index class, such that the index can be easily customized. The previous section already explained how an existing class ("timeDate") can be used as the index if the necessary methods are created. This section has a similar but slightly different focus: it describes how new index classes can be created

```
R> plot(diff(log(MSFT)))
```

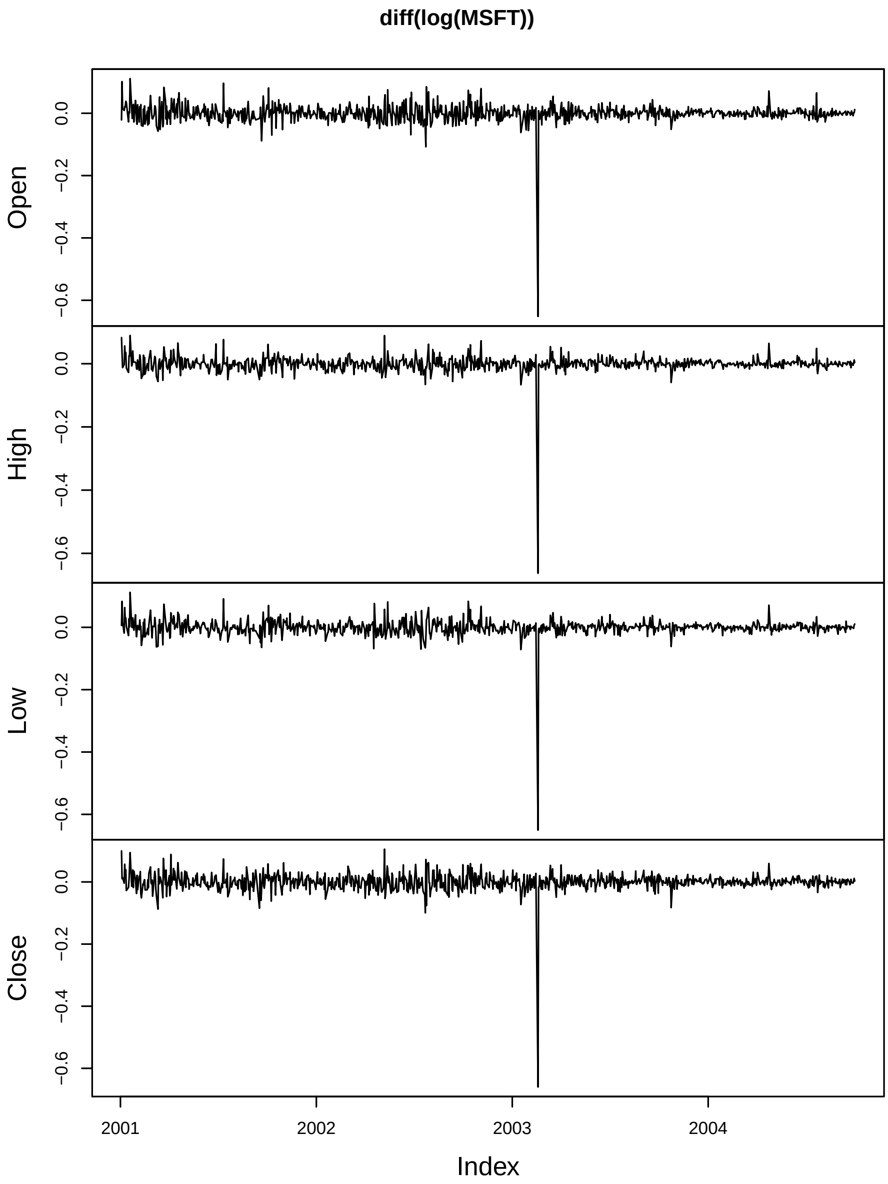

Figure 4: Log-difference returns for Microsoft Corp.

addressing a certain type of indexes. These classes are `"yearmon"` and `"yearqtr"` (already contained in **zoo**) which provide indexes for monthly and quarterly data respectively. As the code is virtually identical for both classes—except that one has the frequency 12 and the other 4—we will only discuss `"yearmon"` explicitly.

Of course, monthly data can simply be stored using a numeric index just as the class `"ts"` does. The problem is that this does not have the meta-information attached that this is really

specifying monthly data which is in "yearmon" simply added by a class attribute. Hence, the class creator is simply defined as

```
yearmon <- function(x) structure(floor(12*x + .0001)/12, class = "yearmon")
```

which is very similar to the `as.yearmon` coercion functions provided.

As "yearmon" data is now explicitly declared to describe monthly data, this can be exploited for coercion to other time classes: either to coarser time scales such as "yearqtr" or to finer time scales such as "Date", "POSIXct" or "POSIXlt" which by default associate the first day within a month with a "yearmon" observation. Adding a `format` and `as.character` method produces human readable character representations of "yearmon" data and `Ops` and `MATCH` methods complete the methods needed for conveniently working with monthly data in **zoo**. Note, that all of these methods are very simple and rather obvious (as can be seen in the **zoo** sources), but prove very helpful in the following examples.

First, we create a regular series `zr3` with "yearmon" index which leads to improved printing compared to the regular series `zr1` and `zr2` from Section 2.2.

```
R> zr3 <- zooreg(rnorm(9), start = as.yearmon(2000), frequency = 12)
R> zr3

   Jan 2000    Feb 2000    Mar 2000    Apr 2000    May 2000    Jun 2000
-0.30969096  0.08699142 -0.64837101 -0.62786277 -0.61932674 -0.95506154
   Jul 2000    Aug 2000    Sep 2000
-1.91736406  0.38108885  1.51405511
```

This could be aggregated to quarterly data via

```
R> aggregate(zr3, as.yearqtr, mean)

   2000 Q1    2000 Q2    2000 Q3
-0.2903569 -0.7340837 -0.0074067
```

The index can easily be transformed to "Date", the default being the first day of the month but which can also be changed to the last day of the month.

```
R> as.Date(index(zr3))

[1] "2000-01-01" "2000-02-01" "2000-03-01" "2000-04-01" "2000-05-01"
[6] "2000-06-01" "2000-07-01" "2000-08-01" "2000-09-01"
```

```
R> as.Date(index(zr3), frac = 1)

[1] "2000-01-31" "2000-02-29" "2000-03-31" "2000-04-30" "2000-05-31"
[6] "2000-06-30" "2000-07-31" "2000-08-31" "2000-09-30"
```

Furthermore, "yearmon" indexes can easily be coerced to "POSIXct" such that the series could be exported as a "its" or "irts" series.

```
R> index(zr3) <- as.POSIXct(index(zr3))
R> as.irts(zr3)
```

```
2000-01-01 00:00:00 GMT -0.3097
2000-02-01 00:00:00 GMT 0.08699
2000-03-01 00:00:00 GMT -0.6484
2000-04-01 00:00:00 GMT -0.6279
2000-05-01 00:00:00 GMT -0.6193
2000-06-01 00:00:00 GMT -0.9551
2000-07-01 00:00:00 GMT -1.917
2000-08-01 00:00:00 GMT 0.3811
2000-09-01 00:00:00 GMT 1.514
```

Again, this functionality makes switching between different time scales or index representations particularly easy and **zoo** provides the user with the flexibility to adjust a certain index to his/her problem of interest.

4. Summary and outlook

The package **zoo** provides an S3 class and methods for indexed totally ordered observations, such as both regular and irregular time series. Its key design goals are independence of a particular index class and compatibility with standard generics similar to the behaviour of the corresponding "ts" methods. This paper describes how these are implemented in **zoo** and illustrates the usage of the methods for plotting, merging and binding, several mathematical operations, extracting and replacing data and index, coercion and NA handling.

An indexed object of class "zoo" can be thought of as data plus index where the data are essentially vectors or matrices and the index can be a vector of (in principle) arbitrary class. For (weakly) regular "zooreg" series, a "frequency" attribute is stored in addition. Therefore, objects of classes "ts", "its", "irts" and "timeSeries" can easily be transformed into "zoo" objects—the reverse transformation is also possible provided that the index fulfills the restrictions of the respective class. Hence, the "zoo" class can also be used as the basis for other classes of indexed observations and more specific functionality can be built on top of it. Furthermore, it bridges the gap between irregular and regular series, facilitating operations such as NA handling compared to "ts".

Whereas a lot of effort was put into achieving independence of a particular index class, the types of data that can be indexed with "zoo" are currently limited to vectors and matrices, typically containing numeric values. Although, there is some limited support available for indexed factors, one important direction for future development of **zoo** is to add better support for other objects that can also naturally be indexed including specifically factors, data frames and lists.

Computational details

The results in this paper were obtained using R 3.1.2 with the packages **zoo** 1.7–12, **strucchange** 1.5–0, **timeDate** 3010.98, **tseries** 0.10–34 and **DAAG** 1.20. R itself and all packages used are available from CRAN at http://CRAN.R-project.org/.

References

Heywood G (2009). ***its**: Irregular Time Series.* Portfolio & Risk Advisory Group and Commerzbank Securities. R package version 1.1.8, URL http://CRAN.R-project.org/package=its.

Maindonald J, Braun WJ (2009). ***DAAG**: Data Analysis and Graphics.* R package version 1.01, URL http://CRAN.R-project.org/package=DAAG.

R Development Core Team (2008). *R: A Language and Environment for Statistical Computing.* R Foundation for Statistical Computing, Vienna, Austria. ISBN 3-900051-07-0, URL http://www.R-project.org/.

Ryan JA, Ulrich JM (2010). ***xts**: Extensible Time Series.* R package version 0.7-1, URL http://CRAN.R-project.org/package=xts.

Trapletti A (2009). ***tseries**: Time Series Analysis and Computational Finance.* R package version 0.10-22, URL http://CRAN.R-project.org/package=tseries.

Wuertz D (2010). ***Rmetrics**: An Environment and Software Collection for Teaching Financial Engineering and Computational Finance.* R packages **fArma**, **fAsianOptions**, **fAssets**, **fBasics**, **fCalendar**, **fCopulae**, **fEcofin**, **fExoticOptions**, **fExtremes**, **fGarch**, **fImport**, **fMultivar**, **fNonlinear**, **fOptions**, **fPortfolio**, **fRegression**, **fSeries**, **fTrading**, **fUnitRoots**, **fUtilities**, URL http://www.Rmetrics.org/.

Zeileis A (2006). "Implementing a Class of Structural Change Tests: An Econometric Computing Approach." *Computational Statistics & Data Analysis*, **50**, 2987–3008. doi:10.1016/j.csda.2005.07.001.

Zeileis A, Grothendieck G (2005). "**zoo**: S3 Infrastructure for Regular and Irregular Time Series." *Journal of Statistical Software*, **14**(6), 1–27. URL http://www.jstatsoft.org/v14/i06/.

Zeileis A, Leisch F, Hornik K, Kleiber C (2002). "**strucchange**: An R Package for Testing for Structural Change in Linear Regression Models." *Journal of Statistical Software*, **7**(2), 1–38. URL http://www.jstatsoft.org/v07/i02/.

A. Reference card

Creation

`zoo(x, order.by)`	creation of a `"zoo"` object from the observations `x` (a vector or a matrix) and an index `order.by` by which the observations are ordered. For computations on arbitrary index classes, methods to the following generic functions are assumed to work: combining `c()`, querying length `length()`, subsetting `[`, ordering `ORDER()` and value matching `MATCH()`. For pretty printing an `as.character` and/or `index2char` method might be helpful.

Creation of regular series

`zoo(x, order.by, freq)`	works as above but creates a `"zooreg"` object which inherits from `"zoo"` if the frequency `freq` complies with the index `order.by`. An `as.numeric` method has to be available for the index class.
`zooreg(x, start, end, freq)`	creates a `"zooreg"` series with a numeric index as above and has (almost) the same interface as `ts()`.

Standard methods

`plot`	plotting
`lines`	adding a `"zoo"` series to a plot
`print`	printing
`summary`	summarizing (column-wise)
`str`	displaying structure of `"zoo"` objects
`head`, `tail`	head and tail of `"zoo"` objects

Coercion

`as.zoo`	coercion to `"zoo"` is available for objects of class `"ts"`, `"its"`, `"irts"` (plus a default method).
`as.`*class*`.zoo`	coercion from `"zoo"` to other classes. Currently available for *class* in `"matrix"`, `"vector"`, `"data.frame"`, `"list"`, `"irts"`, `"its"` and `"ts"`.
`is.zoo`	querying wether an object is of class `"zoo"`

Merging and binding

`merge`	union, intersection, left join, right join along indexes
`cbind`	column binding along the intersection of the index
`c`, `rbind`	combining/row binding (indexes may not overlap)
`aggregate`	compute summary statistics along a coarser grid of indexes

Mathematical operations

`Ops`	group generic functions performed along the intersection of indexes
`t`	transposing (coerces to `"matrix"` before)
`cumsum`	compute (columnwise) cumulative quantities: sums `cumsum()`, products `cumprod()`, maximum `cummax()`, minimum `cummin()`.

Extracting and replacing data and index

`index, time`	extract the index of a series
`index<-`, `time<-`	replace the index of a series
`coredata`, `coredata<-`	extract and replace the data associated with a `"zoo"` object
`lag`	lagged observations
`diff`	arithmetic and geometric differences
`start, end`	querying start and end of a series
`window, window<-`	subsetting of `"zoo"` objects using their index

`NA` handling

`na.omit`	omit `NA`s
`na.contiguous`	compute longest sequence of non-`NA` observations
`na.locf`	impute `NA`s by carrying forward the last observation
`na.approx`	impute `NA`s by interpolation
`na.trim`	remove leading and/or trailing `NA`s

Rolling functions

`rollapply`	apply a function to rolling margin of an array
`rollmean`	more efficient functions for computing the rolling mean, median and maximum are `rollmean()`, `rollmedian()` and `rollmax()`, respectively

Methods for regular series

`is.regular`	checks whether a series is weakly (or strictly if `strict = TRUE`) regular
`frequency`, `deltat`	extracts the frequency or its reciprocal value respectively from a series, for `"zoo"` series the functions try to determine the regularity and frequency in a data-driven way
`cycle`	gives the position in the cycle of a regular series

Affiliation:

Achim Zeileis
Universität Innsbruck
E-mail: Achim.Zeileis@R-project.org

Gabor Grothendieck
GKX Associates Inc.
E-mail: ggrothendieck@gmail.com
