# Supplementary material for: Comparing Kaolin and Pinolene to Improve Sustainable Grapevine Production during Drought
Source: PLoS One. 2016 Jun 13;11(6):e0156631. doi: 10.1371/journal.pone.0156631 (PMC4905681; doi:10.1371/journal.pone.0156631)
Supplement: S1 File — (GZ) [file pone.0156631.s003.gz › PONE_Brillante_et_al_2016_Code_and_Data/packrat/src/zoo/zoo_1.7-12.tar.gz!/zoo/vignettes/zoo-refcard.pdf]

zoo reference card

Creation

`zoo(x, order.by)`	creation of a `"zoo"` object from the observations `x` (a vector or a matrix) and an index `order.by` by which the observations are ordered.<br>For computations on arbitrary index classes, methods to the following generic functions are assumed to work: combining `c()`, querying length `length()`, subsetting `[`, ordering `ORDER()` and value matching `MATCH()`. For pretty printing an `as.character` and/or `index2char` method might be helpful.

Creation of regular series

`zoo(x, order.by, freq)`	works as above but creates a `"zooreg"` object which inherits from `"zoo"` if the frequency `freq` complies with the index `order.by`. An `as.numeric` method has to be available for the index class.
`zooreg(x, start, end, freq)`	creates a `"zooreg"` series with a numeric index as above and has (almost) the same interface as `ts()`.

Standard methods

`plot`	plotting
`lines`	adding a `"zoo"` series to a plot
`print`	printing
`summary`	summarizing (column-wise)
`str`	displaying structure of `"zoo"` objects
`head`, `tail`	head and tail of `"zoo"` objects

Coercion

`as.zoo`	coercion to `"zoo"` is available for objects of class `"ts"`, `"its"`, `"irts"` (plus a default method).
`as.`*class*`.zoo`	coercion from `"zoo"` to other classes. Currently available for *class* in `"matrix"`, `"vector"`, `"data.frame"`, `"list"`, `"irts"`, `"its"` and `"ts"`.
`is.zoo`	querying wether an object is of class `"zoo"`

Merging and binding

`merge`	union, intersection, left join, right join along indexes
`cbind`	column binding along the intersection of the index
`c`, `rbind`	combining/row binding (indexes may not overlap)
`aggregate`	compute summary statistics along a coarser grid of indexes

Mathematical operations

`Ops`	group generic functions performed along the intersection of indexes
`t`	transposing (coerces to `"matrix"` before)
`cumsum`	compute (columnwise) cumulative quantities: sums `cumsum()`, products `cumprod()`, maximum `cummax()`, minimum `cummin()`.

Extracting and replacing data and index

`index`, `time`	extract the index of a series
`index<-`, `time<-`	replace the index of a series
`coredata`, `coredata<-`	extract and replace the data associated with a `"zoo"` object
`lag`	lagged observations
`diff`	arithmetic and geometric differences
`start`, `end`	querying start and end of a series
`window`, `window<-`	subsetting of `"zoo"` objects using their index

`NA` handling

`na.omit`	omit `NA`s
`na.contiguous`	compute longest sequence of non-`NA` observations
`na.locf`	impute `NA`s by carrying forward the last observation
`na.approx`	impute `NA`s by interpolation
`na.trim`	remove leading and/or trailing `NA`s

Rolling functions

`rollapply`	apply a function to rolling margin of an array
`rollmean`	more efficient functions for computing the rolling mean, median and maximum are `rollmean()`, `rollmedian()` and `rollmax()`, respectively

Methods for regular series

`is.regular`	checks whether a series is weakly (or strictly if `strict = TRUE`) regular
`frequency`, `deltat`	extracts the frequency or its reciprocal value respectively from a series, for `"zoo"` series the functions try to determine the regularity and frequency in a data-driven way
`cycle`	gives the position in the cycle of a regular series
